# *De novo* assembly and functional annotation of *Myrciaria dubia* fruit transcriptome reveals multiple metabolic pathways for L-ascorbic acid biosynthesis

**DOI:** 10.1186/s12864-015-2225-6

**Published:** 2015-11-24

**Authors:** Juan C. Castro, J. Dylan Maddox, Marianela Cobos, David Requena, Mirko Zimic, Aureliano Bombarely, Sixto A. Imán, Luis A. Cerdeira, Andersson E. Medina

**Affiliations:** Unidad Especializada de Biotecnología, Centro de Investigaciones de Recursos Naturales de la Amazonía (CIRNA), Universidad Nacional de la Amazonía Peruana (UNAP), Pasaje Los Paujiles S/N, San Juan Bautista, Iquitos, Perú; Círculo de Investigación en Plantas con Efecto en Salud (FONDECYT N° 010–2014), Lima, Perú; Pritzker Laboratory for Molecular Systematics and Evolution, The Field Museum of Natural History, Chicago, IL USA; Laboratorio de Biotecnología y Bioenergética, Universidad Científica del Perú (UCP), Av. Abelardo Quiñones km 2.5, San Juan Bautista, Iquitos, Perú; Laboratorio de Bioinformática y Biología Molecular, Laboratorios de Investigación y Desarrollo (LID), Facultad de Ciencias, Universidad Peruana Cayetano Heredia (UPCH), Av. Honorio Delgado 430, San Martín de Porres, Lima, Perú; FARVET S.A.C. Carretera Panamericana Sur N° 766 Km 198.5, Chincha Alta, Ica Perú; Department of Horticulture, Virginia Tech, Blacksburg, VA 24061 USA; Área de Conservación de Recursos Fitogenéticos, Instituto Nacional de Innovación Agraria (INIA), Calle San Roque 209, Iquitos, Perú

**Keywords:** Camu-camu, Metabolic pathway reconstruction, Next-generation sequencing, Plant vitamin C metabolism

## Abstract

**Background:**

*Myrciaria dubia* is an Amazonian fruit shrub that produces numerous bioactive phytochemicals, but is best known by its high L-ascorbic acid (AsA) content in fruits. Pronounced variation in AsA content has been observed both within and among individuals, but the genetic factors responsible for this variation are largely unknown. The goals of this research, therefore, were to assemble, characterize, and annotate the fruit transcriptome of *M. dubia* in order to reconstruct metabolic pathways and determine if multiple pathways contribute to AsA biosynthesis.

**Results:**

In total 24,551,882 high-quality sequence reads were *de novo* assembled into 70,048 unigenes (mean length = 1150 bp, N50 = 1775 bp). Assembled sequences were annotated using BLASTX against public databases such as TAIR, GR-protein, FB, MGI, RGD, ZFIN, SGN, WB, TIGR_CMR, and JCVI-CMR with 75.2 % of unigenes having annotations. Of the three core GO annotation categories, biological processes comprised 53.6 % of the total assigned annotations, whereas cellular components and molecular functions comprised 23.3 and 23.1 %, respectively. Based on the KEGG pathway assignment of the functionally annotated transcripts, five metabolic pathways for AsA biosynthesis were identified: animal-like pathway, myo-inositol pathway, L-gulose pathway, D-mannose/L-galactose pathway, and uronic acid pathway. All transcripts coding enzymes involved in the ascorbate-glutathione cycle were also identified. Finally, we used the assembly to identified 6314 genic microsatellites and 23,481 high quality SNPs.

**Conclusions:**

This study describes the first next-generation sequencing effort and transcriptome annotation of a non-model Amazonian plant that is relevant for AsA production and other bioactive phytochemicals. Genes encoding key enzymes were successfully identified and metabolic pathways involved in biosynthesis of AsA, anthocyanins, and other metabolic pathways have been reconstructed. The identification of these genes and pathways is in agreement with the empirically observed capability of *M. dubia* to synthesize and accumulate AsA and other important molecules, and adds to our current knowledge of the molecular biology and biochemistry of their production in plants. By providing insights into the mechanisms underpinning these metabolic processes, these results can be used to direct efforts to genetically manipulate this organism in order to enhance the production of these bioactive phytochemicals.

The accumulation of AsA precursor and discovery of genes associated with their biosynthesis and metabolism in *M. dubia* is intriguing and worthy of further investigation. The sequences and pathways produced here present the genetic framework required for further studies. Quantitative transcriptomics in concert with studies of the genome, proteome, and metabolome under conditions that stimulate production and accumulation of AsA and their precursors are needed to provide a more comprehensive view of how these pathways for AsA metabolism are regulated and linked in this species.

**Electronic supplementary material:**

The online version of this article (doi:10.1186/s12864-015-2225-6) contains supplementary material, which is available to authorized users.

## Background

*Myrciaria dubia* (Kunth) McVaugh “camu-camu” is an diploid Amazonian plant species with 2*n* = 22 chromosomes [1] that produces numerous bioactive phytochemicals [2–6], but is best known by its high Vitamin C (L-ascorbic acid) content in fruits [7], which can contain as much as 2 g of L-ascorbic acid (AsA) per 100 g of fruit pulp [8], which is equivalent to 50 times the AsA content of orange juice [9]. Pronounced variation in AsA content among different tissue types in the same individual and among individuals has been observed [10], but the genetic factors responsible for AsA content variation in this species are largely unknown.

Results from our research group have demonstrated that *M. dubia* possesses the capability for AsA biosynthesis in several tissues (unpublished data), and that the large variation of this bioactive molecule in the leaves and fruit pulp and peel is likely due, in part, to differential gene expression and enzyme activities in the D-mannose/L-galactose pathway [11]. In other plant species, radiolabelling, mutant analysis, and transgenic manipulation have provided evidence for the occurrence of multiple metabolic pathways of AsA biosynthesis [12, 13]. It is therefore reasonable to hypothesize that AsA pool size in *M. dubia* is also the result of multiple metabolic pathways and that their identification and understanding may ultimately explain the large variation observed in AsA content.

Recent advances in high-throughput next-generation sequencing and bioinformatics tools have been used successfully to reveal the transcriptome and identify metabolic pathways in several plant species [14–18]. In this study, we present the sequencing, assembly, and annotation of the fruit transcriptome of *M. dubia* in order to reconstruct metabolic pathways and identify those associated with AsA biosynthesis.

## Results

### Illumina paired end sequencing and *de novo* assembly

**Fig. 1:**
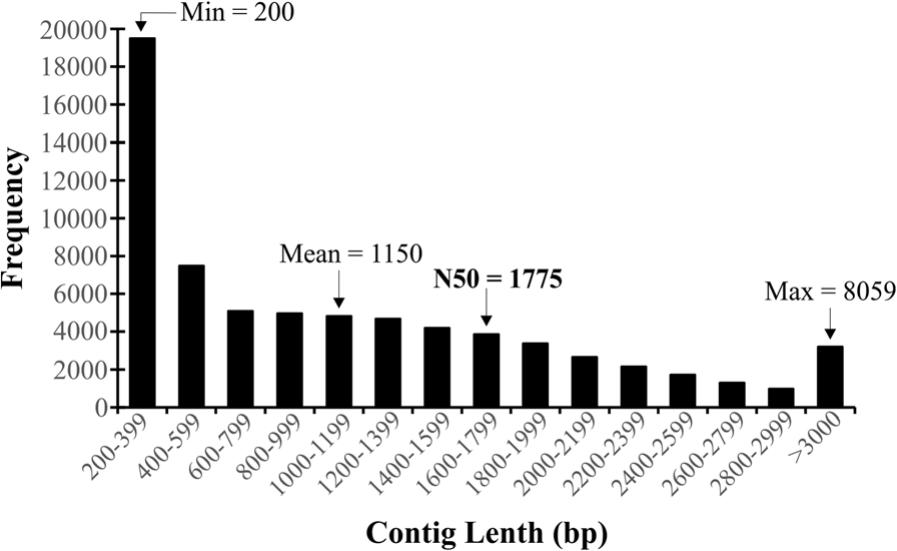
Distribution of unigene lengths after de novo transcriptome assembly of fruits from *M. dubia*

A total of 25,787,070 raw sequencing reads of 100 bp were generated from a 200 bp insert library. After raw reads were filtered and cleaned, 24,551,882 (95.2 %) high-quality reads were used to assemble the fruit transcriptome of *M. dubia*. A total of 70,048 unigenes were generated in the meta-assembly with a length between 200 and 8059 bp, mean length of 1150 bp, and a N50 of 1775 bp (Fig. 1). The Illumina paired-end reads have been submitted to the Short Read Archive [SRA: SRR1630824].

### Functional annotation, and metabolic pathway assignments

**Fig. 2:**
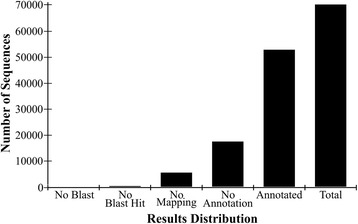
Distribution of Blast2GO three-step processes including BLASTX, mapping, and annotation of *de novo M. dubia* fruit transcriptome meta-assembly

**Fig. 3:**
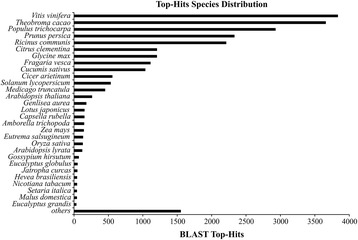
Top-hits species distribution based on BLASTX alignments in the *M. dubia* fruit transcriptome meta-assembly

All unigenes were included in the annotation process. This process included best BLASTX match selection, Gene Ontology ID assignment, enzyme code assignments and InterPro domains calculation. BLASTX comparison with the NCBI nonredundant (nr) database revealed 52,707 (75.2 %) unigenes with annotations (Fig. 2). A significant amount of mapping data (93.0 % of unigenes with mapping information) was derived from UniProtKB database followed by TAIR and GR_protein. Additional databases (i.e., FB, MGI, RGD, ZFIN, SGN, WB, TIGR_CMR, and JCVI-CMR) were searched but did not contribute to the mapping process. The top five species that contributed the greatest number of gene annotations from BLASTx were *Vitis vinifera*, *Theobroma cacao*, *Populus trichocarpa*, *Prunus persica*, and *Ricinus communis* (Fig. 3).

**Fig. 4:**
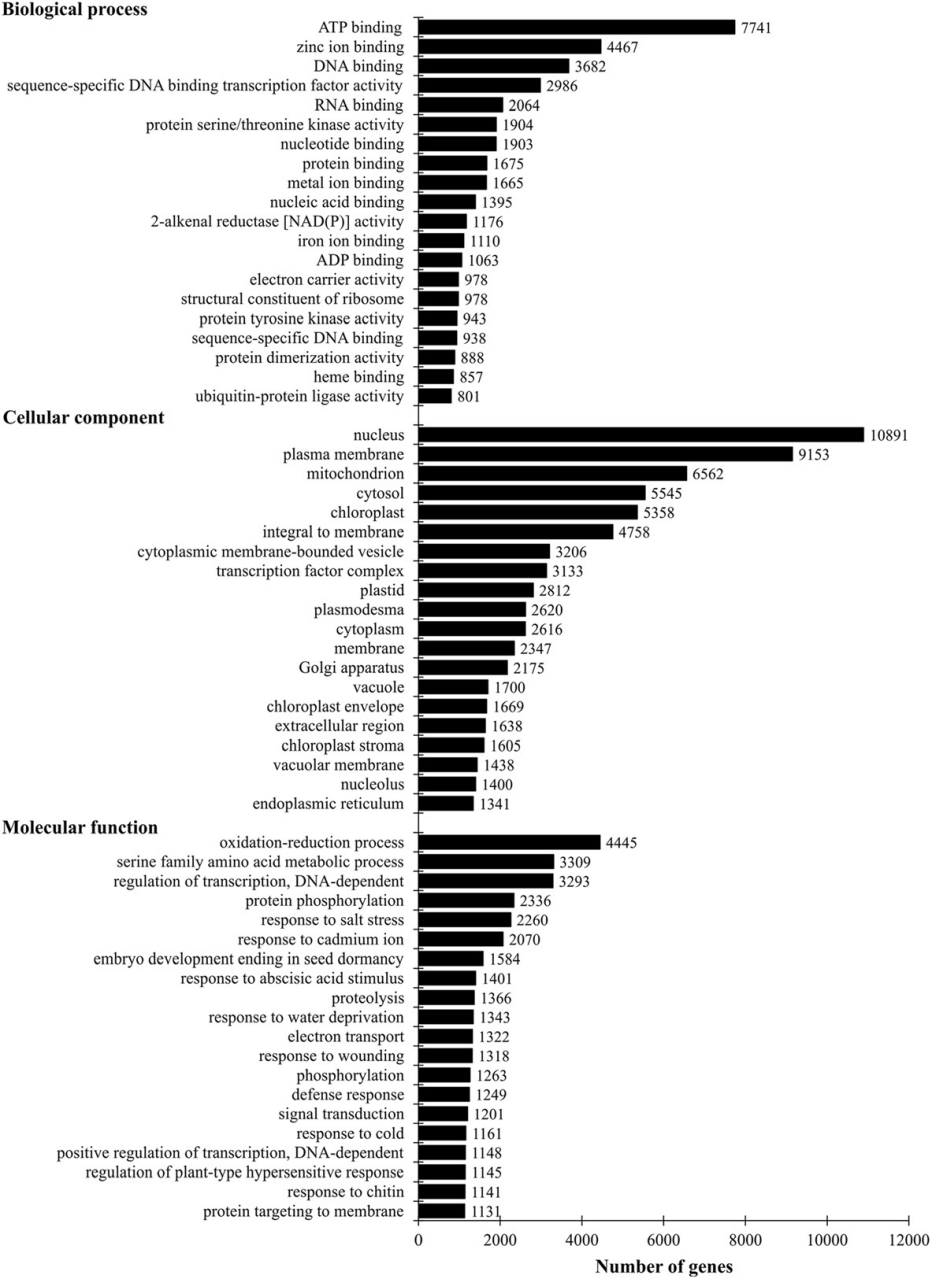
Gene Ontology classifications of assembled sequences. Numbers indicate the number of sequences associated with the particulate GO term

Of the three core GO annotation categories, biological processes (BP) comprised 53.6 % of the total assigned annotations, whereas cellular components (CC) and molecular functions (MF) comprised 23.3 % and 23.1 %, respectively. The GO terms with the largest number of assigned sequences in the BP category were ATP binding (7,741; 3.5 %), zinc ion binding (4,467; 2.0 %), DNA binding (3682; 1.7 %), and sequence-specific DNA binding transcription factor activity (2,986; 1.3 %) (Fig. 4). For CC the terms with the most sequences were nucleus (10,891; 11.3), plasma membrane (9,153; 9.5 %), mitochondrion (6,562; 6.8 %), and cytosol (5,545; 5.8 %). In the MF category the terms with the most sequences were oxidation-reduction process (4,445; 4.7 %), serine family amino acid metabolic process (3,309; 3.5 %), regulation of transcription, DNA-dependent (3,293; 3.5 %), and protein phosphorylation (2,336; 2.4 %). In total, KEGG maps for more than 160 metabolic pathways were generated. The full pathway list and the KEGG maps are available as Additional file 1: Table S1 and Additional file 2: Figure S1, respectively.

### L-ascorbic acid biosynthesis and recycling

**Fig. 5:**
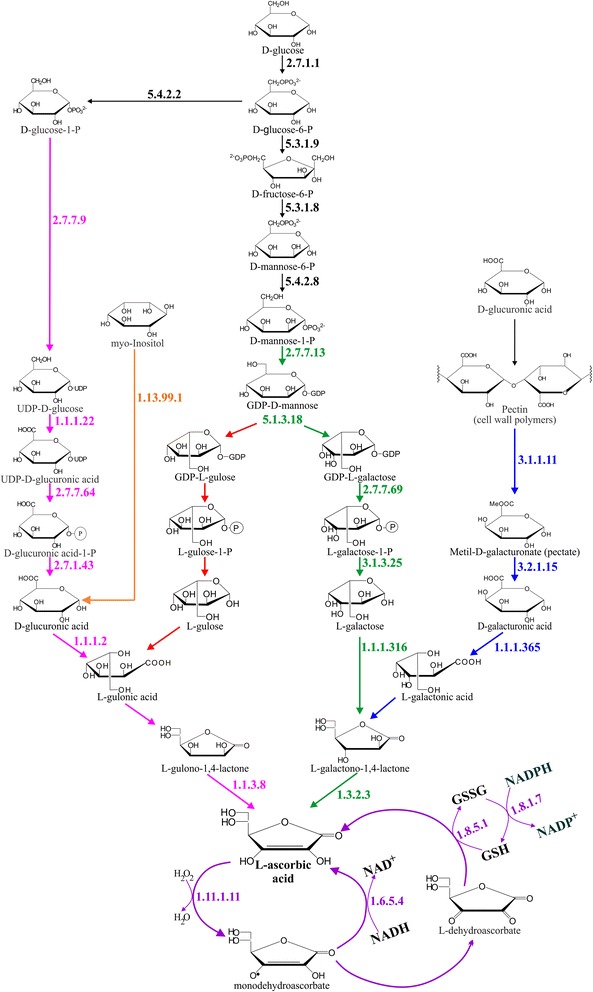
L-ascorbic acid biosynthesis and recycling pathways reconstructed based on the meta-assembly and annotation of the *M. dubia* fruit transcriptome

Although the metabolic pathways for AsA biosynthesis and recycling are known for several plant species, the existing knowledge of these pathways and enzymes involved in *M. dubia* are limited. Based on the KEGG pathway assignment (map00010, map00051, map00053, map00500, map00520, and map00562) of the functionally annotated sequences and local blast search of the *de novo* meta-assembly transcriptome, transcripts coding for the D-mannose/L-galactose pathway were found. This pathway involves the generation of AsA from D-mannose-1-phosphate (Fig. 5). GDP-D-mannose synthesis from D-mannose-1-phosphate and GTP is catalyzed by GDP-D-mannose pyrophosphorylase (E.C. 2.7.7.13), GDP-D-mannose is converted to GDP-L-galactose by a reversible double epimerization, catalyzed by GDP-mannose-3′,5′-epimerase (E.C. 5.1.3.18), then GDP-L-galactose is broken down by GDP-L-galactose:hexose-1-phosphate guanyltransferase (E.C. 2.7.7.69) to L-galactose-1-phosphate, which is subsequently hydrolyzed to L-galactose and inorganic phosphate by L-galactose-1-phosphate phosphatase (E.C. 3.1.3.25). L-galactose is then oxidized to L-galactono-1,4-lactone by the NAD-dependent L-galactose dehydrogenase (E.C. 1.1.1.316), finally L-galactono-1,4-lactone is oxidized to AsA by L-galactono-1,4-lactone dehydrogenase (E.C. 1.3.2.3).

Four additional AsA biosynthetic pathways were also identified. The first is the animal-like pathway. In this pathway D-glucuronic acid is generated from D-glucose via the intermediates: D-glucose-1-phosphate, UDP-D-glucose, UDP-D-glucuronic acid, and D-glucuronic acid-1-phosphate. D-glucuronic acid is then converted to L-gulonic acid by glucuronate reductase (E.C. 1.1.1.2), which is converted to L-gulono-1,4-lactone, from this compound AsA is generated by L-gulono-1,4-lactone oxidase/dehydrogenase (E.C. 1.1.3.8). The second alternative pathway uses *myo*-inositol as a precursor of AsA. In this pathway, D-glucuronic acid, an intermediate of the animal-like pathway, can be generated from myo-inositol by inositol oxygenase (E.C. 1.13.99.1). The third is the L-gulose pathway. In this putative pathway, the first metabolic intermediary (GDP-L-gulose) is generated from GDP-D-mannose by action of GDP-mannose-3′,5′-epimerase (E.C. 5.1.3.18), subsequently GDP-L-gulose is transformed to L-gulono-1,4-lactone throughput four sequential biochemical reactions. Finally, the fourth is the uronic acid pathway. In this pathway pectin-derived D-galacturonic acid is metabolized to AsA by an inversion pathway. The enzyme D-galacturonic acid reductase (E.C. 1.1.1.365) reduces the compound D-galacturonic acid to L-galactonic acid, which in turn is spontaneously converted to L-galactono-1,4-lactone. This compound is the substrate of the L-galactono-1,4-lactone dehydrogenase enzyme (E.C. 1.3.2.3).

We also identified all transcripts coding enzymes involved in the recycling pathway (i.e., ascorbate-gluthatione cycle). When AsA is oxidized to monodehydroascorbate by ascorbate peroxidase (E.C. 1.11.1.11), it can be reduced to AsA by monodehydroascorbate reductase (E.C. 1.6.5.4) or it can disproportionate non-enzymatically to AsA and dehydroascorbate (DHA). DHA can also be reduced to AsA by dehydroascorbate reductase (E.C. 1.8.5.1), using glutathione as the reductant (GSH) that is then converted to oxidized glutathione (GSSG). Finally, this compound is reduced by glutathione reductase (E.C. 1.8.1.7) using NADPH as the reductant.

### Discovery of molecular markers

**Fig. 6:**
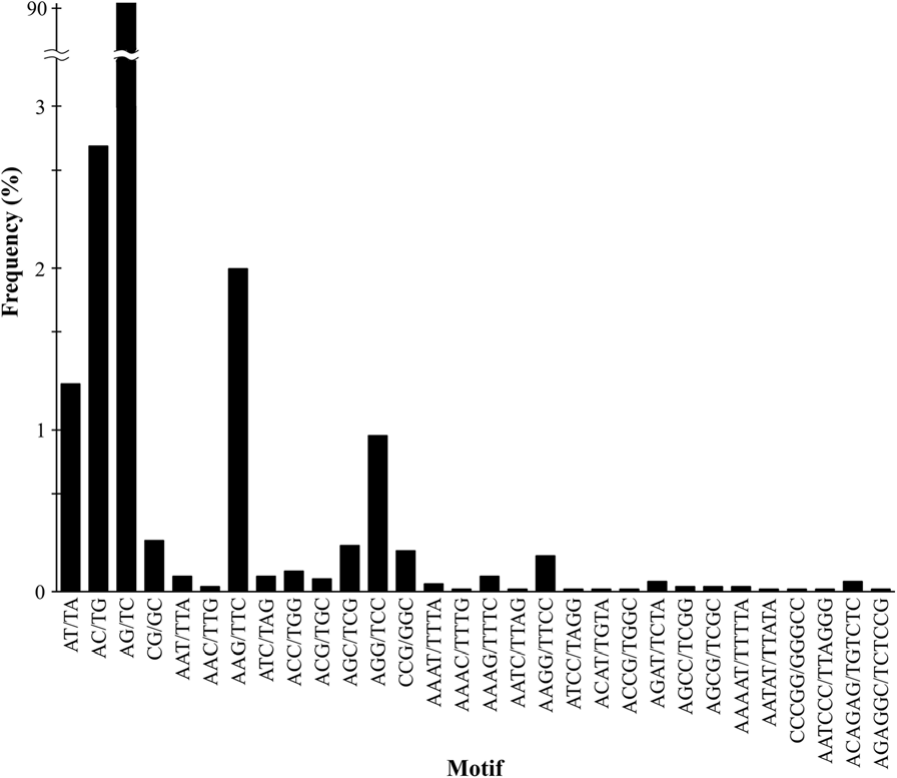
Frequency distribution of simple sequence repeats based on motif types identified in the *M. dubia* fruit transcriptome meta-assembly

All unigenes (70,048) of the meta-assembly were used to mine potential genic simple sequence repeats (genic-SSR) or microsatellites that were defined as di- to hexa-nucleotide motifs with a minimum of five repetitions. A total of 30 motifs with simple sequence repeats were identified in 6,314 (9.0 %) unigenes, but 287 genic-SSRs were included in unigenes without a match in the nr database (Additional file 3: Table S2). The di-nucleotide repeat AG/TC was the most abundant type (91.0 %), followed by other di- (4.4 %) and tri-nucleotide repeats (3.9 %; Fig. 6). The tetra-, penta-, and hexa-nucleotide repeats together exhibited the lowest frequency (0.7 %). Using Primer 3 Software we were able to design primers for 3,240 unigenes containing SSRs with product size ranging from 100 to 450 bp (Additional file 4: Table S3).

A total of 73,889 putative SNPs were also discovered, although only 23,481 met the selection criteria for robustness. The majority of these SNPs were bi-allelic (23,464) and only 17 were tri-allelic. These SNPs were found in 5,587 unigenes of which 5,226 (93.5 %) could be annotated with a GO term (Additional file 5: Table S4). The transition substitutions (33.5 % G↔A, and 33.6 % T↔C; totalling 67 %) were high compared to transversions (6.7 % C↔A, 9.9 % G↔C, 7.5 % T↔A, and 8.9 % T↔G; totalling 33 %) with an observed transition over transversion ratio of approximately 2.0.

Several of the genes involved in AsA metabolism proved to be polymorphic as evidenced by SNP discovery. For example, the D-mannose/L-galactose pathway mannose-1-phosphate guanylyltransferase (E.C. 2.7.7.13) contained >20 SNPs, GDP-mannose-3′,5′-epimerase (E.C. 5.1.3.18) had 13 SNPs, whereas L-galactono-1,4-lactone dehydrogenase (E.C. 1.3.2.3) only had 5 SNPs. The animal-like pathway UTP:glucose-1-phosphate uridylyltransferase (E.C. 2.7.7.9) contained 7 SNPs. In the uronic acid pathway pectin esterase (E.C. 3.1.1.11) and galacturan-1,4-alpha-galacturonidase (E.C. 3.2.1.15) showed more than 20 and 14 SNPs, respectively. Finally, in the ascorbate-glutathione pathway the unigenes monodehydroascorbate reductase (E.C. 1.6.5.4) and glutathione reductase (E.C. 1.8.1.7) contained 2 and 3 SNPs, respectively (Additional file 6: Table S5).

## Discussion

### Illumina paired end sequencing and *de novo* assembly

Fruit transcriptome sequencing of *M. dubia* with Illumina paired end sequencing technology and *de novo* assembly with the meta-assembly bioinformatics strategy were able to produce more than 24 million high-quality reads, and ~70,000 assembled unigenes with high N50 value (Fig. 1). Similar strategies have been widely utilized for successful *de novo* transcriptome sequencing and assembly of fruits in other plant species such as *Ananas comosus* [19], *Capsicum annuum* [20], *Litchi chinensis* [17], *Mangifera indica* [21], *Momordica cochinchinensis* [22], *Pyrus bretschneideri* [23], and *Vaccinium* spp. [24]. Additionally, analogous approaches were effectively used for sequencing and *de novo* assembly of transcriptome in various tissues of several non-model plant species without a reference genome [25–35]. The novel assembly methods [36–39] have made short read assembly to be a cost-effective and reliable tool for gene discovery and molecular markers development in non-model plant species.

### Functional annotation and metabolic pathway assignments

In the present study we annotated 75.2 % of the assembled transcriptome, leaving 17,341 unigenes unidentified. Similar results with a large number of unidentified sequences have been reported for other non-model organisms [17, 19–24, 26]. These unidentified sequences are likely to correspond to non-coding RNAs; short sequences lacking informative domains for conclusive annotation; or novel and/or specific genes of *M. dubia* that have not been previously characterized or coding orphan enzymes (i.e., unannotated gene sequences). The latter are all sequence-lacking enzymatic activities described in the literature and often catalogued in the EC database [40]. According to Sorokina et al. [41], 22.4 % of enzymatic activities from 5,096 ECs were orphans, and a large proportion of pathways (87 % in KEGG and 36 % in MetaCyc) contain at least one orphan activity. Of the pathways containing a mix of orphan and non-orphan activities in KEGG and MetaCyc, an average of 26.0 % and 39.5 % of the reactions per pathway corresponds to orphan enzymes, respectively. Consequently, most metabolic pathways are still not entirely resolved at the gene level, which restricts *in silico* reconstructions of metabolic pathways.

Two additional problems that create challenges for automated reconstructions of metabolic pathways are the number of misannotations in large public databases and the variation in metabolic pathways. First, Schnoes et al. [42] investigating the prevalence of annotation error in primary and secondary large public protein databases commonly used today, found that the manually curated database Swiss-Prot shows the lowest annotation error levels. The other two protein sequence databases (GenBank NR and TrEMBL) and the protein sequences in the KEGG pathways database exhibit similar and surprisingly high levels of misannotation that average 25 % in the enolase superfamily to over 60 % in the HAD superfamily. Second, the availability of sequenced genomes has revealed the diversity of biochemical solutions to similar chemical problems, because the pathway enzymes first discovered in model organisms are often not universally conserved [43]. For example, the tetrahydrofolate biosynthesis pathway and enzymes are not universal and alternate solutions are found for most steps, making this pathway and others like it a challenge for automatic annotation in many genomes [43].

Due to these limitations several metabolic pathways reconstructed from the annotated unigenes of *M. dubia* show gaps or missing genes. Consequently, comparative genomics, enzymatic, metabolomic, and structural analyses will be required to fill these pathway gaps [40, 44–46]. Despite such limitations, it was possible to completely reconstruct several KEGG pathways with the *M. dubia* transcriptome. The well represented pathways discovered in this study included L-ascorbic acid biosynthesis and recycling, phenylpropanoid biosynthesis, flavonoid biosynthesis, anthocyanin biosynthesis, pentose phosphate pathway, glutathione metabolism, plant pathogen interaction, biosynthesis of plant hormones, aminoacids biosynthesis and degradation, and circadian rhythm (Additional file 1: Table S1 and Additional file 2: Figure S1). In conclusion, while transcriptomic analysis is not a substitute for detailed gene and pathway studies, it does provide a broad overview of the important metabolic processes from which to efficiently build hypotheses that can guide future detailed studies on improving our understanding of L-ascorbic acid metabolism and the accumulation of other bioactive phytochemicals in this plant species.

### L-ascorbic acid biosynthesis and recycling

Plants can possess a total of five metabolic pathways for AsA biosynthesis. These metabolic pathways are the animal-like pathway [47], the myo-inositol pathway [48], the L-gulose pathway [49], the D-mannose/L-galactose pathway [11], and the uronic acid pathway [12]. All these pathways were identified in the fruit transcriptome of *M. dubia* (Fig. 5) and have also been documented in other plant species. For example, analysis of the *Citrus sinensis* fruit transcriptome indicated that genes from four of the five biosynthetic pathways (all but the animal-like pathway) are expressed [50], whereas expressed sequence tags from fruits and other tissues of four Actinidia species (*A. arguta*, *A. chinensis*, *A. deliciosa*, and *A. eriantha*) indicated that myo-inositol, D-mannose/L-galactose, and uronic acid are active [51]. In contrast, the analysis of fruit transcriptomes of *Ziziphus jujuba, Myrica rubra,* and *Ananas comosus* were only able to identify the D-mannose/L-galactose pathway as active [19, 52, 53]. From an evolutionary perspective, however, it is possible that many or even most of these metabolic pathways for AsA biosynthesis are conserved in plants, because AsA is one of the most abundant low molecular weight antioxidant of plants that plays an essential role in the detoxification of reactive oxygen species. In addition, AsA is important in plant development, hormone and light signaling, cell cycle, death, and cell expansion, pathogen responses, and as a cofactor for several key enzymes [13, 47, 54, 55]. Consequently, the differential activation of one or more, but not all five, metabolic pathways from the above examples via gene expression depends on several factors (e.g., organ/tissue type, development stage, physiological condition, environmental factors) and does not necessarily indicate their absence.

The L-gulose pathway is derived from the D-mannose/L-galactose pathway by action of the GDP-mannose-3′,5′-epimerase (E.C. 5.1.3.18) and evidence exists that it is active in plant cells, because both L-gulose and L-gulono-1,4-lactone serve as precursors of L-ascorbic acid biosynthesis [49], but have limited molecular and biochemical studies. To date, genic sequences coding enzymes catalysing four consecutive biochemical reactions are unknown: GDP-L-gulose → L-gulose-1-phosphate → L-gulose → L-gulonic acid → L-gulono-1,4-lactone (or L-gulose → L-gulono-1,4-lactone) → L-ascorbic acid. Except for L-gulono-1,4-lactone dehydrogenase, enzyme activities for these biochemical reactions also were not tested. Enzymatic activity of L-gulono-1,4-lactone dehydrogenase has been observed in cytosolic and mitochondrial fractions of the leaves and fruit pulp and peel of *M. dubia* [56] and tubers of *Solanum tuberosum* [49]. In addition, the genome of *Arabidopsis thaliana* contains genes (i.e., At1g 32300, At2g 46740, At2g 46750, At2g 46760, At5g11540, and At5g 56490) that are closely related with the rat L-gulono-1,4-lactone oxidase. Some of these genes could be coding enzymes responsible for the conversion of L-gulono-1,4-lactone to AsA [49]. Also, one putative unigene, probably coding for this enzyme, was identified in our assembled transcriptome (E.C. 1.1.3.8).

Some of the biochemical reactions of the L-gulose pathway, however, can likely be catalysed from promiscuous enzymes, which is an inherent property of many enzymes catalysing analogous biochemical reactions [57]. Indeed, some enzymes of the D-mannose/L-galactose pathway catalysing similar reactions of the L-gulose pathway have shown promiscuity. For example, the enzyme GDP-L-galactose:hexose 1-phosphate guanyltransferase (E.C. 2.7.7.69) has a low Km value (10 μM and 4.4 μM), a high turnover rate kcat (64 s^−1^ and 23 s^−1^), and a similar specificity constant kcat/Km (6.3x10^6^ s^−1^ M^−1^ and 5.7x10^6^ s^−1^ M^−1^) with the GDP-L-galactose and GDP-D-glucose substrates, respectively [58]. Furthermore, the enzyme L-galactose dehydrogenase catalyses the oxidative reduction of both L-galactose and L-gulose substrates in *Spinacia oleracea* [59]. Consequently, it is necessary to conduct additional research to further our understanding of these genes, enzymes and the contribution of these metabolic pathways to AsA biosynthesis and accumulation in fruits, other organs and tissues of *M. dubia*.

Based on the KEGG pathway assignments, we identified transcripts coding for all enzymes of the ascorbate-glutathione or “Foyer-Halliwell-Asada” pathway (Fig. 5). The enzymes of this metabolic pathway have been localized in several compartments of the plant cells, such as the cytosol, mitochondria, peroxisomes, and chloroplast [60, 61]. This distribution of the ascorbate-glutathione pathway components is attributable to its vital role, since this pathway is recognized to be a key player in H_2_O_2_ metabolism and AsA recycling [54]. AsA recycling requires a continuous supply of GSH and NADPH. The pathways supplying these reductant molecules lie outside of the AsA biosynthetic machinery. The key suppliers of GSH are by *de novo* biosynthesis in two ATP-dependent reactions catalyzed by γ-glutamylcysteine synthetase and glutathione synthetase [62]. The second biochemical process is catalyzed by glutathione reductase (E.C. 1.8.1.7), which uses NADPH to reduce GSSG to GSH [54]. Moreover, there are various sources of the essential reductant NADPH. The first and principal source is the oxidative pentose phosphate pathway [63]. The second significant source includes L-malate:NADP oxidoreductase (E.C. 1.1.1.40) that catalyzes the oxidative decarboxylation of L-malate to yield pyruvate, CO_2_ and NADPH in the presence of a bivalent cation [64]. Finally, NADPH is generated in photosynthetic cells (i.e. immature fruit peel of *M. dubia*) primarily from the light reactions of photosynthesis [65]. Therefore, genetic manipulations that increase the availability of GSH and NADPH for AsA recycling, through up-regulation/over-expression of related genes identified here, could be promising approaches to increase the yield of AsA in *M. dubia* and other plant species.

### Discovery of molecular markers

It is well-known that genic-SSR markers have numerous applications, such as functional genomics, association mapping, diversity analysis, genome mapping, transferability and comparative mapping, marker assisted selection breeding, and other applications [66]. Nevertheless, only eight genic-SSR markers have been developed for *M. dubia* until now [67, 68], limiting the applications previously mentioned. However, with this research mining the assembly fruit transcriptome of *M. dubia* it was possible to identify a large number of unigenes containing SSR (primers were designed for 3,240 unigenes) motifs that would be appropriates for developing a comprehensive set of genic-SSR markers that will need experimental validation. In conclusion, the genic-SSR markers identified in the assembly transcriptome database represent a significant addition to the limited set of markers available in *M. dubia* and it will be feasible to conduct marker assisted gene mapping for important agronomical traits (e.g., L-ascorbic acid and anthocyanin accumulation, fruit size) or biological processes (e.g., seed germination, growth and development, disease resistance, etc.).

Our results regarding genic-SSR markers are largely similar to other plant studies, but differences do exist. First, the percentage of unigenes containing SSRs (9.0 % in *M. dubia*) is comparable to reports for this species from Brazil with 10.9 % [67] as well as for *Ipomoea batatas* with 7.3 % [32], *Cajanus cajan* with 7.7 % [69], *Capsicum anuum* with 7.7 % [15], and *Sesamum indicum* with 8.9 % [28]. Differences, however, existed when compared to *Apium graveolens* with 6.2 % [70] and the monocot *Phoenix dactylifera* with 16.0 % [71]. Second, regarding the distribution of the perfect repeat motif types, tri-nucleotide repeats have generally been observed to have the highest frequency in cereals and other plant species [71–73]. However, here, as in a previous study on *M. dubia* [67] and other plant species [14, 28, 69, 70, 74], the most abundant repeat motif type was di-nucleotide repeats, followed by tri-nucleotide repeats. Of the thirty motifs, (AG/TC)n showed the highest frequency (91.0 %), which is in agreement with other plant species [14, 28, 32, 71, 74]. As in monocot [74] and other dicot plants [28, 32, 75] the (AAG/TTC)n motif in *M.* dubia was the most abundant of the tri-nucleotide repeat motifs. This triplet codes for lysine, which is commonly found in the exons of plants [75]. This finding is consistent with Katti et al. [76] who showed that expansions of codon repeats corresponding to small hydrophilic amino acids are tolerated more, while strong selection pressures probably eliminate codon repeats encoding for hydrophobic and basic amino acids.

In addition, in our dataset some unigenes containing genic-SSR were lacking functional annotations. These unidentified unigenes probably correspond to untranslated (UTR) regions. Several researches showed that SSR frequency is high in the 5′ UTR regions of plant transcripts [77–80], suggesting that SSRs located in this genic region can potentially act as factors in regulating gene expression in the transcriptional or translational levels [78, 81]. Consequently, these insights are likely to play a significant role in selecting SSRs loci to be used in molecular breeding programs of *M. dubia*.

Sequencing a pool of cDNA using next-generation sequencing technologies and appropriate mining software allows for the rapid and inexpensive SNP discovery within genes in non-model plants without a reference genome. Our transcriptome dataset contained a large number of high quality SNPs (>23,000) and marks the highest number of SNP markers discovered to date from *M. dubia* using transcriptome sequencing. While the majority of SNPs were bi-allelic (>99.9 %), an insignificant fraction showed tri-allelic (0.072 %) polymorphisms. These results are in agreement with the diploid nature of the *M. dubia* genome [1]. Similar low levels of tri-allelic SNPs also were reported in other plant species such as *Brassica napus* with 0.029–0.06 % [82, 83] and *Manihot esculenta* with 0.52 % [84], whereas tri-allelic SNPs were not detected in *Sesamum indicum* [85] and *Hevea brasiliensis* [86]. Nevertheless, the switchgrass *Panicum virgatum* possesses a substancial number of tri-allelic SNPs (15 %), which is consistent with the polyploid condition of the genome of this species [87]. Although in principle, at each position of a sequence any of the four nucleotide bases can be present, however, SNPs are frequently biallelic. One possible explanation is the low frequency of single nucleotide substitutions (5.0-30.0 x 10^−9^) at the nuclear genes of plants [88]. Consequently, the probability of two or three independent mutations occurring at a single position is very low. Another important cause for the prevalence of bi-allelic SNP is attributable to a clear bias in the mutation mechanism that results in a prevalence of transitions over transversions exchanges (67 % vs 33 % in our data set). One probable explanation for this is the high frequency of spontaneous deamination of 5-methyl cytosine to thymidine in the CpG dinucleotides [89].

Although SNPs are less polymorphic than SSR markers, they easily compensate for this drawback by being abundant and amenable to high- and ultra-high-throughput automation [90]. Consequently, this large collection of SNP markers could facilitate genetic applications in *M. dubia* such as genetic diversity and characterization, linkage mapping, high-density quantitative trait locus analysis, association studies, map-based cloning, marker-assisted plant breeding, and functional genomics.

## Conclusions

This study describes the first next-generation sequencing effort and transcriptome annotation of a non-model Amazonian plant that is relevant for AsA production and other bioactive phytochemicals. Genes encoding key enzymes were successfully identified and metabolic pathways involved in biosynthesis of AsA, anthocyanins, and other metabolic pathways have been reconstructed. The identification of these genes and pathways is in agreement with the empirically observed capability of *M. dubia* to synthesize and accumulate AsA and other important molecules, and adds to our current knowledge of the molecular biology and biochemistry of their production in plants. By providing insights into the mechanisms underpinning these metabolic processes, these results can be used to direct efforts to genetically manipulate this organism in order to enhance the production of these bioactive phytochemicals.

The accumulation of AsA precursor and discovery of genes associated with their biosynthesis and metabolism in *M. dubia* is intriguing and worthy of further investigation. The sequences and pathways produced here present the genetic framework required for further studies. Quantitative transcriptomics in concert with studies of the genome, proteome, and metabolome under conditions that stimulate production and accumulation of AsA and their precursors are needed to provide a more comprehensive view of how these pathways for AsA metabolism are regulated and linked in this species.

## Methods

### Plant material

Unripe (60 days after anthesis) and ripe fruits (70 days after anthesis) were randomly collected from 10 different accessions (one plant by accession) from the *M. dubia* germplasm bank (03°57′17″S, 73°24′55″W) at the Instituto Nacional de Innovación Agraria of Peru, region Loreto. Established approximately 20 years ago, this germplasm bank consists of 43 representative accessions of genetic variability of *M. dub*ia from the eight major river basins of the Loreto Region (Nanay, Itaya, Napo, Ucayali, Putumayo, Curaray, Tigre and Amazonas). Immediately after harvesting, samples were stored at −80°C until further use. A graphical representation of our work flow is provided in Additional file 7: Figure S2.

### Total RNA isolation and cDNA synthesis

Total RNA was isolated from seeds and fruit pulp and peel from each of the 10 plants using the CTAB method, solvent extractions, and DNase treatment as described by Castro et al. [91]. RNA samples were chosen and pooled equally for cDNA library construction and sequencing if the OD ratio A_260_/A_280_ > 1.9, A_260_/A_230_ > 2.0, and the samples were not degraded as assessed by formaldehyde denaturing gel electrophoresis [92].

### cDNA library construction and sequencing

Illumina sequencing was performed at Macrogen’s sequencing service according to the manufacturer’s instructions (Illumina Inc., San Diego, CA, USA). First, mRNA with a poly(A) tail was isolated from 20 μg of pooled total RNA using Sera-mag magnetic oligo (dT) beads (Illumina). To avoid priming bias, the purified mRNA was first fragmented into small pieces (100–400 bp) using divalent cations at 94°C for 5 minutes. With random hexamer primers (Illumina), the double-stranded cDNA was synthesized using the SuperScript double-stranded cDNA synthesis kit (Invitrogen, CA). The synthesized cDNA was subjected to end-repair and phosphorylation, and then the repaired cDNA fragments were 3′ adenylated with Klenow Exo- (3′ to 5′ exo minus, Illumina). Illumina paired-end adapters were ligated to the ends of these 3′-adenylated cDNA fragments. To select the proper templates for downstream enrichment, products from the ligation reaction were gel (2 % agarose) purified and excised. Fifteen cycles of PCR amplification were carried out to enrich the purified cDNA template using PCR primers PE 1.0 and 2.0 (Illumina) with phusion DNA polymerase. Finally, the cDNA library was constructed with a 200 bp insertion fragment. After validation on an Agilent Technologies 2100 Bioanalyzer, the library was sequenced using an Illumina HiSeq™ 2000 (Illumina Inc., San Diego, CA, USA).

### Data filtering and *de novo* assembly

Prior to assembly raw sequencing reads were filtered and trimmed with Trimmomatic v0.32 [93] using the following steps: (1) leading and trailing bases of low quality or N bases were removed, (2) the 3′ end was cut and removed if the quality score of a 4 bp wide sliding window dropped below 15, and (3) remaining reads less than 36 bp and singletons were removed.

Cleaned reads were *de novo* assembled following the multiple k-mer approach of Melicher et al. [94]. Briefly, we used Velvet v1.2.10 [36] and Oases v0.2.08 [37] to produce assembles of k-mer lengths 21, 25, 29, 33, and 37. An additional assembly was produced with Trinity v20140717 [38] using the default settings at a k-length of 25. The transcripts from the 6 assemblies were then pooled and re-assembled using CAP3 [39] to produce a meta-assembly.

### Functional annotation, and metabolic pathway assignments

To elucidate the potential functions of gene transcripts we utilized the web tool FastAnnotator [95], which integrates the well-established annotation tools Blast2GO [96], PRIAM [97], and RPS BLAST [98] to assign Gene Ontology (GO) terms, Enzyme Commission numbers (EC numbers), and functional domains to query sequences (cut-off Evalue ≤ 10^−6^). To determine metabolic pathways, sequences assigned ECs from FastAnnotator were mapped to the Kyoto Encyclopedia of Genes and Genomes (KEGG) metabolic pathway database [99]. To further enrich the pathway annotation and to identify the BRITE functional hierarchies, sequences were also submitted to the KEGG Automatic Annotation Server (KAAS) [100] with the single-directional best hit information method selected.

### Discovery of molecular markers

Unigenes were mined for genic simple sequence repeats (genic-SSR) with MSATCOMMANDER v1.0.8 [101] and primers designed with the integrated Primer 3 Software [102] using the default setting for both programs, except that only perfect repeats (i.e., di-, tri-, tetra-, penta-, hexanucleotides) were selected and mononucleotide repeats and complex SSR types were excluded. Only those genic-SSRs with ≥ 5 repeats were retained.

SNPs were identified by first mapping the filtered reads to the final meta-assembly using BWA v0.7.10 [103] and then mpileup of SAMtools v1.1 [104] was used to detect SNP sites. Only SNPs with quality scores >20 and coverage depth >20 were labeled as high quality. The locations of the SNPs in unigenes were predicted with TransDecoder (http://transdecoder.github.io/) and snpEff v3.1 [105] was used to predict the effects of SNPs on genes.

## Additional files

Additional file 1: Table S1. KEEG Pathway list in transcriptome of *M. dubia. (CSV 6 kb)*
Additional file 2: Figure S1. KEEG Pathway Maps in transcriptome of *M. dubia. (PDF 7038 kb)*
Additional file 3: Table S2. Genic-SSR characteristics of *M. dubia. (CSV 330 kb)*
Additional file 4: Table S3. Primers characteristics for genic-SSR of *M. dubia. (CSV 1694 kb)*
Additional file 5: Table S4. SNPs in unigenes of *M. dubia. (XLSX 2653 kb)*
Additional file 6: Table S5. Summary of SNPs effect in *M. dubia. (CSV 576 bytes)*
Additional file 7: Figure S2. Flow chart of methods used. (PNG 495 kb)
