# Supplementary material for: De novo assembly and functional annotation of Myrciaria dubia fruit transcriptome reveals multiple metabolic pathways for L-ascorbic acid biosynthesis
Source: BMC Genomics. 2015 Nov 24;16:997. doi: 10.1186/s12864-015-2225-6 (PMC4658800; doi:10.1186/s12864-015-2225-6)
Supplement: Additional file 2: Figure S1. — KEEG Pathway Maps in transcriptome of M. dubia. (PDF 7038 kb) [file 12864_2015_2225_MOESM2_ESM.pdf]

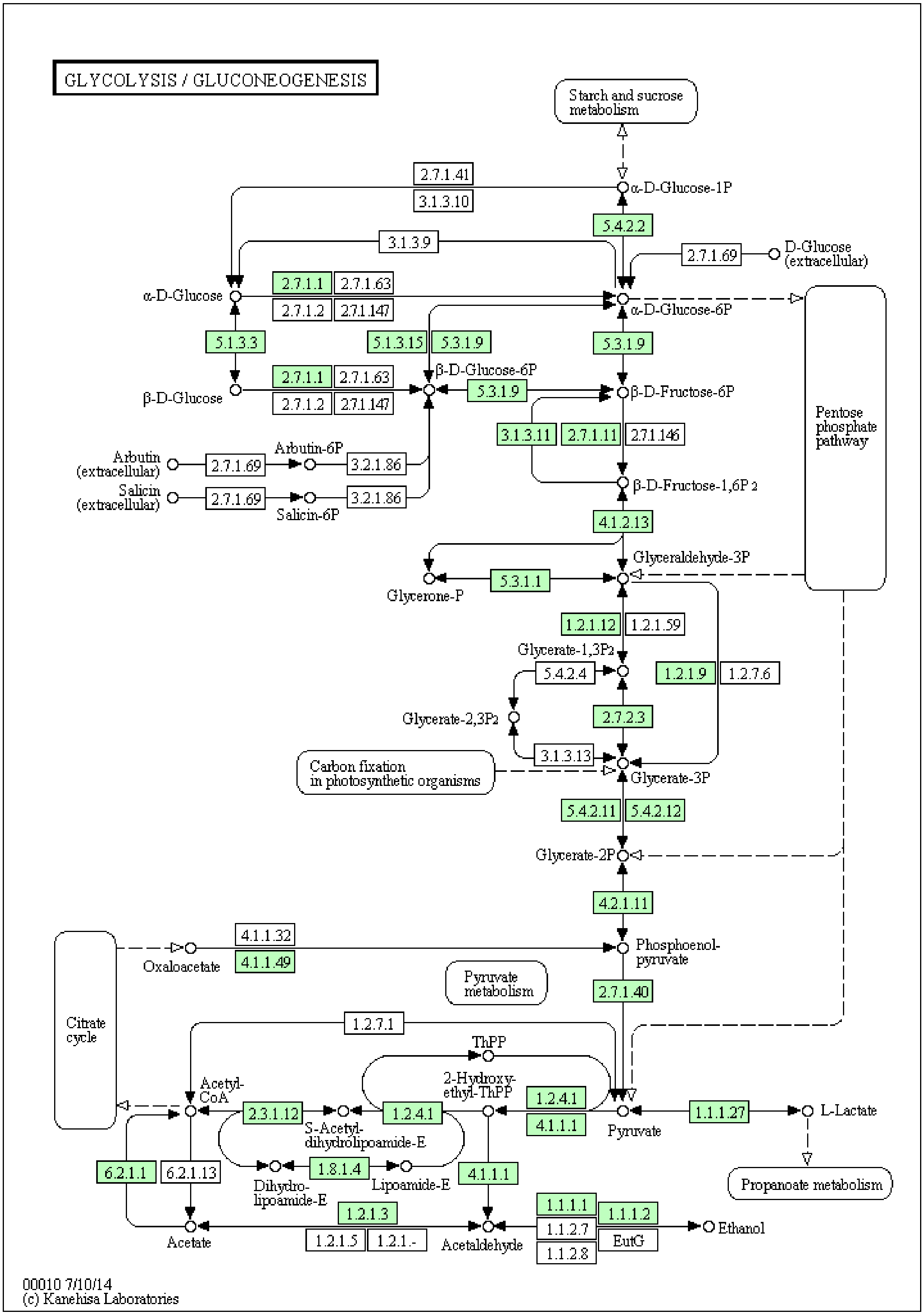
GLYCOLYSIS / GLUCONEOGENESIS
Starch and sucrose metabolism
2.7.1.41
3.1.3.10
α-D-Glucose-1P
5.4.2.2
3.1.3.9
2.7.1.69
D-Glucose (extracellular)
α-D-Glucose
2.7.1.1
2.7.1.63
2.7.1.2
2.7.1.147
α-D-Glucose-6P
5.1.3.3
5.1.3.15
5.3.1.9
5.3.1.9
β-D-Glucose-6P
2.7.1.1
2.7.1.63
β-D-Glucose
2.7.1.2
2.7.1.147
5.3.1.9
β-D-Fructose-6P
Pentose phosphate pathway
3.1.3.11
2.7.1.11
2.7.1.146
Arbutin (extracellular)
2.7.1.69
Arbutin-6P
3.2.1.86
Salicin (extracellular)
2.7.1.69
3.2.1.86
Salicin-6P
β-D-Fructose-1,6P2
4.1.2.13
Glyceraldehyde-3P
5.3.1.1
Glycerone-P
1.2.1.12
1.2.1.59
Glycerate-1,3P2
5.4.2.4
1.2.1.9
1.2.7.6
Glycerate-2,3P2
2.7.2.3
3.1.3.13
Carbon fixation in photosynthetic organisms
Glycerate-3P
5.4.2.11
5.4.2.12
Glycerate-2P
4.2.1.11
4.1.1.32
Citrate cycle
Oxaloacetate
4.1.1.49
Phosphoenol-pyruvate
Pyruvate metabolism
2.7.1.40
1.2.7.1
ThPP
2-Hydroxy-ethyl-ThPP
Acetyl-CoA
2.3.1.12
S-Acetyl-dihydrolipoamide-E
1.2.4.1
1.2.4.1
4.1.1.1
Pyruvate
1.1.1.27
L-Lactate
6.2.1.1
6.2.1.13
Dihydro-lipoamide-E
1.8.1.4
Lipoamide-E
4.1.1.1
Propanoate metabolism
1.2.1.3
1.1.1.1
1.1.1.2
Acetate
1.2.1.5
1.2.1.-
Acetaldehyde
1.1.2.7
EutG
Ethanol
1.1.2.8
00010 7/10/14
(c) Kanehisa Laboratories

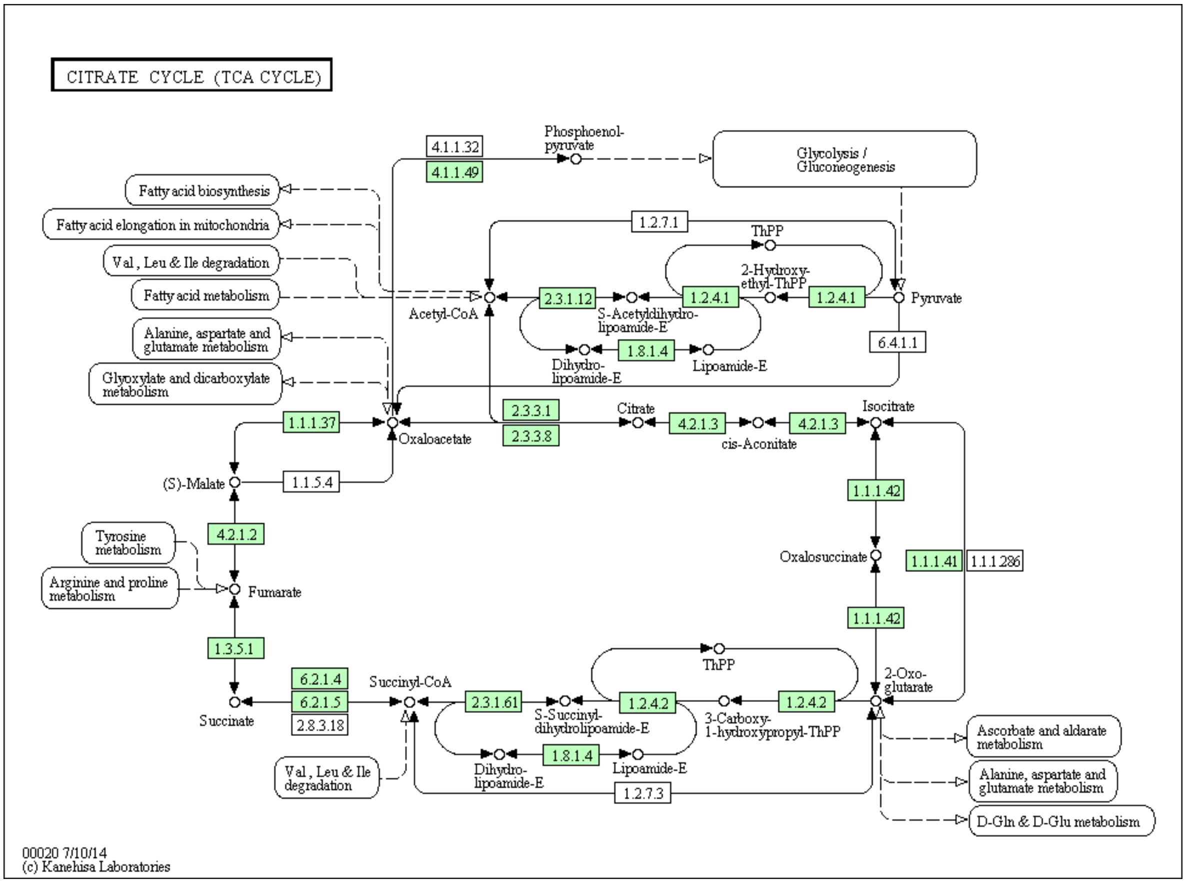
CITRATE CYCLE (TCA CYCLE)
Phosphoenol-pyruvate
4.1.1.32
4.1.1.49
Glycolysis / Gluconeogenesis
Fatty acid biosynthesis
Fatty acid elongation in mitochondria
Val, Leu & Ile degradation
Fatty acid metabolism
Alanine, aspartate and glutamate metabolism
Glyoxylate and dicarboxylate metabolism
1.2.7.1
ThPP
2-Hydroxy-ethyl-ThPP
Acetyl-CoA
2.3.1.12
S-Acetyldihydro-lipoamide-E
1.2.4.1
1.2.4.1
Pyruvate
1.8.1.4
Dihydro-lipoamide-E
Lipoamide-E
6.4.1.1
2.3.3.1
2.3.3.8
Citrate
4.2.1.3
cis-Aconitate
4.2.1.3
Isocitrate
1.1.1.37
Oxaloacetate
(S)-Malate
1.1.5.4
4.2.1.2
1.1.1.42
Oxalosuccinate
1.1.1.41
1.1.1.286
Tyrosine metabolism
Arginine and proline metabolism
Fumarate
1.1.1.42
1.3.5.1
ThPP
6.2.1.4
6.2.1.5
2.8.3.18
Succinyl-CoA
2.3.1.61
S-Succinyl-dihydrolipoamide-E
1.2.4.2
3-Carboxy-1-hydroxypropyl-ThPP
1.2.4.2
2-Oxo-glutarate
Succinate
Val, Leu & Ile degradation
1.8.1.4
Dihydro-lipoamide-E
Lipoamide-E
1.2.7.3
Ascorbate and aldarate metabolism
Alanine, aspartate and glutamate metabolism
D-Gln & D-Glu metabolism
00020 7/10/14
(c) Kanehisa Laboratories

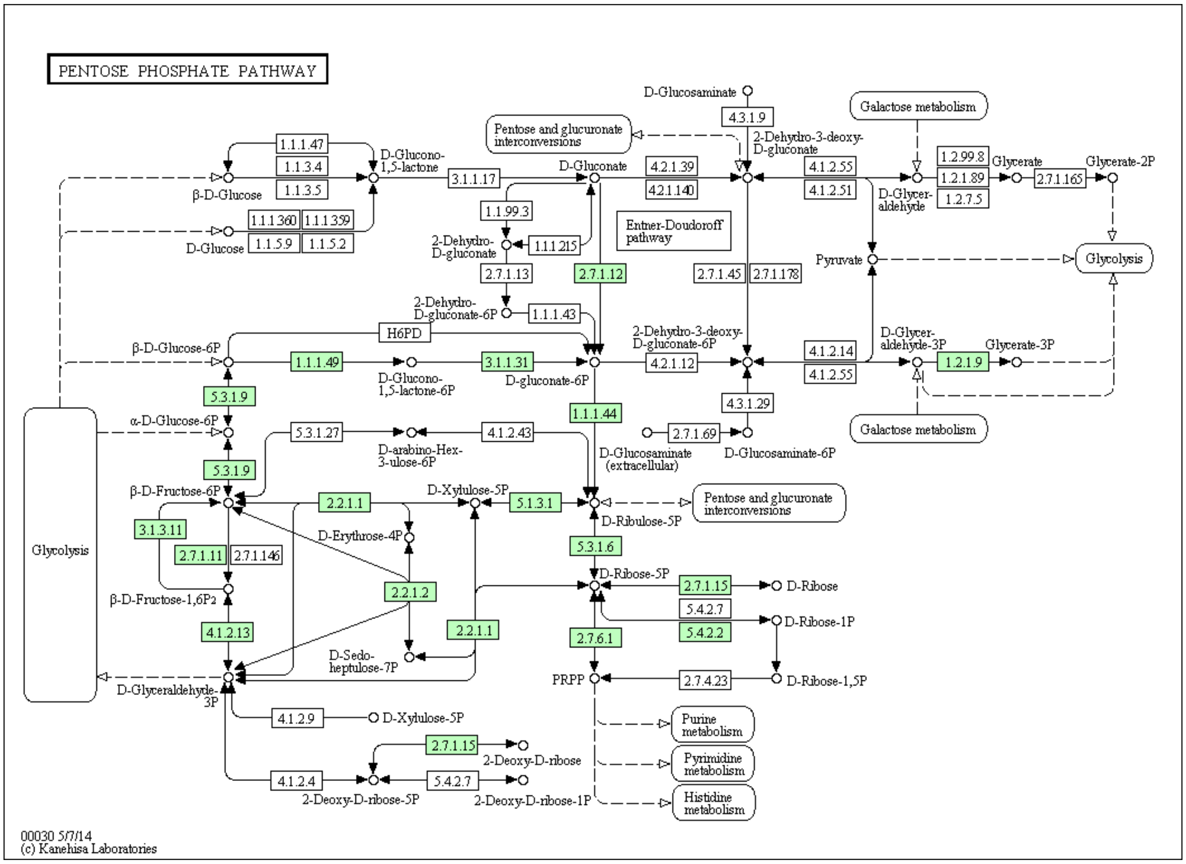
PENTOSE PHOSPHATE PATHWAY
D-Glucosaminate
4.3.1.9
Galactose metabolism
Pentose and glucuronate interconversions
2-Dehydro-3-deoxy-D-gluconate
1.1.1.47
1.1.3.4
1.1.3.5
D-Glucono-1,5-lactone
3.1.1.17
D-Gluconate
4.2.1.39
4.2.1.140
4.1.2.55
4.1.2.51
1.2.99.8
1.2.1.89
1.2.7.5
Glycerate
2.7.1.165
Glycerate-2P
β-D-Glucose
1.1.1.360
1.1.1.359
1.1.5.9
1.1.5.2
D-Glucose
1.1.99.3
2-Dehydro-D-gluconate
1.1.1.215
Entner-Doudoroff pathway
D-Glycer-aldehyde
Pyruvate
Glycolysis
2.7.1.13
2.7.1.12
2.7.1.45
2.7.1.178
2-Dehydro-D-gluconate-6P
1.1.1.43
H6PD
2-Dehydro-3-deoxy-D-gluconate-6P
4.1.2.14
4.1.2.55
D-Glycer-aldehyde-3P
1.2.1.9
Glycerate-3P
β-D-Glucose-6P
1.1.1.49
D-Glucono-1,5-lactone-6P
3.1.1.31
D-gluconate-6P
4.2.1.12
5.3.1.9
1.1.1.44
4.3.1.29
Galactose metabolism
α-D-Glucose-6P
5.3.1.27
D-arabino-Hex-3-ulose-6P
4.1.2.43
2.7.1.69
D-Glucosaminate (extracellular)
D-Glucosamimate-6P
5.3.1.9
β-D-Fructose-6P
2.2.1.1
D-Xylulose-5P
5.1.3.1
D-Ribulose-5P
Pentose and glucuronate interconversions
Glycolysis
3.1.3.11
D-Erythrose-4P
5.3.1.6
2.7.1.11
2.7.1.146
D-Ribose-5P
2.7.1.15
D-Ribose
β-D-Fructose-1,6P2
2.2.1.2
5.4.2.7
D-Ribose-1P
5.4.2.2
4.1.2.13
2.2.1.1
2.7.6.1
D-Sedo-heptulose-7P
PRPP
2.7.4.23
D-Ribose-1,5P
D-Glyceraldehyde-3P
4.1.2.9
D-Xylulose-5P
Purine metabolism
2.7.1.15
2-Deoxy-D-ribose
Pyrimidine metabolism
4.1.2.4
5.4.2.7
2-Deoxy-D-ribose-5P
2-Deoxy-D-ribose-1P
Histidine metabolism
00030 5/7/14
(c) Kanehisa Laboratories

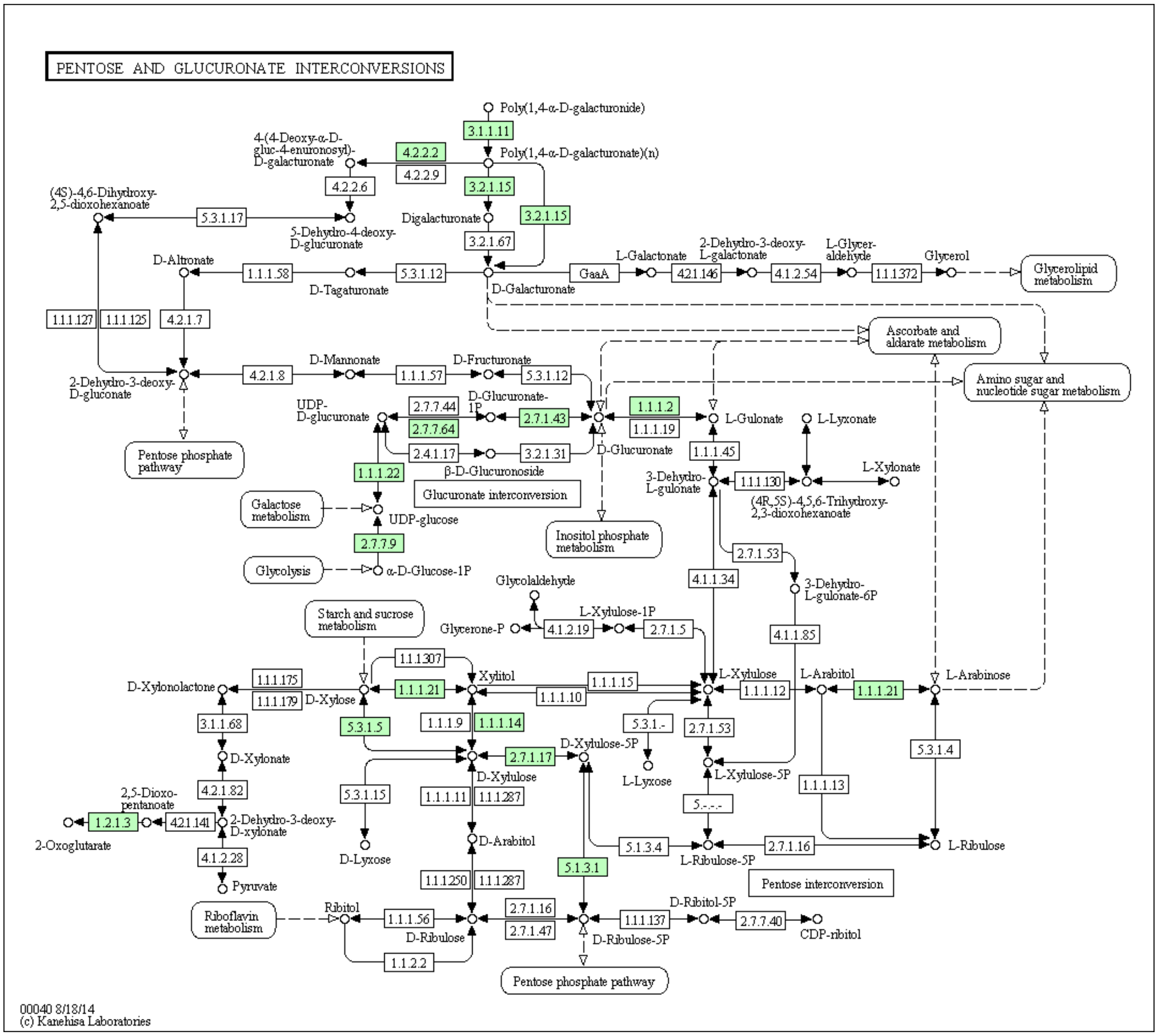

PENTOSE AND GLUCURONATE INTERCONVERSIONS
Poly(1,4-α-D-galacturonide)
3.1.1.11
Poly(1,4-α-D-galacturonate)(n)
4-(4-Deoxy-α-D-gluc-4-enuronosyl)-D-galacturonate
4.2.2.2
4.2.2.9
4.2.2.6
3.2.1.15
Digalacturonate
3.2.1.67
(4S)-4,6-Dihydroxy-2,5-dioxohexanoate
5.3.1.17
5-Dehydro-4-deoxy-D-glucuronate
D-Altronate
1.1.1.58
D-Tagaturonate
5.3.1.12
D-Galacturonate
GaaA
L-Galactonate
4.2.1.146
2-Dehydro-3-deoxy-L-galactonate
4.1.2.54
L-Glycer-aldehyde
1.1.1.372
Glycerol
Glycerolipid metabolism
Ascorbate and aldarate metabolism
Amino sugar and nucleotide sugar metabolism
1.1.1.127
1.1.1.125
4.2.1.7
2-Dehydro-3-deoxy-D-gluconate
4.2.1.8
D-Mannonate
1.1.1.57
D-Fructuronate
5.3.1.12
UDP-D-glucuronate
2.7.7.44
2.7.7.64
D-Glucuronate-1P
2.7.1.43
1.1.1.2
1.1.1.19
L-Gulonate
L-Lyxonate
2.4.1.17
3.2.1.31
β-D-Glucuronoside
D-Glucuronate
1.1.1.45
3-Dehydro-L-gulonate
1.1.1.130
L-Xylonate
(4R,5S)-4,5,6-Trihydroxy-2,3-dioxohexanoate
Pentose phosphate pathway
1.1.1.22
Glucuronate interconversion
Galactose metabolism
UDP-glucose
Inositol phosphate metabolism
2.7.7.9
Glycolysis
α-D-Glucose-1P
2.7.1.53
4.1.1.34
3-Dehydro-L-gulonate-6P
Glycolaldehyde
L-Xylulose-1P
Starch and sucrose metabolism
Glycerone-P
4.1.2.19
2.7.1.5
4.1.1.85
1.1.1.307
Xylitol
L-Xylulose
L-Arabitol
L-Arabinose
D-Xylonolactone
1.1.1.175
1.1.1.179
D-Xylose
1.1.1.21
1.1.1.15
1.1.1.10
1.1.1.12
1.1.1.21
3.1.1.68
5.3.1.5
1.1.1.9
1.1.1.14
5.3.1.-
2.7.1.53
5.3.1.4
D-Xylonate
2.7.1.17
D-Xylulose-5P
L-Lyxose
L-Xylulose-5P
D-Xylulose
1.1.1.13
2,5-Dioxo-pentanoate
4.2.1.82
5.3.1.15
1.1.1.11
1.1.1.287
5.-.-.-
1.2.1.3
4.2.1.141
2-Dehydro-3-deoxy-D-xylonate
2-Oxoglutarate
D-Lyxose
D-Arabitol
5.1.3.4
L-Ribulose-5P
2.7.1.16
L-Ribulose
4.1.2.28
5.1.3.1
Pyruvate
1.1.1.250
1.1.1.287
Pentose interconversion
Riboflavin metabolism
Ribitol
1.1.1.56
D-Ribulose
2.7.1.16
2.7.1.47
D-Ribulose-5P
1.1.1.137
D-Ribitol-5P
2.7.7.40
CDP-ribitol
1.1.2.2
Pentose phosphate pathway
00040 8/18/14
(c) Kanehisa Laboratories

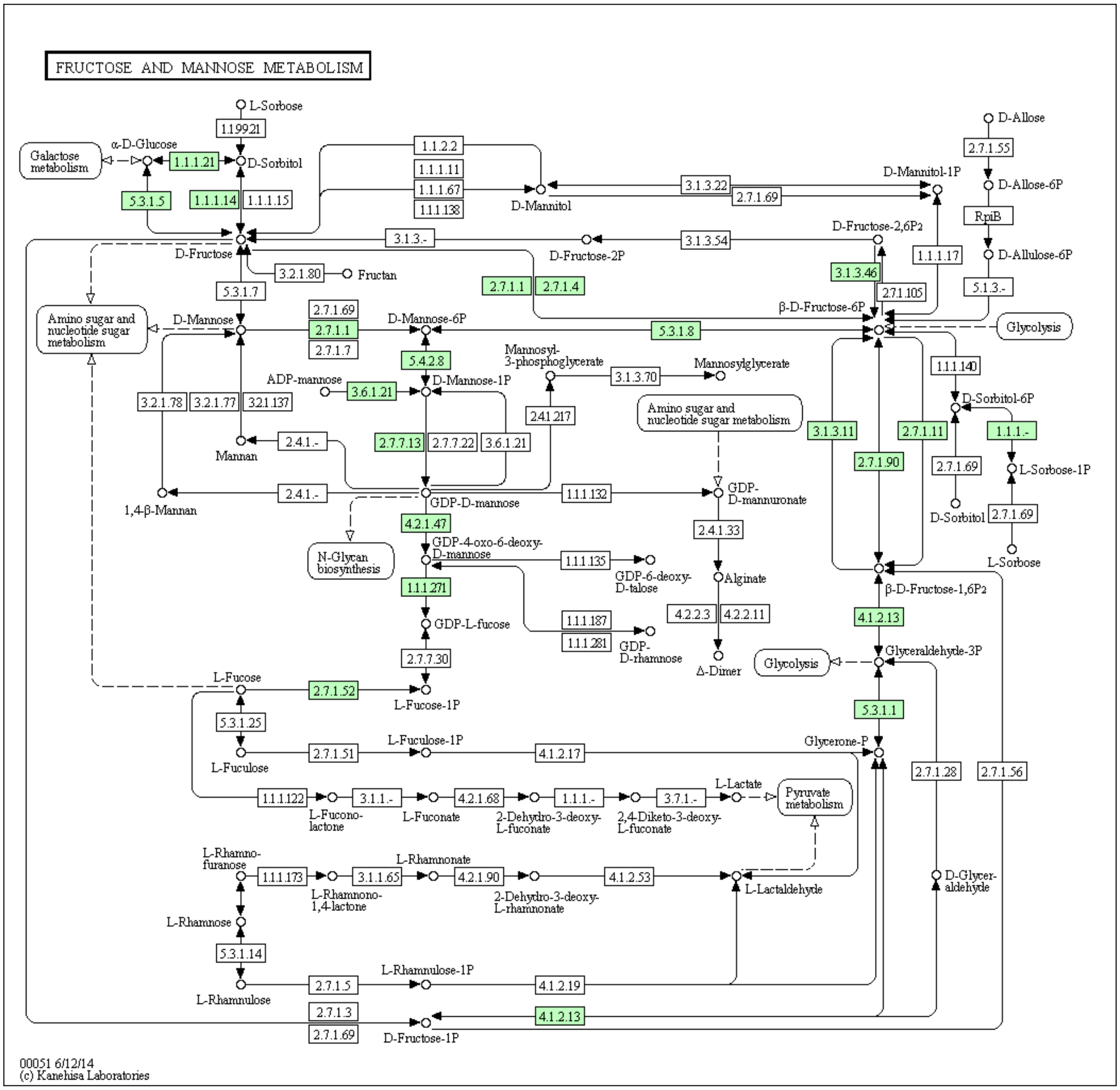
FRUCTOSE AND MANNOSE METABOLISM
L-Sorbose
1.1.99.21
α-D-Glucose
D-Sorbitol
Galactose metabolism
1.1.1.21
5.3.1.5
1.1.1.14
1.1.1.15
1.1.2.2
1.1.1.11
1.1.1.67
1.1.1.138
D-Mannitol
3.1.3.22
2.7.1.69
D-Mannitol-1P
D-Allose
2.7.1.55
D-Allose-6P
RpiB
D-Allulose-6P
5.1.3.-
D-Fructose
3.1.3.-
D-Fructose-2P
3.1.3.54
D-Fructose-2,6P2
1.1.1.17
3.1.3.46
2.7.1.105
3.2.1.80
Fructan
2.7.1.1
2.7.1.4
5.3.1.7
β-D-Fructose-6P
Glycolysis
Amino sugar and nucleotide sugar metabolism
D-Mannose
2.7.1.69
2.7.1.1
2.7.1.7
D-Mannose-6P
5.3.1.8
5.4.2.8
ADP-mannose
3.6.1.21
D-Mannose-1P
Mannosyl-3-phosphoglycerate
3.1.3.70
Mannosylglycerate
3.2.1.78
3.2.1.77
3.2.1.137
2.4.1.217
1.1.1.140
D-Sorbitol-6P
1.1.1.-
Amino sugar and nucleotide sugar metabolism
3.1.3.11
2.7.1.11
2.7.1.90
2.7.1.69
L-Sorbose-1P
D-Sorbitol
2.7.1.69
L-Sorbose
Mannan
2.4.1.-
2.7.7.13
2.7.7.22
3.6.1.21
1,4-β-Mannan
2.4.1.-
GDP-D-mannose
1.1.1.132
GDP-D-mannuronate
2.4.1.33
Alginate
4.2.2.3
4.2.2.11
Δ-Dimer
4.2.1.47
GDP-4-oxo-6-deoxy-D-mannose
N-Glycan biosynthesis
1.1.1.135
GDP-6-deoxy-D-talose
1.1.1.271
GDP-L-fucose
1.1.1.187
1.1.1.281
GDP-D-rhamnose
β-D-Fructose-1,6P2
4.1.2.13
Glyceraldehyde-3P
Glycolysis
2.7.7.30
L-Fucose
2.7.1.52
L-Fucose-1P
5.3.1.25
5.3.1.1
L-Fuculose
2.7.1.51
L-Fuculose-1P
4.1.2.17
Glycerone-P
2.7.1.28
2.7.1.56
1.1.1.122
L-Fucono-lactone
3.1.1.-
L-Fuconate
4.2.1.68
2-Dehydro-3-deoxy-L-fuconate
1.1.1.-
2,4-Diketo-3-deoxy-L-fuconate
3.7.1.-
L-Lactate
Pyruvate metabolism
L-Rhamno-furanose
1.1.1.173
L-Rhamnono-1,4-lactone
3.1.1.65
L-Rhamnonate
4.2.1.90
2-Dehydro-3-deoxy-L-rhamnonate
4.1.2.53
L-Lactaldehyde
D-Glyceraldehyde
L-Rhamnose
5.3.1.14
L-Rhamnulose
2.7.1.5
L-Rhamnulose-1P
4.1.2.19
2.7.1.3
2.7.1.69
4.1.2.13
D-Fructose-1P
00051 6/12/14
(c) Kanehisa Laboratories

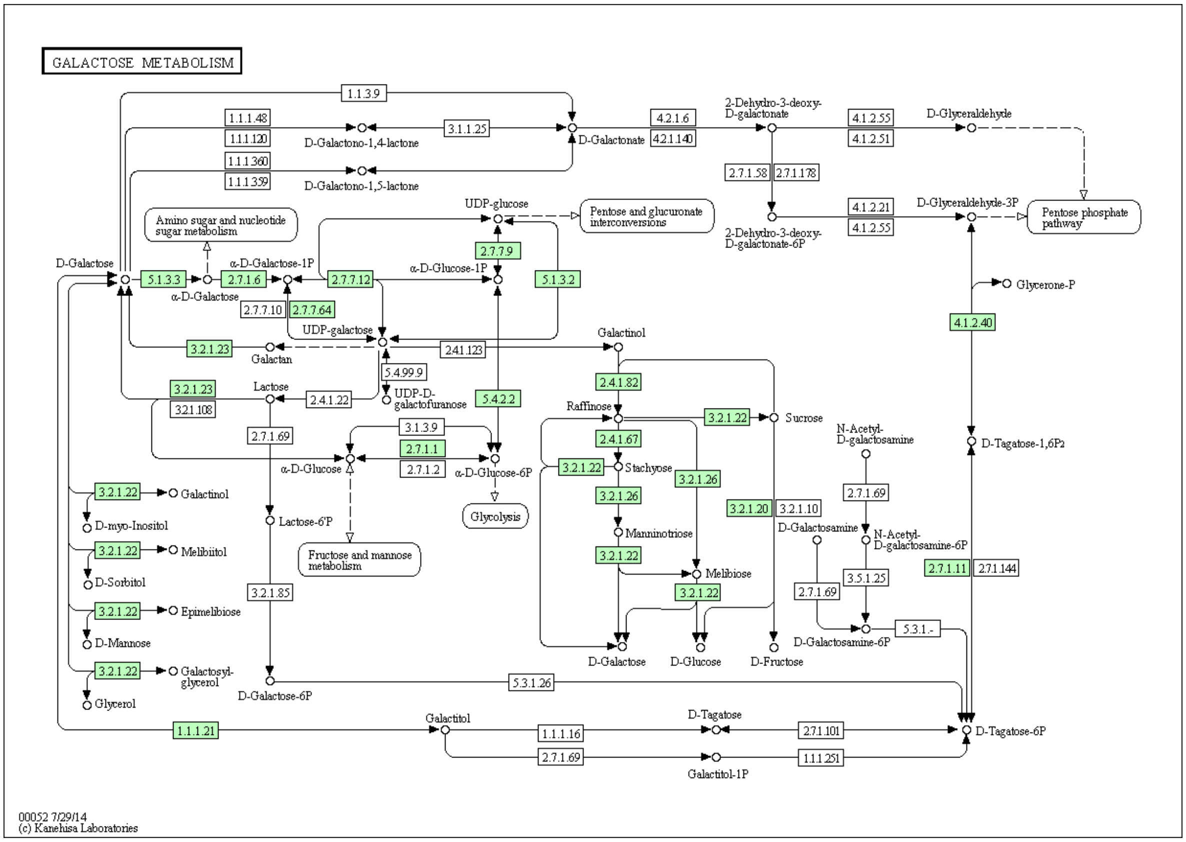
GALACTOSE METABOLISM
1.1.3.9
1.1.1.48
1.1.1.120
3.1.1.25
D-Galactono-1,4-lactone
1.1.1.360
1.1.1.359
D-Galactono-1,5-lactone
D-Galactonate
4.2.1.6
4.2.1.140
2-Dehydro-3-deoxy-D-galactonate
2.7.1.58
2.7.1.178
2-Dehydro-3-deoxy-D-galactonate-6P
4.1.2.55
4.1.2.51
D-Glyceraldehyde
4.1.2.21
4.1.2.55
D-Glyceraldehyde-3P
Pentose phosphate pathway
Amino sugar and nucleotide sugar metabolism
UDP-glucose
Pentose and glucuronate interconversions
2.7.7.9
5.1.3.2
D-Galactose
5.1.3.3
α-D-Galactose
2.7.1.6
α-D-Galactose-1P
2.7.7.12
α-D-Glucose-1P
2.7.7.10
2.7.7.64
UDP-galactose
3.2.1.23
Galactan
2.4.1.123
Galactinol
5.4.99.9
UDP-D-galactofuranose
5.4.2.2
3.2.1.23
3.2.1.108
Lactose
2.4.1.22
2.7.1.69
3.1.3.9
2.7.1.1
2.7.1.2
α-D-Glucose
α-D-Glucose-6P
Glycolysis
Lactose-6P
Fructose and mannose metabolism
3.2.1.85
D-Galactose-6P
5.3.1.26
3.2.1.22
Galactinol
D-myo-Inositol
3.2.1.22
Melibiitol
D-Sorbitol
3.2.1.22
Epimelibiose
D-Mannose
3.2.1.22
Galactosyl-glycerol
Glycerol
1.1.1.21
Galactitol
1.1.1.16
2.7.1.69
D-Tagatose
Galactitol-1P
2.7.1.101
1.1.1.251
D-Tagatose-6P
2.4.1.82
Raffinose
3.2.1.22
Sucrose
2.4.1.67
3.2.1.22
Stachyose
3.2.1.26
3.2.1.26
Manninotriose
3.2.1.22
Melibiose
3.2.1.22
3.2.1.20
3.2.1.10
D-Galactose
D-Glucose
D-Fructose
N-Acetyl-D-galactosamine
2.7.1.69
D-Galactosamine
N-Acetyl-D-galactosamine-6P
3.5.1.25
2.7.1.69
D-Galactosamine-6P
5.3.1.-
4.1.2.40
Glycerone-P
D-Tagatose-1,6P2
2.7.1.11
2.7.1.144
00052 7/29/14
(c) Kanehisa Laboratories

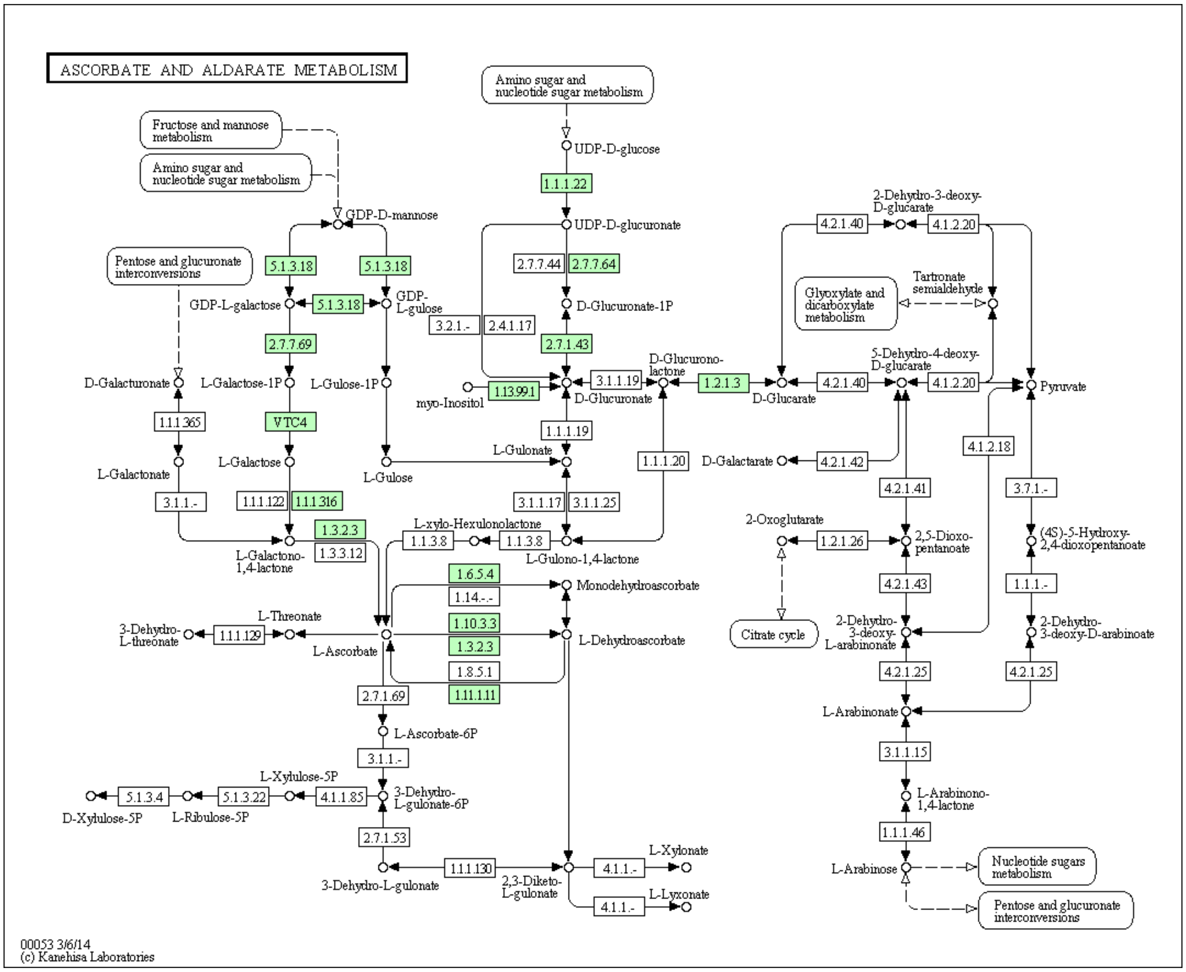

ASCORBATE AND ALDARATE METABOLISM
Amino sugar and nucleotide sugar metabolism
Fructose and mannose metabolism
Amino sugar and nucleotide sugar metabolism
Pentose and glucuronate interconversions
UDP-D-glucose
1.1.1.22
UDP-D-glucuronate
GDP-D-mannose
5.1.3.18
5.1.3.18
GDP-L-galactose
5.1.3.18
GDP-L-gulose
2.7.7.44
2.7.7.64
D-Glucuronate-1P
3.2.1.-
2.4.1.17
2.7.7.69
2.7.1.43
D-Galacturonate
L-Galactose-1P
L-Gulose-1P
myo-Inositol
1.13.99.1
3.1.1.19
D-Glucurono-lactone
1.2.1.3
D-Glucuronate
D-Glucarate
1.1.1.365
VTC4
1.1.1.19
L-Galactonate
L-Galactose
L-Gulose
L-Gulonate
1.1.1.20
3.1.1.-
1.1.1.122
1.1.1.316
3.1.1.17
3.1.1.25
1.3.2.3
1.3.3.12
L-xylo-Hexulonolactone
1.1.3.8
1.1.3.8
L-Galactono-1,4-lactone
L-Gulono-1,4-lactone
1.6.5.4
1.14.-.-
Monodehydroascorbate
L-Threonate
3-Dehydro-L-threonate
1.1.1.129
1.10.3.3
1.3.2.3
L-Ascorbate
L-Dehydroascorbate
1.8.5.1
1.11.1.11
2.7.1.69
L-Ascorbate-6P
3.1.1.-
L-Xylulose-5P
5.1.3.4
5.1.3.22
4.1.1.85
3-Dehydro-L-gulonate-6P
D-Xylulose-5P
L-Ribulose-5P
2.7.1.53
1.1.1.130
4.1.1.-
L-Xylonate
3-Dehydro-L-gulonate
2,3-Diketo-L-gulonate
4.1.1.-
L-Lyxonate
2-Dehydro-3-deoxy-D-glucarate
4.2.1.40
4.1.2.20
Tartronate semialdehyde
Glyoxylate and dicarboxylate metabolism
5-Dehydro-4-deoxy-D-glucarate
4.2.1.40
4.1.2.20
Pyruvate
D-Galactarate
4.2.1.42
4.1.2.18
4.2.1.41
3.7.1.-
2-Oxoglutarate
1.2.1.26
2,5-Dioxo-pentanoate
(4S)-5-Hydroxy-2,4-dioxopentanoate
4.2.1.43
1.1.1.-
Citrate cycle
2-Dehydro-3-deoxy-L-arabinoate
2-Dehydro-3-deoxy-D-arabinoate
4.2.1.25
4.2.1.25
L-Arabinonate
3.1.1.15
L-Arabinono-1,4-lactone
1.1.1.46
L-Arabinose
Nucleotide sugars metabolism
Pentose and glucuronate interconversions
00053 3/6/14
(c) Kanehisa Laboratories

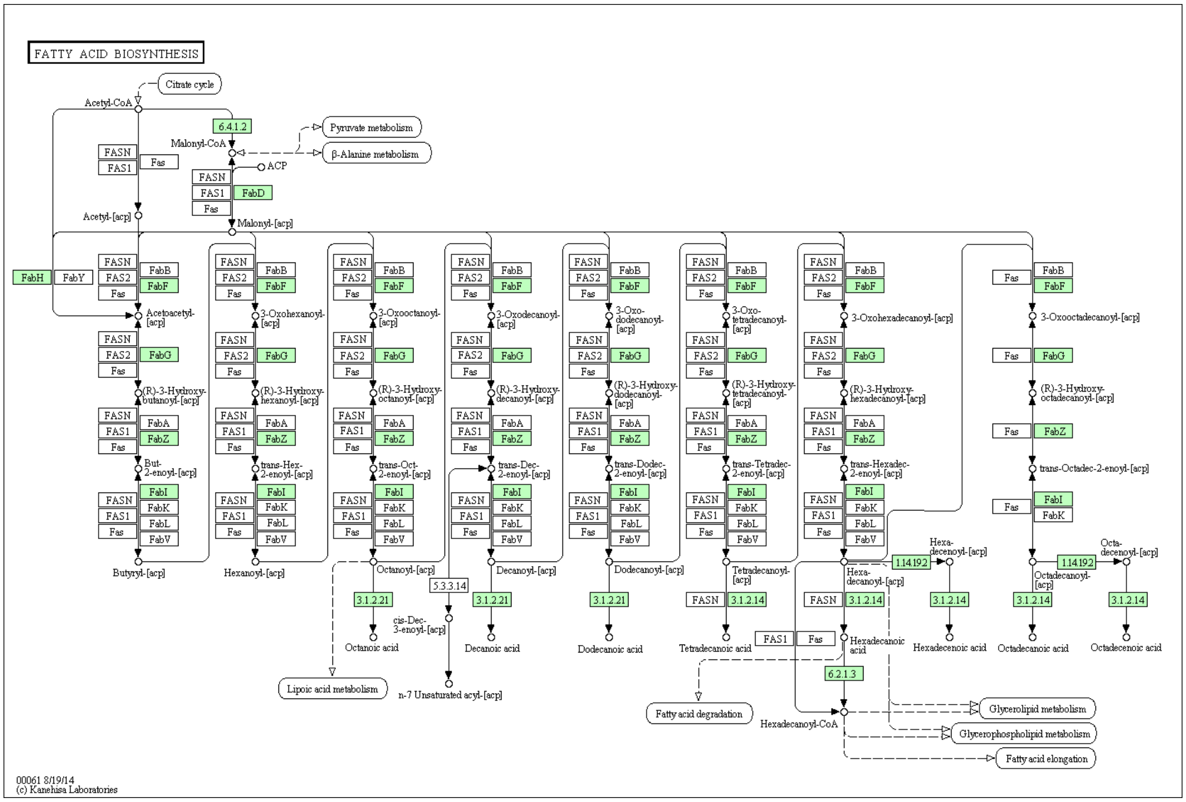

FATTY ACID BIOSYNTHESIS
Citrate cycle
Acetyl-CoA
6.4.1.2
Malonyl-CoA
Pyruvate metabolism
β-Alanine metabolism
ACP
FASN
FAS1
Fas
FAS2
FabD
FabH
FabY
FabB
FabF
FabG
FabA
FabZ
FabI
FabK
FabL
FabV
Acetyl-[acp]
Malonyl-[acp]
Acetoacetyl-[acp]
(R)-3-Hydroxy-butanoyl-[acp]
But-2-enoyl-[acp]
Butyryl-[acp]
3-Oxohexanoyl-[acp]
(R)-3-Hydroxy-hexanoyl-[acp]
trans-Hex-2-enoyl-[acp]
Hexanoyl-[acp]
3-Oxooctanoyl-[acp]
(R)-3-Hydroxy-octanoyl-[acp]
trans-Oct-2-enoyl-[acp]
Octanoyl-[acp]
3-Oxodecanoyl-[acp]
(R)-3-Hydroxy-decanoyl-[acp]
trans-Dec-2-enoyl-[acp]
Decanoyl-[acp]
3-Oxo-dodecanoyl-[acp]
(R)-3-Hydroxy-dodecanoyl-[acp]
trans-Dodec-2-enoyl-[acp]
Dodecanoyl-[acp]
3-Oxo-tetradecanoyl-[acp]
(R)-3-Hydroxy-tetradecanoyl-[acp]
trans-Tetradec-2-enoyl-[acp]
Tetradecanoyl-[acp]
3-Oxohexadecanoyl-[acp]
(R)-3-Hydroxy-hexadecanoyl-[acp]
trans-Hexadec-2-enoyl-[acp]
Hexa-decanoyl-[acp]
3-Oxooctadecanoyl-[acp]
(R)-3-Hydroxy-octadecanoyl-[acp]
trans-Octadec-2-enoyl-[acp]
Octadecanoyl-[acp]
Hexa-decenoyl-[acp]
Octa-decenoyl-[acp]
1.14.19.2
3.1.2.21
3.1.2.14
5.3.3.14
6.2.1.3
cis-Dec-3-enoyl-[acp]
n-7 Unsaturated acyl-[acp]
Octanoic acid
Decanoic acid
Dodecanoic acid
Tetradecanoic acid
Hexadecanoic acid
Hexadecenoic acid
Octadecanoic acid
Octadecenoic acid
Hexadecanoyl-CoA
Lipoic acid metabolism
Fatty acid degradation
Glycerolipid metabolism
Glycerophospholipid metabolism
Fatty acid elongation
00061 8/19/14
(c) Kanehisa Laboratories

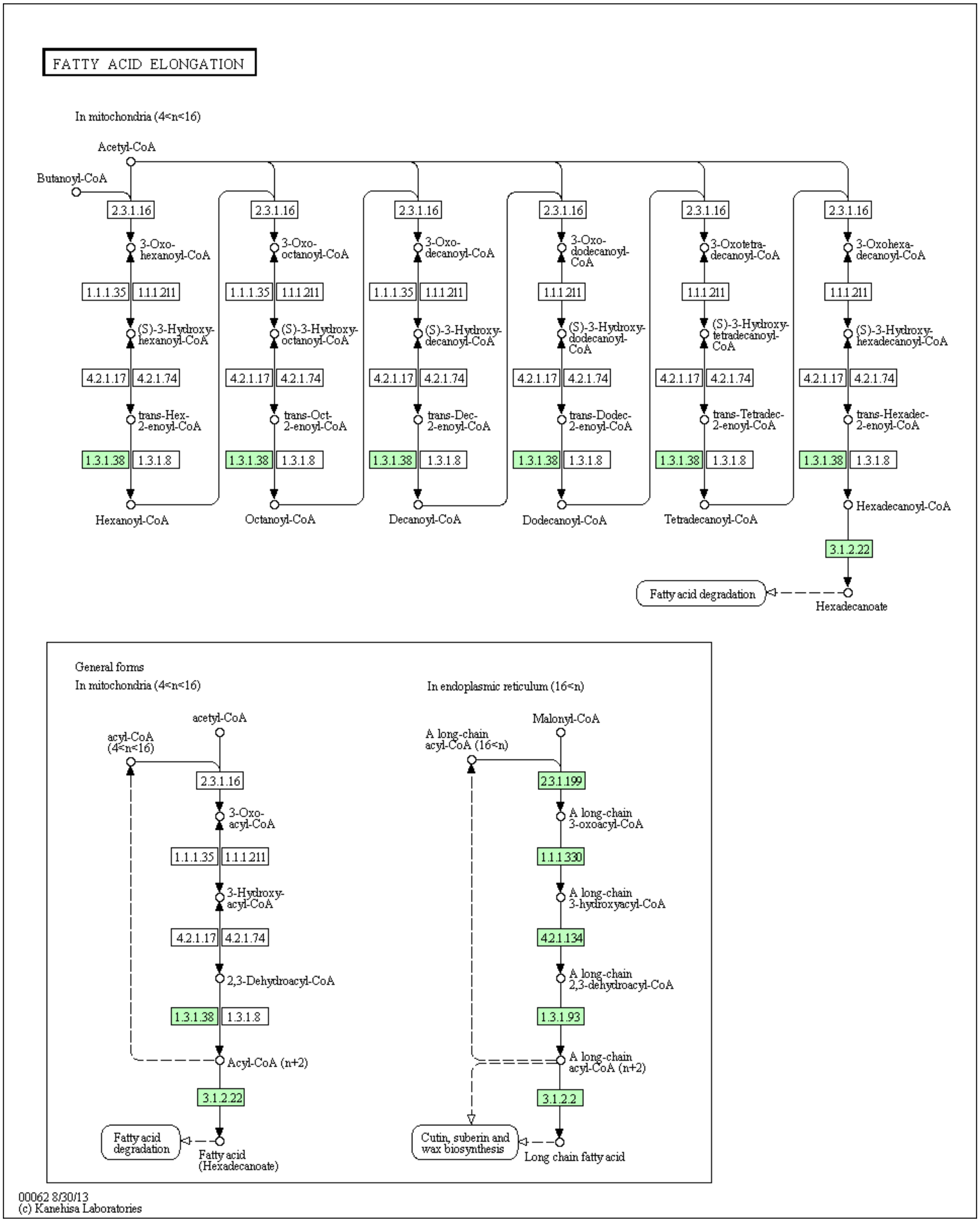

FATTY ACID ELONGATION
In mitochondria (4<n<16)
Acetyl-CoA
Butanoyl-CoA
2.3.1.16
3-Oxo-hexanoyl-CoA
3-Oxo-octanoyl-CoA
3-Oxo-decanoyl-CoA
3-Oxo-dodecanoyl-CoA
3-Oxotetra-decanoyl-CoA
3-Oxohexa-decanoyl-CoA
1.1.1.35
1.1.1.211
(S)-3-Hydroxy-hexanoyl-CoA
(S)-3-Hydroxy-octanoyl-CoA
(S)-3-Hydroxy-decanoyl-CoA
(S)-3-Hydroxy-dodecanoyl-CoA
(S)-3-Hydroxy-tetradecanoyl-CoA
(S)-3-Hydroxy-hexadecanoyl-CoA
4.2.1.17
4.2.1.74
trans-Hex-2-enoyl-CoA
trans-Oct-2-enoyl-CoA
trans-Dec-2-enoyl-CoA
trans-Dodec-2-enoyl-CoA
trans-Tetradec-2-enoyl-CoA
trans-Hexadec-2-enoyl-CoA
1.3.1.38
1.3.1.8
Hexanoyl-CoA
Octanoyl-CoA
Decanoyl-CoA
Dodecanoyl-CoA
Tetradecanoyl-CoA
Hexadecanoyl-CoA
3.1.2.22
Fatty acid degradation
Hexadecanoate
General forms
In mitochondria (4<n<16)
acetyl-CoA
acyl-CoA (4<n<16)
2.3.1.16
3-Oxo-acyl-CoA
1.1.1.35
1.1.1.211
3-Hydroxy-acyl-CoA
4.2.1.17
4.2.1.74
2,3-Dehydroacyl-CoA
1.3.1.38
1.3.1.8
Acyl-CoA (n+2)
3.1.2.22
Fatty acid degradation
Fatty acid (Hexadecanoate)
In endoplasmic reticulum (16<n)
Malonyl-CoA
A long-chain acyl-CoA (16<n)
2.3.1.199
A long-chain 3-oxoacyl-CoA
1.1.1.330
A long-chain 3-hydroxyacyl-CoA
4.2.1.134
A long-chain 2,3-dehydroacyl-CoA
1.3.1.93
A long-chain acyl-CoA (n+2)
3.1.2.2
Cutin, suberin and wax biosynthesis
Long chain fatty acid
00062 8/30/13
(c) Kanehisa Laboratories

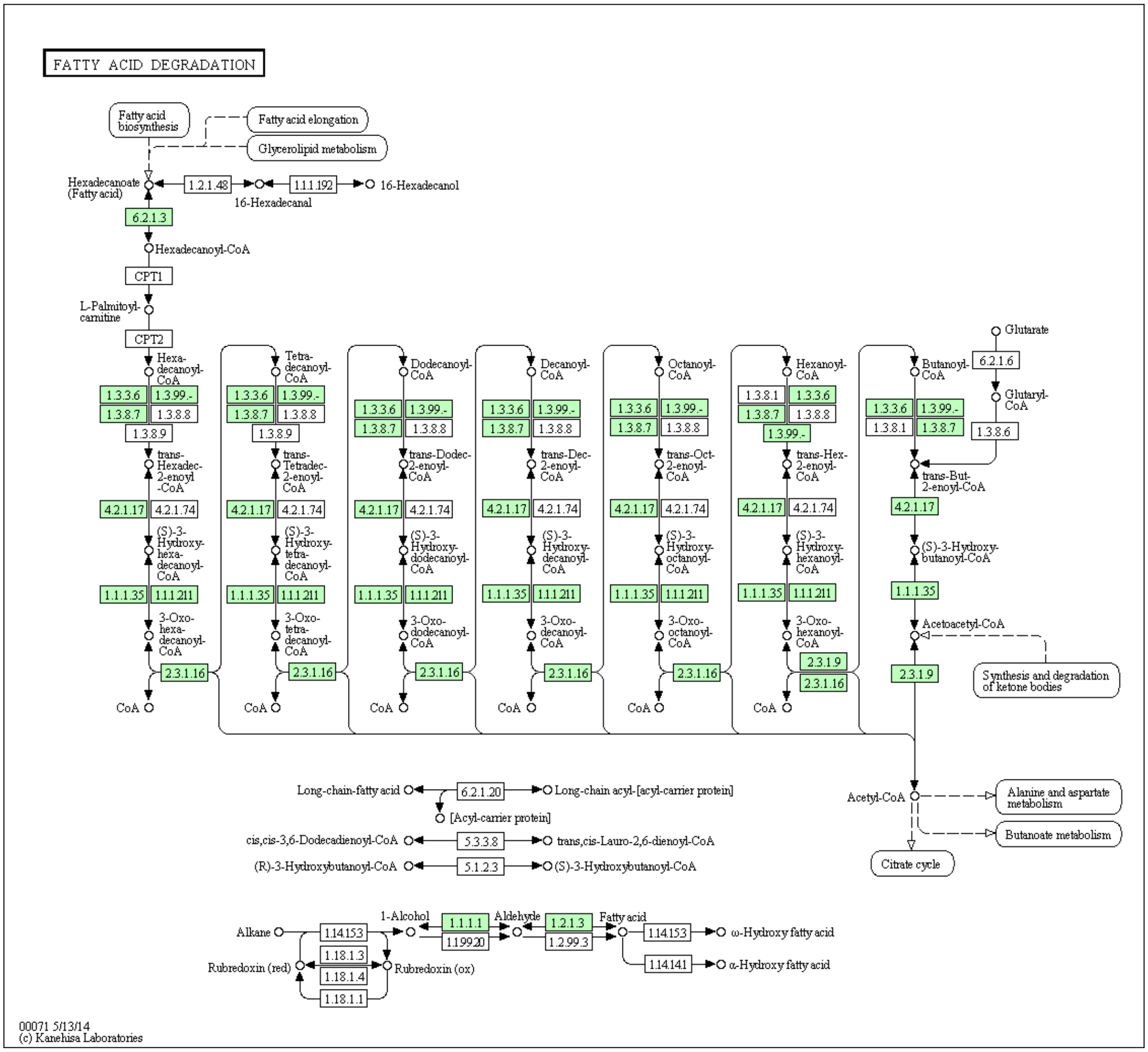
FATTY ACID DEGRADATION
Fatty acid biosynthesis
Fatty acid elongation
Glycerolipid metabolism
Hexadecanoate (Fatty acid)
16-Hexadecanal
16-Hexadecanol
Hexadecanoyl-CoA
CPT1
L-Palmitoyl-carnitine
CPT2
Hexa-decanoyl-CoA
Tetra-decanoyl-CoA
Dodecanoyl-CoA
Decanoyl-CoA
Octanoyl-CoA
Hexanoyl-CoA
Butanoyl-CoA
Glutarate
Glutaryl-CoA
trans-Hexadec-2-enoyl-CoA
trans-Tetradec-2-enoyl-CoA
trans-Dodec-2-enoyl-CoA
trans-Dec-2-enoyl-CoA
trans-Oct-2-enoyl-CoA
trans-Hex-2-enoyl-CoA
trans-But-2-enoyl-CoA
(S)-3-Hydroxy-hexa-decanoyl-CoA
(S)-3-Hydroxy-tetra-decanoyl-CoA
(S)-3-Hydroxy-dodecanoyl-CoA
(S)-3-Hydroxy-decanoyl-CoA
(S)-3-Hydroxy-octanoyl-CoA
(S)-3-Hydroxy-hexanoyl-CoA
(S)-3-Hydroxy-butanoyl-CoA
3-Oxo-hexa-decanoyl-CoA
3-Oxo-tetra-decanoyl-CoA
3-Oxo-dodecanoyl-CoA
3-Oxo-decanoyl-CoA
3-Oxo-octanoyl-CoA
3-Oxo-hexanoyl-CoA
Acetoacetyl-CoA
Synthesis and degradation of ketone bodies
CoA
Acetyl-CoA
Alanine and aspartate metabolism
Butanoate metabolism
Citrate cycle
Long-chain-fatty acid
Long-chain acyl-[acyl-carrier protein]
[Acyl-carrier protein]
cis,cis-3,6-Dodecadienoyl-CoA
trans,cis-Lauro-2,6-dienoyl-CoA
(R)-3-Hydroxybutanoyl-CoA
(S)-3-Hydroxybutanoyl-CoA
Alkane
1-Alcohol
Aldehyde
Fatty acid
ω-Hydroxy fatty acid
α-Hydroxy fatty acid
Rubredoxin (red)
Rubredoxin (ox)
1.2.1.48
1.1.1.192
6.2.1.3
1.3.3.6
1.3.99.-
1.3.8.7
1.3.8.8
1.3.8.9
1.3.8.1
1.3.8.6
6.2.1.6
4.2.1.17
4.2.1.74
1.1.1.35
1.1.1.211
2.3.1.16
2.3.1.9
6.2.1.20
5.3.3.8
5.1.2.3
1.14.15.3
1.18.1.3
1.18.1.4
1.18.1.1
1.1.1.1
1.1.99.20
1.2.1.3
1.2.99.3
1.14.14.1
00071 5/13/14
(c) Kanehisa Laboratories

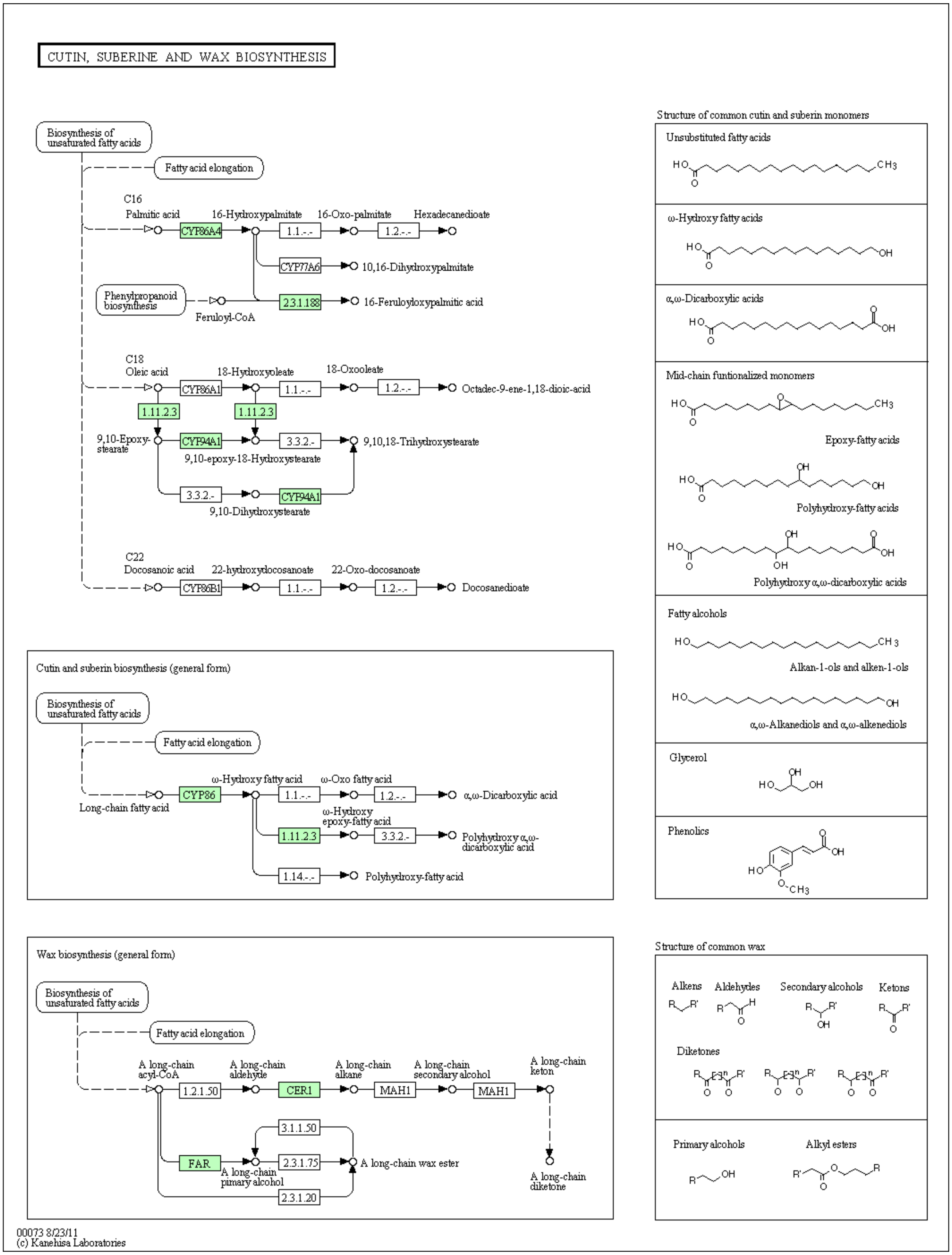
CUTIN, SUBERINE AND WAX BIOSYNTHESIS
Biosynthesis of unsaturated fatty acids
Fatty acid elongation
C16
Palmitic acid
CYP86A4
16-Hydroxypalmitate
1.1.-.-
16-Oxo-palmitate
1.2.-.-
Hexadecanedioate
CYP77A6
10,16-Dihydroxypalmitate
Phenylpropanoid biosynthesis
Feruloyl-CoA
2.3.1.188
16-Feruloyloxypalmitic acid
C18
Oleic acid
CYP86A1
18-Hydroxyoleate
1.1.-.-
18-Oxooleate
1.2.-.-
Octadec-9-ene-1,18-dioic-acid
1.11.2.3
1.11.2.3
9,10-Epoxy-stearate
CYP94A1
9,10-epoxy-18-Hydroxystearate
3.3.2.-
9,10,18-Trihydroxystearate
3.3.2.-
CYP94A1
9,10-Dihydroxystearate
C22
Docosanoic acid
CYP86B1
22-hydroxydocosanoate
1.1.-.-
22-Oxo-docosanoate
1.2.-.-
Docosanedioate
Cutin and suberin biosynthesis (general form)
Biosynthesis of unsaturated fatty acids
Fatty acid elongation
Long-chain fatty acid
CYP86
ω-Hydroxy fatty acid
1.1.-.-
ω-Oxo fatty acid
1.2.-.-
α,ω-Dicarboxylic acid
ω-Hydroxy epoxy-fatty acid
1.11.2.3
3.3.2.-
Polyhydroxy α,ω-dicarboxylic acid
1.14.-.-
Polyhydroxy-fatty acid
Wax biosynthesis (general form)
Biosynthesis of unsaturated fatty acids
Fatty acid elongation
A long-chain acyl-CoA
1.2.1.50
A long-chain aldehyde
CER1
A long-chain alkane
MAH1
A long-chain secondary alcohol
MAH1
A long-chain keton
A long-chain diketone
FAR
A long-chain primary alcohol
3.1.1.50
2.3.1.75
2.3.1.20
A long-chain wax ester
Structure of common cutin and suberin monomers
Unsubstituted fatty acids
ω-Hydroxy fatty acids
α,ω-Dicarboxylic acids
Mid-chain funtionalized monomers
Epoxy-fatty acids
Polyhydroxy-fatty acids
Polyhydroxy α,ω-dicarboxylic acids
Fatty alcohols
Alkan-1-ols and alken-1-ols
α,ω-Alkanediols and α,ω-alkenediols
Glycerol
Phenolics
Structure of common wax
Alkens
Aldehydes
Secondary alcohols
Ketons
Diketones
Primary alcohols
Alkyl esters
00073 8/23/11
(c) Kanehisa Laboratories

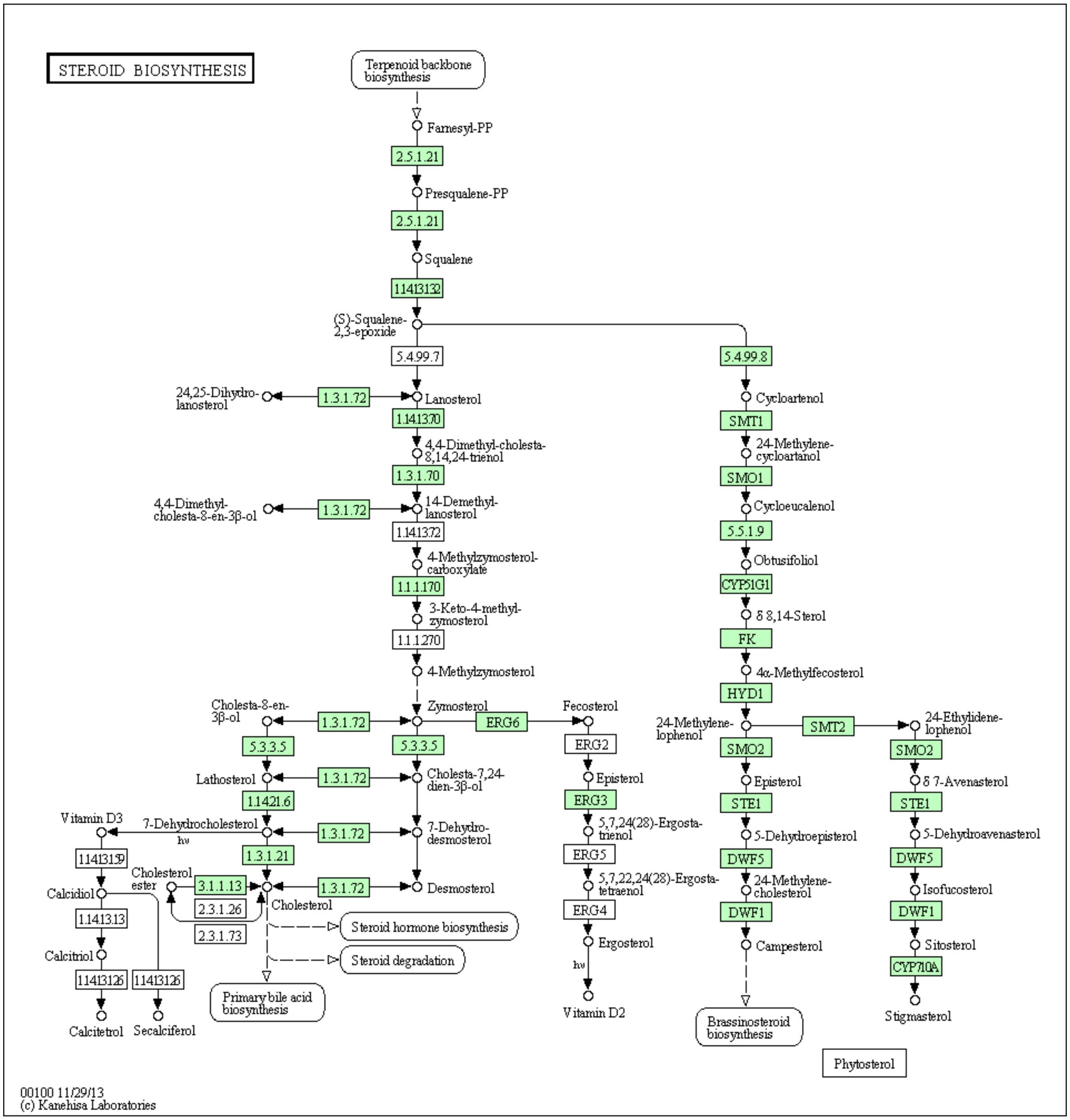
STEROID BIOSYNTHESIS
Terpenoid backbone biosynthesis
Farnesyl-PP
2.5.1.21
Presqualene-PP
2.5.1.21
Squalene
1.14.13.132
(S)-Squalene-2,3-epoxide
5.4.99.7
5.4.99.8
24,25-Dihydro-lanosterol
1.3.1.72
Lanosterol
Cycloartenol
1.14.13.70
SMT1
4,4-Dimethyl-cholesta-8,14,24-trienol
24-Methylene-cycloartanol
1.3.1.70
SMO1
4,4-Dimethyl-cholesta-8-en-3β-ol
1.3.1.72
14-Demethyl-lanosterol
Cycloeucalenol
1.14.13.72
5.5.1.9
4-Methylzymosterol-carboxylate
Obtusifoliol
1.1.1.170
CYP51G1
3-Keto-4-methyl-zymosterol
δ 8,14-Sterol
1.1.1.270
FK
4-Methylzymosterol
4α-Methylfecosterol
HYD1
Cholesta-8-en-3β-ol
1.3.1.72
Zymosterol
ERG6
Fecosterol
24-Methylene-lophenol
SMT2
24-Ethylidene-lophenol
5.3.3.5
5.3.3.5
ERG2
SMO2
SMO2
Lathosterol
1.3.1.72
Cholesta-7,24-dien-3β-ol
Episterol
Episterol
δ 7-Avenasterol
1.14.21.6
ERG3
STE1
STE1
Vitamin D3
7-Dehydrocholesterol
hν
1.3.1.72
7-Dehydro-desmosterol
5,7,24(28)-Ergosta-trienol
5-Dehydroepisterol
5-Dehydroavenasterol
1.14.13.159
1.3.1.21
ERG5
DWF5
DWF5
Calcidiol
Cholesterol ester
3.1.1.13
1.3.1.72
Desmosterol
5,7,22,24(28)-Ergosta-tetraenol
24-Methylene-cholesterol
Isofucosterol
2.3.1.26
Cholesterol
ERG4
DWF1
DWF1
1.14.13.13
2.3.1.73
Steroid hormone biosynthesis
Ergosterol
Campesterol
Sitosterol
Calcitriol
Steroid degradation
CYP710A
hν
1.14.13.126
1.14.13.126
Primary bile acid biosynthesis
Vitamin D2
Brassinosteroid biosynthesis
Stigmasterol
Calcitetrol
Secalciferol
Phytosterol
00100 11/29/13
(c) Kanehisa Laboratories

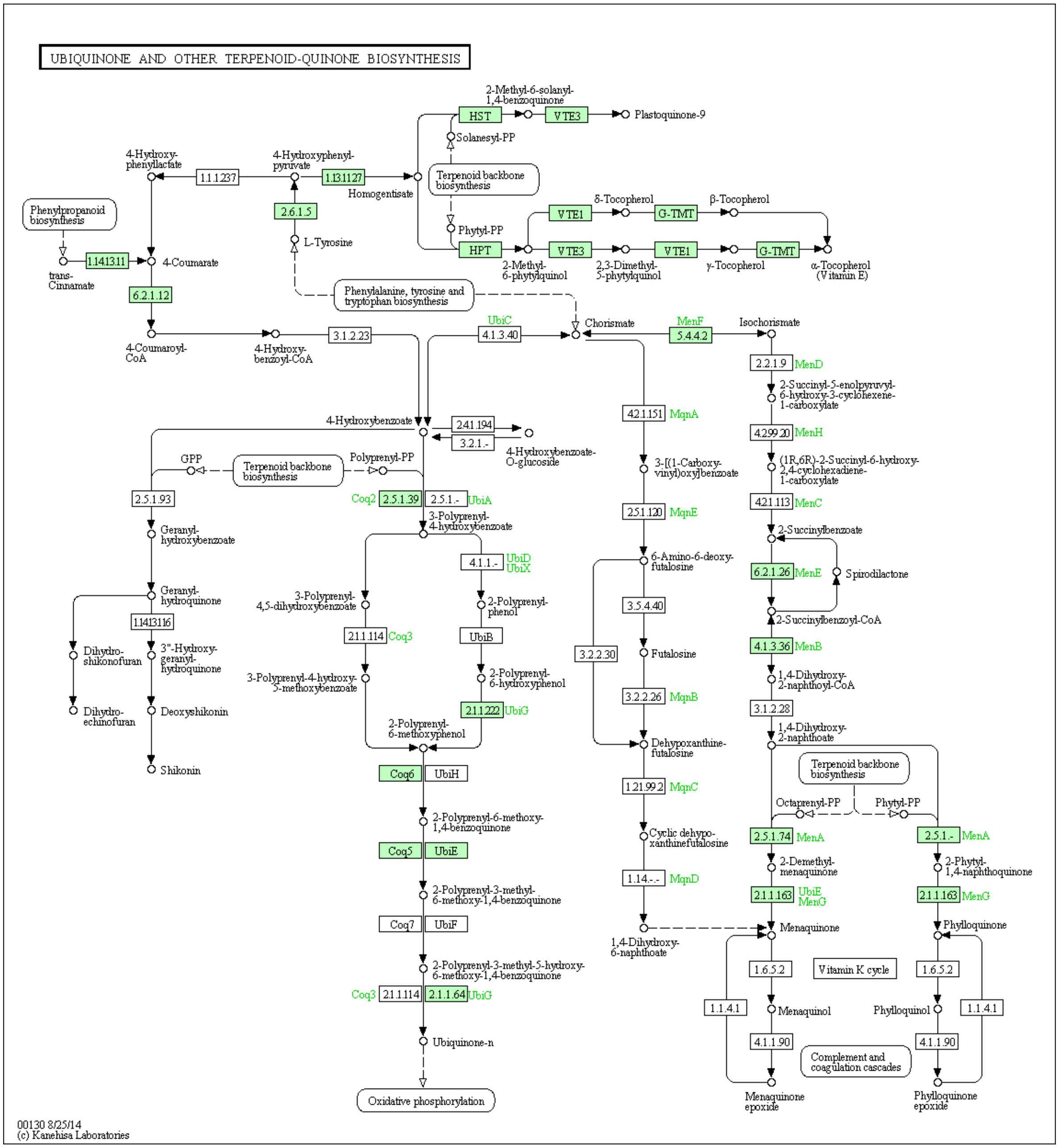

UBIQUINONE AND OTHER TERPENOID-QUINONE BIOSYNTHESIS
2-Methyl-6-solanyl-1,4-benzoquinone
HST
VTE3
Plastoquinone-9
Solanesyl-PP
4-Hydroxy-phenyllactate
4-Hydroxyphenyl-pyruvate
1.1.1.237
1.13.11.27
Homogentisate
Terpenoid backbone biosynthesis
Phenylpropanoid biosynthesis
2.6.1.5
L-Tyrosine
δ-Tocopherol
β-Tocopherol
VTE1
G-TMT
Phytyl-PP
HPT
VTE3
VTE1
G-TMT
trans-Cinnamate
1.14.13.11
4-Coumarate
2-Methyl-6-phytylquinol
2,3-Dimethyl-5-phytylquinol
γ-Tocopherol
α-Tocopherol (Vitamin E)
6.2.1.12
Phenylalanine, tyrosine and tryptophan biosynthesis
UbiC
MenF
Chorismate
Isochorismate
4-Coumaroyl-CoA
4-Hydroxy-benzoyl-CoA
3.1.2.23
4.1.3.40
5.4.4.2
2.2.1.9
MenD
2-Succinyl-5-enolpyruvyl-6-hydroxy-3-cyclohexene-1-carboxylate
4.2.1.151
MqnA
4-Hydroxybenzoate
2.4.1.194
3.2.1.-
4.2.99.20
MenH
4-Hydroxybenzoate-O-glucoside
GPP
Terpenoid backbone biosynthesis
Polyprenyl-PP
3-[(1-Carboxy-vinyl)oxy]benzoate
(1R,6R)-2-Succinyl-6-hydroxy-2,4-cyclohexadiene-1-carboxylate
2.5.1.93
Coq2
2.5.1.39
2.5.1.-
UbiA
4.2.1.113
MenC
3-Polyprenyl-4-hydroxybenzoate
2.5.1.120
MqnE
Geranyl-hydroxybenzoate
2-Succinylbenzoate
4.1.1.-
UbiD
UbiX
6-Amino-6-deoxy-futalosine
6.2.1.26
MenE
Spirodilactone
Geranyl-hydroquinone
3-Polyprenyl-4,5-dihydroxybenzoate
2-Polyprenyl-phenol
1.14.13.116
3.5.4.40
2-Succinylbenzoyl-CoA
2.1.1.114
Coq3
UbiB
Dihydro-shikonofuran
3"-Hydroxy-geranyl-hydroquinone
3.2.2.30
Futalosine
4.1.3.36
MenB
3-Polyprenyl-4-hydroxy-5-methoxybenzoate
2-Polyprenyl-6-hydroxyphenol
1,4-Dihydroxy-2-naphthoyl-CoA
3.2.2.26
MqnB
Dihydro-echinofuran
Deoxyshikonin
2.1.1.222
UbiG
3.1.2.28
2-Polyprenyl-6-methoxyphenol
1,4-Dihydroxy-2-naphthoate
Dehypoxanthine-futalosine
Coq6
UbiH
Shikonin
Terpenoid backbone biosynthesis
1.21.99.2
MqnC
Octaprenyl-PP
Phytyl-PP
2-Polyprenyl-6-methoxy-1,4-benzoquinone
Cyclic dehypo-xanthinefutalosine
2.5.1.74
MenA
2.5.1.-
MenA
Coq5
UbiE
2-Demethyl-menaquinone
2-Phytyl-1,4-naphthoquinone
2-Polyprenyl-3-methyl-6-methoxy-1,4-benzoquinone
1.14.-.-
MqnD
2.1.1.163
UbiE
MenG
2.1.1.163
MenG
Coq7
UbiF
Menaquinone
Phylloquinone
1,4-Dihydroxy-6-naphthoate
2-Polyprenyl-3-methyl-5-hydroxy-6-methoxy-1,4-benzoquinone
1.6.5.2
Vitamin K cycle
1.6.5.2
Coq3
2.1.1.114
2.1.1.64
UbiG
1.1.4.1
Menaquinol
Phylloquinol
1.1.4.1
Ubiquinone-n
4.1.1.90
4.1.1.90
Complement and coagulation cascades
Oxidative phosphorylation
Menaquinone epoxide
Phylloquinone epoxide
00130 8/25/14
(c) Kanehisa Laboratories

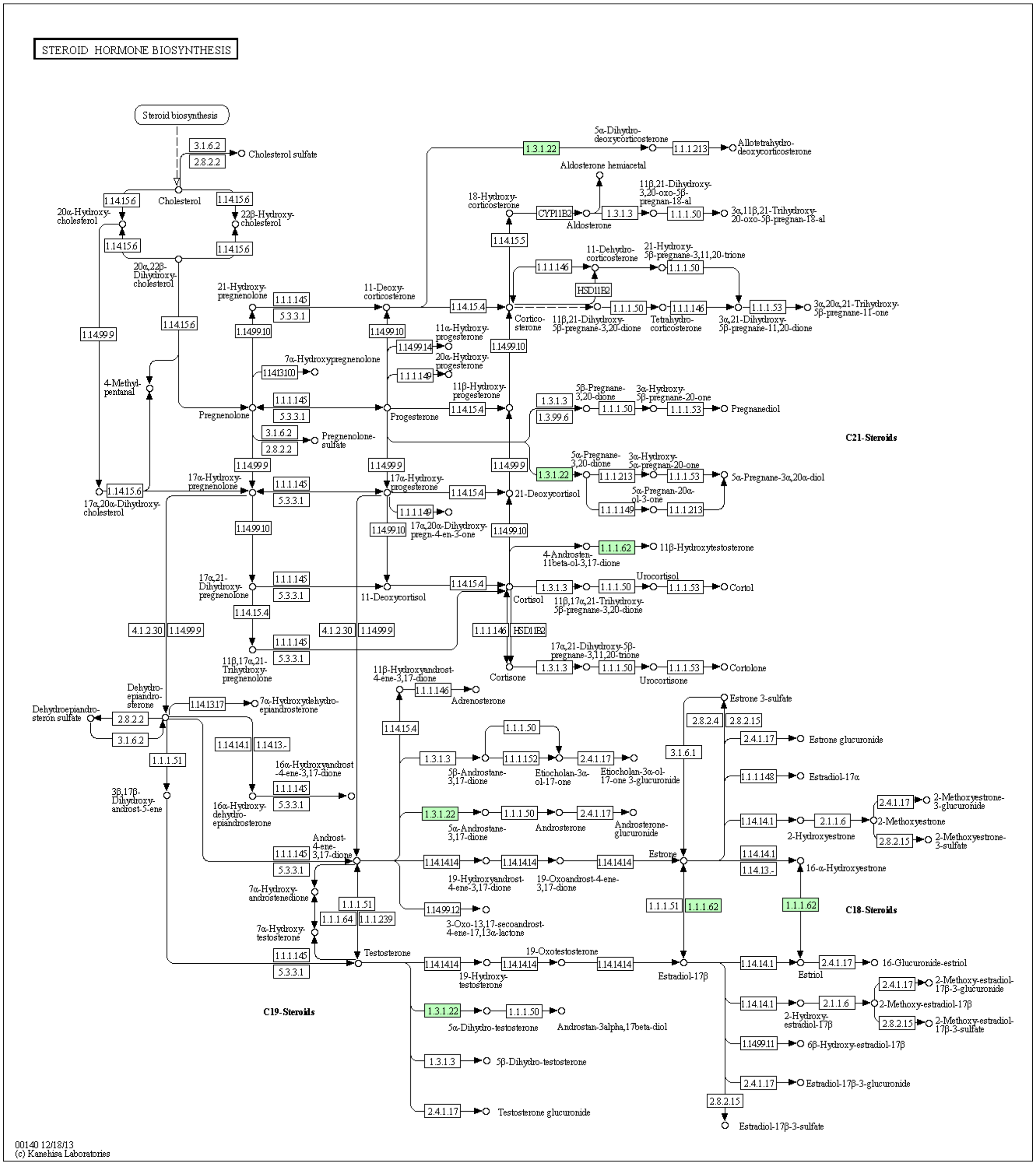
STEROID HORMONE BIOSYNTHESIS
Steroid biosynthesis
3.1.6.2
2.8.2.2
Cholesterol sulfate
Cholesterol
1.14.15.6
20α-Hydroxy-cholesterol
22β-Hydroxy-cholesterol
20α,22β-Dihydroxy-cholesterol
1.14.99.9
4-Methyl-pentanal
17α,20α-Dihydroxy-cholesterol
21-Hydroxy-pregnenolone
1.1.1.145
5.3.3.1
1.14.99.10
7α-Hydroxypregnenolone
1.14.13.100
Pregnenolone
Pregnenolone-sulfate
17α-Hydroxy-pregnenolone
17α,21-Dihydroxy-pregnenolone
1.14.15.4
11β,17α,21-Trihydroxy-pregnenolone
4.1.2.30
11-Deoxy-corticosterone
11α-Hydroxy-progesterone
1.14.99.14
20α-Hydroxy-progesterone
1.1.1.149
11β-Hydroxy-progesterone
Progesterone
17α-Hydroxy-progesterone
17α,20α-Dihydroxy-pregn-4-en-3-one
11-Deoxycortisol
5α-Dihydro-deoxycorticosterone
1.3.1.22
1.1.1.213
Allotetrahydro-deoxycorticosterone
Aldosterone hemiacetal
18-Hydroxy-corticosterone
CYP11B2
Aldosterone
1.3.1.3
11β,21-Dihydroxy-3,20-oxo-5β-pregnan-18-al
1.1.1.50
3α,11β,21-Trihydroxy-20-oxo-5β-pregnan-18-al
1.14.15.5
1.1.1.146
11-Dehydro-corticosterone
21-Hydroxy-5β-pregnane-3,11,20-trione
HSD11B2
Cortico-sterone
11β,21-Dihydroxy-5β-pregnane-3,20-dione
Tetrahydro-corticosterone
3α,21-Dihydroxy-5β-pregnane-11,20-dione
1.1.1.53
3α,20α,21-Trihydroxy-5β-pregnane-11-one
1.3.99.6
5β-Pregnane-3,20-dione
3α-Hydroxy-5β-pregnane-20-one
Pregnanediol
C21-Steroids
5α-Pregnane-3,20-dione
3α-Hydroxy-5α-pregnan-20-one
5α-Pregnane-3α,20α-diol
5α-Pregnan-20α-ol-3-one
21-Deoxycortisol
4-Androsten-11beta-ol-3,17-dione
1.1.1.62
11β-Hydroxytestosterone
Cortisol
Urocortisol
Cortol
11β,17α,21-Trihydroxy-5β-pregnane-3,20-dione
17α,21-Dihydroxy-5β-pregnane-3,11,20-trione
Cortisone
Urocortisone
Cortolone
Dehydro-epiandro-sterone
Dehydroepiandro-steron sulfate
1.14.13.17
7α-Hydroxydehydro-epiandrosterone
1.14.14.1
1.14.13.-
1.1.1.51
3β,17β-Dihydroxy-androst-5-ene
16α-Hydroxyandrost-4-ene-3,17-dione
16α-Hydroxy-dehydro-epiandrosterone
11β-Hydroxyandrost-4-ene-3,17-dione
Adrenosterone
5β-Androstane-3,17-dione
1.1.1.152
Etiocholan-3α-ol-17-one
2.4.1.17
Etiocholan-3α-ol-17-one 3-glucuronide
5α-Androstane-3,17-dione
Androsterone
Androsterone-glucuronide
Androst-4-ene-3,17-dione
7α-Hydroxy-androstenedione
7α-Hydroxy-testosterone
1.1.1.64
1.1.1.239
Testosterone
1.14.14.14
19-Hydroxyandrost-4-ene-3,17-dione
19-Oxoandrost-4-ene-3,17-dione
1.14.99.12
3-Oxo-13,17-secoandrost-4-ene-17,13α-lactone
19-Hydroxy-testosterone
19-Oxotestosterone
5α-Dihydro-testosterone
Androstan-3alpha,17beta-diol
5β-Dihydro-testosterone
Testosterone glucuronide
C19-Steroids
Estrone 3-sulfate
2.8.2.4
2.8.2.15
3.1.6.1
Estrone glucuronide
1.1.1.148
Estradiol-17α
Estrone
2-Hydroxyestrone
2.1.1.6
2-Methoxyestrone
2-Methoxyestrone-3-glucuronide
2-Methoxyestrone-3-sulfate
16-α-Hydroxyestrone
C18-Steroids
Estradiol-17β
Estriol
16-Glucuronide-estriol
2-Hydroxy-estradiol-17β
2-Methoxy-estradiol-17β
2-Methoxy-estradiol-17β-3-glucuronide
2-Methoxy-estradiol-17β-3-sulfate
1.14.99.11
6β-Hydroxy-estradiol-17β
Estradiol-17β-3-glucuronide
Estradiol-17β-3-sulfate
00140 12/18/13
(c) Kanehisa Laboratories

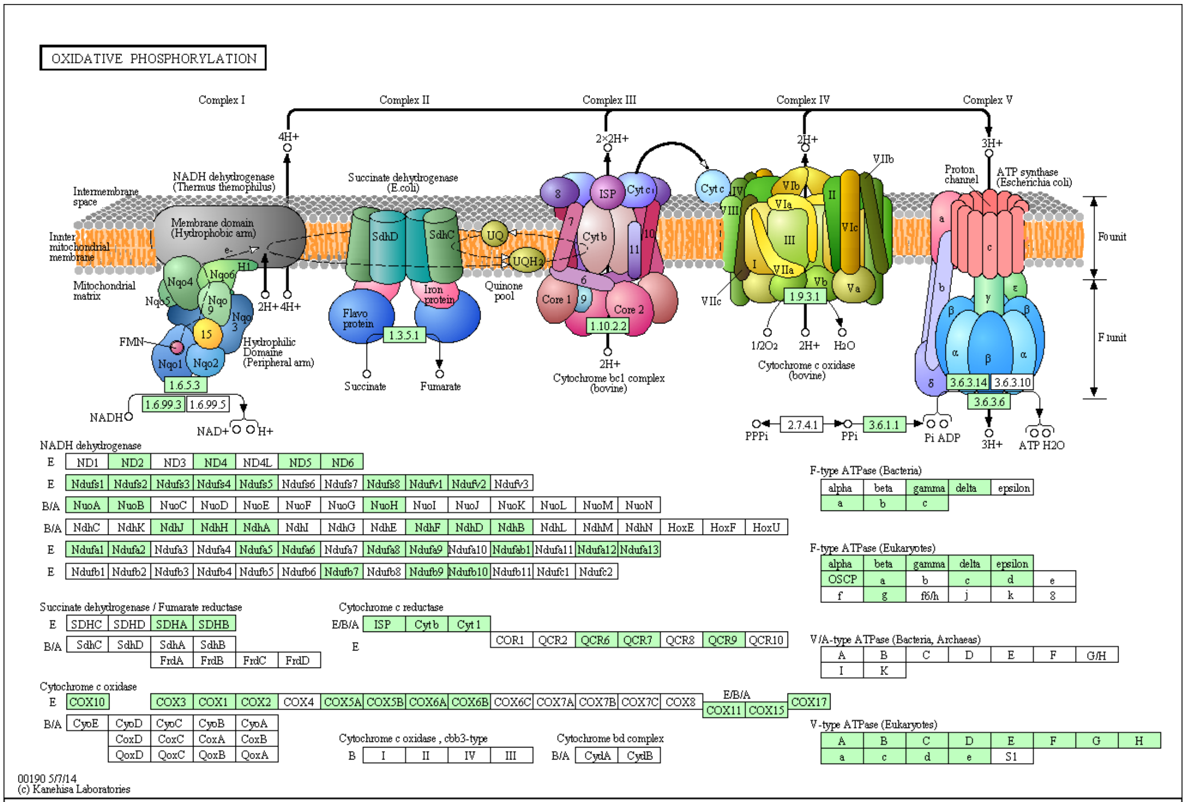
OXIDATIVE PHOSPHORYLATION
Complex I
Complex II
Complex III
Complex IV
Complex V
NADH dehydrogenase (Thermus themophilus)
Succinate dehydrogenase (E.coli)
Cytochrome bc1 complex (bovine)
Cytochrome c oxidase (bovine)
ATP synthase (Escherichia coli)
Intermembrane space
Inner mitochondrial membrane
Mitochondrial matrix
Membrane domain (Hydrophobic arm)
Hydrophilic Domain (Peripheral arm)
Quinone pool
Succinate
Fumarate
F0 unit
F1 unit
1.6.5.3
1.6.99.3
1.6.99.5
1.3.5.1
1.10.2.2
1.9.3.1
3.6.3.14
3.6.3.10
3.6.3.6
2.7.4.1
3.6.1.1
NADH dehydrogenase
ND1 ND2 ND3 ND4 ND4L ND5 ND6
Ndufs1 Ndufs2 Ndufs3 Ndufs4 Ndufs5 Ndufs6 Ndufs7 Ndufs8 Ndufv1 Ndufv2 Ndufv3
NuoA NuoB NuoC NuoD NuoE NuoF NuoG NuoH NuoI NuoJ NuoK NuoL NuoM NuoN
NdhC NdhK NdhJ NdhH NdhA NdhI NdhG NdhE NdhF NdhD NdhB NdhL NdhM NdhN HoxE HoxF HoxU
Ndufa1 Ndufa2 Ndufa3 Ndufa4 Ndufa5 Ndufa6 Ndufa7 Ndufa8 Ndufa9 Ndufa10 Ndufab1 Ndufa11 Ndufa12 Ndufa13
Ndufb1 Ndufb2 Ndufb3 Ndufb4 Ndufb5 Ndufb6 Ndufb7 Ndufb8 Ndufb9 Ndufb10 Ndufb11 Ndufc1 Ndufc2
Succinate dehydrogenase / Fumarate reductase
SDHC SDHD SDHA SDHB
SdhC SdhD SdhA SdhB
FrdA FrdB FrdC FrdD
Cytochrome c reductase
ISP Cytb Cyt1
COR1 QCR2 QCR6 QCR7 QCR8 QCR9 QCR10
Cytochrome c oxidase
COX10 COX3 COX1 COX2 COX4 COX5A COX5B COX6A COX6B COX6C COX7A COX7B COX7C COX8 COX11 COX15 COX17
CyoE CyoD CyoC CyoB CyoA
CoxD CoxC CoxA CoxB
QoxD QoxC QoxB QoxA
Cytochrome c oxidase , cbb3-type
I II IV III
Cytochrome bd complex
CydA CydB
F-type ATPase (Bacteria)
alpha beta gamma delta epsilon
a b c
F-type ATPase (Eukaryotes)
alpha beta gamma delta epsilon
OSCP a b c d e
f g f6/h j k 8
V/A-type ATPase (Bacteria, Archaeas)
A B C D E F G/H
I K
V-type ATPase (Eukaryotes)
A B C D E F G H
a c d e S1
00190 5/7/14
(c) Kanehisa Laboratories

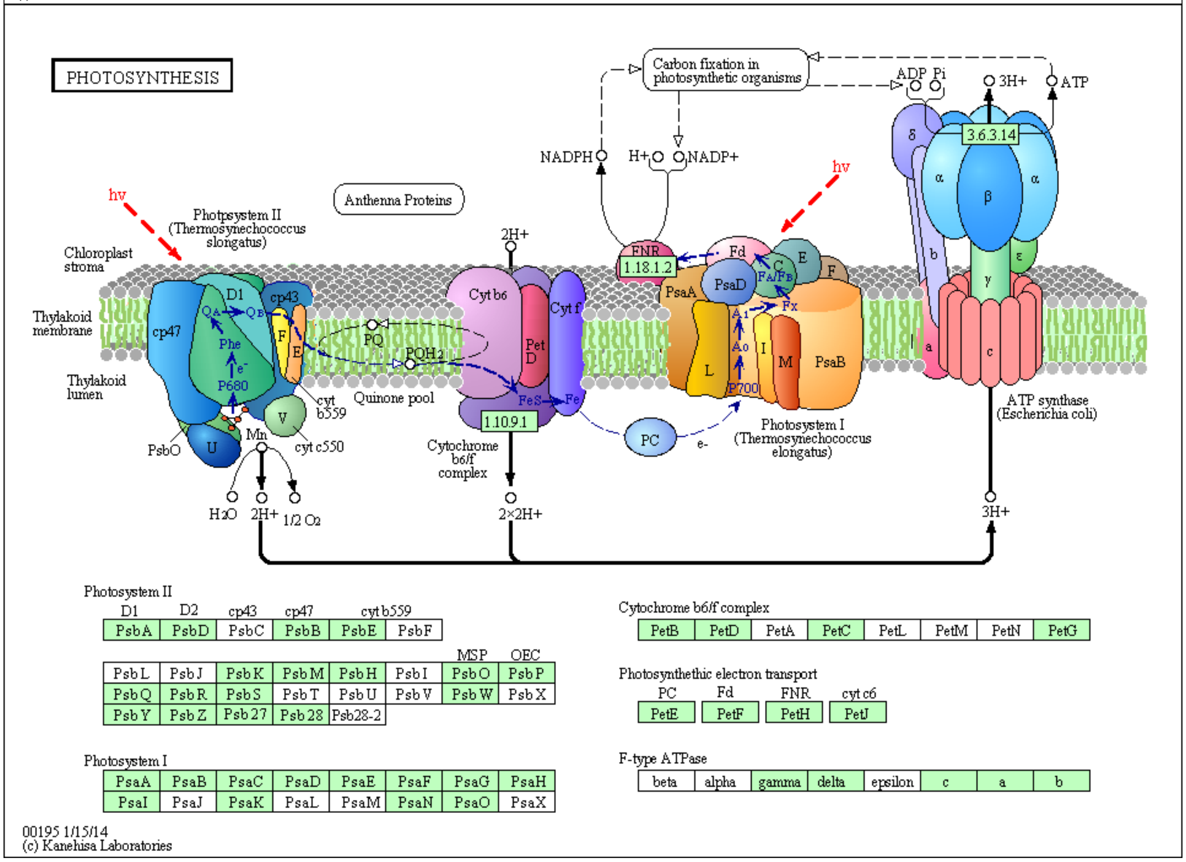
PHOTOSYNTHESIS
Carbon fixation in photosynthetic organisms
Anthenna Proteins
Photpsystem II (Thermosynechococcus slongatus)
Cytochrome b6/f complex
Photosystem I (Thermosynechococcus elongatus)
ATP synthase (Escherichia coli)
Chloroplast stroma
Thylakoid membrane
Thylakoid lumen
Quinone pool
NADPH
NADP+
ADP Pi
ATP
3.6.3.14
1.18.1.2
1.10.9.1
H2O 2H+ 1/2 O2
2×2H+
3H+
Photosystem II
D1 D2 cp43 cp47 cyt b559
PsbA PsbD PsbC PsbB PsbE PsbF
MSP OEC
PsbL PsbJ PsbK PsbM PsbH PsbI PsbO PsbP
PsbQ PsbR PsbS PsbT PsbU PsbV PsbW PsbX
PsbY PsbZ Psb27 Psb28 Psb28-2
Photosystem I
PsaA PsaB PsaC PsaD PsaE PsaF PsaG PsaH
PsaI PsaJ PsaK PsaL PsaM PsaN PsaO PsaX
Cytochrome b6/f complex
PetB PetD PetA PetC PetL PetM PetN PetG
Photosynthethic electron transport
PC Fd FNR cyt c6
PetE PetF PetH PetJ
F-type ATPase
beta alpha gamma delta epsilon c a b
00195 1/15/14
(c) Kanehisa Laboratories

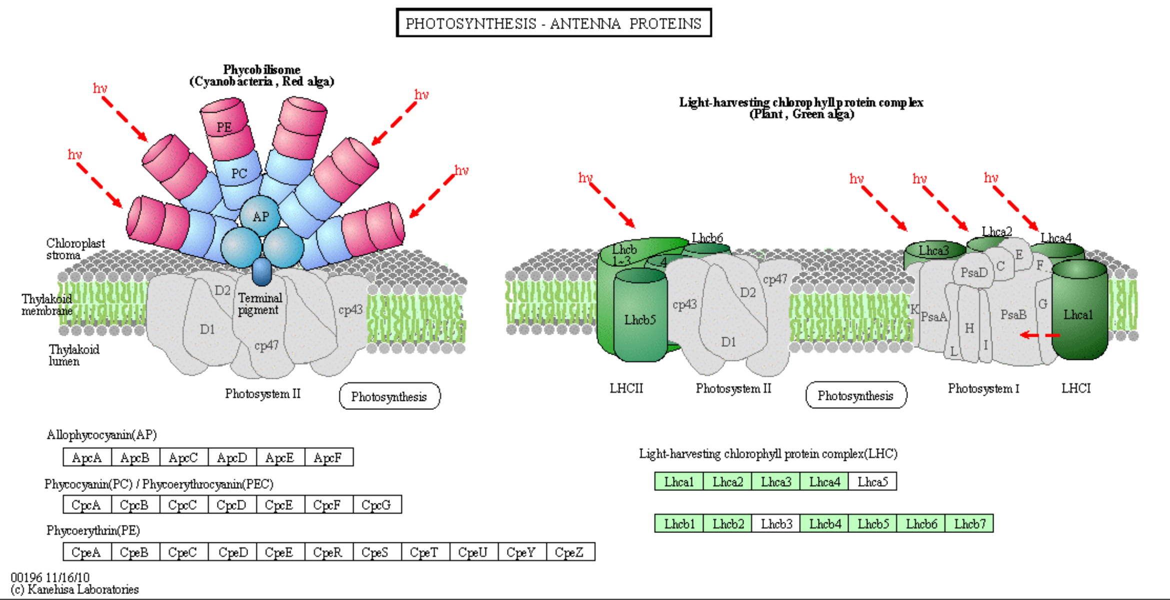
PHOTOSYNTHESIS - ANTENNA PROTEINS
Phycobilisome
(Cyanobacteria , Red alga)
hv
PE
PC
AP
Chloroplast stroma
Thylakoid membrane
Thylakoid lumen
D2
D1
Terminal pigment
cp43
cp47
Photosystem II
Photosynthesis
Light-harvesting chlorophyll protein complex
(Plant , Green alga)
Lhcb 1-3
Lhcb6
Lhcb4
Lhcb5
LHCII
Photosystem II
Photosynthesis
Lhca3
Lhca2
Lhca4
Lhca1
PsaD
PsaA
PsaB
Photosystem I
LHCI
Allophycocyanin(AP)
ApcA ApcB ApcC ApcD ApcE ApcF
Phycocyanin(PC) / Phycoerythrocyanin(PEC)
CpcA CpcB CpcC CpcD CpcE CpcF CpcG
Phycoerythrin(PE)
CpeA CpeB CpeC CpeD CpeE CpeR CpeS CpeT CpeU CpeY CpeZ
Light-harvesting chlorophyll protein complex(LHC)
Lhca1 Lhca2 Lhca3 Lhca4 Lhca5
Lhcb1 Lhcb2 Lhcb3 Lhcb4 Lhcb5 Lhcb6 Lhcb7
00196 11/16/10
(c) Kanehisa Laboratories

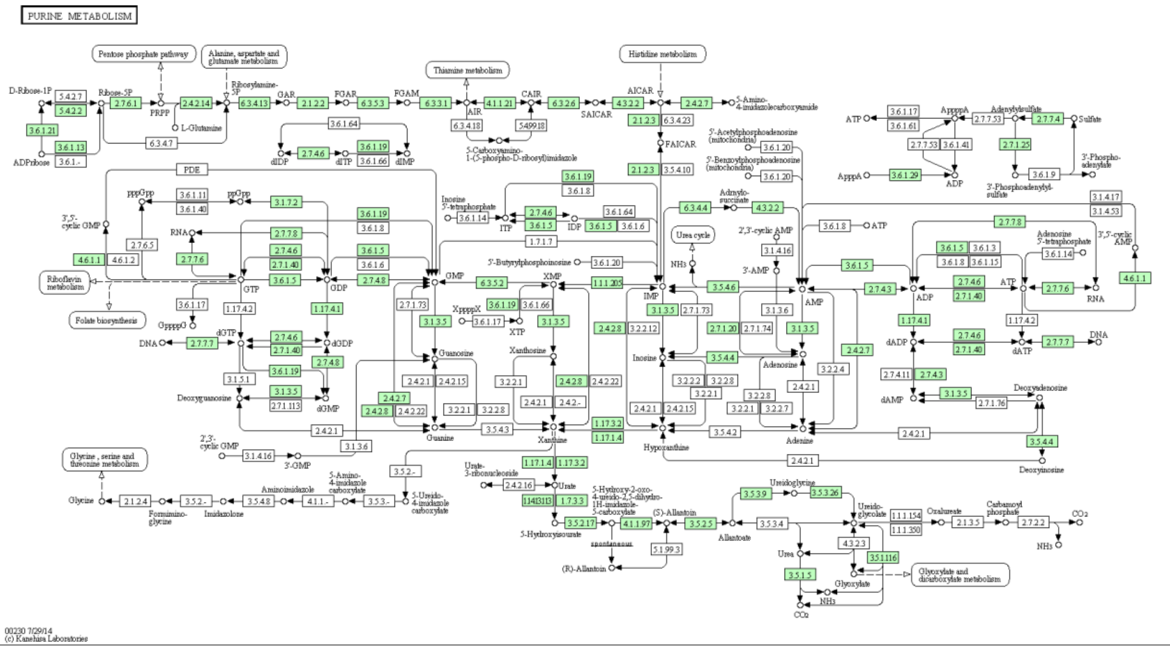
PURINE METABOLISM
Pentose phosphate pathway
Alanine, aspartate and glutamate metabolism
Thiamine metabolism
Histidine metabolism
D-Ribose-1P
Ribose-5P
PRPP
GAR
FGAR
FGAM
AIR
CAIR
SAICAR
AICAR
FAICAR
IMP
XMP
GMP
AMP
ADP
ATP
GDP
GTP
dGTP
dGDP
dGMP
Deoxyguanosine
Guanosine
Guanine
Xanthosine
Xanthine
Inosine
Hypoxanthine
Adenosine
Adenine
dADP
dAMP
dATP
Deoxyadenosine
Urate
(S)-Allantoin
Allantoate
Ureidoglycine
Ureidoglycolate
Urea
Glyoxylate
Glyoxylate and dicarboxylate metabolism
Urea cycle
Riboflavin metabolism
Folate biosynthesis
Glycine, serine and threonine metabolism
Glycine
Imidazolone
DNA
RNA
00230 7/29/14
(c) Kanehisa Laboratories

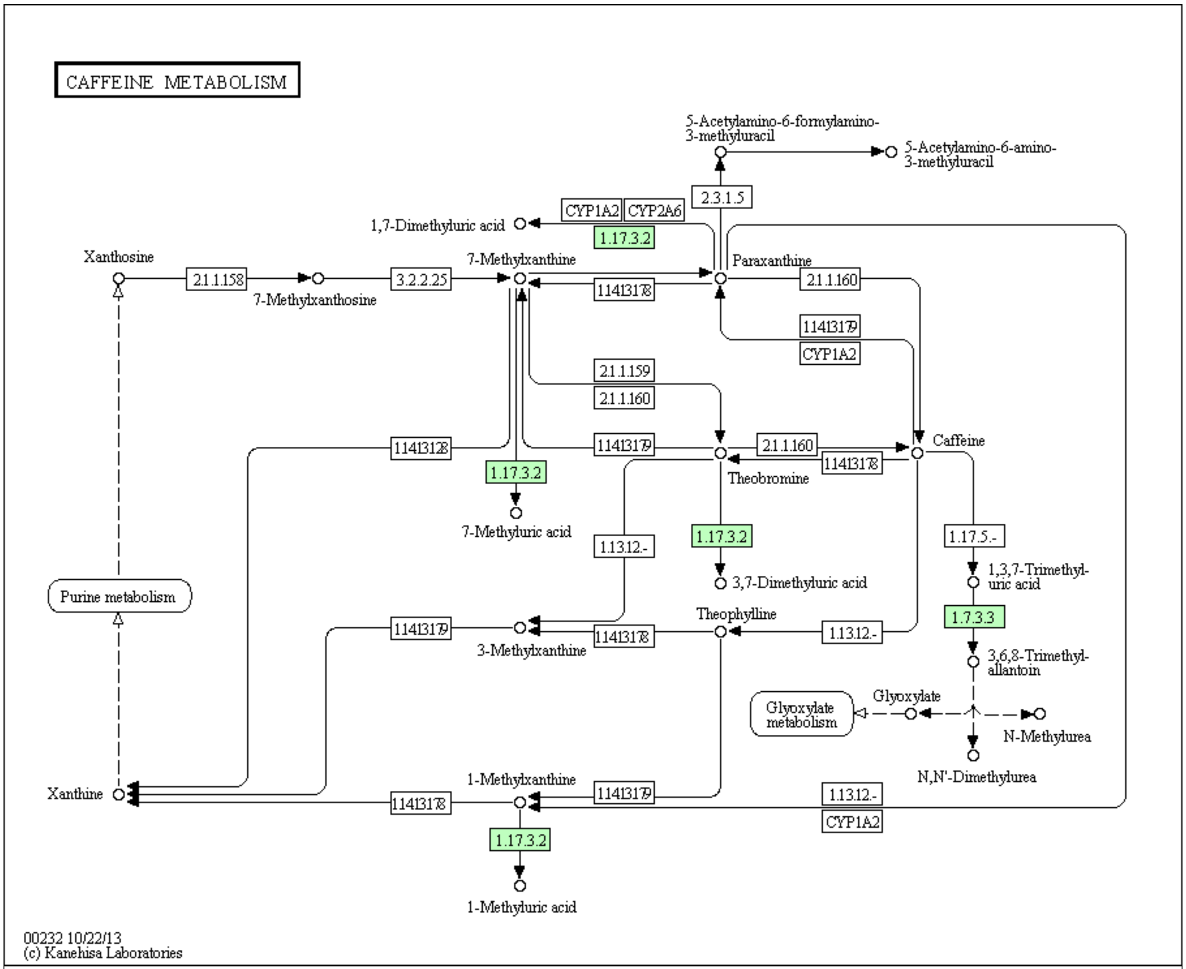
CAFFEINE METABOLISM
5-Acetylamino-6-formylamino-3-methyluracil
5-Acetylamino-6-amino-3-methyluracil
2.3.1.5
1,7-Dimethyluric acid
CYP1A2
CYP2A6
1.17.3.2
Xanthosine
2.1.1.158
7-Methylxanthosine
3.2.2.25
7-Methylxanthine
Paraxanthine
1.14.13.178
2.1.1.160
1.14.13.179
CYP1A2
2.1.1.159
2.1.1.160
1.14.13.128
1.14.13.179
2.1.1.160
Caffeine
1.14.13.178
Theobromine
1.17.3.2
7-Methyluric acid
1.14.13.-
1.17.3.2
1.17.5.-
1,3,7-Trimethyluric acid
3,7-Dimethyluric acid
Purine metabolism
1.7.3.3
1.14.13.179
Theophylline
3-Methylxanthine
1.14.13.178
1.13.12.-
3,6,8-Trimethylallantoin
Glyoxylate metabolism
Glyoxylate
N-Methylurea
N,N'-Dimethylurea
Xanthine
1-Methylxanthine
1.14.13.178
1.14.13.179
1.14.13.-
CYP1A2
1.17.3.2
1-Methyluric acid
00232 10/22/13
(c) Kanehisa Laboratories

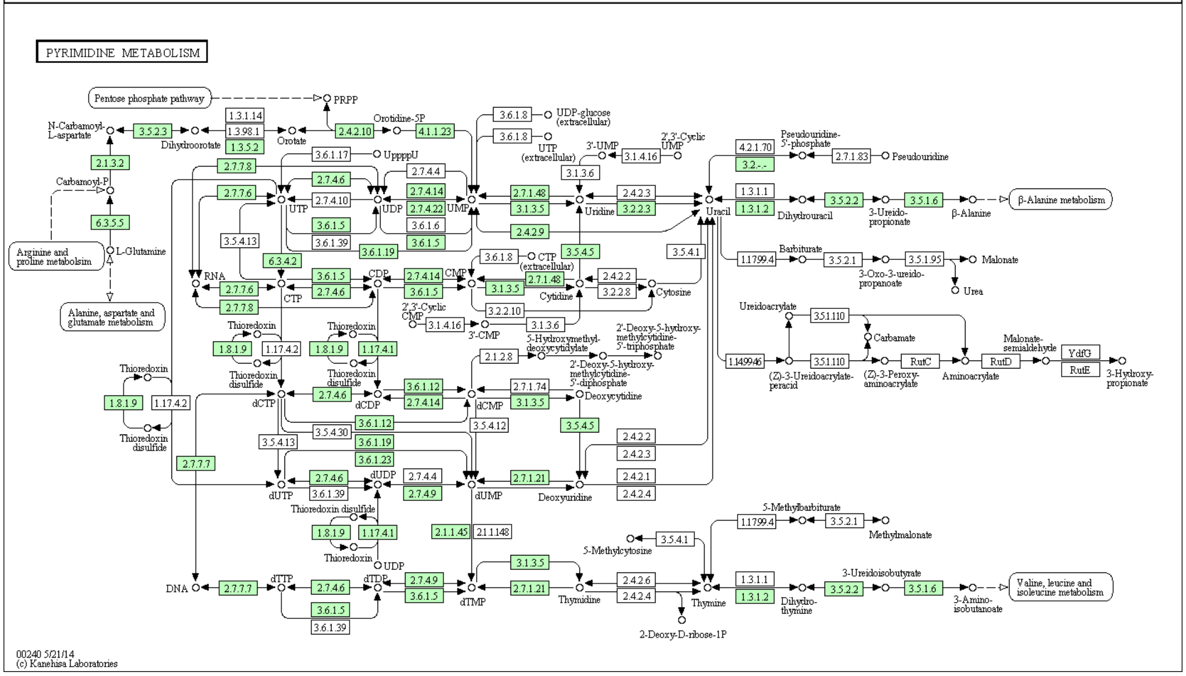
PYRIMIDINE METABOLISM
Pentose phosphate pathway
PRPP
N-Carbamoyl-L-aspartate
Dihydroorotate
Orotate
Orotidine-5P
UDP-glucose (extracellular)
UTP (extracellular)
3'-UMP
2',3'-Cyclic UMP
Pseudouridine-5'-phosphate
Pseudouridine
Carbamoyl-P
UppppU
UTP
UDP
UMP
Uridine
Uracil
Dihydrouracil
3-Ureido-propionate
β-Alanine
β-Alanine metabolism
L-Glutamine
Arginine and proline metabolism
Alanine, aspartate and glutamate metabolism
Barbiturate
3-Oxo-3-ureido-propanoate
Malonate
Urea
RNA
CTP
CDP
CMP
CTP (extracellular)
Cytidine
Cytosine
2',3'-Cyclic CMP
3'-CMP
Thioredoxin
Thioredoxin disulfide
Ureidoacrylate
Carbamate
Malonate-semialdehyde
3-Hydroxy-propionate
(Z)-3-Ureidoacrylate-peracid
(Z)-3-Peroxy-aminoacrylate
Aminoacrylate
RutC
RutD
YdfG
RutE
5-Hydroxymethyl-deoxycytidylate
2'-Deoxy-5-hydroxy-methylcytidine-5'-triphosphate
2'-Deoxy-5-hydroxy-methylcytidine-5'-diphosphate
dCTP
dCDP
dCMP
Deoxycytidine
dUTP
dUDP
dUMP
Deoxyuridine
5-Methylbarbiturate
Methylmalonate
5-Methylcytosine
3-Ureidoisobutyrate
DNA
dTTP
dTDP
dTMP
Thymidine
Thymine
Dihydro-thymine
3-Amino-isobutanoate
Valine, leucine and isoleucine metabolism
2-Deoxy-D-ribose-1P
00240 5/21/14
(c) Kanehisa Laboratories

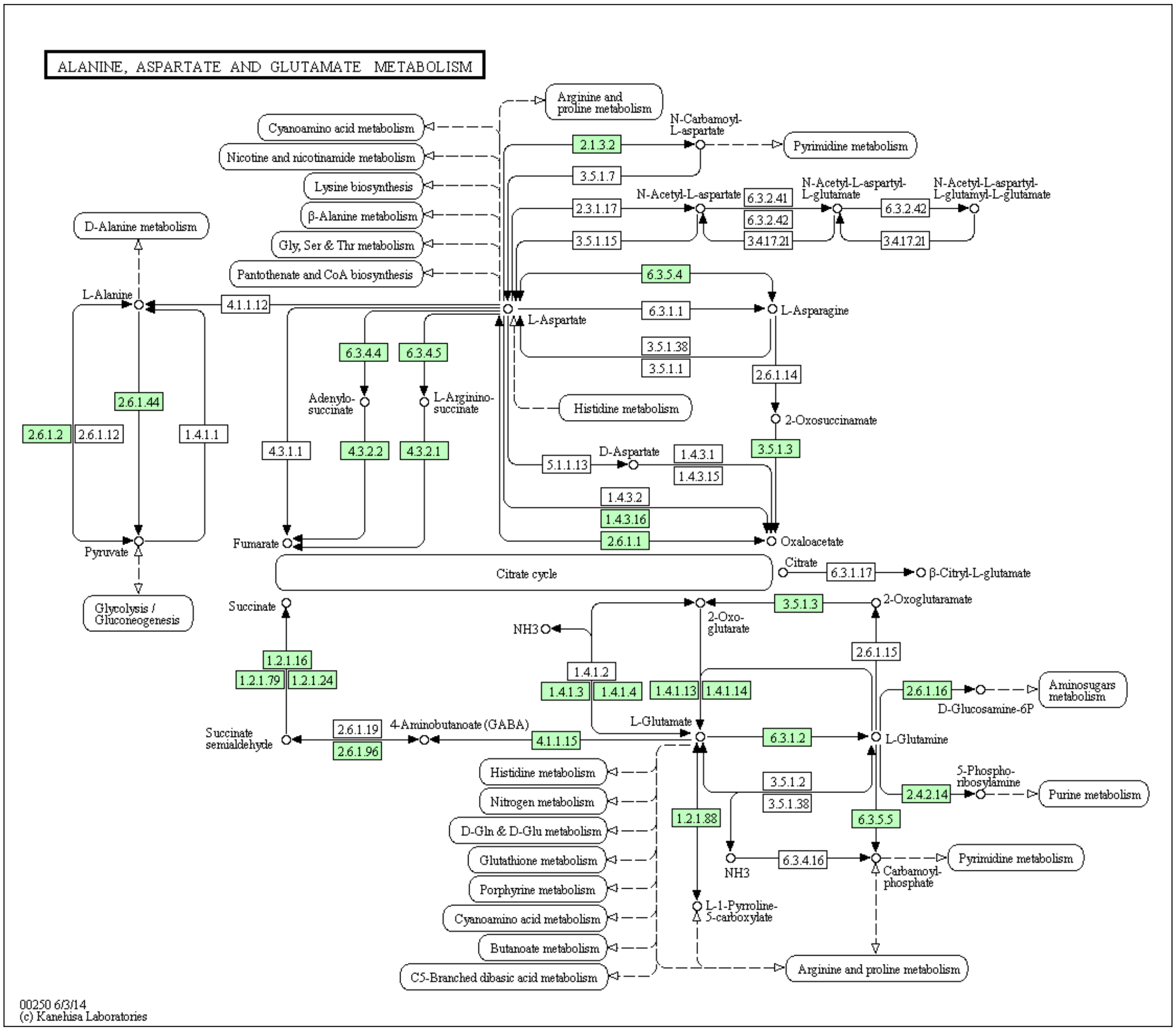

ALANINE, ASPARTATE AND GLUTAMATE METABOLISM
Arginine and proline metabolism
Cyanoamino acid metabolism
Nicotine and nicotinamide metabolism
Lysine biosynthesis
β-Alanine metabolism
Gly, Ser & Thr metabolism
Pantothenate and CoA biosynthesis
D-Alanine metabolism
N-Carbamoyl-L-aspartate
Pyrimidine metabolism
N-Acetyl-L-aspartate
N-Acetyl-L-aspartyl-L-glutamate
N-Acetyl-L-aspartyl-L-glutamyl-L-glutamate
L-Alanine
L-Aspartate
L-Asparagine
Adenylo-succinate
L-Arginino-succinate
Histidine metabolism
2-Oxosuccinamate
D-Aspartate
Fumarate
Oxaloacetate
Pyruvate
Citrate cycle
Citrate
β-Citryl-L-glutamate
Glycolysis / Gluconeogenesis
Succinate
2-Oxoglutaramate
NH3
2-Oxo-glutarate
Aminosugars metabolism
D-Glucosamine-6P
Succinate semialdehyde
4-Aminobutanoate (GABA)
L-Glutamate
L-Glutamine
5-Phospho-ribosylamine
Purine metabolism
Histidine metabolism
Nitrogen metabolism
D-Gln & D-Glu metabolism
Glutathione metabolism
Porphyrine metabolism
Cyanoamino acid metabolism
Butanoate metabolism
C5-Branched dibasic acid metabolism
Carbamoyl-phosphate
L-1-Pyrroline-5-carboxylate
Arginine and proline metabolism
2.1.3.2
3.5.1.7
2.3.1.17
3.5.1.15
6.3.2.41
6.3.2.42
3.4.17.21
6.3.5.4
4.1.1.12
6.3.1.1
3.5.1.38
3.5.1.1
2.6.1.14
6.3.4.4
6.3.4.5
2.6.1.44
2.6.1.2
2.6.1.12
1.4.1.1
4.3.1.1
4.3.2.2
4.3.2.1
3.5.1.3
5.1.1.13
1.4.3.1
1.4.3.15
1.4.3.2
1.4.3.16
2.6.1.1
6.3.1.17
1.2.1.16
1.2.1.79
1.2.1.24
1.4.1.2
1.4.1.3
1.4.1.4
1.4.1.13
1.4.1.14
2.6.1.15
2.6.1.16
2.6.1.19
2.6.1.96
4.1.1.15
6.3.1.2
3.5.1.2
2.4.2.14
1.2.1.88
6.3.5.5
6.3.4.16
00250 6/3/14
(c) Kanehisa Laboratories

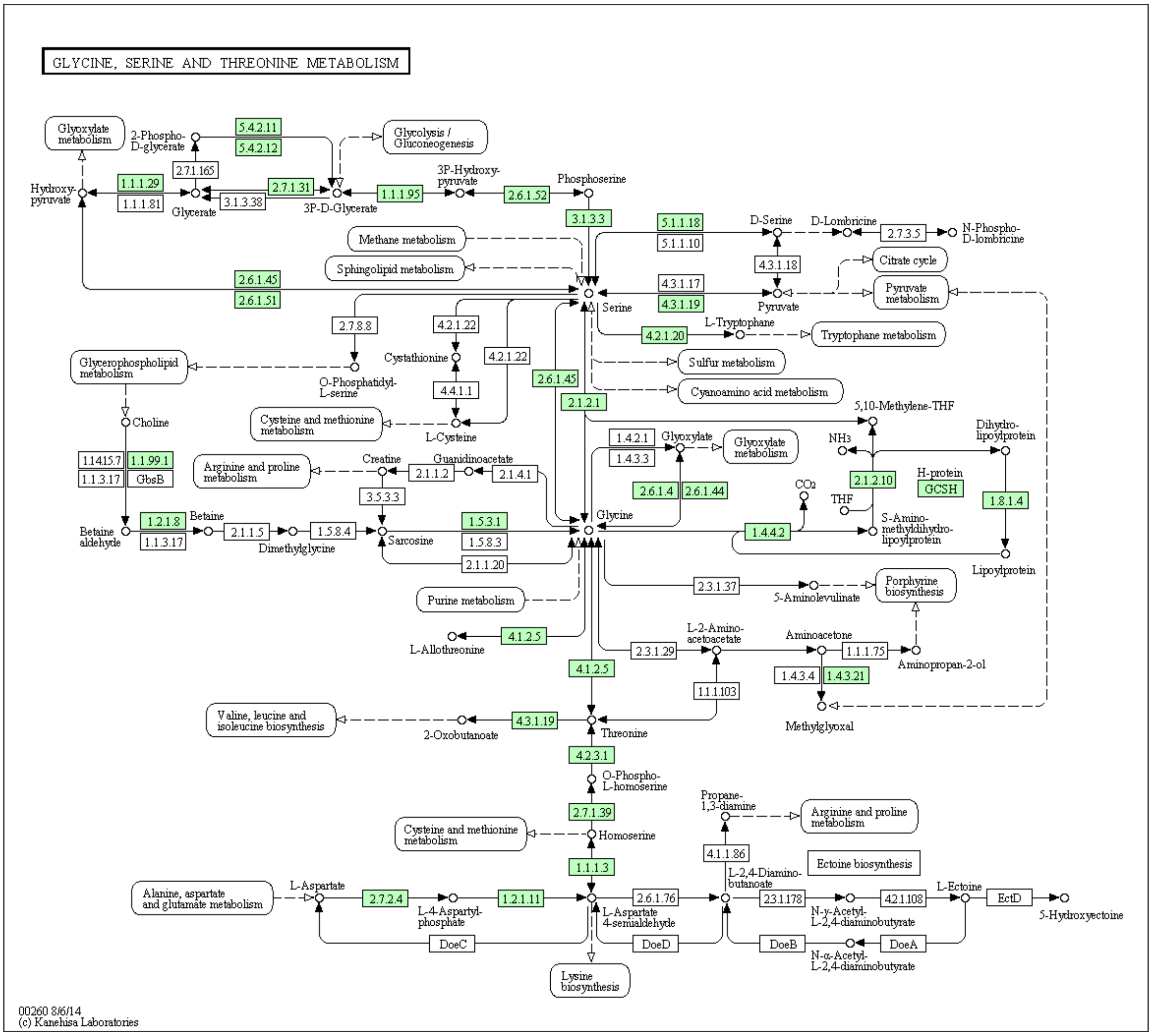

GLYCINE, SERINE AND THREONINE METABOLISM
Glyoxylate metabolism
2-Phospho-D-glycerate
5.4.2.11
5.4.2.12
Glycolysis / Gluconeogenesis
2.7.1.165
1.1.1.29
2.7.1.31
1.1.1.95
3P-Hydroxy-pyruvate
2.6.1.52
Phosphoserine
Hydroxy-pyruvate
1.1.1.81
Glycerate
3.1.3.38
3P-D-Glycerate
3.1.3.3
5.1.1.18
5.1.1.10
D-Serine
D-Lombricine
2.7.3.5
N-Phospho-D-lombricine
Methane metabolism
Sphingolipid metabolism
4.3.1.18
Citrate cycle
2.6.1.45
2.6.1.51
4.3.1.17
4.3.1.19
Pyruvate
Pyruvate metabolism
Serine
2.7.8.8
4.2.1.22
4.2.1.22
4.2.1.20
L-Tryptophane
Tryptophane metabolism
Glycerophospholipid metabolism
Cystathionine
O-Phosphatidyl-L-serine
Sulfur metabolism
2.6.1.45
4.4.1.1
Cyanoamino acid metabolism
2.1.2.1
Choline
Cysteine and methionine metabolism
L-Cysteine
5,10-Methylene-THF
Dihydro-lipoylprotein
1.4.2.1
Glyoxylate
Glyoxylate metabolism
NH3
1.1.4.15.7
1.1.99.1
1.1.3.17
GbsB
Arginine and proline metabolism
Creatine
Guanidinoacetate
2.1.1.2
2.1.4.1
1.4.3.3
2.1.2.10
H-protein
GCSH
3.5.3.3
2.6.1.4
2.6.1.44
CO2
THF
1.8.1.4
Betaine
1.2.1.8
1.5.3.1
Glycine
S-Amino-methyldihydro-lipoylprotein
Betaine aldehyde
1.1.3.17
2.1.1.5
1.5.8.4
Sarcosine
1.5.8.3
1.4.4.2
Dimethylglycine
2.1.1.20
Lipoylprotein
2.3.1.37
5-Aminolevulinate
Porphyrine biosynthesis
Purine metabolism
L-Allothreonine
4.1.2.5
L-2-Amino-acetoacetate
Aminoacetone
2.3.1.29
1.1.1.75
Aminopropan-2-ol
4.1.2.5
1.4.3.4
1.4.3.21
1.1.1.103
Valine, leucine and isoleucine biosynthesis
2-Oxobutanoate
4.3.1.19
Threonine
Methylglyoxal
4.2.3.1
O-Phospho-L-homoserine
Propane-1,3-diamine
Arginine and proline metabolism
2.7.1.39
Cysteine and methionine metabolism
Homoserine
4.1.1.86
Ectoine biosynthesis
1.1.1.3
L-2,4-Diamino-butanoate
Alanine, aspartate and glutamate metabolism
L-Aspartate
2.7.2.4
L-4-Aspartyl-phosphate
1.2.1.11
L-Aspartate 4-semialdehyde
2.6.1.76
2.3.1.178
N-γ-Acetyl-L-2,4-diaminobutyrate
4.2.1.108
L-Ectoine
EctD
5-Hydroxyectoine
DoeC
DoeD
DoeB
N-α-Acetyl-L-2,4-diaminobutyrate
DoeA
Lysine biosynthesis
00260 8/6/14
(c) Kanehisa Laboratories

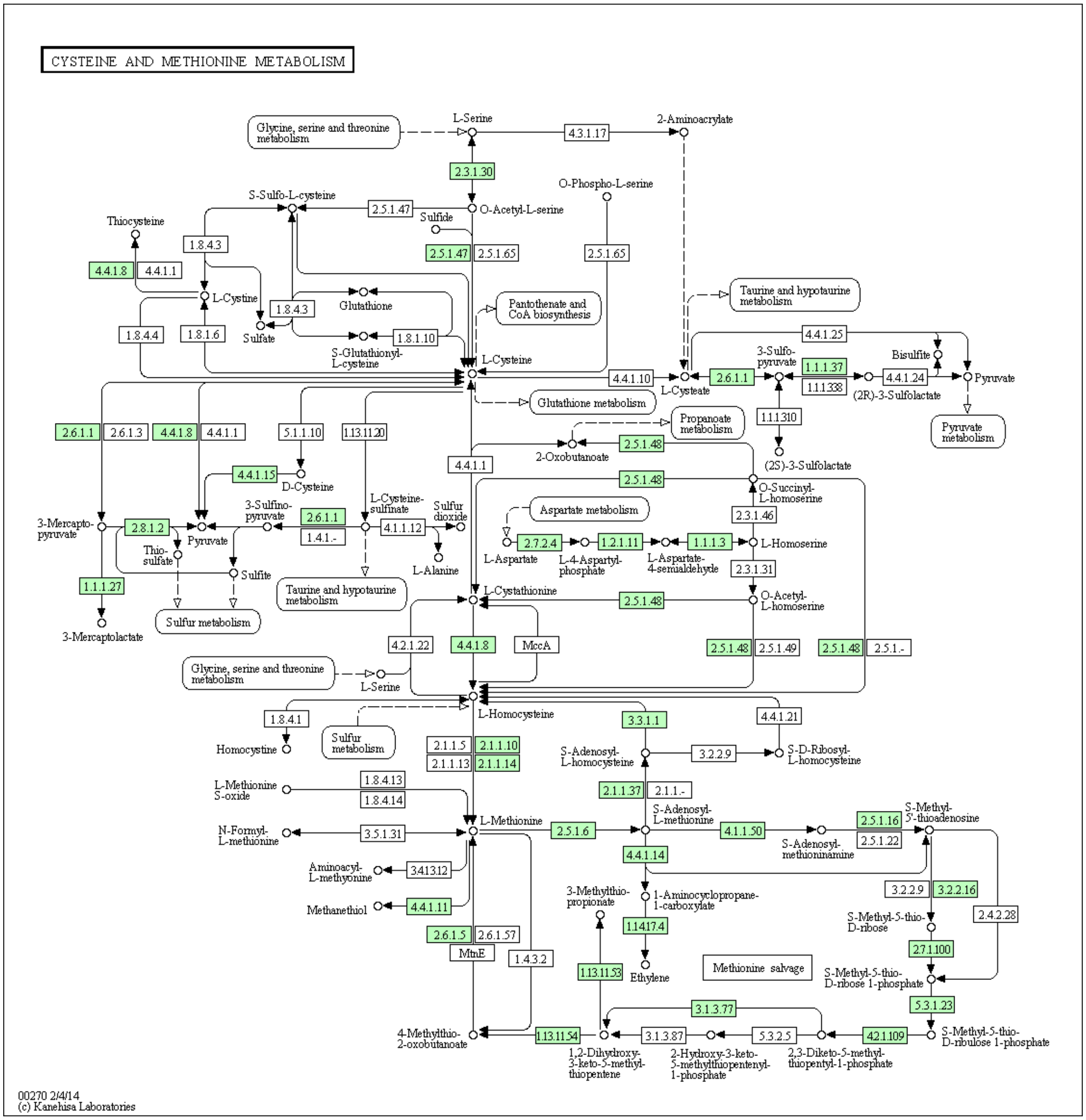

CYSTEINE AND METHIONINE METABOLISM
Glycine, serine and threonine metabolism
L-Serine
4.3.1.17
2-Aminoacrylate
2.3.1.30
O-Phospho-L-serine
S-Sulfo-L-cysteine
2.5.1.47
Sulfide
O-Acetyl-L-serine
Thiocysteine
1.8.4.3
2.5.1.47
2.5.1.65
2.5.1.65
4.4.1.8
4.4.1.1
L-Cystine
Glutathione
Pantothenate and CoA biosynthesis
Taurine and hypotaurine metabolism
1.8.4.3
Sulfate
1.8.4.4
1.8.1.6
S-Glutathionyl-L-cysteine
1.8.1.10
L-Cysteine
4.4.1.25
3-Sulfo-pyruvate
Bisulfite
1.1.1.37
4.4.1.10
L-Cysteate
2.6.1.1
1.1.1.338
4.4.1.24
Pyruvate
(2R)-3-Sulfolactate
Glutathione metabolism
Propanoate metabolism
1.1.1.310
Pyruvate metabolism
2.6.1.1
2.6.1.3
4.4.1.8
4.4.1.1
5.1.1.10
1.13.11.20
2-Oxobutanoate
2.5.1.48
(2S)-3-Sulfolactate
4.4.1.1
4.4.1.15
D-Cysteine
2.5.1.48
O-Succinyl-L-homoserine
3-Sulfino-pyruvate
L-Cysteine-sulfinate
Sulfur dioxide
Aspartate metabolism
2.3.1.46
3-Mercapto-pyruvate
2.8.1.2
Pyruvate
2.6.1.1
1.4.1.-
4.1.1.12
2.7.2.4
1.2.1.11
1.1.1.3
L-Homoserine
Thio-sulfate
Sulfite
L-Alanine
L-Aspartate
L-4-Aspartyl-phosphate
L-Aspartate-4-semialdehyde
2.3.1.31
1.1.1.27
Taurine and hypotaurine metabolism
L-Cystathionine
2.5.1.48
O-Acetyl-L-homoserine
3-Mercaptolactate
Sulfur metabolism
4.2.1.22
4.4.1.8
MccA
2.5.1.48
2.5.1.49
2.5.1.48
2.5.1.-
Glycine, serine and threonine metabolism
L-Serine
L-Homocysteine
1.8.4.1
3.3.1.1
4.4.1.21
Homocystine
Sulfur metabolism
2.1.1.5
2.1.1.10
2.1.1.13
2.1.1.14
S-Adenosyl-L-homocysteine
3.2.2.9
S-D-Ribosyl-L-homocysteine
L-Methionine S-oxide
1.8.4.13
1.8.4.14
2.1.1.37
2.1.1.-
S-Adenosyl-L-methionine
S-Methyl-5'-thioadenosine
N-Formyl-L-methionine
3.5.1.31
L-Methionine
2.5.1.6
4.1.1.50
2.5.1.16
S-Adenosyl-methioninamine
2.5.1.22
4.4.1.14
Aminoacyl-L-methionine
3.4.13.12
3-Methylthio-propionate
1-Aminocyclopropane-1-carboxylate
3.2.2.9
3.2.2.16
Methanethiol
4.4.1.11
S-Methyl-5-thio-D-ribose
2.4.2.28
2.6.1.5
2.6.1.57
1.14.17.4
2.7.1.100
MtnE
1.4.3.2
1.13.11.53
Ethylene
Methionine salvage
S-Methyl-5-thio-D-ribose 1-phosphate
5.3.1.23
3.1.3.77
4-Methylthio-2-oxobutanoate
1.13.11.54
3.1.3.87
5.3.2.5
4.2.1.109
S-Methyl-5-thio-D-ribulose 1-phosphate
1,2-Dihydroxy-3-keto-5-methyl-thiopentene
2-Hydroxy-3-keto-5-methylthiopentenyl-1-phosphate
2,3-Diketo-5-methyl-thiopentyl-1-phosphate
00270 2/4/14
(c) Kanehisa Laboratories

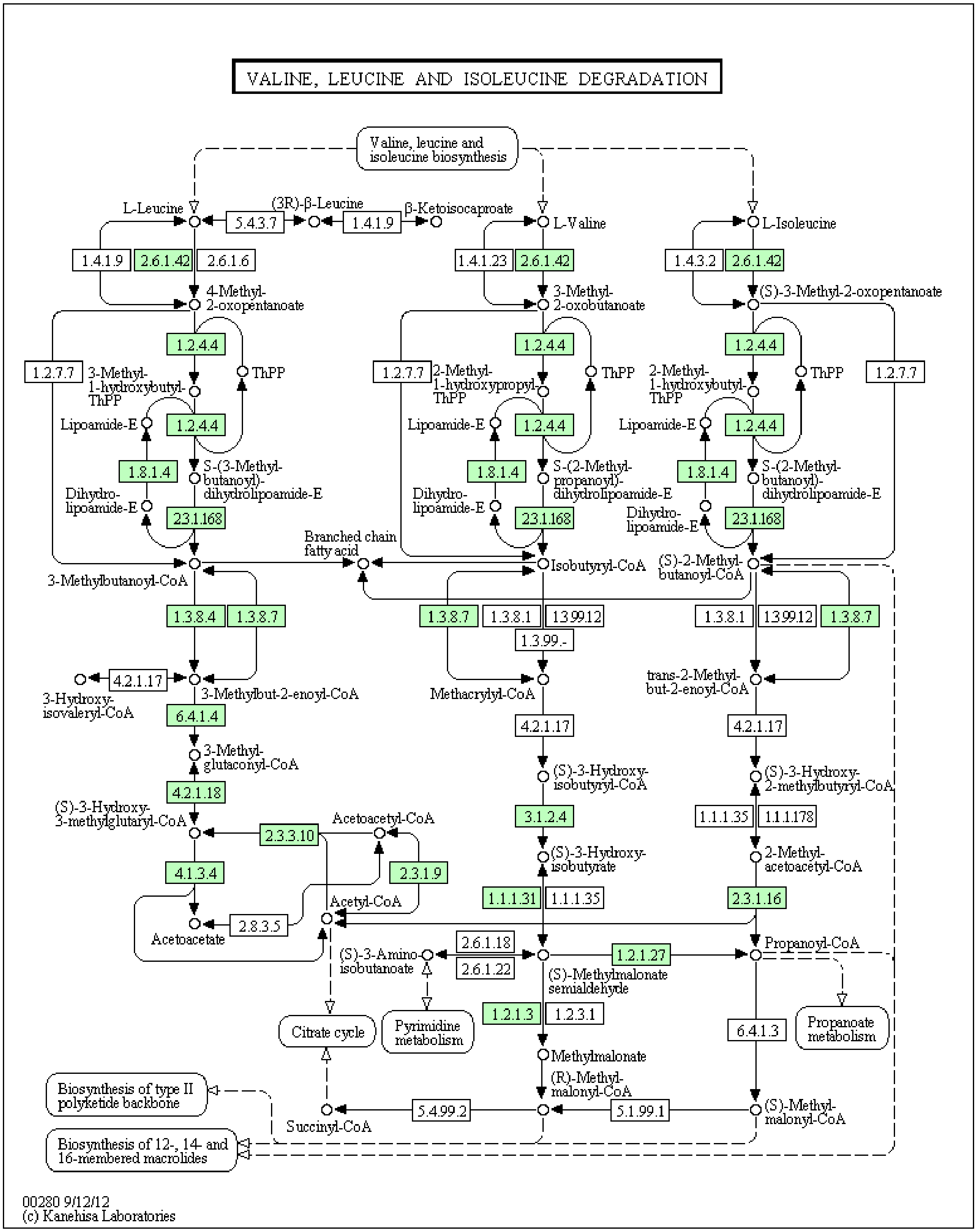

VALINE, LEUCINE AND ISOLEUCINE DEGRADATION
Valine, leucine and isoleucine biosynthesis
L-Leucine
(3R)-β-Leucine
β-Ketoisocaproate
5.4.3.7
1.4.1.9
L-Valine
L-Isoleucine
1.4.1.9
2.6.1.42
2.6.1.6
1.4.1.23
2.6.1.42
1.4.3.2
2.6.1.42
4-Methyl-
2-oxopentanoate
3-Methyl-
2-oxobutanoate
(S)-3-Methyl-2-oxopentanoate
1.2.4.4
1.2.4.4
1.2.4.4
1.2.7.7
3-Methyl-
1-hydroxybutyl-
ThPP
ThPP
1.2.7.7
2-Methyl-
1-hydroxypropyl-
ThPP
ThPP
2-Methyl-
1-hydroxybutyl-
ThPP
ThPP
1.2.7.7
Lipoamide-E
1.2.4.4
Lipoamide-E
1.2.4.4
Lipoamide-E
1.2.4.4
1.8.1.4
S-(3-Methyl-
butanoyl)-
dihydrolipoamide-E
1.8.1.4
S-(2-Methyl-
propanoyl)-
dihydrolipoamide-E
1.8.1.4
S-(2-Methyl-
butanoyl)-
dihydrolipoamide-E
Dihydro-
lipoamide-E
2.3.1.168
Dihydro-
lipoamide-E
2.3.1.168
Dihydro-
lipoamide-E
2.3.1.168
Branched chain
fatty acid
3-Methylbutanoyl-CoA
Isobutyryl-CoA
(S)-2-Methyl-
butanoyl-CoA
1.3.8.4
1.3.8.7
1.3.8.7
1.3.8.1
1.3.99.12
1.3.99.-
1.3.8.1
1.3.99.12
1.3.8.7
4.2.1.17
3-Hydroxy-
isovaleryl-CoA
3-Methylbut-2-enoyl-CoA
Methacrylyl-CoA
trans-2-Methyl-
but-2-enoyl-CoA
6.4.1.4
4.2.1.17
4.2.1.17
3-Methyl-
glutaconyl-CoA
(S)-3-Hydroxy-
isobutyryl-CoA
(S)-3-Hydroxy-
2-methylbutyryl-CoA
4.2.1.18
(S)-3-Hydroxy-
3-methylglutaryl-CoA
2.3.3.10
Acetoacetyl-CoA
3.1.2.4
1.1.1.35
1.1.1.178
4.1.3.4
2.3.1.9
(S)-3-Hydroxy-
isobutyrate
2-Methyl-
acetoacetyl-CoA
Acetyl-CoA
1.1.1.31
1.1.1.35
2.3.1.16
Acetoacetate
2.8.3.5
(S)-3-Amino-
isobutanoate
2.6.1.18
2.6.1.22
1.2.1.27
Propanoyl-CoA
(S)-Methylmalonate
semialdehyde
Citrate cycle
Pyrimidine
metabolism
1.2.1.3
1.2.3.1
6.4.1.3
Propanoate
metabolism
Methylmalonate
Biosynthesis of type II
polyketide backbone
(R)-Methyl-
malonyl-CoA
5.4.99.2
5.1.99.1
(S)-Methyl-
malonyl-CoA
Succinyl-CoA
Biosynthesis of 12-, 14- and
16-membered macrolides
00280 9/12/12
(c) Kanehisa Laboratories

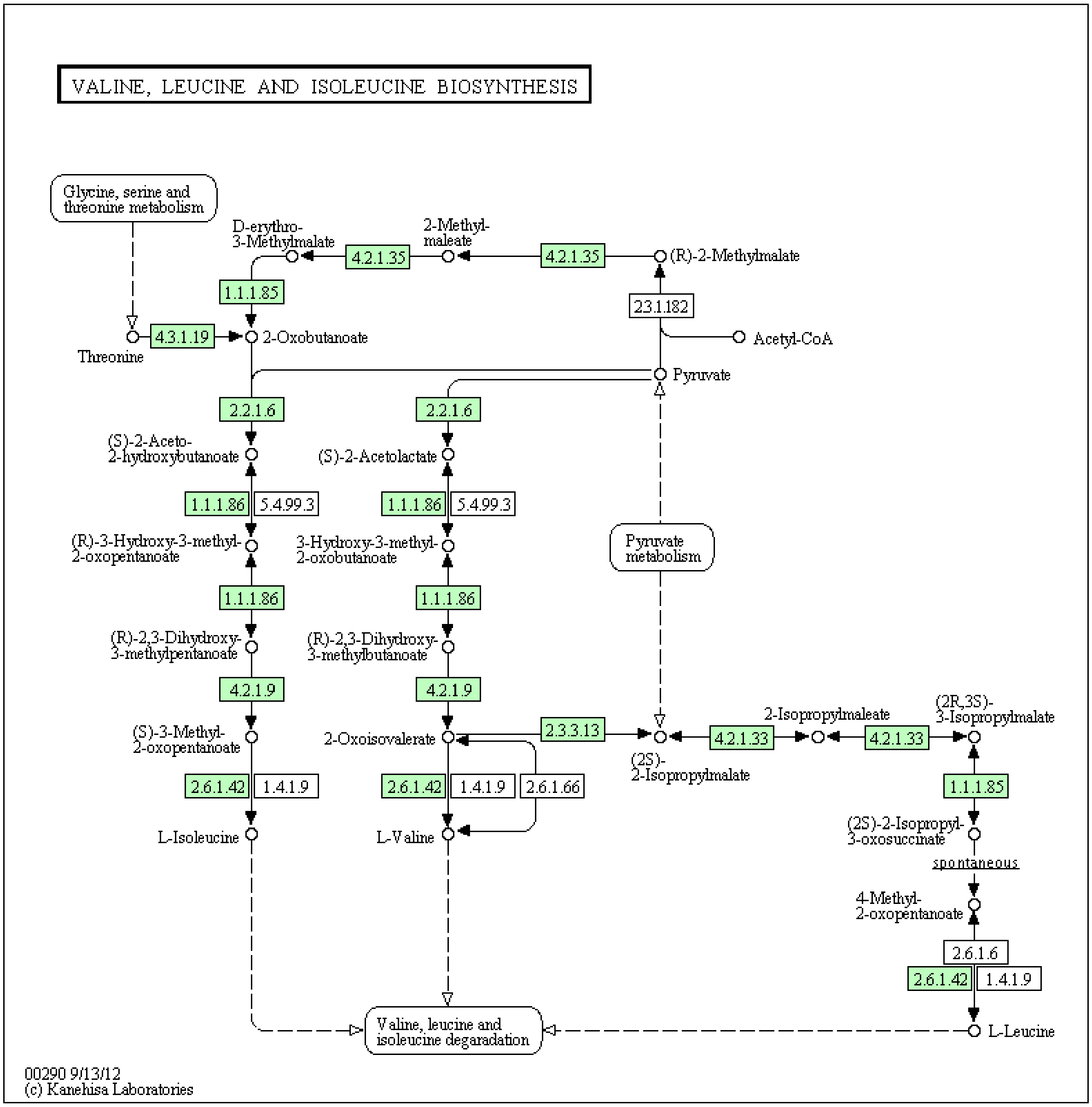
VALINE, LEUCINE AND ISOLEUCINE BIOSYNTHESIS
Glycine, serine and threonine metabolism
D-erythro-3-Methylmalate
2-Methyl-maleate
4.2.1.35
4.2.1.35
(R)-2-Methylmalate
1.1.1.85
2.3.1.182
Threonine
4.3.1.19
2-Oxobutanoate
Acetyl-CoA
Pyruvate
2.2.1.6
2.2.1.6
(S)-2-Aceto-2-hydroxybutanoate
(S)-2-Acetolactate
1.1.1.86
5.4.99.3
1.1.1.86
5.4.99.3
(R)-3-Hydroxy-3-methyl-2-oxopentanoate
3-Hydroxy-3-methyl-2-oxobutanoate
Pyruvate metabolism
1.1.1.86
1.1.1.86
(R)-2,3-Dihydroxy-3-methylpentanoate
(R)-2,3-Dihydroxy-3-methylbutanoate
4.2.1.9
4.2.1.9
(S)-3-Methyl-2-oxopentanoate
2-Oxoisovalerate
2.3.3.13
(2S)-2-Isopropylmalate
4.2.1.33
2-Isopropylmaleate
4.2.1.33
(2R,3S)-3-Isopropylmalate
2.6.1.42
1.4.1.9
2.6.1.42
1.4.1.9
2.6.1.66
1.1.1.85
L-Isoleucine
L-Valine
(2S)-2-Isopropyl-3-oxosuccinate
spontaneous
4-Methyl-2-oxopentanoate
2.6.1.6
2.6.1.42
1.4.1.9
Valine, leucine and isoleucine degaradation
L-Leucine
00290 9/13/12
(c) Kanehisa Laboratories

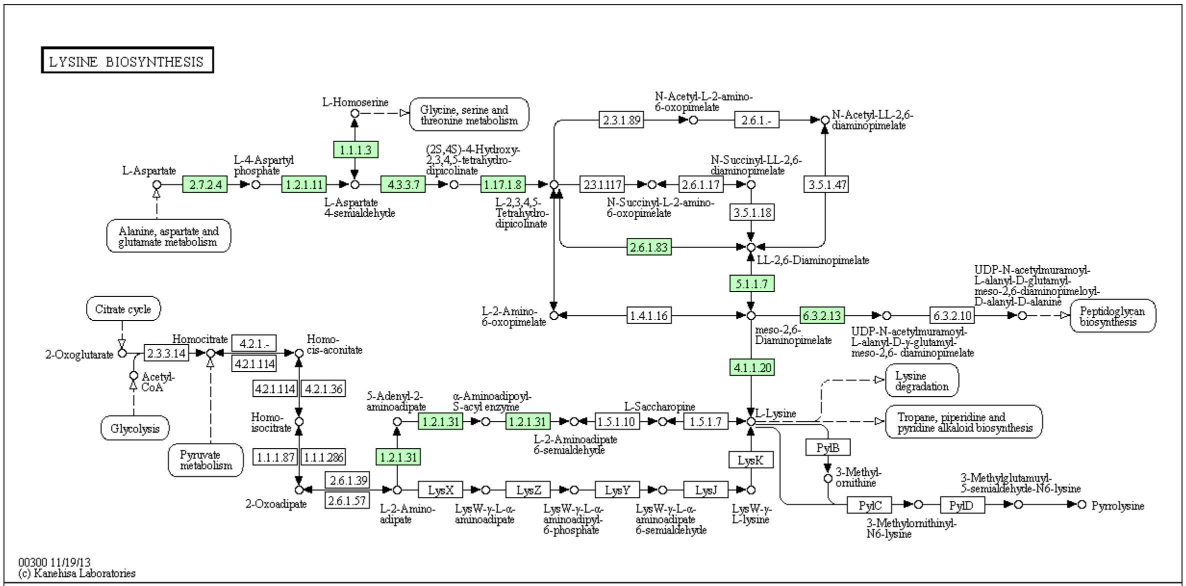
LYSINE BIOSYNTHESIS
L-Homoserine
Glycine, serine and threonine metabolism
1.1.1.3
L-Aspartate
2.7.2.4
L-4-Aspartyl phosphate
1.2.1.11
L-Aspartate 4-semialdehyde
4.3.3.7
(2S,4S)-4-Hydroxy-2,3,4,5-tetrahydro-dipicolinate
1.17.1.8
L-2,3,4,5-Tetrahydro-dipicolinate
Alanine, aspartate and glutamate metabolism
2.3.1.89
N-Acetyl-L-2-amino-6-oxopimelate
2.6.1.-
N-Acetyl-LL-2,6-diaminopimelate
2.3.1.117
N-Succinyl-L-2-amino-6-oxopimelate
2.6.1.17
N-Succinyl-LL-2,6-diaminopimelate
3.5.1.18
3.5.1.47
2.6.1.83
LL-2,6-Diaminopimelate
5.1.1.7
L-2-Amino-6-oxopimelate
1.4.1.16
meso-2,6-Diaminopimelate
6.3.2.13
UDP-N-acetylmuramoyl-L-alanyl-D-γ-glutamyl-meso-2,6-diaminopimelate
6.3.2.10
UDP-N-acetylmuramoyl-L-alanyl-D-glutamyl-meso-2,6-diaminopimeloyl-D-alanyl-D-alanine
Peptidoglycan biosynthesis
4.1.1.20
Citrate cycle
2-Oxoglutarate
2.3.3.14
Homocitrate
4.2.1.-
4.2.1.114
Homo-cis-aconitate
Acetyl-CoA
Glycolysis
Pyruvate metabolism
4.2.1.114
4.2.1.36
Homo-isocitrate
1.1.1.87
1.1.1.286
2.6.1.39
2.6.1.57
2-Oxoadipate
5-Adenyl-2-aminoadipate
1.2.1.31
α-Aminoadipoyl-S-acyl enzyme
1.2.1.31
1.2.1.31
L-2-Aminoadipate 6-semialdehyde
1.5.1.10
L-Saccharopine
1.5.1.7
L-Lysine
Lysine degradation
Tropane, piperidine and pyridine alkaloid biosynthesis
LysK
PylB
3-Methyl-ornithine
3-Methylglutamuyl-5-semialdehyde-N6-lysine
PylC
PylD
Pyrrolysine
3-Methylornithinyl-N6-lysine
L-2-Amino-adipate
LysX
LysW-γ-L-α-aminoadipate
LysZ
LysW-γ-L-α-aminoadipyl-6-phosphate
LysY
LysW-γ-L-α-aminoadipate 6-semialdehyde
LysJ
LysW-γ-L-lysine
00300 11/19/13
(c) Kanehisa Laboratories

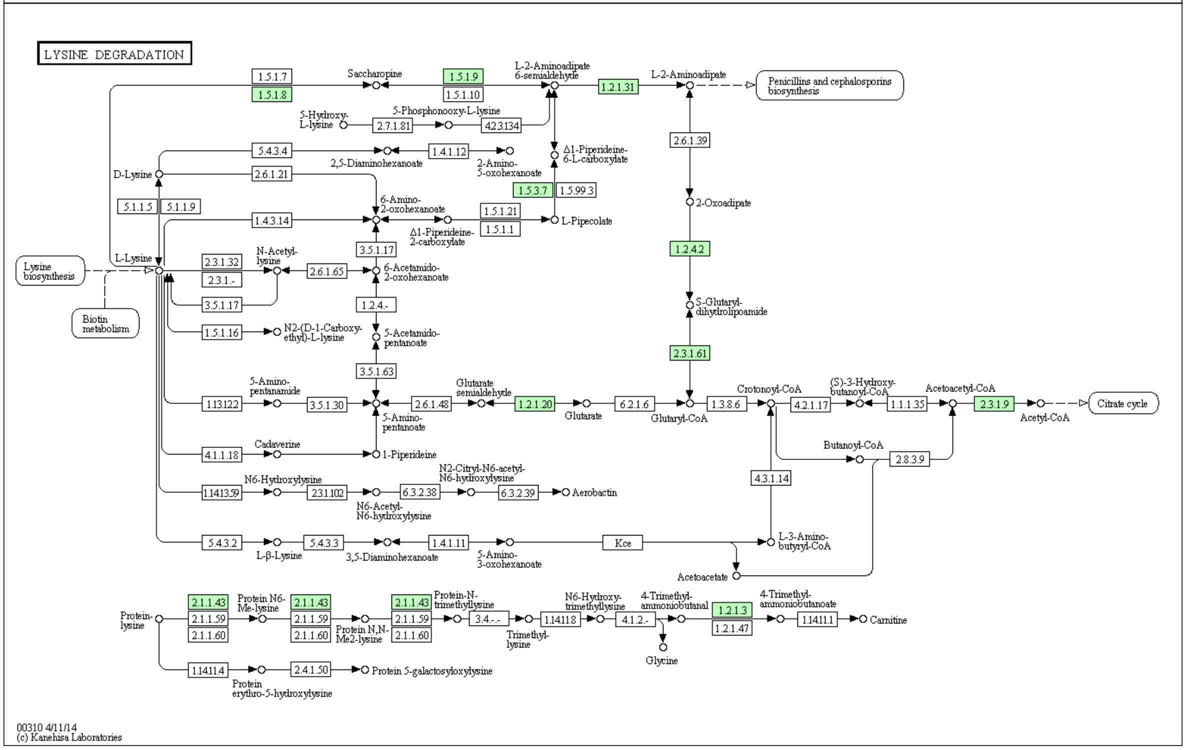
LYSINE DEGRADATION
1.5.1.7
1.5.1.8
Saccharopine
1.5.1.9
1.5.1.10
L-2-Aminoadipate 6-semialdehyde
1.2.1.31
L-2-Aminoadipate
Penicillins and cephalosporins biosynthesis
5-Hydroxy-L-lysine
2.7.1.81
5-Phosphonooxy-L-lysine
4.2.3.134
Δ1-Piperideine-6-L-carboxylate
5.4.3.4
2,5-Diaminohexanoate
1.4.1.12
2-Amino-5-oxohexanoate
D-Lysine
2.6.1.21
1.5.3.7
1.5.99.3
2.6.1.39
2-Oxoadipate
5.1.1.5
5.1.1.9
6-Amino-2-oxohexanoate
1.5.1.21
1.5.1.1
L-Pipecolate
1.4.3.14
Δ1-Piperideine-2-carboxylate
3.5.1.17
1.2.4.2
Lysine biosynthesis
L-Lysine
2.3.1.32
2.3.1.-
N-Acetyl-lysine
2.6.1.65
6-Acetamido-2-oxohexanoate
Biotin metabolism
3.5.1.17
1.2.4.-
S-Glutaryl-dihydrolipoamide
1.5.1.16
N2-(D-1-Carboxy-ethyl)-L-lysine
5-Acetamido-pentanoate
2.3.1.61
3.5.1.63
5-Amino-pentanamide
1.13.12.2
3.5.1.30
5-Amino-pentanoate
2.6.1.48
Glutarate semialdehyde
1.2.1.20
Glutarate
6.2.1.6
Glutaryl-CoA
1.3.8.6
Crotonoyl-CoA
4.2.1.17
(S)-3-Hydroxy-butanoyl-CoA
1.1.1.35
Acetoacetyl-CoA
2.3.1.9
Acetyl-CoA
Citrate cycle
4.1.1.18
Cadaverine
1-Piperideine
Butanoyl-CoA
2.8.3.9
4.3.1.14
1.14.13.59
N6-Hydroxylysine
2.3.1.102
N6-Acetyl-N6-hydroxylysine
6.3.2.38
N2-Citryl-N6-acetyl-N6-hydroxylysine
6.3.2.39
Aerobactin
5.4.3.2
L-β-Lysine
5.4.3.3
3,5-Diaminohexanoate
1.4.1.11
5-Amino-3-oxohexanoate
Kce
L-3-Amino-butyryl-CoA
Acetoacetate
Protein-lysine
2.1.1.43
2.1.1.59
2.1.1.60
Protein N6-Me-lysine
2.1.1.43
2.1.1.59
2.1.1.60
Protein N,N-Me2-lysine
2.1.1.43
2.1.1.59
2.1.1.60
Protein-N-trimethyllysine
3.4.-.-
Trimethyl-lysine
1.14.11.8
N6-Hydroxy-trimethyllysine
4.1.2.-
Glycine
4-Trimethyl-ammoniobutanal
1.2.1.3
1.2.1.47
4-Trimethyl-ammoniobutanoate
1.14.11.1
Carnitine
1.14.11.4
Protein erythro-5-hydroxylysine
2.4.1.50
Protein 5-galactosyloxylysine
00310 4/11/14
(c) Kanehisa Laboratories

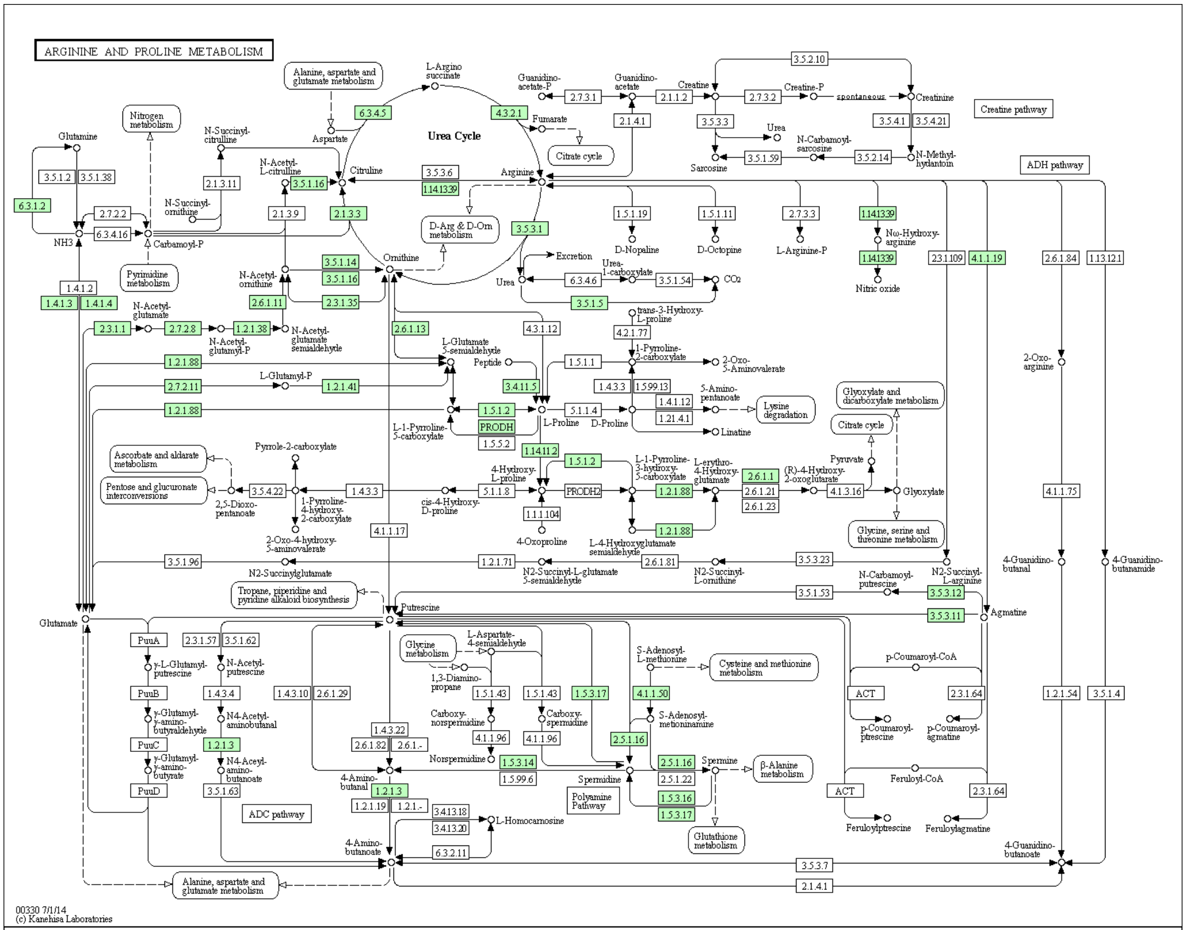
ARGININE AND PROLINE METABOLISM
Urea Cycle
L-Argino succinate
Alanine, aspartate and glutamate metabolism
Aspartate
Fumarate
Citrate cycle
Citrulline
Arginine
Ornithine
Urea
Excretion
D-Arg & D-Orn metabolism
Nitrogen metabolism
Pyrimidine metabolism
Glutamine
NH3
Carbamoyl-P
N-Succinyl-citrulline
N-Succinyl-ornithine
N-Acetyl-L-citrulline
N-Acetyl-ornithine
N-Acetyl-glutamate
N-Acetyl-glutamyl-P
N-Acetyl-glutamate semialdehyde
L-Glutamyl-P
L-Glutamate 5-semialdehyde
Peptide
L-1-Pyrroline-5-carboxylate
L-Proline
D-Proline
1-Pyrroline-2-carboxylate
2-Oxo-5-Aminovalerate
5-Amino-pentanoate
Lysine degradation
Linatine
trans-3-Hydroxy-L-proline
Guanidino-acetate-P
Guanidino-acetate
Creatine
Creatine-P
spontaneous
Creatinine
Creatine pathway
Sarcosine
N-Carbamoyl-sarcosine
N-Methyl-hydantoin
ADH pathway
D-Nopaline
D-Octopine
L-Arginine-P
Nω-Hydroxy-arginine
Nitric oxide
Urea-1-carboxylate
CO2
2-Oxo-arginine
4-Guanidino-butanal
4-Guanidino-butanamide
Ascorbate and aldarate metabolism
Pentose and glucuronate interconversions
Pyrrole-2-carboxylate
2,5-Dioxo-pentanoate
1-Pyrroline-4-hydroxy-2-carboxylate
2-Oxo-4-hydroxy-5-aminovalerate
cis-4-Hydroxy-D-proline
4-Hydroxy-L-proline
4-Oxoproline
L-1-Pyrroline-3-hydroxy-5-carboxylate
L-erythro-4-Hydroxy-glutamate
L-4-Hydroxyglutamate semialdehyde
(R)-4-Hydroxy-2-oxoglutarate
Pyruvate
Glyoxylate
Glyoxylate and dicarboxylate metabolism
Glycine, serine and threonine metabolism
N2-Succinylglutamate
N2-Succinyl-L-glutamate 5-semialdehyde
N2-Succinyl-L-ornithine
N2-Succinyl-L-arginine
N-Carbamoyl-putrescine
Agmatine
Glutamate
Putrescine
Tropane, piperidine and pyridine alkaloid biosynthesis
γ-L-Glutamyl-putrescine
γ-Glutamyl-γ-amino-butyraldehyde
γ-Glutamyl-γ-amino-butyrate
N-Acetyl-putrescine
N4-Acetyl-aminobutanal
N4-Acetyl-aminobutanoate
ADC pathway
Glycine metabolism
L-Aspartate-4-semialdehyde
1,3-Diamino-propane
Carboxy-norspermidine
Carboxy-spermidine
Norspermidine
S-Adenosyl-L-methionine
Cysteine and methionine metabolism
S-Adenosyl-methioninamine
Spermidine
Spermine
β-Alanine metabolism
Polyamine Pathway
Glutathione metabolism
4-Amino-butanal
4-Amino-butanoate
L-Homocarnosine
p-Coumaroyl-CoA
p-Coumaroyl-ptrescine
p-Coumaroyl-agmatine
Feruloyl-CoA
Feruloylptrescine
Feruloylagmatine
ACT
PuuA
PuuB
PuuC
PuuD
PRODH
PRODH2
4-Guanidino-butanoate
00330 7/1/14
(c) Kanehisa Laboratories

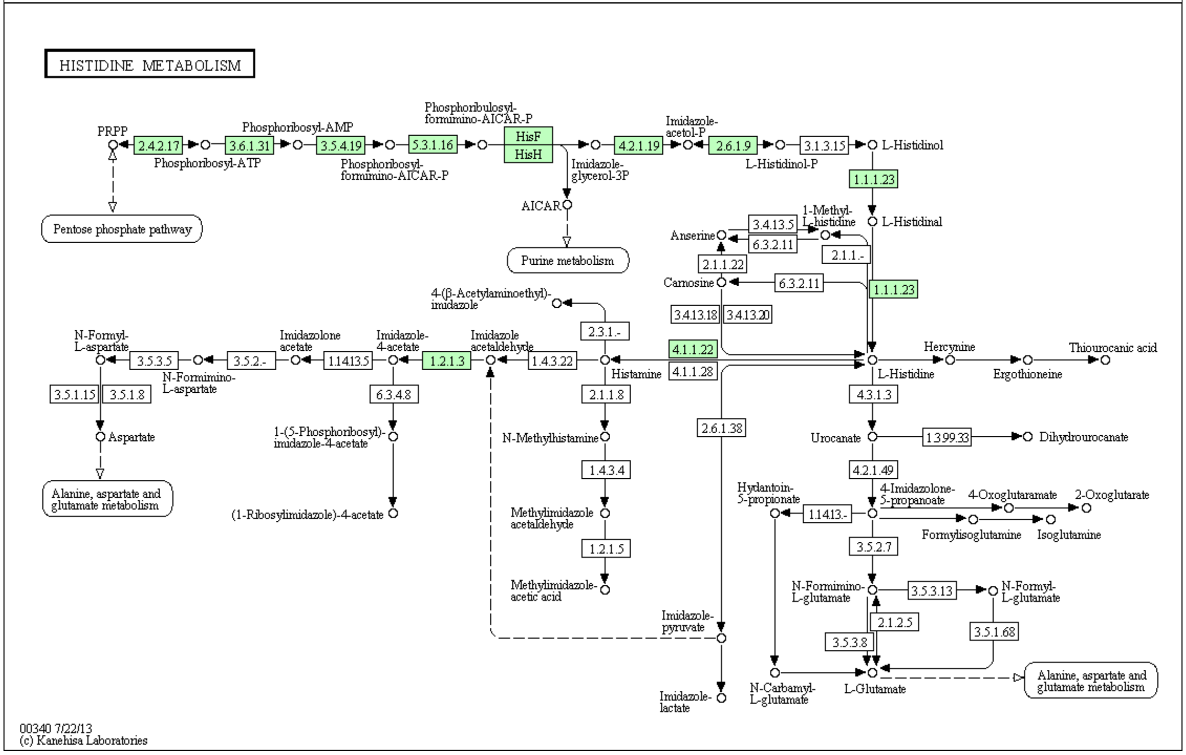
HISTIDINE METABOLISM
PRPP
Phosphoribosyl-ATP
Phosphoribosyl-AMP
Phosphoribosyl-formimino-AICAR-P
Phosphoribulosyl-formimino-AICAR-P
HisF
HisH
Imidazole-glycerol-3P
AICAR
Purine metabolism
Imidazole-acetol-P
L-Histidinol-P
L-Histidinol
L-Histidinal
Pentose phosphate pathway
1-Methyl-L-histidine
Anserine
Carnosine
L-Histidine
Hercynine
Ergothioneine
Thiourocanic acid
4-(β-Acetylaminoethyl)-imidazole
Histamine
Imidazole acetaldehyde
Imidazole-4-acetate
Imidazolone acetate
N-Formimino-L-aspartate
N-Formyl-L-aspartate
Aspartate
Alanine, aspartate and glutamate metabolism
1-(5-Phosphoribosyl)-imidazole-4-acetate
(1-Ribosylimidazole)-4-acetate
N-Methylhistamine
Methylimidazole acetaldehyde
Methylimidazole-acetic acid
Urocanate
Dihydrourocanate
4-Imidazolone-5-propanoate
Hydantoin-5-propionate
4-Oxoglutaramate
2-Oxoglutarate
Formylisoglutamine
Isoglutamine
N-Formimino-L-glutamate
N-Formyl-L-glutamate
N-Carbamyl-L-glutamate
L-Glutamate
Imidazole-pyruvate
Imidazole-lactate
00340 7/22/13
(c) Kanehisa Laboratories

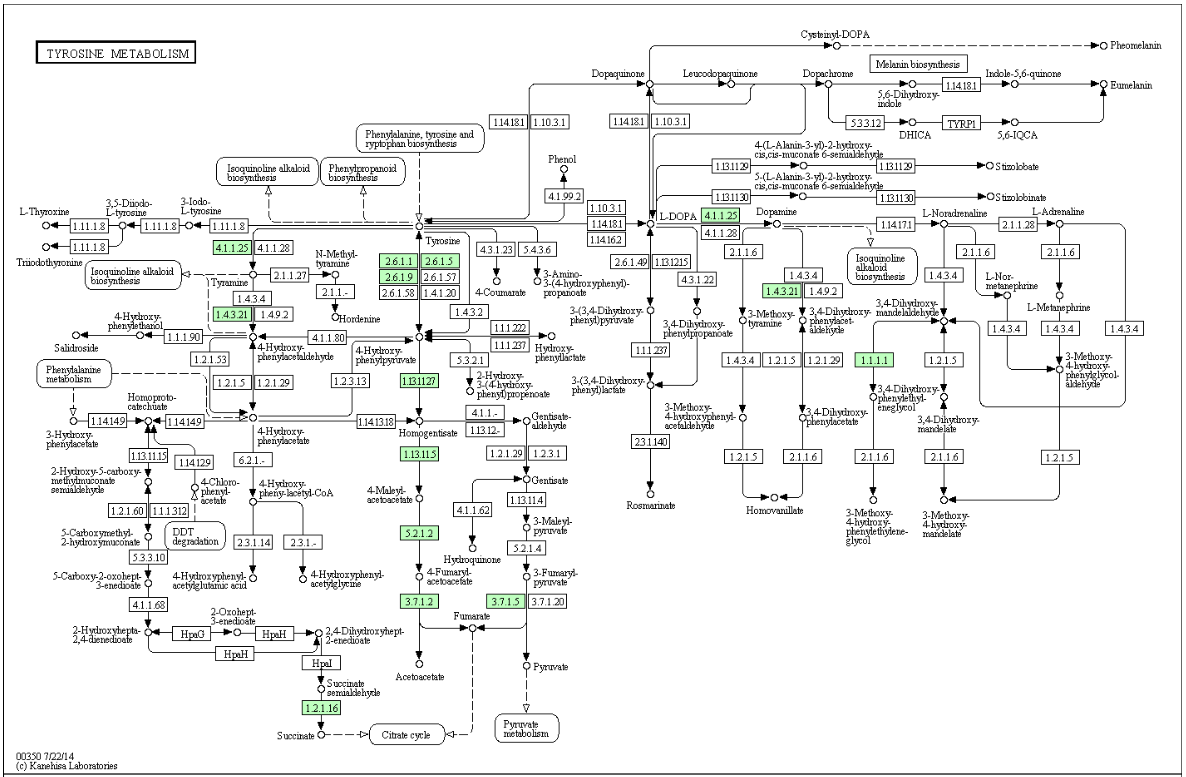
TYROSINE METABOLISM
Cysteinyl-DOPA
Pheomelanin
Dopaquinone
Leucodopaquinone
Dopachrome
Melanin biosynthesis
Indole-5,6-quinone
Eumelanin
5,6-Dihydroxy-indole
DHICA
5,6-IQCA
TYRP1
5.3.3.12
1.14.18.1
1.10.3.1
Phenylalanine, tyrosine and tryptophan biosynthesis
Isoquinoline alkaloid biosynthesis
Phenylpropanoid biosynthesis
Phenol
4.1.99.2
4-(L-Alanin-3-yl)-2-hydroxy-cis,cis-muconate 6-semialdehyde
5-(L-Alanin-3-yl)-2-hydroxy-cis,cis-muconate 6-semialdehyde
Stizolobate
Stizolobinate
1.13.11.29
1.13.11.30
L-Thyroxine
3,5-Diiodo-L-tyrosine
3-Iodo-L-tyrosine
Triiodothyronine
1.11.1.8
L-DOPA
4.1.1.25
4.1.1.28
Dopamine
L-Noradrenaline
L-Adrenaline
1.14.17.1
2.1.1.28
Tyrosine
N-Methyl-tyramine
Tyramine
Hordenine
4-Coumarate
3-Amino-3-(4-hydroxyphenyl)-propanoate
2.6.1.1
2.6.1.5
2.6.1.9
2.6.1.57
2.6.1.58
1.4.1.20
4.3.1.23
5.4.3.6
2.1.1.27
2.1.1.-
1.4.3.4
1.4.3.21
1.4.9.2
4-Hydroxy-phenylethanol
Salidroside
1.1.1.90
4.1.1.80
4-Hydroxy-phenylacetaldehyde
4-Hydroxy-phenylpyruvate
1.1.1.222
1.1.1.237
Hydroxy-phenyllactate
2-Hydroxy-3-(4-hydroxyphenyl)propenoate
5.3.2.1
1.2.1.53
1.2.1.5
1.2.1.29
1.2.3.13
1.13.11.27
Phenylalanine metabolism
Homoprotocatechuate
3-Hydroxy-phenylacetate
1.14.14.9
1.14.13.18
4-Hydroxy-phenylacetate
Homogentisate
Gentisate-aldehyde
4.1.1.-
1.13.12.-
1.13.11.15
1.14.12.9
6.2.1.-
1.13.11.5
2-Hydroxy-5-carboxy-methylmuconate semialdehyde
4-Chloro-phenyl-acetate
4-Hydroxy-pheny-lacetyl-CoA
4-Maleyl-acetoacetate
1.2.1.29
1.2.3.1
Gentisate
1.13.11.4
3-Maleyl-pyruvate
4.1.1.62
Hydroquinone
1.2.1.60
1.1.1.312
5-Carboxymethyl-2-hydroxymuconate
DDT degradation
5.3.3.10
2.3.1.14
2.3.1.-
5.2.1.2
5.2.1.4
4-Hydroxyphenyl-acetylglutamic acid
4-Hydroxyphenyl-acetylglycine
4-Fumaryl-acetoacetate
3-Fumaryl-pyruvate
5-Carboxy-2-oxohept-3-enedioate
4.1.1.68
3.7.1.2
3.7.1.5
3.7.1.20
2-Oxohept-3-enedioate
Fumarate
2-Hydroxyhepta-2,4-dienedioate
HpaG
HpaH
2,4-Dihydroxyhept-2-enedioate
HpaI
Acetoacetate
Pyruvate
Succinate semialdehyde
1.2.1.16
Succinate
Citrate cycle
Pyruvate metabolism
2.6.1.49
1.13.12.15
4.3.1.22
3-(3,4-Dihydroxy-phenyl)pyruvate
3,4-Dihydroxy-phenylpropanoate
1.1.1.237
3-(3,4-Dihydroxy-phenyl)lactate
3-Methoxy-4-hydroxyphenyl-acetaldehyde
2.3.1.140
Rosmarinate
2.1.1.6
3-Methoxy-tyramine
3,4-Dihydroxy-phenylacet-aldehyde
Isoquinoline alkaloid biosynthesis
1.1.1.1
1.2.1.5
1.2.1.29
3,4-Dihydroxy-phenylacetate
Homovanillate
3,4-Dihydroxy-mandelaldehyde
3,4-Dihydroxy-phenylethyl-eneglycol
3,4-Dihydroxy-mandelate
L-Nor-metanephrine
L-Metanephrine
3-Methoxy-4-hydroxy-phenylglycol-aldehyde
3-Methoxy-4-hydroxy-phenylethylene-glycol
3-Methoxy-4-hydroxy-mandelate
00350 7/22/14
(c) Kanehisa Laboratories

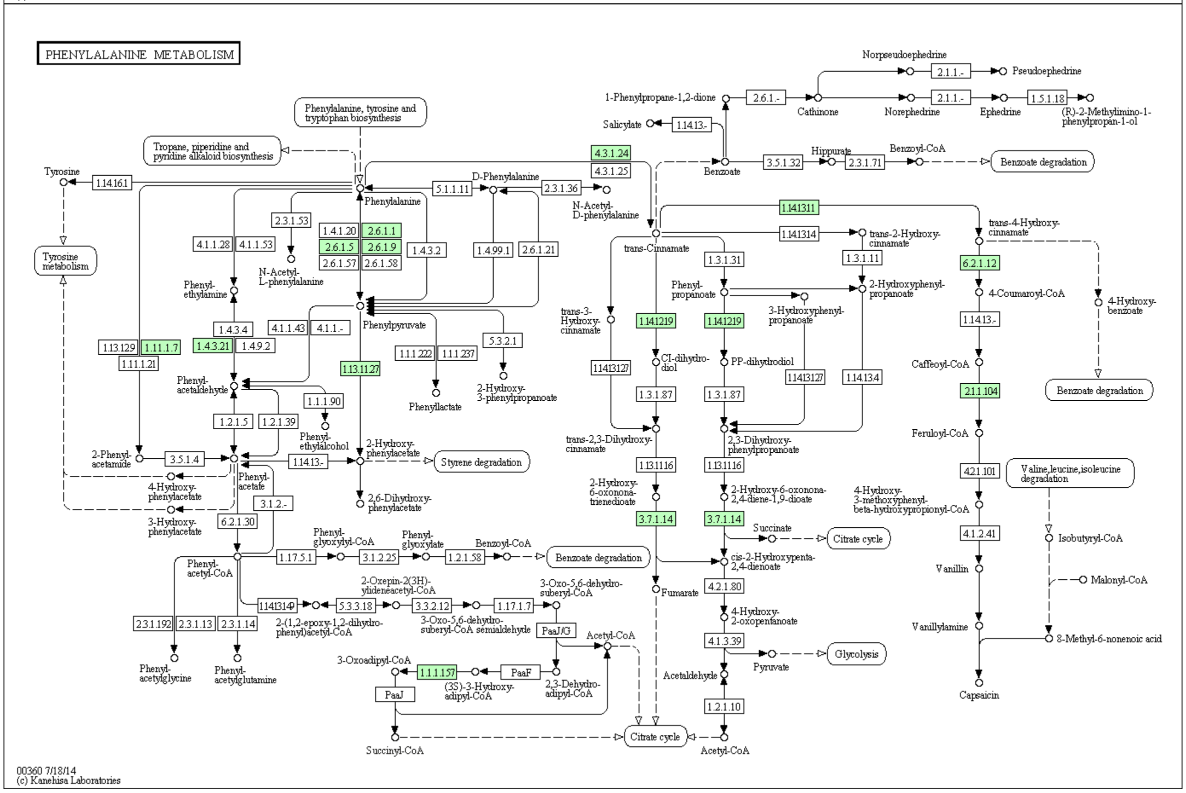
PHENYLALANINE METABOLISM
Norpseudoephedrine
Pseudoephedrine
1-Phenylpropane-1,2-dione
2.6.1.-
Cathinone
Norephedrine
Ephedrine
2.1.1.-
1.5.1.18
(R)-2-Methylimino-1-phenylpropan-1-ol
Salicylate
1.14.13.-
Phenylalanine, tyrosine and tryptophan biosynthesis
Tropane, piperidine and pyridine alkaloid biosynthesis
4.3.1.24
4.3.1.25
Benzoate
3.5.1.32
Hippurate
2.3.1.71
Benzoyl-CoA
Benzoate degradation
Tyrosine
1.14.16.1
Tyrosine metabolism
D-Phenylalanine
5.1.1.11
2.3.1.36
N-Acetyl-D-phenylalanine
Phenylalanine
1.14.13.11
1.14.13.14
trans-Cinnamate
trans-2-Hydroxy-cinnamate
trans-4-Hydroxy-cinnamate
2.3.1.53
4.1.1.28
4.1.1.53
1.4.1.20
2.6.1.1
2.6.1.5
2.6.1.9
2.6.1.57
2.6.1.58
1.4.3.2
1.4.99.1
2.6.1.21
N-Acetyl-L-phenylalanine
Phenyl-ethylamine
1.4.3.4
1.4.3.21
1.4.9.2
4.1.1.43
4.1.1.-
Phenylpyruvate
1.1.1.222
1.1.1.237
5.3.2.1
Phenyllactate
2-Hydroxy-3-phenylpropanoate
1.13.12.9
1.11.1.7
1.11.1.21
1.13.11.27
Phenyl-acetaldehyde
1.1.1.90
1.2.1.5
1.2.1.39
Phenyl-ethylalcohol
2-Phenyl-acetamide
3.5.1.4
Phenyl-acetate
1.14.13.-
2-Hydroxy-phenylacetate
Styrene degradation
2,6-Dihydroxy-phenylacetate
4-Hydroxy-phenylacetate
3-Hydroxy-phenylacetate
6.2.1.30
3.1.2.-
Phenyl-acetyl-CoA
Phenyl-glyoxylyl-CoA
Phenyl-glyoxylate
Benzoyl-CoA
1.17.5.1
3.1.2.25
1.2.1.58
2-Oxepin-2(3H)-ylideneacetyl-CoA
3-Oxo-5,6-dehydro-suberyl-CoA
1.14.13.149
5.3.3.18
3.3.2.12
1.17.1.7
2-(1,2-epoxy-1,2-dihydro-phenyl)acetyl-CoA
3-Oxo-5,6-dehydro-suberyl-CoA semialdehyde
PaaJ/G
Acetyl-CoA
2.3.1.192
2.3.1.13
2.3.1.14
Phenyl-acetylglycine
Phenyl-acetylglutamine
3-Oxoadipyl-CoA
1.1.1.157
PaaF
(3S)-3-Hydroxy-adipyl-CoA
2,3-Dehydro-adipyl-CoA
PaaJ
Succinyl-CoA
Citrate cycle
1.3.1.31
Phenyl-propanoate
3-Hydroxyphenyl-propanoate
1.3.1.11
2-Hydroxyphenyl-propanoate
trans-3-Hydroxy-cinnamate
1.14.12.19
CI-dihydro-diol
PP-dihydrodiol
1.14.13.127
1.14.13.4
1.3.1.87
trans-2,3-Dihydroxy-cinnamate
2,3-Dihydroxy-phenylpropanoate
1.13.11.16
2-Hydroxy-6-oxonona-trienedioate
2-Hydroxy-6-oxonona-2,4-diene-1,9-dioate
3.7.1.14
Succinate
cis-2-Hydroxypenta-2,4-dienoate
Fumarate
4.2.1.80
4-Hydroxy-2-oxopentanoate
4.1.3.39
Pyruvate
Glycolysis
Acetaldehyde
1.2.1.10
Acetyl-CoA
6.2.1.12
4-Coumaroyl-CoA
4-Hydroxy-benzoate
1.14.13.-
Caffeoyl-CoA
2.1.1.104
Benzoate degradation
Feruloyl-CoA
4.2.1.101
Valine, leucine, isoleucine degradation
4-Hydroxy-3-methoxyphenyl-beta-hydroxypropionoyl-CoA
4.1.2.41
Vanillin
Isobutyryl-CoA
Malonyl-CoA
Vanillylamine
8-Methyl-6-nonenoic acid
Capsaicin
00360 7/18/14
(c) Kanehisa Laboratories

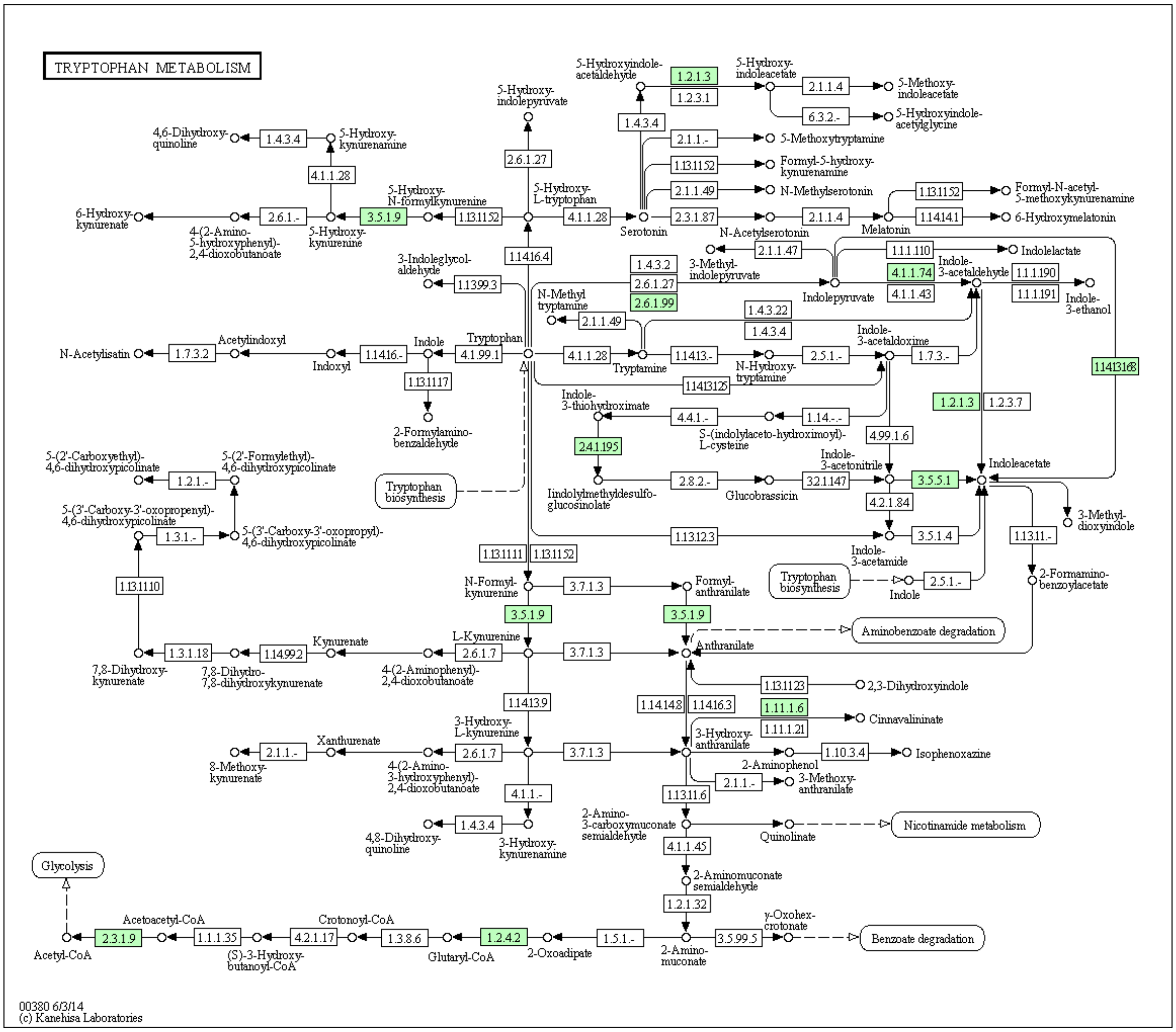
TRYPTOPHAN METABOLISM
5-Hydroxyindole-acetaldehyde
1.2.1.3
1.2.3.1
5-Hydroxy-indoleacetate
2.1.1.4
5-Methoxy-indoleacetate
6.3.2.-
5-Hydroxyindole-acetylglycine
5-Hydroxy-indolepyruvate
1.4.3.4
2.1.1.-
5-Methoxytryptamine
1.13.11.52
Formyl-5-hydroxy-kynurenamine
2.1.1.49
N-Methylserotonin
4,6-Dihydroxy-quinoline
5-Hydroxy-kynurenamine
2.6.1.27
4.1.1.28
5-Hydroxy-N-formylkynurenine
5-Hydroxy-L-tryptophan
6-Hydroxy-kynurenate
2.6.1.-
3.5.1.9
Serotonin
2.3.1.87
Melatonin
Formyl-N-acetyl-5-methoxykynurenamine
1.14.14.1
6-Hydroxymelatonin
4-(2-Amino-5-hydroxyphenyl)-2,4-dioxobutanoate
5-Hydroxy-kynurenine
N-Acetylserotonin
2.1.1.47
1.1.1.110
Indolelactate
1.14.16.4
3-Indoleglycol-aldehyde
1.4.3.2
3-Methyl-indolepyruvate
4.1.1.74
Indole-3-acetaldehyde
1.1.1.190
1.13.99.3
2.6.1.27
Indolepyruvate
4.1.1.43
1.1.1.191
Indole-3-ethanol
N-Methyl tryptamine
2.6.1.99
1.4.3.22
Acetylindoxyl
2.1.1.49
N-Acetylisatin
1.7.3.2
1.14.16.-
Indole
4.1.99.1
Tryptophan
Tryptamine
1.4.3.4
Indole-3-acetaldoxime
1.14.13.-
N-Hydroxy-tryptamine
2.5.1.-
1.7.3.-
Indoxyl
1.13.11.17
1.14.13.125
1.14.13.168
Indole-3-thiohydroximate
4.4.1.-
1.14.-.-
1.2.1.3
1.2.3.7
2-Formylamino-benzaldehyde
S-(indolylaceto-hydroximoyl)-L-cysteine
4.99.1.6
5-(2'-Carboxyethyl)-4,6-dihydroxypicolinate
5-(2'-Formylethyl)-4,6-dihydroxypicolinate
2.4.1.195
Indole-3-acetonitrile
1.2.1.-
Tryptophan biosynthesis
2.8.2.-
Glucobrassicin
3.2.1.147
3.5.5.1
Indoleacetate
Indolylmethyl-desulfo-glucosinolate
4.2.1.84
3-Methyl-dioxyindole
5-(3'-Carboxy-3'-oxopropenyl)-4,6-dihydroxypicolinate
1.3.1.-
5-(3'-Carboxy-3'-oxopropyl)-4,6-dihydroxypicolinate
1.13.12.3
3.5.1.4
1.13.11.-
Indole-3-acetamide
1.13.11.11
1.13.11.52
Tryptophan biosynthesis
2-Formamino-benzoylacetate
Indole
2.5.1.-
1.13.11.10
N-Formyl-kynurenine
3.7.1.3
Formyl-anthranilate
3.5.1.9
3.5.1.9
Aminobenzoate degradation
Kynurenate
L-Kynurenine
Anthranilate
1.3.1.18
1.14.99.2
2.6.1.7
3.7.1.3
7,8-Dihydroxy-kynurenate
7,8-Dihydro-7,8-dihydroxykynurenate
4-(2-Aminophenyl)-2,4-dioxobutanoate
1.13.11.23
2,3-Dihydroxyindole
1.14.13.9
1.14.14.8
1.14.16.3
1.11.1.6
Cinnavalininate
3-Hydroxy-L-kynurenine
1.11.1.21
Xanthurenate
3-Hydroxy-anthranilate
2.1.1.-
2.6.1.7
3.7.1.3
1.10.3.4
Isophenoxazine
2-Aminophenol
8-Methoxy-kynurenate
4-(2-Amino-3-hydroxyphenyl)-2,4-dioxobutanoate
2.1.1.-
3-Methoxy-anthranilate
4.1.1.-
1.13.11.6
2-Amino-3-carboxymuconate semialdehyde
1.4.3.4
4,8-Dihydroxy-quinoline
3-Hydroxy-kynurenamine
Quinolinate
Nicotinamide metabolism
4.1.1.45
Glycolysis
2-Aminomuconate semialdehyde
1.2.1.32
Acetoacetyl-CoA
Crotonoyl-CoA
γ-Oxohex-crotonate
2.3.1.9
1.1.1.35
4.2.1.17
1.3.8.6
1.2.4.2
1.5.1.-
3.5.99.5
Benzoate degradation
Acetyl-CoA
(S)-3-Hydroxy-butanoyl-CoA
Glutaryl-CoA
2-Oxoadipate
2-Amino-muconate
00380 6/3/14
(c) Kanehisa Laboratories

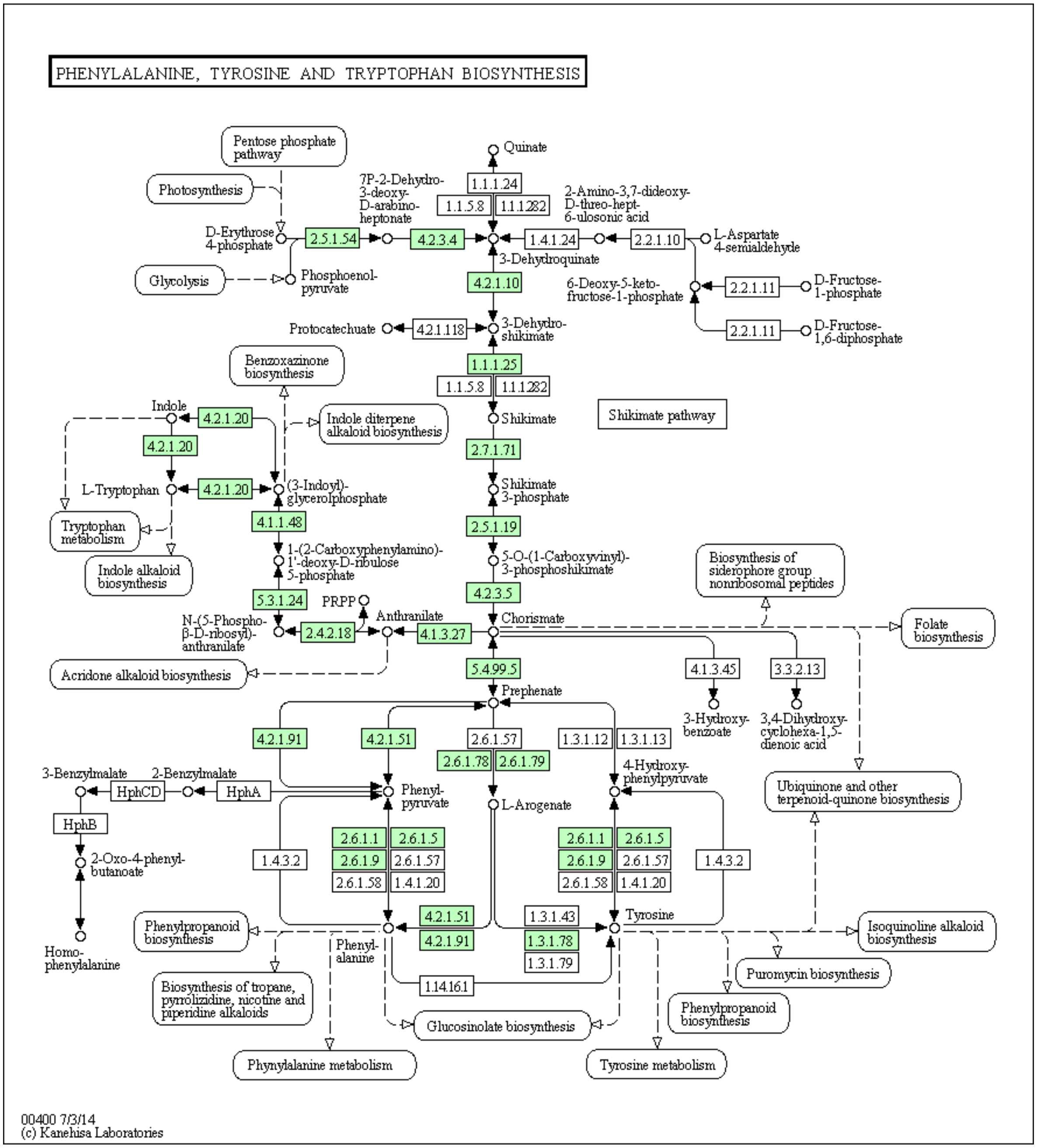
PHENYLALANINE, TYROSINE AND TRYPTOPHAN BIOSYNTHESIS
Pentose phosphate pathway
Photosynthesis
Glycolysis
D-Erythrose 4-phosphate
Phosphoenol-pyruvate
2.5.1.54
7P-2-Dehydro-3-deoxy-D-arabino-heptonate
4.2.3.4
Quinate
1.1.1.24
1.1.5.8
1.1.1282
2-Amino-3,7-dideoxy-D-threo-hept-6-ulosonic acid
1.4.1.24
2.2.1.10
L-Aspartate 4-semialdehyde
3-Dehydroquinate
4.2.1.10
6-Deoxy-5-keto-fructose-1-phosphate
2.2.1.11
D-Fructose-1-phosphate
2.2.1.11
D-Fructose-1,6-diphosphate
Protocatechuate
4.2.1.118
3-Dehydro-shikimate
1.1.1.25
1.1.5.8
1.1.1282
Shikimate
Shikimate pathway
2.7.1.71
Shikimate 3-phosphate
2.5.1.19
5-O-(1-Carboxyvinyl)-3-phosphoshikimate
4.2.3.5
Chorismate
Benzoxazinone biosynthesis
Indole
4.2.1.20
Indole diterpene alkaloid biosynthesis
4.2.1.20
L-Tryptophan
4.2.1.20
(3-Indoyl)-glycerolphosphate
Tryptophan metabolism
4.1.1.48
1-(2-Carboxyphenylamino)-1'-deoxy-D-ribulose 5-phosphate
Indole alkaloid biosynthesis
5.3.1.24
PRPP
N-(5-Phospho-β-D-ribosyl)-anthranilate
2.4.2.18
Anthranilate
4.1.3.27
Acridone alkaloid biosynthesis
Biosynthesis of siderophore group nonribosomal peptides
Folate biosynthesis
5.4.99.5
Prephenate
4.1.3.45
3.3.2.13
3-Hydroxy-benzoate
3,4-Dihydroxy-cyclohexa-1,5-dienoic acid
4.2.1.91
4.2.1.51
2.6.1.57
2.6.1.78
2.6.1.79
1.3.1.12
1.3.1.13
4-Hydroxy-phenylpyruvate
Ubiquinone and other terpenoid-quinone biosynthesis
3-Benzylmalate
2-Benzylmalate
HphCD
HphA
Phenyl-pyruvate
L-Arogenate
HphB
2-Oxo-4-phenyl-butanoate
1.4.3.2
2.6.1.1
2.6.1.5
2.6.1.9
2.6.1.57
2.6.1.58
1.4.1.20
2.6.1.1
2.6.1.5
2.6.1.9
2.6.1.57
2.6.1.58
1.4.1.20
1.4.3.2
Homo-phenylalanine
Phenylpropanoid biosynthesis
4.2.1.51
4.2.1.91
Phenyl-alanine
1.3.1.43
1.3.1.78
1.3.1.79
Tyrosine
Isoquinoline alkaloid biosynthesis
Puromycin biosynthesis
Biosynthesis of tropane, pyrrolizidine, nicotine and piperidine alkaloids
1.14.16.1
Phenylpropanoid biosynthesis
Glucosinolate biosynthesis
Phynylalanine metabolism
Tyrosine metabolism
00400 7/3/14
(c) Kanehisa Laboratories

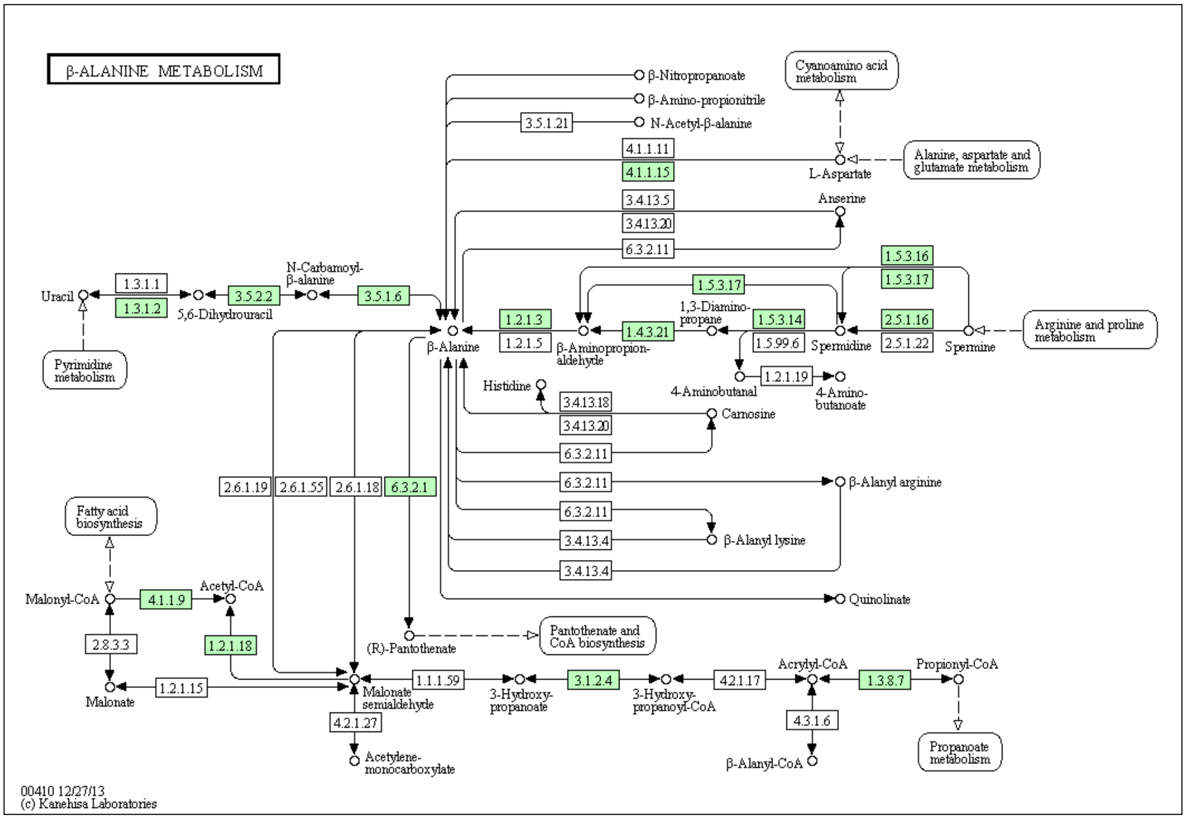
β-ALANINE METABOLISM
β-Nitropropanoate
β-Amino-propionitrile
3.5.1.21
N-Acetyl-β-alanine
Cyanoamino acid metabolism
4.1.1.11
4.1.1.15
L-Aspartate
Alanine, aspartate and glutamate metabolism
3.4.13.5
3.4.13.20
6.3.2.11
Anserine
1.5.3.16
1.5.3.17
1.5.3.17
Uracil
1.3.1.1
1.3.1.2
5,6-Dihydrouracil
3.5.2.2
N-Carbamoyl-β-alanine
3.5.1.6
β-Alanine
1.2.1.3
1.2.1.5
β-Aminopropion-aldehyde
1.4.3.21
1,3-Diamino-propane
1.5.3.14
1.5.99.6
Spermidine
2.5.1.16
2.5.1.22
Spermine
Arginine and proline metabolism
Pyrimidine metabolism
4-Aminobutanal
1.2.1.19
4-Amino-butanoate
Histidine
3.4.13.18
3.4.13.20
Carnosine
6.3.2.11
2.6.1.19
2.6.1.55
2.6.1.18
6.3.2.1
6.3.2.11
β-Alanyl arginine
6.3.2.11
Fatty acid biosynthesis
3.4.13.4
β-Alanyl lysine
3.4.13.4
Malonyl-CoA
4.1.1.9
Acetyl-CoA
Quinolinate
(R)-Pantothenate
Pantothenate and CoA biosynthesis
2.8.3.3
1.2.1.18
Malonate
1.2.1.15
Malonate semialdehyde
1.1.1.59
3-Hydroxy-propanoate
3.1.2.4
3-Hydroxy-propanoyl-CoA
4.2.1.17
Acrylyl-CoA
1.3.8.7
Propionyl-CoA
4.2.1.27
Acetylene-monocarboxylate
4.3.1.6
β-Alanyl-CoA
Propanoate metabolism
00410 12/27/13
(c) Kanehisa Laboratories

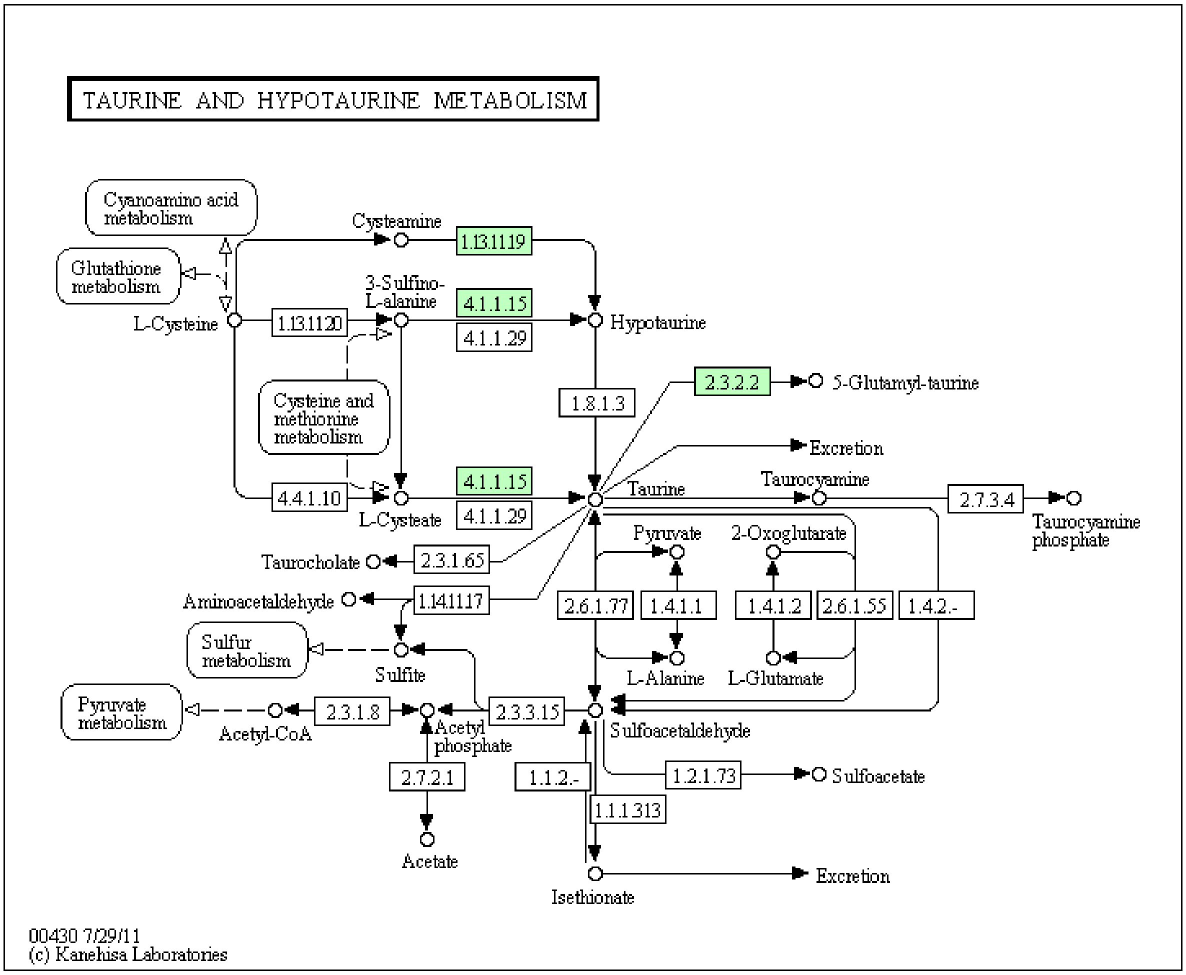

TAURINE AND HYPOTAURINE METABOLISM
Cyanoamino acid metabolism
Glutathione metabolism
Cysteamine
1.13.11.19
3-Sulfino-L-alanine
4.1.1.15
4.1.1.29
L-Cysteine
1.13.11.20
Hypotaurine
Cysteine and methionine metabolism
1.8.1.3
2.3.2.2
5-Glutamyl-taurine
Excretion
4.4.1.10
L-Cysteate
4.1.1.15
4.1.1.29
Taurine
Taurocyamine
2.7.3.4
Taurocyamine phosphate
Taurocholate
2.3.1.65
Pyruvate
2-Oxoglutarate
Aminoacetaldehyde
1.14.11.17
2.6.1.77
1.4.1.1
1.4.1.2
2.6.1.55
1.4.2.-
Sulfur metabolism
Sulfite
L-Alanine
L-Glutamate
Pyruvate metabolism
Acetyl-CoA
2.3.1.8
Acetyl phosphate
2.3.3.15
Sulfoacetaldehyde
2.7.2.1
1.1.2.-
1.2.1.73
Sulfoacetate
1.1.1.313
Acetate
Isethionate
Excretion
00430 7/29/11
(c) Kanehisa Laboratories

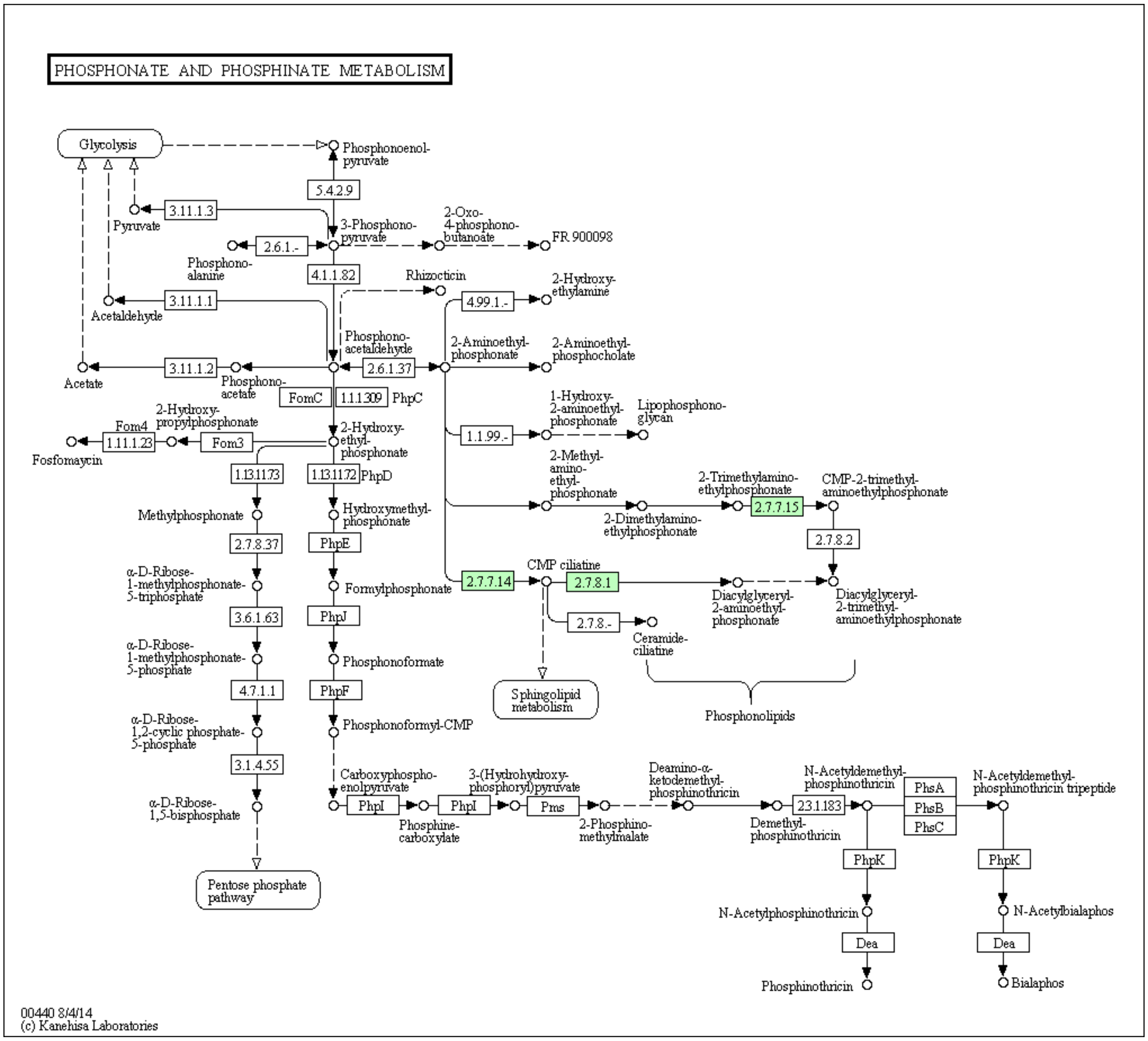
PHOSPHONATE AND PHOSPHINATE METABOLISM
Glycolysis
Phosphonoenol-pyruvate
5.4.2.9
3.11.1.3
Pyruvate
3-Phosphono-pyruvate
2-Oxo-4-phosphono-butanoate
FR 900098
2.6.1.-
Phosphono-alanine
4.1.1.82
Rhizocticin
3.11.1.1
Acetaldehyde
4.99.1.-
2-Hydroxy-ethylamine
Phosphono-acetaldehyde
2-Aminoethyl-phosphonate
2-Aminoethyl-phosphocholate
3.11.1.2
2.6.1.37
Acetate
Phosphono-acetate
FomC
1.1.1.309
PhpC
1-Hydroxy-2-aminoethyl-phosphonate
Lipophosphono-glycan
2-Hydroxy-propylphosphonate
Fom4
1.11.1.23
Fom3
2-Hydroxy-ethyl-phosphonate
1.1.99.-
Fosfomaycin
1.13.11.73
1.13.11.72
PhpD
2-Methyl-amino-ethyl-phosphonate
2-Trimethylamino-ethylphosphonate
CMP-2-trimethyl-aminoethylphosphonate
Methylphosphonate
Hydroxymethyl-phosphonate
2-Dimethylamino-ethylphosphonate
2.7.7.15
2.7.8.37
PhpE
2.7.8.2
α-D-Ribose-1-methylphosphonate-5-triphosphate
Formylphosphonate
2.7.7.14
CMP ciliatine
2.7.8.1
Diacylglyceryl-2-aminoethyl-phosphonate
Diacylglyceryl-2-trimethyl-aminoethylphosphonate
3.6.1.63
PhpJ
2.7.8.-
Ceramide-ciliatine
α-D-Ribose-1-methylphosphonate-5-phosphate
Phosphonoformate
4.7.1.1
PhpF
Sphingolipid metabolism
Phosphonolipids
α-D-Ribose-1,2-cyclic phosphate-5-phosphate
Phosphonoformyl-CMP
3.1.4.55
Carboxyphospho-enolpyruvate
3-(Hydrohydroxy-phosphoryl)pyruvate
Deamino-α-ketodemethyl-phosphinothricin
N-Acetyldemethyl-phosphinothricin
N-Acetyldemethyl-phosphinothricin tripeptide
α-D-Ribose-1,5-bisphosphate
PhpI
PhpI
Pms
2.3.1.183
PhsA
PhsB
PhsC
Phosphine-carboxylate
2-Phosphino-methylmalate
Demethyl-phosphinothricin
PhpK
PhpK
Pentose phosphate pathway
N-Acetylphosphinothricin
N-Acetylbialaphos
Dea
Dea
Phosphinothricin
Bialaphos
00440 8/4/14
(c) Kanehisa Laboratories

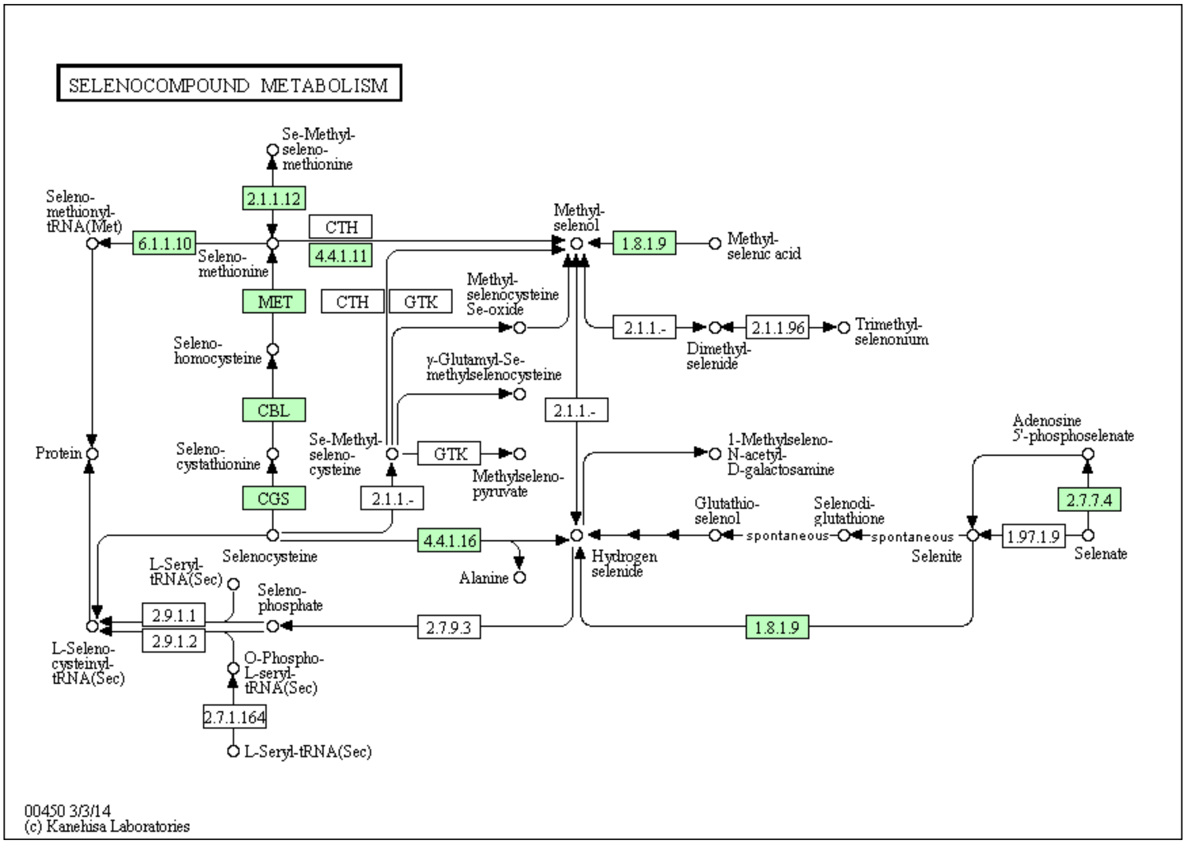
SELENOCOMPOUND METABOLISM
Se-Methyl-seleno-methionine
2.1.1.12
Seleno-methionyl-tRNA(Met)
6.1.1.10
CTH
4.4.1.11
Seleno-methionine
Methyl-selenol
1.8.1.9
Methyl-selenic acid
MET
CTH
GTK
Methyl-selenocysteine Se-oxide
2.1.1.-
2.1.1.96
Trimethyl-selenonium
Dimethyl-selenide
Seleno-homocysteine
γ-Glutamyl-Se-methylselenocysteine
CBL
2.1.1.-
Adenosine 5'-phosphoselenate
Protein
Seleno-cystathionine
Se-Methyl-seleno-cysteine
GTK
1-Methylseleno-N-acetyl-D-galactosamine
Methylseleno-pyruvate
2.7.7.4
CGS
2.1.1.-
Glutathio-selenol
Selenodi-glutathione
4.4.1.16
spontaneous
spontaneous
1.97.1.9
Selenocysteine
Hydrogen selenide
Selenite
Selenate
L-Seryl-tRNA(Sec)
Alanine
Seleno-phosphate
2.9.1.1
2.7.9.3
1.8.1.9
2.9.1.2
L-Seleno-cysteinyl-tRNA(Sec)
O-Phospho-L-seryl-tRNA(Sec)
2.7.1.164
L-Seryl-tRNA(Sec)
00450 3/3/14
(c) Kanehisa Laboratories

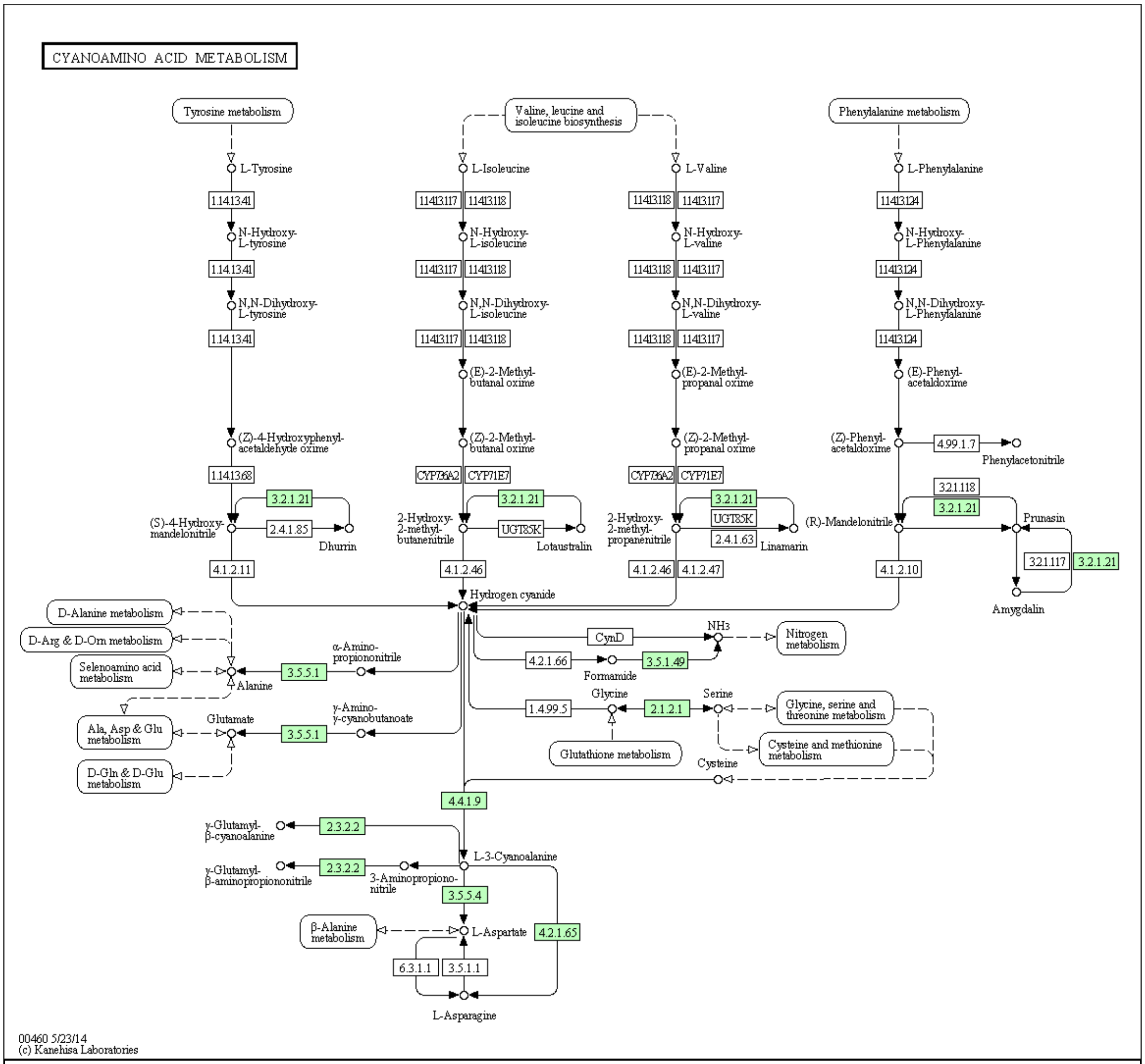
CYANOAMINO ACID METABOLISM
Tyrosine metabolism
Valine, leucine and isoleucine biosynthesis
Phenylalanine metabolism
L-Tyrosine
L-Isoleucine
L-Valine
L-Phenylalanine
N-Hydroxy-L-tyrosine
N-Hydroxy-L-isoleucine
N-Hydroxy-L-valine
N-Hydroxy-L-Phenylalanine
N,N-Dihydroxy-L-tyrosine
N,N-Dihydroxy-L-isoleucine
N,N-Dihydroxy-L-valine
N,N-Dihydroxy-L-Phenylalanine
(E)-2-Methyl-butanal oxime
(E)-2-Methyl-propanal oxime
(E)-Phenyl-acetaldoxime
(Z)-4-Hydroxyphenyl-acetaldehyde oxime
(Z)-2-Methyl-butanal oxime
(Z)-2-Methyl-propanal oxime
(Z)-Phenyl-acetaldoxime
4.99.1.7
Phenylacetonitrile
CYP79A2
CYP71E7
3.2.1.21
2.4.1.85
UGT85K
2.4.1.63
3.2.1.118
3.2.1.117
(S)-4-Hydroxy-mandelonitrile
Dhurrin
2-Hydroxy-2-methyl-butanenitrile
Lotaustralin
2-Hydroxy-2-methyl-propanenitrile
Linamarin
(R)-Mandelonitrile
Prunasin
Amygdalin
4.1.2.11
4.1.2.46
4.1.2.47
4.1.2.10
Hydrogen cyanide
D-Alanine metabolism
D-Arg & D-Orn metabolism
Selenoamino acid metabolism
Alanine
3.5.5.1
α-Amino-propiononitrile
CynD
NH3
Nitrogen metabolism
4.2.1.66
Formamide
3.5.1.49
1.4.99.5
Glycine
2.1.2.1
Serine
Glycine, serine and threonine metabolism
Glutathione metabolism
Cysteine and methionine metabolism
Cysteine
Ala, Asp & Glu metabolism
Glutamate
γ-Amino-γ-cyanobutanoate
D-Gln & D-Glu metabolism
4.4.1.9
γ-Glutamyl-β-cyanoalanine
2.3.2.2
L-3-Cyanoalanine
γ-Glutamyl-β-aminopropionitrile
3-Aminopropiono-nitrile
3.5.5.4
β-Alanine metabolism
L-Aspartate
4.2.1.65
6.3.1.1
3.5.1.1
L-Asparagine
00460 5/23/14
(c) Kanehisa Laboratories

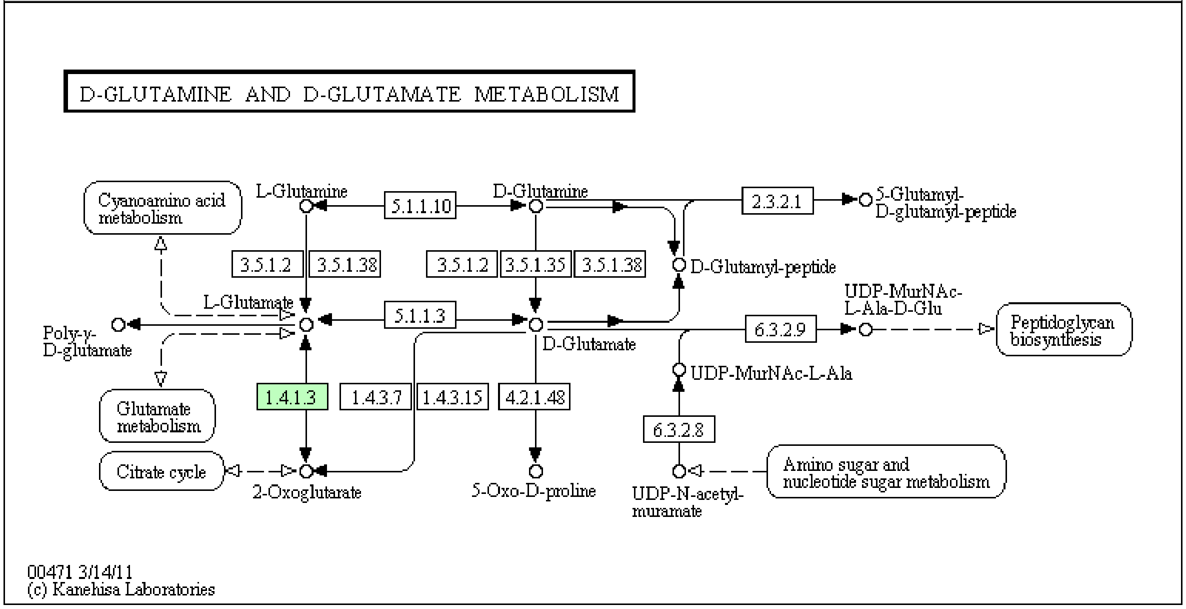
D-GLUTAMINE AND D-GLUTAMATE METABOLISM
Cyanoamino acid metabolism
L-Glutamine
5.1.1.10
D-Glutamine
2.3.2.1
5-Glutamyl-D-glutamyl-peptide
3.5.1.2
3.5.1.38
3.5.1.35
D-Glutamyl-peptide
L-Glutamate
5.1.1.3
UDP-MurNAc-L-Ala-D-Glu
Poly-γ-D-glutamate
6.3.2.9
Peptidoglycan biosynthesis
D-Glutamate
UDP-MurNAc-L-Ala
Glutamate metabolism
1.4.1.3
1.4.3.7
1.4.3.15
4.2.1.48
6.3.2.8
Citrate cycle
Amino sugar and nucleotide sugar metabolism
2-Oxoglutarate
5-Oxo-D-proline
UDP-N-acetyl-muramate
00471 3/14/11
(c) Kanehisa Laboratories

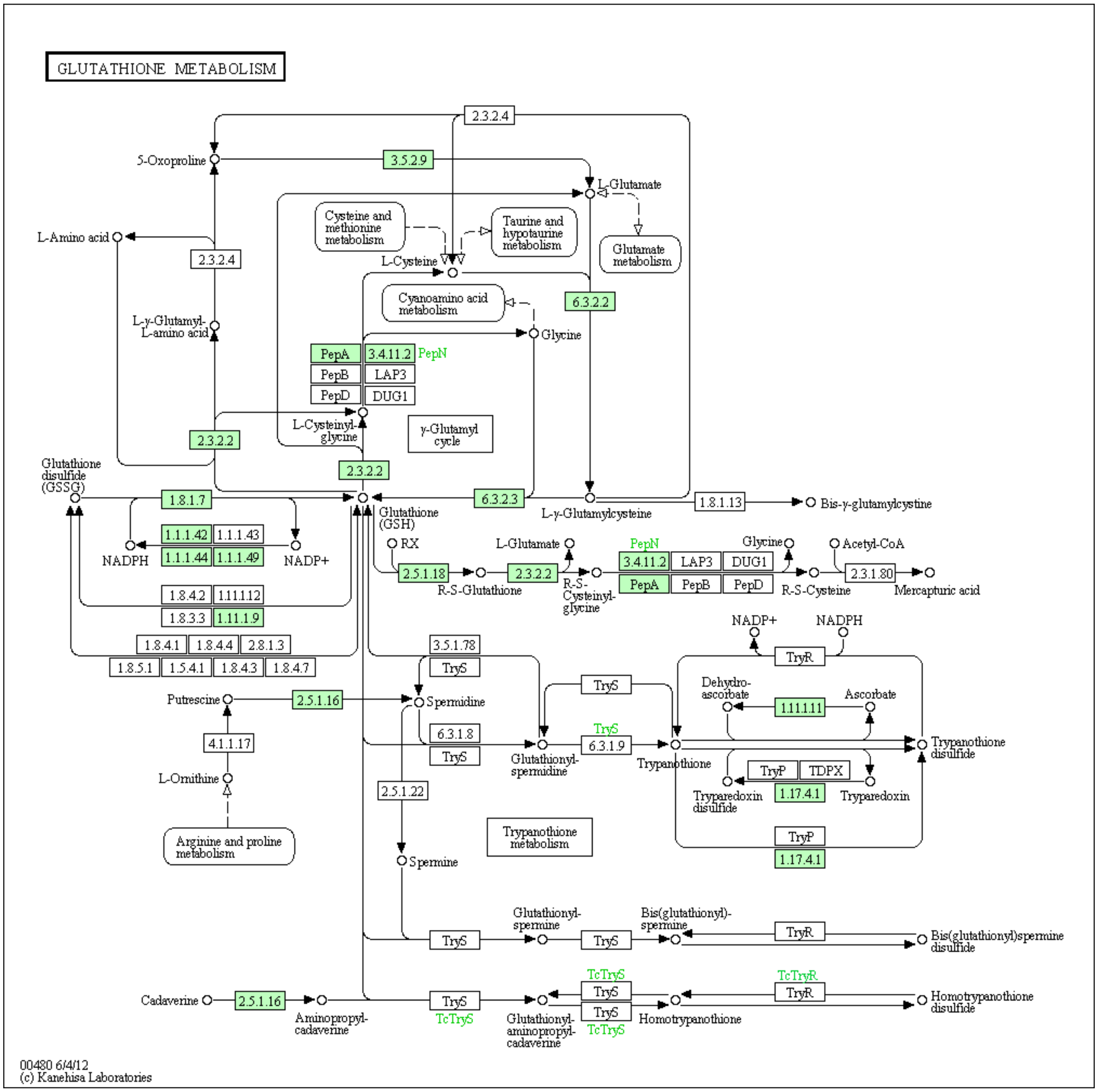
GLUTATHIONE METABOLISM
2.3.2.4
5-Oxoproline
3.5.2.9
L-Glutamate
Cysteine and methionine metabolism
Taurine and hypotaurine metabolism
Glutamate metabolism
L-Amino acid
2.3.2.4
L-Cysteine
Cyanoamino acid metabolism
6.3.2.2
L-γ-Glutamyl-L-amino acid
Glycine
PepA
3.4.11.2
PepN
PepB
LAP3
PepD
DUG1
L-Cysteinyl-glycine
γ-Glutamyl cycle
2.3.2.2
2.3.2.2
Glutathione disulfide (GSSG)
1.8.1.7
6.3.2.3
L-γ-Glutamylcysteine
1.8.1.13
Bis-γ-glutamylcystine
Glutathione (GSH)
1.1.1.42
1.1.1.43
1.1.1.44
1.1.1.49
NADPH
NADP+
RX
L-Glutamate
PepN
Glycine
Acetyl-CoA
2.5.1.18
2.3.2.2
3.4.11.2
LAP3
DUG1
2.3.1.80
R-S-Glutathione
R-S-Cysteinyl-glycine
PepA
PepB
PepD
R-S-Cysteine
Mercapturic acid
1.8.4.2
1.11.1.12
1.8.3.3
1.11.1.9
NADP+
NADPH
1.8.4.1
1.8.4.4
2.8.1.3
3.5.1.78
TryR
1.8.5.1
1.5.4.1
1.8.4.3
1.8.4.7
TryS
TryS
Dehydro-ascorbate
Ascorbate
Putrescine
2.5.1.16
Spermidine
1.11.1.11
TryS
6.3.1.8
4.1.1.17
TryS
6.3.1.9
Trypanothione disulfide
Glutathionyl-spermidine
Trypanothione
L-Ornithine
TryP
TDPX
2.5.1.22
Tryparedoxin disulfide
1.17.4.1
Tryparedoxin
Trypanothione metabolism
Arginine and proline metabolism
TryP
1.17.4.1
Spermine
Glutathionyl-spermine
Bis(glutathionyl)-spermine
TryS
TryS
TryR
Bis(glutathionyl)spermine disulfide
TcTryS
TcTryR
Cadaverine
2.5.1.16
TryS
TryS
TryR
Homotrypanothione disulfide
Aminopropyl-cadaverine
TcTryS
TryS
Glutathionyl-aminopropyl-cadaverine
TcTryS
Homotrypanothione
00480 6/4/12
(c) Kanehisa Laboratories

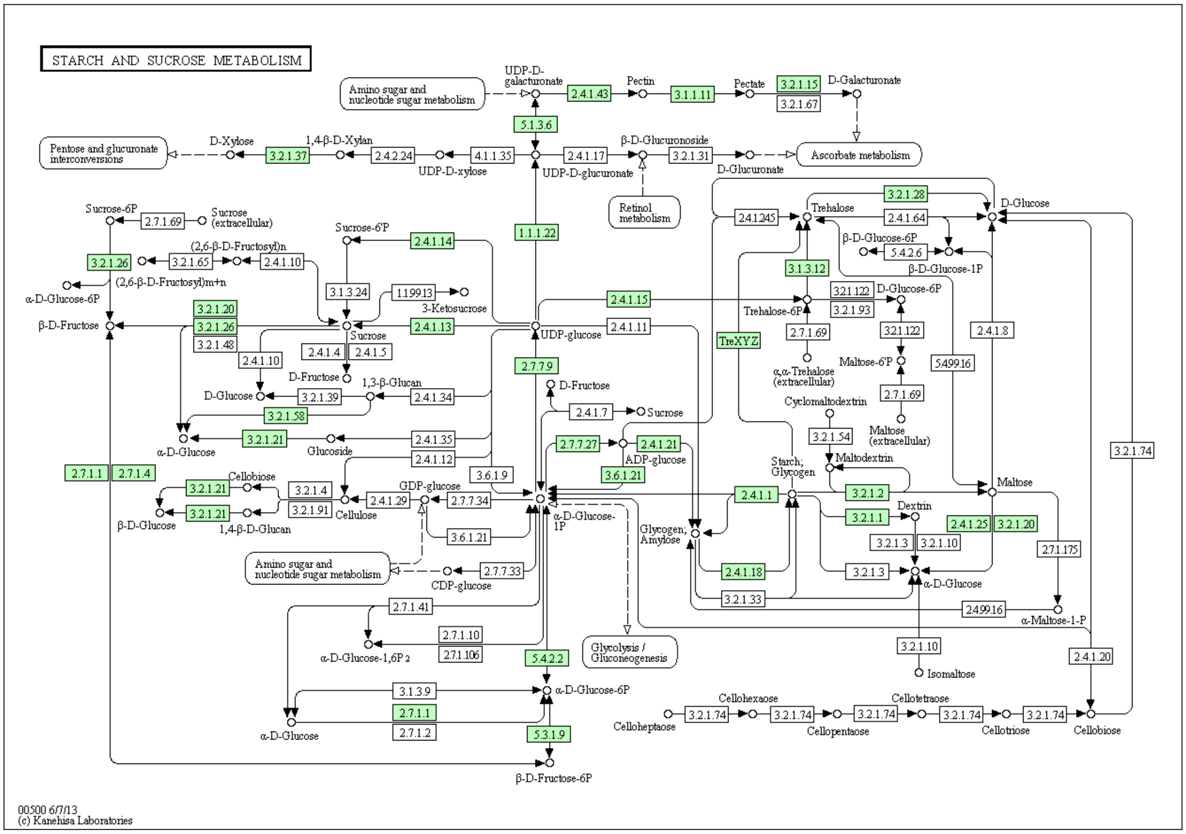
STARCH AND SUCROSE METABOLISM
Amino sugar and nucleotide sugar metabolism
UDP-D-galacturonate
2.4.1.43
Pectin
3.1.1.11
Pectate
3.2.1.15
3.2.1.67
D-Galacturonate
5.1.3.6
Pentose and glucuronate interconversions
D-Xylose
3.2.1.37
1,4-β-D-Xylan
2.4.2.24
UDP-D-xylose
4.1.1.35
UDP-D-glucuronate
2.4.1.17
β-D-Glucuronoside
3.2.1.31
D-Glucuronate
Ascorbate metabolism
Retinol metabolism
Sucrose-6P
2.7.1.69
Sucrose (extracellular)
3.2.1.26
3.2.1.65
(2,6-β-D-Fructosyl)n
2.4.1.10
(2,6-β-D-Fructosyl)m+n
α-D-Glucose-6P
Sucrose-6'P
2.4.1.14
1.1.1.22
3.1.3.24
1.1.99.13
3-Ketosucrose
3.2.1.20
3.2.1.26
3.2.1.48
β-D-Fructose
Sucrose
2.4.1.13
UDP-glucose
2.4.1.11
2.4.1.15
2.4.1.4
2.4.1.5
2.4.1.10
D-Fructose
1,3-β-Glucan
D-Glucose
3.2.1.39
2.4.1.34
3.2.1.58
2.7.7.9
D-Fructose
2.4.1.7
Sucrose
α-D-Glucose
3.2.1.21
Glucoside
2.4.1.35
2.4.1.12
2.7.7.27
2.4.1.21
ADP-glucose
3.6.1.21
2.7.1.1
2.7.1.4
Cellobiose
3.2.1.21
3.2.1.4
3.2.1.91
3.6.1.9
GDP-glucose
2.4.1.29
2.7.7.34
Cellulose
β-D-Glucose
3.2.1.21
1,4-β-D-Glucan
3.6.1.21
α-D-Glucose-1P
Amino sugar and nucleotide sugar metabolism
CDP-glucose
2.7.7.33
2.7.1.41
α-D-Glucose-1,6P2
2.7.1.10
2.7.1.106
5.4.2.2
Glycolysis / Gluconeogenesis
3.1.3.9
α-D-Glucose-6P
2.7.1.1
2.7.1.2
α-D-Glucose
5.3.1.9
β-D-Fructose-6P
3.2.1.28
D-Glucose
2.4.1.245
Trehalose
2.4.1.64
β-D-Glucose-6P
5.4.2.6
β-D-Glucose-1P
3.1.3.12
3.2.1.122
D-Glucose-6P
Trehalose-6P
3.2.1.93
2.4.1.8
TreXYZ
2.4.1.69
3.2.1.122
α,α-Trehalose (extracellular)
Maltose-6'P
5.4.99.16
2.7.1.69
Cyclomaltodextrin
3.2.1.54
Maltose (extracellular)
Maltodextrin
Starch; Glycogen
3.2.1.2
Maltose
3.2.1.74
2.4.1.1
3.2.1.1
Dextrin
2.4.1.25
3.2.1.20
Glycogen; Amylose
3.2.1.3
3.2.1.10
2.7.1.175
2.4.1.18
3.2.1.3
α-D-Glucose
3.2.1.33
2.4.99.16
α-Maltose-1-P
3.2.1.10
Isomaltose
2.4.1.20
Celloheptaose
3.2.1.74
Cellohexaose
3.2.1.74
Cellopentaose
3.2.1.74
Cellotetraose
3.2.1.74
Cellotriose
3.2.1.74
Cellobiose
00500 6/7/13
(c) Kanehisa Laboratories

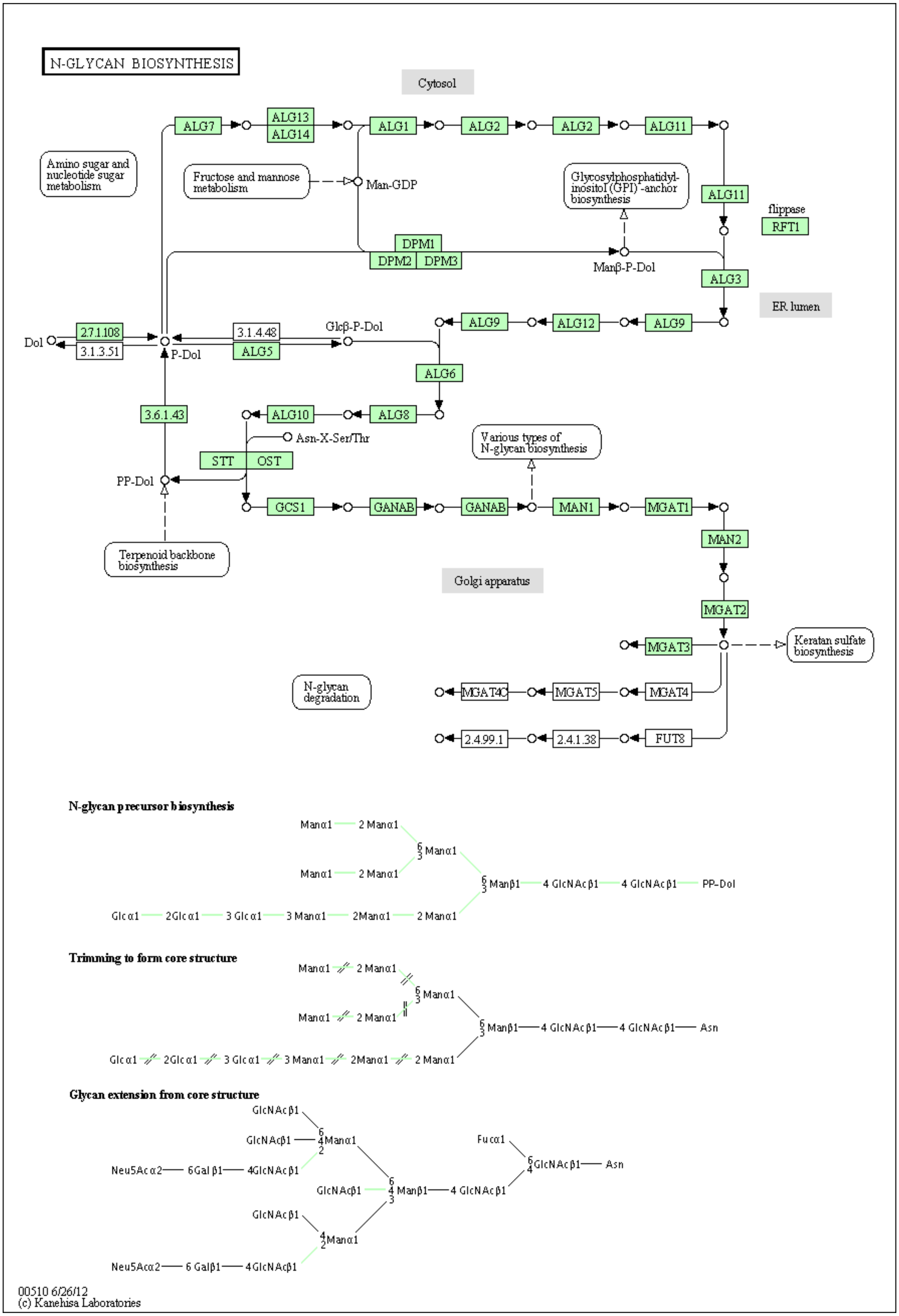
N-GLYCAN BIOSYNTHESIS
Cytosol
ALG7
ALG13
ALG14
ALG1
ALG2
ALG2
ALG11
Amino sugar and nucleotide sugar metabolism
Fructose and mannose metabolism
Man-GDP
Glycosylphosphatidyl-inositol (GPI) -anchor biosynthesis
ALG11
flippase
RFT1
DPM1
DPM2
DPM3
Manβ-P-Dol
ALG3
ER lumen
Dol
2.7.1.108
3.1.3.51
P-Dol
3.1.4.48
ALG5
Glcβ-P-Dol
ALG9
ALG12
ALG9
ALG6
3.6.1.43
ALG10
ALG8
Asn-X-Ser/Thr
STT
OST
PP-Dol
Various types of N-glycan biosynthesis
GCS1
GANAB
GANAB
MAN1
MGAT1
MAN2
Terpenoid backbone biosynthesis
Golgi apparatus
MGAT2
MGAT3
Keratan sulfate biosynthesis
N-glycan degradation
MGAT4C
MGAT5
MGAT4
2.4.99.1
2.4.1.38
FUT8
N-glycan precursor biosynthesis
Manα1 — 2 Manα1
6 3 Manα1
Manα1 — 2 Manα1
6 3 Manβ1 — 4 GlcNAcβ1 — 4 GlcNAcβ1 — PP-Dol
Glcα1 — 2Glcα1 — 3 Glcα1 — 3 Manα1 — 2Manα1 — 2 Manα1
Trimming to form core structure
Manα1 — 2 Manα1
6 3 Manα1
Manα1 — 2 Manα1
6 3 Manβ1 — 4 GlcNAcβ1 — 4 GlcNAcβ1 — Asn
Glcα1 — 2Glcα1 — 3 Glcα1 — 3 Manα1 — 2Manα1 — 2 Manα1
Glycan extension from core structure
GlcNAcβ1
GlcNAcβ1 — 6 4 2 Manα1
Neu5Acα2 — 6Galβ1 — 4GlcNAcβ1
Fucα1
6 4 GlcNAcβ1 — Asn
GlcNAcβ1 — 6 4 3 Manβ1 — 4 GlcNAcβ1
GlcNAcβ1
4 2 Manα1
Neu5Acα2 — 6 Galβ1 — 4GlcNAcβ1
00510 6/26/12
(c) Kanehisa Laboratories

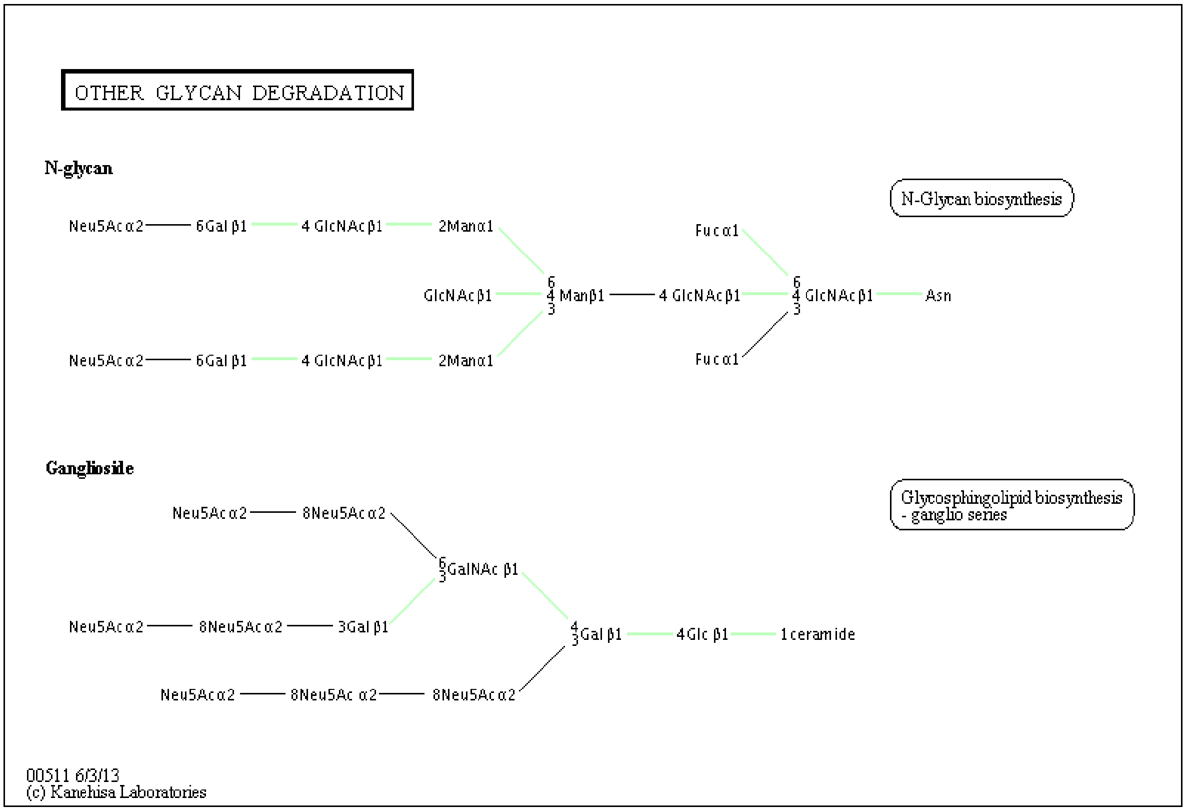

OTHER GLYCAN DEGRADATION
N-glycan
N-Glycan biosynthesis
Neu5Acα2
6Gal β1
4 GlcNAcβ1
2Manα1
GlcNAcβ1
6 4 3 Manβ1
4 GlcNAcβ1
Fucα1
6 4 3 GlcNAcβ1
Asn
Neu5Acα2
6Gal β1
4 GlcNAcβ1
2Manα1
Fucα1
Ganglioside
Glycosphingolipid biosynthesis - ganglio series
Neu5Acα2
8Neu5Acα2
6 3 GalNAc β1
Neu5Acα2
8Neu5Acα2
3Gal β1
4 3 Gal β1
4Glc β1
1ceramide
Neu5Acα2
8Neu5Ac α2
8Neu5Acα2
00511 6/3/13
(c) Kanehisa Laboratories

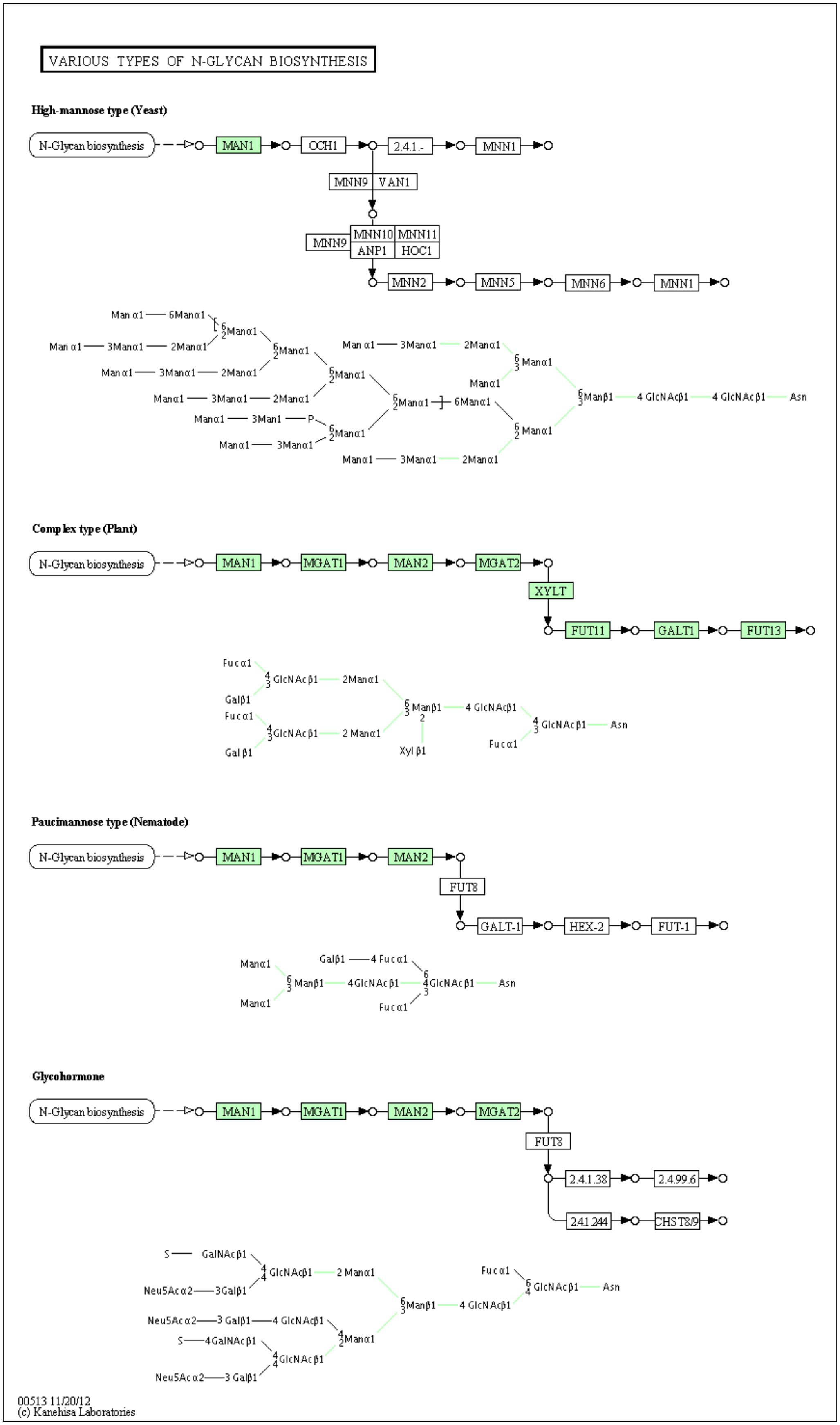

VARIOUS TYPES OF N-GLYCAN BIOSYNTHESIS
High-mannose type (Yeast)
N-Glycan biosynthesis
MAN1
OCH1
2.4.1.-
MNN1
MNN9
VAN1
MNN9
MNN10
MNN11
ANP1
HOC1
MNN2
MNN5
MNN6
MNN1
Complex type (Plant)
N-Glycan biosynthesis
MAN1
MGAT1
MAN2
MGAT2
XYLT
FUT11
GALT1
FUT13
Paucimannose type (Nematode)
N-Glycan biosynthesis
MAN1
MGAT1
MAN2
FUT8
GALT-1
HEX-2
FUT-1
Glycohormone
N-Glycan biosynthesis
MAN1
MGAT1
MAN2
MGAT2
FUT8
2.4.1.38
2.4.99.6
2.4.1.244
CHST8/9
00513 11/20/12
(c) Kanehisa Laboratories

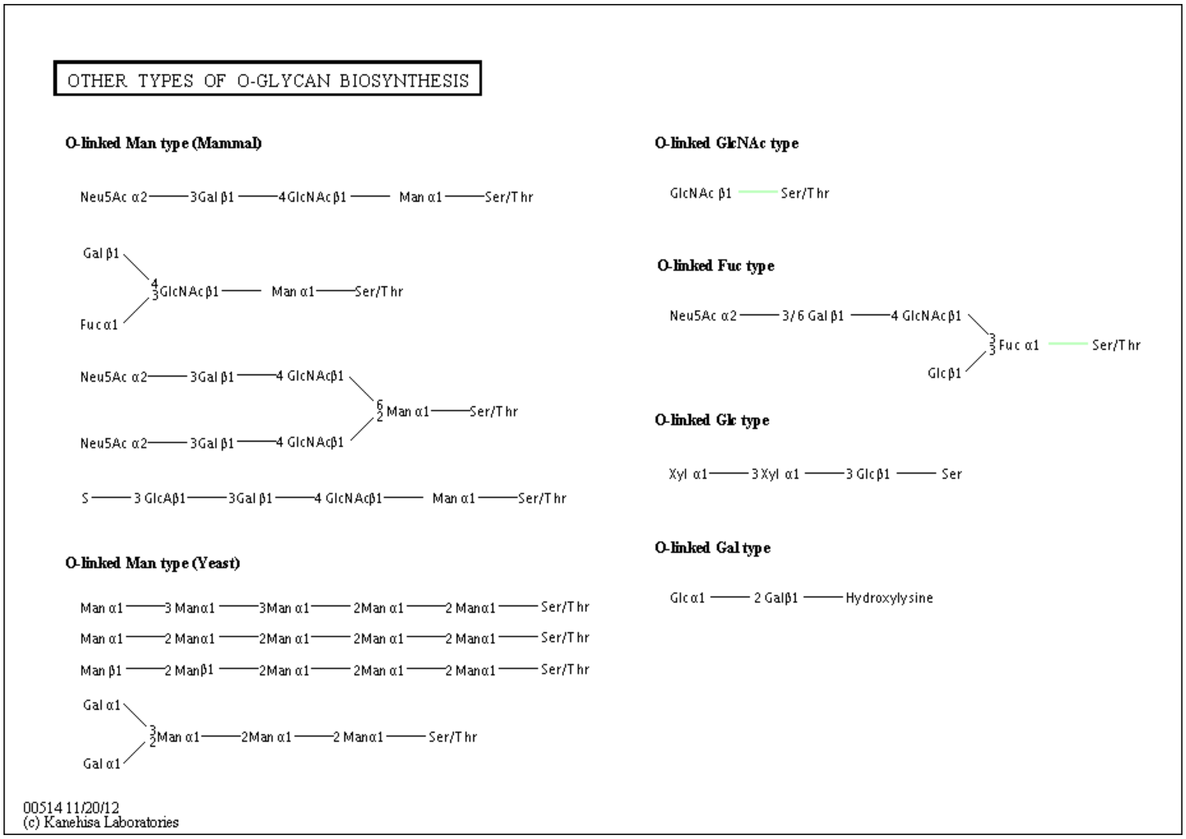
OTHER TYPES OF O-GLYCAN BIOSYNTHESIS
O-linked Man type (Mammal)
Neu5Ac α2 — 3Gal β1 — 4GlcNAc β1 — Man α1 — Ser/Thr
Gal β1
4
3 GlcNAc β1 — Man α1 — Ser/Thr
Fuc α1
Neu5Ac α2 — 3Gal β1 — 4 GlcNAc β1
6
2 Man α1 — Ser/Thr
Neu5Ac α2 — 3Gal β1 — 4 GlcNAc β1
S — 3 GlcA β1 — 3Gal β1 — 4 GlcNAc β1 — Man α1 — Ser/Thr
O-linked Man type (Yeast)
Man α1 — 3 Man α1 — 3Man α1 — 2Man α1 — 2 Man α1 — Ser/Thr
Man α1 — 2 Man α1 — 2Man α1 — 2Man α1 — 2 Man α1 — Ser/Thr
Man β1 — 2 Man β1 — 2Man α1 — 2Man α1 — 2 Man α1 — Ser/Thr
Gal α1
3
2 Man α1 — 2Man α1 — 2 Man α1 — Ser/Thr
Gal α1
O-linked GlcNAc type
GlcNAc β1 — Ser/Thr
O-linked Fuc type
Neu5Ac α2 — 3/6 Gal β1 — 4 GlcNAc β1
3
3 Fuc α1 — Ser/Thr
Glc β1
O-linked Glc type
Xyl α1 — 3 Xyl α1 — 3 Glc β1 — Ser
O-linked Gal type
Glc α1 — 2 Gal β1 — Hydroxylysine
00514 11/20/12
(c) Kanehisa Laboratories

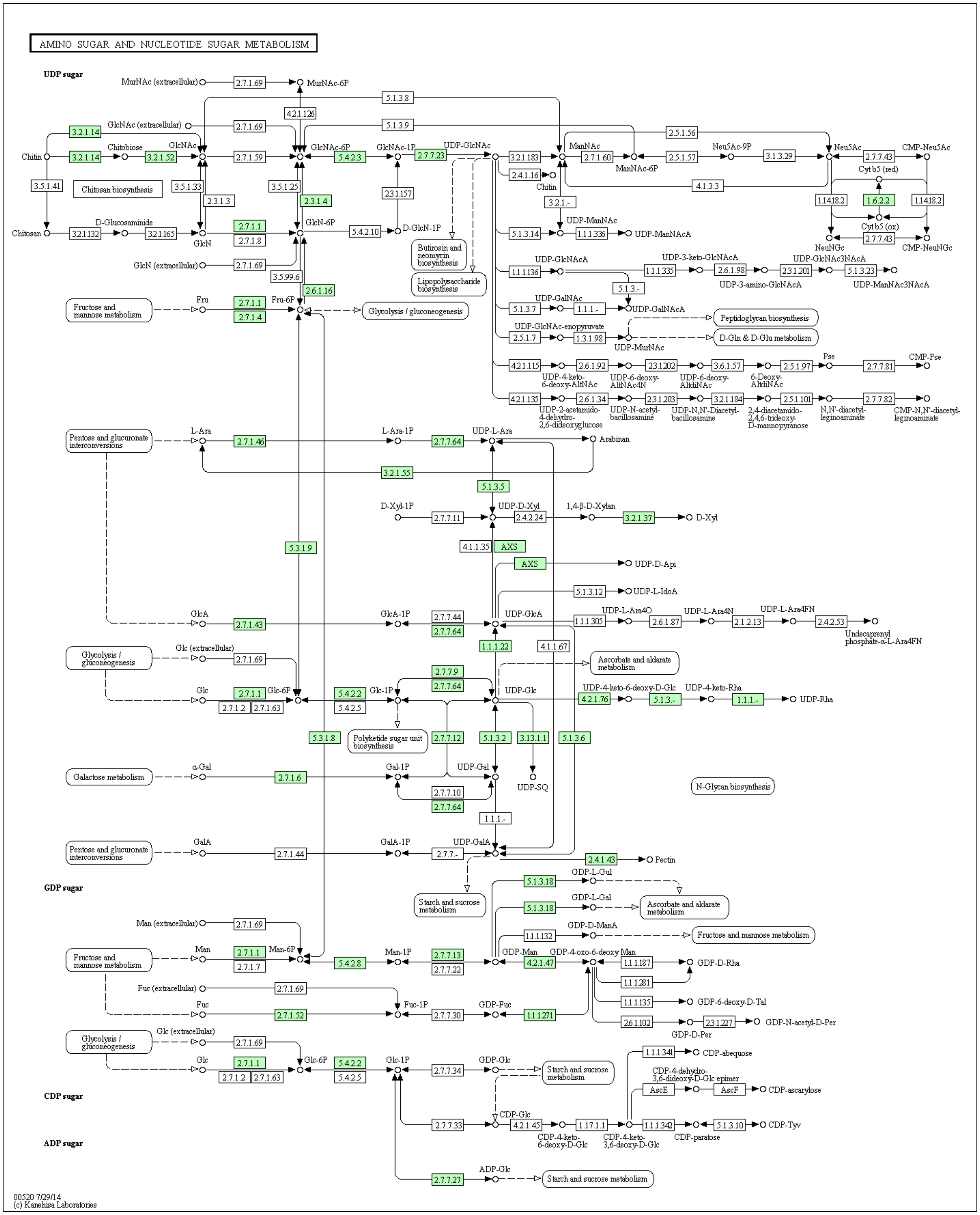

AMINO SUGAR AND NUCLEOTIDE SUGAR METABOLISM
UDP sugar
GDP sugar
CDP sugar
ADP sugar
MurNAc (extracellular)
MurNAc-6P
GlcNAc (extracellular)
Chitin
Chitobiose
GlcNAc
GlcNAc-6P
GlcNAc-1P
UDP-GlcNAc
ManNAc
ManNAc-6P
Neu5Ac-9P
Neu5Ac
CMP-Neu5Ac
Cytb5 (red)
Cytb5 (ox)
NeuNGc
CMP-NeuNGc
Chitosan biosynthesis
Chitosan
D-Glucosaminide
GlcN
GlcN-6P
D-GlcN-1P
GlcN (extracellular)
Butirosin and neomycin biosynthesis
Lipopolysaccharide biosynthesis
Chitin
UDP-ManNAc
UDP-ManNAcA
UDP-GlcNAcA
UDP-3-keto-GlcNAcA
UDP-GlcNAc3NAcA
UDP-3-amino-GlcNAcA
UDP-ManNAc3NAcA
UDP-GalNAc
UDP-GalNAcA
Peptidoglycan biosynthesis
UDP-GlcNAc-enopyruvate
UDP-MurNAc
D-Gln & D-Glu metabolism
Pse
CMP-Pse
UDP-4-keto-6-deoxy-AltNAc
UDP-6-deoxy-AltNAc4N
UDP-6-deoxy-AltdiNAc
6-Deoxy-AltdiNAc
UDP-2-acetamido-4-dehydro-2,6-dideoxyglucose
UDP-N-acetyl-bacillosamine
UDP-N,N'-Diacetyl-bacillosamine
2,4-diacetamido-2,4,6-trideoxy-D-mannopyranose
N,N'-diacetyl-legionaminate
CMP-N,N'-diacetyl-legionaminate
Fructose and mannose metabolism
Fru
Fru-6P
Glycolysis / gluconeogenesis
Pentose and glucuronate interconversions
L-Ara
L-Ara-1P
UDP-L-Ara
Arabinan
D-Xyl-1P
UDP-D-Xyl
1,4-β-D-Xylan
D-Xyl
UDP-D-Api
UDP-L-IdoA
GlcA
GlcA-1P
UDP-GlcA
UDP-L-Ara4O
UDP-L-Ara4N
UDP-L-Ara4FN
Undecaprenyl phosphate-α-L-Ara4FN
Glc (extracellular)
Ascorbate and aldarate metabolism
Glc
Glc-6P
Glc-1P
UDP-Glc
UDP-4-keto-6-deoxy-D-Glc
UDP-4-keto-Rha
UDP-Rha
Polyketide sugar unit biosynthesis
Galactose metabolism
α-Gal
Gal-1P
UDP-Gal
UDP-SQ
N-Glycan biosynthesis
GalA
GalA-1P
UDP-GalA
Pectin
Starch and sucrose metabolism
GDP-L-Gul
GDP-L-Gal
GDP-D-ManA
Man (extracellular)
Man
Man-6P
Man-1P
GDP-Man
GDP-4-oxo-6-deoxy Man
GDP-D-Rha
GDP-6-deoxy-D-Tal
Fuc (extracellular)
Fuc
Fuc-1P
GDP-Fuc
GDP-D-Per
GDP-N-acetyl-D-Per
GDP-Glc
CDP-abequose
CDP-4-dehydro-3,6-dideoxy-D-Glc epimer
AscE
AscF
CDP-ascarylose
CDP-Glc
CDP-4-keto-6-deoxy-D-Glc
CDP-4-keto-3,6-deoxy-D-Glc
CDP-paratose
CDP-Tyv
ADP-Glc
00520 7/29/14
(c) Kanehisa Laboratories

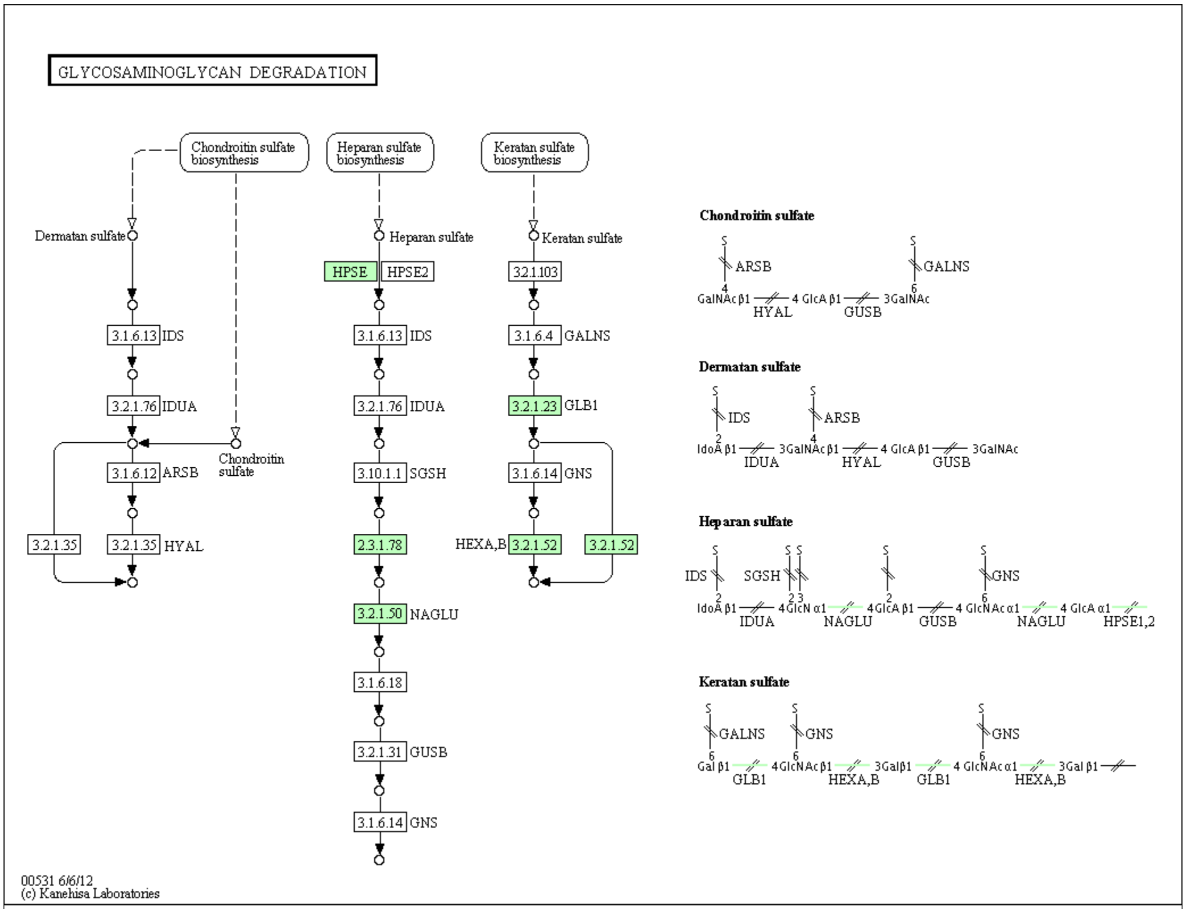
GLYCOSAMINOGLYCAN DEGRADATION
Chondroitin sulfate biosynthesis
Heparan sulfate biosynthesis
Keratan sulfate biosynthesis
Dermatan sulfate
Heparan sulfate
Keratan sulfate
HPSE
HPSE2
3.2.1.103
3.1.6.13 IDS
3.1.6.4 GALNS
3.2.1.76 IDUA
3.2.1.23 GLB1
Chondroitin sulfate
3.1.6.12 ARSB
3.10.1.1 SGSH
3.1.6.14 GNS
3.2.1.35
3.2.1.35 HYAL
2.3.1.78
HEXA,B 3.2.1.52
3.2.1.52
3.2.1.50 NAGLU
3.1.6.18
3.2.1.31 GUSB
3.1.6.14 GNS
Chondroitin sulfate
ARSB
GALNS
GalNAc β1 — 4 GlcA β1 — 3GalNAc
HYAL
GUSB
Dermatan sulfate
IDS
ARSB
IdoA β1 — 3GalNAc β1 — 4 GlcA β1 — 3GalNAc
IDUA
HYAL
GUSB
Heparan sulfate
IDS
SGSH
GNS
IdoA β1 — 4GlcN α1 — 4GlcA β1 — 4 GlcNAc α1 — 4 GlcA α1
IDUA
NAGLU
GUSB
NAGLU
HPSE1,2
Keratan sulfate
GALNS
GNS
GNS
Gal β1 — 4GlcNAc β1 — 3Gal β1 — 4 GlcNAc α1 — 3Gal β1
GLB1
HEXA,B
GLB1
HEXA,B
00531 6/6/12
(c) Kanehisa Laboratories

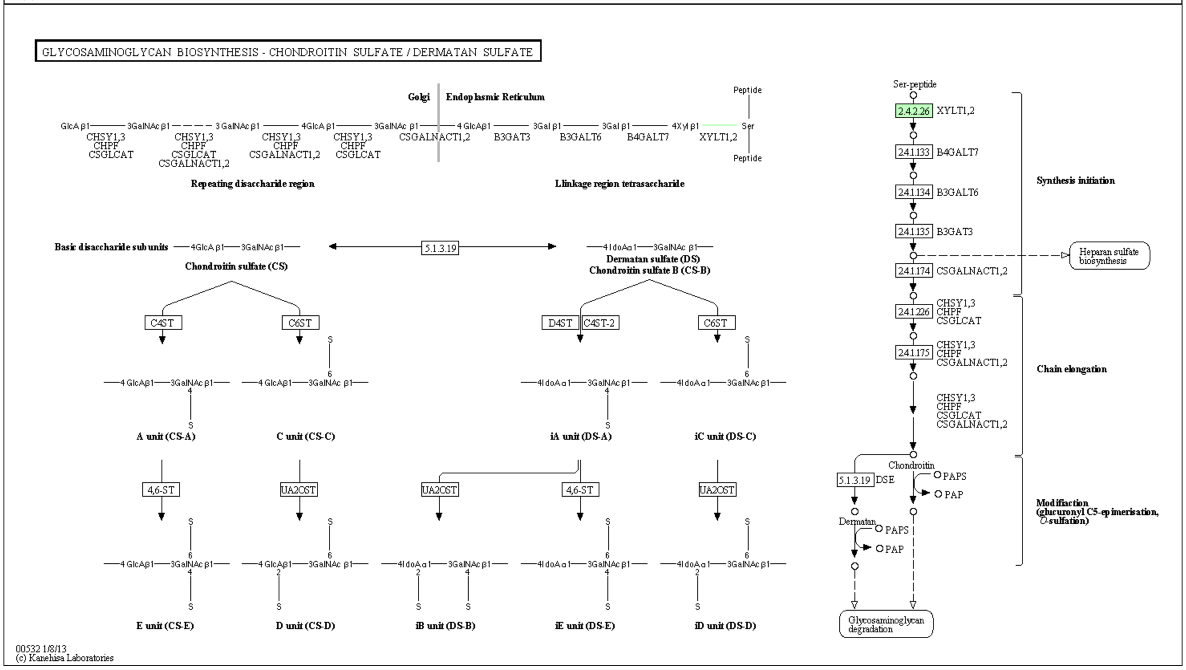
GLYCOSAMINOGLYCAN BIOSYNTHESIS - CHONDROITIN SULFATE / DERMATAN SULFATE
Golgi
Endoplasmic Reticulum
Peptide
Ser
CHSY1,3
CHPF
CSGLCAT
CSGALNACT1,2
B3GAT3
B3GALT6
B4GALT7
XYLT1,2
Repeating disaccharide region
Llinkage region tetrasaccharide
Basic disaccharide subunits
Chondroitin sulfate (CS)
5.1.3.19
Dermatan sulfate (DS)
Chondroitin sulfate B (CS-B)
C4ST
C6ST
D4ST
C4ST-2
A unit (CS-A)
C unit (CS-C)
iA unit (DS-A)
iC unit (DS-C)
4,6-ST
UA2OST
E unit (CS-E)
D unit (CS-D)
iB unit (DS-B)
iE unit (DS-E)
iD unit (DS-D)
Ser-peptide
2.4.2.26 XYLT1,2
2.4.1.133 B4GALT7
2.4.1.134 B3GALT6
2.4.1.135 B3GAT3
2.4.1.174 CSGALNACT1,2
2.4.1.226
2.4.1.175
Heparan sulfate biosynthesis
Synthesis initiation
Chain elongation
Chondroitin
5.1.3.19 DSE
Dermatan
PAPS
PAP
Modifiaction (glucuronyl C5-epimerisation, O-sulfation)
Glycosaminoglycan degradation
00532 1/8/13
(c) Kanehisa Laboratories

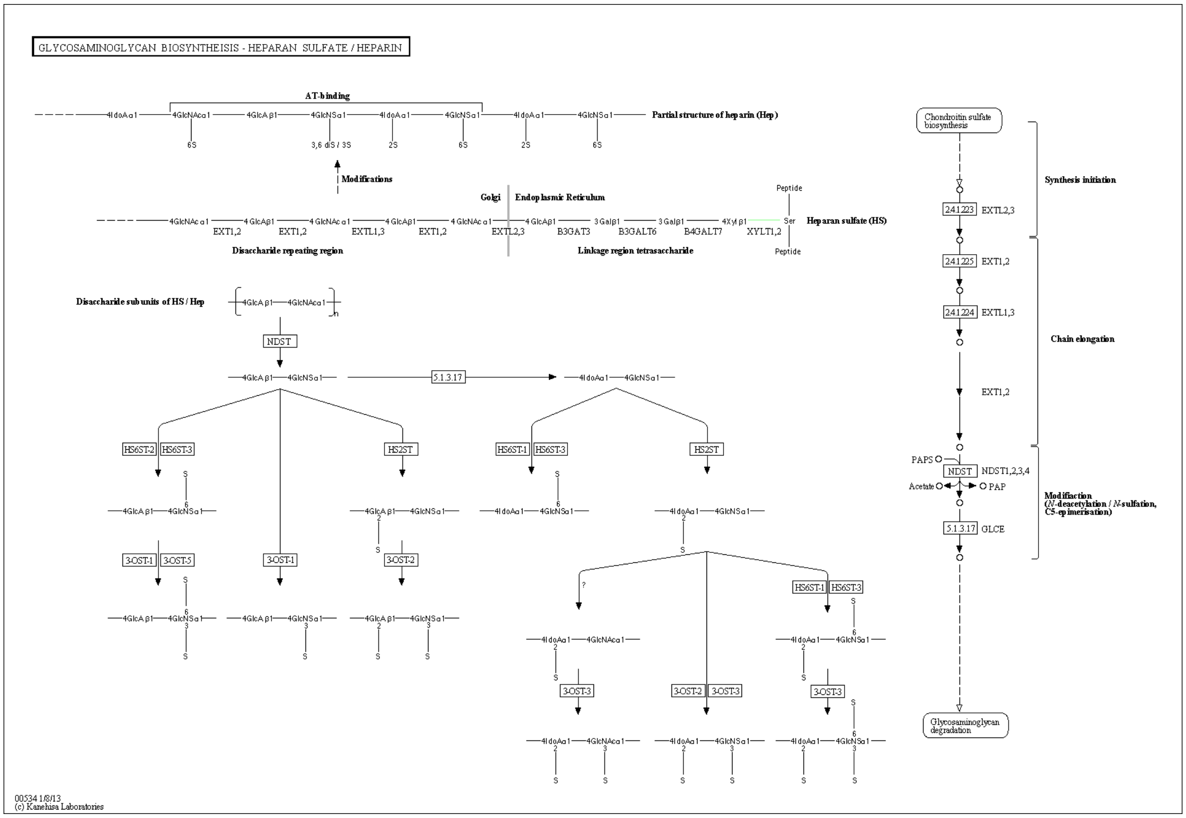

GLYCOSAMINOGLYCAN BIOSYNTHEISIS - HEPARAN SULFATE / HEPARIN
AT-binding
Partial structure of heparin (Hep)
Modifications
Golgi
Endoplasmic Reticulum
Heparan sulfate (HS)
Peptide
Ser
EXT1,2
EXTL1,3
EXTL2,3
B3GAT3
B3GALT6
B4GALT7
XYLT1,2
Disaccharide repeating region
Linkage region tetrasaccharide
Disaccharide subunits of HS / Hep
NDST
5.1.3.17
HS6ST-1
HS6ST-2
HS6ST-3
HS2ST
3-OST-1
3-OST-2
3-OST-3
3-OST-5
Chondroitin sulfate biosynthesis
Synthesis initiation
2.4.1.223
EXTL2,3
Chain elongation
2.4.1.225
2.4.1.224
PAPS
PAP
Acetate
NDST1,2,3,4
GLCE
Modifiaction (N-deacetylation / N-sulfation, C5-epimerisation)
Glycosaminoglycan degradation
00534 1/8/13
(c) Kanehisa Laboratories

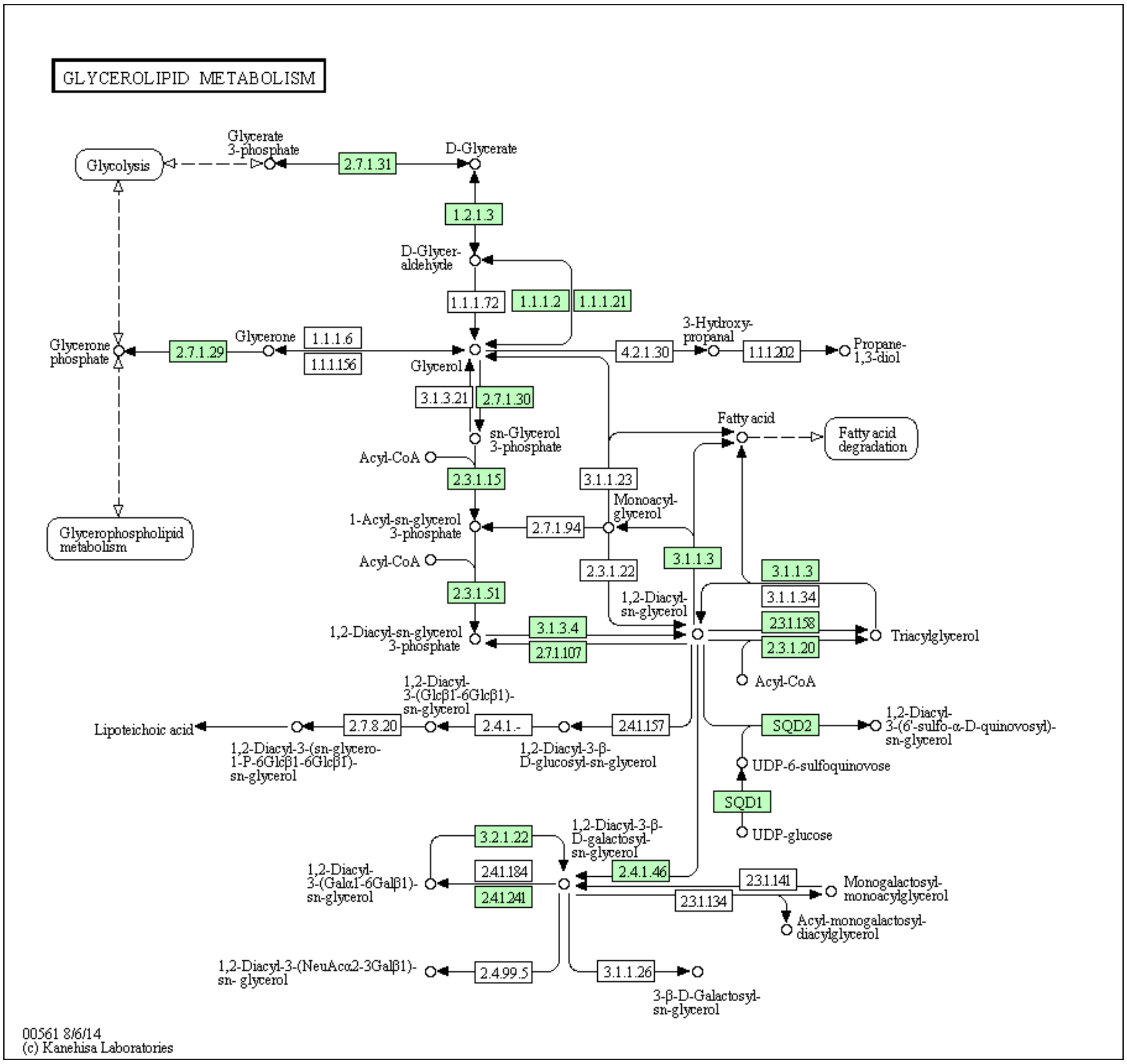

GLYCEROLIPID METABOLISM
Glycolysis
Glycerate 3-phosphate
2.7.1.31
D-Glycerate
1.2.1.3
D-Glycer-aldehyde
1.1.1.72
1.1.1.2
1.1.1.21
Glycerone phosphate
2.7.1.29
Glycerone
1.1.1.6
1.1.1.156
Glycerol
4.2.1.30
3-Hydroxy-propanal
1.1.1.202
Propane-1,3-diol
3.1.3.21
2.7.1.30
sn-Glycerol 3-phosphate
Fatty acid
Fatty acid degradation
Acyl-CoA
2.3.1.15
3.1.1.23
Monoacyl-glycerol
1-Acyl-sn-glycerol 3-phosphate
2.7.1.94
Glycerophospholipid metabolism
3.1.1.3
3.1.1.3
Acyl-CoA
2.3.1.22
3.1.1.34
2.3.1.51
1,2-Diacyl-sn-glycerol
2.3.1.158
1,2-Diacyl-sn-glycerol 3-phosphate
3.1.3.4
2.7.1.107
2.3.1.20
Triacylglycerol
Acyl-CoA
1,2-Diacyl-3-(Glcβ1-6Glcβ1)-sn-glycerol
Lipoteichoic acid
2.7.8.20
2.4.1.-
2.4.1.157
SQD2
1,2-Diacyl-3-(6'-sulfo-α-D-quinovosyl)-sn-glycerol
1,2-Diacyl-3-(sn-glycero-1-P-6Glcβ1-6Glcβ1)-sn-glycerol
1,2-Diacyl-3-β-D-glucosyl-sn-glycerol
UDP-6-sulfoquinovose
SQD1
UDP-glucose
3.2.1.22
1,2-Diacyl-3-β-D-galactosyl-sn-glycerol
1,2-Diacyl-3-(Galα1-6Galβ1)-sn-glycerol
2.4.1.184
2.4.1.241
2.4.1.46
2.3.1.141
Monogalactosyl-monoacylglycerol
2.3.1.134
Acyl-monogalactosyl-diacylglycerol
1,2-Diacyl-3-(NeuAcα2-3Galβ1)-sn- glycerol
2.4.99.5
3.1.1.26
3-β-D-Galactosyl-sn-glycerol
00561 8/6/14
(c) Kanehisa Laboratories

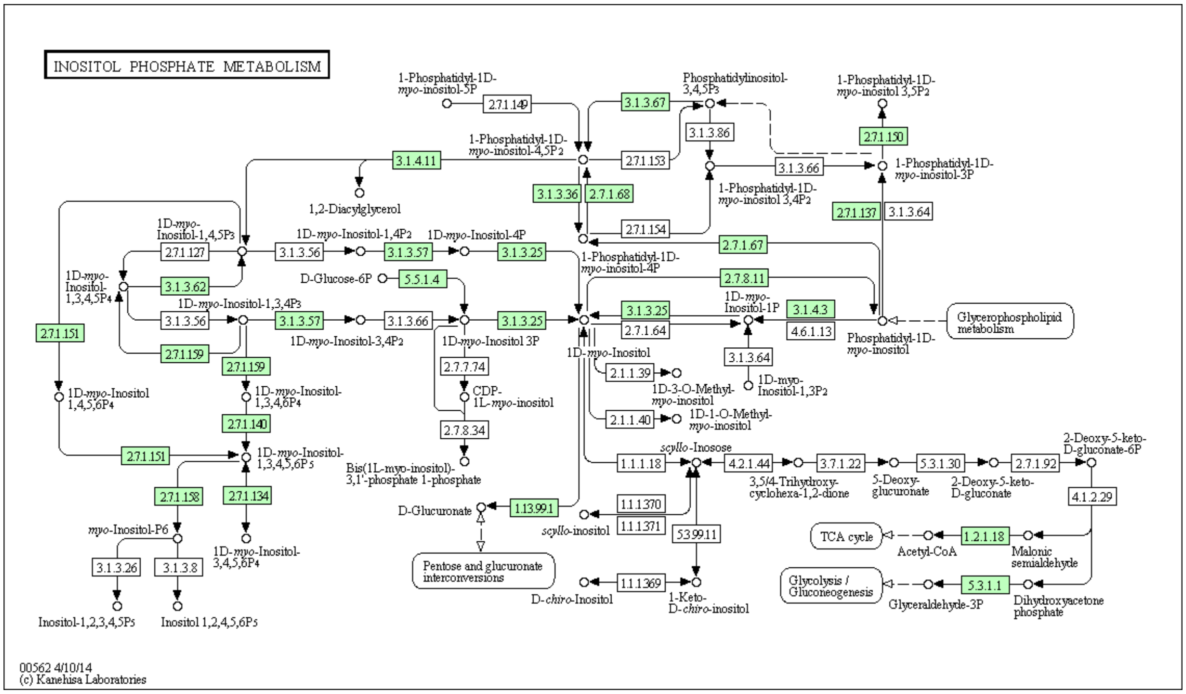
INOSITOL PHOSPHATE METABOLISM
1-Phosphatidyl-1D-myo-inositol-5P
2.7.1.149
3.1.3.67
Phosphatidylinositol-3,4,5P3
1-Phosphatidyl-1D-myo-inositol 3,5P2
2.7.1.150
3.1.3.86
1-Phosphatidyl-1D-myo-inositol-4,5P2
3.1.4.11
2.7.1.153
3.1.3.66
1-Phosphatidyl-1D-myo-inositol-3P
1,2-Diacylglycerol
3.1.3.36
2.7.1.68
1-Phosphatidyl-1D-myo-inositol 3,4P2
2.7.1.137
3.1.3.64
1D-myo-Inositol-1,4,5P3
2.7.1.127
1D-myo-Inositol-1,4P2
3.1.3.56
3.1.3.57
1D-myo-Inositol-4P
3.1.3.25
2.7.1.154
2.7.1.67
1-Phosphatidyl-1D-myo-inositol-4P
1D-myo-Inositol-1,3,4,5P4
3.1.3.62
D-Glucose-6P
5.5.1.4
2.7.8.11
1D-myo-Inositol-1,3,4P3
1D-myo-Inositol-1P
3.1.4.3
Glycerophospholipid metabolism
2.7.1.151
3.1.3.56
3.1.3.57
3.1.3.66
3.1.3.25
3.1.3.25
2.7.1.64
4.6.1.13
1D-myo-Inositol-3,4P2
1D-myo-Inositol 3P
Phosphatidyl-1D-myo-inositol
2.7.1.159
1D-myo-Inositol
2.7.1.159
2.7.7.74
3.1.3.64
2.1.1.39
1D-3-O-Methyl-myo-inositol
1D-myo-Inositol-1,3P2
1D-myo-Inositol 1,4,5,6P4
1D-myo-Inositol-1,3,4,6P4
CDP-1L-myo-inositol
2.7.1.140
2.7.8.34
2.1.1.40
1D-1-O-Methyl-myo-inositol
2-Deoxy-5-keto-D-gluconate-6P
2.7.1.151
1D-myo-Inositol-1,3,4,5,6P5
scyllo-Inosose
1.1.1.18
4.2.1.44
3.7.1.22
5.3.1.30
2.7.1.92
Bis(1L-myo-inositol)-3,1'-phosphate 1-phosphate
3,5/4-Trihydroxy-cyclohexa-1,2-dione
5-Deoxy-glucuronate
2-Deoxy-5-keto-D-gluconate
2.7.1.158
2.7.1.134
4.1.2.29
D-Glucuronate
1.13.99.1
1.1.1.370
scyllo-inositol
1.1.1.371
53.99.11
TCA cycle
Acetyl-CoA
1.2.1.18
Malonic semialdehyde
myo-Inositol-P6
1D-myo-Inositol-3,4,5,6P4
3.1.3.26
3.1.3.8
Pentose and glucuronate interconversions
Glycolysis / Gluconeogenesis
5.3.1.1
D-chiro-Inositol
1.1.1.369
1-Keto-D-chiro-inositol
Glyceraldehyde-3P
Dihydroxyacetone phosphate
Inositol-1,2,3,4,5P5
Inositol 1,2,4,5,6P5
00562 4/10/14
(c) Kanehisa Laboratories

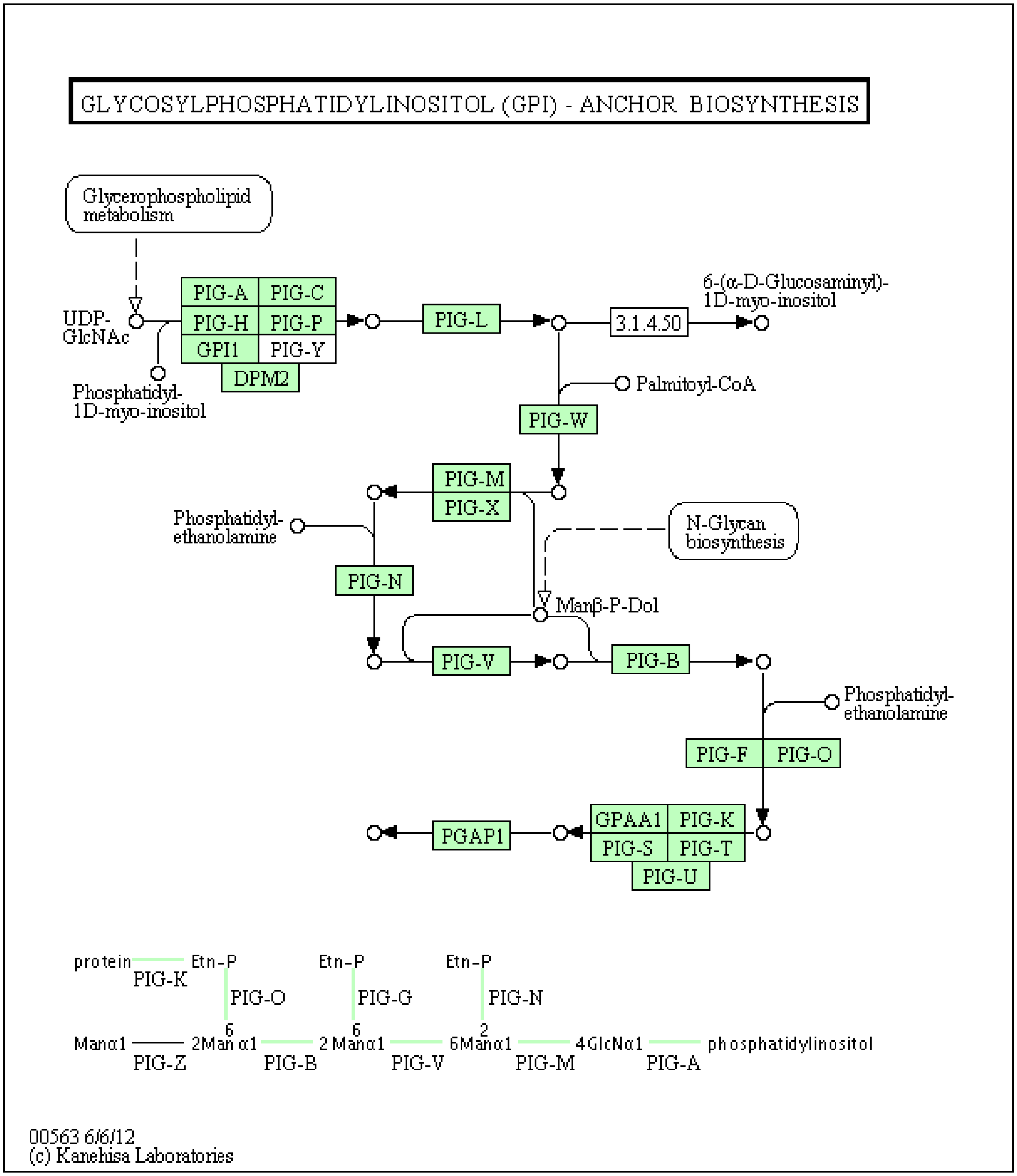
GLYCOSYLPHOSPHATIDYLINOSITOL (GPI) - ANCHOR BIOSYNTHESIS
Glycerophospholipid metabolism
UDP-GlcNAc
PIG-A
PIG-C
PIG-H
PIG-P
GPI1
PIG-Y
DPM2
Phosphatidyl-1D-myo-inositol
PIG-L
3.1.4.50
6-(α-D-Glucosaminyl)-1D-myo-inositol
Palmitoyl-CoA
PIG-W
PIG-M
PIG-X
Phosphatidyl-ethanolamine
N-Glycan biosynthesis
PIG-N
Manβ-P-Dol
PIG-V
PIG-B
Phosphatidyl-ethanolamine
PIG-F
PIG-O
GPAA1
PIG-K
PIG-S
PIG-T
PIG-U
PGAP1
protein
PIG-K
Etn-P
PIG-O
Etn-P
PIG-G
Etn-P
PIG-N
Manα1
2Manα1
2 Manα1
6Manα1
4GlcNα1
phosphatidylinositol
PIG-Z
PIG-B
PIG-V
PIG-M
PIG-A
00563 6/6/12
(c) Kanehisa Laboratories

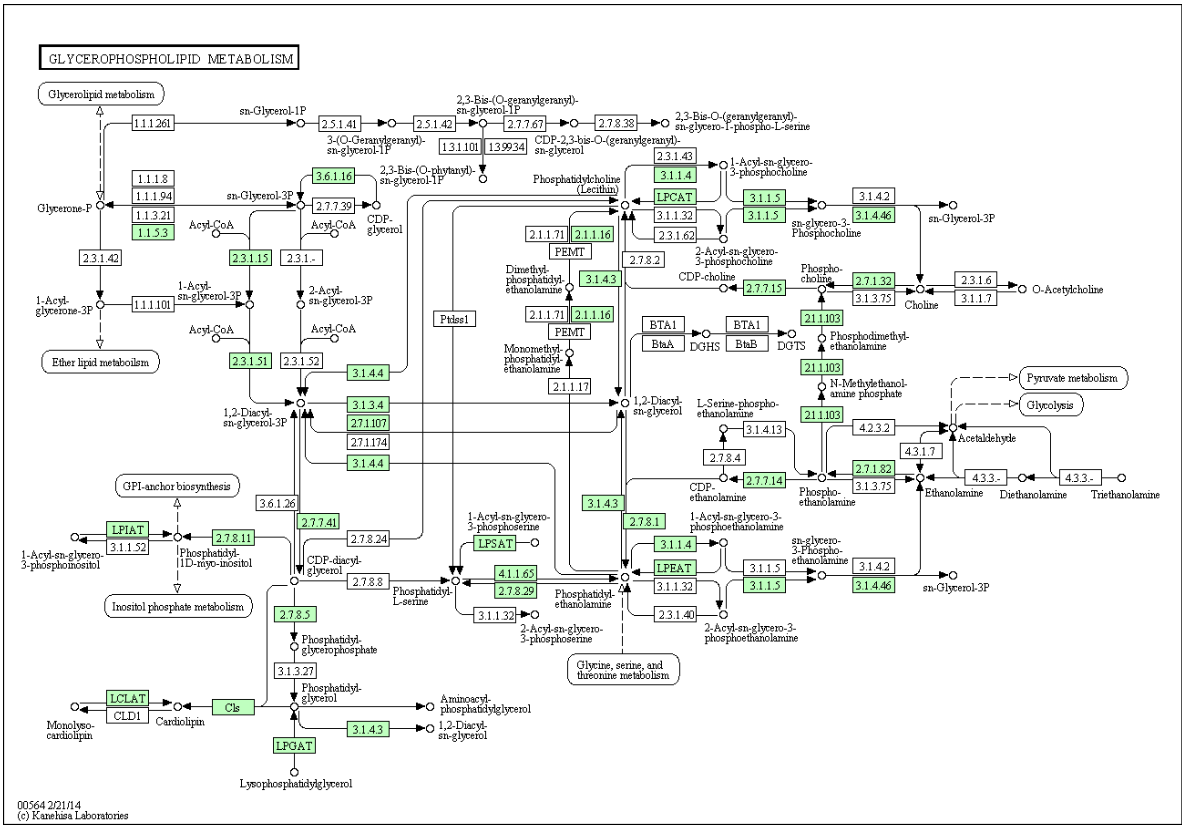

GLYCEROPHOSPHOLIPID METABOLISM
Glycerolipid metabolism
sn-Glycerol-1P
2.5.1.41
2.5.1.42
2,3-Bis-(O-geranylgeranyl)-sn-glycerol-1P
2.7.7.67
2.7.8.38
2,3-Bis-O-(geranylgeranyl)-sn-glycero-1-phospho-L-serine
1.1.1.261
3-(O-Geranylgeranyl)-sn-glycerol-1P
1.3.1.101
1.3.99.34
CDP-2,3-bis-O-(geranylgeranyl)-sn-glycerol
3.6.1.16
2,3-Bis-(O-phytanyl)-sn-glycerol-1P
1.1.1.8
1.1.1.94
1.1.3.21
1.1.5.3
Glycerone-P
sn-Glycerol-3P
2.7.7.39
CDP-glycerol
Acyl-CoA
2.3.1.15
2.3.1.-
2.3.1.42
1-Acyl-glycerone-3P
1.1.1.101
1-Acyl-sn-glycerol-3P
2-Acyl-sn-glycerol-3P
2.3.1.51
2.3.1.52
Ether lipid metabolism
3.1.4.4
1,2-Diacyl-sn-glycerol-3P
3.1.3.4
2.7.1.107
2.7.1.174
GPI-anchor biosynthesis
3.6.1.26
2.7.7.41
LPIAT
3.1.1.52
2.7.8.11
1-Acyl-sn-glycero-3-phosphoinositol
Phosphatidyl-1D-myo-inositol
Inositol phosphate metabolism
CDP-diacylglycerol
2.7.8.24
2.7.8.8
2.7.8.5
Phosphatidylglycerophosphate
3.1.3.27
Phosphatidylglycerol
LCLAT
CLD1
Cls
Monolysocardiolipin
Cardiolipin
LPGAT
Lysophosphatidylglycerol
3.1.4.3
Aminoacyl-phosphatidylglycerol
1,2-Diacyl-sn-glycerol
Ptdss1
Phosphatidylcholine (Lecithin)
2.3.1.43
3.1.1.4
LPCAT
3.1.1.32
3.1.1.5
1-Acyl-sn-glycero-3-phosphocholine
2-Acyl-sn-glycero-3-phosphocholine
3.1.4.2
3.1.4.46
sn-glycero-3-Phosphocholine
sn-Glycerol-3P
2.1.1.71
2.1.1.16
PEMT
3.1.1.62
2.7.8.2
Dimethyl-phosphatidyl-ethanolamine
Monomethyl-phosphatidyl-ethanolamine
2.1.1.17
CDP-choline
2.7.7.15
Phosphocholine
2.7.1.32
3.1.3.75
Choline
2.3.1.6
3.1.1.7
O-Acetylcholine
BTA1
BtaA
BtaB
DGHS
DGTS
2.1.1.103
Phosphodimethyl-ethanolamine
N-Methylethanolamine phosphate
Pyruvate metabolism
Glycolysis
L-Serine-phospho-ethanolamine
3.1.4.13
4.2.3.2
Acetaldehyde
2.7.8.4
4.3.1.7
CDP-ethanolamine
2.7.7.14
Phospho-ethanolamine
2.7.1.82
4.3.3.-
Ethanolamine
Diethanolamine
Triethanolamine
2.7.8.1
1-Acyl-sn-glycero-3-phosphoserine
LPSAT
4.1.1.65
2.7.8.29
Phosphatidyl-L-serine
Phosphatidyl-ethanolamine
LPEAT
1-Acyl-sn-glycero-3-phosphoethanolamine
2-Acyl-sn-glycero-3-phosphoserine
2.3.1.40
2-Acyl-sn-glycero-3-phosphoethanolamine
sn-glycero-3-Phospho-ethanolamine
Glycine, serine, and threonine metabolism
00564 2/21/14
(c) Kanehisa Laboratories

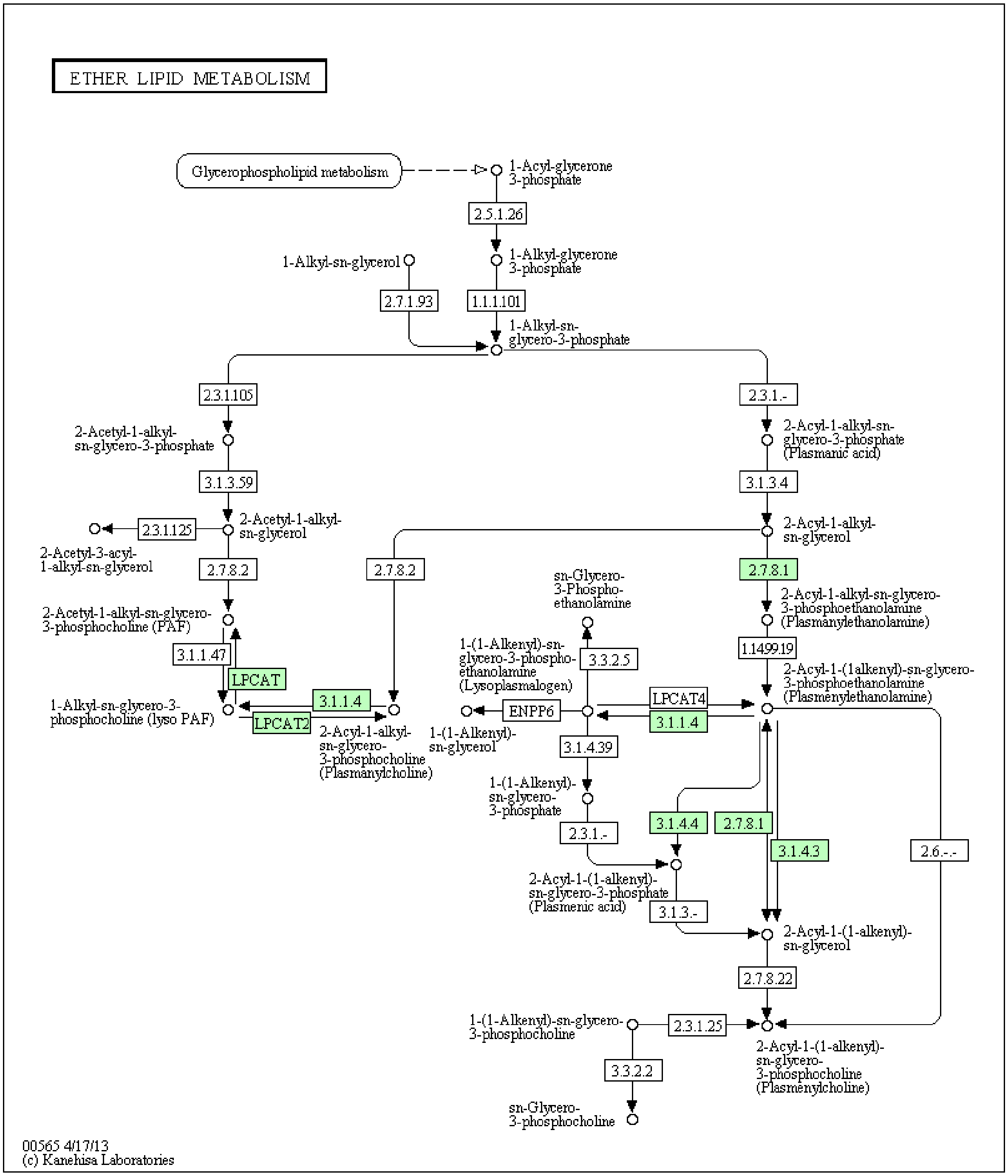
ETHER LIPID METABOLISM
Glycerophospholipid metabolism
1-Acyl-glycerone 3-phosphate
2.5.1.26
1-Alkyl-glycerone 3-phosphate
1-Alkyl-sn-glycerol
2.7.1.93
1.1.1.101
1-Alkyl-sn-glycero-3-phosphate
2.3.1.105
2.3.1.-
2-Acetyl-1-alkyl-sn-glycero-3-phosphate
2-Acyl-1-alkyl-sn-glycero-3-phosphate (Plasmanic acid)
3.1.3.59
3.1.3.4
2.3.1.125
2-Acetyl-1-alkyl-sn-glycerol
2-Acyl-1-alkyl-sn-glycerol
2-Acetyl-3-acyl-1-alkyl-sn-glycerol
2.7.8.2
2.7.8.2
2.7.8.1
sn-Glycero-3-Phospho-ethanolamine
2-Acyl-1-alkyl-sn-glycero-3-phosphoethanolamine (Plasmanylethanolamine)
2-Acetyl-1-alkyl-sn-glycero-3-phosphocholine (PAF)
1-(1-Alkenyl)-sn-glycero-3-phospho-ethanolamine (Lysoplasmalogen)
3.3.2.5
1.14.99.19
3.1.1.47
LPCAT
2-Acyl-1-(1alkenyl)-sn-glycero-3-phosphoethanolamine (Plasmenylethanolamine)
3.1.1.4
LPCAT4
1-Alkyl-sn-glycero-3-phosphocholine (lyso PAF)
ENPP6
3.1.1.4
LPCAT2
2-Acyl-1-alkyl-sn-glycero-3-phosphocholine (Plasmanylcholine)
1-(1-Alkenyl)-sn-glycerol
3.1.4.39
1-(1-Alkenyl)-sn-glycero-3-phosphate
3.1.4.4
2.7.8.1
3.1.4.3
2.6.-.-
2.3.1.-
2-Acyl-1-(1-alkenyl)-sn-glycero-3-phosphate (Plasmenic acid)
3.1.3.-
2-Acyl-1-(1-alkenyl)-sn-glycerol
2.7.8.22
1-(1-Alkenyl)-sn-glycero-3-phosphocholine
2.3.1.25
2-Acyl-1-(1-alkenyl)-sn-glycero-3-phosphocholine (Plasmenylcholine)
3.3.2.2
sn-Glycero-3-phosphocholine
00565 4/17/13
(c) Kanehisa Laboratories

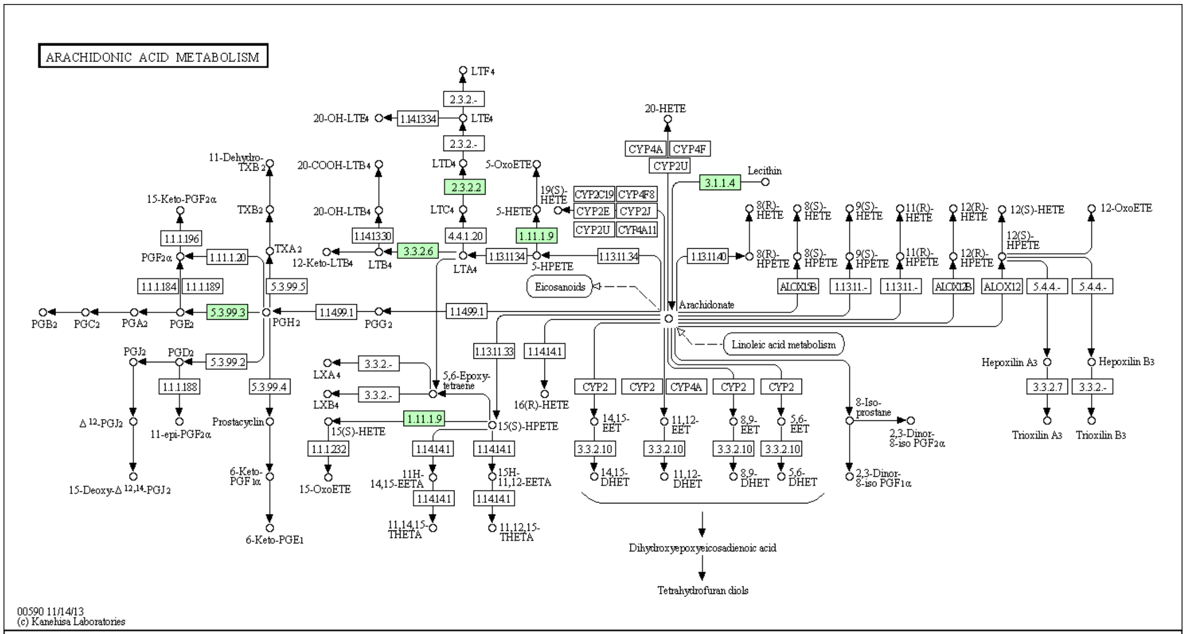
ARACHIDONIC ACID METABOLISM
LTF4
2.3.2.-
20-OH-LTE4
1.14.13.34
LTE4
2.3.2.-
20-HETE
CYP4A
CYP4F
CYP2U
11-Dehydro-TXB2
20-COOH-LTB4
LTD4
5-OxoETE
3.1.1.4
Lecithin
2.3.2.2
15-Keto-PGF2α
TXB2
20-OH-LTB4
LTC4
5-HETE
19(S)-HETE
CYP2C19
CYP4F8
CYP2E
CYP2J
CYP2U
CYP4A11
8(R)-HETE
8(S)-HETE
9(S)-HETE
11(R)-HETE
12(R)-HETE
12(S)-HETE
12-OxoETE
1.1.1.196
1.14.13.30
4.4.1.20
1.11.1.9
TXA2
3.3.2.6
12(S)-HPETE
PGF2α
1.1.1.20
12-Keto-LTB4
LTB4
LTA4
1.13.11.34
5-HPETE
1.13.11.34
1.13.11.40
8(R)-HPETE
8(S)-HPETE
9(S)-HPETE
11(R)-HPETE
12(R)-HPETE
1.1.1.184
1.1.1.189
5.3.99.5
Eicosanoids
ALOX15B
1.13.11.-
1.13.11.-
ALOX12B
ALOX12
5.4.4.-
5.4.4.-
PGB2
PGC2
PGA2
PGE2
5.3.99.3
PGH2
1.14.99.1
PGG2
1.14.99.1
Arachidonate
PGJ2
PGD2
5.3.99.2
1.13.11.33
1.14.14.1
Linoleic acid metabolism
LXA4
3.3.2.-
5,6-Epoxy-tetraene
Hepoxilin A3
Hepoxilin B3
1.1.1.188
5.3.99.4
CYP2
CYP2
CYP4A
CYP2
CYP2
LXB4
3.3.2.-
16(R)-HETE
8-Iso-prostane
3.3.2.7
3.3.2.-
Δ12-PGJ2
11-epi-PGF2α
Prostacyclin
15(S)-HETE
1.11.1.9
15(S)-HPETE
14,15-EET
11,12-EET
8,9-EET
5,6-EET
2,3-Dinor-8-iso PGF2α
Trioxilin A3
Trioxilin B3
1.1.1.232
1.14.14.1
1.14.14.1
3.3.2.10
3.3.2.10
3.3.2.10
3.3.2.10
6-Keto-PGF1α
15-Deoxy-Δ12,14-PGJ2
15-OxoETE
11H-14,15-EETA
15H-11,12-EETA
14,15-DHET
11,12-DHET
8,9-DHET
5,6-DHET
2,3-Dinor-8-iso PGF1α
1.14.14.1
1.14.14.1
11,14,15-THETA
11,12,15-THETA
6-Keto-PGE1
Dihydroxyepoxyeicosadienoic acid
Tetrahydrofuran diols
00590 11/14/13
(c) Kanehisa Laboratories

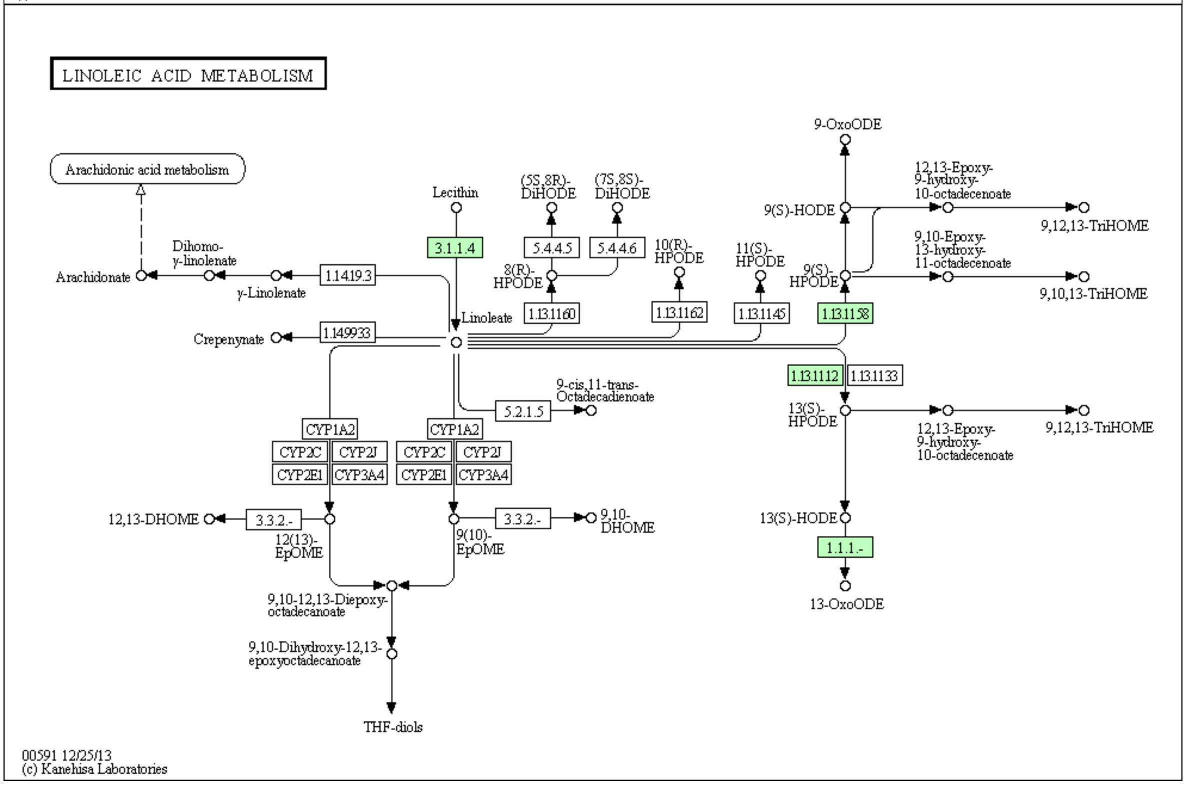
LINOLEIC ACID METABOLISM
9-OxoODE
Arachidonic acid metabolism
12,13-Epoxy-9-hydroxy-10-octadecenoate
Lecithin
(5S,8R)-DiHODE
(7S,8S)-DiHODE
9(S)-HODE
9,12,13-TriHOME
Dihomo-γ-linolenate
3.1.1.4
5.4.4.5
5.4.4.6
10(R)-HPODE
11(S)-HPODE
9,10-Epoxy-13-hydroxy-11-octadecenoate
Arachidonate
γ-Linolenate
1.14.19.3
8(R)-HPODE
9(S)-HPODE
9,10,13-TriHOME
Linoleate
1.13.11.60
1.13.11.62
1.13.11.45
1.13.11.58
Crepenynate
1.14.99.33
1.13.11.12
1.13.11.33
9-cis,11-trans-Octadecadienoate
5.2.1.5
13(S)-HPODE
CYP1A2
CYP1A2
12,13-Epoxy-9-hydroxy-10-octadecenoate
9,12,13-TriHOME
CYP2C
CYP2J
CYP2C
CYP2J
CYP2E1
CYP3A4
CYP2E1
CYP3A4
12,13-DHOME
3.3.2.-
3.3.2.-
9,10-DHOME
13(S)-HODE
12(13)-EpOME
9(10)-EpOME
1.1.1.-
9,10-12,13-Diepoxy-octadecanoate
13-OxoODE
9,10-Dihydroxy-12,13-epoxyoctadecanoate
THF-diols
00591 12/25/13
(c) Kanehisa Laboratories

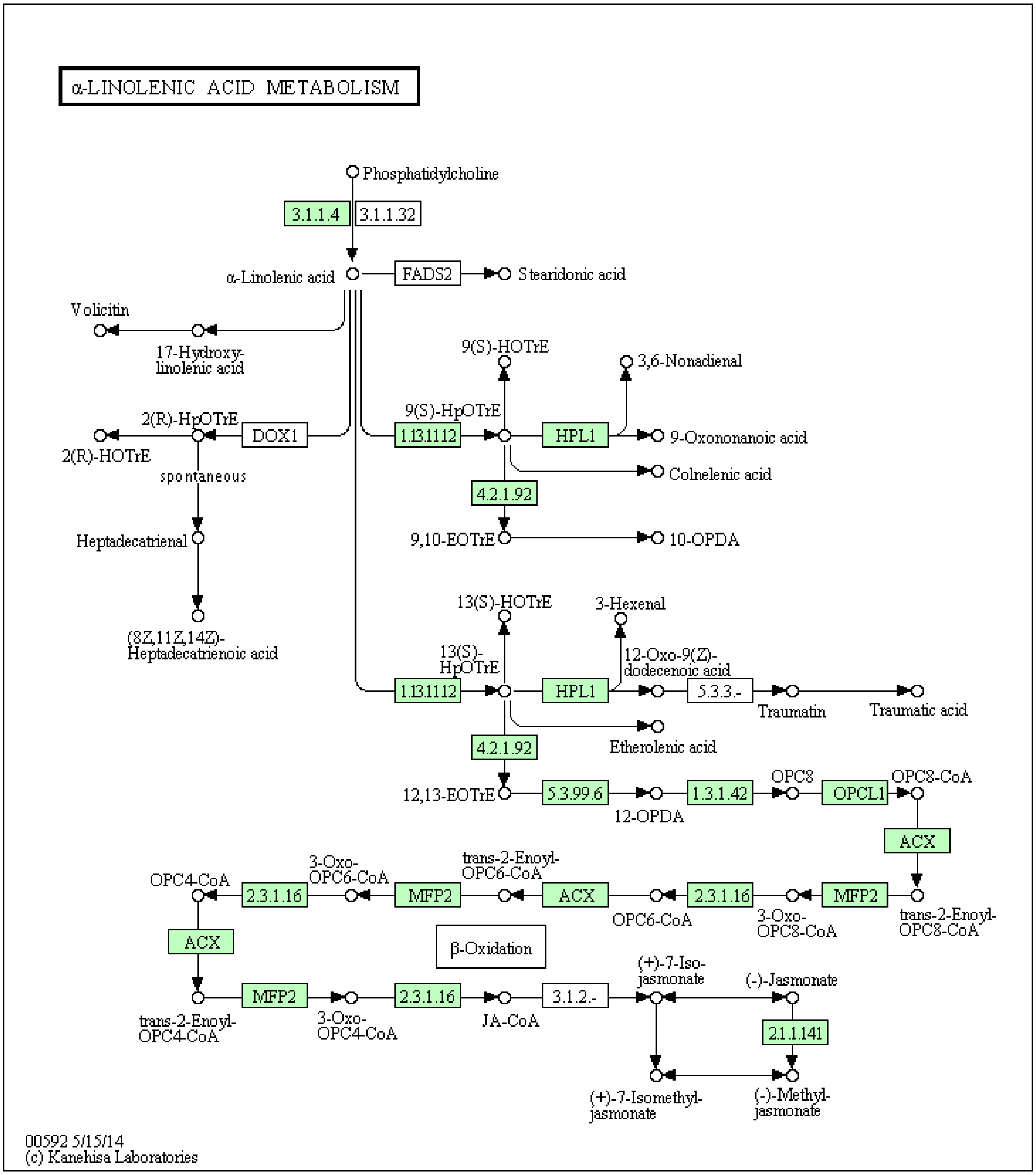
α-LINOLENIC ACID METABOLISM
Phosphatidylcholine
3.1.1.4
3.1.1.32
α-Linolenic acid
FADS2
Stearidonic acid
Volicitin
17-Hydroxy-linolenic acid
9(S)-HOTrE
3,6-Nonadienal
2(R)-HpOTrE
DOX1
2(R)-HOTrE
9(S)-HpOTrE
1.13.11.12
HPL1
9-Oxononanoic acid
Colnelenic acid
spontaneous
4.2.1.92
Heptadecatrienal
9,10-EOTrE
10-OPDA
(8Z,11Z,14Z)-Heptadecatrienoic acid
13(S)-HOTrE
3-Hexenal
13(S)-HpOTrE
12-Oxo-9(Z)-dodecenoic acid
1.13.11.12
HPL1
5.3.3.-
Traumatin
Traumatic acid
Etherolenic acid
4.2.1.92
12,13-EOTrE
5.3.99.6
12-OPDA
1.3.1.42
OPC8
OPCL1
OPC8-CoA
ACX
OPC4-CoA
2.3.1.16
3-Oxo-OPC6-CoA
MFP2
trans-2-Enoyl-OPC6-CoA
ACX
OPC6-CoA
2.3.1.16
3-Oxo-OPC8-CoA
MFP2
trans-2-Enoyl-OPC8-CoA
ACX
β-Oxidation
trans-2-Enoyl-OPC4-CoA
MFP2
3-Oxo-OPC4-CoA
2.3.1.16
JA-CoA
3.1.2.-
(+)-7-Iso-jasmonate
(-)-Jasmonate
2.1.1.141
(+)-7-Isomethyl-jasmonate
(-)-Methyl-jasmonate
00592 5/15/14
(c) Kanehisa Laboratories

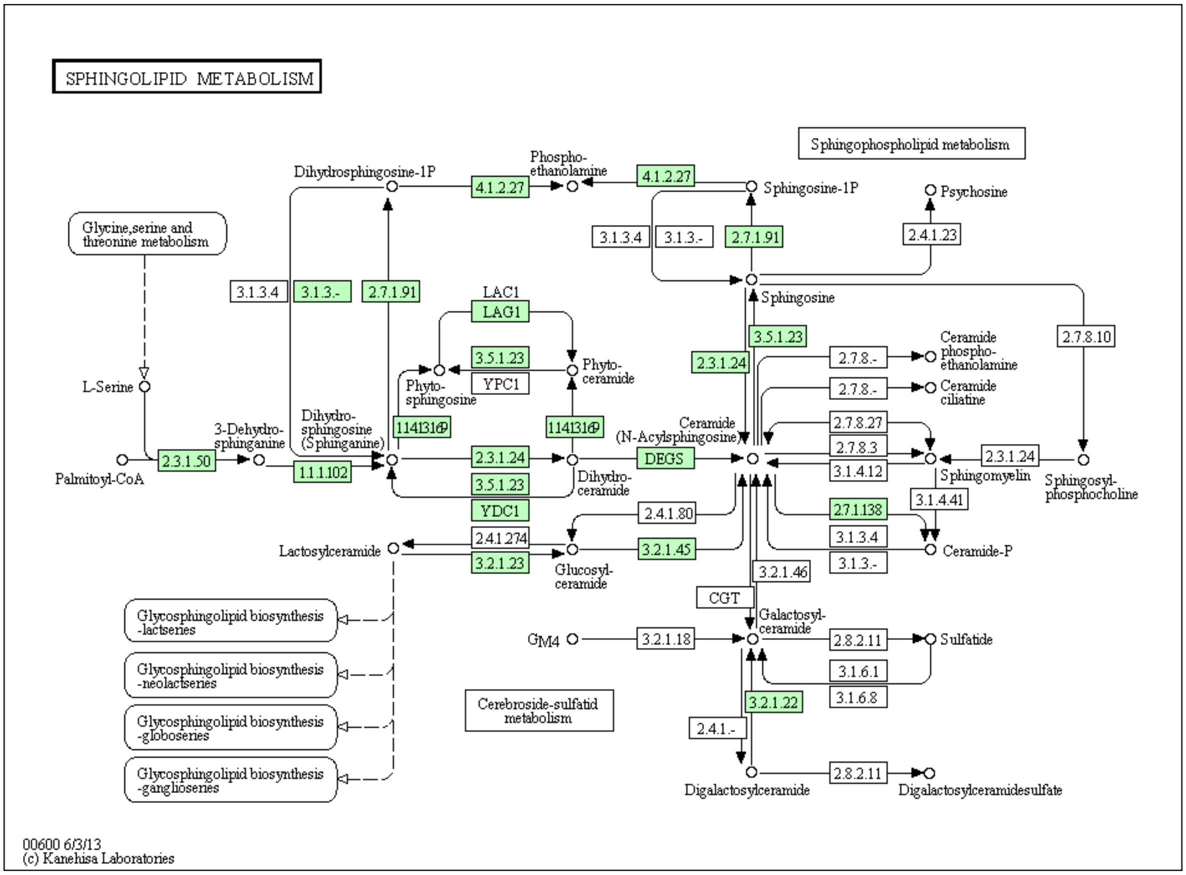
SPHINGOLIPID METABOLISM
Sphingophospholipid metabolism
Glycine,serine and threonine metabolism
Dihydrosphingosine-1P
Phospho-ethanolamine
Sphingosine-1P
Psychosine
Sphingosine
L-Serine
Palmitoyl-CoA
3-Dehydro-sphinganine
Dihydro-sphingosine (Sphinganine)
Phyto-sphingosine
Phyto-ceramide
Dihydro-ceramide
Ceramide (N-Acylsphingosine)
Ceramide phospho-ethanolamine
Ceramide ciliatine
Sphingomyelin
Sphingosyl-phosphocholine
Ceramide-P
Lactosylceramide
Glucosyl-ceramide
Galactosyl-ceramide
GM4
Sulfatide
Digalactosylceramide
Digalactosylceramidesulfate
Glycosphingolipid biosynthesis -lactseries
Glycosphingolipid biosynthesis -neolactseries
Glycosphingolipid biosynthesis -globoseries
Glycosphingolipid biosynthesis -ganglioseries
Cerebroside-sulfatid metabolism
LAC1
LAG1
YPC1
YDC1
DEGS
CGT
2.3.1.50
1.1.1.102
3.1.3.4
3.1.3.-
2.7.1.91
4.1.2.27
3.5.1.23
2.3.1.24
2.4.1.23
2.7.8.10
2.7.8.-
2.7.8.27
2.7.8.3
3.1.4.12
2.7.1.138
3.1.4.41
2.4.1.80
2.4.1.274
3.2.1.23
3.2.1.45
3.2.1.46
3.2.1.18
2.8.2.11
3.1.6.1
3.1.6.8
3.2.1.22
2.4.1.-
00600 6/3/13
(c) Kanehisa Laboratories

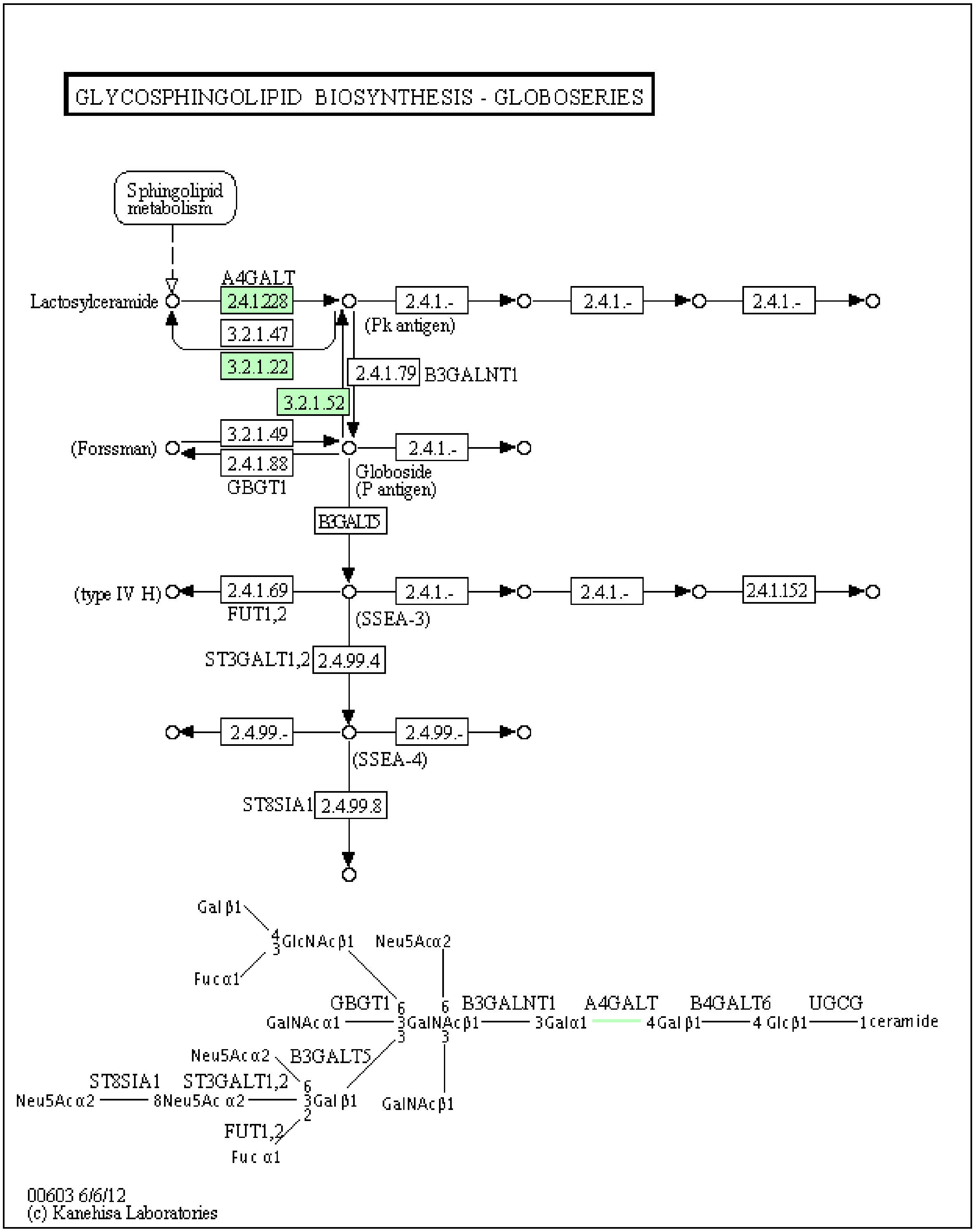
GLYCOSPHINGOLIPID BIOSYNTHESIS - GLOBOSERIES
Sphingolipid metabolism
Lactosylceramide
A4GALT
2.4.1.228
3.2.1.47
3.2.1.22
3.2.1.52
2.4.1.-
2.4.1.-
2.4.1.-
(Pk antigen)
2.4.1.79
B3GALNT1
(Forssman)
3.2.1.49
2.4.1.88
GBGT1
2.4.1.-
Globoside
(P antigen)
B3GALT5
(type IV H)
2.4.1.69
FUT1,2
2.4.1.-
2.4.1.-
2.4.1.152
(SSEA-3)
ST3GALT1,2
2.4.99.4
2.4.99.-
2.4.99.-
(SSEA-4)
ST8SIA1
2.4.99.8
Galβ1
4
3
GlcNAcβ1
Fucα1
Neu5Acα2
GBGT1
B3GALNT1
A4GALT
B4GALT6
UGCG
GalNAcα1
3GalNAcβ1
3Galα1
4Galβ1
4 Glcβ1
1ceramide
Neu5Acα2
B3GALT5
ST8SIA1
ST3GALT1,2
Neu5Acα2
8Neu5Acα2
3Galβ1
GalNAcβ1
FUT1,2
Fucα1
00603 6/6/12
(c) Kanehisa Laboratories

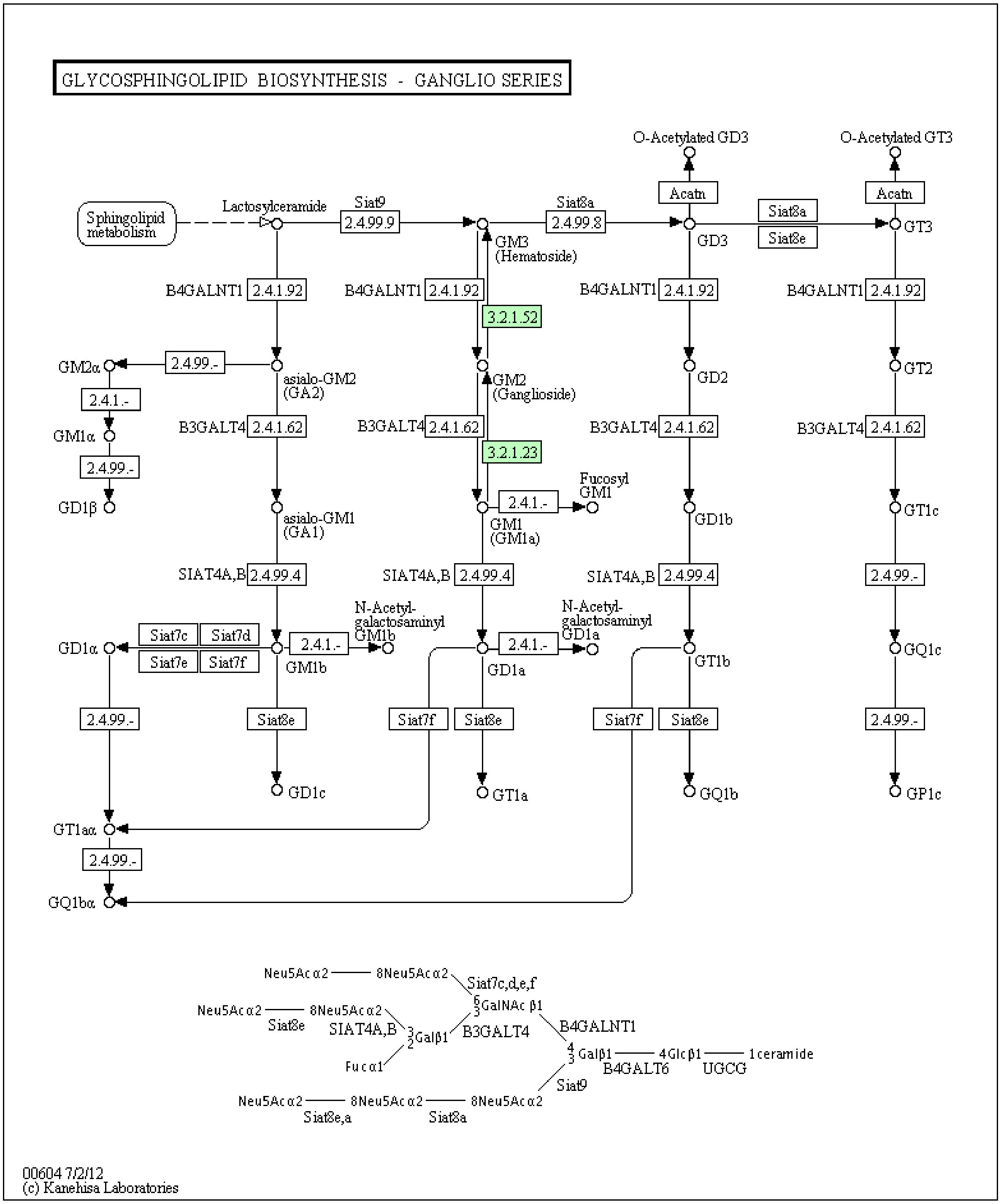
GLYCOSPHINGOLIPID BIOSYNTHESIS - GANGLIO SERIES
O-Acetylated GD3
O-Acetylated GT3
Acatn
Acatn
Sphingolipid metabolism
Lactosylceramide
Siat9
2.4.99.9
Siat8a
2.4.99.8
Siat8a
Siat8e
GM3 (Hematoside)
GD3
GT3
B4GALNT1
2.4.1.92
B4GALNT1
2.4.1.92
B4GALNT1
2.4.1.92
B4GALNT1
2.4.1.92
3.2.1.52
GM2α
2.4.99.-
asialo-GM2 (GA2)
GM2 (Ganglioside)
GD2
GT2
2.4.1.-
B3GALT4
2.4.1.62
B3GALT4
2.4.1.62
B3GALT4
2.4.1.62
B3GALT4
2.4.1.62
GM1α
3.2.1.23
2.4.99.-
Fucosyl GM1
2.4.1.-
GD1β
asialo-GM1 (GA1)
GM1 (GM1a)
GD1b
GT1c
SIAT4A,B
2.4.99.4
SIAT4A,B
2.4.99.4
SIAT4A,B
2.4.99.4
2.4.99.-
N-Acetyl-galactosaminyl GM1b
N-Acetyl-galactosaminyl GD1a
Siat7c
Siat7d
Siat7e
Siat7f
GD1α
2.4.1.-
GM1b
2.4.1.-
GD1a
GT1b
GQ1c
2.4.99.-
Siat8e
Siat7f
Siat8e
Siat7f
Siat8e
2.4.99.-
GD1c
GT1a
GQ1b
GP1c
GT1aα
2.4.99.-
GQ1bα
Neu5Acα2 — 8Neu5Acα2
Siat7c,d,e,f
Neu5Acα2 — 8Neu5Acα2
Siat8e
SIAT4A,B
6 3 GalNAc β1
B4GALNT1
3 2 Galβ1
B3GALT4
4 3 Galβ1 — 4Glcβ1 — 1ceramide
B4GALT6
UGCG
Fucα1
Siat9
Neu5Acα2 — 8Neu5Acα2 — 8Neu5Acα2
Siat8e,a
Siat8a
00604 7/2/12
(c) Kanehisa Laboratories

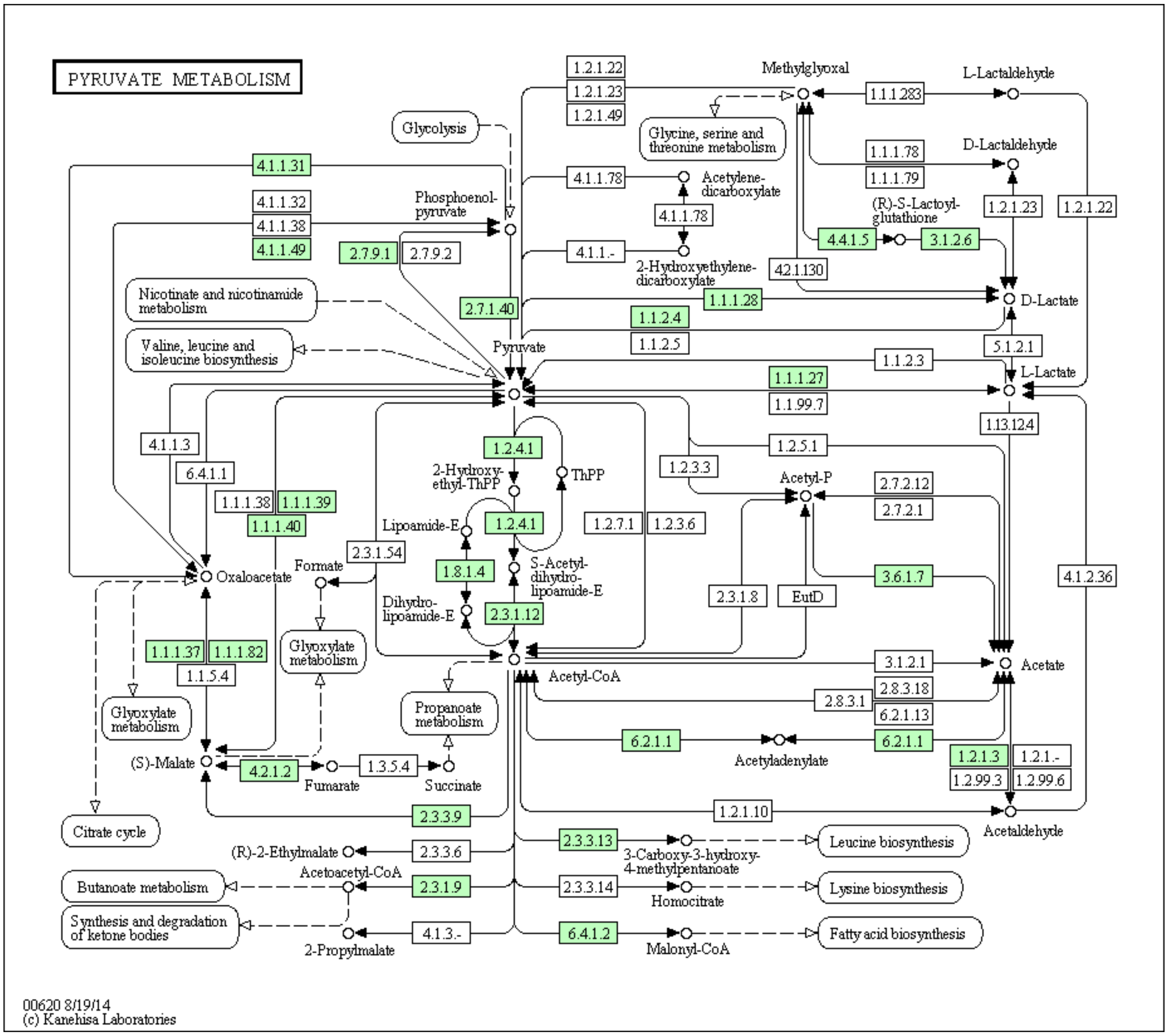
PYRUVATE METABOLISM
Glycolysis
Methylglyoxal
L-Lactaldehyde
D-Lactaldehyde
Glycine, serine and threonine metabolism
Phosphoenol-pyruvate
Acetylene-dicarboxylate
2-Hydroxyethylene-dicarboxylate
(R)-S-Lactoyl-glutathione
D-Lactate
L-Lactate
Nicotinate and nicotinamide metabolism
Valine, leucine and isoleucine biosynthesis
Pyruvate
2-Hydroxy-ethyl-ThPP
ThPP
Lipoamide-E
S-Acetyl-dihydro-lipoamide-E
Dihydro-lipoamide-E
Acetyl-P
Oxaloacetate
Formate
Glyoxylate metabolism
Acetyl-CoA
Acetate
Propanoate metabolism
Acetyladenylate
(S)-Malate
Fumarate
Succinate
Citrate cycle
Acetaldehyde
(R)-2-Ethylmalate
Acetoacetyl-CoA
Butanoate metabolism
Synthesis and degradation of ketone bodies
2-Propylmalate
3-Carboxy-3-hydroxy-4-methylpentanoate
Homocitrate
Malonyl-CoA
Leucine biosynthesis
Lysine biosynthesis
Fatty acid biosynthesis
1.2.1.22
1.2.1.23
1.2.1.49
1.1.1.283
4.1.1.31
4.1.1.32
4.1.1.38
4.1.1.49
2.7.9.1
2.7.9.2
4.1.1.78
4.1.1.-
1.1.1.78
1.1.1.79
4.4.1.5
3.1.2.6
4.2.1.130
2.7.1.40
1.1.1.28
1.1.2.4
1.1.2.5
5.1.2.1
1.1.2.3
1.1.1.27
1.1.99.7
1.13.12.4
4.1.1.3
6.4.1.1
1.2.4.1
1.2.3.3
1.2.5.1
2.7.2.12
2.7.2.1
1.1.1.38
1.1.1.39
1.1.1.40
1.2.7.1
1.2.3.6
2.3.1.54
1.8.1.4
2.3.1.12
2.3.1.8
EutD
3.6.1.7
4.1.2.36
1.1.1.37
1.1.1.82
1.1.5.4
3.1.2.1
2.8.3.18
2.8.3.1
6.2.1.13
6.2.1.1
4.2.1.2
1.3.5.4
1.2.1.3
1.2.1.-
1.2.99.3
1.2.99.6
2.3.3.9
1.2.1.10
2.3.3.6
2.3.3.13
2.3.1.9
2.3.3.14
4.1.3.-
6.4.1.2
00620 8/19/14
(c) Kanehisa Laboratories

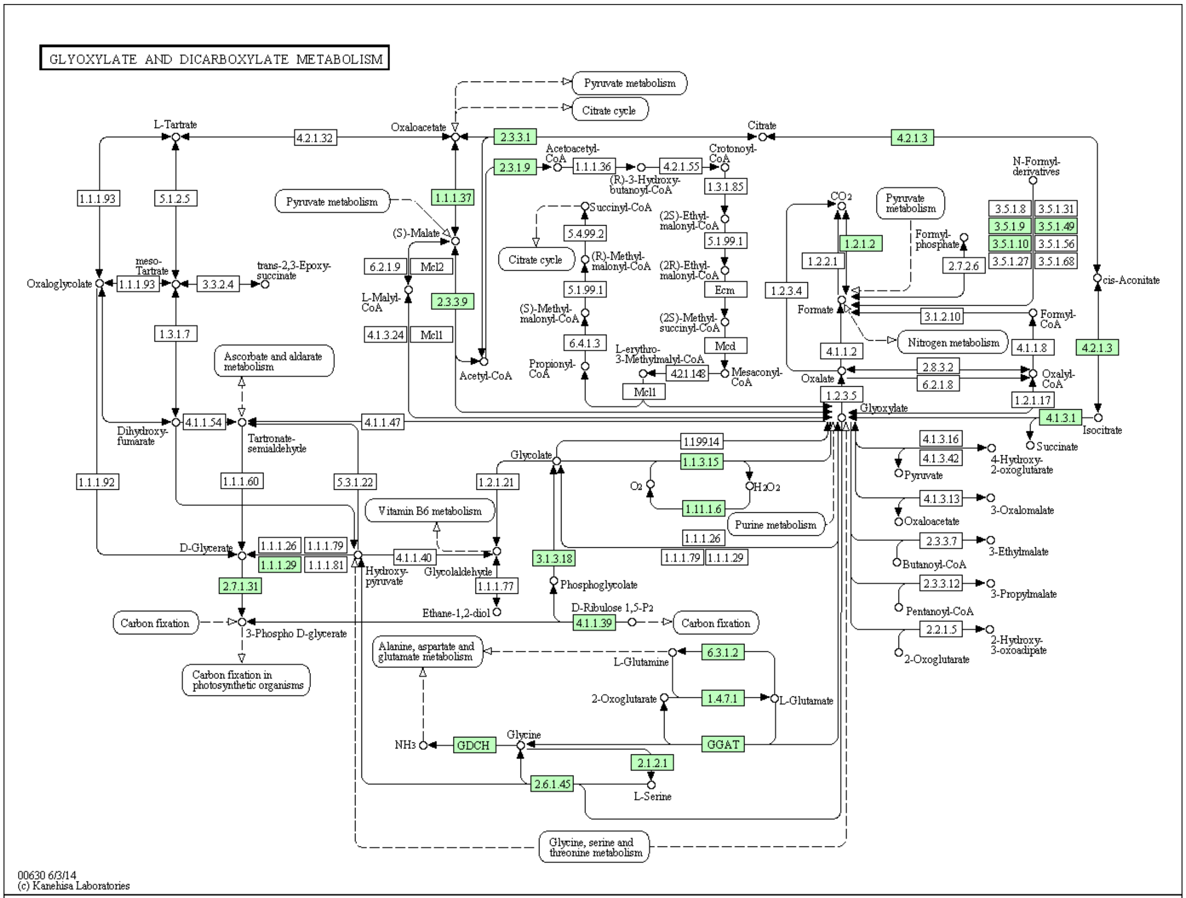
GLYOXYLATE AND DICARBOXYLATE METABOLISM
Pyruvate metabolism
Citrate cycle
L-Tartrate
Oxaloacetate
Citrate
4.2.1.32
2.3.3.1
4.2.1.3
Acetoacetyl-CoA
1.1.1.36
(R)-3-Hydroxybutanoyl-CoA
4.2.1.55
Crotonoyl-CoA
N-Formyl-derivatives
2.3.1.9
1.3.1.85
1.1.1.93
5.1.2.5
1.1.1.37
Succinyl-CoA
(2S)-Ethylmalonyl-CoA
CO2
3.5.1.8
3.5.1.31
3.5.1.9
3.5.1.49
3.5.1.10
3.5.1.56
3.5.1.27
3.5.1.68
(S)-Malate
5.4.99.2
5.1.99.1
1.2.1.2
Formylphosphate
(R)-Methylmalonyl-CoA
(2R)-Ethylmalonyl-CoA
1.2.2.1
2.7.2.6
6.2.1.9
Mcl2
meso-Tartrate
trans-2,3-Epoxysuccinate
cis-Aconitate
Oxaloglycolate
1.1.1.93
3.3.2.4
L-Malyl-CoA
2.3.3.9
5.1.99.1
Ecm
1.2.3.4
Formate
3.1.2.10
Formyl-CoA
(S)-Methylmalonyl-CoA
(2S)-Methylsuccinyl-CoA
1.3.1.7
4.1.3.24
Mcl1
6.4.1.3
Mcd
Nitrogen metabolism
Ascorbate and aldarate metabolism
Acetyl-CoA
Propionyl-CoA
L-erythro-3-Methylmalyl-CoA
4.2.1.148
Mesaconyl-CoA
4.1.1.2
2.8.3.2
4.1.1.8
4.2.1.3
Oxalate
Oxalyl-CoA
Mcl1
6.2.1.8
1.2.3.5
1.2.1.17
Glyoxylate
4.1.3.1
Isocitrate
Dihydroxyfumarate
4.1.1.54
Tartronate-semialdehyde
4.1.1.47
4.1.3.16
Succinate
1.1.99.14
4.1.3.42
4-Hydroxy-2-oxoglutarate
Glycolate
1.1.3.15
Pyruvate
O2
H2O2
1.1.1.92
1.1.1.60
5.3.1.22
1.2.1.21
4.1.3.13
3-Oxalomalate
Vitamin B6 metabolism
1.11.1.6
Oxaloacetate
Purine metabolism
2.3.3.7
3-Ethylmalate
1.1.1.26
D-Glycerate
1.1.1.26
1.1.1.79
1.1.1.29
1.1.1.81
4.1.1.40
Hydroxypyruvate
Glycolaldehyde
3.1.3.18
1.1.1.79
1.1.1.29
Butanoyl-CoA
2.7.1.31
1.1.1.77
Phosphoglycolate
2.3.3.12
3-Propylmalate
Ethane-1,2-diol
D-Ribulose 1,5-P2
Pentanoyl-CoA
Carbon fixation
4.1.1.39
Carbon fixation
2.2.1.5
3-Phospho D-glycerate
2-Hydroxy-3-oxoadipate
Alanine, aspartate and glutamate metabolism
6.3.1.2
L-Glutamine
2-Oxoglutarate
Carbon fixation in photosynthetic organisms
2-Oxoglutarate
1.4.7.1
L-Glutamate
NH3
GDCH
Glycine
GGAT
2.1.2.1
2.6.1.45
L-Serine
Glycine, serine and threonine metabolism
00630 6/3/14
(c) Kanehisa Laboratories

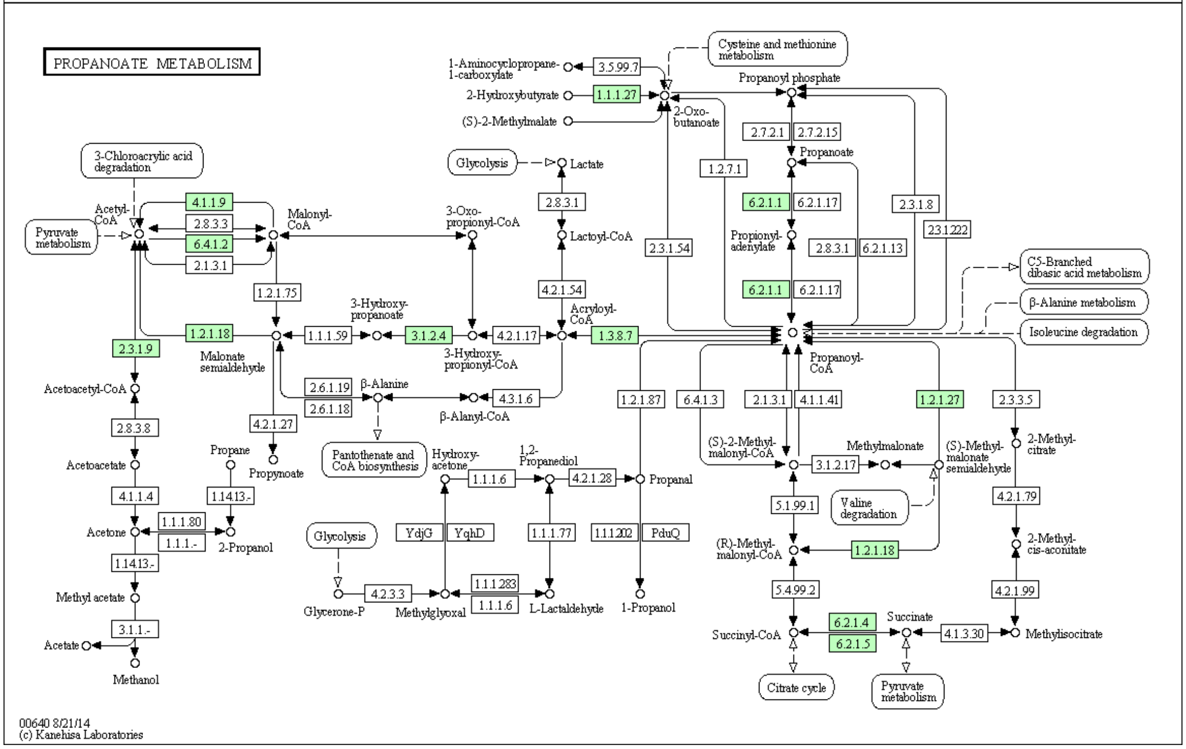
PROPANOATE METABOLISM
Cysteine and methionine metabolism
1-Aminocyclopropane-1-carboxylate
3.5.99.7
Propanoyl phosphate
2-Hydroxybutyrate
1.1.1.27
2-Oxobutanoate
(S)-2-Methylmalate
2.7.2.1
2.7.2.15
3-Chloroacrylic acid degradation
Glycolysis
Lactate
Propanoate
1.2.7.1
Acetyl-CoA
4.1.1.9
Malonyl-CoA
3-Oxopropionyl-CoA
2.8.3.1
2.3.1.8
2.8.3.3
6.2.1.1
6.2.1.17
Pyruvate metabolism
6.4.1.2
Lactoyl-CoA
2.3.1.54
Propionyl-adenylate
2.8.3.1
6.2.1.13
2.3.1.222
2.1.3.1
C5-Branched dibasic acid metabolism
1.2.1.75
4.2.1.54
6.2.1.1
6.2.1.17
3-Hydroxypropanoate
Acryloyl-CoA
β-Alanine metabolism
1.2.1.18
1.1.1.59
3.1.2.4
4.2.1.17
1.3.8.7
Isoleucine degradation
2.3.1.9
Malonate semialdehyde
3-Hydroxypropionyl-CoA
Propanoyl-CoA
Acetoacetyl-CoA
2.6.1.19
β-Alanine
4.3.1.6
1.2.1.87
6.4.1.3
2.1.3.1
4.1.1.41
1.2.1.27
2.3.3.5
2.6.1.18
β-Alanyl-CoA
2.8.3.8
4.2.1.27
2-Methylcitrate
Acetoacetate
Propane
Pantothenate and CoA biosynthesis
Hydroxyacetone
1,2-Propanediol
(S)-2-Methylmalonyl-CoA
Methylmalonate
(S)-Methylmalonate semialdehyde
Propynoate
4.1.1.4
1.14.13.-
1.1.1.6
4.2.1.28
Propanal
3.1.2.17
Valine degradation
4.2.1.79
5.1.99.1
1.1.1.80
Acetone
1.1.1.-
2-Propanol
Glycolysis
YdjG
YqhD
1.1.1.77
1.1.1.202
PduQ
(R)-Methylmalonyl-CoA
1.2.1.18
2-Methyl-cis-aconitate
1.14.13.-
Methyl acetate
4.2.3.3
1.1.1.283
Glycerone-P
Methylglyoxal
1.1.1.6
L-Lactaldehyde
1-Propanol
5.4.99.2
4.2.1.99
3.1.1.-
6.2.1.4
Succinate
Acetate
Succinyl-CoA
6.2.1.5
4.1.3.30
Methylisocitrate
Methanol
Citrate cycle
Pyruvate metabolism
00640 8/21/14
(c) Kanehisa Laboratories

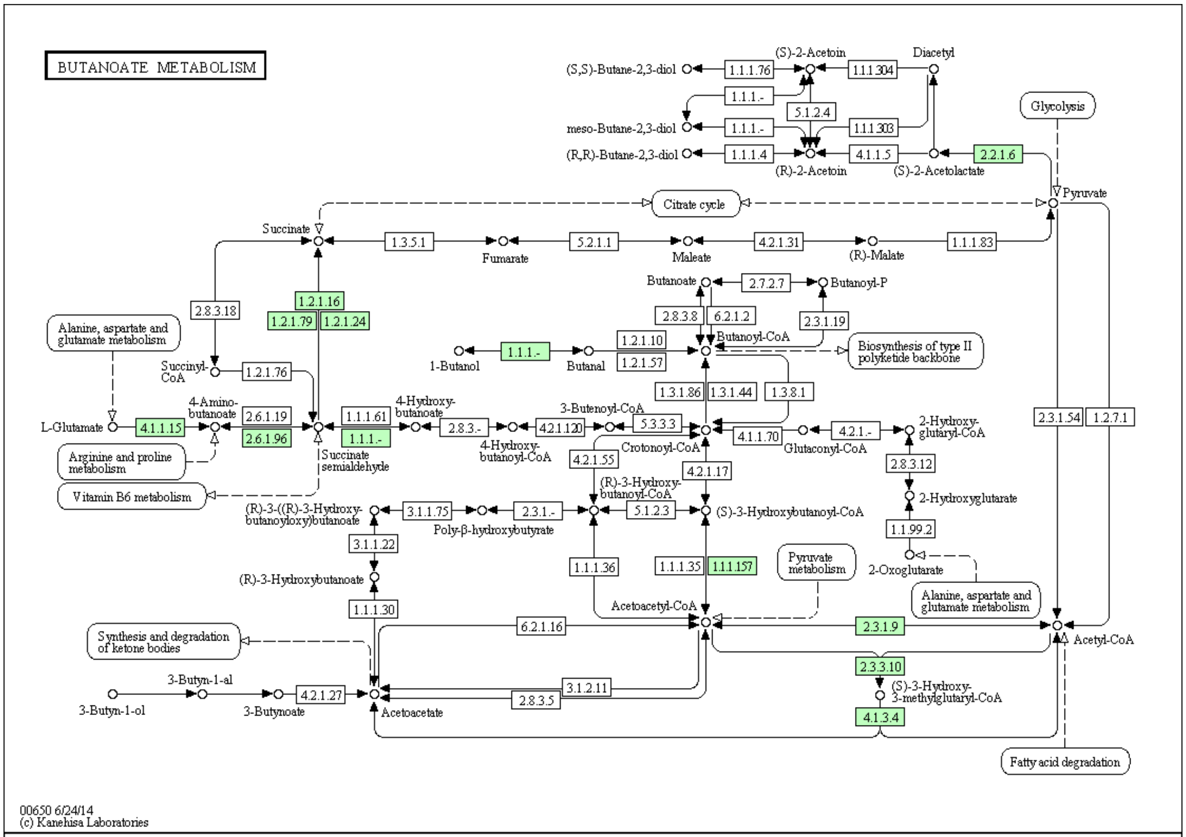

BUTANOATE METABOLISM
(S)-2-Acetoin
Diacetyl
(S,S)-Butane-2,3-diol
1.1.1.76
1.1.1.304
1.1.1.-
5.1.2.4
meso-Butane-2,3-diol
1.1.1.303
(R,R)-Butane-2,3-diol
1.1.1.4
4.1.1.5
2.2.1.6
(R)-2-Acetoin
(S)-2-Acetolactate
Glycolysis
Pyruvate
Citrate cycle
Succinate
1.3.5.1
Fumarate
5.2.1.1
Maleate
4.2.1.31
(R)-Malate
1.1.1.83
Butanoate
2.7.2.7
Butanoyl-P
2.8.3.18
1.2.1.16
1.2.1.79
1.2.1.24
2.8.3.8
6.2.1.2
2.3.1.19
Alanine, aspartate and glutamate metabolism
1.2.1.10
Butanoyl-CoA
Biosynthesis of type II polyketide backbone
1-Butanol
1.1.1.-
Butanal
1.2.1.57
Succinyl-CoA
1.2.1.76
1.3.1.86
1.3.1.44
1.3.8.1
4-Amino-butanoate
4-Hydroxy-butanoate
3-Butenoyl-CoA
L-Glutamate
4.1.1.15
2.6.1.19
1.1.1.61
2.8.3.-
4.2.1.120
5.3.3.3
4.1.1.70
4.2.1.-
2-Hydroxy-glutaryl-CoA
2.3.1.54
1.2.7.1
2.6.1.96
1.1.1.-
Succinate semialdehyde
4-Hydroxy-butanoyl-CoA
Crotonoyl-CoA
Glutaconyl-CoA
Arginine and proline metabolism
4.2.1.55
4.2.1.17
2.8.3.12
Vitamin B6 metabolism
(R)-3-Hydroxy-butanoyl-CoA
2-Hydroxyglutarate
(R)-3-((R)-3-Hydroxy-butanoyloxy)butanoate
3.1.1.75
2.3.1.-
5.1.2.3
(S)-3-Hydroxybutanoyl-CoA
Poly-β-hydroxybutyrate
3.1.1.22
1.1.99.2
Pyruvate metabolism
1.1.1.36
1.1.1.35
1.1.1.157
2-Oxoglutarate
(R)-3-Hydroxybutanoate
Alanine, aspartate and glutamate metabolism
1.1.1.30
Acetoacetyl-CoA
6.2.1.16
2.3.1.9
Acetyl-CoA
Synthesis and degradation of ketone bodies
2.3.3.10
3-Butyn-1-al
3.1.2.11
(S)-3-Hydroxy-3-methylglutaryl-CoA
3-Butyn-1-ol
3-Butynoate
4.2.1.27
2.8.3.5
Acetoacetate
4.1.3.4
Fatty acid degradation
00650 6/24/14
(c) Kanehisa Laboratories

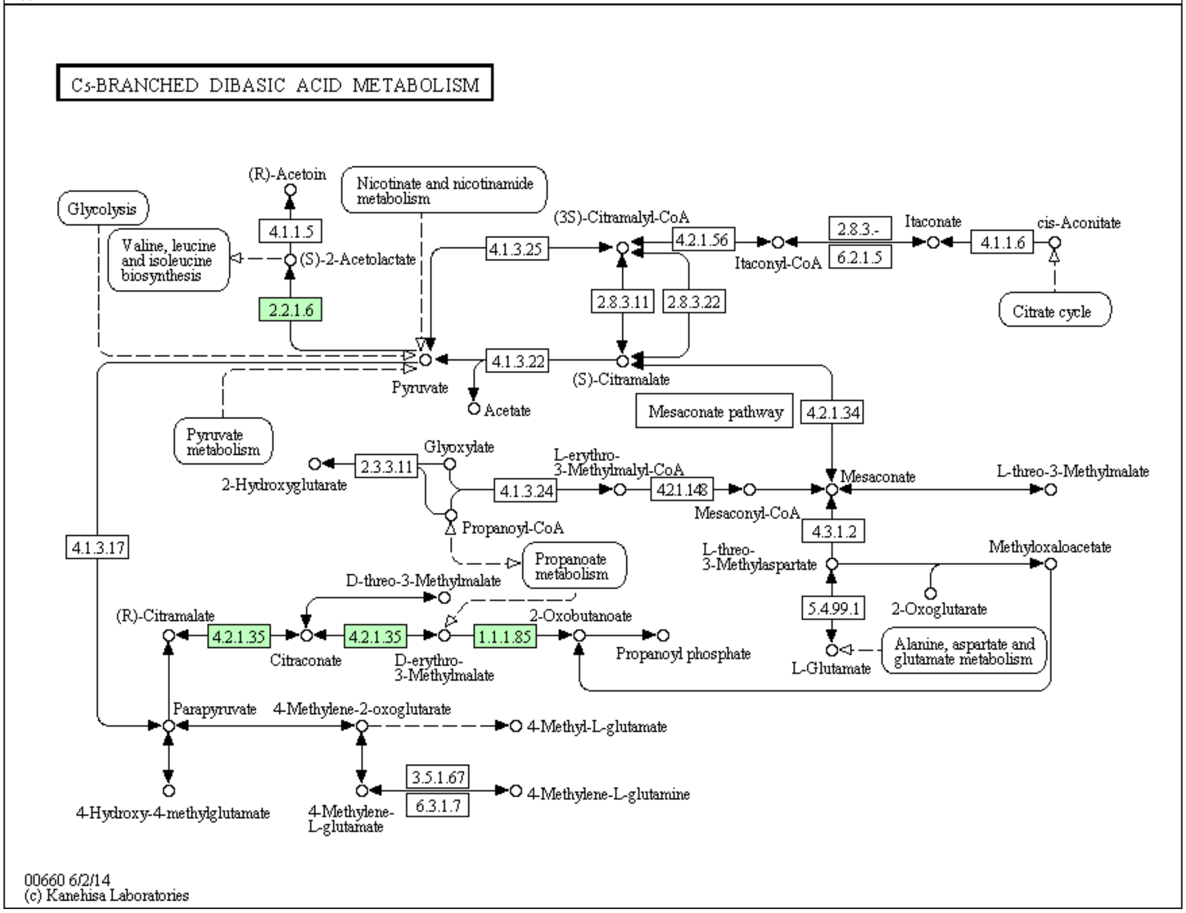

C5-BRANCHED DIBASIC ACID METABOLISM
(R)-Acetoin
Nicotinate and nicotinamide metabolism
Glycolysis
4.1.1.5
(3S)-Citramalyl-CoA
4.2.1.56
2.8.3.-
Itaconate
4.1.1.6
cis-Aconitate
Valine, leucine and isoleucine biosynthesis
(S)-2-Acetolactate
4.1.3.25
Itaconyl-CoA
6.2.1.5
2.2.1.6
2.8.3.11
2.8.3.22
Citrate cycle
4.1.3.22
Pyruvate
(S)-Citramalate
Acetate
Mesaconate pathway
4.2.1.34
Pyruvate metabolism
Glyoxylate
L-erythro-3-Methylmalyl-CoA
2.3.3.11
2-Hydroxyglutarate
4.1.3.24
4.2.1.148
Mesaconate
L-threo-3-Methylmalate
Mesaconyl-CoA
Propanoyl-CoA
4.3.1.2
4.1.3.17
L-threo-3-Methylaspartate
Methyloxaloacetate
Propanoate metabolism
D-threo-3-Methylmalate
5.4.99.1
2-Oxoglutarate
(R)-Citramalate
2-Oxobutanoate
4.2.1.35
4.2.1.35
1.1.1.85
Citraconate
D-erythro-3-Methylmalate
Propanoyl phosphate
L-Glutamate
Alanine, aspartate and glutamate metabolism
Parapyruvate
4-Methylene-2-oxoglutarate
4-Methyl-L-glutamate
3.5.1.67
6.3.1.7
4-Methylene-L-glutamine
4-Hydroxy-4-methylglutamate
4-Methylene-L-glutamate
00660 6/2/14
(c) Kanehisa Laboratories

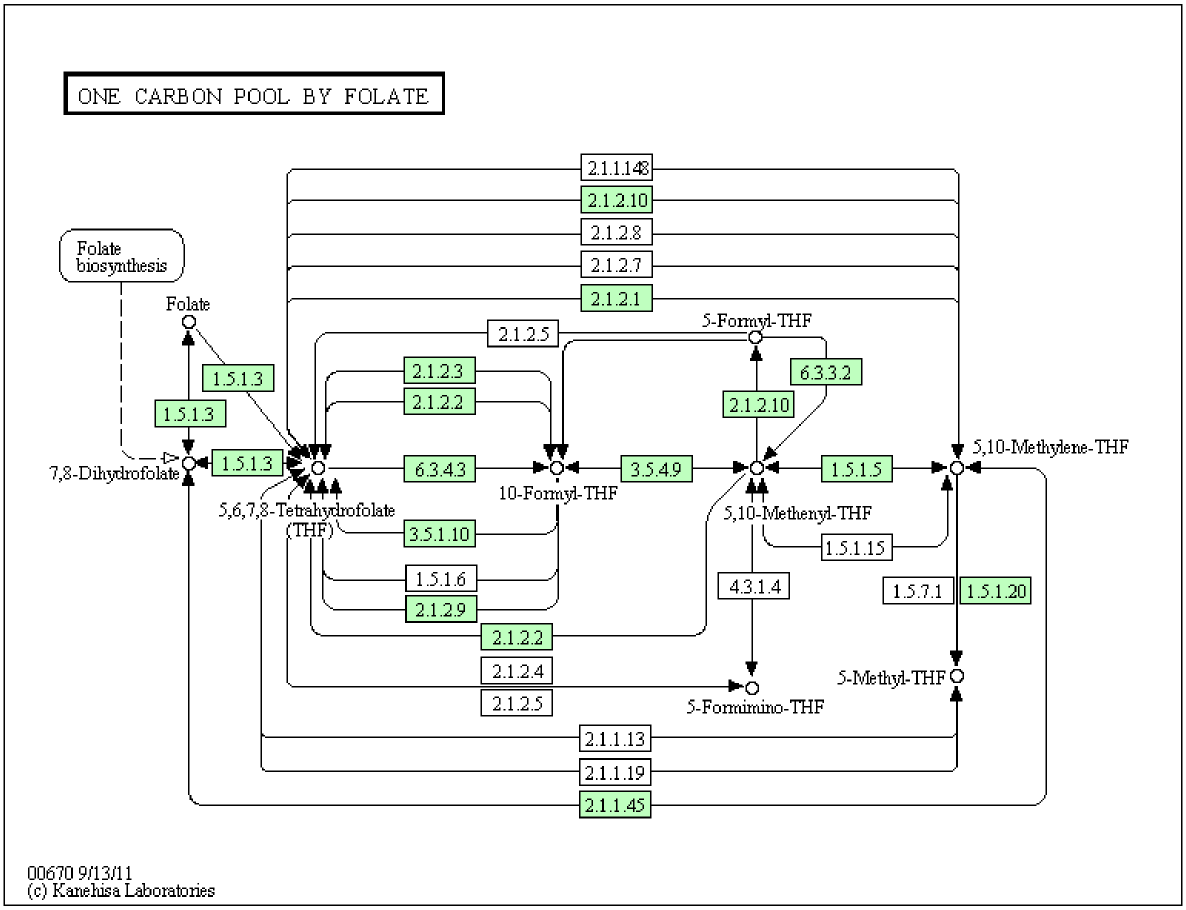
ONE CARBON POOL BY FOLATE
2.1.1.148
2.1.2.10
2.1.2.8
2.1.2.7
2.1.2.1
Folate biosynthesis
Folate
1.5.1.3
1.5.1.3
1.5.1.3
7,8-Dihydrofolate
2.1.2.5
5-Formyl-THF
2.1.2.3
2.1.2.2
6.3.3.2
2.1.2.10
6.3.4.3
3.5.4.9
1.5.1.5
5,10-Methylene-THF
5,6,7,8-Tetrahydrofolate (THF)
10-Formyl-THF
5,10-Methenyl-THF
3.5.1.10
1.5.1.15
1.5.1.6
4.3.1.4
1.5.7.1
1.5.1.20
2.1.2.9
2.1.2.2
2.1.2.4
5-Methyl-THF
2.1.2.5
5-Formimino-THF
2.1.1.13
2.1.1.19
2.1.1.45
00670 9/13/11
(c) Kanehisa Laboratories

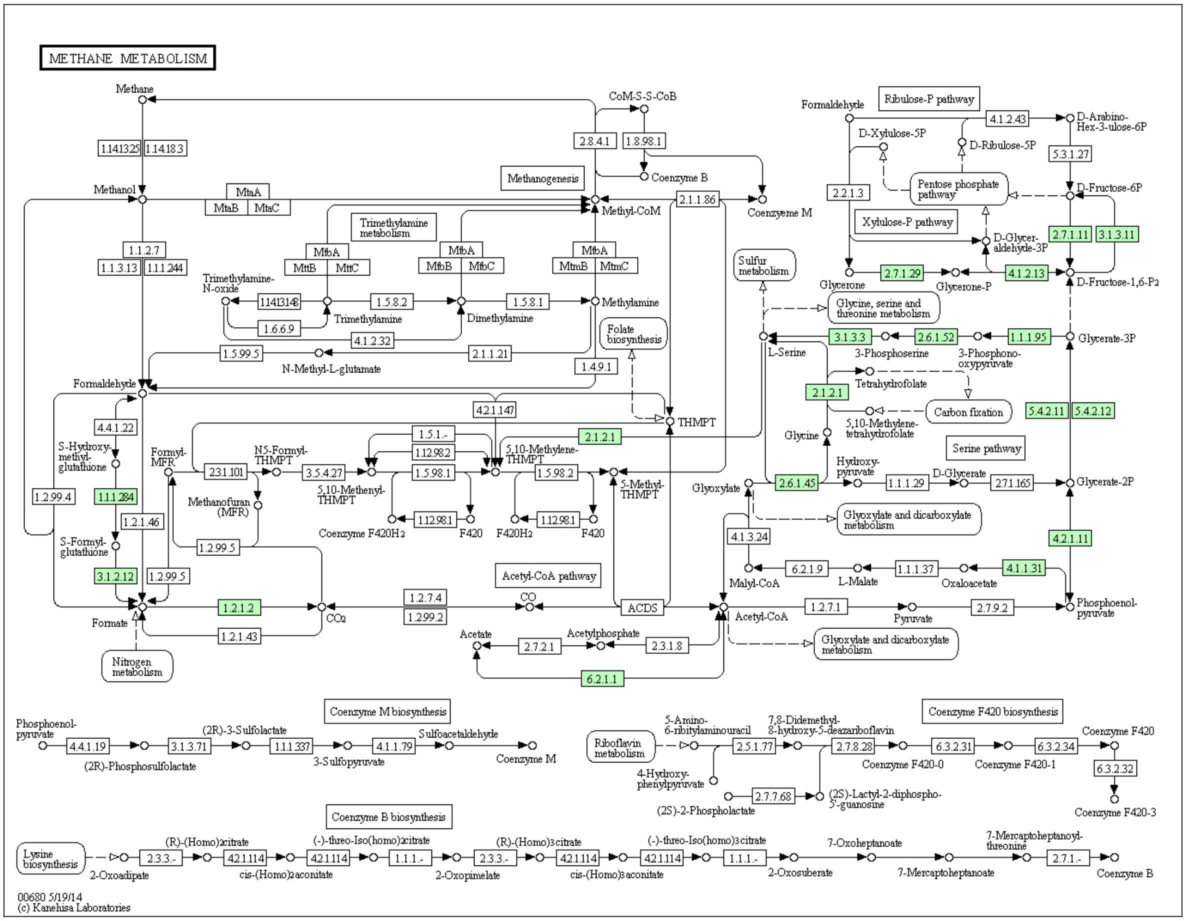
METHANE METABOLISM
Methane
Methanol
Formaldehyde
Formate
CO2
CO
CoM-S-S-CoB
Coenzyme B
Coenzyme M
Methyl-CoM
Methanogenesis
Trimethylamine metabolism
Trimethylamine-N-oxide
Trimethylamine
Dimethylamine
Methylamine
N-Methyl-L-glutamate
MtaA
MtaB
MtaC
MttB
MttC
MtbA
MtbB
MtbC
MtmB
MtmC
Folate biosynthesis
THMPT
S-Hydroxy-methyl-glutathione
S-Formyl-glutathione
Formyl-MFR
Methanofuran (MFR)
N5-Formyl-THMPT
5,10-Methenyl-THMPT
5,10-Methylene-THMPT
5-Methyl-THMPT
Coenzyme F420H2
F420
F420H2
Acetyl-CoA pathway
ACDS
Acetate
Acetylphosphate
Acetyl-CoA
Nitrogen metabolism
Ribulose-P pathway
Formaldehyde
D-Xylulose-5P
D-Ribulose-5P
D-Arabino-Hex-3-ulose-6P
D-Fructose-6P
Pentose phosphate pathway
Xylulose-P pathway
D-Glyceraldehyde-3P
Glycerone
Glycerone-P
D-Fructose-1,6-P2
Sulfur metabolism
Glycine, serine and threonine metabolism
L-Serine
3-Phosphoserine
3-Phosphonooxypyruvate
Glycerate-3P
Tetrahydrofolate
Carbon fixation
5,10-Methylene-tetrahydrofolate
Glycine
Serine pathway
Glyoxylate
Hydroxypyruvate
D-Glycerate
Glycerate-2P
Glyoxylate and dicarboxylate metabolism
Malyl-CoA
L-Malate
Oxaloacetate
Phosphoenolpyruvate
Pyruvate
Coenzyme M biosynthesis
Phosphoenolpyruvate
(2R)-Phosphosulfolactate
(2R)-3-Sulfolactate
3-Sulfopyruvate
Sulfoacetaldehyde
Coenzyme M
Coenzyme F420 biosynthesis
Riboflavin metabolism
5-Amino-6-ribitylaminouracil
4-Hydroxyphenylpyruvate
7,8-Didemethyl-8-hydroxy-5-deazariboflavin
(2S)-2-Phospholactate
(2S)-Lactyl-2-diphospho-5'-guanosine
Coenzyme F420-0
Coenzyme F420-1
Coenzyme F420
Coenzyme F420-3
Coenzyme B biosynthesis
Lysine biosynthesis
2-Oxoadipate
(R)-(Homo)2citrate
cis-(Homo)2aconitate
(-)-threo-Iso(homo)2citrate
2-Oxopimelate
(R)-(Homo)3citrate
cis-(Homo)3aconitate
(-)-threo-Iso(homo)3citrate
2-Oxosuberate
7-Oxoheptanoate
7-Mercaptoheptanoate
7-Mercaptoheptanoyl-threonine
Coenzyme B
1.14.13.25
1.14.18.3
1.1.2.7
1.1.3.13
1.1.2.44
2.8.4.1
1.8.98.1
2.1.1.86
1.14.13.148
1.6.6.9
1.5.8.2
1.5.8.1
4.1.2.32
1.5.99.5
2.1.1.21
1.4.9.1
4.4.1.22
1.1.1.284
3.1.2.12
1.2.1.46
1.2.99.4
1.2.99.5
1.2.1.2
1.2.1.43
4.2.1.147
2.3.1.101
3.5.4.27
1.5.1.-
1.12.98.2
1.5.98.1
1.5.98.2
1.12.98.1
2.1.2.1
1.2.7.4
1.2.99.2
2.7.2.1
2.3.1.8
6.2.1.1
4.1.2.43
5.3.1.27
2.2.1.3
2.7.1.11
3.1.3.11
2.7.1.29
4.1.2.13
3.1.3.3
2.6.1.52
1.1.1.95
5.4.2.11
5.4.2.12
2.6.1.45
1.1.1.29
2.7.1.165
4.2.1.11
4.1.3.24
6.2.1.9
1.1.1.37
4.1.1.31
1.2.7.1
2.7.9.2
4.4.1.19
3.1.3.71
1.1.1.337
4.1.1.79
2.5.1.77
2.7.8.28
6.3.2.31
6.3.2.34
6.3.2.32
2.7.7.68
2.3.3.-
4.2.1.114
1.1.1.-
2.7.1.-
00680 5/19/14
(c) Kanehisa Laboratories

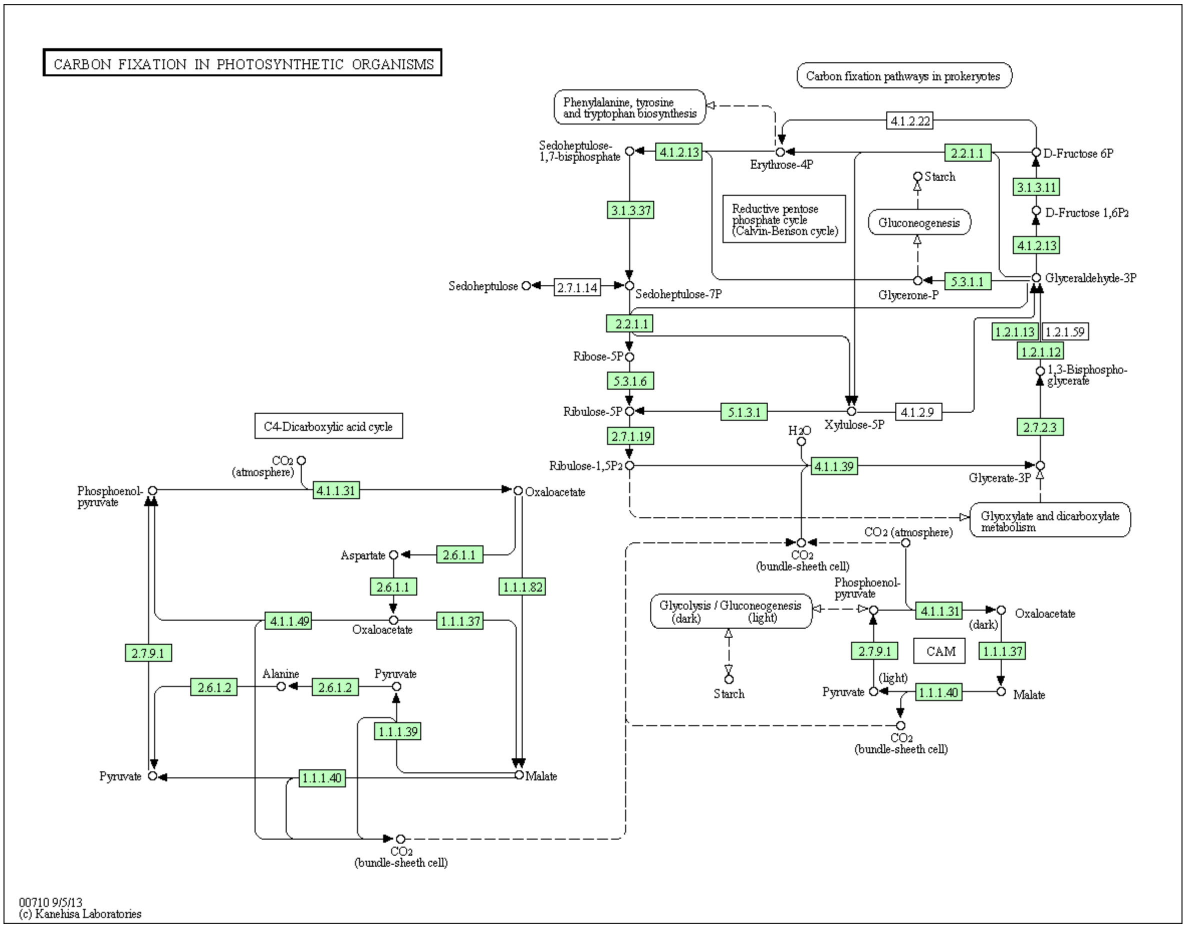

CARBON FIXATION IN PHOTOSYNTHETIC ORGANISMS
Carbon fixation pathways in prokaryotes
Phenylalanine, tyrosine and tryptophan biosynthesis
4.1.2.22
Sedoheptulose-1,7-bisphosphate
4.1.2.13
Erythrose-4P
2.2.1.1
D-Fructose 6P
3.1.3.11
D-Fructose 1,6P2
4.1.2.13
Starch
Gluconeogenesis
Reductive pentose phosphate cycle (Calvin-Benson cycle)
3.1.3.37
Sedoheptulose
2.7.1.14
Sedoheptulose-7P
Glycerone-P
5.3.1.1
Glyceraldehyde-3P
2.2.1.1
1.2.1.13
1.2.1.59
1.2.1.12
Ribose-5P
1,3-Bisphospho-glycerate
5.3.1.6
Ribulose-5P
5.1.3.1
Xylulose-5P
4.1.2.9
C4-Dicarboxylic acid cycle
2.7.1.19
H2O
2.7.2.3
Ribulose-1,5P2
4.1.1.39
Glycerate-3P
CO2 (atmosphere)
Phosphoenol-pyruvate
4.1.1.31
Oxaloacetate
Glyoxylate and dicarboxylate metabolism
CO2 (bundle-sheath cell)
CO2 (atmosphere)
Aspartate
2.6.1.1
2.6.1.1
1.1.1.82
Glycolysis / Gluconeogenesis (dark) (light)
Phosphoenol-pyruvate
4.1.1.31
Oxaloacetate
4.1.1.49
Oxaloacetate
1.1.1.37
(dark)
2.7.9.1
CAM
1.1.1.37
2.7.9.1
Alanine
Pyruvate
Starch
(light)
2.6.1.2
2.6.1.2
Pyruvate
1.1.1.40
Malate
1.1.1.39
CO2 (bundle-sheath cell)
Pyruvate
1.1.1.40
Malate
CO2 (bundle-sheath cell)
00710 9/5/13
(c) Kanehisa Laboratories

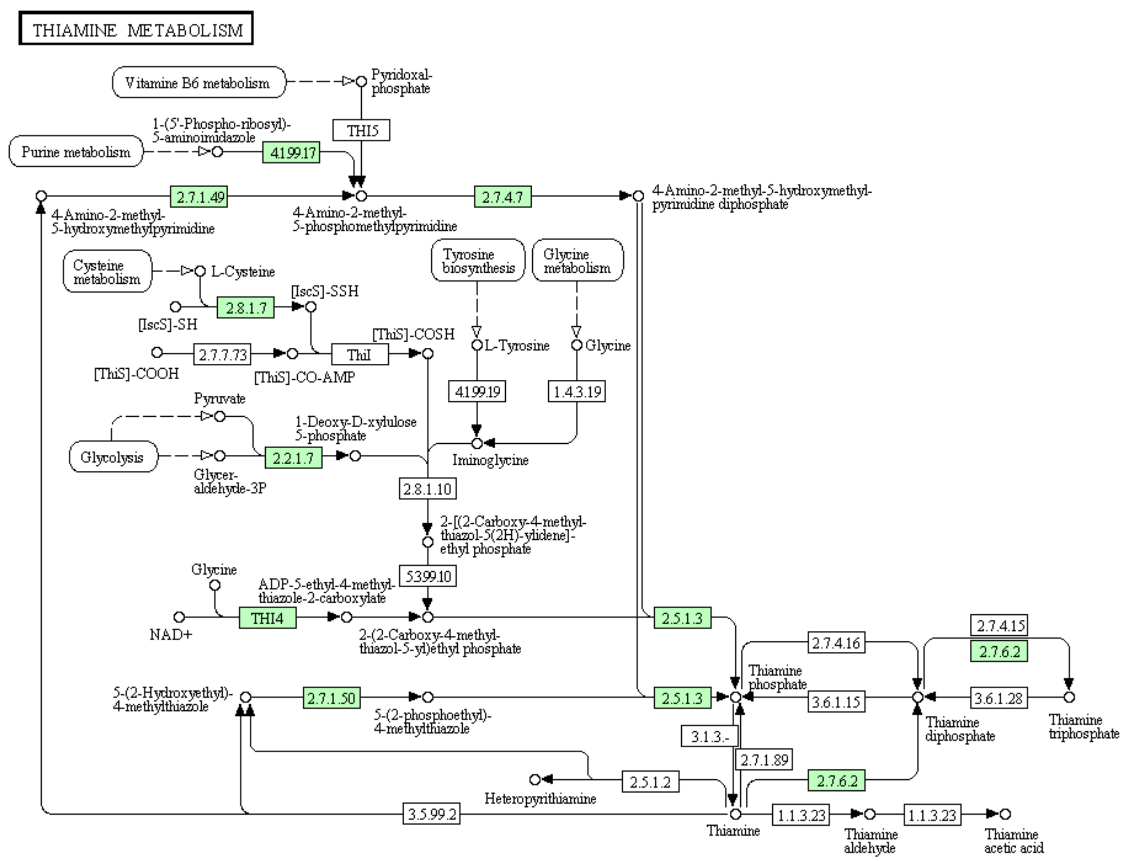

00730 6/26/14
(c) Kanehisa Laboratories

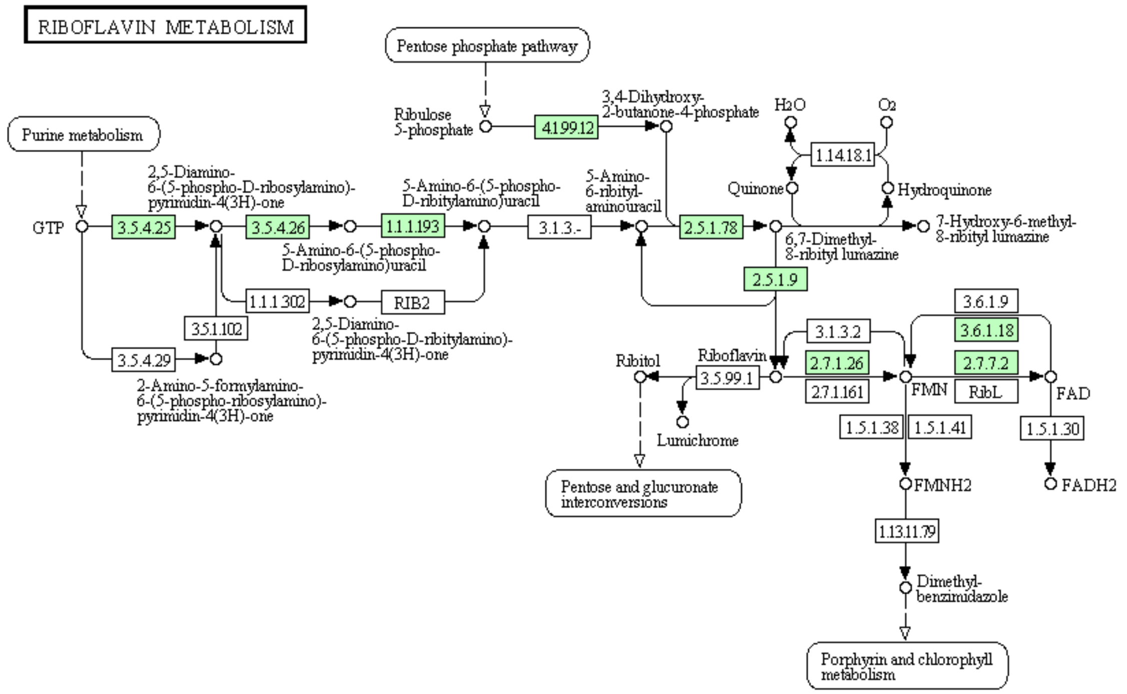

00740 8/4/14
(c) Kanehisa Laboratories

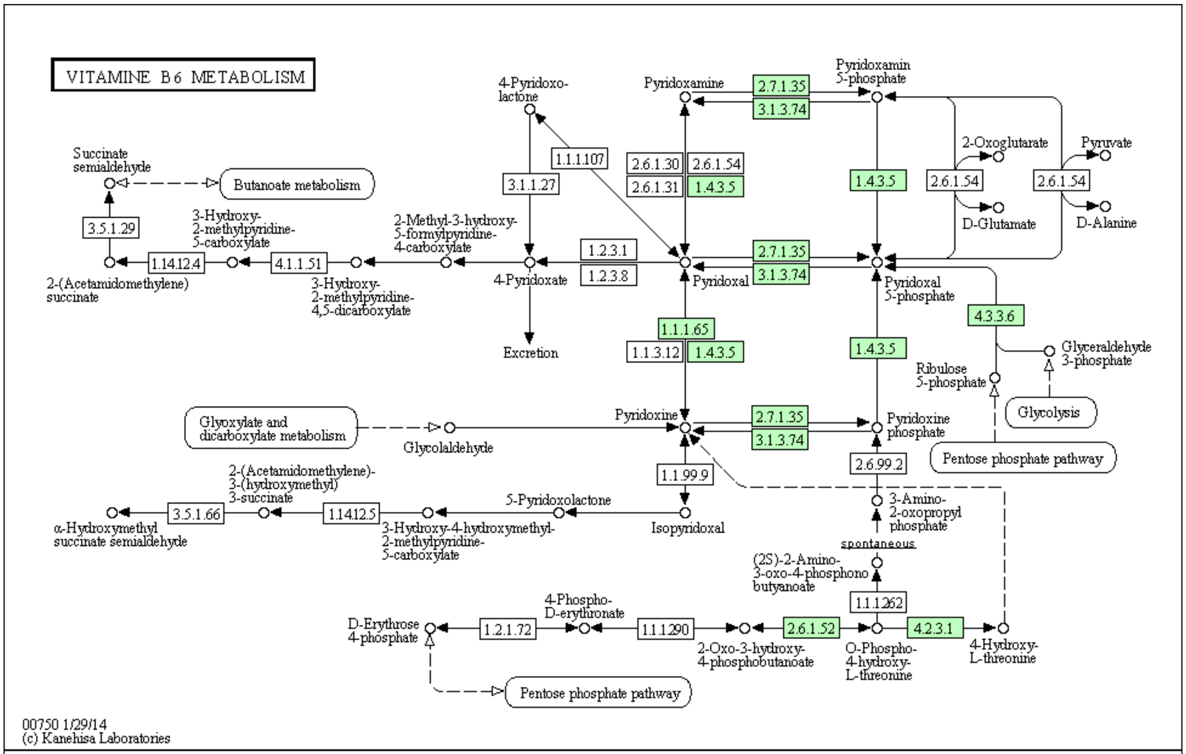
VITAMINE B6 METABOLISM
Succinate semialdehyde
Butanoate metabolism
3.5.1.29
3-Hydroxy-2-methylpyridine-5-carboxylate
2-Methyl-3-hydroxy-5-formylpyridine-4-carboxylate
1.14.12.4
4.1.1.51
2-(Acetamidomethylene) succinate
3-Hydroxy-2-methylpyridine-4,5-dicarboxylate
4-Pyridoxo-lactone
3.1.1.27
1.1.1.107
4-Pyridoxate
Excretion
1.2.3.1
1.2.3.8
Pyridoxamine
2.7.1.35
3.1.3.74
2.6.1.30
2.6.1.54
2.6.1.31
1.4.3.5
Pyridoxamin 5-phosphate
2-Oxoglutarate
Pyruvate
D-Glutamate
D-Alanine
Pyridoxal
Pyridoxal 5-phosphate
4.3.3.6
1.1.1.65
1.1.3.12
Glyceraldehyde 3-phosphate
Ribulose 5-phosphate
Glycolysis
Glyoxylate and dicarboxylate metabolism
Glycolaldehyde
Pyridoxine
Pyridoxine phosphate
Pentose phosphate pathway
2.6.99.2
1.1.99.9
2-(Acetamidomethylene)-3-(hydroxymethyl) 3-succinate
5-Pyridoxolactone
3.5.1.66
1.14.12.5
α-Hydroxymethyl succinate semialdehyde
3-Hydroxy-4-hydroxymethyl-2-methylpyridine-5-carboxylate
Isopyridoxal
3-Amino-2-oxopropyl phosphate
spontaneous
(2S)-2-Amino-3-oxo-4-phosphono butanoate
1.1.1.262
4-Phospho-D-erythronate
D-Erythrose 4-phosphate
1.2.1.72
1.1.1.290
2.6.1.52
4.2.3.1
2-Oxo-3-hydroxy-4-phosphobutanoate
O-Phospho-4-hydroxy-L-threonine
4-Hydroxy-L-threonine
Pentose phosphate pathway
00750 1/29/14
(c) Kanehisa Laboratories

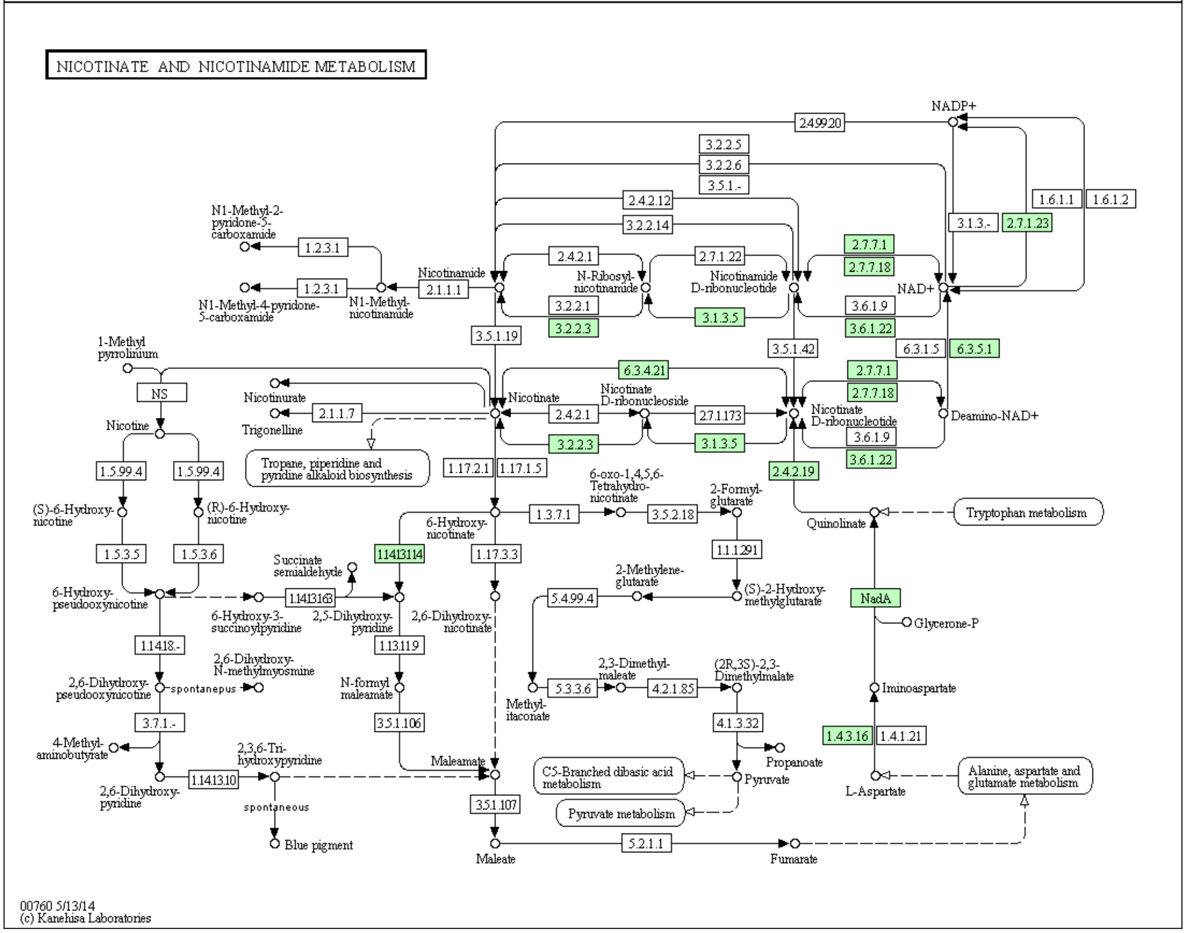
NICOTINATE AND NICOTINAMIDE METABOLISM
NADP+
2.4.99.20
3.2.2.5
3.2.2.6
3.5.1.-
2.4.2.12
3.2.2.14
1.6.1.1
1.6.1.2
3.1.3.-
2.7.1.23
N1-Methyl-2-pyridone-5-carboxamide
1.2.3.1
1.2.3.1
N1-Methyl-4-pyridone-5-carboxamide
N1-Methyl-nicotinamide
Nicotinamide
2.1.1.1
2.4.2.1
N-Ribosyl-nicotinamide
3.2.2.1
3.2.2.3
2.7.1.22
Nicotinamide D-ribonucleotide
3.1.3.5
2.7.7.1
2.7.7.18
NAD+
3.6.1.9
3.6.1.22
3.5.1.19
3.5.1.42
6.3.1.5
6.3.5.1
1-Methyl pyrrolinium
NS
6.3.4.21
Nicotinurate
Trigonelline
2.1.1.7
Nicotinate
2.4.2.1
Nicotinate D-ribonucleoside
2.7.1.173
Nicotinate D-ribonucleotide
Deamino-NAD+
Nicotine
Tropane, piperidine and pyridine alkaloid biosynthesis
3.2.2.3
3.1.3.5
2.4.2.19
1.5.99.4
1.5.99.4
1.17.2.1
1.17.1.5
(S)-6-Hydroxy-nicotine
(R)-6-Hydroxy-nicotine
6-oxo-1,4,5,6-Tetrahydro-nicotinate
2-Formyl-glutarate
1.3.7.1
3.5.2.18
Quinolinate
Tryptophan metabolism
1.5.3.5
1.5.3.6
Succinate semialdehyde
6-Hydroxy-nicotinate
1.14.13.114
1.17.3.3
1.1.1.291
6-Hydroxy-pseudooxynicotine
1.14.13.163
6-Hydroxy-3-succinoylpyridine
2,5-Dihydroxy-pyridine
2,6-Dihydroxy-nicotinate
2-Methylene-glutarate
5.4.99.4
(S)-2-Hydroxy-methylglutarate
NadA
Glycerone-P
1.14.18.-
2,6-Dihydroxy-N-methylmyosmine
2,6-Dihydroxy-pseudooxynicotine
spontaneous
1.13.11.9
N-formyl maleamate
2,3-Dimethyl-maleate
(2R,3S)-2,3-Dimethylmalate
Iminoaspartate
Methyl-itaconate
5.3.3.6
4.2.1.85
3.7.1.-
4-Methyl-aminobutyrate
3.5.1.106
4.1.3.32
1.4.3.16
1.4.1.21
2,3,6-Tri-hydroxypyridine
Maleamate
Propanoate
2,6-Dihydroxy-pyridine
1.14.13.10
C5-Branched dibasic acid metabolism
Pyruvate
L-Aspartate
Alanine, aspartate and glutamate metabolism
spontaneous
3.5.1.107
Pyruvate metabolism
Blue pigment
Maleate
5.2.1.1
Fumarate
00760 5/13/14
(c) Kanehisa Laboratories

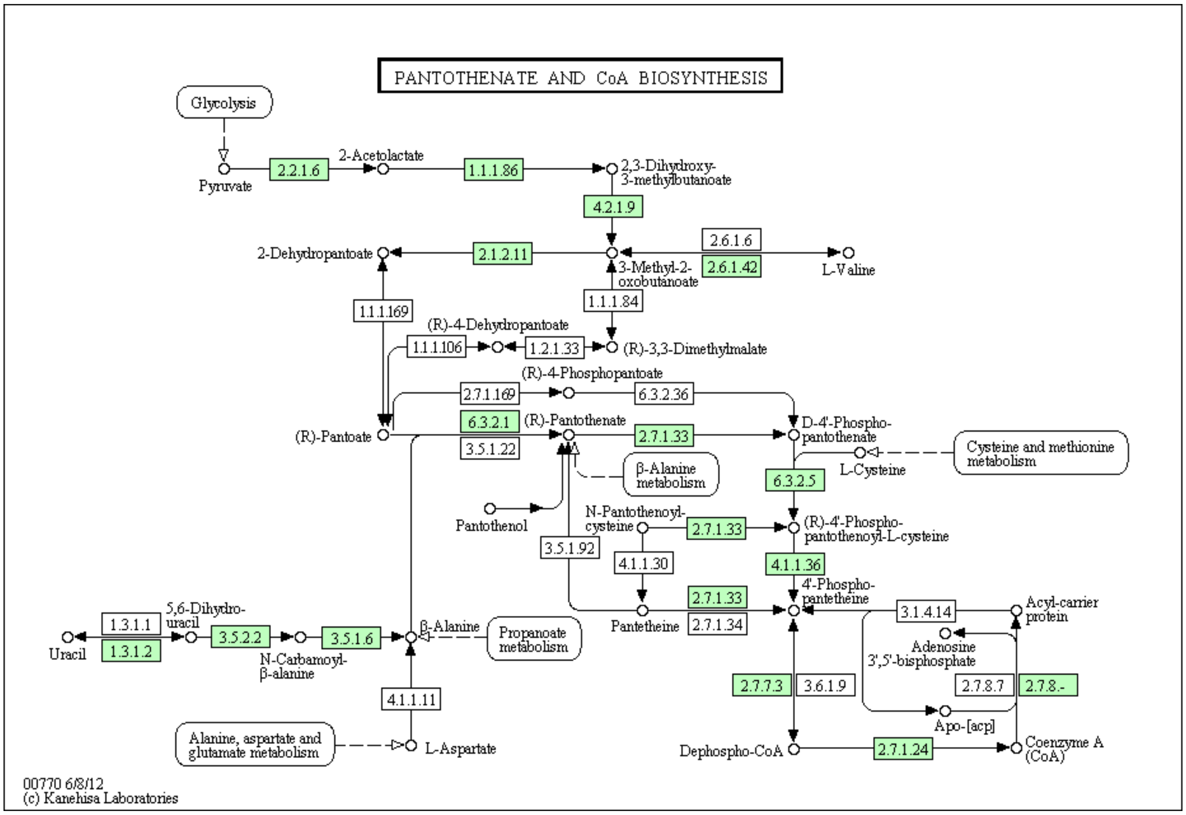
PANTOTHENATE AND CoA BIOSYNTHESIS
Glycolysis
Pyruvate
2.2.1.6
2-Acetolactate
1.1.1.86
2,3-Dihydroxy-
3-methylbutanoate
4.2.1.9
2-Dehydropantoate
2.1.2.11
2.6.1.6
2.6.1.42
L-Valine
3-Methyl-2-
oxobutanoate
1.1.1.84
1.1.1.169
(R)-4-Dehydropantoate
1.1.1.106
1.2.1.33
(R)-3,3-Dimethylmalate
(R)-4-Phosphopantoate
2.7.1.169
6.3.2.36
(R)-Pantoate
6.3.2.1
3.5.1.22
(R)-Pantothenate
2.7.1.33
D-4'-Phospho-
pantothenate
Cysteine and methionine
metabolism
L-Cysteine
β-Alanine
metabolism
6.3.2.5
Pantothenol
N-Pantothenoyl-
cysteine
2.7.1.33
(R)-4'-Phospho-
pantothenoyl-L-cysteine
3.5.1.92
4.1.1.30
4.1.1.36
2.7.1.33
4'-Phospho-
pantetheine
Pantetheine
2.7.1.34
3.1.4.14
Acyl-carrier
protein
1.3.1.1
1.3.1.2
5,6-Dihydro-
uracil
Uracil
3.5.2.2
3.5.1.6
N-Carbamoyl-
β-alanine
β-Alanine
Propanoate
metabolism
Adenosine
3',5'-bisphosphate
2.7.7.3
3.6.1.9
2.7.8.7
2.7.8.-
4.1.1.11
Apo-[acp]
Alanine, aspartate and
glutamate metabolism
L-Aspartate
Dephospho-CoA
2.7.1.24
Coenzyme A
(CoA)
00770 6/8/12
(c) Kanehisa Laboratories

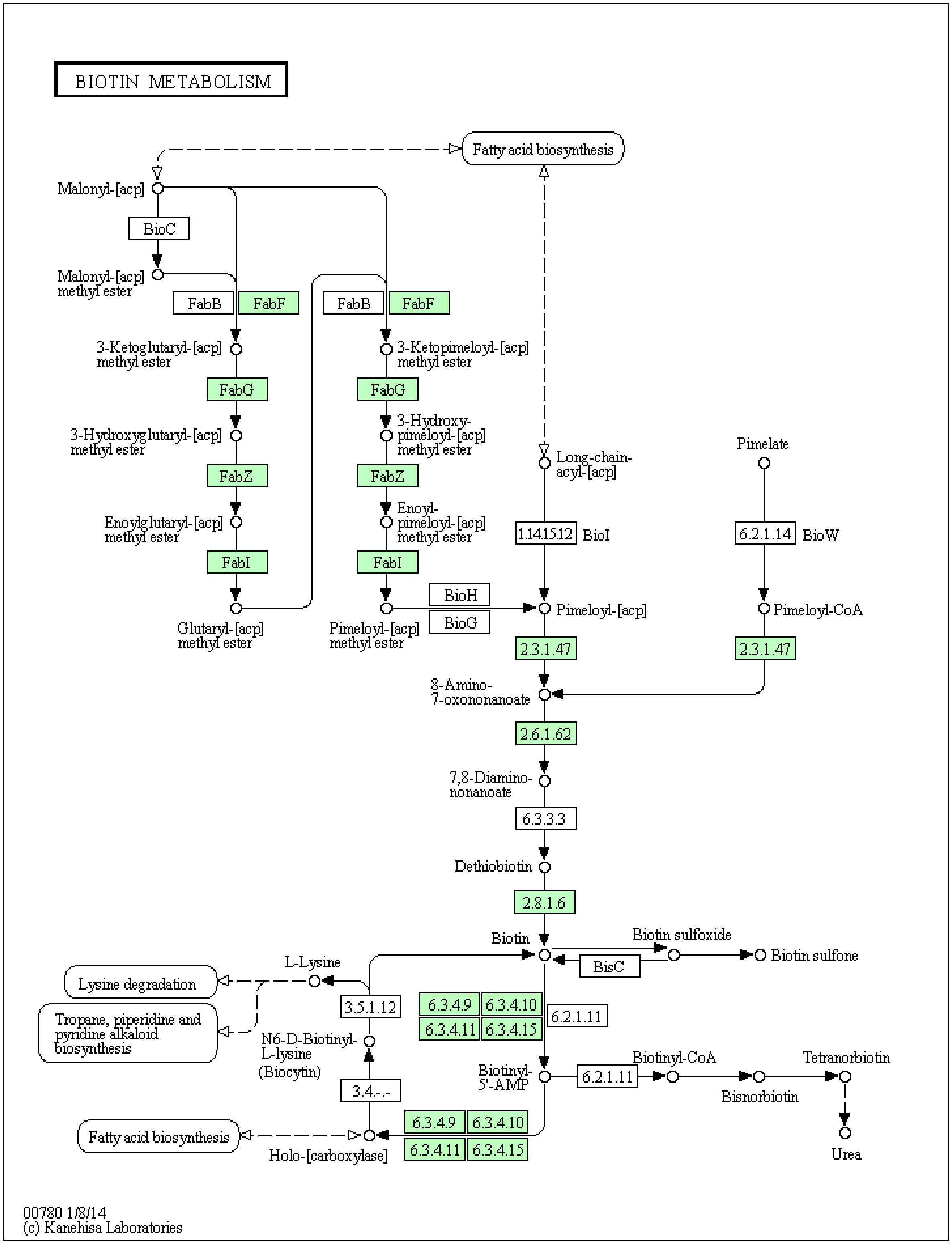
BIOTIN METABOLISM
Fatty acid biosynthesis
Malonyl-[acp]
BioC
Malonyl-[acp] methyl ester
FabB
FabF
FabB
FabF
3-Ketoglutaryl-[acp] methyl ester
3-Ketopimeloyl-[acp] methyl ester
FabG
FabG
3-Hydroxyglutaryl-[acp] methyl ester
3-Hydroxy-pimeloyl-[acp] methyl ester
FabZ
FabZ
Enoylglutaryl-[acp] methyl ester
Enoyl-pimeloyl-[acp] methyl ester
FabI
FabI
Glutaryl-[acp] methyl ester
Pimeloyl-[acp] methyl ester
BioH
BioG
Long-chain-acyl-[acp]
1.14.15.12
BioI
Pimelate
6.2.1.14
BioW
Pimeloyl-[acp]
Pimeloyl-CoA
2.3.1.47
2.3.1.47
8-Amino-7-oxononanoate
2.6.1.62
7,8-Diamino-nonanoate
6.3.3.3
Dethiobiotin
2.8.1.6
Biotin
Biotin sulfoxide
Biotin sulfone
BisC
L-Lysine
Lysine degradation
Tropane, piperidine and pyridine alkaloid biosynthesis
3.5.1.12
6.3.4.9
6.3.4.10
6.3.4.11
6.3.4.15
6.2.1.11
N6-D-Biotinyl-L-lysine (Biocytin)
Biotinyl-5'-AMP
6.2.1.11
Biotinyl-CoA
Bisnorbiotin
Tetranorbiotin
Urea
3.4.-.-
Fatty acid biosynthesis
6.3.4.9
6.3.4.10
6.3.4.11
6.3.4.15
Holo-[carboxylase]
00780 1/8/14
(c) Kanehisa Laboratories

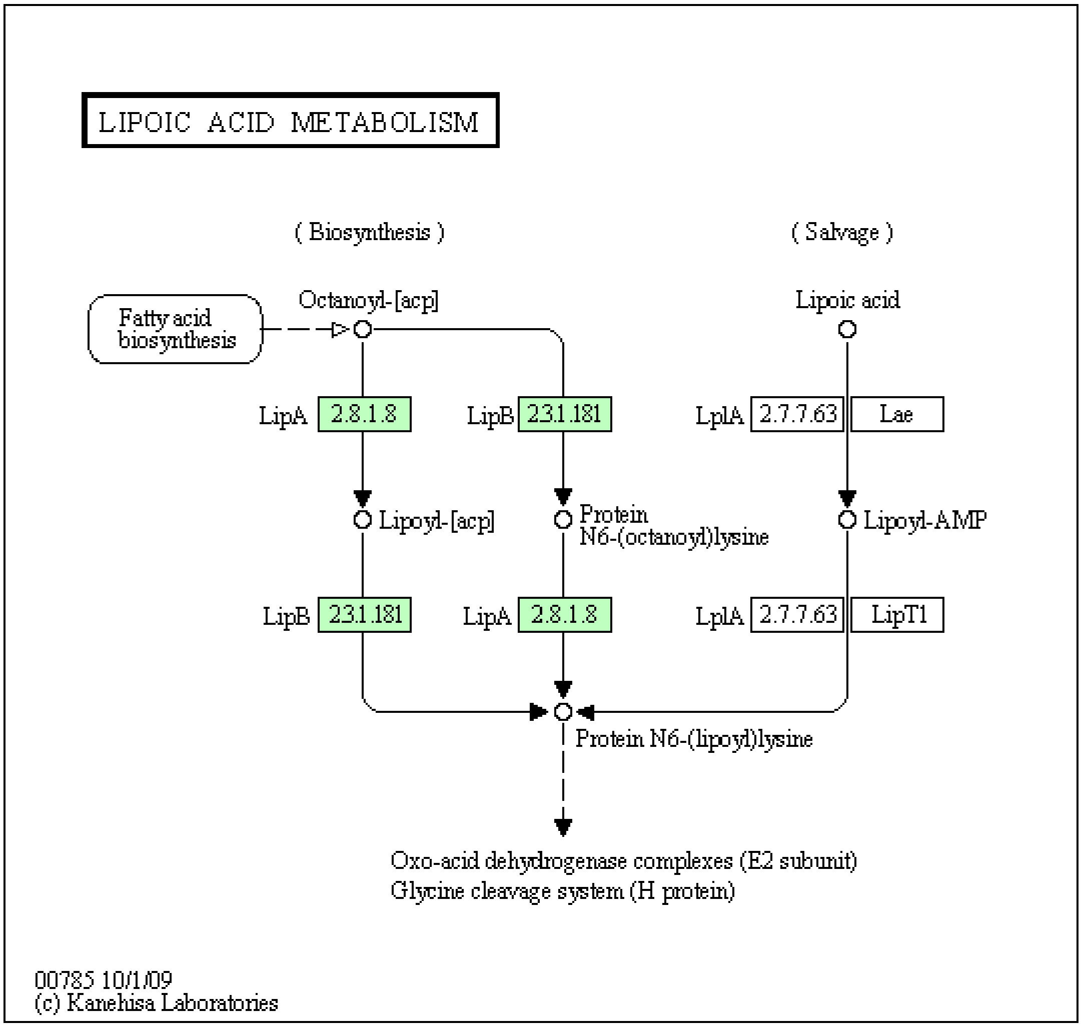
LIPOIC ACID METABOLISM
(Biosynthesis)
(Salvage)
Fatty acid biosynthesis
Octanoyl-[acp]
Lipoic acid
LipA
2.8.1.8
LipB
2.3.1.181
LplA
2.7.7.63
Lae
Lipoyl-[acp]
Protein N6-(octanoyl)lysine
Lipoyl-AMP
LipB
2.3.1.181
LipA
2.8.1.8
LplA
2.7.7.63
LipT1
Protein N6-(lipoyl)lysine
Oxo-acid dehydrogenase complexes (E2 subunit)
Glycine cleavage system (H protein)
00785 10/1/09
(c) Kanehisa Laboratories

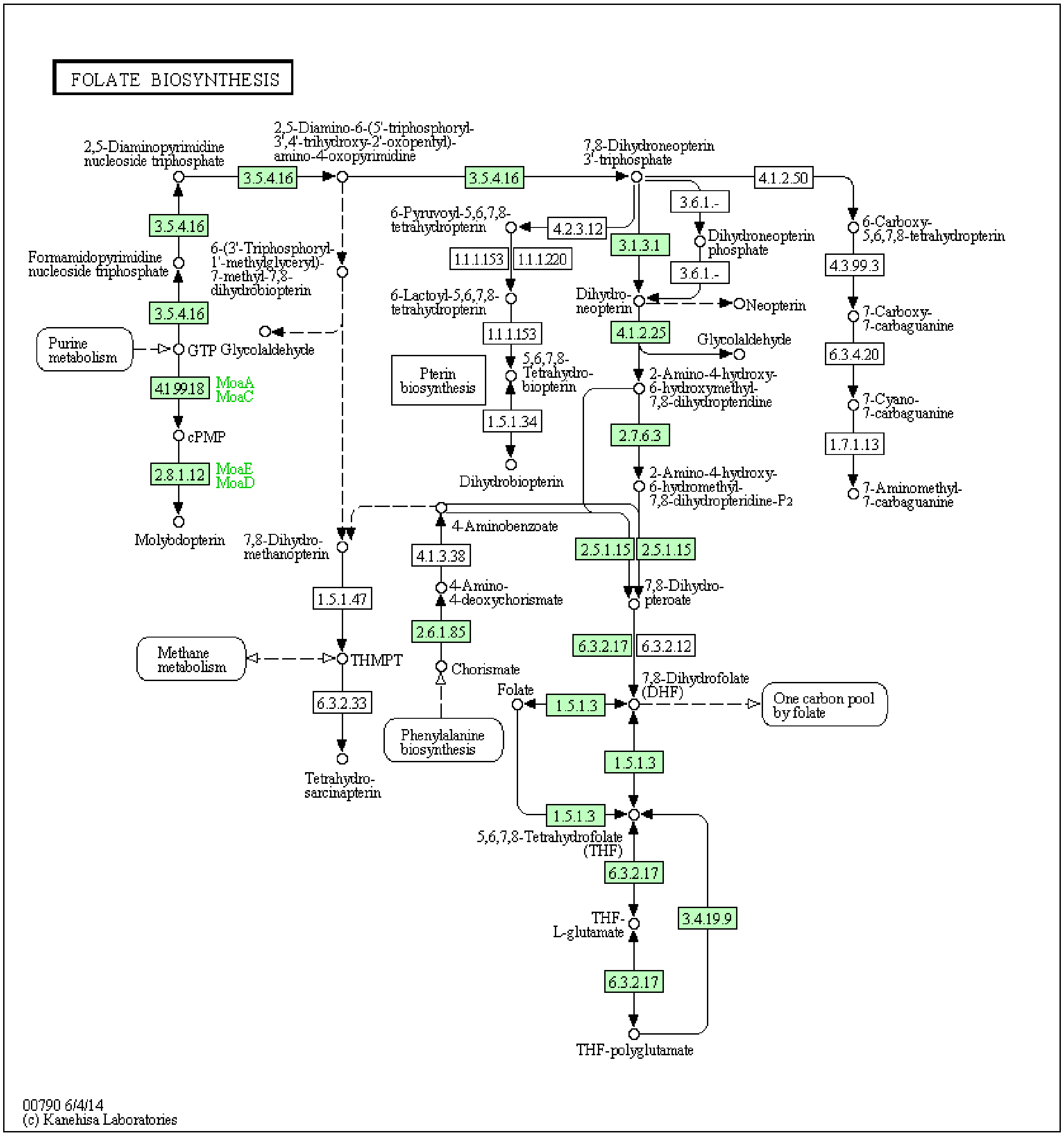
FOLATE BIOSYNTHESIS
2,5-Diaminopyrimidine nucleoside triphosphate
2,5-Diamino-6-(5'-triphosphoryl-3',4'-trihydroxy-2'-oxopentyl)-amino-4-oxopyrimidine
7,8-Dihydroneopterin 3'-triphosphate
3.5.4.16
4.1.2.50
3.6.1.-
6-Carboxy-5,6,7,8-tetrahydropterin
6-Pyruvoyl-5,6,7,8-tetrahydropterin
4.2.3.12
3.1.3.1
Dihydroneopterin phosphate
Formamidopyrimidine nucleoside triphosphate
6-(3'-Triphosphoryl-1'-methylglyceryl)-7-methyl-7,8-dihydrobiopterin
1.1.1.153
1.1.1.220
4.3.99.3
6-Lactoyl-5,6,7,8-tetrahydropterin
Dihydro-neopterin
Neopterin
7-Carboxy-7-carbaguanine
Purine metabolism
GTP
Glycolaldehyde
4.1.2.25
5,6,7,8-Tetrahydro-biopterin
6.3.4.20
Pterin biosynthesis
2-Amino-4-hydroxy-6-hydroxymethyl-7,8-dihydropteridine
4.1.99.18
MoaA
MoaC
7-Cyano-7-carbaguanine
cPMP
1.5.1.34
2.7.6.3
1.7.1.13
2.8.1.12
MoaE
MoaD
Dihydrobiopterin
2-Amino-4-hydroxy-6-hydromethyl-7,8-dihydropteridine-P2
7-Aminomethyl-7-carbaguanine
4-Aminobenzoate
Molybdopterin
7,8-Dihydro-methanopterin
4.1.3.38
2.5.1.15
7,8-Dihydro-pteroate
4-Amino-4-deoxychorismate
1.5.1.47
2.6.1.85
6.3.2.17
6.3.2.12
Methane metabolism
THMPT
Chorismate
7,8-Dihydrofolate (DHF)
Folate
1.5.1.3
One carbon pool by folate
6.3.2.33
Phenylalanine biosynthesis
Tetrahydro-sarcinapterin
5,6,7,8-Tetrahydrofolate (THF)
3.4.19.9
THF-L-glutamate
THF-polyglutamate
00790 6/4/14
(c) Kanehisa Laboratories

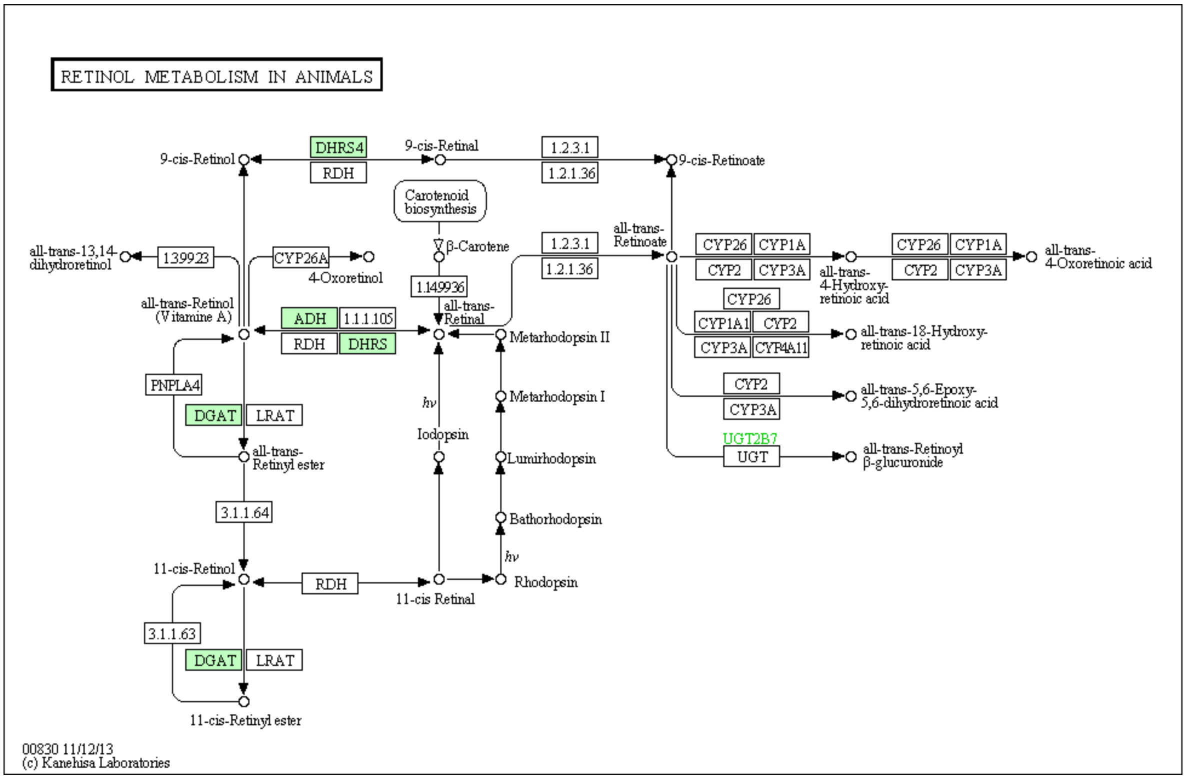
RETINOL METABOLISM IN ANIMALS
9-cis-Retinol
DHRS4
RDH
9-cis-Retinal
1.2.3.1
1.2.1.36
9-cis-Retinoate
Carotenoid biosynthesis
β-Carotene
1.14.99.36
all-trans-Retinal
all-trans-13,14-dihydroretinol
1.3.99.23
CYP26A
4-Oxoretinol
all-trans-Retinol (Vitamine A)
ADH
1.1.1.105
DHRS
PNPLA4
DGAT
LRAT
all-trans-Retinyl ester
3.1.1.64
11-cis-Retinol
11-cis Retinal
3.1.1.63
11-cis-Retinyl ester
hν
Iodopsin
Metarhodopsin II
Metarhodopsin I
Lumirhodopsin
Bathorhodopsin
Rhodopsin
all-trans-Retinoate
CYP26
CYP1A
CYP2
CYP3A
all-trans-4-Hydroxy-retinoic acid
all-trans-4-Oxoretinoic acid
CYP1A1
CYP4A11
all-trans-18-Hydroxy-retinoic acid
all-trans-5,6-Epoxy-5,6-dihydroretinoic acid
UGT2B7
UGT
all-trans-Retinoyl β-glucuronide
00830 11/12/13
(c) Kanehisa Laboratories

PORPHYRIN AND CHLOROPHYLL METABOLISM

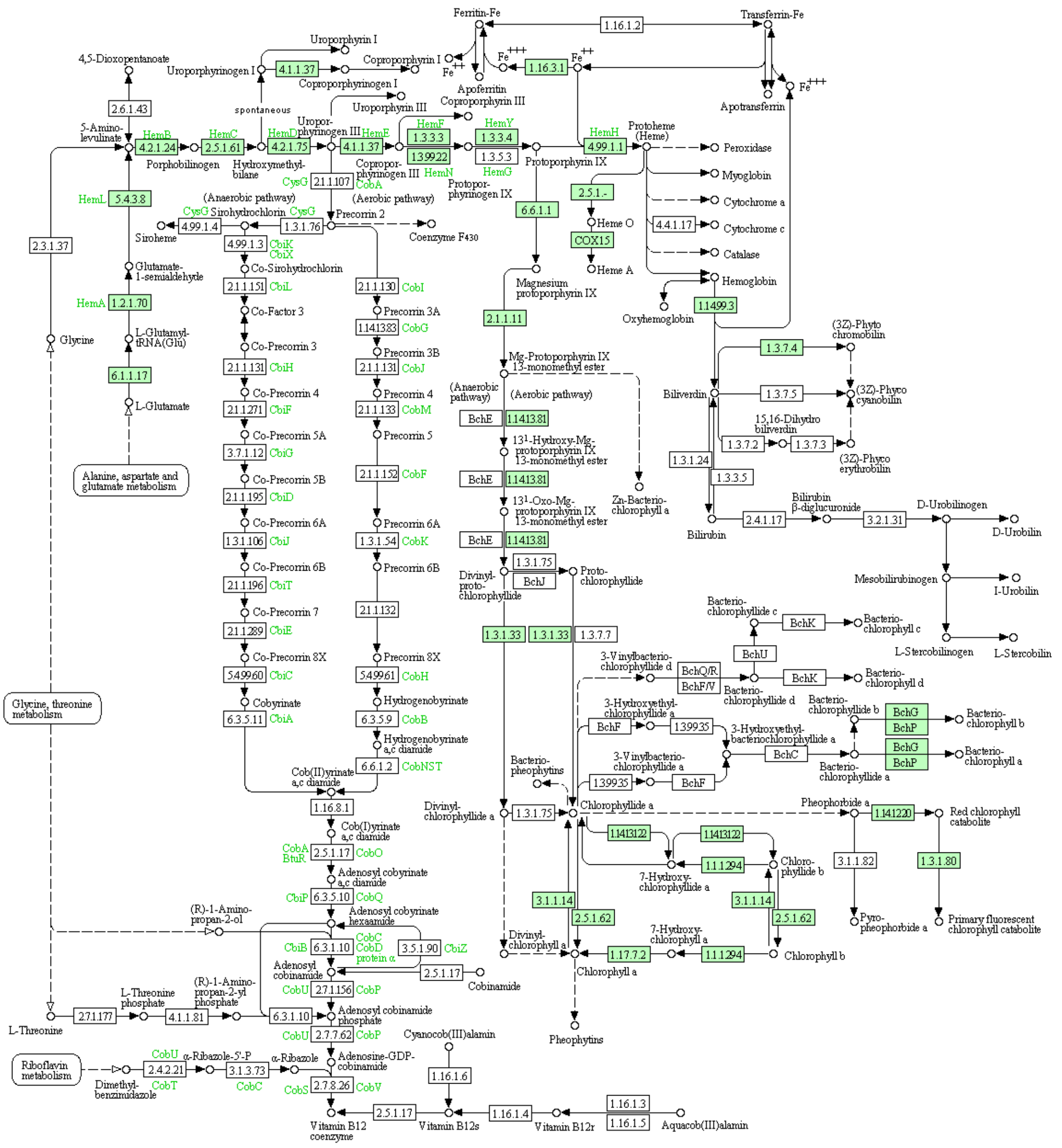

00860 4/14/14

(c) Kanehisa Laboratories

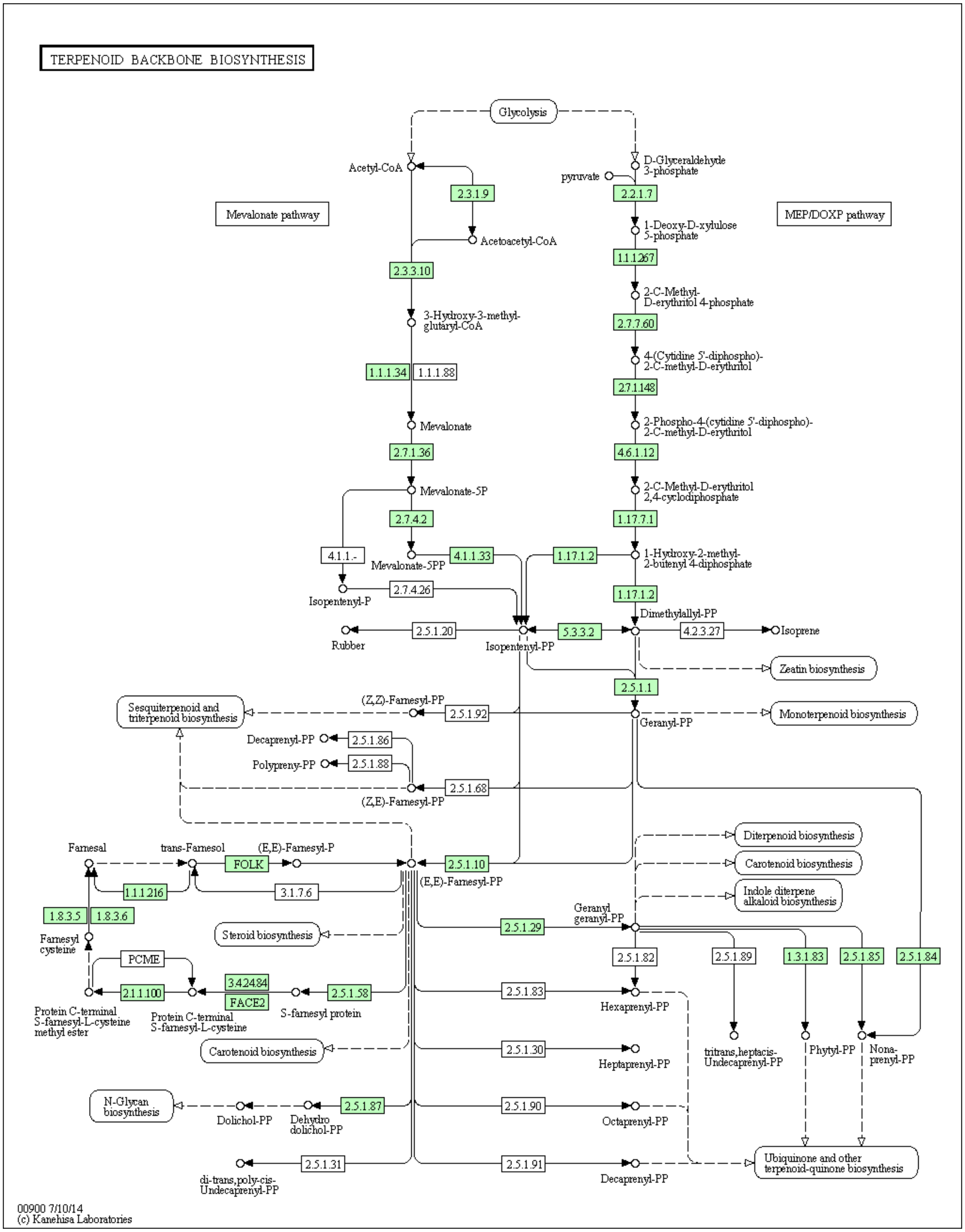
TERPENOID BACKBONE BIOSYNTHESIS
Glycolysis
Acetyl-CoA
D-Glyceraldehyde 3-phosphate
pyruvate
2.3.1.9
2.2.1.7
Mevalonate pathway
MEP/DOXP pathway
Acetoacetyl-CoA
1-Deoxy-D-xylulose 5-phosphate
2.3.3.10
1.1.1.267
3-Hydroxy-3-methyl-glutaryl-CoA
2-C-Methyl-D-erythritol 4-phosphate
2.7.7.60
1.1.1.34
1.1.1.88
4-(Cytidine 5'-diphospho)-2-C-methyl-D-erythritol
2.7.1.148
Mevalonate
2-Phospho-4-(cytidine 5'-diphospho)-2-C-methyl-D-erythritol
2.7.1.36
4.6.1.12
Mevalonate-5P
2-C-Methyl-D-erythritol 2,4-cyclodiphosphate
2.7.4.2
1.17.7.1
4.1.1.-
4.1.1.33
1.17.1.2
Mevalonate-5PP
1-Hydroxy-2-methyl-2-butenyl 4-diphosphate
2.7.4.26
Isopentenyl-P
1.17.1.2
Dimethylallyl-PP
2.5.1.20
5.3.3.2
4.2.3.27
Isoprene
Rubber
Isopentenyl-PP
Zeatin biosynthesis
2.5.1.1
Sesquiterpenoid and triterpenoid biosynthesis
(Z,Z)-Farnesyl-PP
2.5.1.92
Geranyl-PP
Monoterpenoid biosynthesis
Decaprenyl-PP
2.5.1.86
Polypreny-PP
2.5.1.88
2.5.1.68
(Z,E)-Farnesyl-PP
Diterpenoid biosynthesis
Farnesal
trans-Farnesol
(E,E)-Farnesyl-P
FOLK
2.5.1.10
Carotenoid biosynthesis
(E,E)-Farnesyl-PP
1.1.1.216
3.1.7.6
Indole diterpene alkaloid biosynthesis
1.8.3.5
1.8.3.6
Geranyl geranyl-PP
Steroid biosynthesis
2.5.1.29
Farnesyl cysteine
PCME
2.5.1.82
2.5.1.89
1.3.1.83
2.5.1.85
2.5.1.84
3.4.24.84
2.1.1.100
2.5.1.58
2.5.1.83
FACE2
Hexaprenyl-PP
Protein C-terminal S-farnesyl-L-cysteine methyl ester
Protein C-terminal S-farnesyl-L-cysteine
S-farnesyl protein
tritrans,heptacis-Undecaprenyl-PP
Phytyl-PP
Nona-prenyl-PP
Carotenoid biosynthesis
2.5.1.30
Heptaprenyl-PP
N-Glycan biosynthesis
2.5.1.87
2.5.1.90
Dolichol-PP
Dehydro dolichol-PP
Octaprenyl-PP
2.5.1.31
2.5.1.91
Ubiquinone and other terpenoid-quinone biosynthesis
di-trans,poly-cis-Undecaprenyl-PP
Decaprenyl-PP
00900 7/10/14
(c) Kanehisa Laboratories

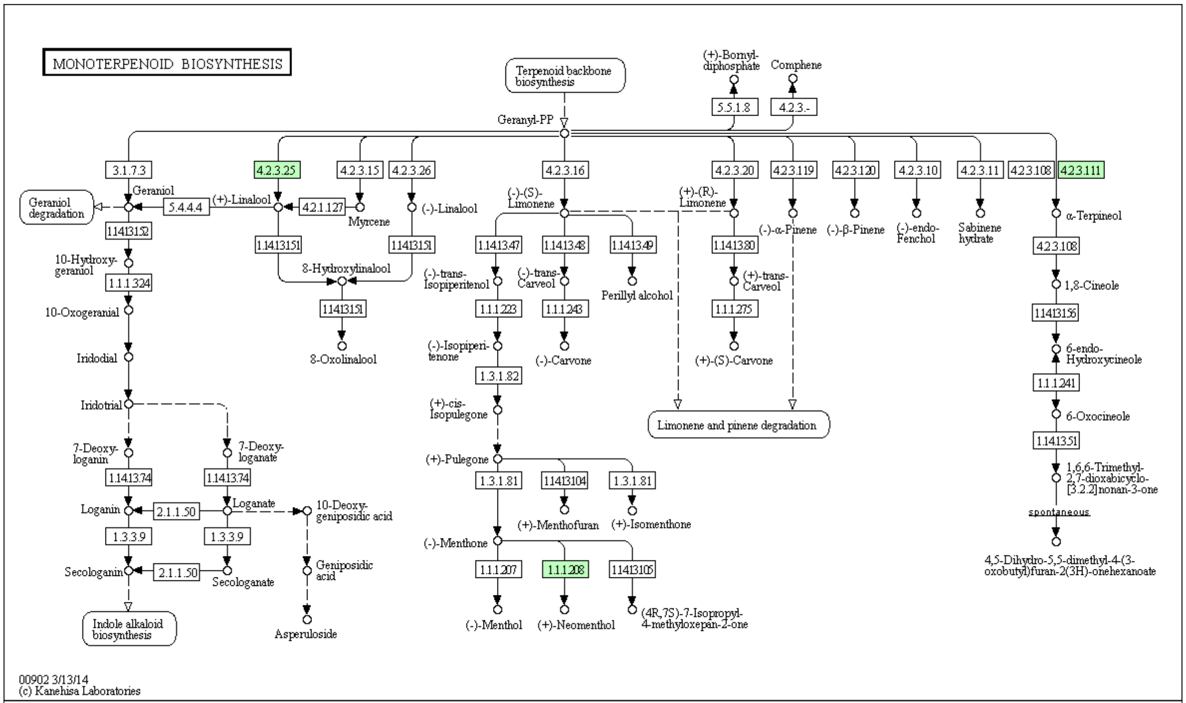
MONOTERPENOID BIOSYNTHESIS
Terpenoid backbone biosynthesis
Geranyl-PP
Geraniol degradation
Limonene and pinene degradation
Indole alkaloid biosynthesis
00902 3/13/14
(c) Kanehisa Laboratories

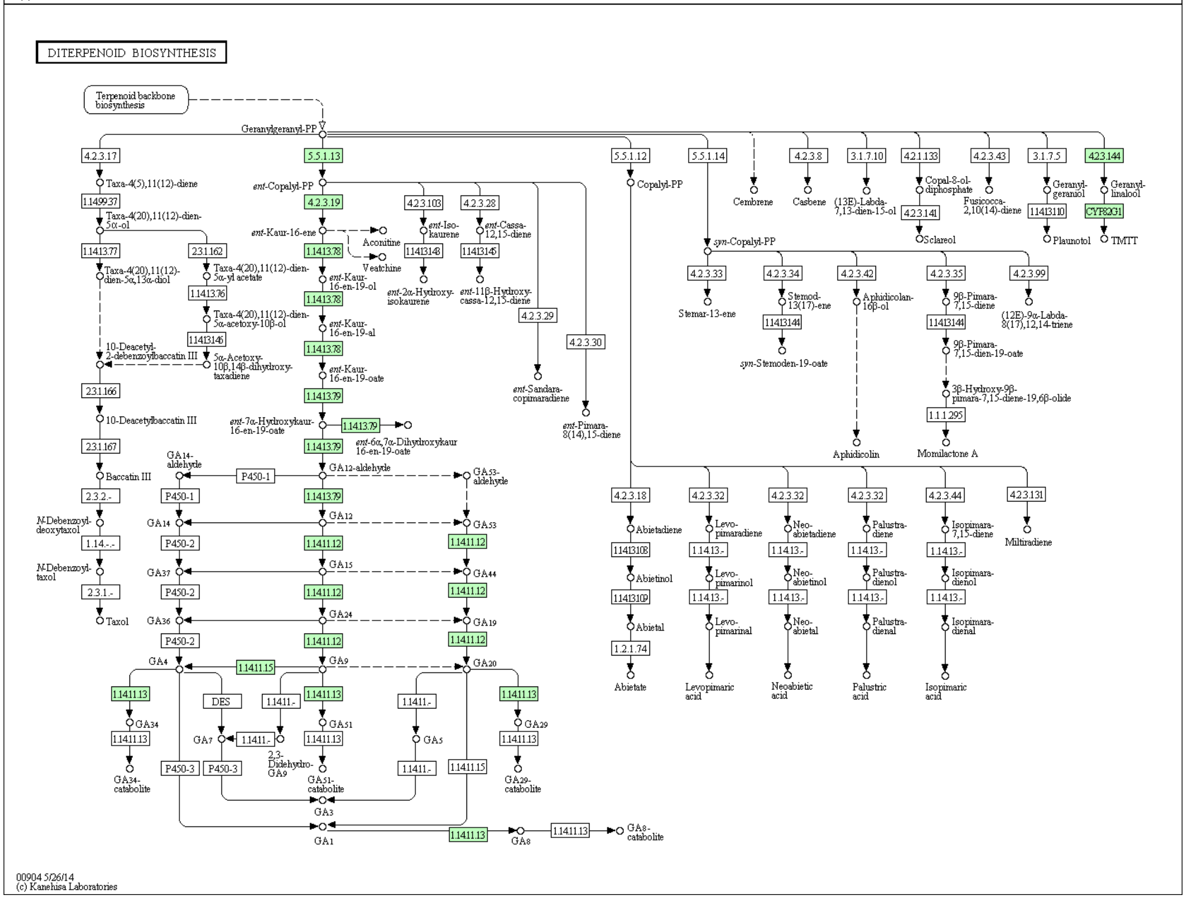
DITERPENOID BIOSYNTHESIS
Terpenoid backbone biosynthesis
Geranylgeranyl-PP
00904 5/26/14
(c) Kanehisa Laboratories

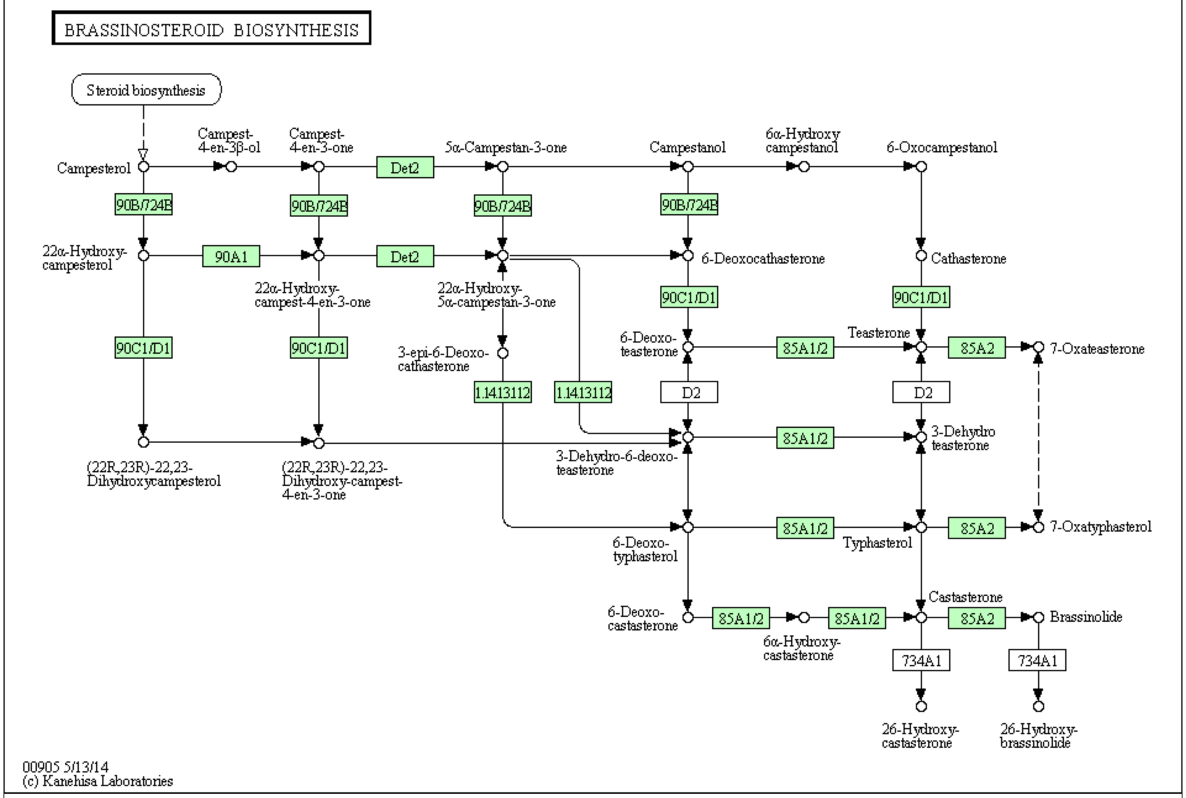
BRASSINOSTEROID BIOSYNTHESIS
Steroid biosynthesis
Campesterol
Campest-4-en-3β-ol
Campest-4-en-3-one
Det2
5α-Campestan-3-one
Campestanol
6α-Hydroxy campestanol
6-Oxocampestanol
90B/724B
22α-Hydroxy-campesterol
90A1
22α-Hydroxy-campest-4-en-3-one
22α-Hydroxy-5α-campestan-3-one
6-Deoxocathasterone
Cathasterone
90C1/D1
3-epi-6-Deoxo-cathasterone
6-Deoxo-teasterone
85A1/2
Teasterone
85A2
7-Oxateasterone
1.14.13.112
D2
(22R,23R)-22,23-Dihydroxycampesterol
(22R,23R)-22,23-Dihydroxy-campest-4-en-3-one
3-Dehydro-6-deoxo-teasterone
3-Dehydro teasterone
6-Deoxo-typhasterol
Typhasterol
7-Oxatyphasterol
6-Deoxo-castasterone
6α-Hydroxy-castasterone
Castasterone
Brassinolide
734A1
26-Hydroxy-castasterone
26-Hydroxy-brassinolide
00905 5/13/14
(c) Kanehisa Laboratories

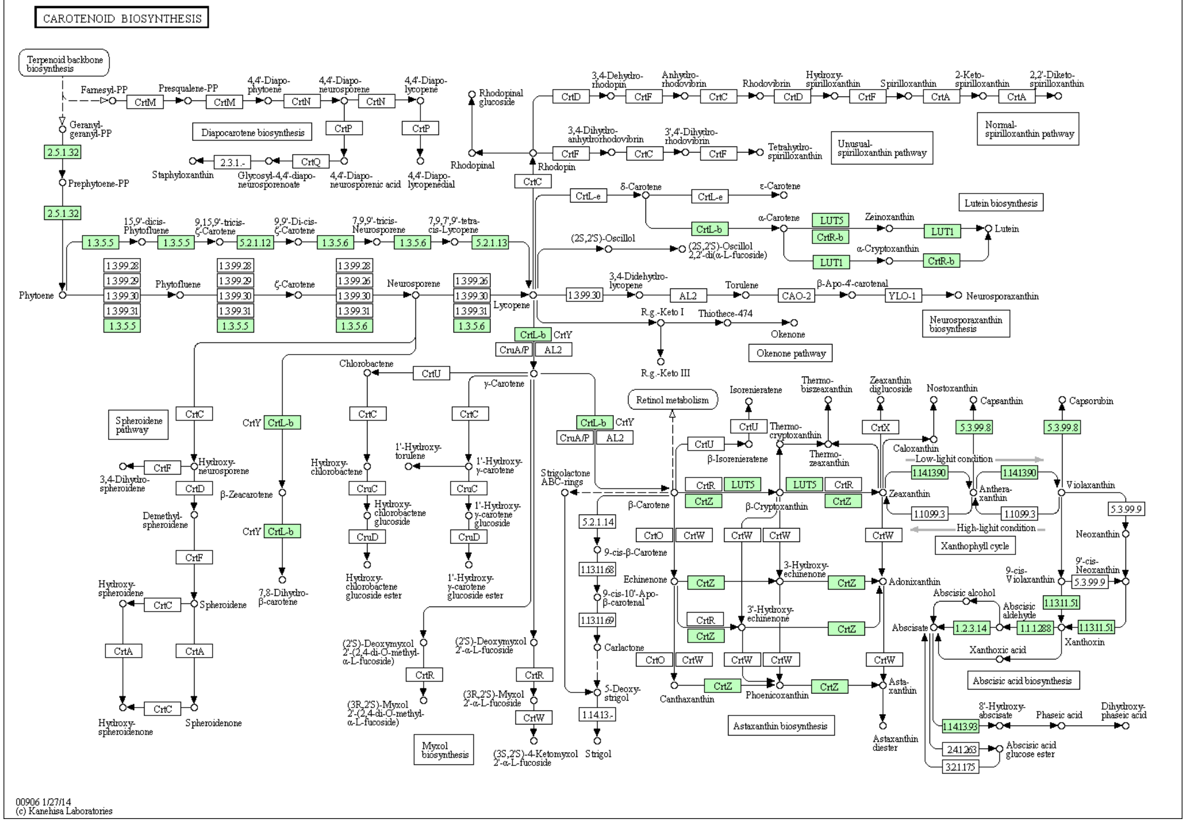
CAROTENOID BIOSYNTHESIS
Terpenoid backbone biosynthesis
Farnesyl-PP
Presqualene-PP
4,4'-Diapo-phytoene
4,4'-Diapo-neurosporene
4,4'-Diapo-lycopene
CrtM
CrtN
CrtP
CrtQ
Geranyl-geranyl-PP
Diapocarotene biosynthesis
Staphyloxanthin
Glycosyl-4,4'-diapo-neurosporenoate
4,4'-Diapo-neurosporenic acid
4,4'-Diapo-lycopenedial
2.5.1.32
Prephytoene-PP
Phytoene
Phytofluene
ζ-Carotene
Neurosporene
Lycopene
1.3.5.5
1.3.5.6
5.2.1.12
5.2.1.13
1.3.99.26
1.3.99.28
1.3.99.29
1.3.99.30
1.3.99.31
Rhodopinal glucoside
Rhodopin
Rhodopinal
3,4-Dehydro-rhodopin
Anhydro-rhodovibrin
Rhodovibrin
Hydroxy-spirilloxanthin
Spirilloxanthin
2-Keto-spirilloxanthin
2,2'-Diketo-spirilloxanthin
CrtD
CrtF
CrtC
CrtA
Normal-spirilloxanthin pathway
Unusual-spirilloxanthin pathway
Tetrahydro-spirilloxanthin
CrtL-e
δ-Carotene
ε-Carotene
α-Carotene
Zeinoxanthin
Lutein
Lutein biosynthesis
LUT5
LUT1
CrtR-b
α-Cryptoxanthin
(2S,2'S)-Oscillol
(2S,2'S)-Oscillol 2,2'-di(α-L-fucoside)
3,4-Didehydro-lycopene
Torulene
β-Apo-4'-carotenal
Neurosporaxanthin
AL2
CAO-2
YLO-1
Neurosporaxanthin biosynthesis
R.g.-Keto I
Thiothece-474
Okenone
Okenone pathway
R.g.-Keto III
CrtL-b
CrtY
CruA/P
Chlorobactene
γ-Carotene
CrtU
Spheroidene pathway
Hydroxy-neurosporene
Spheroidene
Spheroidenone
Hydroxy-spheroidene
Hydroxy-spheroidenone
Demethyl-spheroidene
3,4-Dihydro-spheroidene
β-Zeacarotene
7,8-Dihydro-β-carotene
Hydroxy-chlorobactene
1'-Hydroxy-torulene
1'-Hydroxy-γ-carotene
CruC
CruD
Retinol metabolism
Isorenieratene
β-Isorenieratene
Thermo-biszeaxanthin
Thermo-cryptoxanthin
Thermo-zeaxanthin
Zeaxanthin diglucoside
Nostoxanthin
Caloxanthin
Capsanthin
Capsorubin
5.3.99.8
Strigolactone ABC-rings
β-Carotene
β-Cryptoxanthin
Zeaxanthin
Antheraxanthin
Violaxanthin
Low-light condition
High-light condition
1.14.13.90
1.10.99.3
Xanthophyll cycle
5.3.99.9
Neoxanthin
9'-cis-Neoxanthin
9-cis-Violaxanthin
Xanthoxin
Abscisic alcohol
Abscisic aldehyde
Xanthoxic acid
Abscisate
1.13.11.51
1.1.1.288
1.2.3.14
Abscisic acid biosynthesis
8'-Hydroxy-abscisate
Phaseic acid
Dihydroxy-phaseic acid
1.14.13.93
2.4.1.263
3.2.1.175
Abscisic acid glucose ester
5.2.1.14
9-cis-β-Carotene
1.13.11.68
9-cis-10'-Apo-β-carotenal
1.13.11.69
Carlactone
5-Deoxy-strigol
1.14.13.-
Strigol
Echinenone
3-Hydroxy-echinenone
3'-Hydroxy-echinenone
Adonixanthin
Canthaxanthin
Phoenicoxanthin
Astaxanthin
Astaxanthin diester
Astaxanthin biosynthesis
CrtO
CrtW
CrtZ
CrtR
CrtX
(2'S)-Deoxymyxol 2'-(2,4-di-O-methyl-α-L-fucoside)
(2'S)-Deoxymyxol 2'-α-L-fucoside
(3R,2'S)-Myxol 2'-(2,4-di-O-methyl-α-L-fucoside)
(3R,2'S)-Myxol 2'-α-L-fucoside
(3S,2'S)-4-Ketomyxol 2'-α-L-fucoside
Myxol biosynthesis
00906 1/27/14
(c) Kanehisa Laboratories

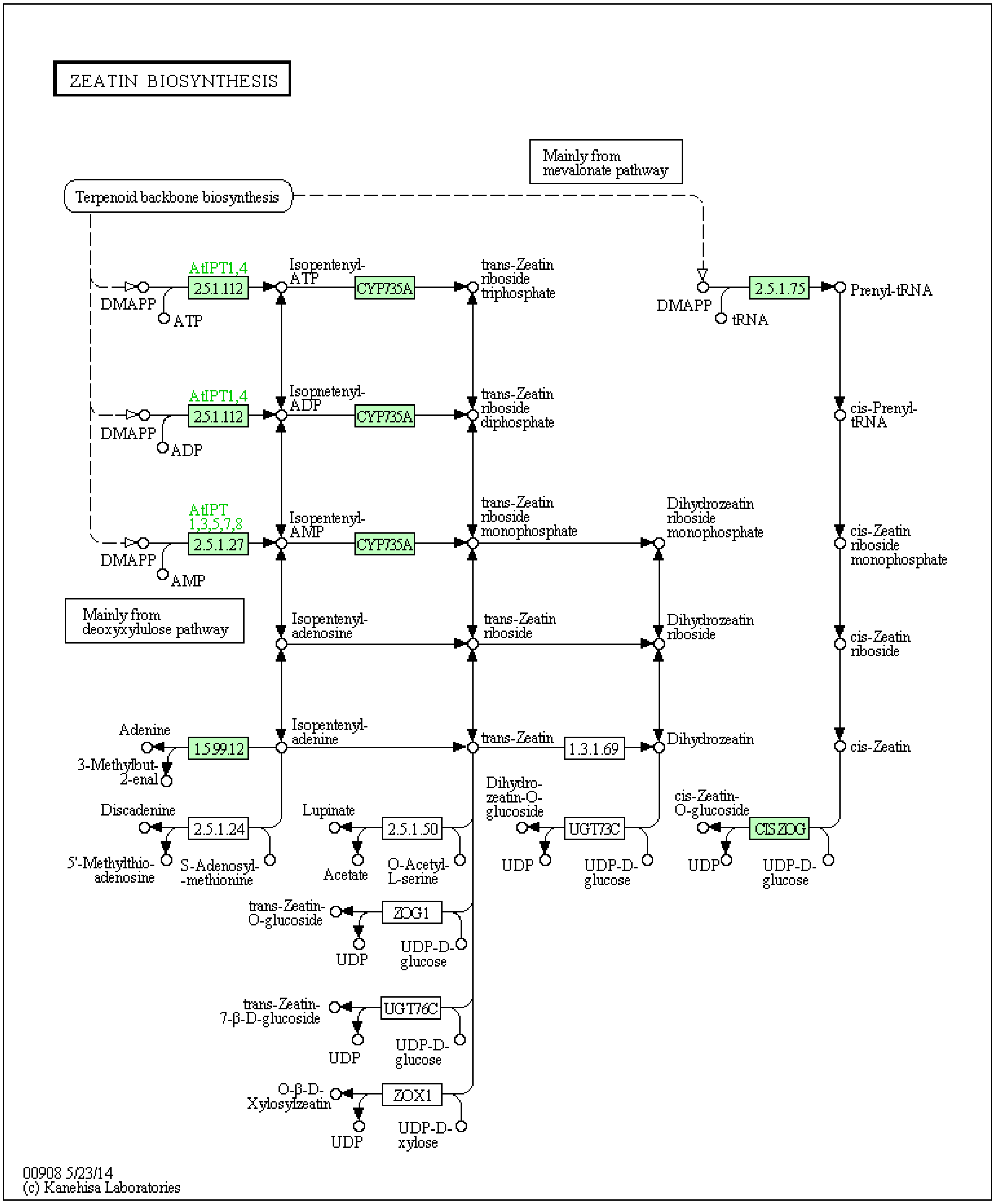

ZEATIN BIOSYNTHESIS
Mainly from mevalonate pathway
Terpenoid backbone biosynthesis
AtIPT1,4
2.5.1.112
DMAPP
ATP
Isopentenyl-ATP
CYP735A
trans-Zeatin riboside triphosphate
DMAPP
2.5.1.75
Prenyl-tRNA
tRNA
AtIPT1,4
2.5.1.112
DMAPP
ADP
Isopnetenyl-ADP
CYP735A
trans-Zeatin riboside diphosphate
cis-Prenyl-tRNA
AtIPT 1,3,5,7,8
2.5.1.27
DMAPP
AMP
Isopentenyl-AMP
CYP735A
trans-Zeatin riboside monophosphate
Dihydrozeatin riboside monophosphate
cis-Zeatin riboside monophosphate
Mainly from deoxyxylulose pathway
Isopentenyl-adenosine
trans-Zeatin riboside
Dihydrozeatin riboside
cis-Zeatin riboside
Adenine
1.5.99.12
3-Methylbut-2-enal
Isopentenyl-adenine
trans-Zeatin
1.3.1.69
Dihydrozeatin
cis-Zeatin
Discadenine
2.5.1.24
5'-Methylthio-adenosine
S-Adenosyl-methionine
Lupinate
2.5.1.50
Acetate
O-Acetyl-L-serine
Dihydro-zeatin-O-glucoside
UGT73C
UDP
UDP-D-glucose
cis-Zeatin-O-glucoside
CISZOG
UDP
UDP-D-glucose
trans-Zeatin-O-glucoside
ZOG1
UDP
UDP-D-glucose
trans-Zeatin-7-β-D-glucoside
UGT76C
UDP
UDP-D-glucose
O-β-D-Xylosylzeatin
ZOX1
UDP
UDP-D-xylose
00908 5/23/14
(c) Kanehisa Laboratories

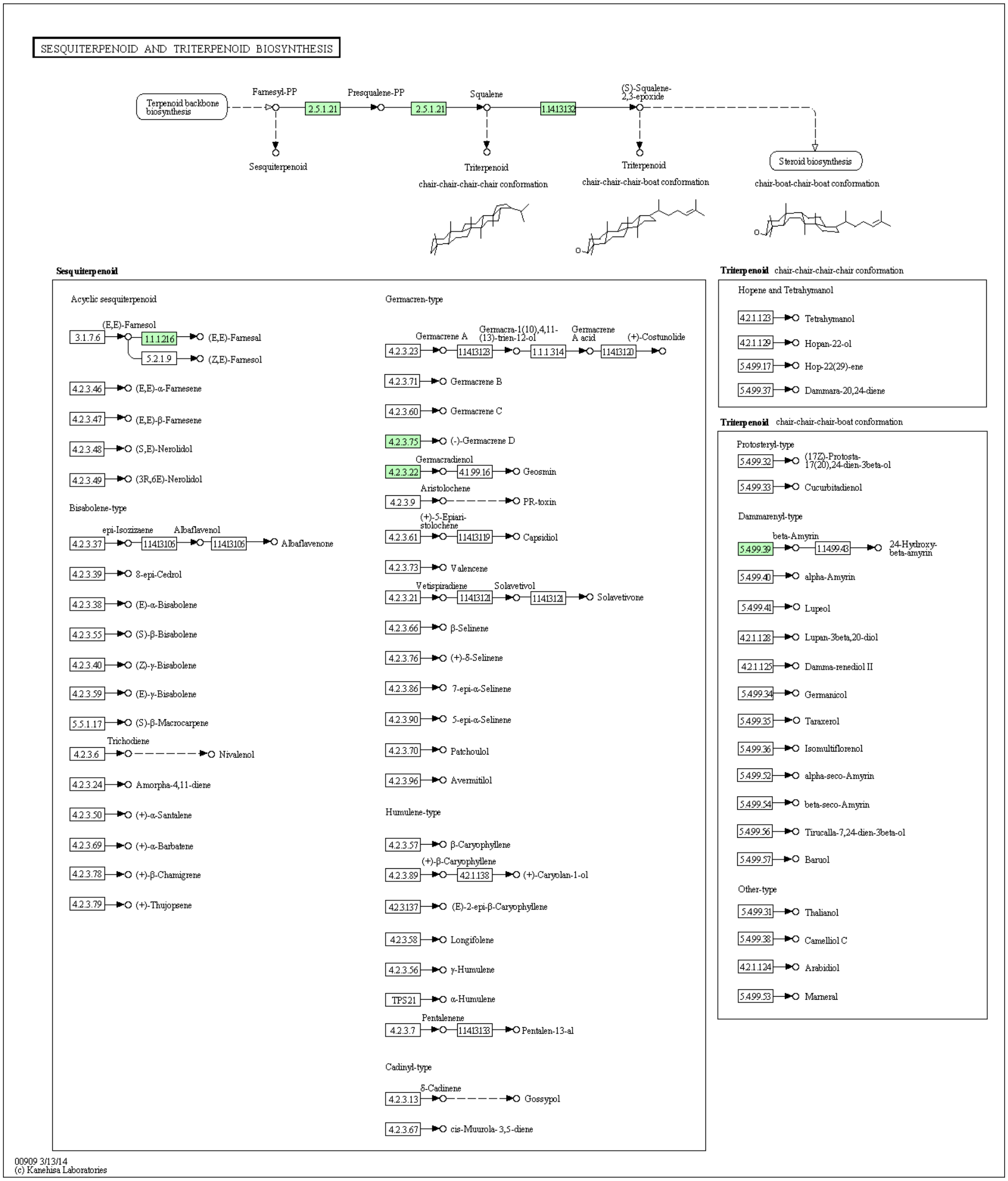

SESQUITERPENOID AND TRITERPENOID BIOSYNTHESIS
Terpenoid backbone biosynthesis
Farnesyl-PP
2.5.1.21
Presqualene-PP
2.5.1.21
Squalene
1.14.13.132
(S)-Squalene-2,3-epoxide
Sesquiterpenoid
Triterpenoid
chair-chair-chair-chair conformation
Triterpenoid
chair-chair-chair-boat conformation
Steroid biosynthesis
chair-boat-chair-boat conformation
Sesquiterpenoid
Acyclic sesquiterpenoid
(E,E)-Farnesol
3.1.7.6
1.1.1.216
(E,E)-Farnesal
5.2.1.9
(Z,E)-Farnesol
4.2.3.46
(E,E)-α-Farnesene
4.2.3.47
(E,E)-β-Farnesene
4.2.3.48
(S,E)-Nerolidol
4.2.3.49
(3R,6E)-Nerolidol
Bisabolene-type
epi-Isozizaene
Albaflavenol
4.2.3.37
1.14.13.106
1.14.13.106
Albaflavenone
4.2.3.39
8-epi-Cedrol
4.2.3.38
(E)-α-Bisabolene
4.2.3.55
(S)-β-Bisabolene
4.2.3.40
(Z)-γ-Bisabolene
4.2.3.59
(E)-γ-Bisabolene
5.5.1.17
(S)-β-Macrocarpene
Trichodiene
4.2.3.6
Nivalenol
4.2.3.24
Amorpha-4,11-diene
4.2.3.50
(+)-α-Santalene
4.2.3.69
(+)-α-Barbatene
4.2.3.78
(+)-β-Chamigrene
4.2.3.79
(+)-Thujopeene
Germacren-type
Germacrene A
Germacra-1(10),4,11-(13)-trien-12-ol
Germacrene A acid
(+)-Costunolide
4.2.3.23
1.14.13.123
1.1.1.314
1.14.13.120
4.2.3.71
Germacrene B
4.2.3.60
Germacrene C
4.2.3.75
(-)-Germacrene D
Germacradienol
4.2.3.22
4.1.99.16
Geosmin
Aristolochene
4.2.3.9
PR-toxin
(+)-5-Epiaristolochene
4.2.3.61
1.14.13.119
Capsidiol
4.2.3.73
Valencene
Vetispiradiene
Solavetivol
4.2.3.21
1.14.13.121
1.14.13.121
Solavetivone
4.2.3.66
β-Selinene
4.2.3.76
(+)-δ-Selinene
4.2.3.86
7-epi-α-Selinene
4.2.3.90
5-epi-α-Selinene
4.2.3.70
Patchoulol
4.2.3.96
Avermitilol
Humulene-type
4.2.3.57
β-Caryophyllene
(+)-β-Caryophyllene
4.2.3.89
4.2.1.138
(+)-Caryolan-1-ol
4.2.3.137
(E)-2-epi-β-Caryophyllene
4.2.3.58
Longifolene
4.2.3.56
γ-Humulene
TPS21
α-Humulene
Pentalenene
4.2.3.7
1.14.13.133
Pentalen-13-al
Cadinyl-type
δ-Cadinene
4.2.3.13
Gossypol
4.2.3.67
cis-Muurola-3,5-diene
Triterpenoid chair-chair-chair-chair conformation
Hopene and Tetrahymanol
4.2.1.123
Tetrahymanol
4.2.1.129
Hopan-22-ol
5.4.99.17
Hop-22(29)-ene
5.4.99.37
Dammara-20,24-diene
Triterpenoid chair-chair-chair-boat conformation
Protosteryl-type
5.4.99.32
(17Z)-Protosta-17(20),24-dien-3beta-ol
5.4.99.33
Cucurbitadienol
Dammarenyl-type
beta-Amyrin
5.4.99.39
1.14.99.43
24-Hydroxy-beta-amyrin
5.4.99.40
alpha-Amyrin
5.4.99.41
Lupeol
4.2.1.128
Lupan-3beta,20-diol
4.2.1.125
Dammarenediol II
5.4.99.34
Germanicol
5.4.99.35
Taraxerol
5.4.99.36
Isomultiflorenol
5.4.99.52
alpha-seco-Amyrin
5.4.99.54
beta-seco-Amyrin
5.4.99.56
Tirucalla-7,24-dien-3beta-ol
5.4.99.57
Baruol
Other-type
5.4.99.31
Thalianol
5.4.99.38
Camelliol C
4.2.1.124
Arabidiol
5.4.99.53
Marneral
00909 3/13/14
(c) Kanehisa Laboratories

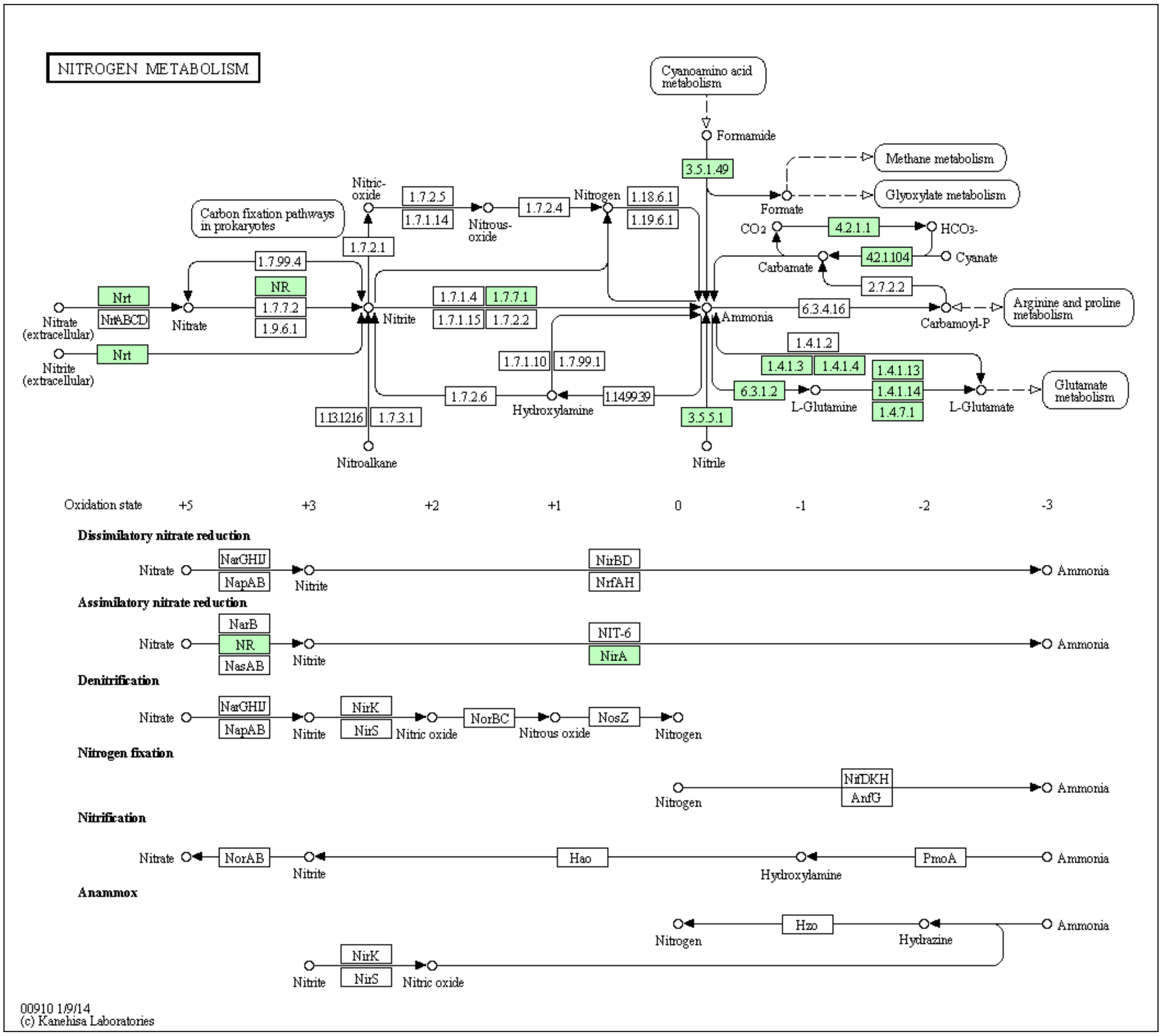
NITROGEN METABOLISM
Cyanoamino acid metabolism
Formamide
3.5.1.49
Methane metabolism
Glyoxylate metabolism
Formate
Carbon fixation pathways in prokaryotes
Nitric-oxide
1.7.2.5
1.7.1.14
Nitrous-oxide
1.7.2.4
Nitrogen
1.18.6.1
1.19.6.1
CO2
4.2.1.1
HCO3-
Carbamate
4.2.1.104
Cyanate
1.7.2.1
1.7.99.4
2.7.2.2
Nrt
NR
NrtABCD
1.7.7.2
1.9.6.1
Nitrate (extracellular)
Nitrate
Nitrite
1.7.1.4
1.7.7.1
1.7.1.15
1.7.2.2
Ammonia
6.3.4.16
Carbamoyl-P
Arginine and proline metabolism
Nitrite (extracellular)
1.4.1.2
1.4.1.3
1.4.1.4
1.4.1.13
1.4.1.14
1.4.7.1
1.7.1.10
1.7.99.1
6.3.1.2
L-Glutamine
L-Glutamate
Glutamate metabolism
1.7.2.6
Hydroxylamine
1.14.99.39
1.13.12.16
1.7.3.1
3.5.5.1
Nitroalkane
Nitrile
Oxidation state +5 +3 +2 +1 0 -1 -2 -3
Dissimilatory nitrate reduction
Nitrate
NarGHIJ
NapAB
Nitrite
NirBD
NrfAH
Ammonia
Assimilatory nitrate reduction
Nitrate
NarB
NR
NasAB
Nitrite
NIT-6
NirA
Ammonia
Denitrification
Nitrate
NarGHIJ
NapAB
Nitrite
NirK
NirS
Nitric oxide
NorBC
Nitrous oxide
NosZ
Nitrogen
Nitrogen fixation
Nitrogen
NifDKH
AnfG
Ammonia
Nitrification
Nitrate
NorAB
Nitrite
Hao
Hydroxylamine
PmoA
Ammonia
Anammox
Nitrogen
Hzo
Hydrazine
Ammonia
Nitrite
NirK
NirS
Nitric oxide
00910 1/9/14
(c) Kanehisa Laboratories

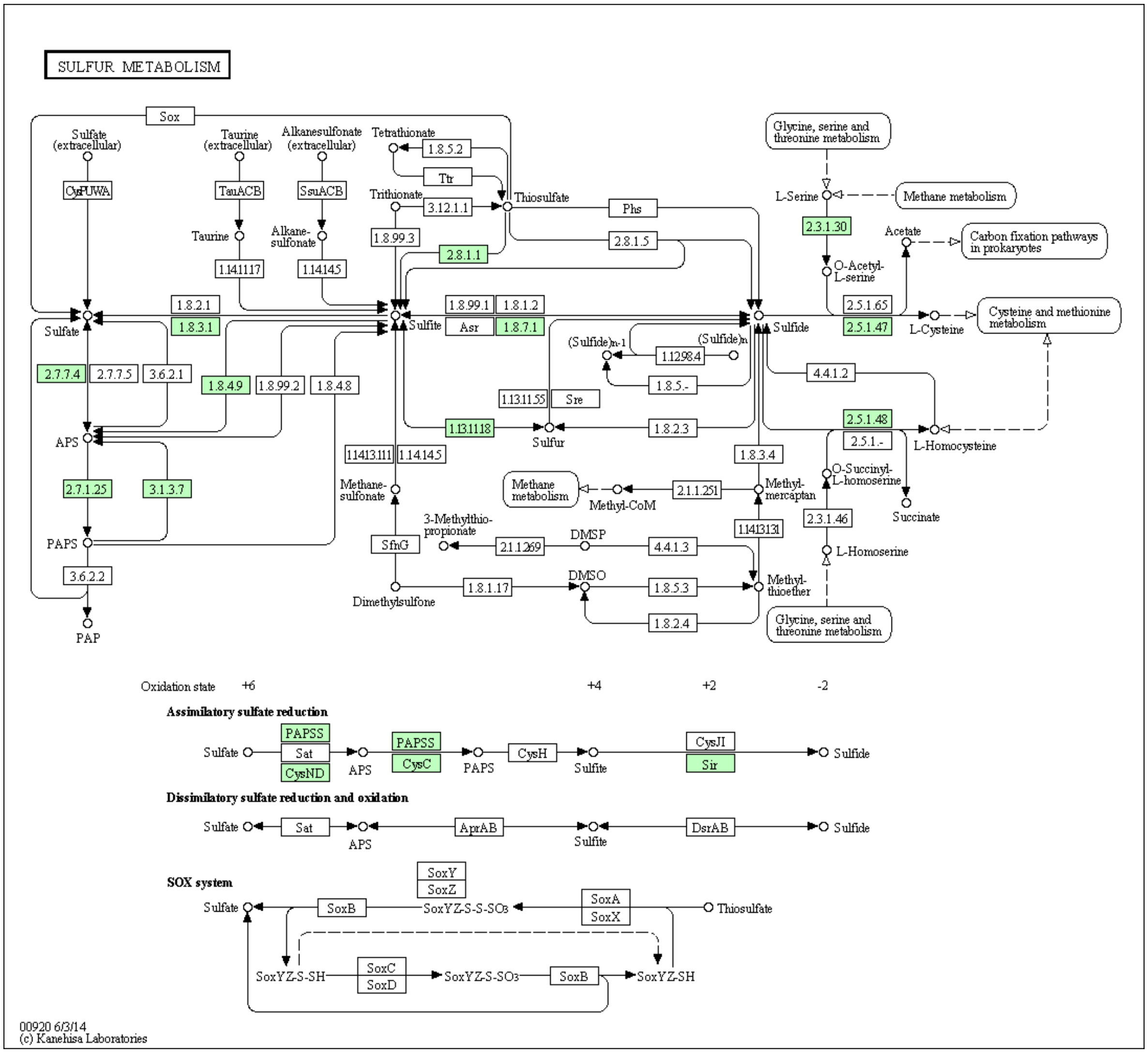
SULFUR METABOLISM
Sox
Sulfate (extracellular)
CysPUWA
Taurine (extracellular)
TauACB
Alkanesulfonate (extracellular)
SsuACB
Tetrathionate
1.8.5.2
Ttr
Trithionate
3.12.1.1
Thiosulfate
Phs
2.8.1.5
1.8.99.3
2.8.1.1
Taurine
Alkane-sulfonate
1.14.11.17
1.14.14.5
1.8.2.1
1.8.3.1
Sulfate
1.8.99.1
1.8.1.2
Asr
1.8.7.1
Sulfite
Sulfide
2.7.7.4
2.7.7.5
3.6.2.1
1.8.4.9
1.8.99.2
1.8.4.8
APS
2.7.1.25
3.1.3.7
PAPS
3.6.2.2
PAP
(Sulfide)n-1
(Sulfide)n
1.12.98.4
1.8.5.-
1.13.11.55
Sre
1.13.11.18
Sulfur
1.8.2.3
1.14.13.111
1.14.14.5
Methane-sulfonate
SfnG
Dimethylsulfone
3-Methylthio-propionate
2.1.1.269
DMSP
4.4.1.3
DMSO
1.8.1.17
1.8.5.3
1.8.2.4
Methane metabolism
Methyl-CoM
2.1.1.251
Methyl-mercaptan
1.8.3.4
1.14.13.131
Methyl-thioether
Glycine, serine and threonine metabolism
L-Serine
Methane metabolism
2.3.1.30
O-Acetyl-L-serine
Acetate
Carbon fixation pathways in prokaryotes
2.5.1.65
2.5.1.47
L-Cysteine
Cysteine and methionine metabolism
4.4.1.2
2.5.1.48
2.5.1.-
L-Homocysteine
O-Succinyl-L-homoserine
Succinate
2.3.1.46
L-Homoserine
Glycine, serine and threonine metabolism
Oxidation state +6 +4 +2 -2
Assimilatory sulfate reduction
Sulfate
PAPSS
Sat
CysND
APS
PAPSS
CysC
PAPS
CysH
Sulfite
CysJI
Sir
Sulfide
Dissimilatory sulfate reduction and oxidation
Sulfate
Sat
APS
AprAB
Sulfite
DsrAB
Sulfide
SOX system
Sulfate
SoxB
SoxY
SoxZ
SoxYZ-S-S-SO3
SoxA
SoxX
Thiosulfate
SoxYZ-S-SH
SoxC
SoxD
SoxYZ-S-SO3
SoxB
SoxYZ-SH
00920 6/3/14
(c) Kanehisa Laboratories

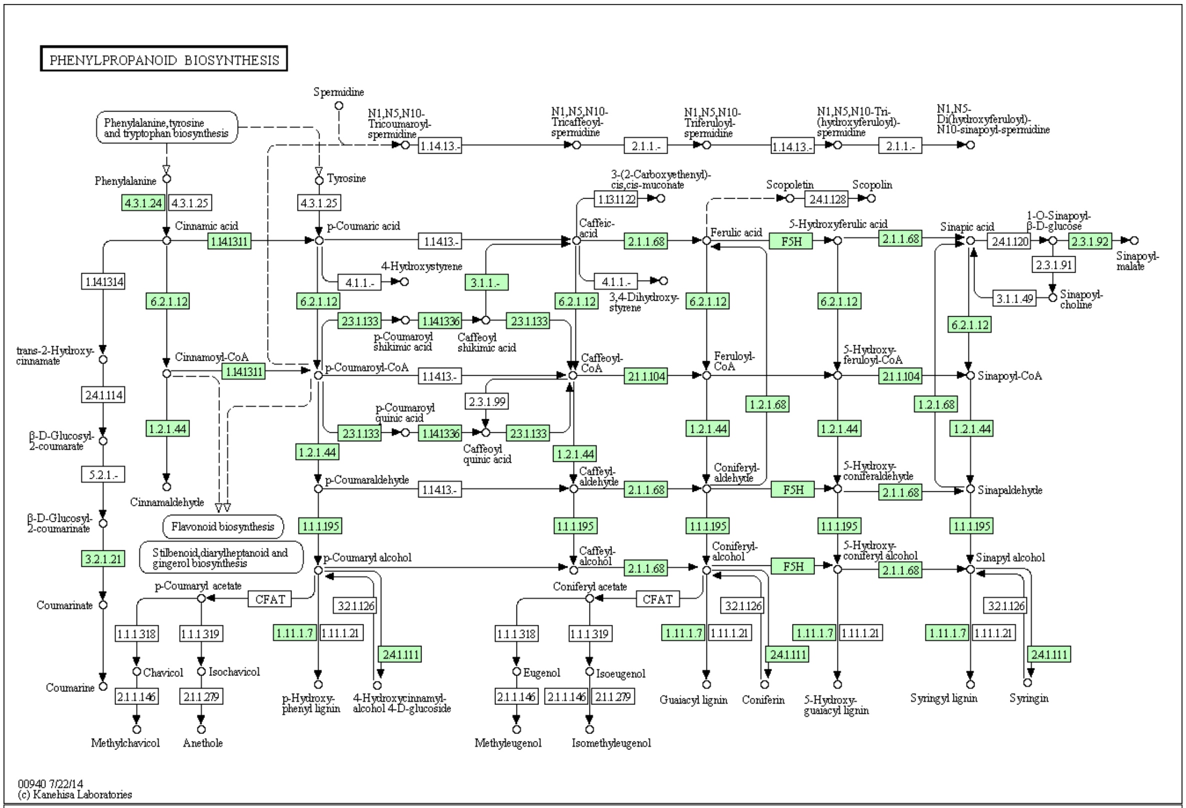
PHENYLPROPANOID BIOSYNTHESIS
Spermidine
Phenylalanine,tyrosine and tryptophan biosynthesis
N1,N5,N10-Tricoumaroyl-spermidine
1.14.13.-
N1,N5,N10-Tricaffeoyl-spermidine
2.1.1.-
N1,N5,N10-Triferuloyl-spermidine
1.14.13.-
N1,N5,N10-Tri-(hydroxyferuloyl)-spermidine
2.1.1.-
N1,N5-Di(hydroxyferuloyl)-N10-sinapoyl-spermidine
Phenylalanine
Tyrosine
4.3.1.24
4.3.1.25
4.3.1.25
3-(2-Carboxyethenyl)-cis,cis-muconate
1.13.11.22
Scopoletin
Scopolin
2.4.1.128
Cinnamic acid
1.14.13.11
p-Coumaric acid
1.14.13.-
Caffeic-acid
2.1.1.68
Ferulic acid
F5H
5-Hydroxyferulic acid
2.1.1.68
Sinapic acid
2.4.1.120
1-O-Sinapoyl-β-D-glucose
2.3.1.92
Sinapoyl-malate
2.3.1.91
3.1.1.49
Sinapoyl-choline
1.14.13.14
4-Hydroxystyrene
4.1.1.-
3.1.1.-
4.1.1.-
3,4-Dihydroxy-styrene
6.2.1.12
6.2.1.12
6.2.1.12
6.2.1.12
6.2.1.12
6.2.1.12
2.3.1.133
1.14.13.36
2.3.1.133
p-Coumaroyl shikimic acid
Caffeoyl shikimic acid
trans-2-Hydroxy-cinnamate
Cinnamoyl-CoA
1.14.13.11
p-Coumaroyl-CoA
1.14.13.-
Caffeoyl-CoA
2.1.1.104
Feruloyl-CoA
5-Hydroxy-feruloyl-CoA
2.1.1.104
Sinapoyl-CoA
2.4.1.114
2.3.1.99
1.2.1.68
1.2.1.68
p-Coumaroyl quinic acid
2.3.1.133
1.14.13.36
2.3.1.133
Caffeoyl quinic acid
β-D-Glucosyl-2-coumarate
1.2.1.44
1.2.1.44
1.2.1.44
1.2.1.44
1.2.1.44
1.2.1.44
5.2.1.-
Cinnamaldehyde
p-Coumaraldehyde
1.14.13.-
Caffeyl-aldehyde
2.1.1.68
Coniferyl-aldehyde
F5H
5-Hydroxy-coniferaldehyde
2.1.1.68
Sinapaldehyde
β-D-Glucosyl-2-coumarinate
Flavonoid biosynthesis
1.1.1.195
1.1.1.195
1.1.1.195
1.1.1.195
1.1.1.195
3.2.1.21
Stilbenoid,diarylheptanoid and gingerol biosynthesis
p-Coumaryl alcohol
Caffeyl-alcohol
2.1.1.68
Coniferyl-alcohol
F5H
5-Hydroxy-coniferyl alcohol
2.1.1.68
Sinapyl alcohol
p-Coumaryl acetate
CFAT
Coniferyl acetate
CFAT
3.2.1.126
3.2.1.126
3.2.1.126
Coumarinate
1.1.1.318
1.1.1.319
1.11.1.7
1.11.1.21
2.4.1.111
1.1.1.318
1.1.1.319
1.11.1.7
1.11.1.21
2.4.1.111
1.11.1.7
1.11.1.21
1.11.1.7
1.11.1.21
2.4.1.111
Chavicol
Isochavicol
Eugenol
Isoeugenol
Coumarine
2.1.1.146
2.1.1.279
2.1.1.146
2.1.1.146
2.1.1.279
p-Hydroxy-phenyl lignin
4-Hydroxycinnamyl-alcohol 4-D-glucoside
Guaiacyl lignin
Coniferin
5-Hydroxy-guaiacyl lignin
Syringyl lignin
Syringin
Methylchavicol
Anethole
Methyleugenol
Isomethyleugenol
00940 7/22/14
(c) Kanehisa Laboratories

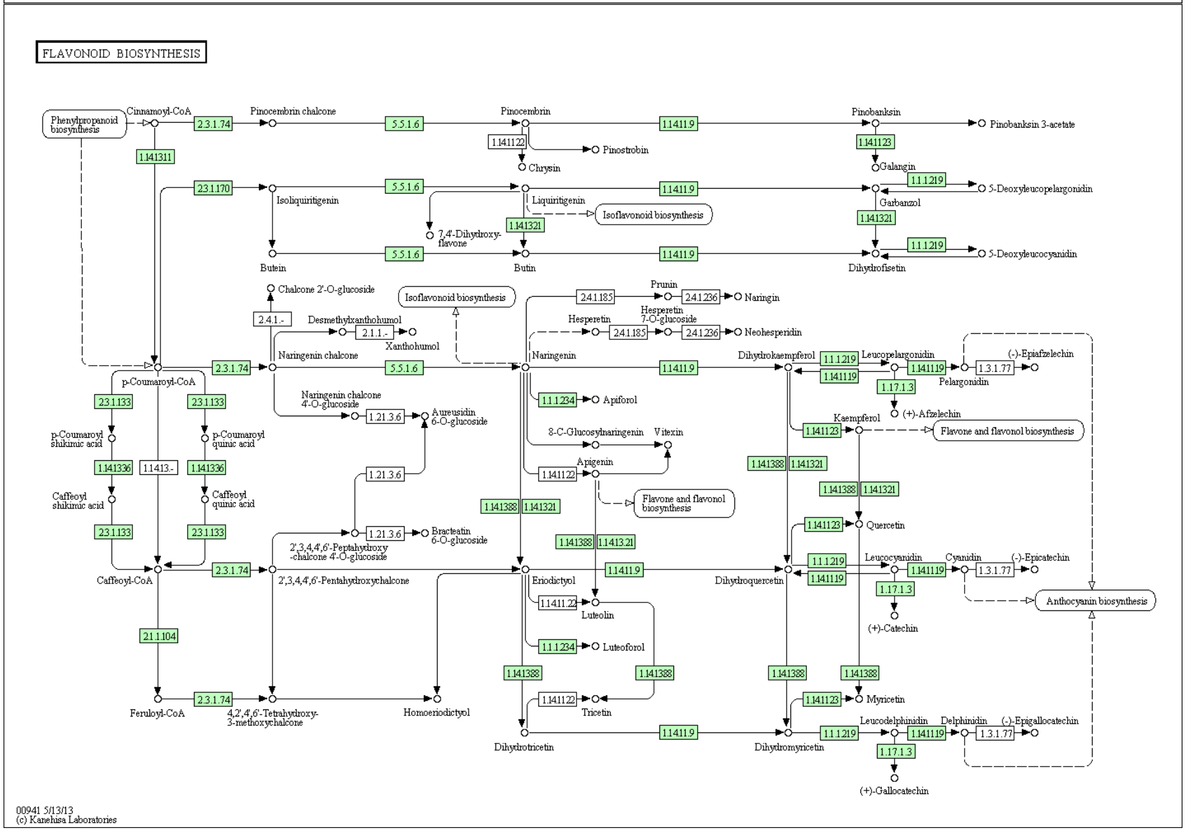
FLAVONOID BIOSYNTHESIS
Phenylpropanoid biosynthesis
Cinnamoyl-CoA
2.3.1.74
Pinocembrin chalcone
5.5.1.6
Pinocembrin
1.14.11.9
Pinobanksin
Pinobanksin 3-acetate
1.14.13.11
1.14.11.22
Pinostrobin
Chrysin
1.14.11.23
Galangin
2.3.1.170
Isoliquiritigenin
5.5.1.6
Liquiritigenin
1.14.11.9
1.1.1.219
5-Deoxyleucopelargonidin
Garbanzol
Isoflavonoid biosynthesis
7,4'-Dihydroxy-flavone
1.14.13.21
1.14.13.21
5.5.1.6
1.14.11.9
1.1.1.219
Butein
Butin
Dihydrofisetin
5-Deoxyleucocyanidin
Chalcone 2'-O-glucoside
Isoflavonoid biosynthesis
2.4.1.185
Prunin
2.4.1.236
Naringin
Hesperetin 7-O-glucoside
Hesperetin
2.4.1.185
2.4.1.236
Neohesperidin
2.4.1.-
Desmethylxanthohumol
2.1.1.-
Xanthohumol
p-Coumaroyl-CoA
2.3.1.74
Naringenin chalcone
5.5.1.6
Naringenin
1.14.11.9
Dihydrokaempferol
1.1.1.219
Leucopelargonidin
1.14.11.19
1.3.1.77
(-)-Epiafzelechin
Pelargonidin
1.14.11.19
1.17.1.3
(+)-Afzelechin
Naringenin chalcone 4'-O-glucoside
1.21.3.6
Aureusidin 6-O-glucoside
1.1.1.234
Apiforol
2.3.1.133
2.3.1.133
p-Coumaroyl shikimic acid
p-Coumaroyl quinic acid
8-C-Glucosylnaringenin
Vitexin
Kaempferol
1.14.11.23
Flavone and flavonol biosynthesis
1.14.13.36
1.14.13.-
1.14.13.36
1.21.3.6
1.14.11.22
Apigenin
1.14.13.88
1.14.13.21
Caffeoyl shikimic acid
Caffeoyl quinic acid
1.14.13.88
1.14.13.21
Flavone and flavonol biosynthesis
1.14.13.88
1.14.13.21
1.14.11.23
Quercetin
2.3.1.133
2.3.1.133
1.21.3.6
Bracteatin 6-O-glucoside
2',3,4,4',6'-Peptahydroxy-chalcone 4'-O-glucoside
1.14.13.88
1.14.13.21
1.1.1.219
Leucocyanidin
Cyanidin
(-)-Epicatechin
Caffeoyl-CoA
2.3.1.74
2',3,4,4',6'-Pentahydroxychalcone
Eriodictyol
1.14.11.9
Dihydroquercetin
1.14.11.19
1.3.1.77
1.14.11.19
1.17.1.3
Anthocyanin biosynthesis
1.14.11.22
Luteolin
(+)-Catechin
2.1.1.104
1.1.1.234
Luteoforol
1.14.13.88
1.14.13.88
1.14.13.88
1.14.13.88
Feruloyl-CoA
2.3.1.74
4,2',4',6'-Tetrahydroxy-3-methoxychalcone
Homoeriodictyol
1.14.11.22
Tricetin
1.14.11.23
Myricetin
1.14.11.9
Dihydrotricetin
Dihydromyricetin
1.1.1.219
Leucodelphinidin
1.14.11.19
Delphinidin
1.3.1.77
(-)-Epigallocatechin
1.17.1.3
(+)-Gallocatechin
00941 5/13/13
(c) Kanehisa Laboratories

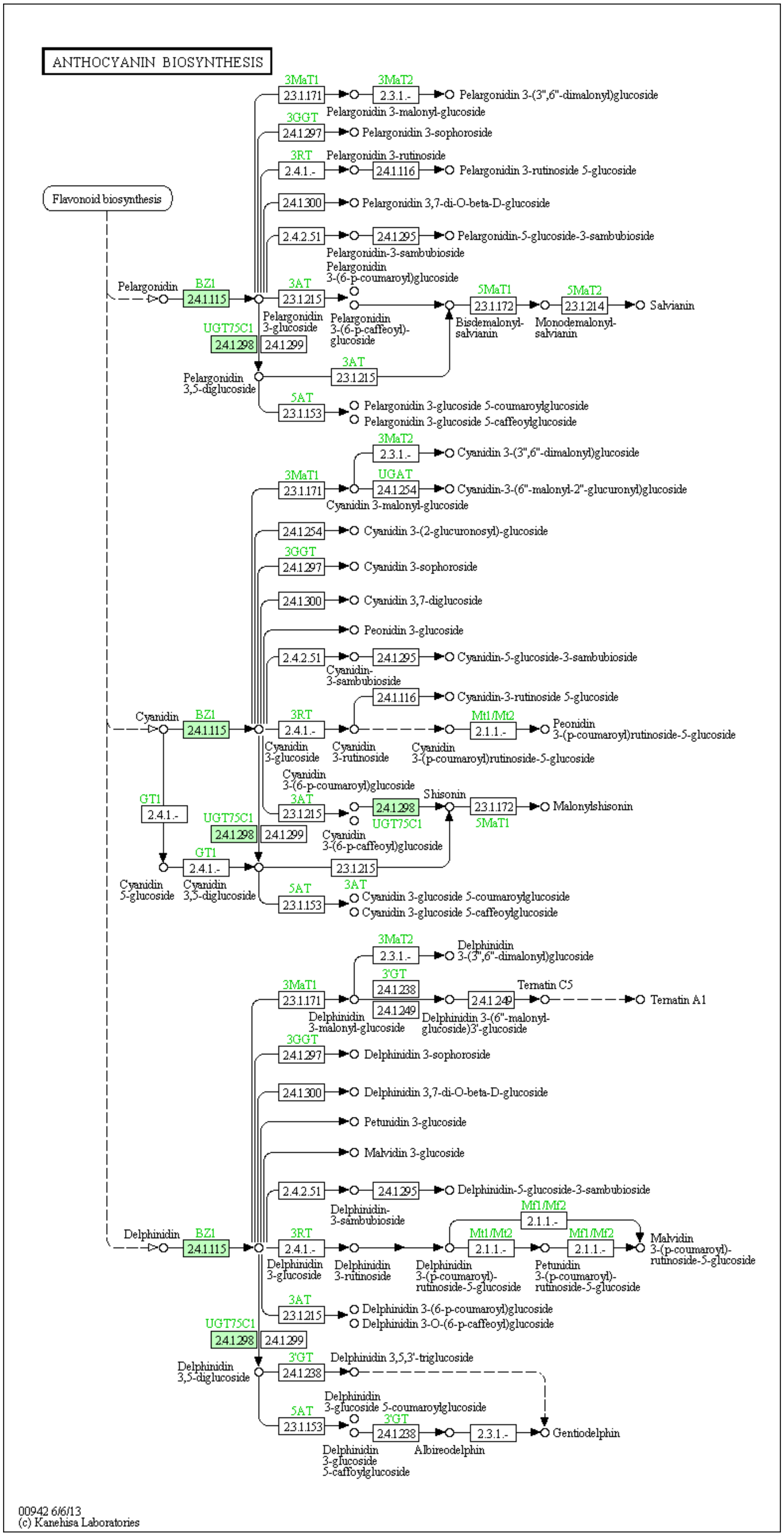
ANTHOCYANIN BIOSYNTHESIS
Flavonoid biosynthesis
3MaT1
2.3.1.171
3MaT2
2.3.1.-
Pelargonidin 3-(3",6"-dimalonyl)glucoside
Pelargonidin 3-malonyl-glucoside
3GGT
2.4.1.297
Pelargonidin 3-sophoroside
3RT
2.4.1.-
Pelargonidin 3-rutinoside
2.4.1.116
Pelargonidin 3-rutinoside 5-glucoside
2.4.1.300
Pelargonidin 3,7-di-O-beta-D-glucoside
2.4.2.51
2.4.1.295
Pelargonidin-5-glucoside-3-sambubioside
Pelargonidin-3-sambubioside
Pelargonidin 3-(6-p-coumaroyl)glucoside
Pelargonidin
BZ1
2.4.1.115
3AT
2.3.1.215
5MaT1
2.3.1.172
5MaT2
2.3.1.214
Salvianin
Pelargonidin 3-glucoside
Pelargonidin 3-(6-p-caffeoyl)-glucoside
Bisdemalonyl-salvianin
Monodemalonyl-salvianin
UGT75C1
2.4.1.298
2.4.1.299
Pelargonidin 3,5-diglucoside
5AT
2.3.1.153
Pelargonidin 3-glucoside 5-coumaroylglucoside
Pelargonidin 3-glucoside 5-caffeoylglucoside
Cyanidin 3-(3",6"-dimalonyl)glucoside
UGAT
2.4.1.254
Cyanidin-3-(6"-malonyl-2"-glucuronyl)glucoside
Cyanidin 3-malonyl-glucoside
Cyanidin 3-(2-glucuronosyl)-glucoside
Cyanidin 3-sophoroside
Cyanidin 3,7-diglucoside
Peonidin 3-glucoside
Cyanidin-5-glucoside-3-sambubioside
Cyanidin-3-sambubioside
Cyanidin-3-rutinoside 5-glucoside
Cyanidin
Mt1/Mt2
2.1.1.-
Peonidin 3-(p-coumaroyl)rutinoside-5-glucoside
Cyanidin 3-glucoside
Cyanidin 3-rutinoside
Cyanidin 3-(p-coumaroyl)rutinoside-5-glucoside
Cyanidin 3-(6-p-coumaroyl)glucoside
Shisonin
Malonylshisonin
GT1
Cyanidin 3-(6-p-caffeoyl)glucoside
Cyanidin 5-glucoside
Cyanidin 3,5-diglucoside
Cyanidin 3-glucoside 5-coumaroylglucoside
Cyanidin 3-glucoside 5-caffeoylglucoside
Delphinidin 3-(3",6"-dimalonyl)glucoside
3'GT
2.4.1.238
2.4.1.249
Ternatin C5
Ternatin A1
Delphinidin 3-malonyl-glucoside
Delphinidin 3-(6"-malonyl-glucoside)3'-glucoside
Delphinidin 3-sophoroside
Delphinidin 3,7-di-O-beta-D-glucoside
Petunidin 3-glucoside
Malvidin 3-glucoside
Delphinidin-5-glucoside-3-sambubioside
Delphinidin-3-sambubioside
Mf1/Mf2
Delphinidin
Malvidin 3-(p-coumaroyl)-rutinoside-5-glucoside
Delphinidin 3-glucoside
Delphinidin 3-rutinoside
Delphinidin 3-(p-coumaroyl)-rutinoside-5-glucoside
Petunidin 3-(p-coumaroyl)-rutinoside-5-glucoside
Delphinidin 3-(6-p-coumaroyl)glucoside
Delphinidin 3-O-(6-p-caffeoyl)glucoside
Delphinidin 3,5,3'-triglucoside
Delphinidin 3,5-diglucoside
Delphinidin 3-glucoside 5-coumaroylglucoside
Gentiodelphin
Delphinidin 3-glucoside 5-caffoylglucoside
Albireodelphin
00942 6/6/13
(c) Kanehisa Laboratories

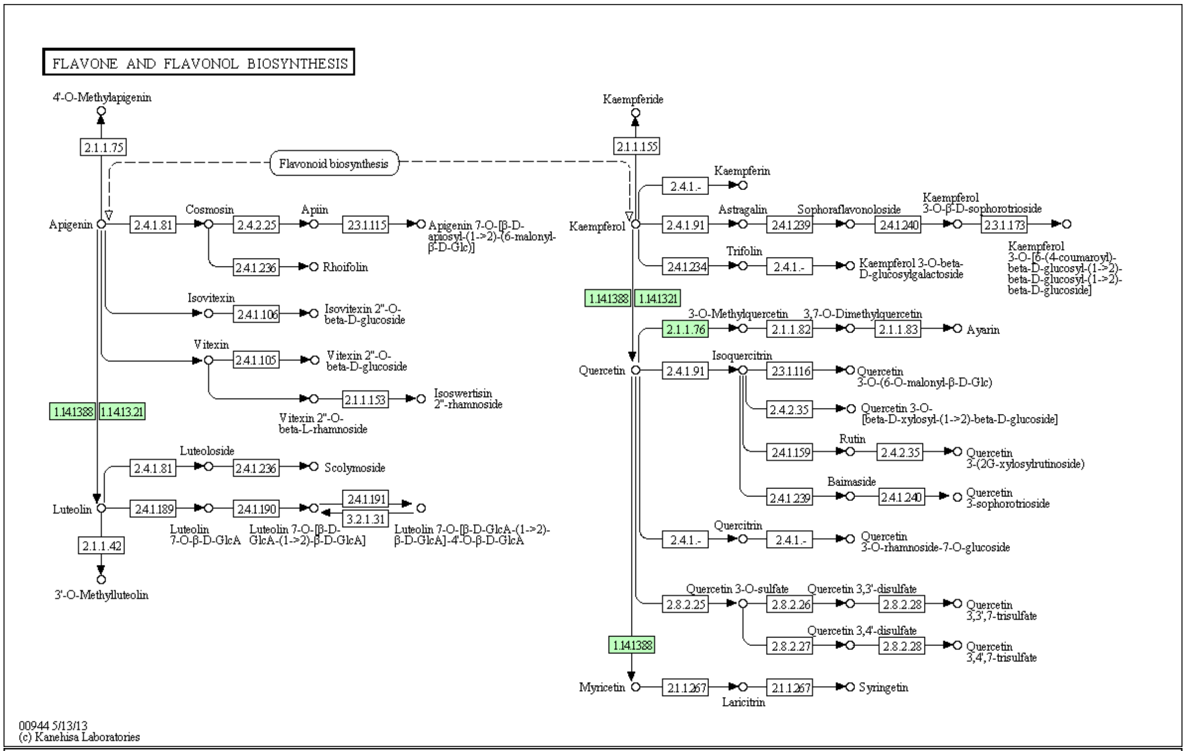
FLAVONE AND FLAVONOL BIOSYNTHESIS
4'-O-Methylapigenin
2.1.1.75
Flavonoid biosynthesis
Apigenin
2.4.1.81
Cosmosin
2.4.2.25
Apiin
2.3.1.115
Apigenin 7-O-[β-D-apiosyl-(1->2)-(6-malonyl-β-D-Glc)]
2.4.1.236
Rhoifolin
Isovitexin
2.4.1.106
Isovitexin 2"-O-beta-D-glucoside
Vitexin
2.4.1.105
Vitexin 2"-O-beta-D-glucoside
2.1.1.153
Isoswertisin 2"-rhamnoside
Vitexin 2"-O-beta-L-rhamnoside
1.14.13.88
1.14.13.21
Luteoloside
2.4.1.81
2.4.1.236
Scolymoside
Luteolin
2.4.1.189
2.4.1.190
2.4.1.191
3.2.1.31
Luteolin 7-O-β-D-GlcA
Luteolin 7-O-[β-D-GlcA-(1->2)-β-D-GlcA]
Luteolin 7-O-[β-D-GlcA-(1->2)-β-D-GlcA]-4'-O-β-D-GlcA
2.1.1.42
3'-O-Methylluteolin
Kaempferide
2.1.1.155
Kaempferin
2.4.1.-
Kaempferol
2.4.1.91
Astragalin
2.4.1.239
Sophoraflavonoloside
2.4.1.240
Kaempferol 3-O-β-D-sophorotrioside
2.3.1.173
Kaempferol 3-O-[6-(4-coumaroyl)-beta-D-glucosyl-(1->2)-beta-D-glucosyl-(1->2)-beta-D-glucoside]
2.4.1.234
Trifolin
2.4.1.-
Kaempferol 3-O-beta-D-glucosylgalactoside
1.14.13.88
1.14.13.21
3-O-Methylquercetin
3,7-O-Dimethylquercetin
2.1.1.76
2.1.1.82
2.1.1.83
Ayarin
Quercetin
2.4.1.91
Isoquercitrin
2.3.1.116
Quercetin 3-O-(6-O-malonyl-β-D-Glc)
2.4.2.35
Quercetin 3-O-[beta-D-xylosyl-(1->2)-beta-D-glucoside]
Rutin
2.4.1.159
2.4.2.35
Quercetin 3-(2G-xylosylrutinoside)
Baimaside
2.4.1.239
2.4.1.240
Quercetin 3-sophorotrioside
Quercitrin
2.4.1.-
2.4.1.-
Quercetin 3-O-rhamnoside-7-O-glucoside
Quercetin 3-O-sulfate
Quercetin 3,3'-disulfate
2.8.2.25
2.8.2.26
2.8.2.28
Quercetin 3,3',7-trisulfate
Quercetin 3,4'-disulfate
2.8.2.27
2.8.2.28
Quercetin 3,4',7-trisulfate
1.14.13.88
Myricetin
2.1.1.267
Laricitrin
2.1.1.267
Syringetin
00944 5/13/13
(c) Kanehisa Laboratories

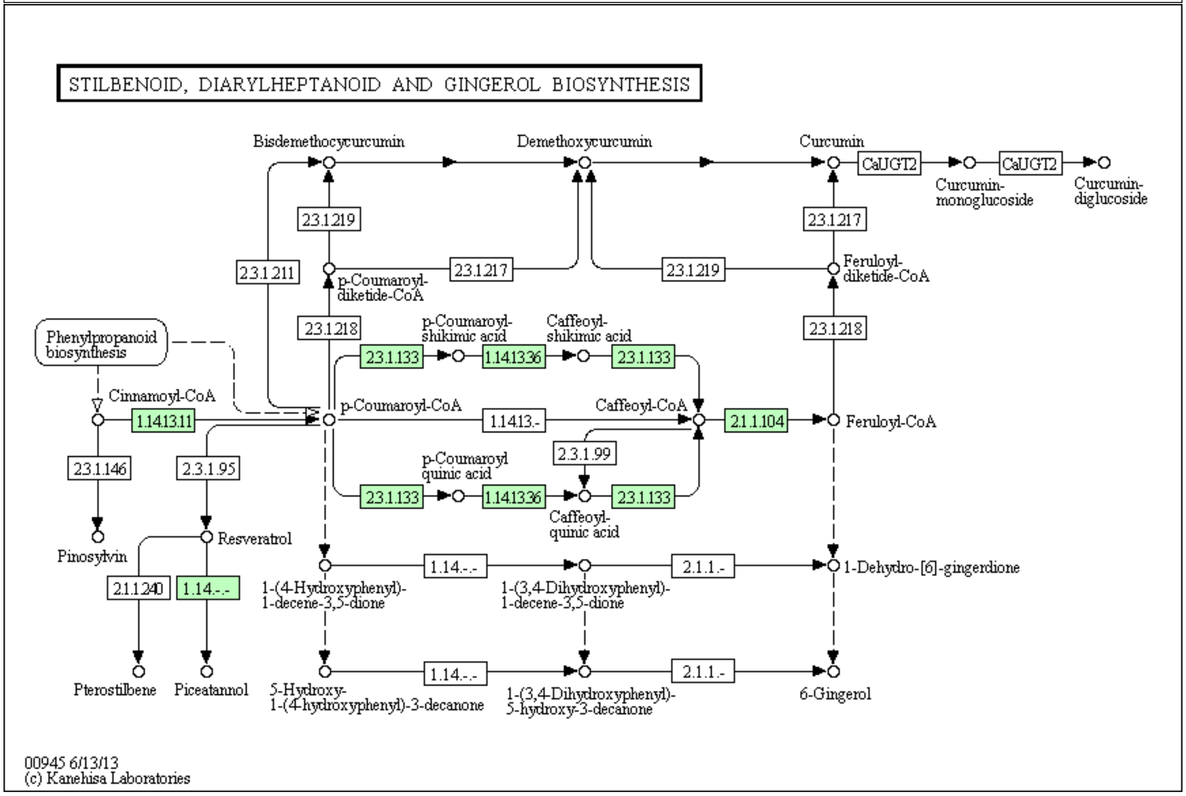
STILBENOID, DIARYLHEPTANOID AND GINGEROL BIOSYNTHESIS
Bisdemethoxycurcumin
Demethoxycurcumin
Curcumin
CaUGT2
CaUGT2
Curcumin-monoglucoside
Curcumin-diglucoside
2.3.1.219
2.3.1.211
2.3.1.217
2.3.1.219
2.3.1.217
p-Coumaroyl-diketide-CoA
Feruloyl-diketide-CoA
2.3.1.218
2.3.1.218
Phenylpropanoid biosynthesis
p-Coumaroyl-shikimic acid
Caffeoyl-shikimic acid
2.3.1.133
1.14.13.36
2.3.1.133
Cinnamoyl-CoA
1.14.13.11
p-Coumaroyl-CoA
1.14.13.-
Caffeoyl-CoA
2.1.1.104
Feruloyl-CoA
2.3.1.99
p-Coumaroyl quinic acid
2.3.1.133
1.14.13.36
2.3.1.133
Caffeoyl-quinic acid
2.3.1.146
2.3.1.95
Pinosylvin
Resveratrol
2.1.1.240
1.14.-.-
Pterostilbene
Piceatannol
1.14.-.-
2.1.1.-
1-(4-Hydroxyphenyl)-1-decene-3,5-dione
1-(3,4-Dihydroxyphenyl)-1-decene-3,5-dione
1-Dehydro-[6]-gingerdione
1.14.-.-
2.1.1.-
5-Hydroxy-1-(4-hydroxyphenyl)-3-decanone
1-(3,4-Dihydroxyphenyl)-5-hydroxy-3-decanone
6-Gingerol
00945 6/13/13
(c) Kanehisa Laboratories

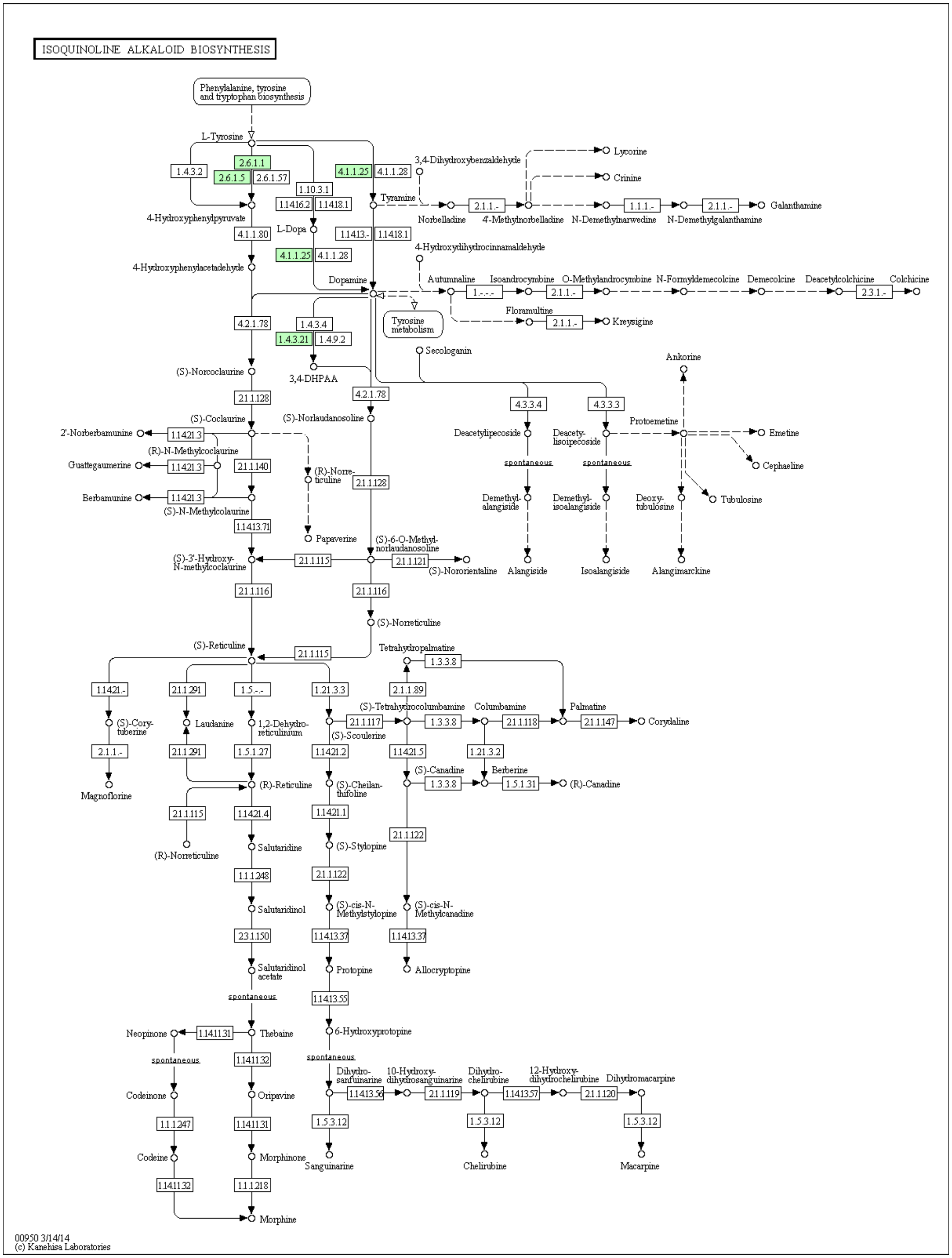
ISOQUINOLINE ALKALOID BIOSYNTHESIS
Phenylalanine, tyrosine and tryptophan biosynthesis
L-Tyrosine
4-Hydroxyphenylpyruvate
4-Hydroxyphenylacetadehyde
L-Dopa
Tyramine
Dopamine
Tyrosine metabolism
3,4-DHPAA
3,4-Dihydroxybenzaldehyde
Norbelladine
4'-Methylnorbelladine
N-Demethylnarwedine
N-Demethylgalanthamine
Galanthamine
Lycorine
Crinine
4-Hydroxydihydrocinnamaldehyde
Autumnaline
Isoandrocymbine
O-Methylandrocymbine
N-Formyldemecolcine
Demecolcine
Deacetylcolchicine
Colchicine
Floramultine
Kreysigine
Secologanin
Ankorine
Deacetylipecoside
Deacetyl-isoipecoside
Protoemetine
Emetine
Cephaeline
Tubulosine
Demethyl-alangiside
Demethyl-isoalangiside
Deoxy-tubulosine
Alangiside
Isoalangiside
Alangimarckine
(S)-Norcoclaurine
(S)-Coclaurine
(S)-Norlaudanosoline
2'-Norberbamunine
(R)-N-Methylcoclaurine
Guattegaumerine
Berbamunine
(S)-N-Methylcolaurine
(R)-Norreticuline
Papaverine
(S)-3'-Hydroxy-N-methylcoclaurine
(S)-6-O-Methyl-norlaudanosoline
(S)-Nororientaline
(S)-Norreticuline
(S)-Reticuline
Tetrahydropalmatine
(S)-Corytuberine
Magnoflorine
Laudanine
1,2-Dehydroreticulinium
(R)-Reticuline
(R)-Norreticuline
(S)-Scoulerine
(S)-Tetrahydrocolumbamine
Columbamine
Palmatine
Corydaline
(S)-Canadine
Berberine
(R)-Canadine
(S)-Cheilanthifoline
(S)-Stylopine
(S)-cis-N-Methylstylopine
(S)-cis-N-Methylcanadine
Protopine
Allocryptopine
6-Hydroxyprotopine
Salutaridine
Salutaridinol
Salutaridinol acetate
Thebaine
Neopinone
Codeinone
Codeine
Oripavine
Morphinone
Morphine
Dihydrosanguinarine
10-Hydroxy-dihydrosanguinarine
Dihydrochelirubine
12-Hydroxy-dihydrochelirubine
Dihydromacarpine
Sanguinarine
Chelirubine
Macarpine
spontaneous
2.6.1.1
2.6.1.5
2.6.1.57
1.4.3.2
4.1.1.25
4.1.1.28
1.10.3.1
1.14.16.2
1.14.18.1
4.1.1.80
1.14.13.-
4.2.1.78
1.4.3.4
1.4.3.21
1.4.9.2
2.1.1.-
1.1.1.-
1.-.-.-
2.3.1.-
4.3.3.4
4.3.3.3
2.1.1.128
1.14.21.3
2.1.1.140
1.14.13.71
2.1.1.115
2.1.1.121
2.1.1.116
1.14.21.-
2.1.1.291
1.5.-.-
1.21.3.3
2.1.1.89
1.3.3.8
2.1.1.117
2.1.1.118
2.1.1.147
1.5.1.27
1.14.21.2
1.14.21.5
1.21.3.2
1.5.1.31
1.14.21.4
1.14.21.1
2.1.1.122
1.1.1.248
1.14.13.37
2.3.1.150
1.14.13.55
1.14.11.31
1.14.11.32
1.1.1.247
1.1.1.218
1.14.13.56
2.1.1.119
1.14.13.57
2.1.1.120
1.5.3.12
00950 3/14/14
(c) Kanehisa Laboratories

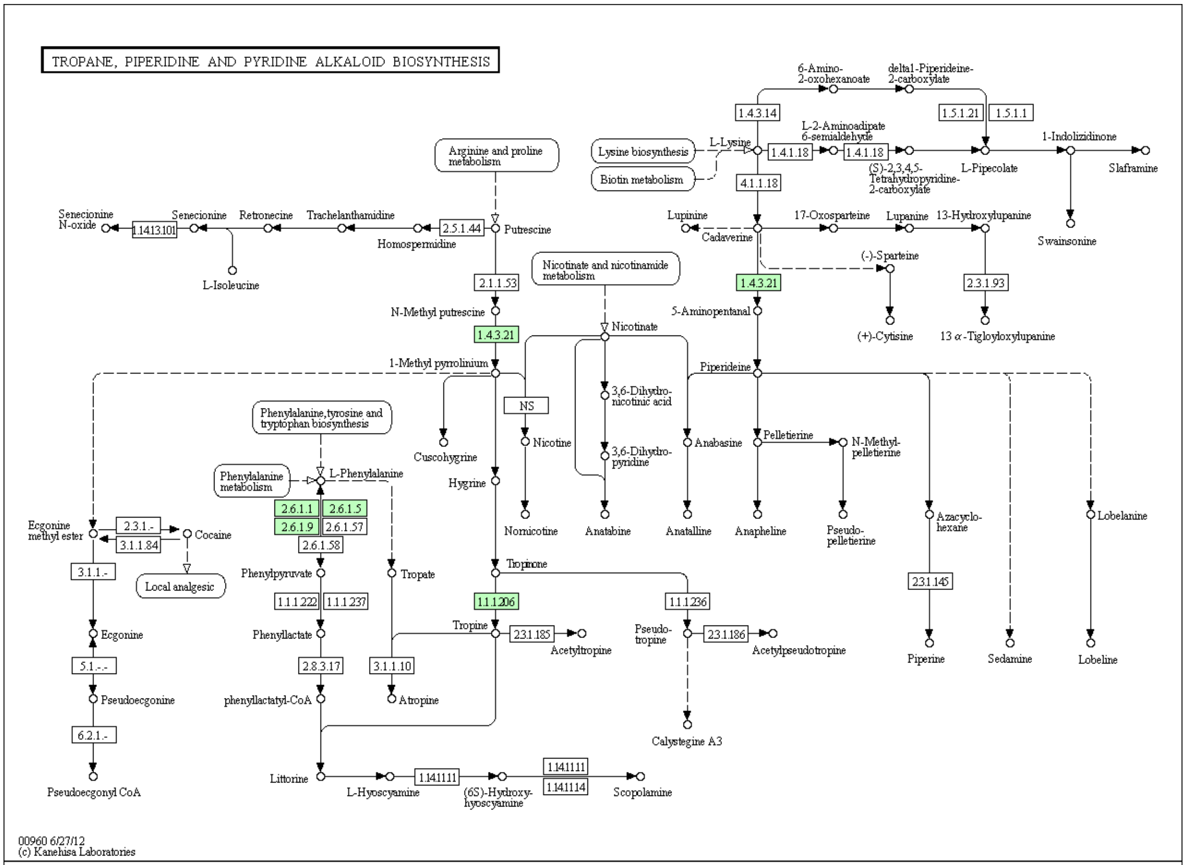
TROPANE, PIPERIDINE AND PYRIDINE ALKALOID BIOSYNTHESIS
Arginine and proline metabolism
Putrescine
2.5.1.44
Homospermidine
Trachelanthamidine
Retronecine
Senecionine
1.14.13.101
Senecionine N-oxide
L-Isoleucine
2.1.1.53
N-Methyl putrescine
1.4.3.21
1-Methyl pyrrolinium
Nicotinate and nicotinamide metabolism
Nicotinate
NS
Nicotine
Nornicotine
3,6-Dihydro-nicotinic acid
3,6-Dihydro-pyridine
Anatabine
Anabasine
Anatalline
Cuscohygrine
Hygrine
Tropinone
1.1.1.206
Tropine
2.3.1.185
Acetyltropine
1.1.1.236
Pseudo-tropine
2.3.1.186
Acetylpseudotropine
Calystegine A3
Phenylalanine, tyrosine and tryptophan biosynthesis
Phenylalanine metabolism
L-Phenylalanine
2.6.1.1
2.6.1.5
2.6.1.9
2.6.1.57
2.6.1.58
Phenylpyruvate
1.1.1.222
1.1.1.237
Phenyllactate
2.8.3.17
phenyllactatyl-CoA
Tropate
3.1.1.10
Atropine
Littorine
L-Hyoscyamine
1.14.11.11
(6S)-Hydroxy-hyoscyamine
1.14.11.14
Scopolamine
Ecgonine methyl ester
2.3.1.-
3.1.1.84
Cocaine
Local analgesic
3.1.1.-
Ecgonine
5.1.-.-
Pseudoecgonine
6.2.1.-
Pseudoecgonyl CoA
6-Amino-2-oxohexanoate
delta1-Piperideine-2-carboxylate
1.4.3.14
1.5.1.21
1.5.1.1
Lysine biosynthesis
Biotin metabolism
L-Lysine
1.4.1.18
L-2-Aminoadipate 6-semialdehyde
(S)-2,3,4,5-Tetrahydropyridine-2-carboxylate
L-Pipecolate
1-Indolizidinone
Slaframine
Swainsonine
4.1.1.18
Lupinine
Cadaverine
17-Oxosparteine
Lupanine
13-Hydroxylupanine
(-)-Sparteine
2.3.1.93
(+)-Cytisine
13 α-Tigloyloxylupanine
5-Aminopentanal
Piperideine
Pelletierine
N-Methyl-pelletierine
Anapheline
Pseudo-pelletierine
Azacyclo-hexane
2.3.1.145
Piperine
Sedamine
Lobelanine
Lobeline
00960 6/27/12
(c) Kanehisa Laboratories

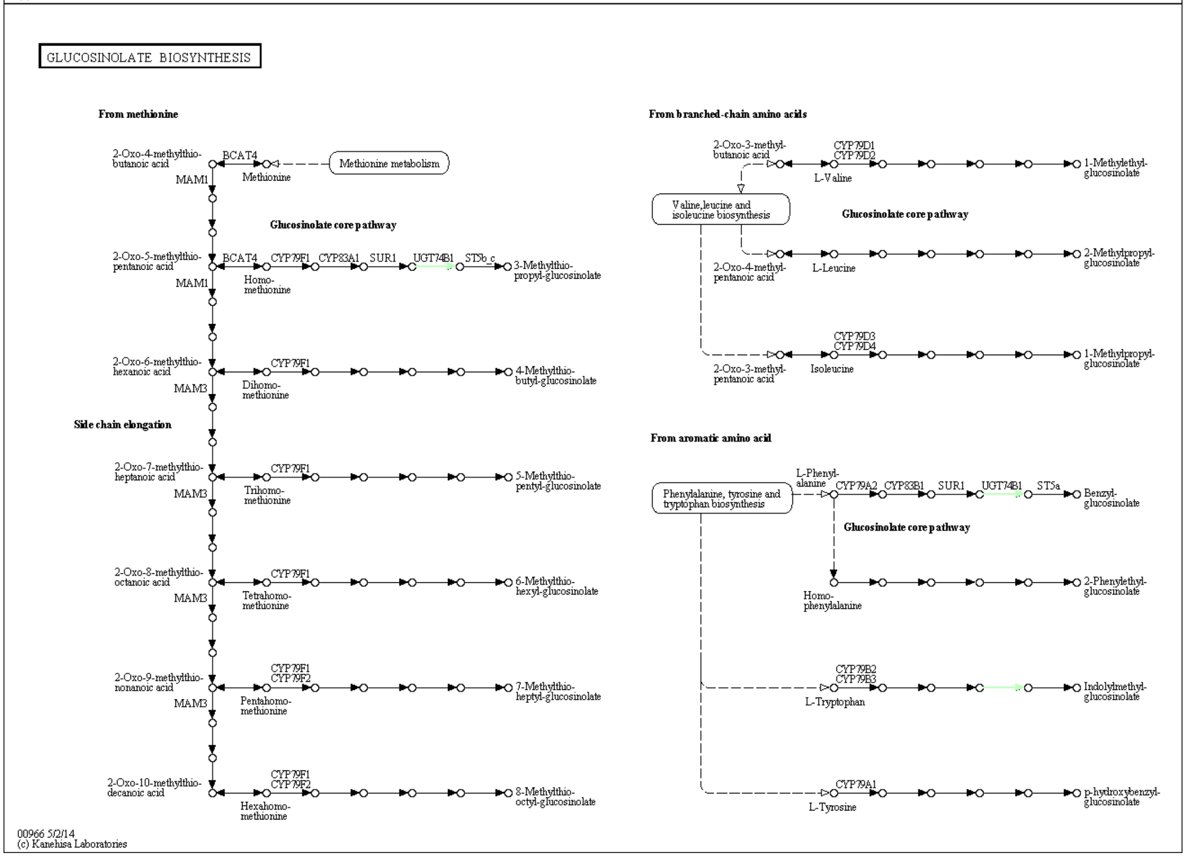
GLUCOSINOLATE BIOSYNTHESIS
From methionine
2-Oxo-4-methylthio-butanoic acid
BCAT4
Methionine
Methionine metabolism
MAM1
Glucosinolate core pathway
2-Oxo-5-methylthio-pentanoic acid
CYP79F1
CYP83A1
SUR1
UGT74B1
ST5b_c
Homo-methionine
3-Methylthio-propyl-glucosinolate
2-Oxo-6-methylthio-hexanoic acid
MAM3
Dihomo-methionine
4-Methylthio-butyl-glucosinolate
Side chain elongation
2-Oxo-7-methylthio-heptanoic acid
Trihomo-methionine
5-Methylthio-pentyl-glucosinolate
2-Oxo-8-methylthio-octanoic acid
Tetrahomo-methionine
6-Methylthio-hexyl-glucosinolate
2-Oxo-9-methylthio-nonanoic acid
CYP79F2
Pentahomo-methionine
7-Methylthio-heptyl-glucosinolate
2-Oxo-10-methylthio-decanoic acid
Hexahomo-methionine
8-Methylthio-octyl-glucosinolate
From branched-chain amino acids
2-Oxo-3-methyl-butanoic acid
CYP79D1
CYP79D2
L-Valine
1-Methylethyl-glucosinolate
Valine, leucine and isoleucine biosynthesis
2-Oxo-4-methyl-pentanoic acid
L-Leucine
2-Methylpropyl-glucosinolate
CYP79D3
CYP79D4
2-Oxo-3-methyl-pentanoic acid
Isoleucine
1-Methylpropyl-glucosinolate
From aromatic amino acid
Phenylalanine, tyrosine and tryptophan biosynthesis
L-Phenyl-alanine
CYP79A2
CYP83B1
ST5a
Benzyl-glucosinolate
Homo-phenylalanine
2-Phenylethyl-glucosinolate
CYP79B2
CYP79B3
L-Tryptophan
Indolylmethyl-glucosinolate
CYP79A1
L-Tyrosine
p-hydroxybenzyl-glucosinolate
00966 5/2/14
(c) Kanehisa Laboratories

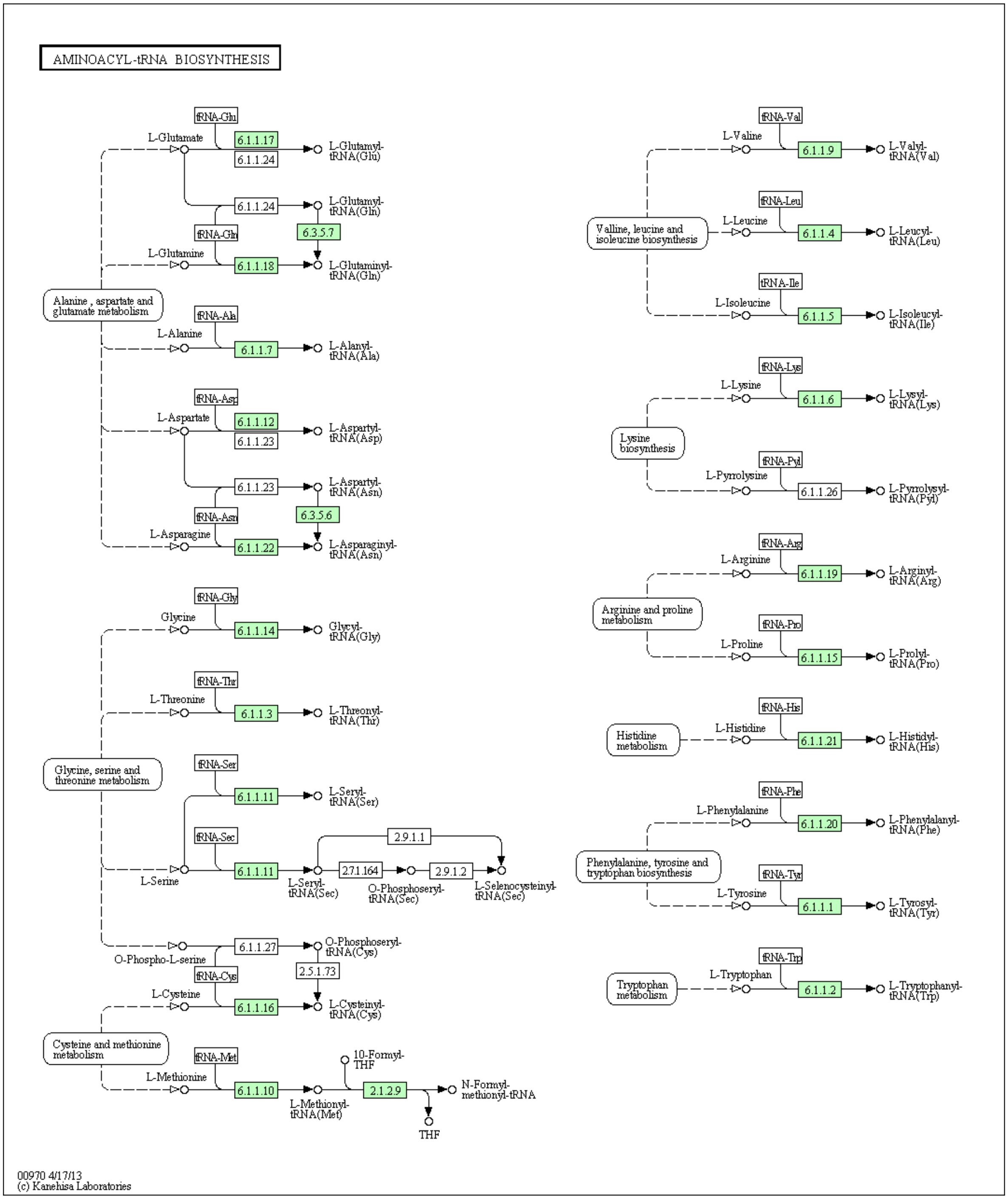
AMINOACYL-tRNA BIOSYNTHESIS
tRNA-Glu
L-Glutamate
6.1.1.17
6.1.1.24
L-Glutamyl-tRNA(Glu)
6.1.1.24
L-Glutamyl-tRNA(Gln)
6.3.5.7
tRNA-Gln
L-Glutamine
6.1.1.18
L-Glutaminyl-tRNA(Gln)
Alanine , aspartate and glutamate metabolism
tRNA-Ala
L-Alanine
6.1.1.7
L-Alanyl-tRNA(Ala)
tRNA-Asp
L-Aspartate
6.1.1.12
6.1.1.23
L-Aspartyl-tRNA(Asp)
6.1.1.23
L-Aspartyl-tRNA(Asn)
6.3.5.6
tRNA-Asn
L-Asparagine
6.1.1.22
L-Asparaginyl-tRNA(Asn)
tRNA-Gly
Glycine
6.1.1.14
Glycyl-tRNA(Gly)
tRNA-Thr
L-Threonine
6.1.1.3
L-Threonyl-tRNA(Thr)
Glycine, serine and threonine metabolism
tRNA-Ser
6.1.1.11
L-Seryl-tRNA(Ser)
tRNA-Sec
2.9.1.1
6.1.1.11
L-Serine
L-Seryl-tRNA(Sec)
2.7.1.164
O-Phosphoseryl-tRNA(Sec)
2.9.1.2
L-Selenocysteinyl-tRNA(Sec)
6.1.1.27
O-Phospho-L-serine
O-Phosphoseryl-tRNA(Cys)
2.5.1.73
tRNA-Cys
L-Cysteine
6.1.1.16
L-Cysteinyl-tRNA(Cys)
Cysteine and methionine metabolism
tRNA-Met
L-Methionine
6.1.1.10
L-Methionyl-tRNA(Met)
10-Formyl-THF
2.1.2.9
N-Formyl-methionyl-tRNA
THF
tRNA-Val
L-Valine
6.1.1.9
L-Valyl-tRNA(Val)
Valline, leucine and isoleucine biosynthesis
tRNA-Leu
L-Leucine
6.1.1.4
L-Leucyl-tRNA(Leu)
tRNA-Ile
L-Isoleucine
6.1.1.5
L-Isoleucyl-tRNA(Ile)
tRNA-Lys
L-Lysine
6.1.1.6
L-Lysyl-tRNA(Lys)
Lysine biosynthesis
tRNA-Pyl
L-Pyrrolysine
6.1.1.26
L-Pyrrolysyl-tRNA(Pyl)
tRNA-Arg
L-Arginine
6.1.1.19
L-Arginyl-tRNA(Arg)
Arginine and proline metabolism
tRNA-Pro
L-Proline
6.1.1.15
L-Prolyl-tRNA(Pro)
Histidine metabolism
tRNA-His
L-Histidine
6.1.1.21
L-Histidyl-tRNA(His)
tRNA-Phe
L-Phenylalanine
6.1.1.20
L-Phenylalanyl-tRNA(Phe)
Phenylalanine, tyrosine and tryptophan biosynthesis
tRNA-Tyr
L-Tyrosine
6.1.1.1
L-Tyrosyl-tRNA(Tyr)
Tryptophan metabolism
tRNA-Trp
L-Tryptophan
6.1.1.2
L-Tryptophanyl-tRNA(Trp)
00970 4/17/13
(c) Kanehisa Laboratories

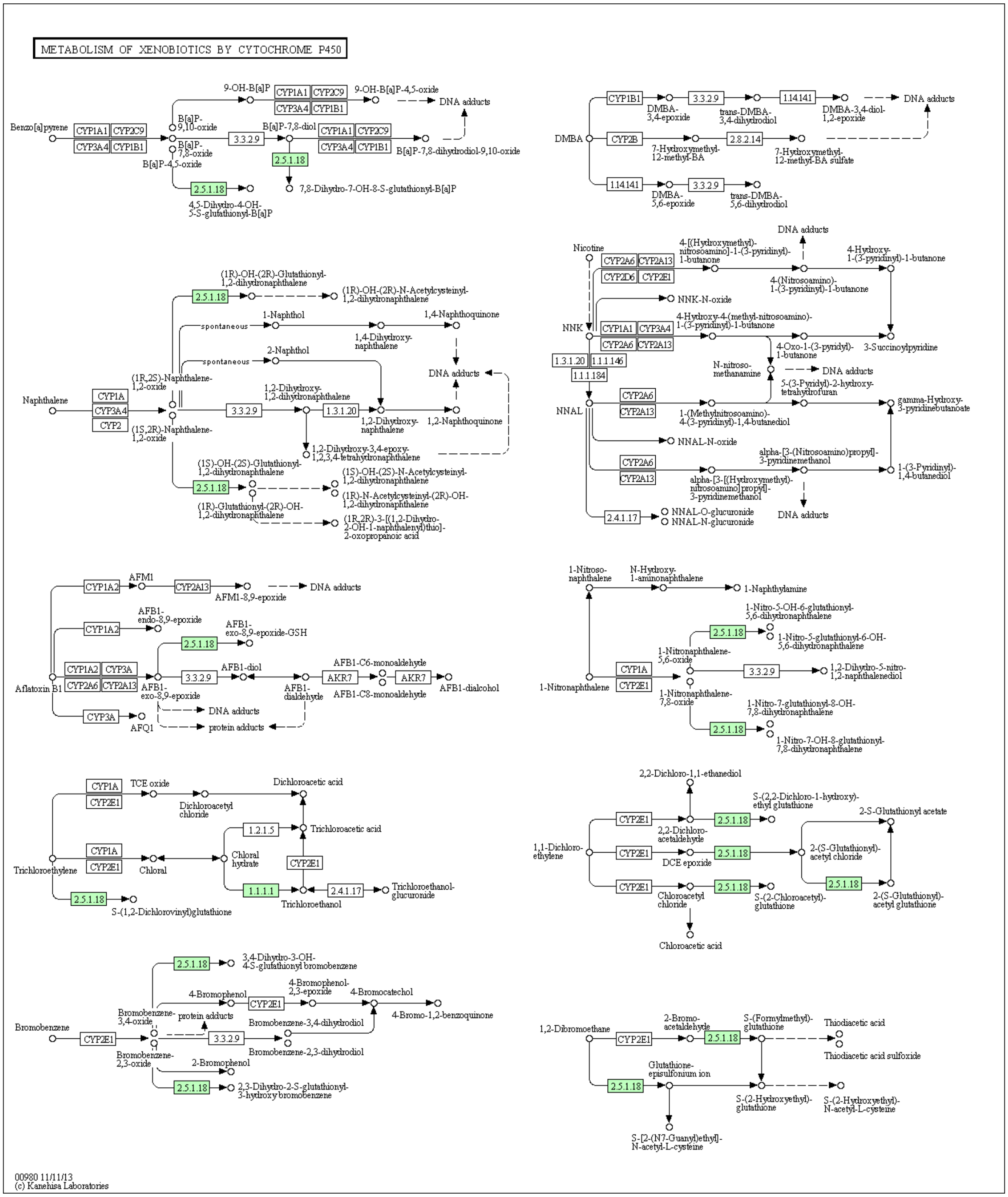
METABOLISM OF XENOBIOTICS BY CYTOCHROME P450
Benzo[a]pyrene
CYP1A1 CYP2C9 CYP3A4 CYP1B1
9-OH-B[a]P
9-OH-B[a]P-4,5-oxide
DNA adducts
B[a]P-9,10-oxide
B[a]P-7,8-oxide
B[a]P-4,5-oxide
3.3.2.9
B[a]P-7,8-diol
2.5.1.18
B[a]P-7,8-dihydrodiol-9,10-oxide
7,8-Dihydro-7-OH-8-S-glutathionyl-B[a]P
4,5-Dihydro-4-OH-5-S-glutathionyl-B[a]P
DMBA
CYP1B1 CYP2B 1.14.14.1 2.8.2.14
DMBA-3,4-epoxide
trans-DMBA-3,4-dihydrodiol
DMBA-3,4-diol-1,2-epoxide
7-Hydroxymethyl-12-methyl-BA
7-Hydroxymethyl-12-methyl-BA sulfate
DMBA-5,6-epoxide
trans-DMBA-5,6-dihydrodiol
(1R)-OH-(2R)-Glutathionyl-1,2-dihydronaphthalene
(1R)-OH-(2R)-N-Acetylcysteinyl-1,2-dihydronaphthalene
spontaneous
1-Naphthol
1,4-Naphthoquinone
1,4-Dihydroxy-naphthalene
2-Naphthol
Naphthalene
CYP1A CYP3A4 CYP2
(1R,2S)-Naphthalene-1,2-oxide
(1S,2R)-Naphthalene-1,2-oxide
1,2-Dihydroxy-1,2-dihydronaphthalene
1.3.1.20
1,2-Dihydroxy-naphthalene
1,2-Naphthoquinone
1,2-Dihydroxy-3,4-epoxy-1,2,3,4-tetrahydronaphthalene
(1S)-OH-(2S)-Glutathionyl-1,2-dihydronaphthalene
(1S)-OH-(2S)-N-Acetylcysteinyl-1,2-dihydronaphthalene
(1R)-N-Acetylcysteinyl-(2R)-OH-1,2-dihydronaphthalene
(1R)-Glutathionyl-(2R)-OH-1,2-dihydronaphthalene
(1R,2R)-3-[(1,2-Dihydro-2-OH-1-naphthalenyl)thio]-2-oxopropanoic acid
Nicotine
NNK
CYP2A6 CYP2A13 CYP2D6 CYP2E1 CYP1A1 CYP3A4
4-[(Hydroxymethyl)-nitrosoamino]-1-(3-pyridinyl)-1-butanone
4-(Nitrosoamino)-1-(3-pyridinyl)-1-butanone
4-Hydroxy-1-(3-pyridinyl)-1-butanone
NNK-N-oxide
4-Hydroxy-4-(methyl-nitrosoamino)-1-(3-pyridinyl)-1-butanone
4-Oxo-1-(3-pyridyl)-1-butanone
3-Succinoylpyridine
1.3.1.20 1.1.1.146 1.1.1.184
N-nitroso-methanamine
NNAL
1-(Methylnitrosoamino)-4-(3-pyridinyl)-1,4-butanediol
5-(3-Pyridyl)-2-hydroxy-tetrahydrofuran
gamma-Hydroxy-3-pyridinebutanoate
NNAL-N-oxide
alpha-[3-(Nitrosoamino)propyl]-3-pyridinemethanol
alpha-[3-[(Hydroxymethyl)-nitrosoamino]propyl]-3-pyridinemethanol
1-(3-Pyridinyl)-1,4-butanediol
2.4.1.17
NNAL-O-glucuronide
NNAL-N-glucuronide
AFM1
CYP1A2 CYP2A13
AFM1-8,9-epoxide
AFB1-endo-8,9-epoxide
AFB1-exo-8,9-epoxide-GSH
CYP3A CYP2A6
AFB1-diol
AFB1-C6-monoaldehyde
AKR7
AFB1-dialcohol
Aflatoxin B1
AFB1-exo-8,9-epoxide
AFB1-dialdehyde
AFB1-C8-monoaldehyde
protein adducts
AFQ1
1-Nitroso-naphthalene
N-Hydroxy-1-aminonaphthalene
1-Naphthylamine
1-Nitro-5-OH-6-glutathionyl-5,6-dihydronaphthalene
1-Nitro-5-glutathionyl-6-OH-5,6-dihydronaphthalene
1-Nitronaphthalene-5,6-oxide
1,2-Dihydro-5-nitro-1,2-naphthalenediol
1-Nitronaphthalene
1-Nitronaphthalene-7,8-oxide
1-Nitro-7-glutathionyl-8-OH-7,8-dihydronaphthalene
1-Nitro-7-OH-8-glutathionyl-7,8-dihydronaphthalene
TCE oxide
Dichloroacetic acid
Dichloroacetyl chloride
1.2.1.5
Trichloroacetic acid
Chloral hydrate
Chloral
Trichloroethylene
1.1.1.1
Trichloroethanol
Trichloroethanol-glucuronide
S-(1,2-Dichlorovinyl)glutathione
2,2-Dichloro-1,1-ethanediol
S-(2,2-Dichloro-1-hydroxy)-ethyl glutathione
2-S-Glutathionyl acetate
2,2-Dichloro-acetaldehyde
1,1-Dichloro-ethylene
DCE epoxide
2-(S-Glutathionyl)-acetyl chloride
Chloroacetyl chloride
S-(2-Chloroacetyl)-glutathione
2-(S-Glutathionyl)-acetyl glutathione
Chloroacetic acid
3,4-Dihydro-3-OH-4-S-glutathionyl bromobenzene
4-Bromophenol
4-Bromophenol-2,3-epoxide
4-Bromocatechol
4-Bromo-1,2-benzoquinone
Bromobenzene-3,4-oxide
Bromobenzene
Bromobenzene-3,4-dihydrodiol
Bromobenzene-2,3-dihydrodiol
Bromobenzene-2,3-oxide
2-Bromophenol
2,3-Dihydro-2-S-glutathionyl-3-hydroxy bromobenzene
1,2-Dibromoethane
2-Bromo-acetaldehyde
S-(Formylmethyl)-glutathione
Thiodiacetic acid
Thiodiacetic acid sulfoxide
Glutathione-episulfonium ion
S-(2-Hydroxyethyl)-glutathione
S-(2-Hydroxyethyl)-N-acetyl-L-cysteine
S-[2-(N7-Guanyl)ethyl]-N-acetyl-L-cysteine
00980 11/11/13
(c) Kanehisa Laboratories

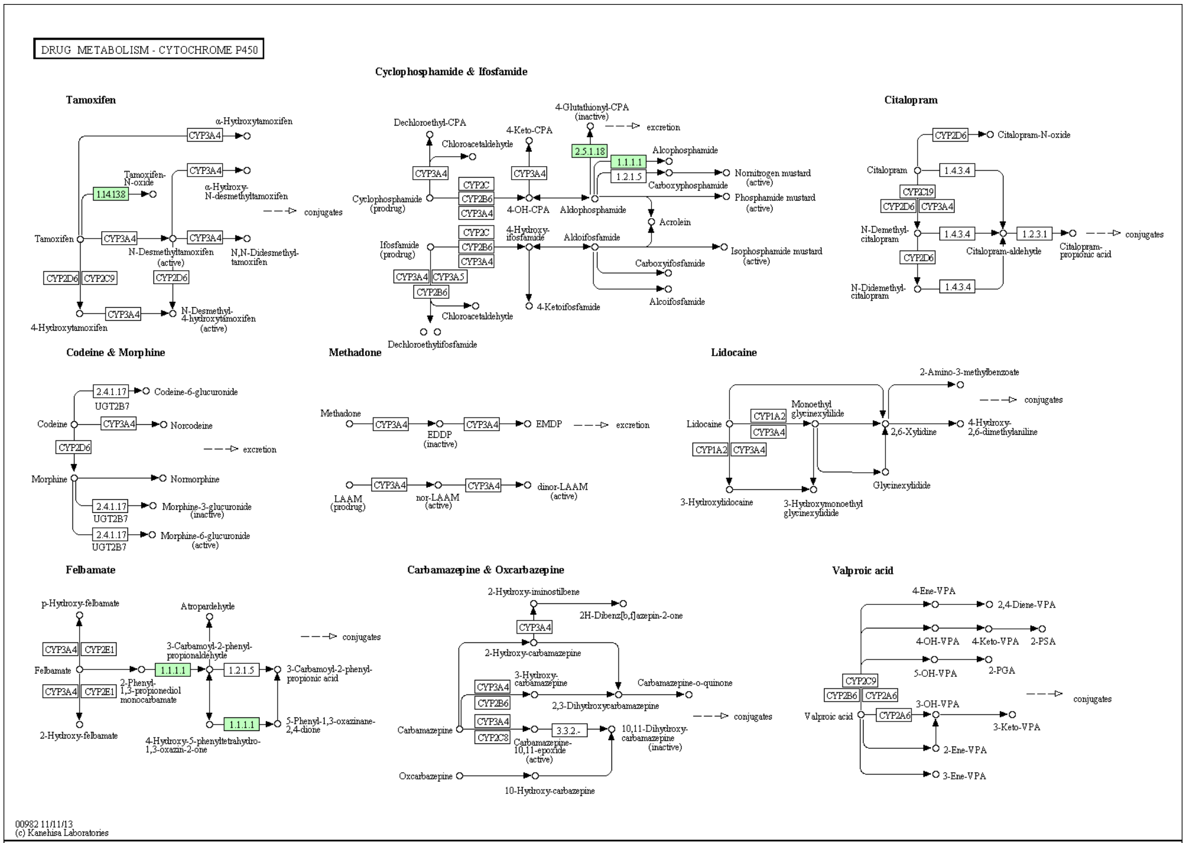
DRUG METABOLISM - CYTOCHROME P450
Cyclophosphamide & Ifosfamide
Tamoxifen
Citalopram
α-Hydroxytamoxifen
CYP3A4
Tamoxifen-N-oxide
1.14.13.8
α-Hydroxy-N-desmethyltamoxifen
conjugates
Tamoxifen
N-Desmethyltamoxifen (active)
N,N-Didesmethyl-tamoxifen
CYP2D6
CYP2C9
4-Hydroxytamoxifen
N-Desmethyl-4-hydroxytamoxifen (active)
Dechloroethyl-CPA
Chloroacetaldehyde
4-Keto-CPA
4-Glutathionyl-CPA (inactive)
excretion
2.5.1.18
1.1.1.1
1.2.1.5
Alcophosphamide
Carboxyphosphamide
Nornitrogen mustard (active)
Phosphamide mustard (active)
Cyclophosphamide (prodrug)
CYP2C
CYP2B6
4-OH-CPA
Aldophosphamide
Acrolein
Ifosfamide (prodrug)
4-Hydroxy-ifosfamide
Aldoifosfamide
Isophosphamide mustard (active)
Carboxyifosfamide
Alcoifosfamide
CYP3A5
4-Ketoifosfamide
Dechloroethylifosfamide
CYP2D6
Citalopram-N-oxide
Citalopram
1.4.3.4
CYP2C19
N-Demethyl-citalopram
1.2.3.1
Citalopram-aldehyde
Citalopram-propionic acid
N-Didemethyl-citalopram
Codeine & Morphine
Methadone
Lidocaine
2.4.1.17
Codeine-6-glucuronide
UGT2B7
Codeine
Norcodeine
Morphine
Normorphine
Morphine-3-glucuronide (inactive)
Morphine-6-glucuronide (active)
Methadone
EDDP (inactive)
EMDP
LAAM (prodrug)
nor-LAAM (active)
dinor-LAAM (active)
2-Amino-3-methylbenzoate
Lidocaine
CYP1A2
Monoethyl glycinexylidide
2,6-Xylidine
4-Hydroxy-2,6-dimethylaniline
Glycinexylidide
3-Hydroxylidocaine
3-Hydroxymonoethyl glycinexylidide
Felbamate
Carbamazepine & Oxcarbazepine
Valproic acid
p-Hydroxy-felbamate
Atropaldehyde
3-Carbamoyl-2-phenyl-propionaldehyde
CYP2E1
Felbamate
2-Phenyl-1,3-propanediol monocarbamate
3-Carbamoyl-2-phenyl-propionic acid
2-Hydroxy-felbamate
4-Hydroxy-5-phenyltetrahydro-1,3-oxazin-2-one
5-Phenyl-1,3-oxazinane-2,4-dione
2-Hydroxy-iminostilbene
2H-Dibenz[b,f]azepin-2-one
2-Hydroxy-carbamazepine
3-Hydroxy-carbamazepine
Carbamazepine-o-quinone
2,3-Dihydroxycarbamazepine
Carbamazepine
CYP2C8
3.3.2.-
Carbamazepine-10,11-epoxide (active)
10,11-Dihydroxy-carbamazepine (inactive)
Oxcarbazepine
10-Hydroxy-carbazepine
4-Ene-VPA
2,4-Diene-VPA
4-OH-VPA
4-Keto-VPA
2-PSA
5-OH-VPA
2-PGA
CYP2A6
3-OH-VPA
3-Keto-VPA
Valproic acid
2-Ene-VPA
3-Ene-VPA
00982 11/11/13
(c) Kanehisa Laboratories

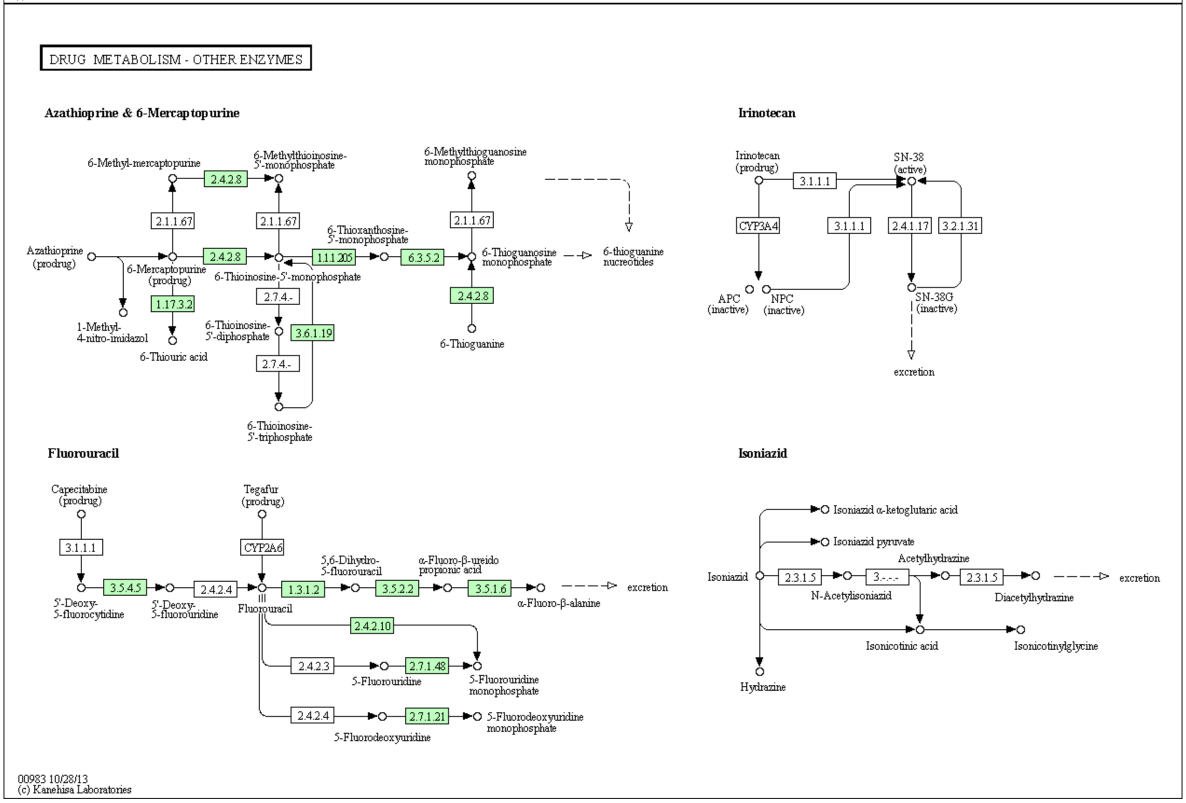
DRUG METABOLISM - OTHER ENZYMES
Azathioprine & 6-Mercaptopurine
Irinotecan
6-Methyl-mercaptopurine
2.4.2.8
6-Methylthioinosine-5'-monophosphate
6-Methylthioguanosine monophosphate
2.1.1.67
6-Thioxanthosine-5'-monophosphate
Azathioprine (prodrug)
6-Mercaptopurine (prodrug)
6-Thioinosine-5'-monophosphate
1.1.1.205
6.3.5.2
6-Thioguanosine monophosphate
6-thioguanine nucreotides
1.17.3.2
2.7.4.-
3.6.1.19
1-Methyl-4-nitro-imidazol
6-Thiouric acid
6-Thioinosine-5'-diphosphate
6-Thioguanine
6-Thioinosine-5'-triphosphate
Irinotecan (prodrug)
3.1.1.1
SN-38 (active)
CYP3A4
2.4.1.17
3.2.1.31
APC (inactive)
NPC (inactive)
SN-38G (inactive)
excretion
Fluorouracil
Isoniazid
Capecitabine (prodrug)
Tegafur (prodrug)
CYP2A6
3.5.4.5
2.4.2.4
5'-Deoxy-5-fluorocytidine
5'-Deoxy-5-fluorouridine
Fluorouracil
1.3.1.2
5,6-Dihydro-5-fluorouracil
3.5.2.2
α-Fluoro-β-ureido propionic acid
3.5.1.6
α-Fluoro-β-alanine
excretion
2.4.2.10
2.4.2.3
2.7.1.48
5-Fluorouridine
5-Fluorouridine monophosphate
2.7.1.21
5-Fluorodeoxyuridine
5-Fluorodeoxyuridine monophosphate
Isoniazid α-ketoglutaric acid
Isoniazid pyruvate
Acetylhydrazine
Isoniazid
2.3.1.5
3.-.-.-
N-Acetylisoniazid
Diacetylhydrazine
Isonicotinic acid
Isonicotinylglycine
Hydrazine
00983 10/28/13
(c) Kanehisa Laboratories

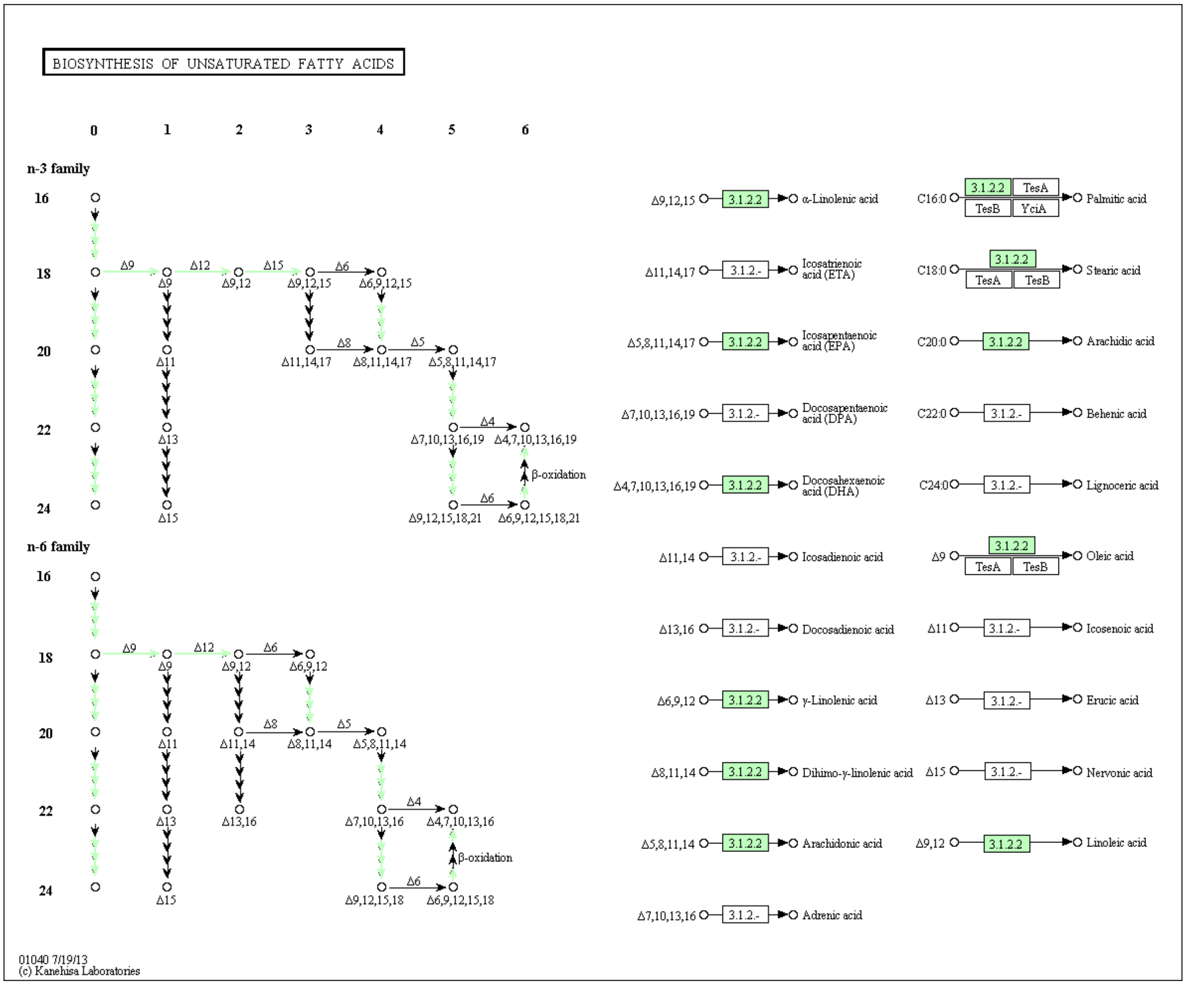

BIOSYNTHESIS OF UNSATURATED FATTY ACIDS
n-3 family
n-6 family
β-oxidation
Δ9,12,15 3.1.2.2 α-Linolenic acid
Δ11,14,17 3.1.2.- Icosatrienoic acid (ETA)
Δ5,8,11,14,17 3.1.2.2 Icosapentaenoic acid (EPA)
Δ7,10,13,16,19 3.1.2.- Docosapentaenoic acid (DPA)
Δ4,7,10,13,16,19 3.1.2.2 Docosahexaenoic acid (DHA)
Δ11,14 3.1.2.- Icosadienoic acid
Δ13,16 3.1.2.- Docosadienoic acid
Δ6,9,12 3.1.2.2 γ-Linolenic acid
Δ8,11,14 3.1.2.2 Dihimo-γ-linolenic acid
Δ5,8,11,14 3.1.2.2 Arachidonic acid
Δ7,10,13,16 3.1.2.- Adrenic acid
C16:0 3.1.2.2 TesA TesB YciA Palmitic acid
C18:0 3.1.2.2 TesA TesB Stearic acid
C20:0 3.1.2.2 Arachidic acid
C22:0 3.1.2.- Behenic acid
C24:0 3.1.2.- Lignoceric acid
Δ9 3.1.2.2 TesA TesB Oleic acid
Δ11 3.1.2.- Icosenoic acid
Δ13 3.1.2.- Erucic acid
Δ15 3.1.2.- Nervonic acid
Δ9,12 3.1.2.2 Linoleic acid
01040 7/19/13
(c) Kanehisa Laboratories

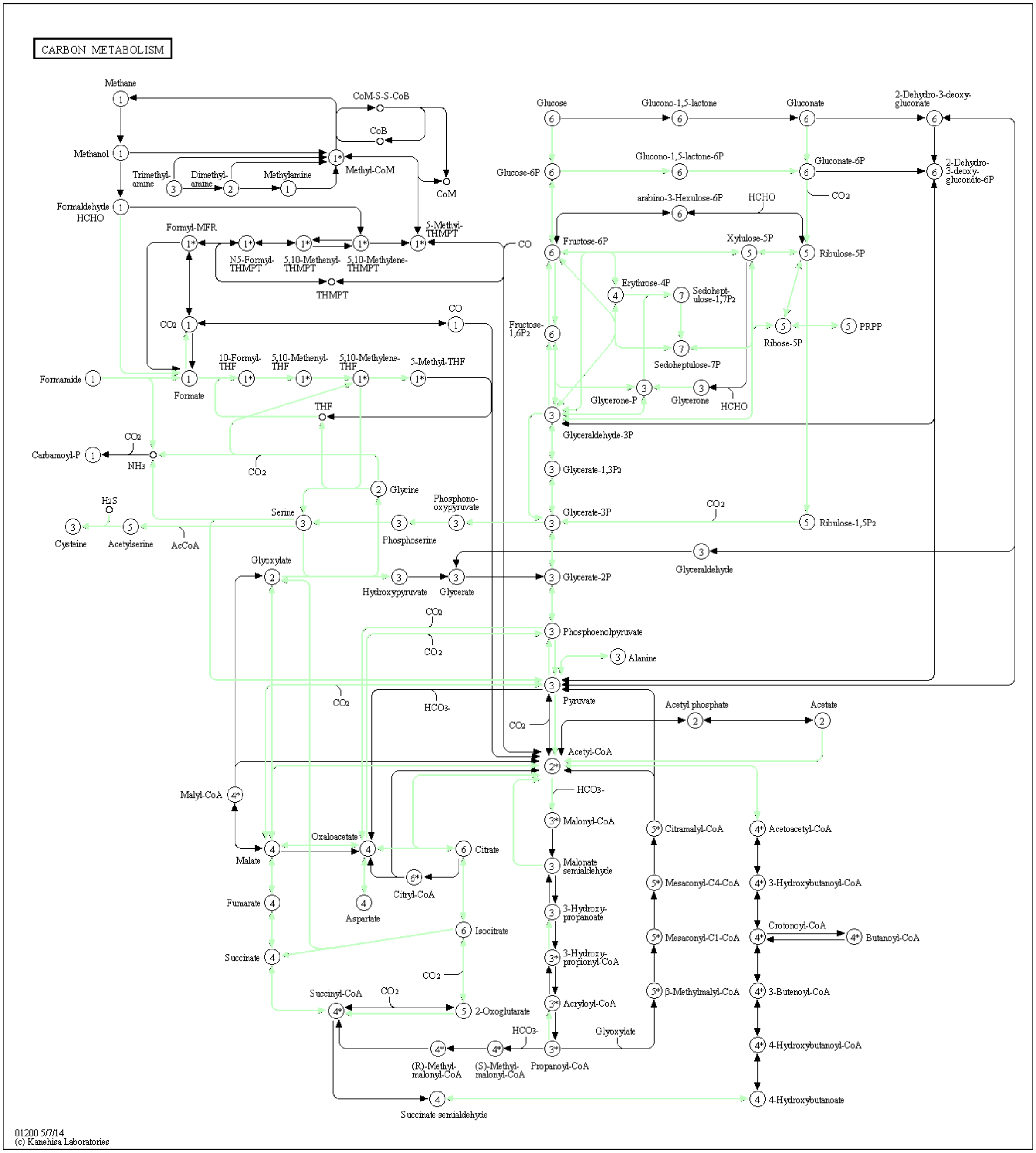
CARBON METABOLISM
Methane
CoM-S-S-CoB
CoB
Methanol
Methyl-CoM
CoM
Trimethyl-amine
Dimethyl-amine
Methylamine
Formaldehyde
HCHO
Formyl-MFR
N5-Formyl-THMPT
5,10-Methenyl-THMPT
5,10-Methylene-THMPT
5-Methyl-THMPT
CO
THMPT
CO2
Formamide
Formate
10-Formyl-THF
5,10-Methenyl-THF
5,10-Methylene-THF
5-Methyl-THF
THF
Carbamoyl-P
NH3
Glycine
Serine
H2S
Cysteine
Acetylserine
AcCoA
Phosphoserine
Phosphono-oxypyruvate
Glyoxylate
Hydroxypyruvate
Glycerate
Glucose
Glucono-1,5-lactone
Gluconate
2-Dehydro-3-deoxy-gluconate
Glucose-6P
Glucono-1,5-lactone-6P
Gluconate-6P
2-Dehydro-3-deoxy-gluconate-6P
arabino-3-Hexulose-6P
Fructose-6P
Xylulose-5P
Ribulose-5P
Erythrose-4P
Sedoheptulose-1,7P2
Fructose-1,6P2
Ribose-5P
PRPP
Sedoheptulose-7P
Glycerone-P
Glycerone
Glyceraldehyde-3P
Glycerate-1,3P2
Glycerate-3P
Ribulose-1,5P2
Glyceraldehyde
Glycerate-2P
Phosphoenolpyruvate
Alanine
Pyruvate
HCO3-
Acetyl phosphate
Acetate
Acetyl-CoA
Malyl-CoA
Malonyl-CoA
Malonate semialdehyde
3-Hydroxy-propanoate
3-Hydroxy-propionyl-CoA
Acryloyl-CoA
Propanoyl-CoA
(R)-Methyl-malonyl-CoA
(S)-Methyl-malonyl-CoA
Citramalyl-CoA
Mesaconyl-C4-CoA
Mesaconyl-C1-CoA
β-Methylmalyl-CoA
Acetoacetyl-CoA
3-Hydroxybutanoyl-CoA
Crotonoyl-CoA
Butanoyl-CoA
3-Butenoyl-CoA
4-Hydroxybutanoyl-CoA
4-Hydroxybutanoate
Malate
Oxaloacetate
Citrate
Citryl-CoA
Fumarate
Aspartate
Isocitrate
Succinate
Succinyl-CoA
2-Oxoglutarate
Succinate semialdehyde
01200 5/7/14
(c) Kanehisa Laboratories

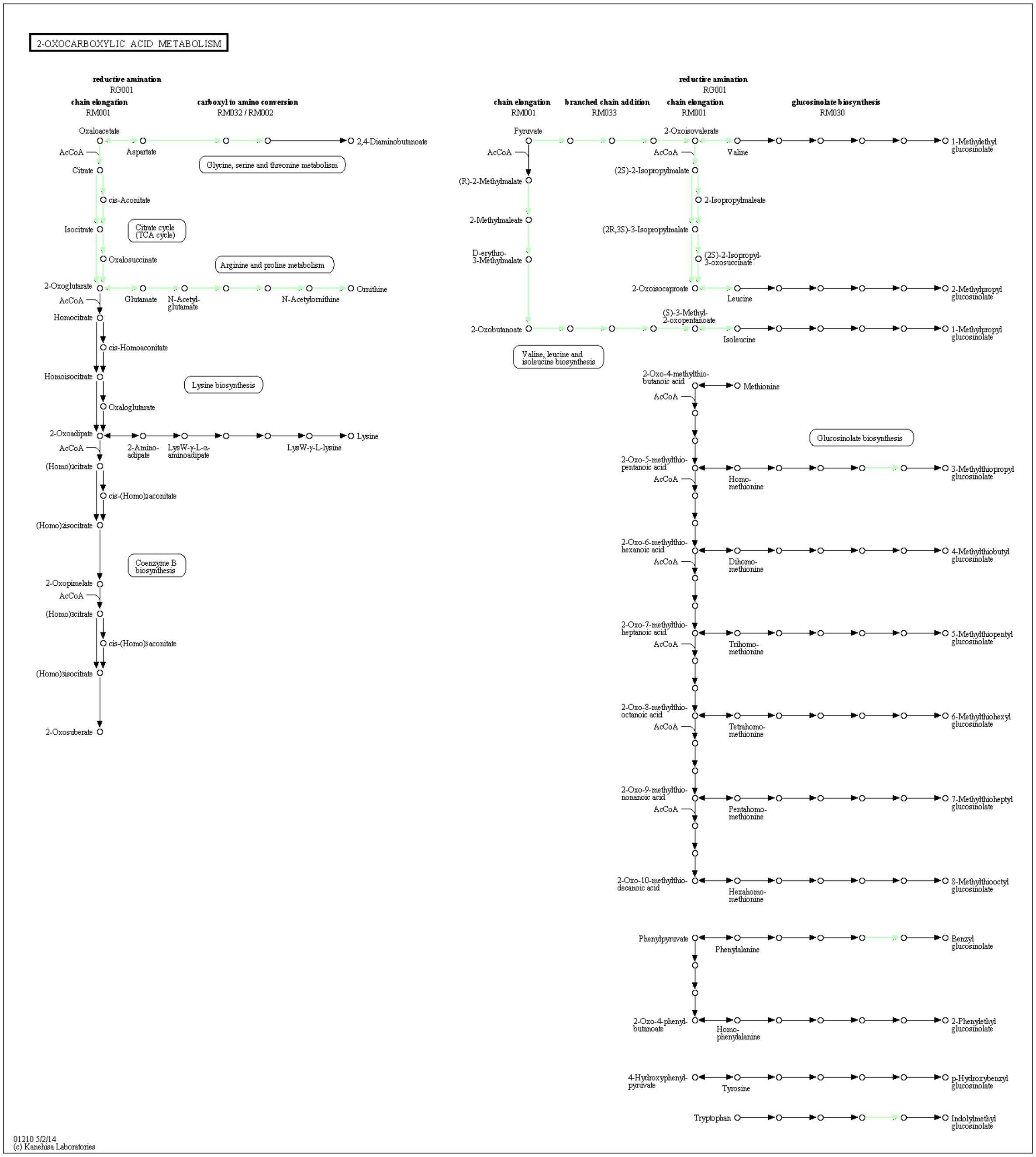
2-OXOCARBOXYLIC ACID METABOLISM
reductive amination
RG001
chain elongation
RM001
carboxyl to amino conversion
RM032 / RM002
branched chain addition
RM033
glucosinolate biosynthesis
RM030
Oxaloacetate
AcCoA
Citrate
cis-Aconitate
Isocitrate
Oxalosuccinate
2-Oxoglutarate
Homocitrate
cis-Homoaconitate
Homoisocitrate
Oxaloglutarate
2-Oxoadipate
(Homo)2citrate
cis-(Homo)2aconitate
(Homo)2isocitrate
2-Oxopimelate
(Homo)3citrate
cis-(Homo)3aconitate
(Homo)3isocitrate
2-Oxosuberate
Aspartate
2,4-Diaminobutanoate
Glycine, serine and threonine metabolism
Citrate cycle (TCA cycle)
Arginine and proline metabolism
Glutamate
N-Acetyl-glutamate
N-Acetylornithine
Ornithine
Lysine biosynthesis
2-Amino-adipate
LysW-γ-L-α-aminoadipate
LysW-γ-L-lysine
Lysine
Coenzyme B biosynthesis
Pyruvate
(R)-2-Methylmalate
2-Methylmaleate
D-erythro-3-Methylmalate
2-Oxobutanoate
Valine, leucine and isoleucine biosynthesis
2-Oxoisovalerate
(2S)-2-Isopropylmalate
2-Isopropylmaleate
(2R,3S)-3-Isopropylmalate
(2S)-2-Isopropyl-3-oxosuccinate
2-Oxoisocaproate
(S)-3-Methyl-2-oxopentanoate
Valine
Leucine
Isoleucine
1-Methylethyl glucosinolate
2-Methylpropyl glucosinolate
1-Methylpropyl glucosinolate
2-Oxo-4-methylthio-butanoic acid
Methionine
Glucosinolate biosynthesis
2-Oxo-5-methylthio-pentanoic acid
Homo-methionine
3-Methylthiopropyl glucosinolate
2-Oxo-6-methylthio-hexanoic acid
Dihomo-methionine
4-Methylthiobutyl glucosinolate
2-Oxo-7-methylthio-heptanoic acid
Trihomo-methionine
5-Methylthiopentyl glucosinolate
2-Oxo-8-methylthio-octanoic acid
Tetrahomo-methionine
6-Methylthiohexyl glucosinolate
2-Oxo-9-methylthio-nonanoic acid
Pentahomo-methionine
7-Methylthioheptyl glucosinolate
2-Oxo-10-methylthio-decanoic acid
Hexahomo-methionine
8-Methylthiooctyl glucosinolate
Phenylpyruvate
Phenylalanine
Benzyl glucosinolate
2-Oxo-4-phenyl-butanoate
Homo-phenylalanine
2-Phenylethyl glucosinolate
4-Hydroxyphenyl-pyruvate
Tyrosine
p-Hydroxybenzyl glucosinolate
Tryptophan
Indolylmethyl glucosinolate
01210 5/2/14
(c) Kanehisa Laboratories

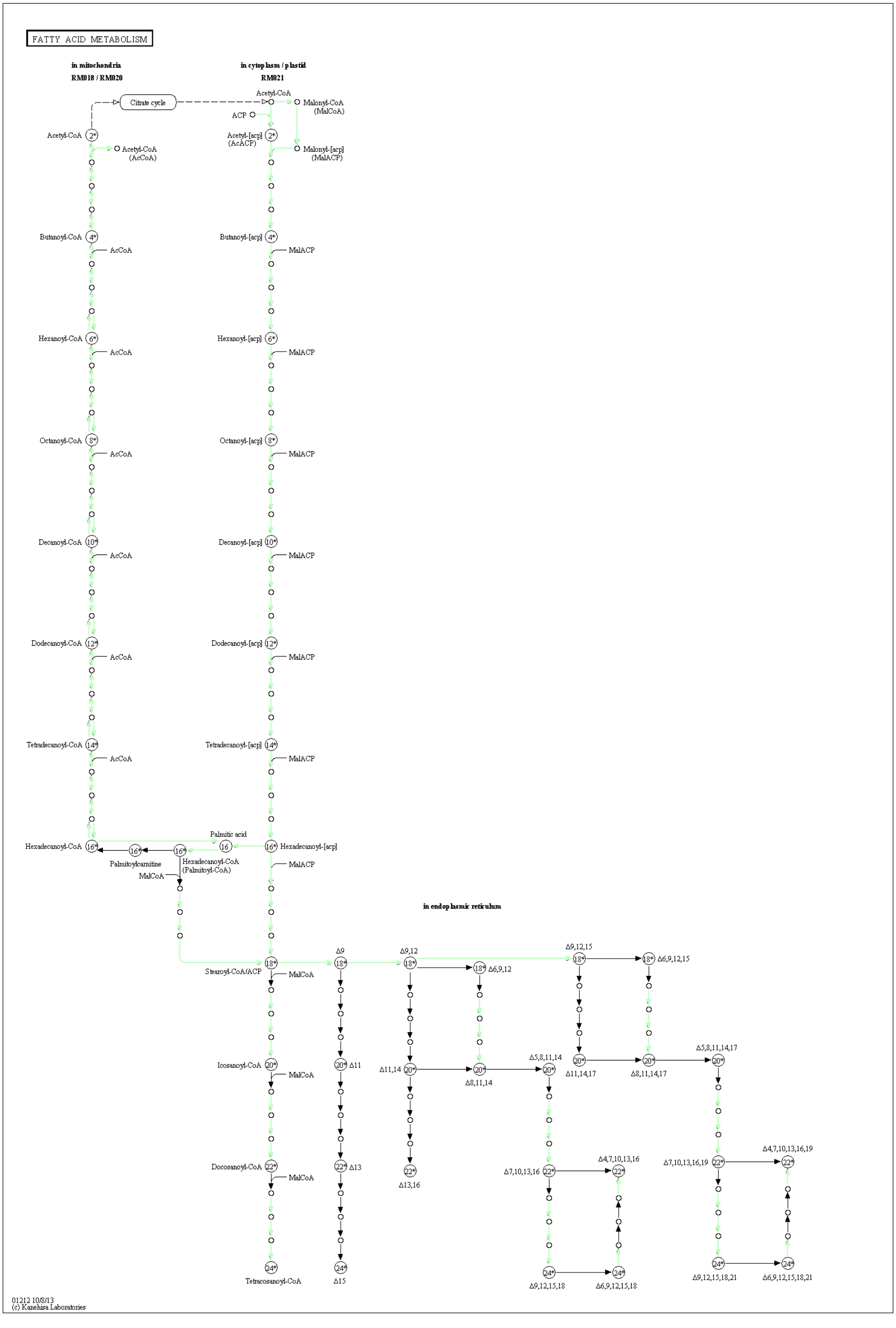
FATTY ACID METABOLISM
in mitochondria
RM018 / RM020
in cytoplasm / plastid
RM021
Citrate cycle
Acetyl-CoA
Malonyl-CoA (MalCoA)
ACP
Acetyl-[acp] (AcACP)
Malonyl-[acp] (MalACP)
Acetyl-CoA (AcCoA)
Butanoyl-CoA
Butanoyl-[acp]
Hexanoyl-CoA
Hexanoyl-[acp]
Octanoyl-CoA
Octanoyl-[acp]
Decanoyl-CoA
Decanoyl-[acp]
Dodecanoyl-CoA
Dodecanoyl-[acp]
Tetradecanoyl-CoA
Tetradecanoyl-[acp]
AcCoA
MalACP
Palmitic acid
Hexadecanoyl-CoA
Hexadecanoyl-[acp]
Palmitoylcarnitine
Hexadecanoyl-CoA (Palmitoyl-CoA)
MalCoA
in endoplasmic reticulum
Stearoyl-CoA/ACP
Icosanoyl-CoA
Docosanoyl-CoA
Tetracosanoyl-CoA
Δ9
Δ11
Δ13
Δ15
Δ9,12
Δ6,9,12
Δ11,14
Δ8,11,14
Δ13,16
Δ5,8,11,14
Δ7,10,13,16
Δ4,7,10,13,16
Δ9,12,15,18
Δ6,9,12,15,18
Δ9,12,15
Δ6,9,12,15
Δ11,14,17
Δ8,11,14,17
Δ5,8,11,14,17
Δ7,10,13,16,19
Δ4,7,10,13,16,19
Δ9,12,15,18,21
Δ6,9,12,15,18,21
01212 10/8/13
(c) Kanehisa Laboratories

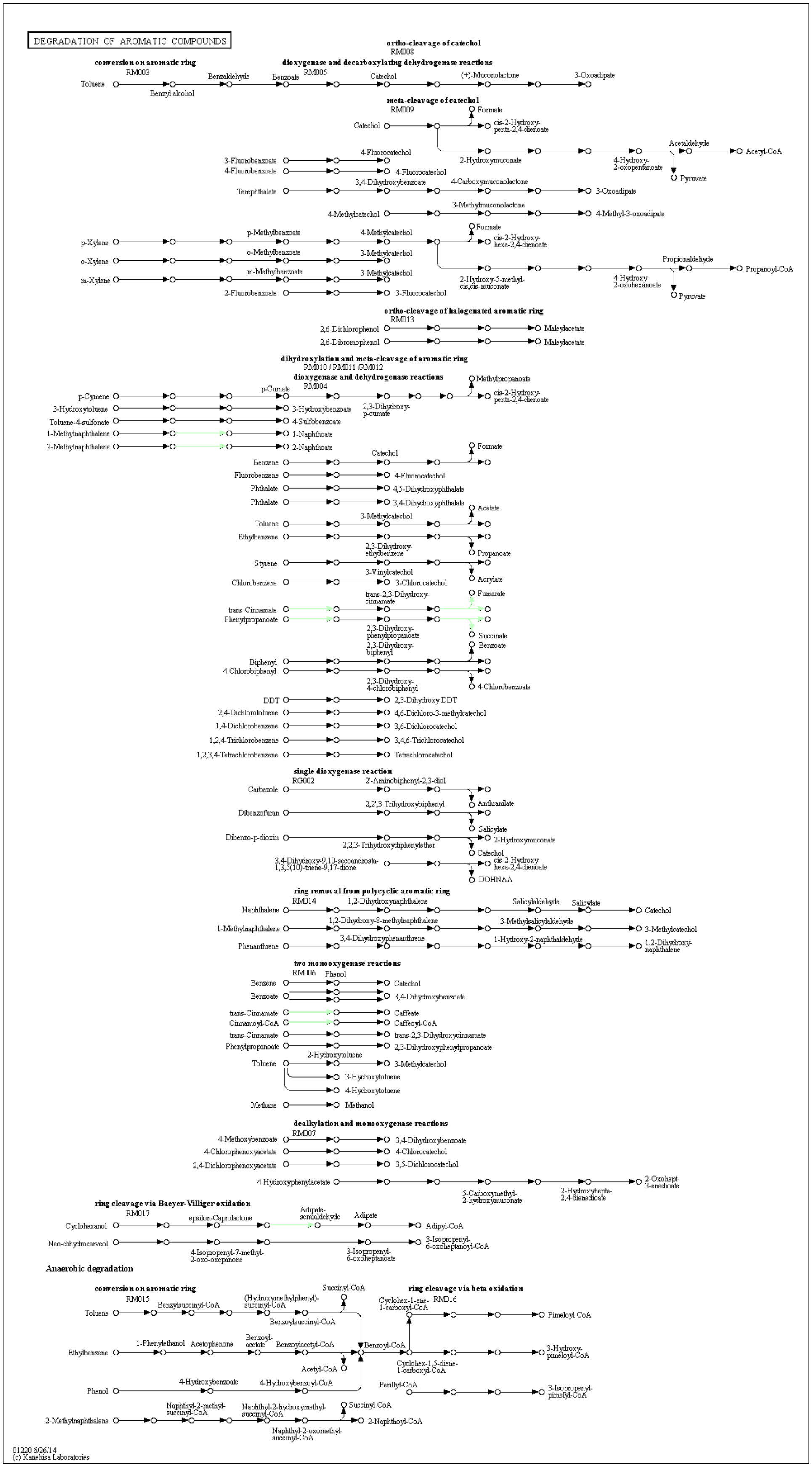
DEGRADATION OF AROMATIC COMPOUNDS
ortho-cleavage of catechol
RM008
conversion on aromatic ring
RM003
dioxygenase and decarboxylating dehydrogenase reactions
RM005
Toluene
Benzyl alcohol
Benzaldehyde
Benzoate
Catechol
(+)-Muconolactone
3-Oxoadipate
meta-cleavage of catechol
RM009
Formate
cis-2-Hydroxy-penta-2,4-dienoate
2-Hydroxymuconate
4-Hydroxy-2-oxopentanoate
Acetaldehyde
Acetyl-CoA
Pyruvate
3-Fluorobenzoate
4-Fluorobenzoate
4-Fluorocatechol
Terephthalate
3,4-Dihydroxybenzoate
4-Carboxymuconolactone
3-Oxoadipate
4-Methylcatechol
3-Methylmuconolactone
4-Methyl-3-oxoadipate
p-Xylene
o-Xylene
m-Xylene
p-Methylbenzoate
o-Methylbenzoate
m-Methylbenzoate
3-Methylcatechol
cis-2-Hydroxy-hexa-2,4-dienoate
2-Hydroxy-5-methyl-cis,cis-muconate
4-Hydroxy-2-oxohexanoate
Propionaldehyde
Propanoyl-CoA
2-Fluorobenzoate
3-Fluorocatechol
ortho-cleavage of halogenated aromatic ring
RM013
2,6-Dichlorophenol
2,6-Dibromophenol
Maleylacetate
dihydroxylation and meta-cleavage of aromatic ring
RM010 / RM011 /RM012
dioxygenase and dehydrogenase reactions
RM004
p-Cymene
3-Hydroxytoluene
Toluene-4-sulfonate
1-Methylnaphthalene
2-Methylnaphthalene
p-Cumate
2,3-Dihydroxy-p-cumate
Methylpropanoate
3-Hydroxybenzoate
4-Sulfobenzoate
1-Naphthoate
2-Naphthoate
Benzene
Fluorobenzene
Phthalate
4,5-Dihydroxyphthalate
3,4-Dihydroxyphthalate
Acetate
Ethylbenzene
2,3-Dihydroxy-ethylbenzene
Propanoate
Styrene
3-Vinylcatechol
Acrylate
Chlorobenzene
3-Chlorocatechol
trans-Cinnamate
Phenylpropanoate
trans-2,3-Dihydroxy-cinnamate
2,3-Dihydroxy-phenylpropanoate
Fumarate
Succinate
Biphenyl
4-Chlorobiphenyl
2,3-Dihydroxy-biphenyl
2,3-Dihydroxy-4-chlorobiphenyl
4-Chlorobenzoate
DDT
2,3-Dihydroxy DDT
2,4-Dichlorotoluene
4,6-Dichloro-3-methylcatechol
1,4-Dichlorobenzene
3,6-Dichlorocatechol
1,2,4-Trichlorobenzene
3,4,6-Trichlorocatechol
1,2,3,4-Tetrachlorobenzene
Tetrachlorocatechol
single dioxygenase reaction
RG002
Carbazole
2'-Aminobiphenyl-2,3-diol
Anthranilate
Dibenzofuran
2,2',3-Trihydroxybiphenyl
Salicylate
Dibenzo-p-dioxin
2,2,3-Trihydroxydiphenylether
2-Hydroxymuconate
3,4-Dihydroxy-9,10-secoandrosta-1,3,5(10)-triene-9,17-dione
cis-2-Hydroxy-hexa-2,4-dienoate
DOHNAA
ring removal from polycyclic aromatic ring
RM014
Naphthalene
1,2-Dihydroxynaphthalene
Salicylaldehyde
Salicylate
1-Methylnaphthalene
1,2-Dihydroxy-8-methylnaphthalene
3-Methylsalicylaldehyde
3-Methylcatechol
Phenanthrene
3,4-Dihydroxyphenanthrene
1-Hydroxy-2-naphthaldehyde
1,2-Dihydroxy-naphthalene
two monooxygenase reactions
RM006
Phenol
Caffeate
Cinnamoyl-CoA
Caffeoyl-CoA
trans-2,3-Dihydroxycinnamate
2,3-Dihydroxyphenylpropanoate
2-Hydroxytoluene
3-Hydroxytoluene
4-Hydroxytoluene
Methane
Methanol
dealkylation and monooxygenase reactions
RM007
4-Methoxybenzoate
4-Chlorophenoxyacetate
4-Chlorocatechol
2,4-Dichlorophenoxyacetate
3,5-Dichlorocatechol
4-Hydroxyphenylacetate
5-Carboxymethyl-2-hydroxymuconate
2-Hydroxyhepta-2,4-dienedioate
2-Oxohept-3-enedioate
ring cleavage via Baeyer-Villiger oxidation
RM017
Cyclohexanol
epsilon-Caprolactone
Adipate-semialdehyde
Adipate
Adipyl-CoA
Neo-dihydrocarveol
4-Isopropenyl-7-methyl-2-oxo-oxepanone
3-Isopropenyl-6-oxoheptanoate
3-Isopropenyl-6-oxoheptanoyl-CoA
Anaerobic degradation
conversion on aromatic ring
RM015
Benzylsuccinyl-CoA
(Hydroxymethylphenyl)-succinyl-CoA
Succinyl-CoA
Benzoylsuccinyl-CoA
ring cleavage via beta oxidation
Cyclohex-1-ene-1-carboxyl-CoA
RM016
Pimeloyl-CoA
1-Phenylethanol
Acetophenone
Benzoyl-acetate
Benzoylacetyl-CoA
Benzoyl-CoA
Acetyl-CoA
Cyclohex-1,5-diene-1-carboxyl-CoA
3-Hydroxy-pimeloyl-CoA
Phenol
4-Hydroxybenzoate
4-Hydroxybenzoyl-CoA
Perillyl-CoA
3-Isopropenyl-pimelyl-CoA
2-Methylnaphthalene
Naphthyl-2-methyl-succinyl-CoA
Naphthyl-2-hydroxymethyl-succinyl-CoA
Naphthyl-2-oxomethyl-succinyl-CoA
2-Naphthoyl-CoA
01220 6/26/14
(c) Kanehisa Laboratories

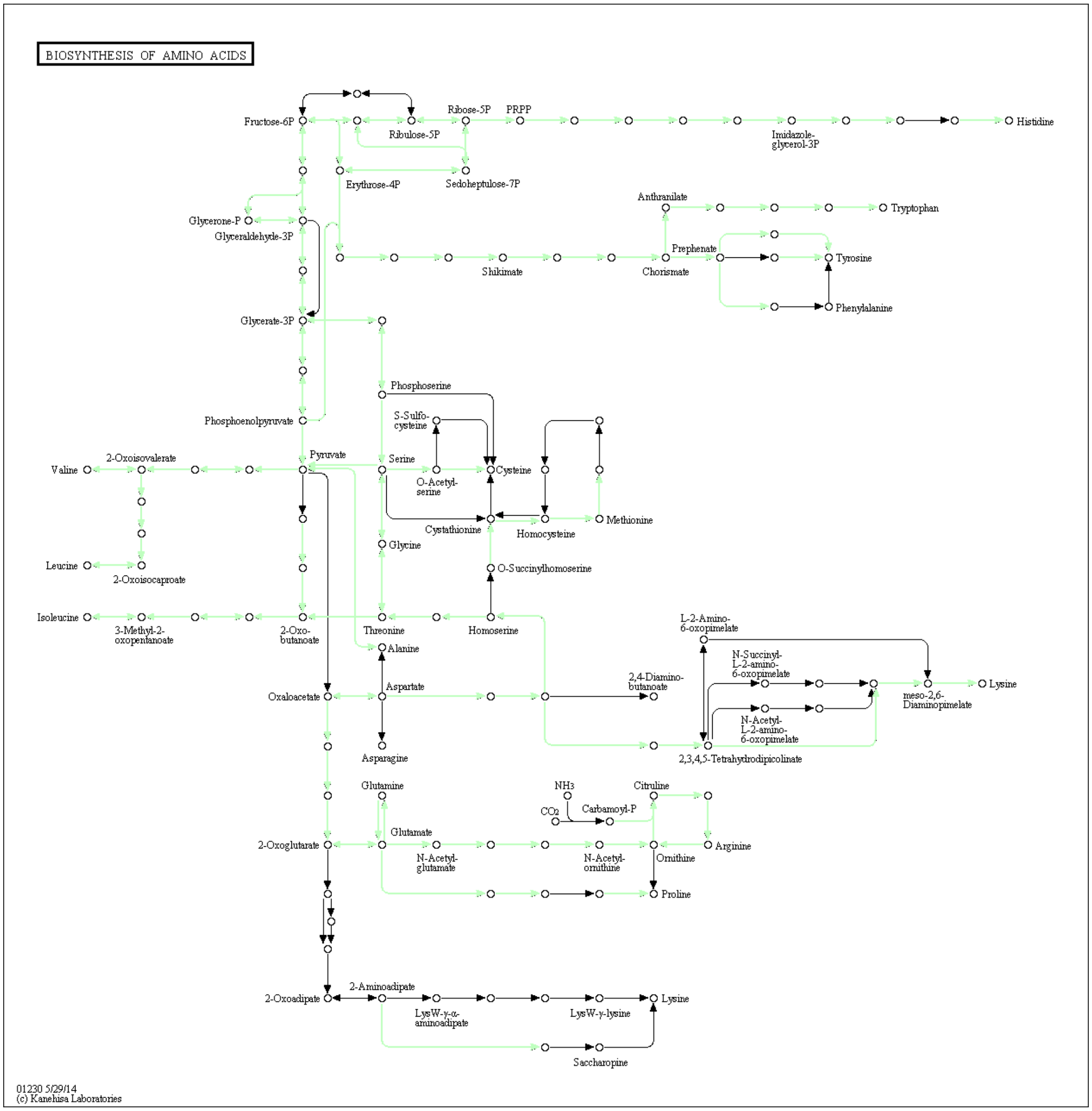
BIOSYNTHESIS OF AMINO ACIDS
Fructose-6P
Ribose-5P
PRPP
Ribulose-5P
Imidazole-
glycerol-3P
Histidine
Erythrose-4P
Sedoheptulose-7P
Anthranilate
Tryptophan
Glycerone-P
Glyceraldehyde-3P
Prephenate
Tyrosine
Shikimate
Chorismate
Phenylalanine
Glycerate-3P
Phosphoserine
S-Sulfo-
cysteine
Phosphoenolpyruvate
2-Oxoisovalerate
Pyruvate
Serine
Valine
Cysteine
O-Acetyl-
serine
Cystathionine
Methionine
Homocysteine
Glycine
Leucine
2-Oxoisocaproate
O-Succinylhomoserine
Isoleucine
3-Methyl-2-
oxopentanoate
2-Oxo-
butanoate
Threonine
Homoserine
L-2-Amino-
6-oxopimelate
Alanine
N-Succinyl-
L-2-amino-
6-oxopimelate
2,4-Diamino-
butanoate
Aspartate
Oxaloacetate
meso-2,6-
Diaminopimelate
Lysine
N-Acetyl-
L-2-amino-
6-oxopimelate
Asparagine
2,3,4,5-Tetrahydrodipicolinate
Glutamine
NH3
Citruline
CO2
Carbamoyl-P
Glutamate
2-Oxoglutarate
Arginine
N-Acetyl-
glutamate
N-Acetyl-
ornithine
Ornithine
Proline
2-Aminoadipate
2-Oxoadipate
LysW-γ-α-
aminoadipate
LysW-γ-lysine
Lysine
Saccharopine
01230 5/29/14
(c) Kanehisa Laboratories

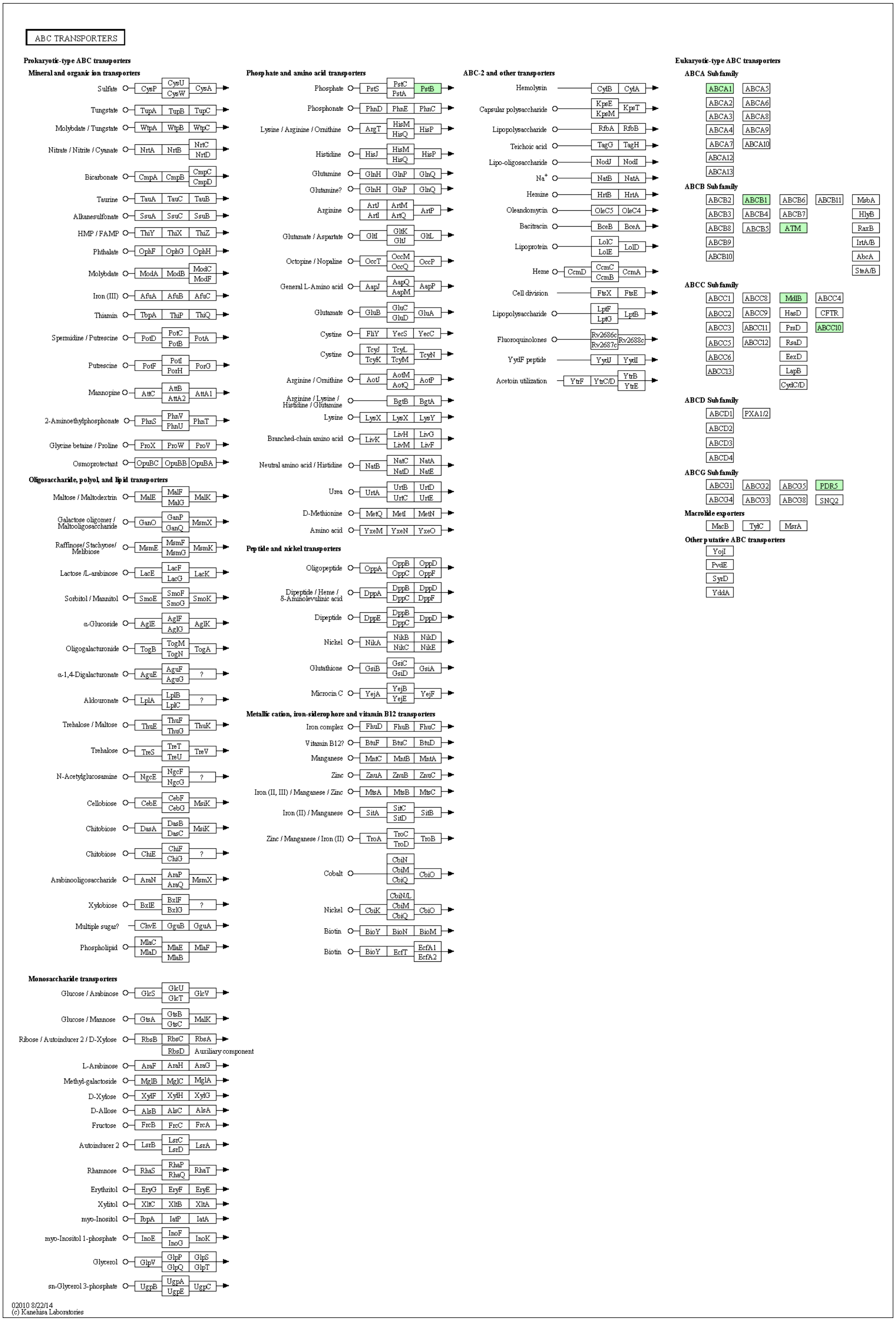
ABC TRANSPORTERS
Prokaryotic-type ABC transporters
Mineral and organic ion transporters
Sulfate CysP CysU CysW CysA
Tungstate TupA TupB TupC
Molybdate / Tungstate WtpA WtpB WtpC
Nitrate / Nitrite / Cyanate NrtA NrtB NrtC NrtD
Bicarbonate CmpA CmpB CmpC CmpD
Taurine TauA TauC TauB
Alkanesulfonate SsuA SsuC SsuB
HMP / FAMP ThiY ThiX ThiZ
Phthalate OphF OphG OphH
Molybdate ModA ModB ModC ModF
Iron (III) AfuA AfuB AfuC
Thiamin TbpA ThiP ThiQ
Spermidine / Putrescine PotD PotC PotB PotA
Putrescine PotF PotI PotH PotG
Mannopine AttC AttB AttA2 AttA1
2-Aminoethylphosphonate PhnS PhnV PhnU PhnT
Glycine betaine / Proline ProX ProW ProV
Osmoprotectant OpuBC OpuBB OpuBA
Oligosaccharide, polyol, and lipid transporters
Maltose / Maltodextrin MalE MalF MalG MalK
Galactose oligomer / Maltooligosaccharide GanO GanP GanQ MsmX
Raffinose/ Stachyose/ Melibiose MsmE MsmF MsmG MsmK
Lactose /L-arabinose LacE LacF LacG LacK
Sorbitol / Mannitol SmoE SmoF SmoG SmoK
α-Glucoside AglE AglF AglG AglK
Oligogalacturonide TogB TogM TogN TogA
α-1,4-Digalacturonate AguE AguF AguG ?
Aldouronate LplA LplB LplC ?
Trehalose / Maltose ThuE ThuF ThuG ThuK
Trehalose TreS TreT TreU TreV
N-Acetylglucosamine NgcE NgcF NgcG ?
Cellobiose CebE CebF CebG MsiK
Chitobiose DasA DasB DasC MsiK
Chitobiose ChiE ChiF ChiG ?
Arabinooligosaccharide AraN AraP AraQ MsmX
Xylobiose BxlE BxlF BxlG ?
Multiple sugar? ChvE GguB GguA
Phospholipid MlaC MlaD MlaE MlaB MlaF
Monosaccharide transporters
Glucose / Arabinose GlcS GlcU GlcT GlcV
Glucose / Mannose GtsA GtsB GtsC MalK
Ribose / Autoinducer 2 / D-Xylose RbsB RbsC RbsA RbsD Auxiliary component
L-Arabinose AraF AraH AraG
Methyl-galactoside MglB MglC MglA
D-Xylose XylF XylH XylG
D-Allose AlsB AlsC AlsA
Fructose FrcB FrcC FrcA
Autoinducer 2 LsrB LsrC LsrD LsrA
Rhamnose RhaS RhaP RhaQ RhaT
Erythritol EryG EryF EryE
Xylitol XltC XltB XltA
myo-Inositol IbpA IatP IatA
myo-Inositol 1-phosphate InoE InoF InoG InoK
Glycerol GlpV GlpP GlpQ GlpS GlpT
sn-Glycerol 3-phosphate UgpB UgpA UgpE UgpC
Phosphate and amino acid transporters
Phosphate PstS PstC PstA PstB
Phosphonate PhnD PhnE PhnC
Lysine / Arginine / Ornithine ArgT HisM HisQ HisP
Histidine HisJ HisM HisQ HisP
Glutamine GlnH GlnP GlnQ
Glutamine? GlnH GlnP GlnQ
Arginine ArtJ ArtI ArtM ArtQ ArtP
Glutamate / Aspartate GltI GltK GltJ GltL
Octopine / Nopaline OccT OccM OccQ OccP
General L-Amino acid AapJ AapQ AapM AapP
Glutamate GluB GluC GluD GluA
Cystine FliY YecS YecC
Cystine TcyJ TcyK TcyL TcyM TcyN
Arginine / Ornithine AotJ AotM AotQ AotP
Arginine / Lysine / Histidine / Glutamine BgtB BgtA
Lysine LysX LysX LysY
Branched-chain amino acid LivK LivH LivM LivG LivF
Neutral amino acid / Histidine NatB NatC NatD NatA NatE
Urea UrtA UrtB UrtC UrtD UrtE
D-Methionine MetQ MetI MetN
Amino acid YxeM YxeN YxeO
Peptide and nickel transporters
Oligopeptide OppA OppB OppC OppD OppF
Dipeptide / Heme / δ-Aminolevulinic acid DppA DppB DppC DppD DppF
Dipeptide DppE DppB DppC DppD
Nickel NikA NikB NikC NikD NikE
Glutathione GsiB GsiC GsiD GsiA
Microcin C YejA YejB YejE YejF
Metallic cation, iron-siderophore and vitamin B12 transporters
Iron complex FhuD FhuB FhuC
Vitamin B12? BtuF BtuC BtuD
Manganese MntC MntB MntA
Zinc ZnuA ZnuB ZnuC
Iron (II, III) / Manganese / Zinc MtsA MtsB MtsC
Iron (II) / Manganese SitA SitC SitD SitB
Zinc / Manganese / Iron (II) TroA TroC TroD TroB
Cobalt CbiN CbiM CbiQ CbiO
Nickel CbiK CbiN/L CbiM CbiQ CbiO
Biotin BioY BioN BioM
Biotin BioY EcfT EcfA1 EcfA2
ABC-2 and other transporters
Hemolysin CylB CylA
Capsular polysaccharide KpsE KpsM KpsT
Lipopolysaccharide RfbA RfbB
Teichoic acid TagG TagH
Lipo-oligosaccharide NodJ NodI
Na+ NatB NatA
Hemine HrtB HrtA
Oleandomycin OleC5 OleC4
Bacitracin BceB BceA
Lipoprotein LolC LolE LolD
Heme CcmD CcmC CcmB CcmA
Cell division FtsX FtsE
Lipopolysaccharide LptF LptG LptB
Fluoroquinolones Rv2686c Rv2687c Rv2688c
YydF peptide YydJ YydI
Acetoin utilization YtrF YtrC/D YtrB YtrE
Eukaryotic-type ABC transporters
ABCA Subfamily
ABCA1 ABCA5 ABCA2 ABCA6 ABCA3 ABCA8 ABCA4 ABCA9 ABCA7 ABCA10 ABCA12 ABCA13
ABCB Subfamily
ABCB2 ABCB1 ABCB6 ABCB11 MsbA ABCB3 ABCB4 ABCB7 HlyB ABCB8 ABCB5 ATM RaxB ABCB9 IrtA/B ABCB10 AbcA SteA/B
ABCC Subfamily
ABCC1 ABCC8 MdlB ABCC4 ABCC2 ABCC9 HasD CFTR ABCC3 ABCC11 PrsD ABCC10 ABCC5 ABCC12 RsaD ABCC6 EexD ABCC13 LapB CydC/D
ABCD Subfamily
ABCD1 PXA1/2 ABCD2 ABCD3 ABCD4
ABCG Subfamily
ABCG1 ABCG2 ABCG5 PDR5 ABCG4 ABCG3 ABCG8 SNQ2
Macrolide exporters
MacB TylC MsrA
Other putative ABC transporters
YojI PvdE SyrD YddA
02010 8/22/14
(c) Kanehisa Laboratories

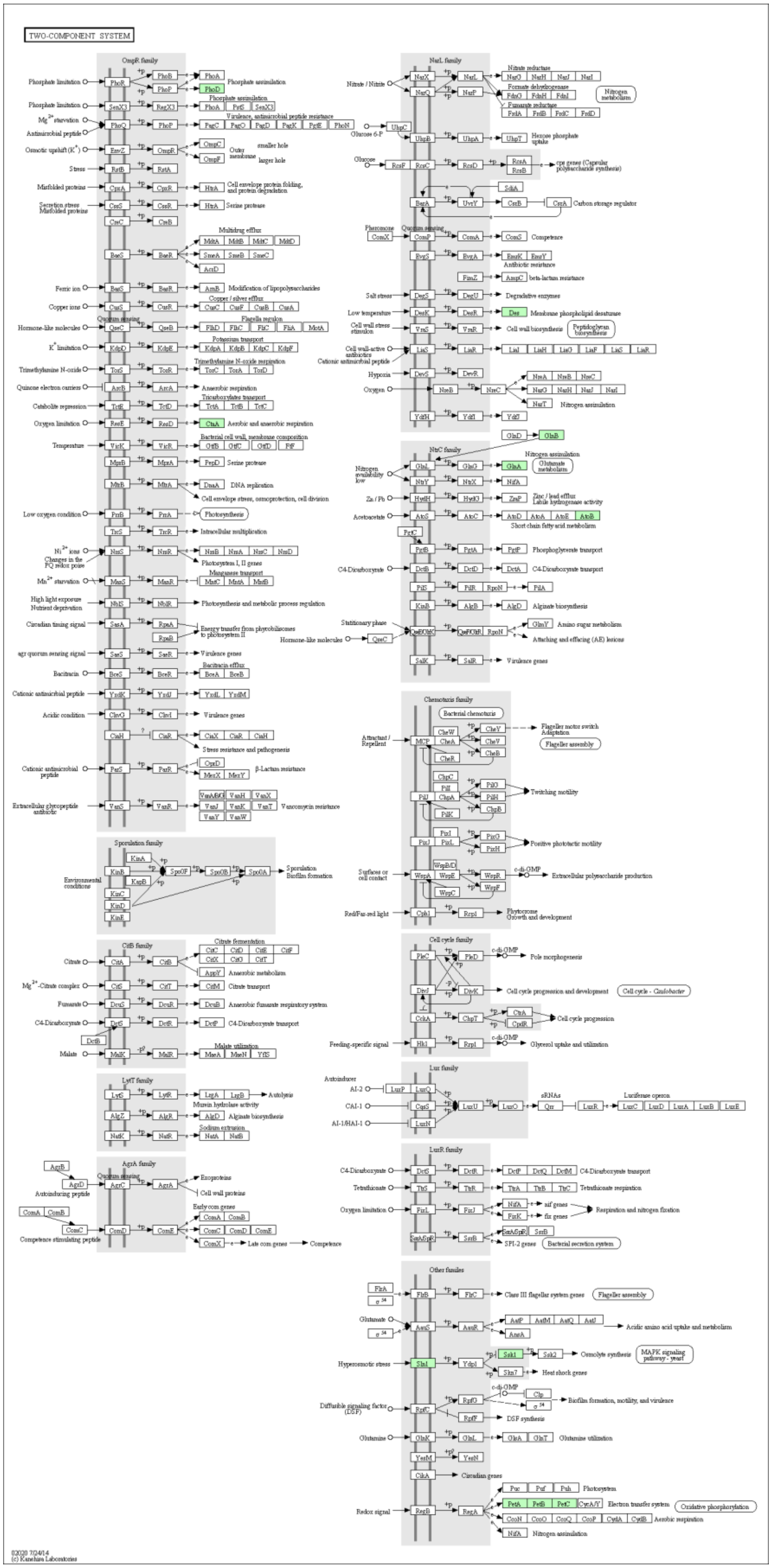

TWO-COMPONENT SYSTEM
02020 7/24/14
(c) Kanehisa Laboratories

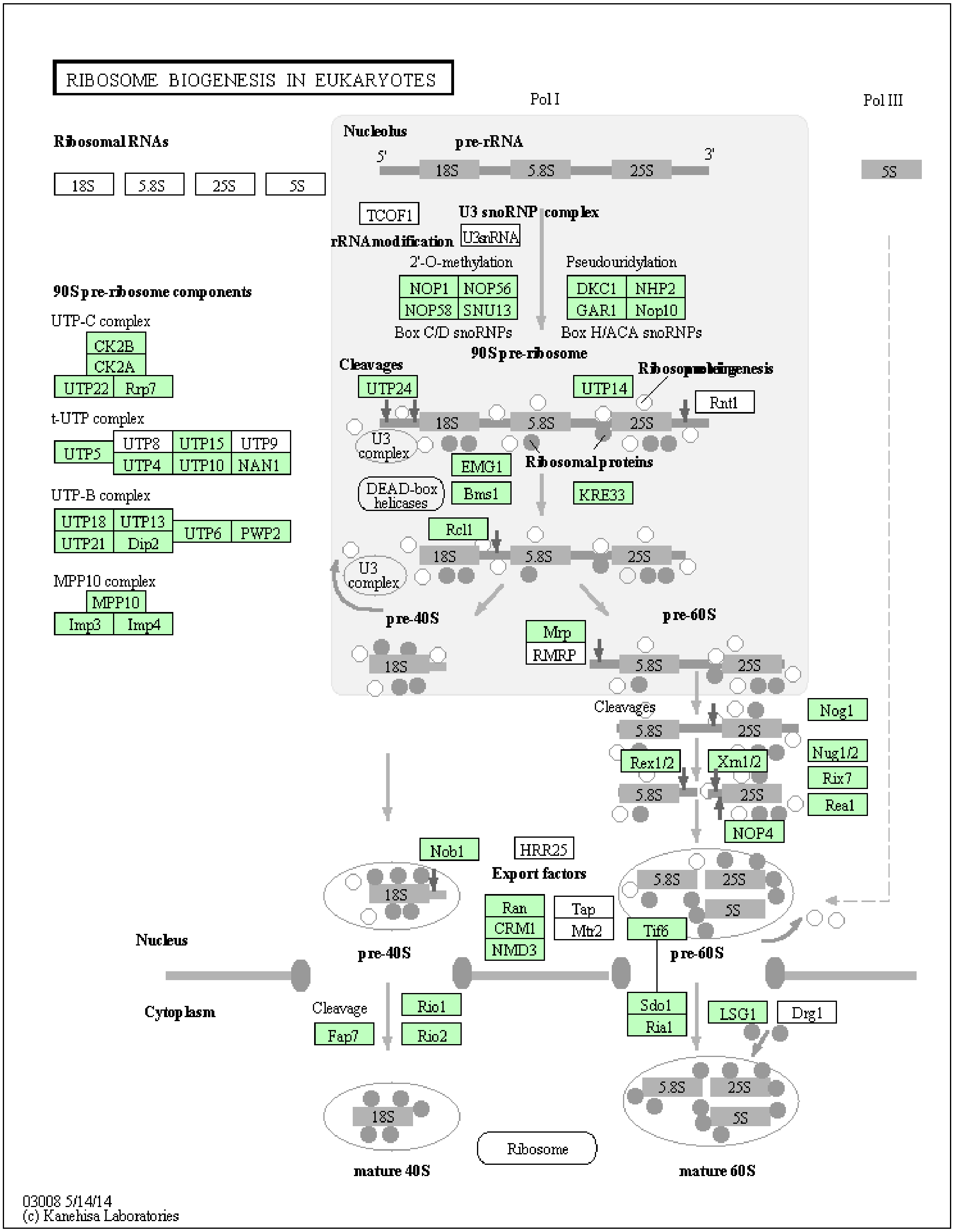
RIBOSOME BIOGENESIS IN EUKARYOTES
Pol I
Pol III
Ribosomal RNAs
18S
5.8S
25S
5S
Nucleolus
pre-rRNA
5'
3'
TCOF1
U3 snoRNP complex
U3snRNA
rRNA modification
2'-O-methylation
Pseudouridylation
NOP1
NOP56
NOP58
SNU13
DKC1
NHP2
GAR1
Nop10
Box C/D snoRNPs
Box H/ACA snoRNPs
90S pre-ribosome components
UTP-C complex
CK2B
CK2A
UTP22
Rrp7
t-UTP complex
UTP5
UTP8
UTP15
UTP9
UTP4
UTP10
NAN1
UTP-B complex
UTP18
UTP13
UTP21
Dip2
UTP6
PWP2
MPP10 complex
MPP10
Imp3
Imp4
90S pre-ribosome
Cleavages
UTP24
UTP14
Rnt1
U3 complex
EMG1
Ribosomal proteins
DEAD-box helicases
Bms1
KRE33
Rcl1
pre-40S
pre-60S
Mrp
RMRP
Nog1
Rex1/2
Xrn1/2
Nug1/2
Rix7
Rea1
NOP4
Nob1
HRR25
Export factors
Ran
CRM1
NMD3
Tap
Mtr2
Tif6
Nucleus
Cytoplasm
Cleavage
Rio1
Fap7
Rio2
Sdo1
Ria1
LSG1
Drg1
Ribosome
mature 40S
mature 60S
03008 5/14/14
(c) Kanehisa Laboratories

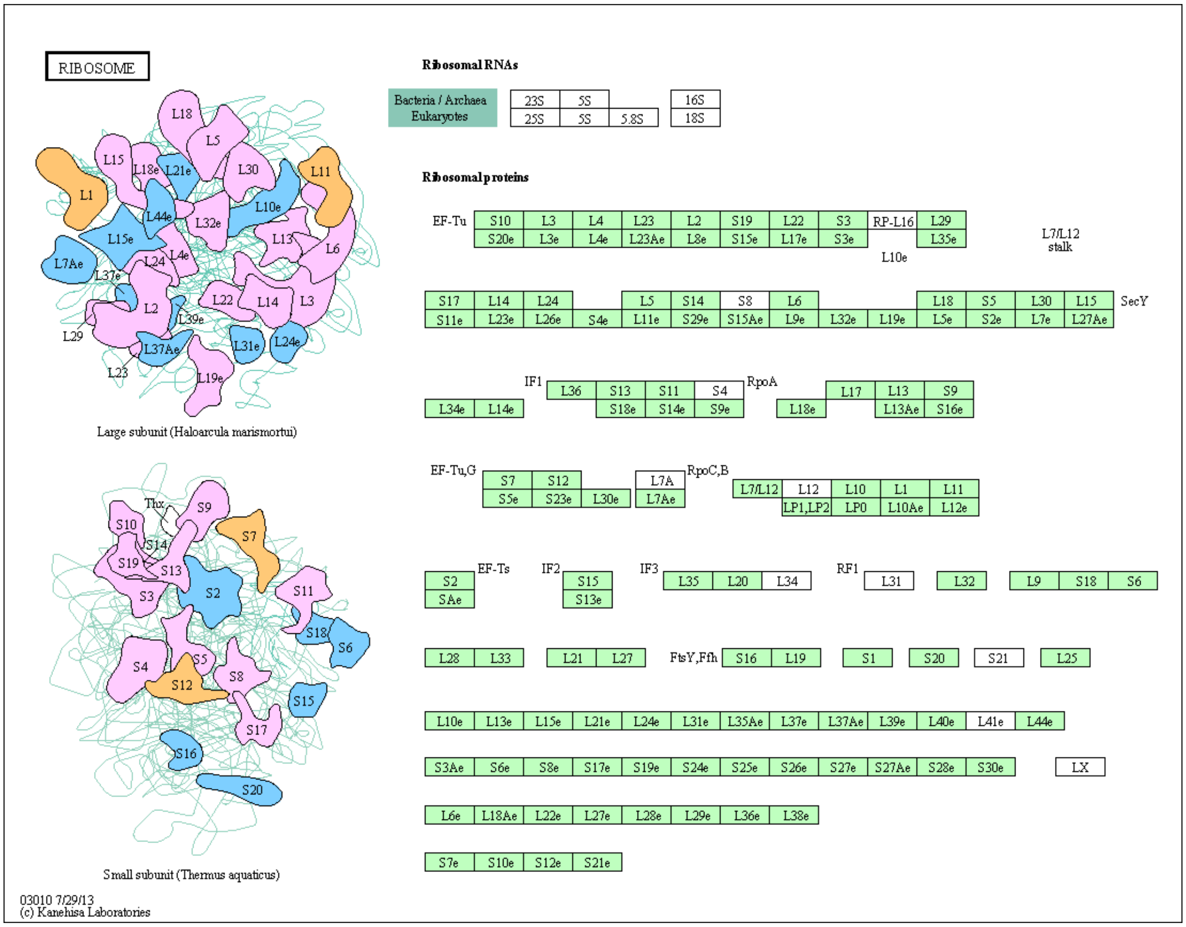
RIBOSOME
L18
L5
L15
L18e
L21e
L30
L11
L1
L44e
L10e
L32e
L15e
L13
L6
L7Ae
L24
L4e
L37e
L22
L14
L3
L2
L39e
L29
L37Ae
L31e
L24e
L23
L19e
Large subunit (Haloarcula marismortui)
Thx
S9
S10
S7
S14
S19
S13
S3
S2
S11
S18
S6
S4
S5
S8
S12
S15
S17
S16
S20
Small subunit (Thermus aquaticus)
Ribosomal RNAs
Bacteria / Archaea
Eukaryotes
23S
5S
16S
25S
5S
5.8S
18S
Ribosomal proteins
EF-Tu
S10
L3
L4
L23
L2
S19
L22
S3
RP-L16
L29
S20e
L3e
L4e
L23Ae
L8e
S15e
L17e
S3e
L35e
L10e
L7/L12
stalk
S17
L14
L24
L5
S14
S8
L6
L18
S5
L30
L15
SecY
S11e
L23e
L26e
S4e
L11e
S29e
S15Ae
L9e
L32e
L19e
L5e
S2e
L7e
L27Ae
IF1
L36
S13
S11
S4
RpoA
L17
L13
S9
L34e
L14e
S18e
S14e
S9e
L18e
L13Ae
S16e
EF-Tu,G
S7
S12
L7A
RpoC,B
S5e
S23e
L30e
L7Ae
L7/L12
L12
L10
L1
L11
LP1,LP2
LP0
L10Ae
L12e
S2
EF-Ts
IF2
S15
IF3
L35
L20
L34
RF1
L31
L32
L9
S18
S6
SAe
S13e
L28
L33
L21
L27
FtsY,Ffh
S16
L19
S1
S20
S21
L25
L10e
L13e
L15e
L21e
L24e
L31e
L35Ae
L37e
L37Ae
L39e
L40e
L41e
L44e
S3Ae
S6e
S8e
S17e
S19e
S24e
S25e
S26e
S27e
S27Ae
S28e
S30e
LX
L6e
L18Ae
L22e
L27e
L28e
L29e
L36e
L38e
S7e
S10e
S12e
S21e
03010 7/29/13
(c) Kanehisa Laboratories

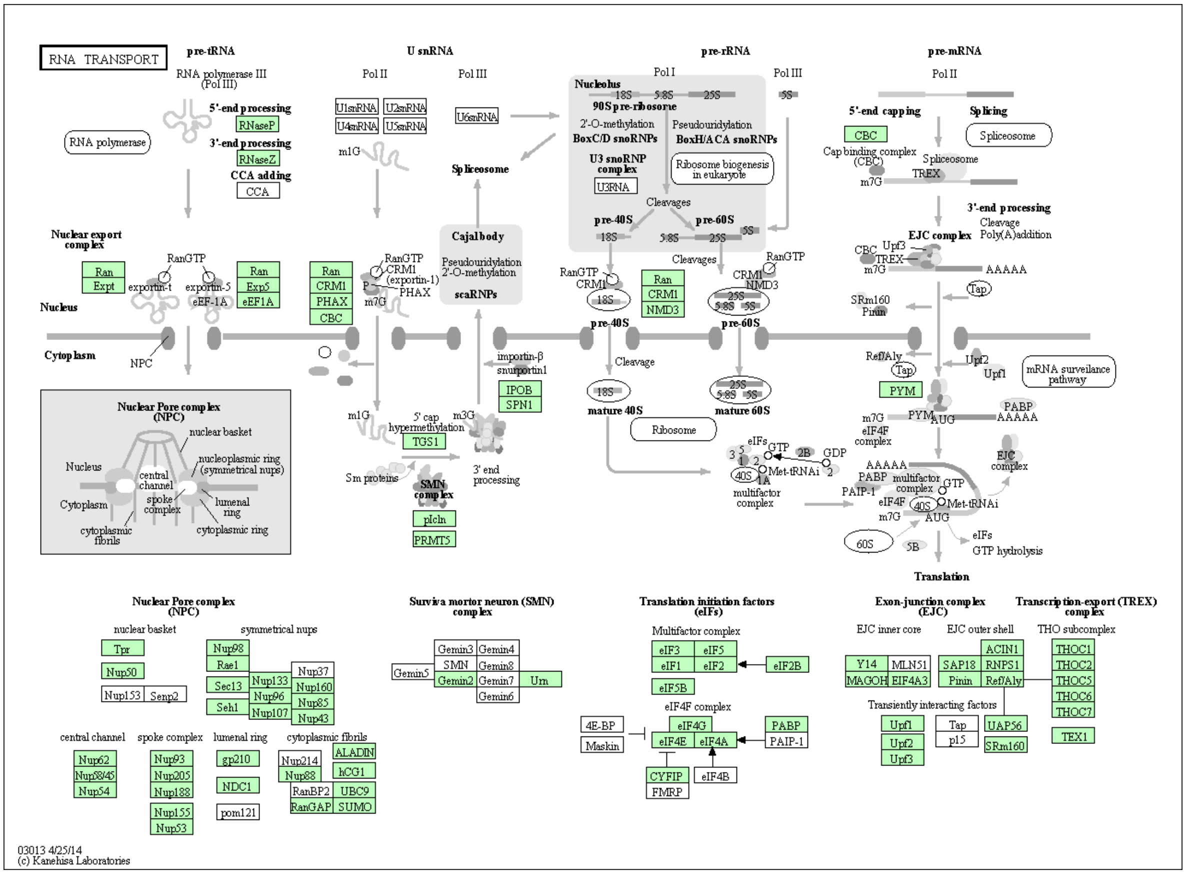
RNA TRANSPORT
pre-tRNA
U snRNA
pre-rRNA
pre-mRNA
Nucleolus
90S pre-ribosome
Spliceosome
Cajal body
scaRNPs
Nuclear export complex
Nucleus
Cytoplasm
NPC
Nuclear Pore complex (NPC)
pre-40S
pre-60S
mature 40S
mature 60S
Ribosome
EJC complex
mRNA surveilance pathway
Translation
Nuclear Pore complex (NPC)
Surviva mortor neuron (SMN) complex
Translation initiation factors (eIFs)
Exon-junction complex (EJC)
Transcription-export (TREX) complex
03013 4/25/14
(c) Kanehisa Laboratories

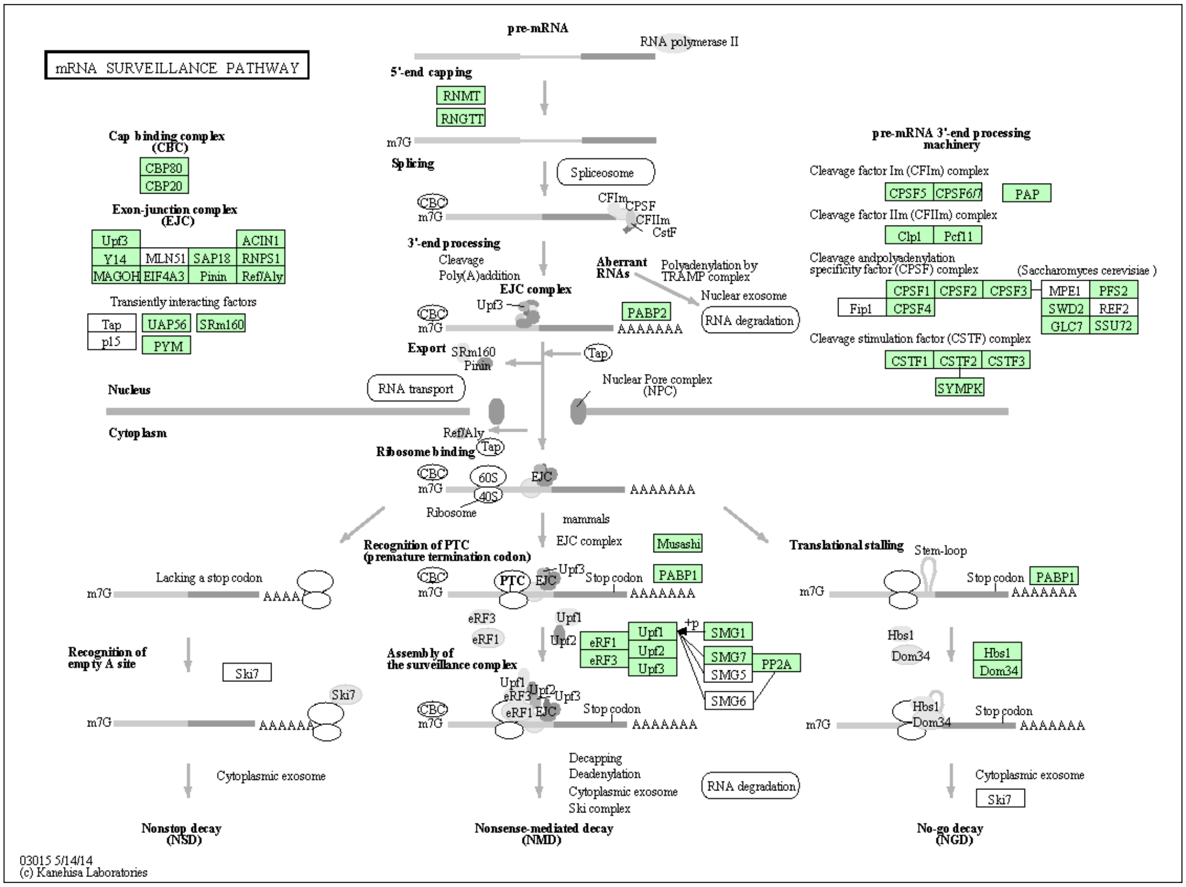
mRNA SURVEILLANCE PATHWAY
pre-mRNA
RNA polymerase II
5'-end capping
RNMT
RNGTT
m7G
Splicing
Spliceosome
CBC
CFIm
CPSF
CFIIm
CstF
3'-end processing
Cleavage
Poly(A)addition
EJC complex
Upf3
PABP2
AAAAAAA
Aberrant RNAs
Polyadenylation by TRAMP complex
Nuclear exosome
RNA degradation
Export
SRm160
Pinin
Tap
RNA transport
Nuclear Pore complex (NPC)
Nucleus
Cytoplasm
Ref/Aly
Ribosome binding
60S
40S
EJC
Ribosome
mammals
EJC complex
Musashi
Cap binding complex (CBC)
CBP80
CBP20
Exon-junction complex (EJC)
Upf3
ACIN1
Y14
MLN51
SAP18
RNPS1
MAGOH
EIF4A3
Pinin
Ref/Aly
Transiently interacting factors
Tap
UAP56
SRm160
p15
PYM
pre-mRNA 3'-end processing machinery
Cleavage factor Im (CFIm) complex
CPSF5
CPSF6/7
PAP
Cleavage factor IIm (CFIIm) complex
Clp1
Pcf11
Cleavage andpolyadenylation specificity factor (CPSF) complex
(Saccharomyces cerevisiae)
CPSF1
CPSF2
CPSF3
MPE1
PFS2
Fip1
CPSF4
SWD2
REF2
GLC7
SSU72
Cleavage stimulation factor (CSTF) complex
CSTF1
CSTF2
CSTF3
SYMPK
Lacking a stop codon
AAAA
Recognition of empty A site
Ski7
AAAAAA
Cytoplasmic exosome
Nonstop decay (NSD)
Recognition of PTC (premature termination codon)
PTC
Stop codon
PABP1
eRF3
eRF1
Upf1
Upf2
Upf3
SMG1
SMG7
SMG5
PP2A
SMG6
+p
Assembly of the surveillance complex
Decapping
Deadenylation
Cytoplasmic exosome
Ski complex
Nonsense-mediated decay (NMD)
Translational stalling
Stem-loop
Hbs1
Dom34
No-go decay (NGD)
03015 5/14/14
(c) Kanehisa Laboratories

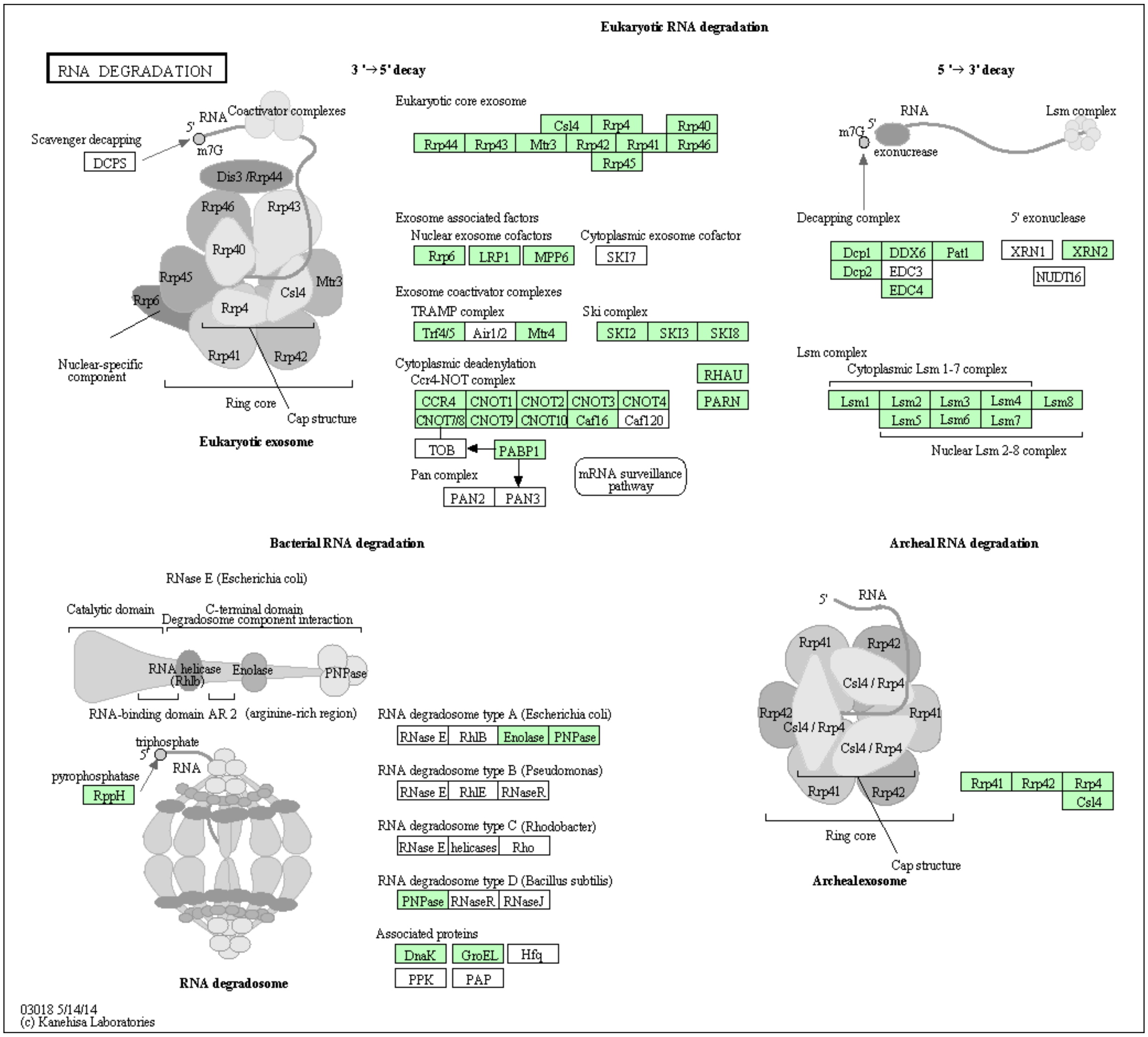

Eukaryotic RNA degradation
RNA DEGRADATION
3 '→ 5' decay
5 '→ 3' decay
Eukaryotic core exosome
Csl4
Rrp4
Rrp40
Rrp44
Rrp43
Mtr3
Rrp42
Rrp41
Rrp46
Rrp45
Scavenger decapping
DCPS
RNA
Coactivator complexes
m7G
Dis3 /Rrp44
Rrp46
Rrp43
Rrp40
Rrp45
Mtr3
Csl4
Rrp6
Rrp4
Rrp41
Rrp42
Nuclear-specific component
Ring core
Cap structure
Eukaryotic exosome
Exosome associated factors
Nuclear exosome cofactors
Rrp6
LRP1
MPP6
Cytoplasmic exosome cofactor
SKI7
Exosome coactivator complexes
TRAMP complex
Trf4/5
Air1/2
Mtr4
Ski complex
SKI2
SKI3
SKI8
Cytoplasmic deadenylation
Ccr4-NOT complex
CCR4
CNOT1
CNOT2
CNOT3
CNOT4
CNOT7/8
CNOT9
CNOT10
Caf16
Caf120
RHAU
PARN
TOB
PABP1
Pan complex
PAN2
PAN3
mRNA surveillance pathway
RNA
Lsm complex
m7G
exonucrease
Decapping complex
5' exonuclease
Dcp1
DDX6
Pat1
Dcp2
EDC3
EDC4
XRN1
XRN2
NUDT16
Lsm complex
Cytoplasmic Lsm 1-7 complex
Lsm1
Lsm2
Lsm3
Lsm4
Lsm8
Lsm5
Lsm6
Lsm7
Nuclear Lsm 2-8 complex
Bacterial RNA degradation
RNase E (Escherichia coli)
Catalytic domain
C-terminal domain
Degradosome component interaction
RNA helicase (Rhlb)
Enolase
PNPase
RNA-binding domain AR 2 (arginine-rich region)
triphosphate
pyrophosphatase
RppH
RNA
RNA degradosome
RNA degradosome type A (Escherichia coli)
RNase E
RhlB
Enolase
PNPase
RNA degradosome type B (Pseudomonas)
RNase E
RhlE
RNaseR
RNA degradosome type C (Rhodobacter)
RNase E
helicases
Rho
RNA degradosome type D (Bacillus subtilis)
PNPase
RNaseR
RNaseJ
Associated proteins
DnaK
GroEL
Hfq
PPK
PAP
Archeal RNA degradation
RNA
Rrp41
Rrp42
Csl4 / Rrp4
Rrp42
Rrp41
Csl4 / Rrp4
Csl4 / Rrp4
Rrp41
Rrp42
Ring core
Cap structure
Archealexosome
Rrp41
Rrp42
Rrp4
Csl4
03018 5/14/14
(c) Kanehisa Laboratories

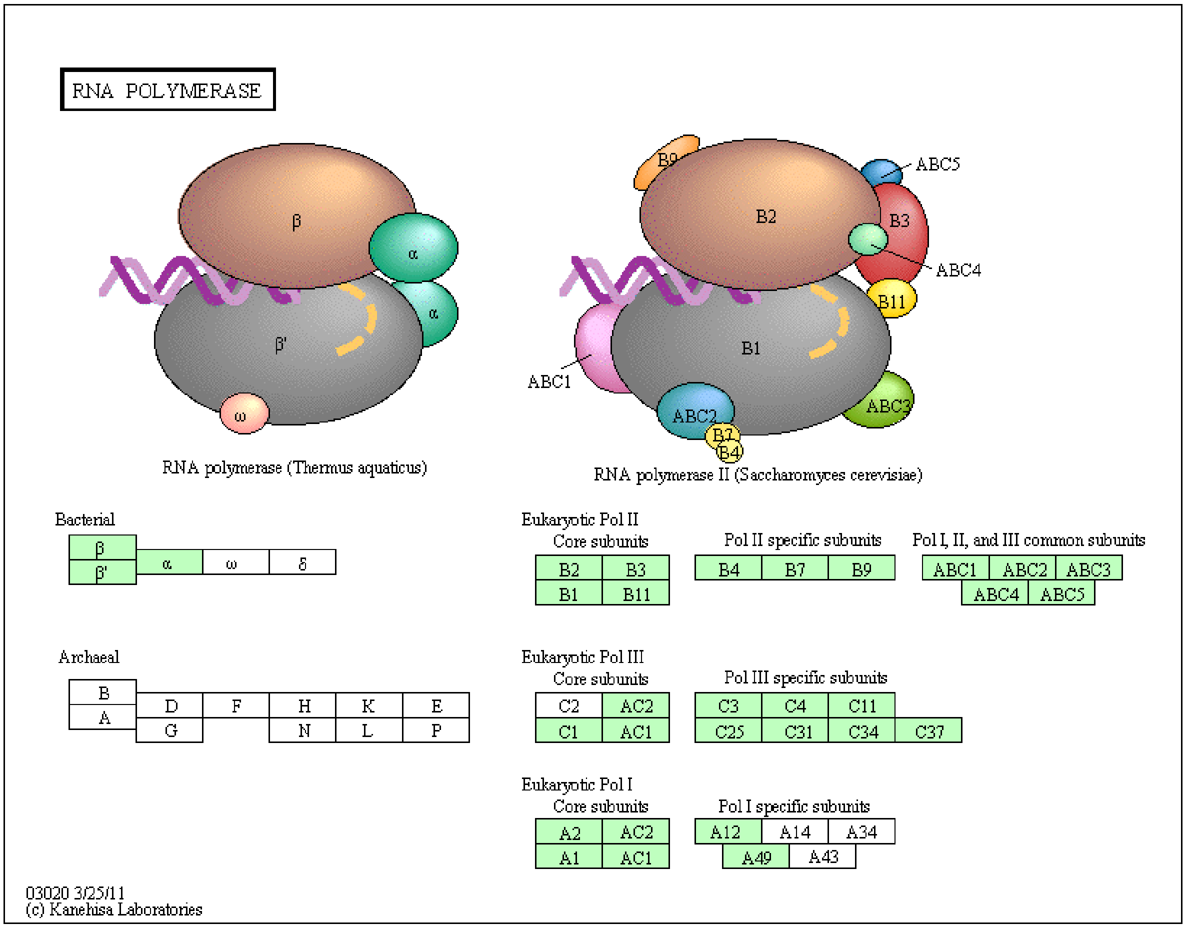
RNA POLYMERASE
β
α
α
β'
ω
RNA polymerase (Thermus aquaticus)
B9
ABC5
B2
B3
ABC4
B11
B1
ABC1
ABC2
ABC3
B7
B4
RNA polymerase II (Saccharomyces cerevisiae)
Bacterial
β
β'
α
ω
δ
Archaeal
B
A
D
F
H
K
E
G
N
L
P
Eukaryotic Pol II
Core subunits
B2
B3
B1
B11
Pol II specific subunits
B4
B7
B9
Pol I, II, and III common subunits
ABC1
ABC2
ABC3
ABC4
ABC5
Eukaryotic Pol III
Core subunits
C2
AC2
C1
AC1
Pol III specific subunits
C3
C4
C11
C25
C31
C34
C37
Eukaryotic Pol I
Core subunits
A2
AC2
A1
AC1
Pol I specific subunits
A12
A14
A34
A49
A43
03020 3/25/11
(c) Kanehisa Laboratories

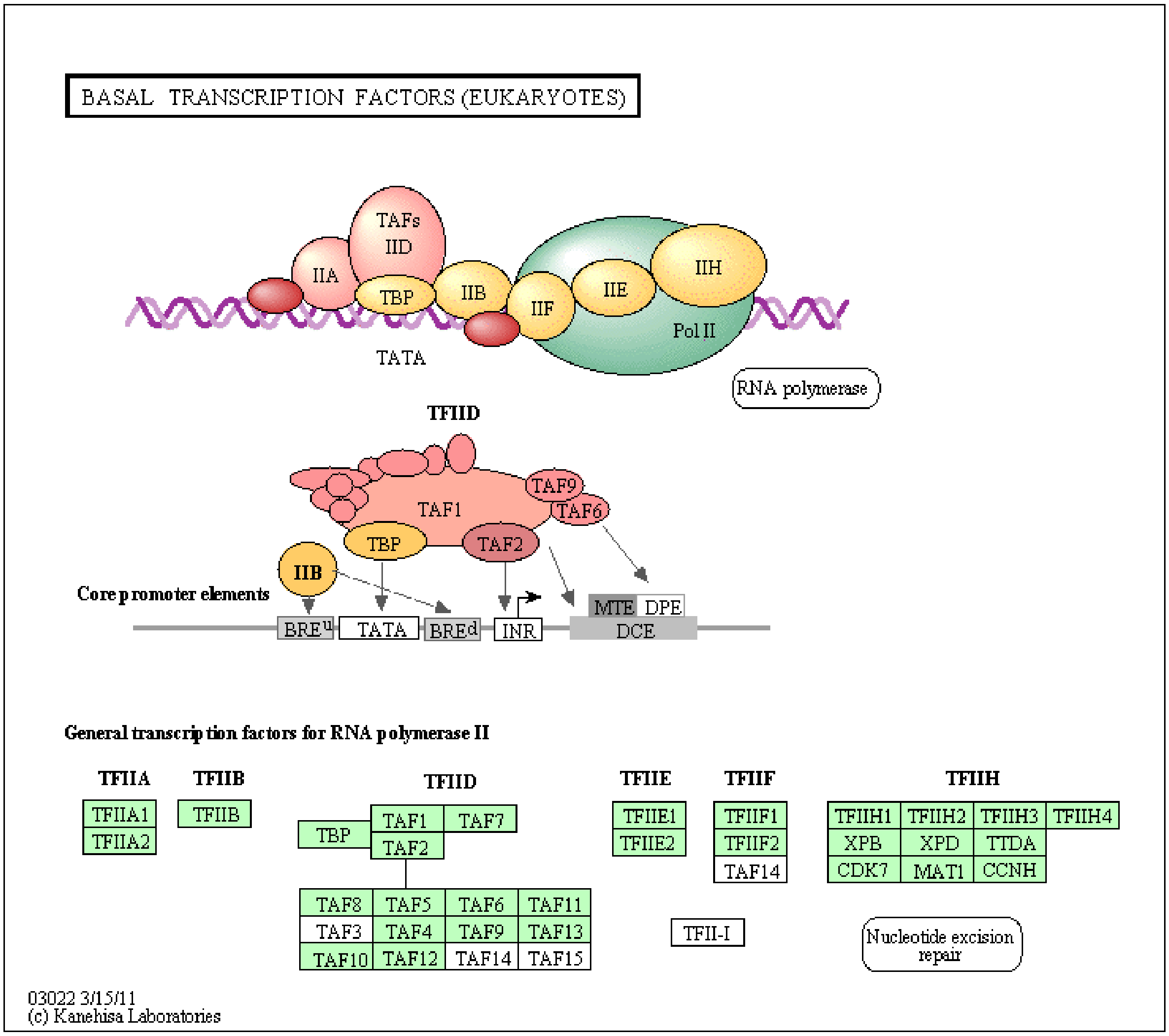
BASAL TRANSCRIPTION FACTORS (EUKARYOTES)
TAFs
IID
IIA
TBP
IIB
IIF
IIE
IIH
Pol II
TATA
RNA polymerase
TFIID
TAF9
TAF1
TAF6
TBP
TAF2
IIB
Core promoter elements
BREu
TATA
BREd
INR
MTE
DPE
DCE
General transcription factors for RNA polymerase II
TFIIA
TFIIA1
TFIIA2
TFIIB
TFIIB
TFIID
TBP
TAF1
TAF7
TAF2
TAF8
TAF5
TAF6
TAF11
TAF3
TAF4
TAF9
TAF13
TAF10
TAF12
TAF14
TAF15
TFIIE
TFIIE1
TFIIE2
TFIIF
TFIIF1
TFIIF2
TAF14
TFIIH
TFIIH1
TFIIH2
TFIIH3
TFIIH4
XPB
XPD
TTDA
CDK7
MAT1
CCNH
TFII-I
Nucleotide excision repair
03022 3/15/11
(c) Kanehisa Laboratories

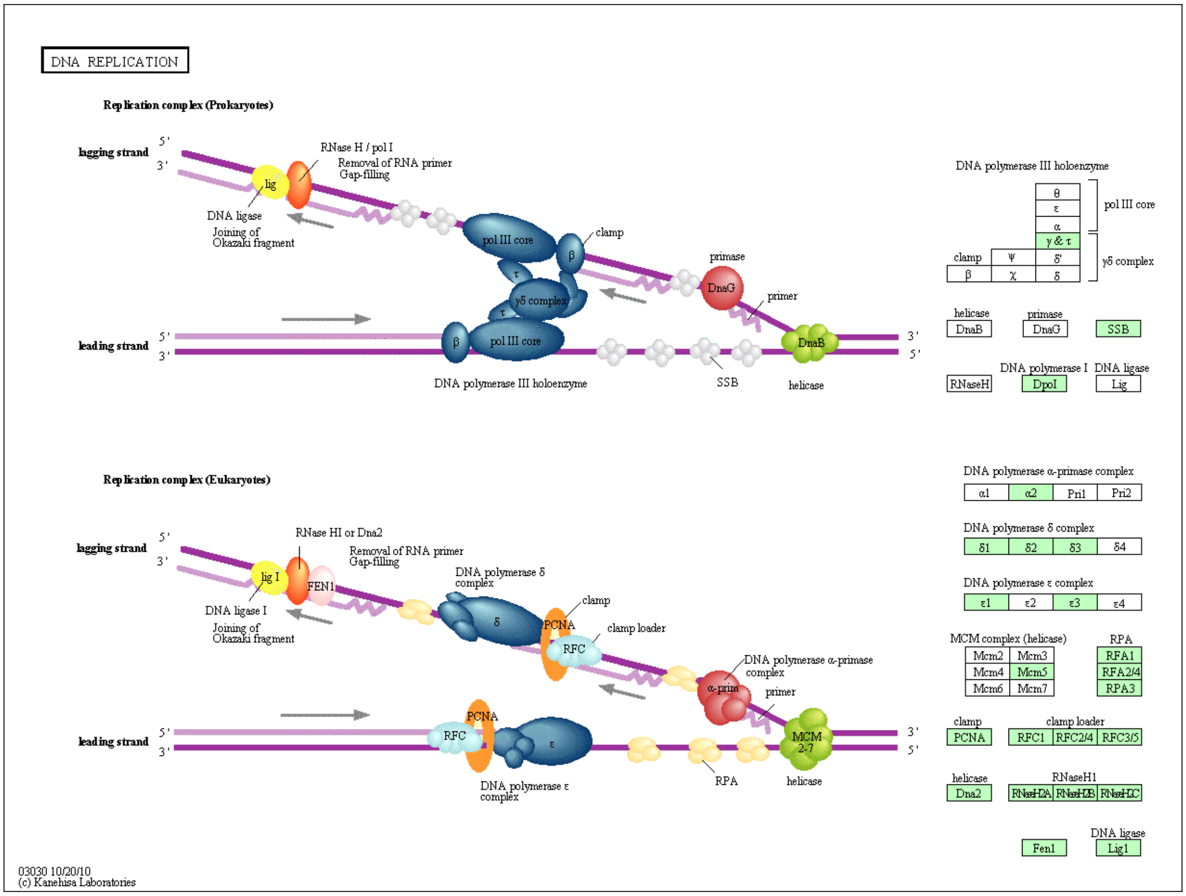
DNA REPLICATION
Replication complex (Prokaryotes)
lagging strand
leading strand
5'
3'
RNase H / pol I
Removal of RNA primer
Gap-filling
lig
DNA ligase
Joining of
Okazaki fragment
pol III core
clamp
β
τ
γδ complex
primase
DnaG
primer
DnaB
DNA polymerase III holoenzyme
SSB
helicase
θ
ε
α
γ & τ
ψ
δ'
χ
δ
pol III core
γδ complex
helicase
primase
DnaB
DnaG
SSB
DNA polymerase I
DNA ligase
RNaseH
DpoI
Lig
Replication complex (Eukaryotes)
RNase HI or Dna2
Removal of RNA primer
Gap-filling
lig I
FEN1
DNA ligase I
DNA polymerase δ
complex
PCNA
RFC
clamp loader
DNA polymerase α-primase
complex
α-prim
MCM
2-7
DNA polymerase ε
complex
RPA
DNA polymerase α-primase complex
α1
α2
Pri1
Pri2
DNA polymerase δ complex
δ1
δ2
δ3
δ4
DNA polymerase ε complex
ε1
ε2
ε3
ε4
MCM complex (helicase)
Mcm2
Mcm3
Mcm4
Mcm5
Mcm6
Mcm7
RFA1
RFA2/4
RFA3
RFC1
RFC2/4
RFC3/5
Dna2
RNaseH1
RNaseH2A
RNaseH2B
RNaseH2C
Fen1
Lig1
03030 10/20/10
(c) Kanehisa Laboratories

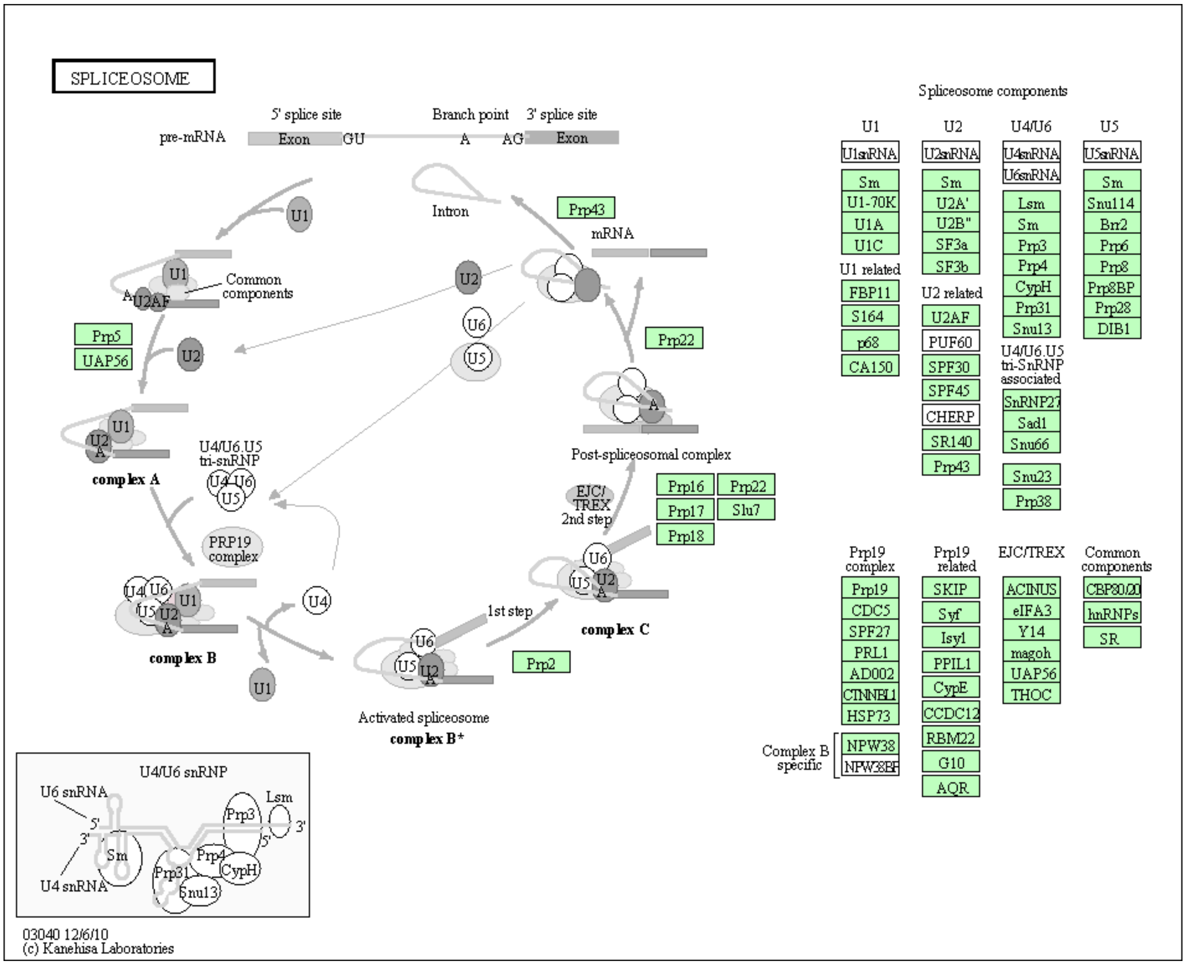
SPLICEOSOME
pre-mRNA
5' splice site
Branch point
3' splice site
Exon
GU
A
AG
Exon
Intron
mRNA
Prp43
U1
U2
Common components
U2AF
Prp5
UAP56
complex A
U4/U6.U5 tri-snRNP
PRP19 complex
complex B
U4
Activated spliceosome
complex B*
1st step
Prp2
complex C
EJC/ TREX
2nd step
Prp16
Prp22
Prp17
Slu7
Prp18
Post-spliceosomal complex
Prp22
U6
U5
U4/U6 snRNP
U6 snRNA
U4 snRNA
Sm
Lsm
Prp3
Prp4
Prp31
CypH
Snu13
Spliceosome components
U1
U1snRNA
Sm
U1-70K
U1A
U1C
U1 related
FBP11
S164
p68
CA150
U2
U2snRNA
Sm
U2A'
U2B"
SF3a
SF3b
U2 related
U2AF
PUF60
SPF30
SPF45
CHERP
SR140
Prp43
U4/U6
U4snRNA
U6snRNA
Lsm
Sm
Prp3
Prp4
CypH
Prp31
Snu13
U4/U6.U5 tri-SnRNP associated
SnRNP27
Sad1
Snu66
Snu23
Prp38
U5
U5snRNA
Sm
Snu114
Brr2
Prp6
Prp8
Prp8BP
Prp28
DIB1
Prp19 complex
Prp19
CDC5
SPF27
PRL1
AD002
CTNNBL1
HSP73
Complex B specific
NPW38
NPW38BP
Prp19 related
SKIP
Syf
Isy1
PPIL1
CypE
CCDC12
RBM22
G10
AQR
EJC/TREX
ACINUS
eIFA3
Y14
magoh
UAP56
THOC
Common components
CBP80/20
hnRNPs
SR
03040 12/6/10
(c) Kanehisa Laboratories

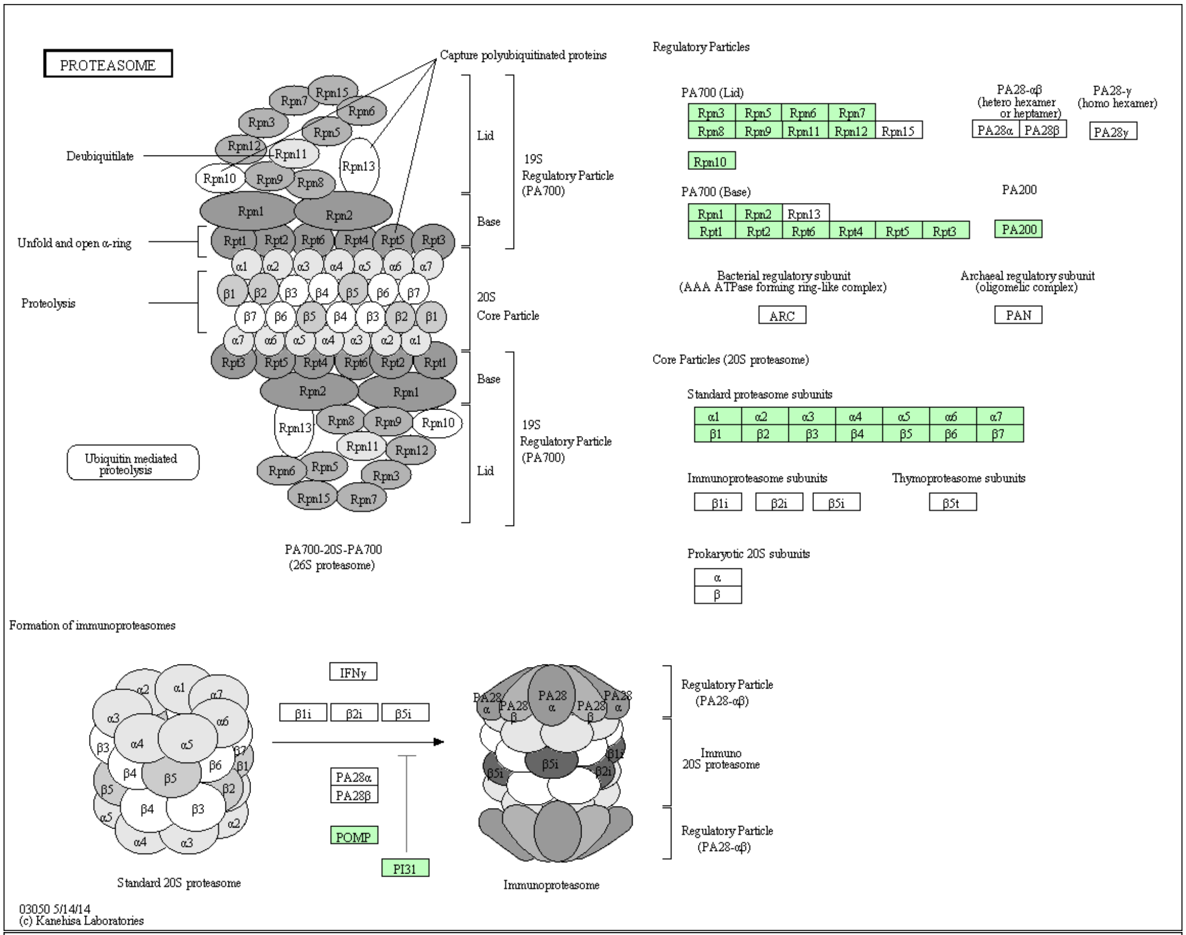
PROTEASOME
Capture polyubiquitinated proteins
Regulatory Particles
Deubiquitilate
Unfold and open α-ring
Proteolysis
Lid
Base
19S
Regulatory Particle
(PA700)
20S
Core Particle
Ubiquitin mediated proteolysis
PA700-20S-PA700
(26S proteasome)
PA700 (Lid)
Rpn3 Rpn5 Rpn6 Rpn7
Rpn8 Rpn9 Rpn11 Rpn12 Rpn15
Rpn10
PA700 (Base)
Rpn1 Rpn2 Rpn13
Rpt1 Rpt2 Rpt6 Rpt4 Rpt5 Rpt3
PA28-αβ
(hetero hexamer or heptamer)
PA28α PA28β
PA28-γ
(homo hexamer)
PA28γ
PA200
Bacterial regulatory subunit
(AAA ATPase forming ring-like complex)
ARC
Archaeal regulatory subunit
(oligomelic complex)
PAN
Core Particles (20S proteasome)
Standard proteasome subunits
α1 α2 α3 α4 α5 α6 α7
β1 β2 β3 β4 β5 β6 β7
Immunoproteasome subunits
β1i β2i β5i
Thymoproteasome subunits
β5t
Prokaryotic 20S subunits
α
β
Formation of immunoproteasomes
IFNγ
β1i β2i β5i
PA28α
PA28β
POMP
PI31
Standard 20S proteasome
Immunoproteasome
Regulatory Particle
(PA28-αβ)
Immuno
20S proteasome
03050 5/14/14
(c) Kanehisa Laboratories

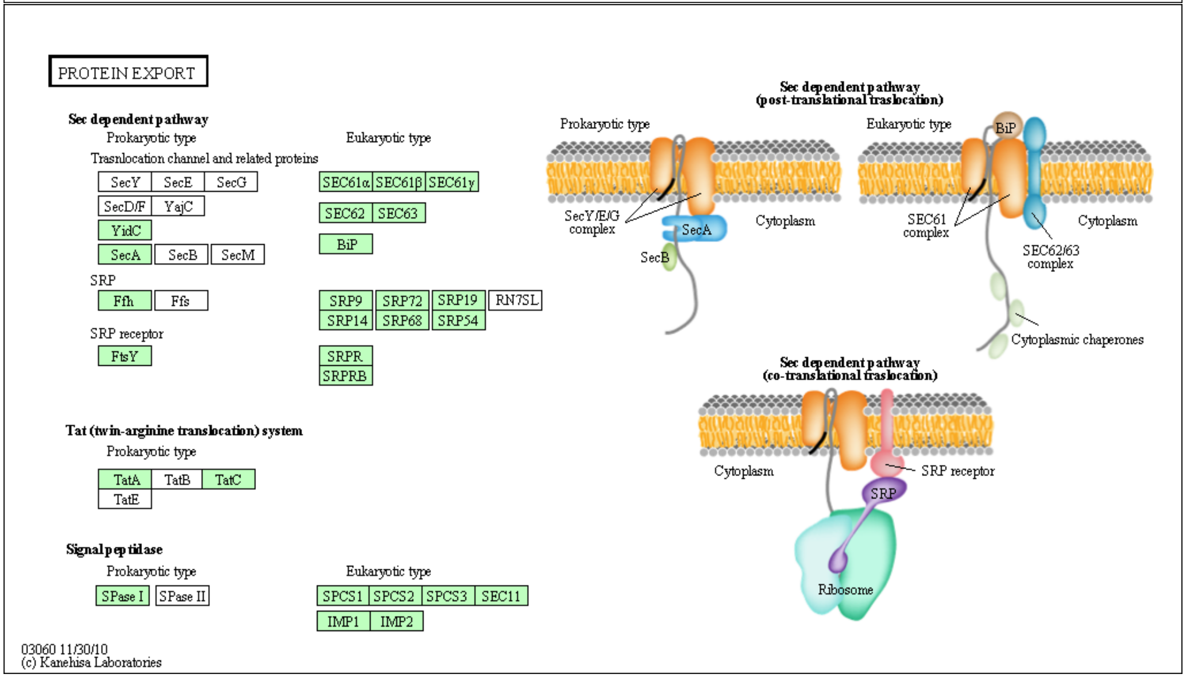
PROTEIN EXPORT
Sec dependent pathway
Prokaryotic type
Trasnlocation channel and related proteins
SecY SecE SecG
SecD/F YajC
YidC
SecA SecB SecM
SRP
Ffh Ffs
SRP receptor
FtsY
Eukaryotic type
SEC61α SEC61β SEC61γ
SEC62 SEC63
BiP
SRP9 SRP72 SRP19 RN7SL
SRP14 SRP68 SRP54
SRPR
SRPRB
Tat (twin-arginine translocation) system
Prokaryotic type
TatA TatB TatC
TatE
Signal peptidase
Prokaryotic type
SPase I SPase II
Eukaryotic type
SPCS1 SPCS2 SPCS3 SEC11
IMP1 IMP2
Sec dependent pathway
(post-translational traslocation)
Prokaryotic type
SecY/E/G complex
SecA
SecB
Cytoplasm
Eukaryotic type
BiP
SEC61 complex
SEC62/63 complex
Cytoplasmic chaperones
Sec dependent pathway
(co-translational traslocation)
SRP receptor
SRP
Ribosome
03060 11/30/10
(c) Kanehisa Laboratories

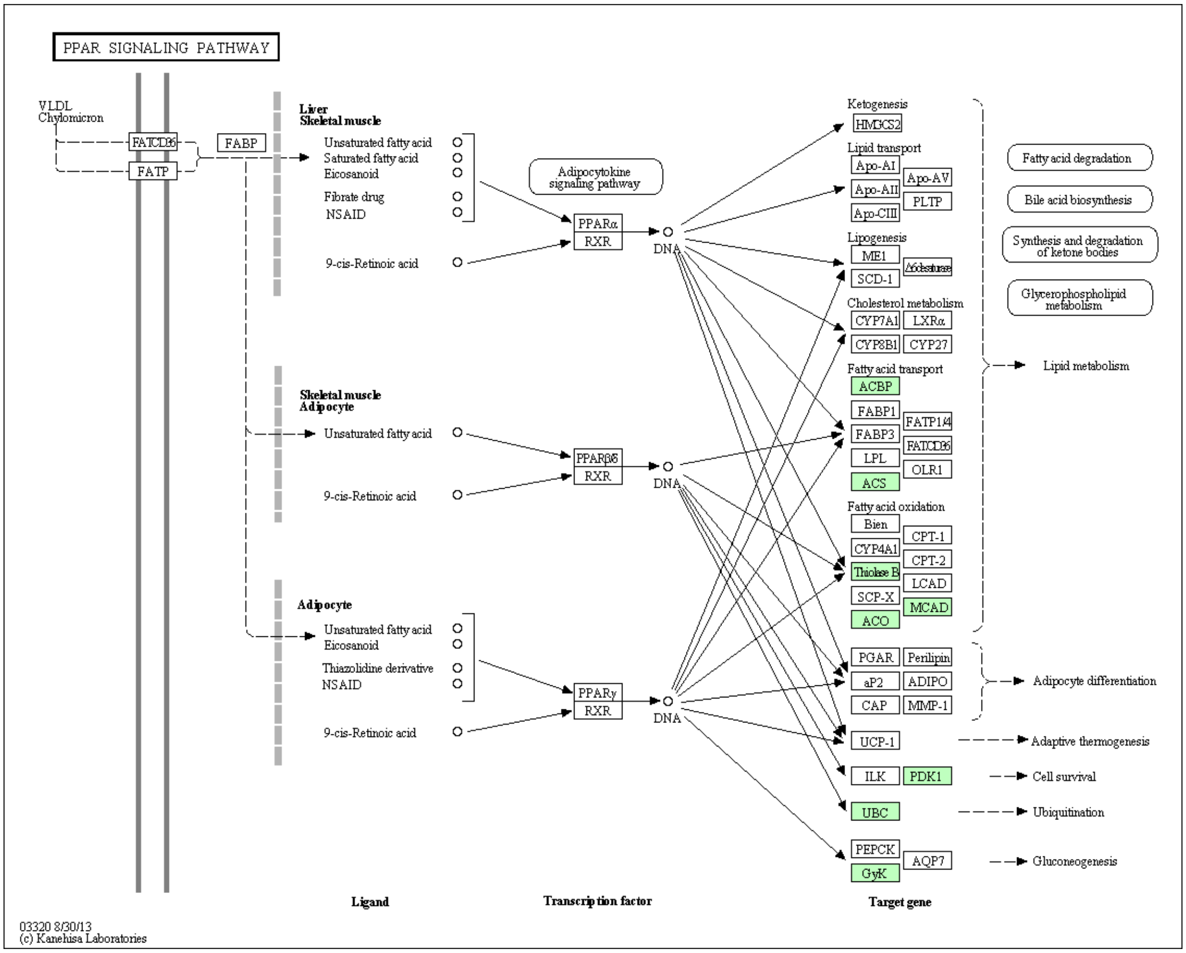
PPAR SIGNALING PATHWAY
VLDL
Chylomicron
FATCD36
FATP
FABP
Liver
Skeletal muscle
Unsaturated fatty acid
Saturated fatty acid
Eicosanoid
Fibrate drug
NSAID
9-cis-Retinoic acid
Adipocytokine signaling pathway
PPARα
RXR
DNA
Skeletal muscle
Adipocyte
Unsaturated fatty acid
9-cis-Retinoic acid
PPARβ/δ
RXR
DNA
Adipocyte
Unsaturated fatty acid
Eicosanoid
Thiazolidine derivative
NSAID
9-cis-Retinoic acid
PPARγ
RXR
DNA
Ketogenesis
HMGCS2
Lipid transport
Apo-AI
Apo-AII
Apo-AV
Apo-CIII
PLTP
Lipogenesis
ME1
SCD-1
Δ6-desaturase
Cholesterol metabolism
CYP7A1
LXRα
CYP8B1
CYP27
Fatty acid transport
ACBP
FABP1
FATP1/4
FABP3
FATCD36
LPL
OLR1
ACS
Fatty acid oxidation
Bien
CPT-1
CYP4A1
CPT-2
Thiolase B
LCAD
SCP-X
MCAD
ACO
PGAR
Perilipin
aP2
ADIPO
CAP
MMP-1
UCP-1
ILK
PDK1
UBC
PEPCK
AQP7
GyK
Fatty acid degradation
Bile acid biosynthesis
Synthesis and degradation of ketone bodies
Glycerophospholipid metabolism
Lipid metabolism
Adipocyte differentiation
Adaptive thermogenesis
Cell survival
Ubiquitination
Gluconeogenesis
Ligand
Transcription factor
Target gene
03320 8/30/13
(c) Kanehisa Laboratories

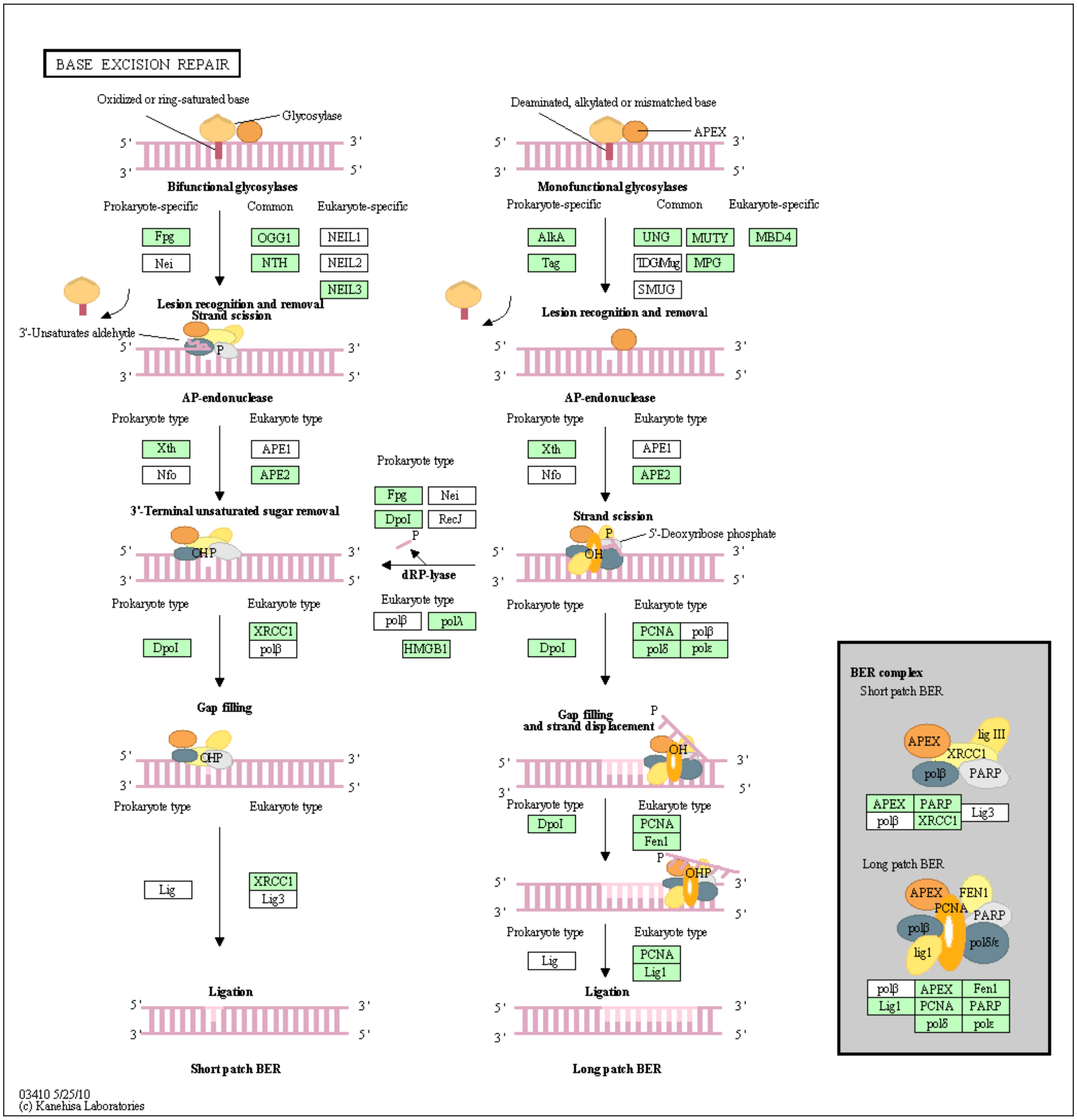
BASE EXCISION REPAIR
Oxidized or ring-saturated base
Glycosylase
Bifunctional glycosylases
Prokaryote-specific
Common
Eukaryote-specific
Fpg
Nei
OGG1
NTH
NEIL1
NEIL2
NEIL3
Lesion recognition and removal
Strand scission
3'-Unsaturates aldehyde
AP-endonuclease
Prokaryote type
Eukaryote type
Xth
Nfo
APE1
APE2
3'-Terminal unsaturated sugar removal
OHP
DpoI
XRCC1
polβ
Gap filling
Lig
Lig3
Ligation
Short patch BER
Deaminated, alkylated or mismatched base
APEX
Monofunctional glycosylases
AlkA
Tag
UNG
MUTY
MBD4
TDG/Mug
MPG
SMUG
Strand scission
5'-Deoxyribose phosphate
OH
dRP-lyase
RecJ
polλ
HMGB1
PCNA
polδ
polε
Gap filling
and strand displacement
Fen1
Lig1
Long patch BER
BER complex
Short patch BER
lig III
PARP
Long patch BER
FEN1
lig1
polδ/ε
03410 5/25/10
(c) Kanehisa Laboratories

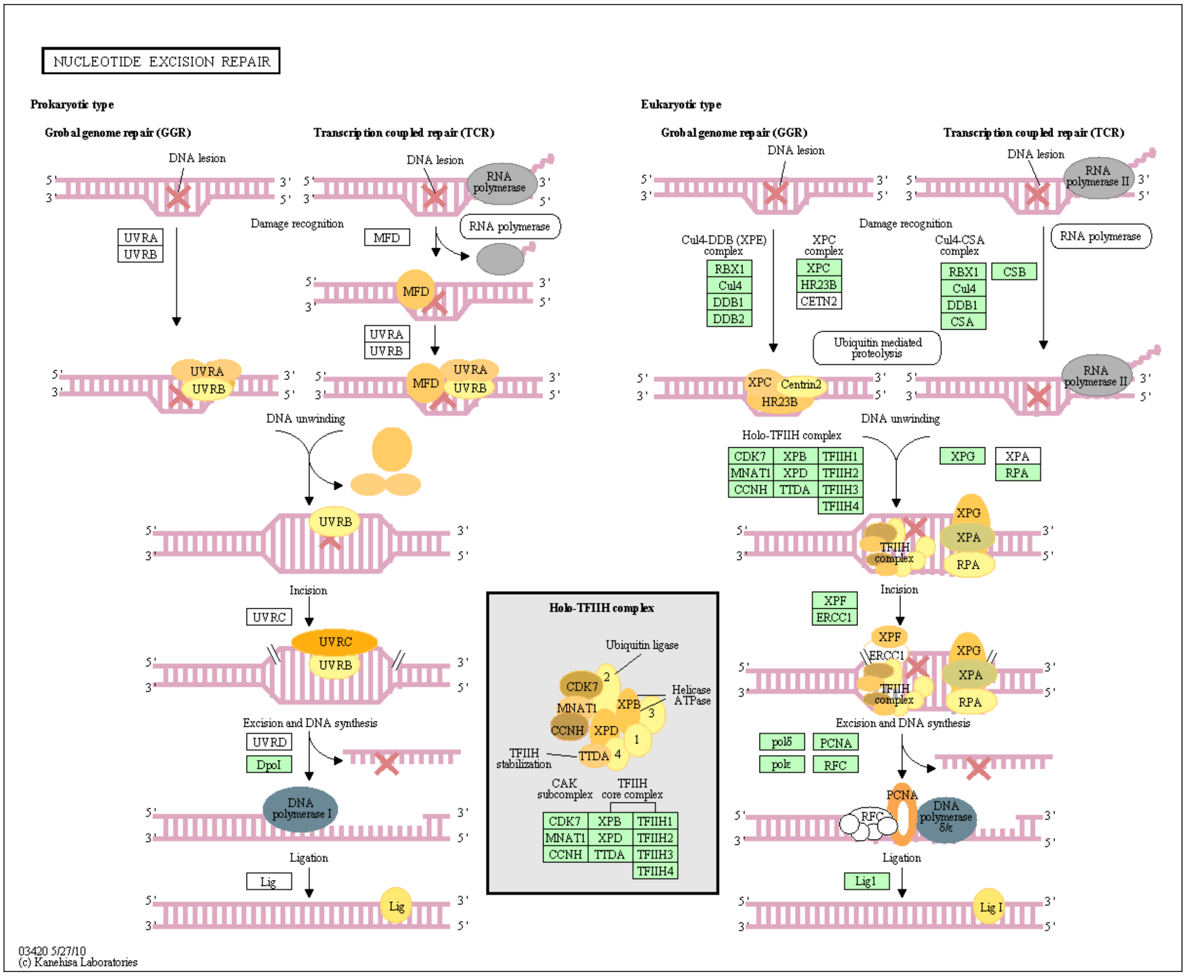
NUCLEOTIDE EXCISION REPAIR
Prokaryotic type
Grobal genome repair (GGR)
Transcription coupled repair (TCR)
Eukaryotic type
DNA lesion
RNA polymerase
RNA polymerase II
Damage recognition
UVRA
UVRB
MFD
DNA unwinding
Incision
UVRC
Excision and DNA synthesis
UVRD
DpoI
DNA polymerase I
Ligation
Lig
Cul4-DDB (XPE) complex
RBX1
Cul4
DDB1
DDB2
XPC complex
XPC
HR23B
CETN2
Cul4-CSA complex
CSA
CSB
Ubiquitin mediated proteolysis
Centrin2
Holo-TFIIH complex
CDK7
XPB
TFIIH1
MNAT1
XPD
TFIIH2
CCNH
TTDA
TFIIH3
TFIIH4
XPG
XPA
RPA
TFIIH complex
XPF
ERCC1
polδ
polε
PCNA
RFC
DNA polymerase δ/ε
Lig1
Lig I
Ubiquitin ligase
Helicase
ATPase
TFIIH stabilization
CAK subcomplex
TFIIH core complex
03420 5/27/10
(c) Kanehisa Laboratories

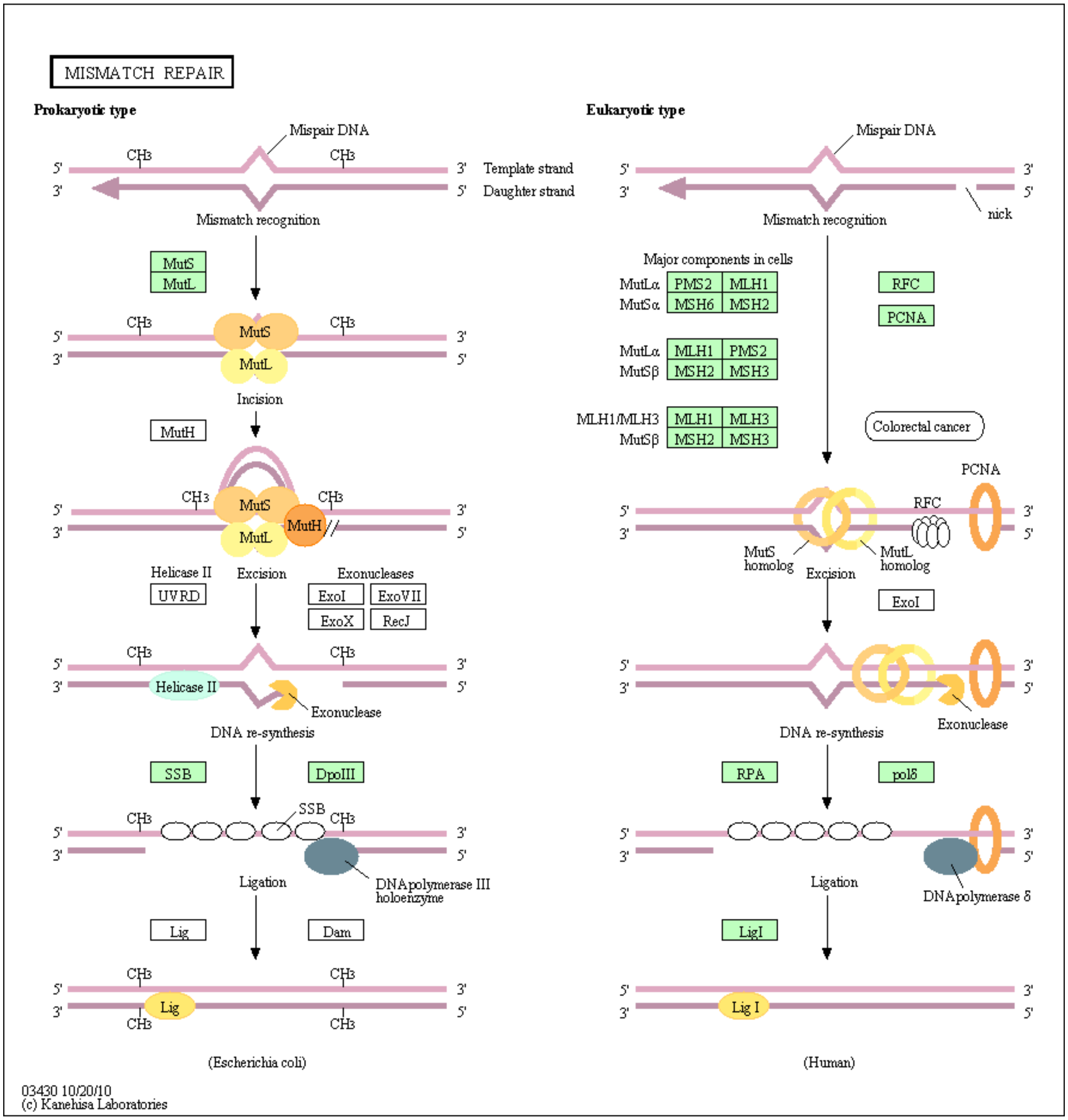
MISMATCH REPAIR
Prokaryotic type
Mispair DNA
CH3
5'
3'
Template strand
Daughter strand
Mismatch recognition
MutS
MutL
Incision
MutH
Helicase II
UVRD
Excision
Exonucleases
ExoI
ExoVII
ExoX
RecJ
Exonuclease
DNA re-synthesis
SSB
DpoIII
DNApolymerase III holoenzyme
Ligation
Lig
Dam
(Escherichia coli)
Eukaryotic type
nick
Major components in cells
MutLα
MutSα
MutSβ
MLH1/MLH3
PMS2
MLH1
MSH6
MSH2
MSH3
MLH3
RFC
PCNA
Colorectal cancer
MutS homolog
MutL homolog
Exonuclease
RPA
polδ
DNApolymerase δ
LigI
Lig I
(Human)
03430 10/20/10
(c) Kanehisa Laboratories

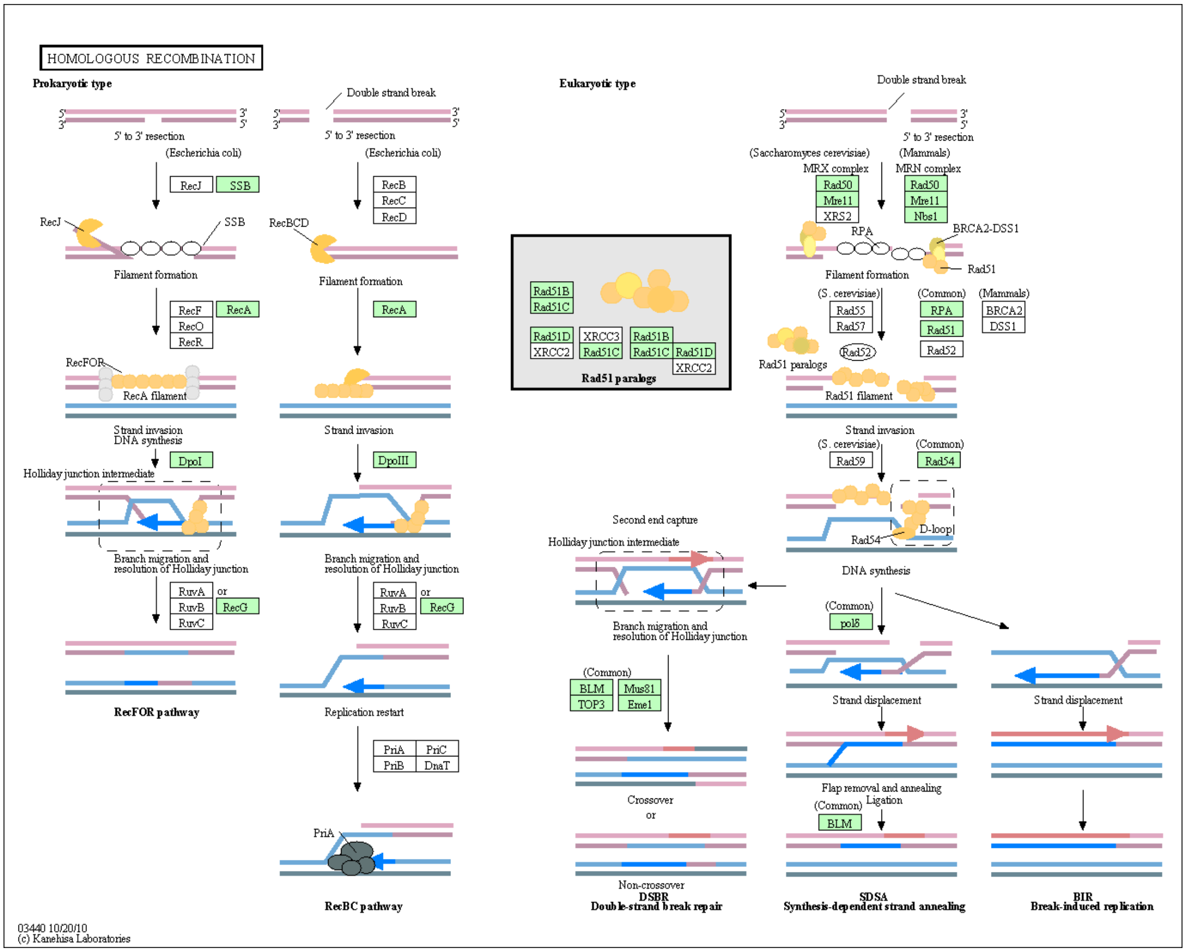
HOMOLOGOUS RECOMBINATION
Prokaryotic type
5' to 3' resection
(Escherichia coli)
RecJ
SSB
Filament formation
RecF
RecO
RecR
RecA
RecFOR
RecA filament
Strand invasion
DNA synthesis
DpoI
Holliday junction intermediate
Branch migration and resolution of Holliday junction
RuvA
RuvB
RuvC
or
RecG
RecFOR pathway
Double strand break
RecB
RecC
RecD
RecBCD
DpoIII
Replication restart
PriA
PriB
PriC
DnaT
RecBC pathway
Rad51B
Rad51C
Rad51D
XRCC2
XRCC3
Rad51 paralogs
Eukaryotic type
(Saccharomyces cerevisiae)
(Mammals)
MRX complex
MRN complex
Rad50
Mre11
XRS2
Nbs1
RPA
BRCA2-DSS1
Rad51
(S. cerevisiae)
(Common)
Rad55
Rad57
Rad52
BRCA2
DSS1
Rad51 paralogs
Rad51 filament
Rad59
Rad54
D-loop
DNA synthesis
Second end capture
BLM
TOP3
Mus81
Eme1
Crossover
or
Non-crossover
DSBR
Double-strand break repair
polδ
Strand displacement
Flap removal and annealing
Ligation
SDSA
Synthesis-dependent strand annealing
BIR
Break-induced replication
03440 10/20/10
(c) Kanehisa Laboratories

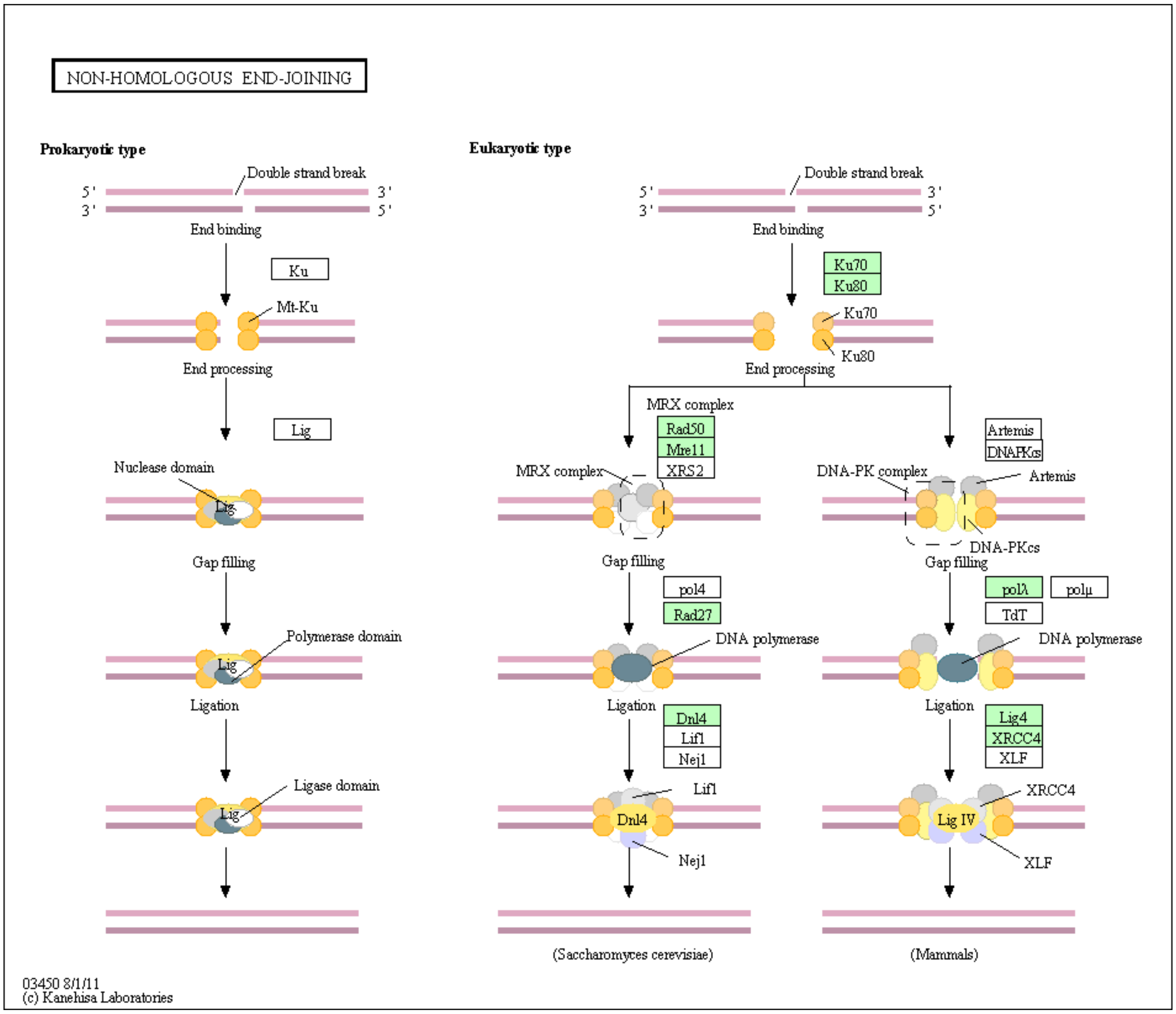
NON-HOMOLOGOUS END-JOINING
Prokaryotic type
Double strand break
5'
3'
3'
5'
End binding
Ku
Mt-Ku
End processing
Lig
Nuclease domain
Lig
Gap filling
Polymerase domain
Lig
Ligation
Ligase domain
Lig
Eukaryotic type
Double strand break
5'
3'
3'
5'
End binding
Ku70
Ku80
Ku70
Ku80
End processing
MRX complex
Rad50
Mre11
XRS2
MRX complex
Artemis
DNAPKcs
DNA-PK complex
Artemis
DNA-PKcs
Gap filling
pol4
Rad27
DNA polymerase
Gap filling
polλ
polμ
TdT
DNA polymerase
Ligation
Dnl4
Lif1
Nej1
Lif1
Dnl4
Nej1
Ligation
Lig4
XRCC4
XLF
XRCC4
Lig IV
XLF
(Saccharomyces cerevisiae)
(Mammals)
03450 8/1/11
(c) Kanehisa Laboratories

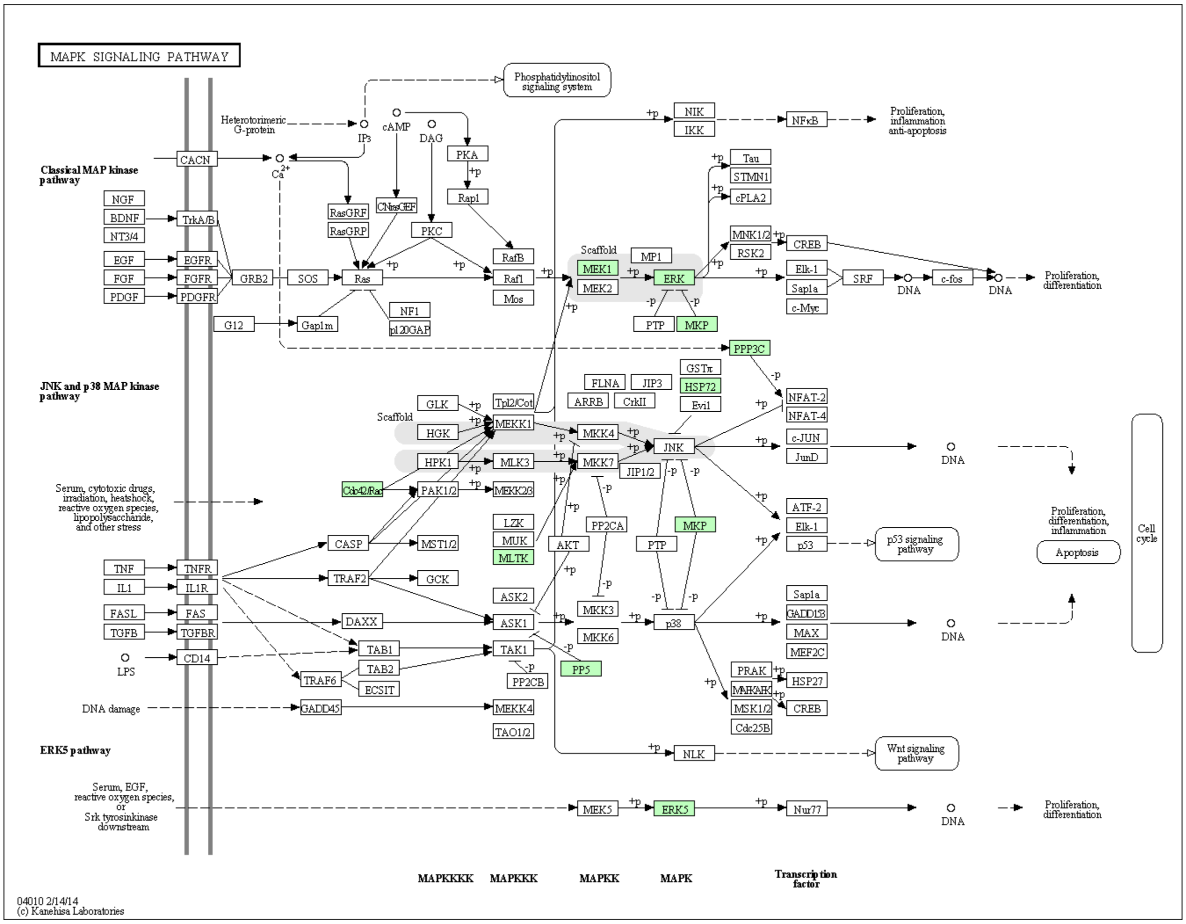
MAPK SIGNALING PATHWAY
Classical MAP kinase pathway
JNK and p38 MAP kinase pathway
ERK5 pathway
Heterotorimeric G-protein
IP3
cAMP
DAG
Ca2+
Phosphatidylinositol signaling system
CACN
NGF
BDNF
NT3/4
TrkA/B
EGF
FGF
PDGF
EGFR
FGFR
PDGFR
GRB2
SOS
Ras
G12
Gap1m
NF1
p120GAP
RasGRF
RasGRP
CNrasGEF
PKC
PKA
Rap1
RafB
Raf1
Mos
Scaffold
MP1
MEK1
MEK2
ERK
PTP
MKP
NIK
IKK
NFκB
Proliferation, inflammation anti-apoptosis
Tau
STMN1
cPLA2
MNK1/2
RSK2
CREB
Elk-1
Sap1a
c-Myc
SRF
DNA
c-fos
Proliferation, differentiation
PPP3C
NFAT-2
NFAT-4
c-JUN
JunD
GSTπ
HSP72
Evi1
FLNA
JIP3
ARRB
CrkII
GLK
HGK
HPK1
Tpl2/Cot
MEKK1
MLK3
MEKK2/3
MKK4
MKK7
JIP1/2
JNK
Cdc42/Rac
PAK1/2
LZK
MUK
MLTK
AKT
PP2CA
Serum, cytotoxic drugs, irradiation, heatshock, reactive oxygen species, lipopolysaccharide, and other stress
TNF
IL1
FASL
TGFB
LPS
TNFR
IL1R
FAS
TGFBR
CD14
CASP
MST1/2
TRAF2
GCK
DAXX
TAB1
TAB2
ECSIT
TRAF6
ASK2
ASK1
TAK1
PP2CB
PP5
MKK3
MKK6
p38
ATF-2
Elk-1
p53
p53 signaling pathway
Sap1a
GADD153
MAX
MEF2C
PRAK
MAPKAPK
MSK1/2
Cdc25B
HSP27
CREB
DNA damage
GADD45
MEKK4
TAO1/2
NLK
Wnt signaling pathway
Proliferation, differentiation, inflammation
Apoptosis
Cell cycle
Serum, EGF, reactive oxygen species, or Srk tyrosinkinase downstream
MEK5
ERK5
Nur77
MAPKKKK
MAPKKK
MAPKK
MAPK
Transcription factor
+p
-p
04010 2/14/14
(c) Kanehisa Laboratories

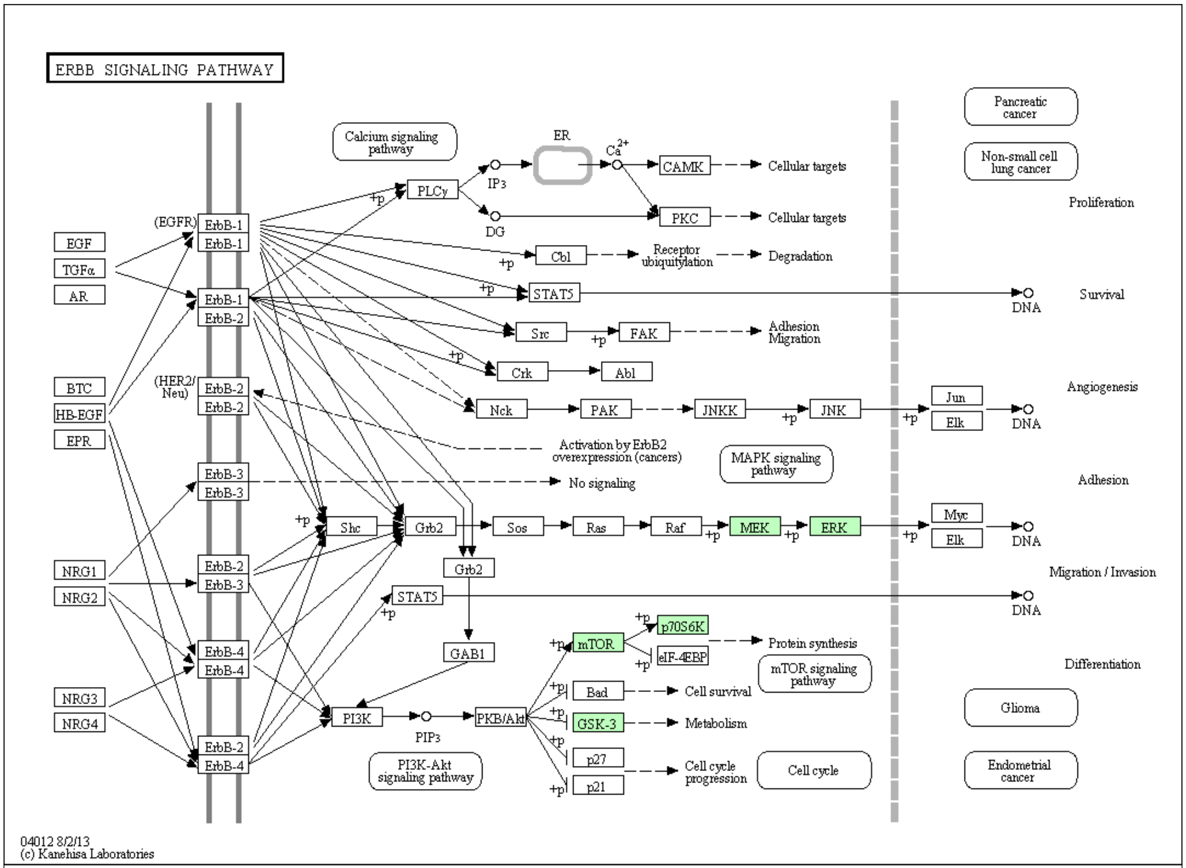
ERBB SIGNALING PATHWAY
Calcium signaling pathway
ER
Ca2+
IP3
DG
PLCγ
CAMK
PKC
Cellular targets
Cellular targets
Pancreatic cancer
Non-small cell lung cancer
Proliferation
(EGFR)
ErbB-1
ErbB-1
EGF
TGFα
AR
ErbB-1
ErbB-2
Cbl
Receptor ubiquitylation
Degradation
STAT5
DNA
Survival
Src
FAK
Adhesion Migration
Crk
Abl
BTC
HB-EGF
EPR
(HER2/ Neu)
ErbB-2
ErbB-2
Nck
PAK
JNKK
JNK
Jun
Elk
DNA
Angiogenesis
Activation by ErbB2 overexpression (cancers)
MAPK signaling pathway
ErbB-3
ErbB-3
No signaling
Adhesion
Shc
Grb2
Sos
Ras
Raf
MEK
ERK
Myc
Elk
DNA
NRG1
NRG2
ErbB-2
ErbB-3
Grb2
STAT5
DNA
Migration / Invasion
mTOR
p70S6K
eIF-4EBP
Protein synthesis
mTOR signaling pathway
Differentiation
ErbB-4
ErbB-4
GAB1
NRG3
NRG4
Bad
Cell survival
Glioma
PI3K
PIP3
PKB/Akt
GSK-3
Metabolism
ErbB-2
ErbB-4
p27
p21
Cell cycle progression
Cell cycle
Endometrial cancer
PI3K-Akt signaling pathway
+p
04012 8/2/13
(c) Kanehisa Laboratories

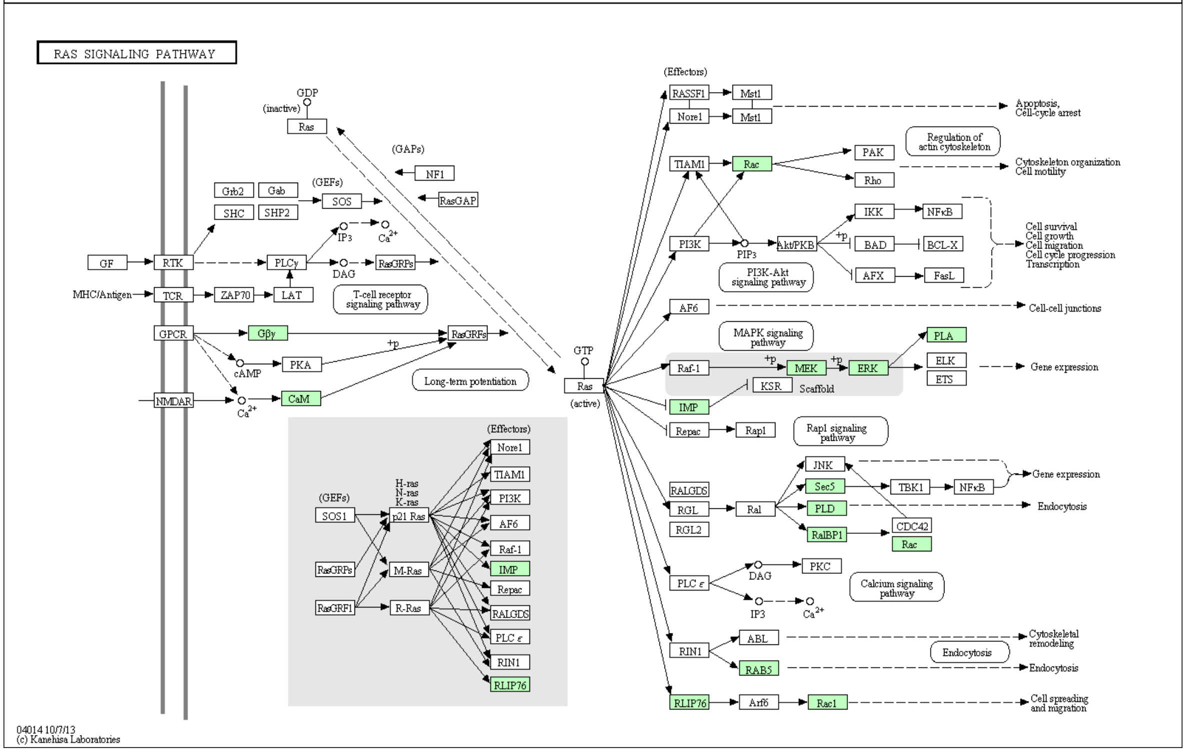
RAS SIGNALING PATHWAY
GDP
(inactive)
Ras
(GAPs)
NF1
RasGAP
(GEFs)
Grb2
Gab
SOS
SHC
SHP2
IP3
Ca2+
DAG
GF
RTK
PLCγ
RasGRPs
MHC/Antigen
TCR
ZAP70
LAT
T-cell receptor signaling pathway
GPCR
Gβγ
RasGRFs
cAMP
PKA
+p
Long-term potentiation
NMDAR
CaM
Ca2+
GTP
Ras
(active)
(Effectors)
RASSF1
Mst1
Nore1
Mst1
Apoptosis, Cell-cycle arrest
Regulation of actin cytoskeleton
TIAM1
Rac
PAK
Rho
Cytoskeleton organization Cell motility
IKK
NFκB
PI3K
PIP3
Akt/PKB
BAD
BCL-X
AFX
FasL
Cell survival Cell growth Cell migration Cell cycle progression Transcription
PI3K-Akt signaling pathway
AF6
Cell-cell junctions
MAPK signaling pathway
Raf-1
MEK
ERK
PLA
ELK
ETS
Gene expression
KSR
Scaffold
IMP
Repac
Rap1
Rap1 signaling pathway
(Effectors)
Nore1
TIAM1
PI3K
AF6
Raf-1
IMP
Repac
RALGDS
PLC ε
RIN1
RLIP76
(GEFs)
SOS1
H-ras
N-ras
K-ras
p21 Ras
RasGRPs
M-Ras
RasGRF1
R-Ras
JNK
Gene expression
RALGDS
Sec5
TBK1
NFκB
RGL
Ral
PLD
Endocytosis
RGL2
RalBP1
CDC42
Rac
PLC ε
DAG
PKC
IP3
Ca2+
Calcium signaling pathway
ABL
Cytoskeletal remodeling
RIN1
Endocytosis
RAB5
Endocytosis
RLIP76
Arf6
Rac1
Cell spreading and migration
04014 10/7/13
(c) Kanehisa Laboratories

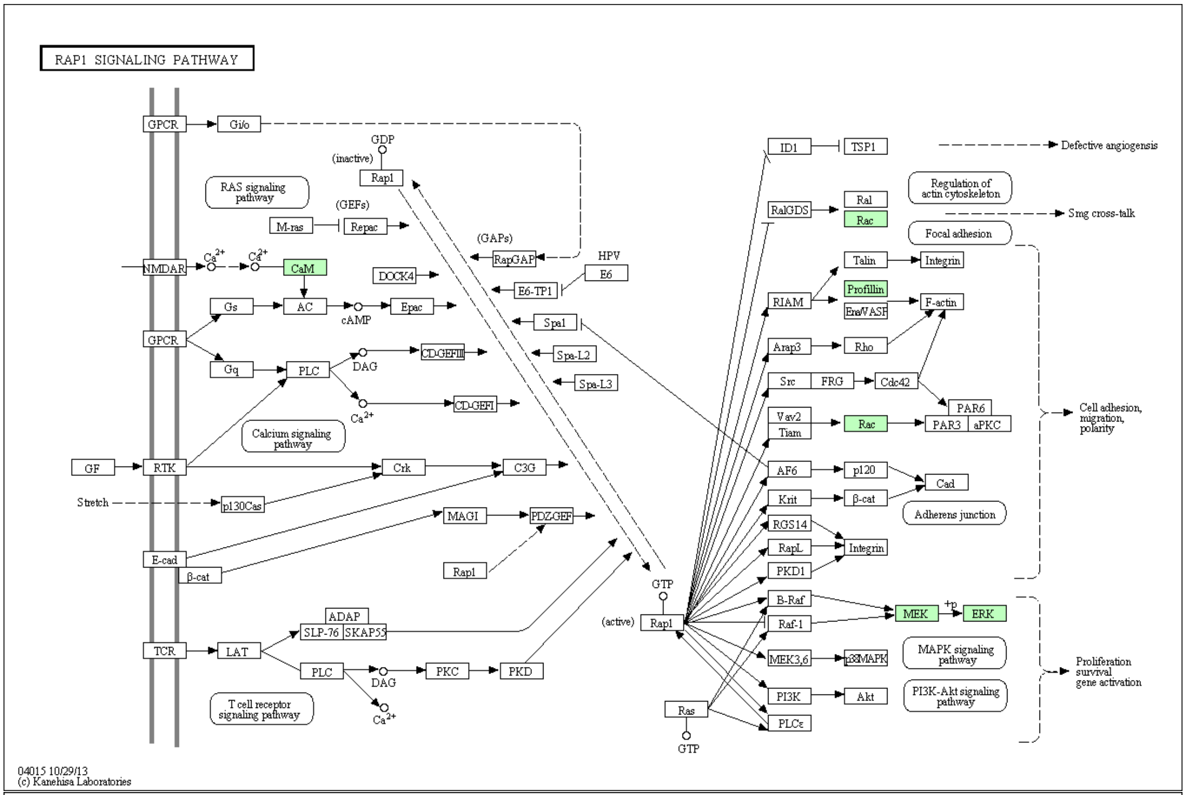
RAP1 SIGNALING PATHWAY
GPCR
Gi/o
GDP
(inactive)
Rap1
RAS signaling pathway
(GEFs)
M-ras
Repac
(GAPs)
RapGAP
HPV
E6
E6-TP1
NMDAR
CaM
DOCK4
Gs
AC
cAMP
Epac
Spa1
Spa-L2
Spa-L3
GPCR
Gq
PLC
DAG
CDGEFIII
CD-GEFI
Calcium signaling pathway
GF
RTK
Crk
C3G
Stretch
p130Cas
MAGI
PDZGEF
E-cad
β-cat
Rap1
ADAP
SLP-76
SKAP55
TCR
LAT
PLC
DAG
PKC
PKD
T cell receptor signaling pathway
GTP
(active)
Rap1
Ras
GTP
ID1
TSP1
Defective angiogensis
Regulation of actin cytoskeleton
RalGDS
Ral
Rac
Smg cross-talk
Focal adhesion
Talin
Integrin
RIAM
Profillin
Ena/VASP
F-actin
Arap3
Rho
Src
FRG
Cdc42
PAR6
Vav2
Tiam
Rac
PAR3
aPKC
Cell adhesion, migration, polarity
AF6
p120
Cad
Krit
β-cat
Adherens junction
RGS14
RapL
Integrin
PKD1
B-Raf
Raf-1
MEK
ERK
+p
MEK3,6
p38MAPK
MAPK signaling pathway
PI3K
Akt
PI3K-Akt signaling pathway
PLCε
Proliferation survival gene activation
04015 10/29/13
(c) Kanehisa Laboratories

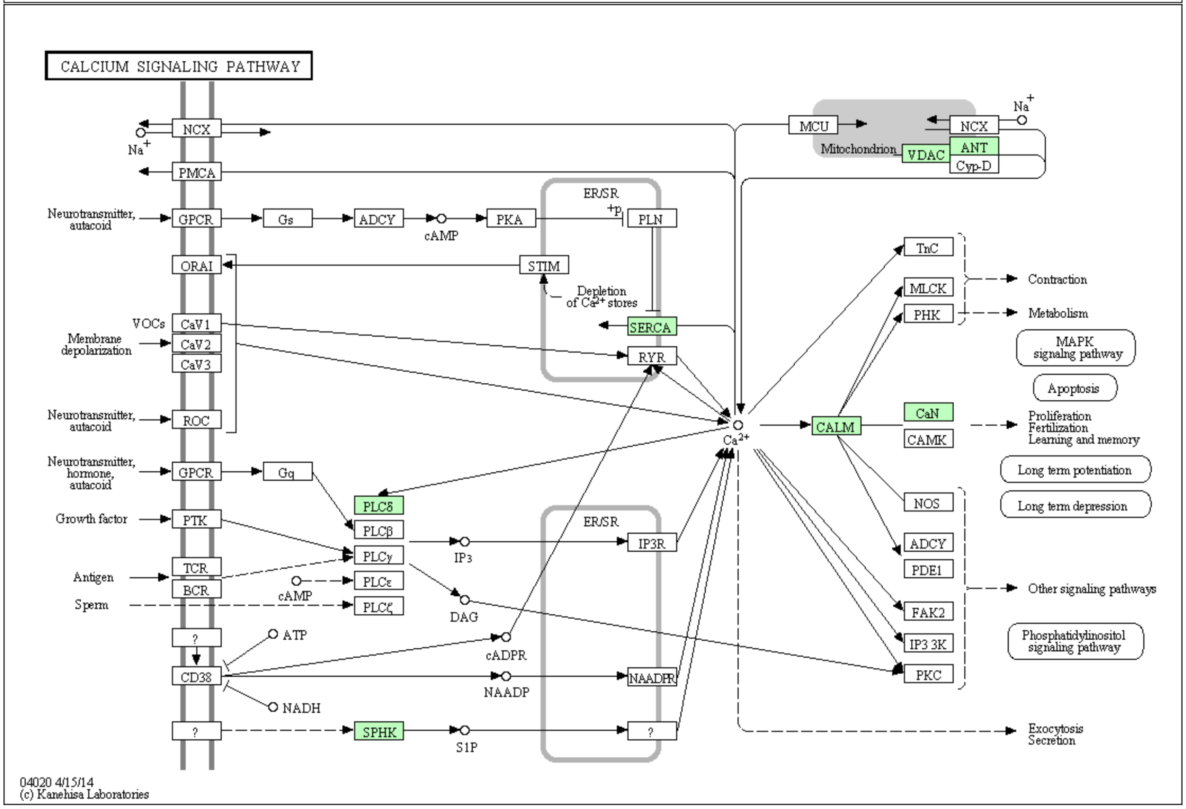
CALCIUM SIGNALING PATHWAY
NCX
Na+
PMCA
Neurotransmitter, autacoid
GPCR
Gs
ADCY
cAMP
PKA
ER/SR
+p
PLN
ORAI
STIM
Depletion of Ca2+ stores
SERCA
VOCs
CaV1
Membrane depolarization
CaV2
CaV3
RYR
Neurotransmitter, autacoid
ROC
Neurotransmitter, hormone, autacoid
GPCR
Gq
PLCδ
Growth factor
PTK
PLCβ
IP3
ER/SR
IP3R
Antigen
TCR
PLCγ
BCR
cAMP
PLCε
Sperm
PLCζ
DAG
?
ATP
cADPR
CD38
NAADP
NAADPR
NADH
?
SPHK
S1P
?
MCU
Mitochondrion
NCX
Na+
VDAC
ANT
Cyp-D
Ca2+
CALM
TnC
MLCK
PHK
Contraction
Metabolism
MAPK signaling pathway
Apoptosis
CaN
CAMK
Proliferation Fertilization Learning and memory
Long term potentiation
Long term depression
NOS
ADCY
PDE1
Other signaling pathways
FAK2
Phosphatidylinositol signaling pathway
IP3 3K
PKC
Exocytosis Secretion
04020 4/15/14
(c) Kanehisa Laboratories

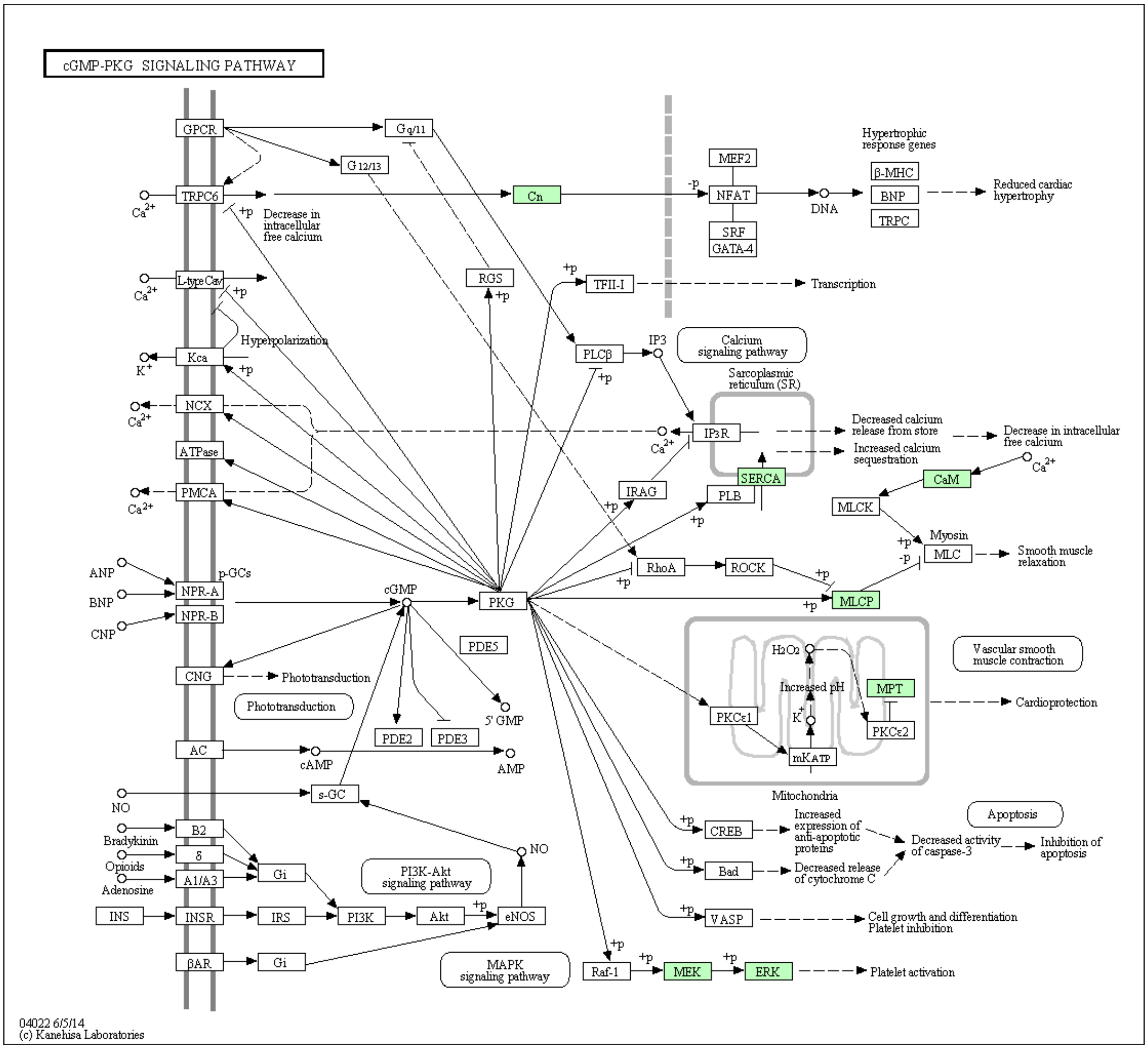
cGMP-PKG SIGNALING PATHWAY
GPCR
Gq/11
G12/13
Hypertrophic response genes
MEF2
-p
NFAT
DNA
SRF
GATA-4
β-MHC
BNP
TRPC
Reduced cardiac hypertrophy
Ca2+
TRPC6
+p
Decrease in intracellular free calcium
Cn
RGS
+p
TFII-I
Transcription
L-type Cav
Hyperpolarization
Kca
K+
PLCβ
IP3
Calcium signaling pathway
Sarcoplasmic reticulum (SR)
NCX
IP3R
Decreased calcium release from store
Increased calcium sequestration
Decrease in intracellular free calcium
ATPase
SERCA
CaM
PMCA
IRAG
PLB
MLCK
Myosin
MLC
Smooth muscle relaxation
ANP
BNP
CNP
p-GCs
NPR-A
NPR-B
cGMP
PKG
RhoA
ROCK
MLCP
PDE5
CNG
Phototransduction
PDE2
PDE3
5' GMP
H2O2
Increased pH
MPT
PKCε1
PKCε2
mKATP
Mitochondria
Vascular smooth muscle contraction
Cardioprotection
AC
cAMP
AMP
NO
s-GC
CREB
Increased expression of anti-apoptotic proteins
Decreased activity of caspase-3
Inhibition of apoptosis
Apoptosis
B2
Bradykinin
δ
Opioids
A1/A3
Adenosine
Gi
PI3K-Akt signaling pathway
Bad
Decreased release of cytochrome C
INS
INSR
IRS
PI3K
Akt
eNOS
VASP
Cell growth and differentiation
Platelet inhibition
βAR
MAPK signaling pathway
Raf-1
MEK
ERK
Platelet activation
04022 6/5/14
(c) Kanehisa Laboratories

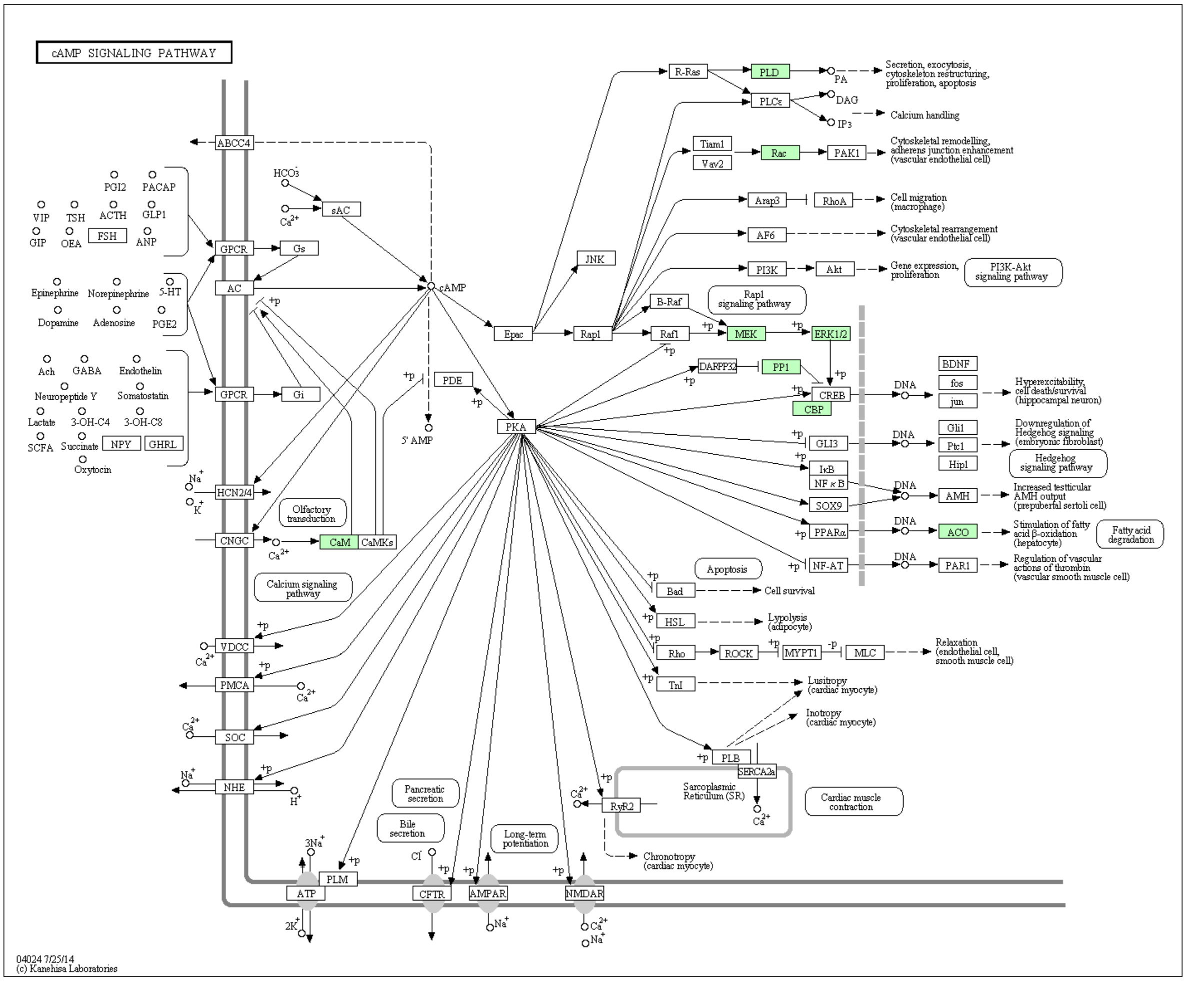
cAMP SIGNALING PATHWAY
ABCC4
PGI2 PACAP
VIP TSH ACTH GLP1
GIP OEA FSH ANP
GPCR Gs
HCO3
Ca2+
sAC
Epinephrine Norepinephrine 5-HT
Dopamine Adenosine PGE2
AC
cAMP
Ach GABA Endothelin
Neuropeptide Y Somatostatin
Lactate 3-OH-C4 3-OH-C8
SCFA Succinate NPY GHRL
Oxytocin
GPCR Gi
PDE
5' AMP
PKA
Epac Rap1
JNK
R-Ras PLD PA
PLCε DAG IP3
Secretion, exocytosis, cytoskeleton restructuring, proliferation, apoptosis
Calcium handling
Tiam1 Vav2 Rac PAK1
Cytoskeletal remodelling, adherens junction enhancement (vascular endothelial cell)
Arap3 RhoA
Cell migration (macrophage)
AF6
Cytoskeletal rearrangement (vascular endothelial cell)
PI3K Akt
Gene expression, proliferation
PI3K-Akt signaling pathway
B-Raf
Rap1 signaling pathway
Raf1 MEK ERK1/2
DARPP32 PP1
CREB CBP
DNA
BDNF fos jun
Hyperexcitability, cell death/survival (hippocampal neuron)
GLI3
Gli1 Ptc1 Hip1
Downregulation of Hedgehog signaling (embryonic fibroblast)
Hedgehog signaling pathway
IκB NF κB
SOX9 AMH
Increased testicular AMH output (prepubertal sertoli cell)
PPARα ACO
Stimulation of fatty acid β-oxidation (hepatocyte)
Fatty acid degradation
NF-AT PAR1
Regulation of vascular actions of thrombin (vascular smooth muscle cell)
Apoptosis
Bad
Cell survival
HSL
Lypolysis (adipocyte)
Rho ROCK MYPT1 MLC
Relaxation (endothelial cell, smooth muscle cell)
TnI
Lusitropy (cardiac myocyte)
Inotropy (cardiac myocyte)
PLB SERCA2a
Sarcoplasmic Reticulum (SR)
Cardiac muscle contraction
RyR2
Chronotropy (cardiac myocyte)
Na+ K+
HCN2/4
Olfactory transduction
CNGC CaM CaMKs
Calcium signaling pathway
VDCC
PMCA
SOC
NHE
H+
Pancreatic secretion
Bile secretion
Long-term potentiation
3Na+ 2K+
ATP PLM
Cl-
CFTR AMPAR NMDAR
+p
04024 7/25/14
(c) Kanehisa Laboratories

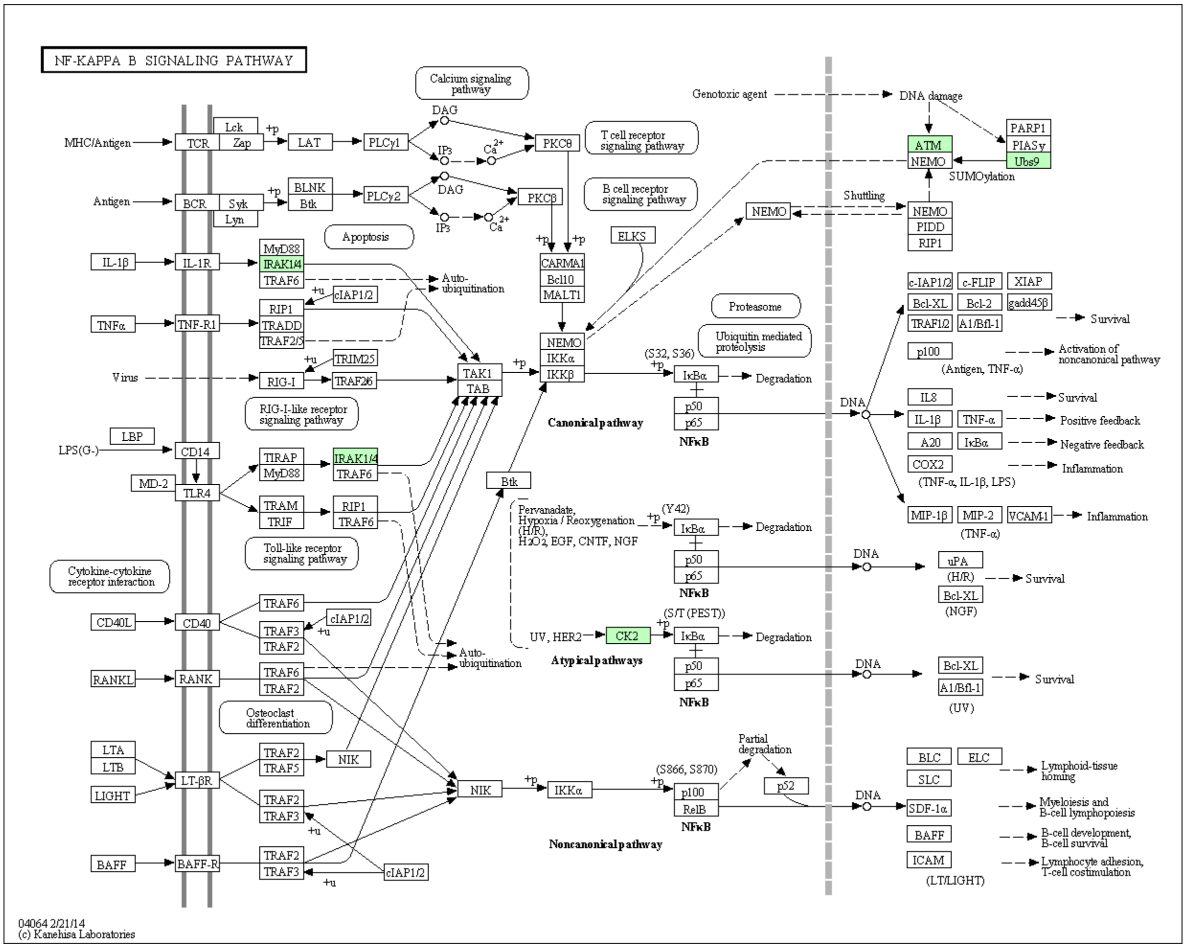
NF-KAPPA B SIGNALING PATHWAY
Calcium signaling pathway
T cell receptor signaling pathway
B cell receptor signaling pathway
MHC/Antigen
TCR
Lck
Zap
+p
LAT
PLCγ1
DAG
IP3
Ca2+
PKCθ
Antigen
BCR
Syk
Lyn
BLNK
Btk
PLCγ2
PKCβ
Apoptosis
IL-1β
IL-1R
MyD88
IRAK1/4
TRAF6
Auto-ubiquitination
+u
cIAP1/2
TNFα
TNF-R1
RIP1
TRADD
TRAF2/5
TRIM25
Virus
RIG-I
TRAF2/6
RIG-I-like receptor signaling pathway
TAK1
TAB
CARMA1
Bcl10
MALT1
ELKS
NEMO
IKKα
IKKβ
Genotoxic agent
DNA damage
ATM
PARP1
PIASy
Ubc9
SUMOylation
Shuttling
PIDD
Proteasome
Ubiquitin mediated proteolysis
(S32, S36)
IκBα
Degradation
p50
p65
NFκB
Canonical pathway
DNA
c-IAP1/2
c-FLIP
XIAP
Bcl-XL
Bcl-2
gadd45β
TRAF1/2
A1/Bfl-1
Survival
p100
Activation of noncanonical pathway
(Antigen, TNF-α)
IL8
TNF-α
Positive feedback
A20
Negative feedback
COX2
Inflammation
(TNF-α, IL-1β, LPS)
MIP-1β
MIP-2
VCAM-1
(TNF-α)
LBP
LPS(G-)
CD14
MD-2
TLR4
TIRAP
TRAM
TRIF
Toll-like receptor signaling pathway
Cytokine-cytokine receptor interaction
Pervanadate, Hypoxia / Reoxygenation (H/R), H2O2, EGF, CNTF, NGF
(Y42)
uPA
(H/R)
(NGF)
CD40L
CD40
TRAF3
TRAF2
UV, HER2
CK2
(S/T (PEST))
Atypical pathways
(UV)
RANKL
RANK
Osteoclast differentiation
LTA
LTB
LIGHT
LT-βR
TRAF5
NIK
IKKα
(S866, S870)
Partial degradation
p52
RelB
Noncanonical pathway
BLC
ELC
SLC
SDF-1α
ICAM
Lymphoid-tissue homing
Myeloiesis and B-cell lymphopoiesis
B-cell development, B-cell survival
Lymphocyte adhesion, T-cell costimulation
(LT/LIGHT)
BAFF
BAFF-R
04064 2/21/14
(c) Kanehisa Laboratories

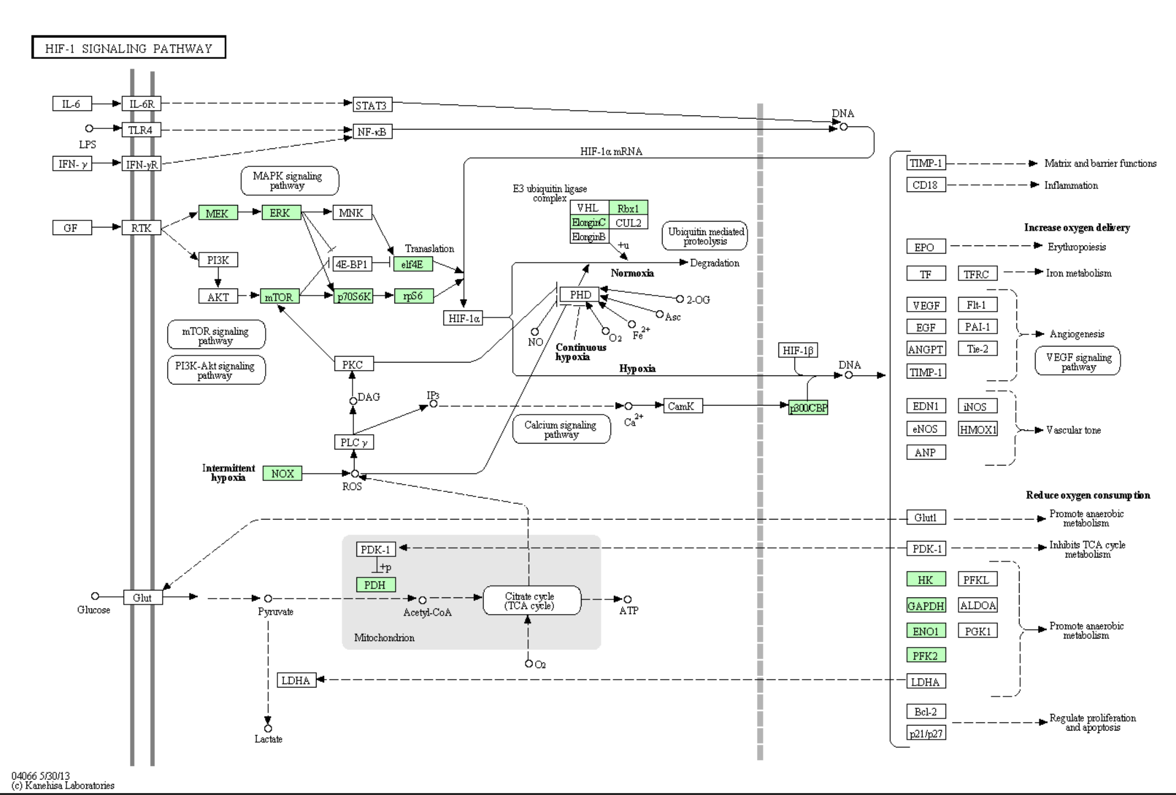
HIF-1 SIGNALING PATHWAY
IL-6
IL-6R
STAT3
LPS
TLR4
NF-κB
IFN-γ
IFN-γR
DNA
HIF-1α mRNA
MAPK signaling pathway
GF
RTK
MEK
ERK
MNK
E3 ubiquitin ligase complex
VHL
Rbx1
ElonginC
CUL2
ElonginB
+u
Ubiquitin mediated proteolysis
Translation
PI3K
4E-BP1
eIF4E
Degradation
Normoxia
AKT
mTOR
p70S6K
rpS6
PHD
2-OG
HIF-1α
Asc
NO
O2
Fe2+
mTOR signaling pathway
Continuous hypoxia
PI3K-Akt signaling pathway
PKC
Hypoxia
HIF-1β
DNA
DAG
IP3
Ca2+
CamK
p300/CBP
Calcium signaling pathway
PLCγ
Intermittent hypoxia
NOX
ROS
PDK-1
+p
PDH
Glucose
Glut
Pyruvate
Acetyl-CoA
Citrate cycle (TCA cycle)
ATP
Mitochondrion
O2
LDHA
Lactate
TIMP-1
Matrix and barrier functions
CD18
Inflammation
Increase oxygen delivery
EPO
Erythropoiesis
TF
TFRC
Iron metabolism
VEGF
Flt-1
EGF
PAI-1
Angiogenesis
ANGPT
Tie-2
VEGF signaling pathway
TIMP-1
EDN1
iNOS
eNOS
HMOX1
Vascular tone
ANP
Reduce oxygen consumption
Glut1
Promote anaerobic metabolism
PDK-1
Inhibits TCA cycle metabolism
HK
PFKL
GAPDH
ALDOA
ENO1
PGK1
Promote anaerobic metabolism
PFK2
LDHA
Bcl-2
Regulate proliferation and apoptosis
p21/p27
04066 5/30/13
(c) Kanehisa Laboratories

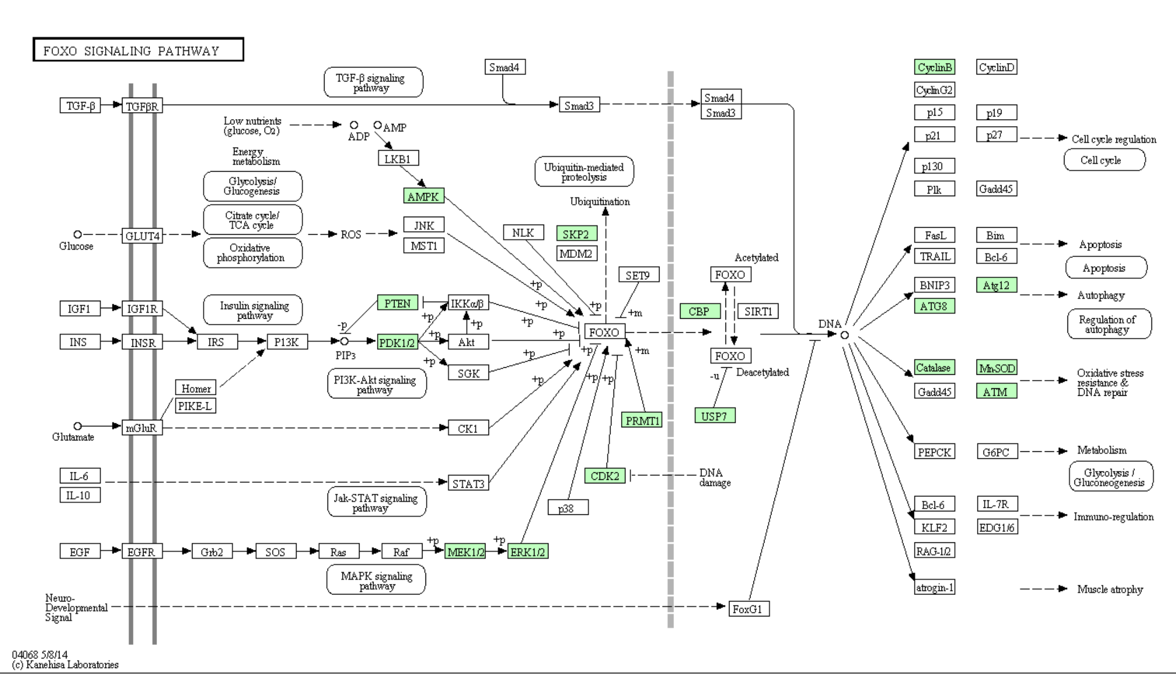
FOXO SIGNALING PATHWAY
TGF-β signaling pathway
Smad4
TGF-β
TGFβR
Smad3
Smad4
Smad3
Low nutrients (glucose, O2)
ADP
AMP
Energy metabolism
LKB1
Glycolysis / Gluconeogenesis
Ubiquitin-mediated proteolysis
AMPK
Ubiquitination
Citrate cycle / TCA cycle
Glucose
GLUT4
ROS
JNK
MST1
Oxidative phosphorylation
NLK
SKP2
MDM2
SET9
Acetylated
FOXO
IGF1
IGF1R
Insulin signaling pathway
PTEN
IKKα/β
CBP
SIRT1
INS
INSR
IRS
PI3K
PIP3
PDK1/2
Akt
FOXO
DNA
+p
-p
+m
-u
Deacetylated
SGK
Homer
PIKE-L
PI3K-Akt signaling pathway
USP7
PRMT1
Glutamate
mGluR
CK1
IL-6
DNA damage
IL-10
STAT3
CDK2
Jak-STAT signaling pathway
p38
EGF
EGFR
Grb2
SOS
Ras
Raf
MEK1/2
ERK1/2
MAPK signaling pathway
Neuro-Developmental Signal
FoxG1
CyclinB
CyclinD
CyclinG2
p15
p19
p21
p27
Cell cycle regulation
Cell cycle
p130
Plk
Gadd45
FasL
Bim
Apoptosis
TRAIL
Bcl-6
Apoptosis
BNIP3
Atg12
Autophagy
ATG8
Regulation of autophagy
Catalase
MnSOD
Oxidative stress resistance & DNA repair
Gadd45
ATM
PEPCK
G6PC
Metabolism
Glycolysis / Gluconeogenesis
Bcl-6
IL-7R
Immuno-regulation
KLF2
EDG1/6
RAG-1/2
atrogin-1
Muscle atrophy
04068 5/8/14
(c) Kanehisa Laboratories

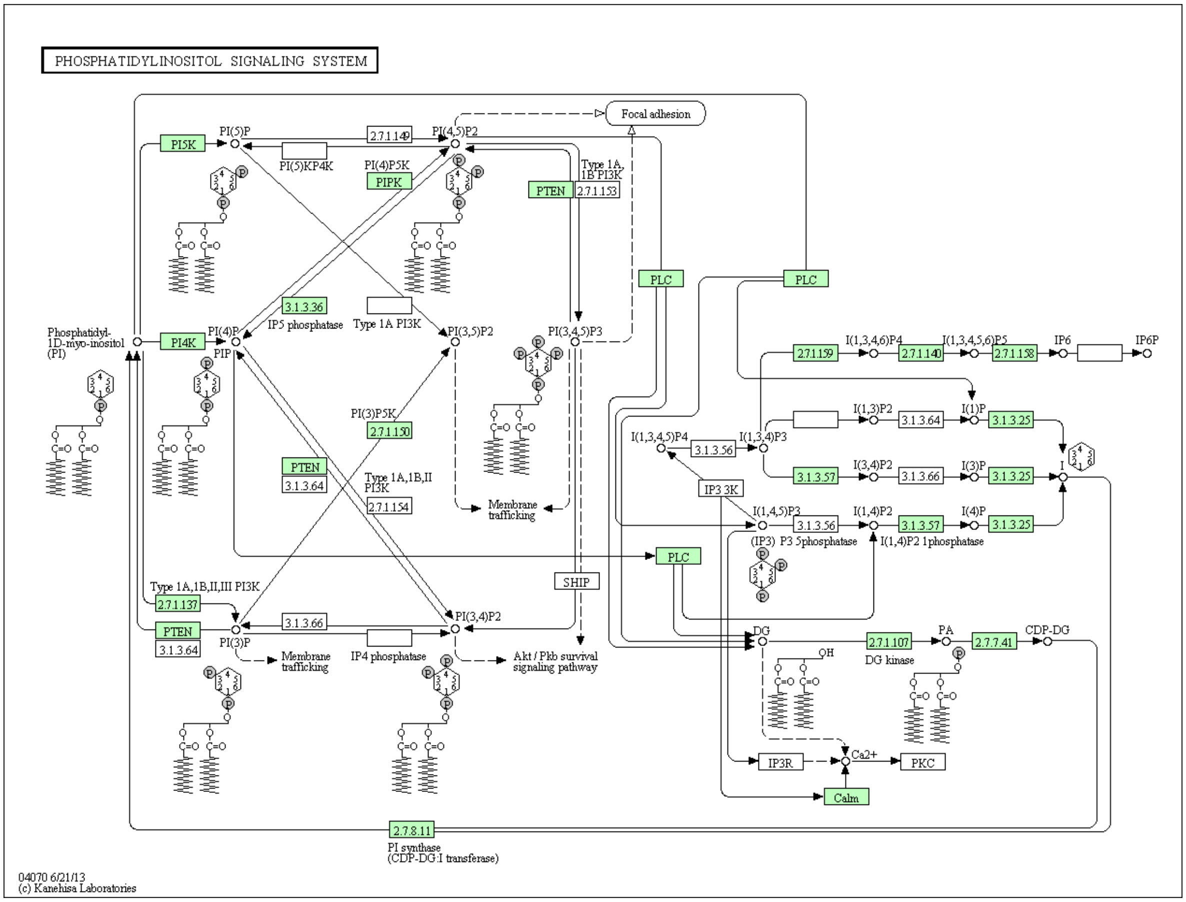

PHOSPHATIDYLINOSITOL SIGNALING SYSTEM
Focal adhesion
PI5K
PI(5)P
2.7.1.149
PI(4,5)P2
PI(5)P4K
PI(4)P5K
PIPK
Type 1A, 1B PI3K
PTEN
2.7.1.153
PLC
3.1.3.36
IP5 phosphatase
Type 1A PI3K
Phosphatidyl-1D-myo-inositol (PI)
PI4K
PI(4)P
PIP
PI(3,5)P2
PI(3,5)P3
PI(3)P5K
2.7.1.150
PTEN
3.1.3.64
Type 1A,1B,II PI3K
2.7.1.154
Membrane trafficking
SHIP
Type 1A,1B,II,III PI3K
2.7.1.137
PTEN
3.1.3.64
PI(3)P
3.1.3.66
IP4 phosphatase
PI(3,4)P2
Membrane trafficking
Akt / Pkb survival signaling pathway
2.7.1.159
I(1,3,4,6)P4
2.7.1.140
I(1,3,4,5,6)P5
2.7.1.158
IP6
IP6P
I(1,3)P2
3.1.3.64
I(1)P
3.1.3.25
I(1,3,4,5)P4
3.1.3.56
I(1,3,4)P3
3.1.3.57
I(3,4)P2
3.1.3.66
I(3)P
3.1.3.25
I
IP3 3K
I(1,4,5)P3
(IP3)
3.1.3.56
P3 5phosphatase
I(1,4)P2
3.1.3.57
I(1,4)P2 1phosphatase
I(4)P
3.1.3.25
DG
2.7.1.107
DG kinase
PA
2.7.7.41
CDP-DG
IP3R
Ca2+
PKC
Calm
2.7.8.11
PI synthase (CDP-DG:I transferase)
04070 6/21/13
(c) Kanehisa Laboratories

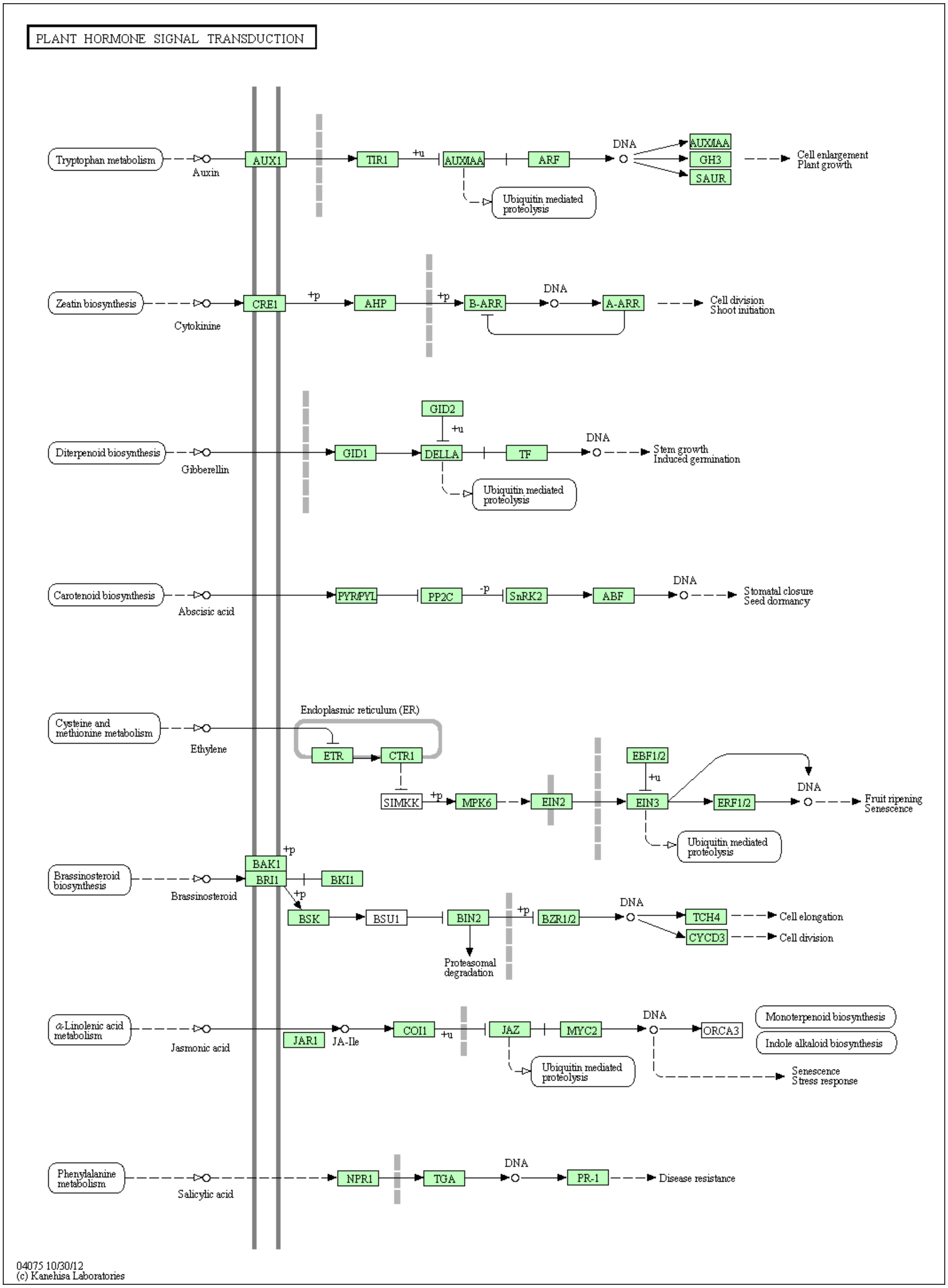
PLANT HORMONE SIGNAL TRANSDUCTION
Tryptophan metabolism
Auxin
AUX1
TIR1
+u
AUX/IAA
ARF
DNA
AUX/IAA
GH3
SAUR
Cell enlargement
Plant growth
Ubiquitin mediated proteolysis
Zeatin biosynthesis
Cytokinine
CRE1
+p
AHP
+p
B-ARR
DNA
A-ARR
Cell division
Shoot initiation
Diterpenoid biosynthesis
Gibberellin
GID1
GID2
+u
DELLA
TF
DNA
Stem growth
Induced germination
Ubiquitin mediated proteolysis
Carotenoid biosynthesis
Abscisic acid
PYR/PYL
PP2C
-p
SnRK2
ABF
DNA
Stomatal closure
Seed dormancy
Cysteine and methionine metabolism
Ethylene
Endoplasmic reticulum (ER)
ETR
CTR1
SIMKK
+p
MPK6
EIN2
EBF1/2
+u
EIN3
ERF1/2
DNA
Fruit ripening
Senescence
Ubiquitin mediated proteolysis
Brassinosteroid biosynthesis
Brassinosteroid
BAK1
+p
BRI1
BKI1
+p
BSK
BSU1
BIN2
+p
BZR1/2
DNA
TCH4
CYCD3
Cell elongation
Cell division
Proteasomal degradation
α-Linolenic acid metabolism
Jasmonic acid
JAR1
JA-Ile
COI1
+u
JAZ
MYC2
DNA
ORCA3
Monoterpenoid biosynthesis
Indole alkaloid biosynthesis
Ubiquitin mediated proteolysis
Senescence
Stress response
Phenylalanine metabolism
Salicylic acid
NPR1
TGA
DNA
PR-1
Disease resistance
04075 10/30/12
(c) Kanehisa Laboratories

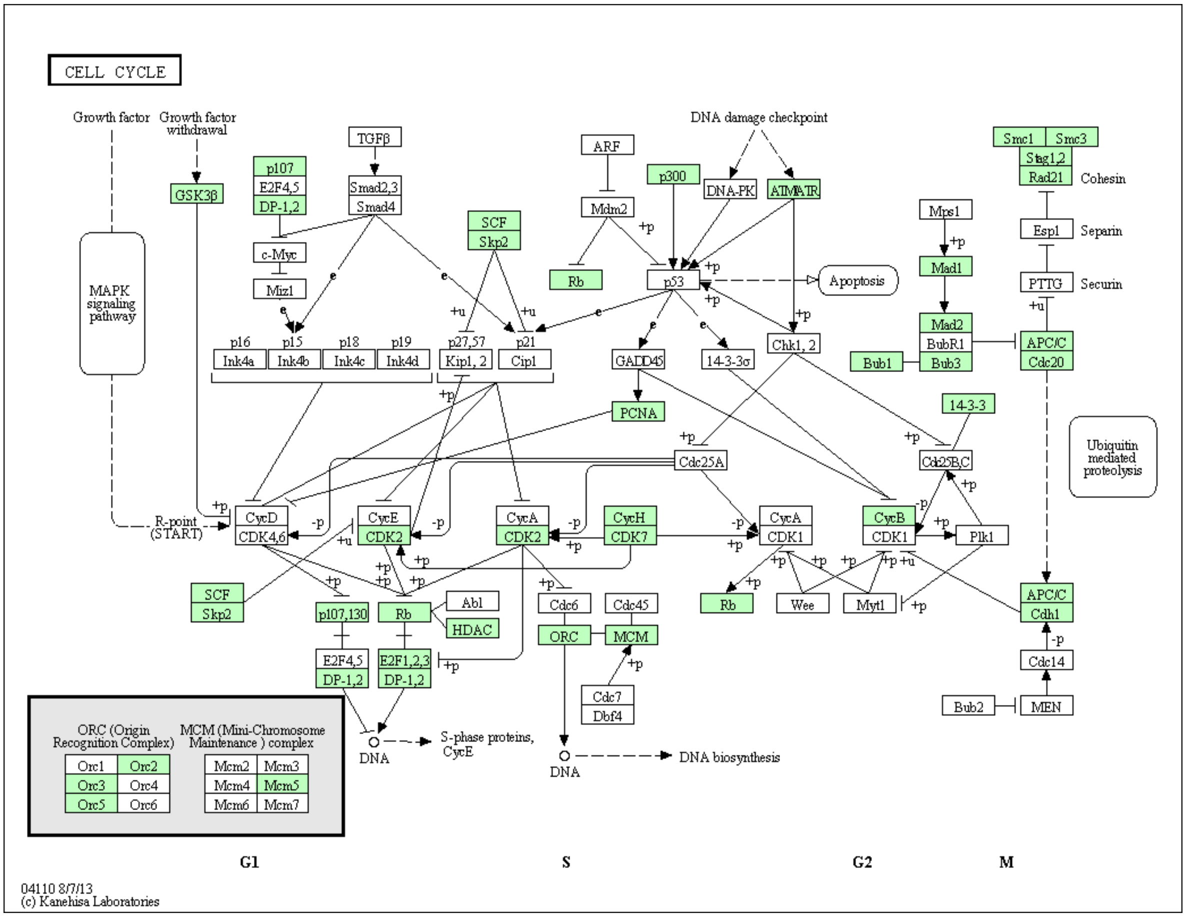
CELL CYCLE
Growth factor
Growth factor withdrawal
GSK3β
MAPK signaling pathway
R-point (START)
p107
E2F4,5
DP-1,2
TGFβ
Smad2,3
Smad4
c-Myc
Miz1
p16 Ink4a
p15 Ink4b
p18 Ink4c
p19 Ink4d
SCF
Skp2
p27,57 Kip1, 2
p21 Cip1
ARF
Mdm2
Rb
p300
DNA damage checkpoint
DNA-PK
ATM/ATR
p53
Apoptosis
GADD45
14-3-3σ
Chk1, 2
PCNA
Cdc25A
CycD CDK4,6
CycE CDK2
CycA CDK2
CycH CDK7
CycA CDK1
CycB CDK1
Plk1
Mps1
Mad1
Mad2
BubR1
Bub1
Bub3
14-3-3
Cdc25B,C
Smc1
Smc3
Stag1,2
Rad21
Cohesin
Esp1
Separin
PTTG
Securin
APC/C Cdc20
Ubiquitin mediated proteolysis
APC/C Cdh1
Cdc14
MEN
Bub2
Wee
Myt1
p107,130
Abl
HDAC
E2F4,5 DP-1,2
E2F1,2,3 DP-1,2
Cdc6
Cdc45
ORC
MCM
Cdc7
Dbf4
DNA
S-phase proteins, CycE
DNA biosynthesis
ORC (Origin Recognition Complex)
Orc1 Orc2 Orc3 Orc4 Orc5 Orc6
MCM (Mini-Chromosome Maintenance) complex
Mcm2 Mcm3 Mcm4 Mcm5 Mcm6 Mcm7
G1
S
G2
M
04110 8/7/13
(c) Kanehisa Laboratories

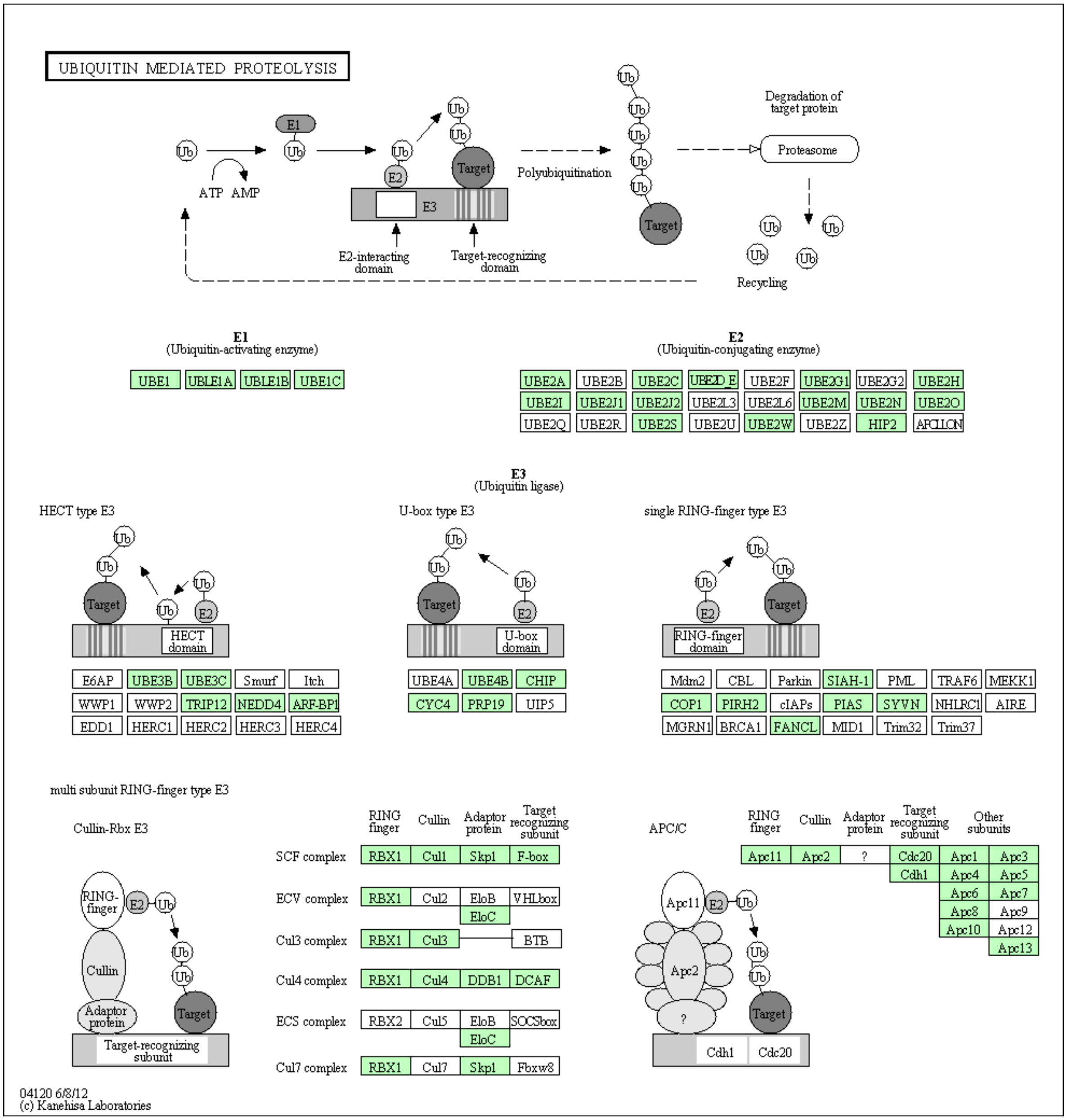

UBIQUITIN MEDIATED PROTEOLYSIS
Ub
E1
E2
E3
ATP AMP
Target
E2-interacting domain
Target-recognizing domain
Polyubiquitination
Degradation of target protein
Proteasome
Recycling
E1
(Ubiquitin-activating enzyme)
UBE1 UBLE1A UBLE1B UBE1C
E2
(Ubiquitin-conjugating enzyme)
UBE2A UBE2B UBE2C UBE2D_E UBE2F UBE2G1 UBE2G2 UBE2H
UBE2I UBE2J1 UBE2J2 UBE2L3 UBE2L6 UBE2M UBE2N UBE2O
UBE2Q UBE2R UBE2S UBE2U UBE2W UBE2Z HIP2
E3
(Ubiquitin ligase)
HECT type E3
HECT domain
E6AP UBE3B UBE3C Smurf Itch
WWP1 WWP2 TRIP12 NEDD4 ARF-BP1
EDD1 HERC1 HERC2 HERC3 HERC4
U-box type E3
U-box domain
UBE4A UBE4B CHIP
CYC4 PRP19 UIP5
single RING-finger type E3
RING-finger domain
Mdm2 CBL Parkin SIAH-1 PML TRAF6 MEKK1
COP1 PIRH2 cIAPs PIAS SYVN NHLRC1 AIRE
MGRN1 BRCA1 FANCL MID1 Trim32 Trim37
multi subunit RING-finger type E3
Cullin-Rbx E3
RING-finger
Cullin
Adaptor protein
Target-recognizing subunit
RING finger
Cullin
Adaptor protein
Target recognizing subunit
SCF complex RBX1 Cul1 Skp1 F-box
ECV complex RBX1 Cul2 EloB VHLbox EloC
Cul3 complex RBX1 Cul3 BTB
Cul4 complex RBX1 Cul4 DDB1 DCAF
ECS complex RBX2 Cul5 EloB SOCSbox EloC
Cul7 complex RBX1 Cul7 Skp1 Fbxw8
APC/C
RING finger
Cullin
Adaptor protein
Target recognizing subunit
Other subunits
Apc11 Apc2 ? Cdc20 Apc1 Apc3
Cdh1 Apc4 Apc5
Apc6 Apc7
Apc8 Apc9
Apc10 Apc12
Apc13
Apc11
Apc2
?
Cdh1 Cdc20
04120 6/8/12
(c) Kanehisa Laboratories

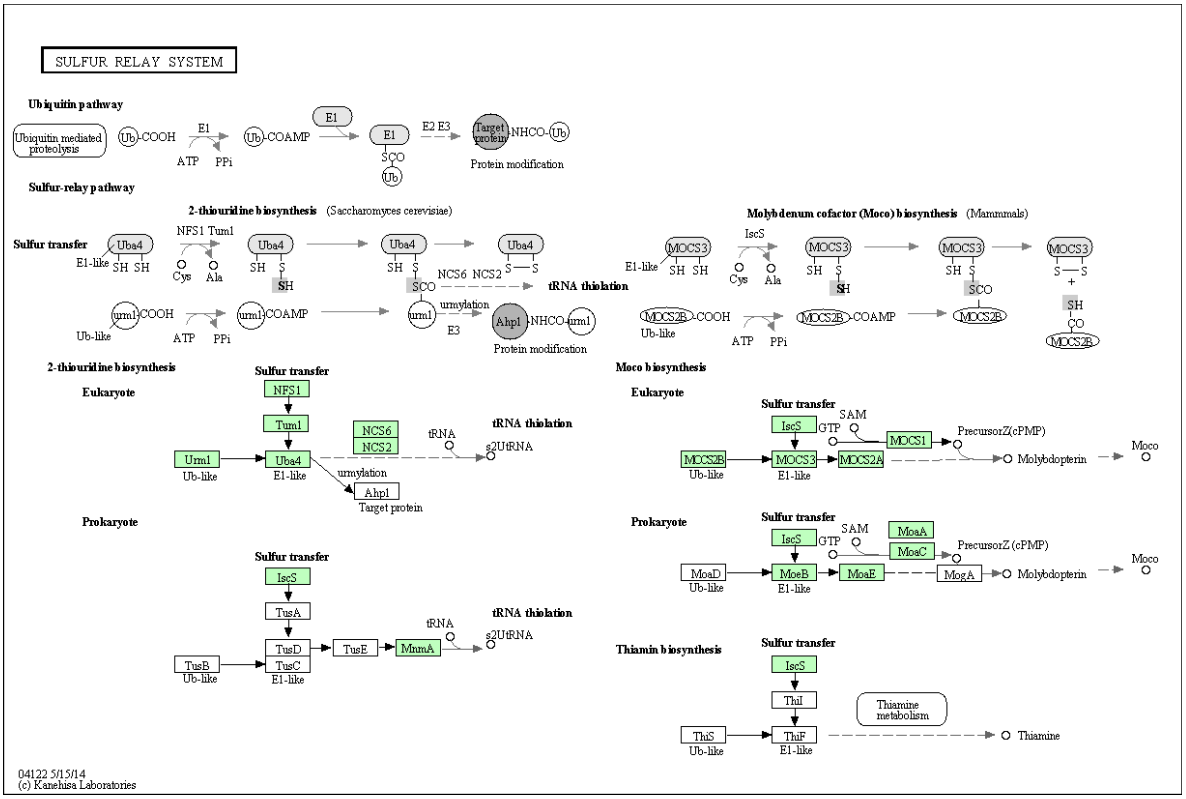

SULFUR RELAY SYSTEM
Ubiquitin pathway
Ubiquitin mediated proteolysis
Ub-COOH
E1
ATP
PPi
Ub-COAMP
E1
SCO
Ub
E2 E3
Target protein-NHCO-Ub
Protein modification
Sulfur-relay pathway
2-thiouridine biosynthesis (Saccharomyces cerevisiae)
Sulfur transfer
Uba4
E1-like
SH SH
NFS1 Tum1
Cys
Ala
SH
NCS6 NCS2
tRNA thiolation
urm1-COOH
Ub-like
urm1-COAMP
urmylation
E3
Ahp1-NHCO-urm1
Protein modification
Molybdenum cofactor (Moco) biosynthesis (Mammals)
MOCS3
IscS
MOCS2B-COOH
MOCS2B-COAMP
MOCS2B
2-thiouridine biosynthesis
Eukaryote
Sulfur transfer
NFS1
Tum1
NCS6
NCS2
tRNA
s2UtRNA
Urm1
Ub-like
Uba4
E1-like
urmylation
Ahp1
Target protein
Prokaryote
IscS
TusA
TusD
TusC
TusB
TusE
MnmA
Moco biosynthesis
SAM
GTP
MOCS1
PrecursorZ (cPMP)
MOCS2B
MOCS3
MOCS2A
Molybdopterin
Moco
MoaA
MoaC
MoaD
MoeB
MoaE
MogA
Thiamin biosynthesis
ThiI
Thiamine metabolism
ThiS
ThiF
Thiamine
04122 5/15/14
(c) Kanehisa Laboratories

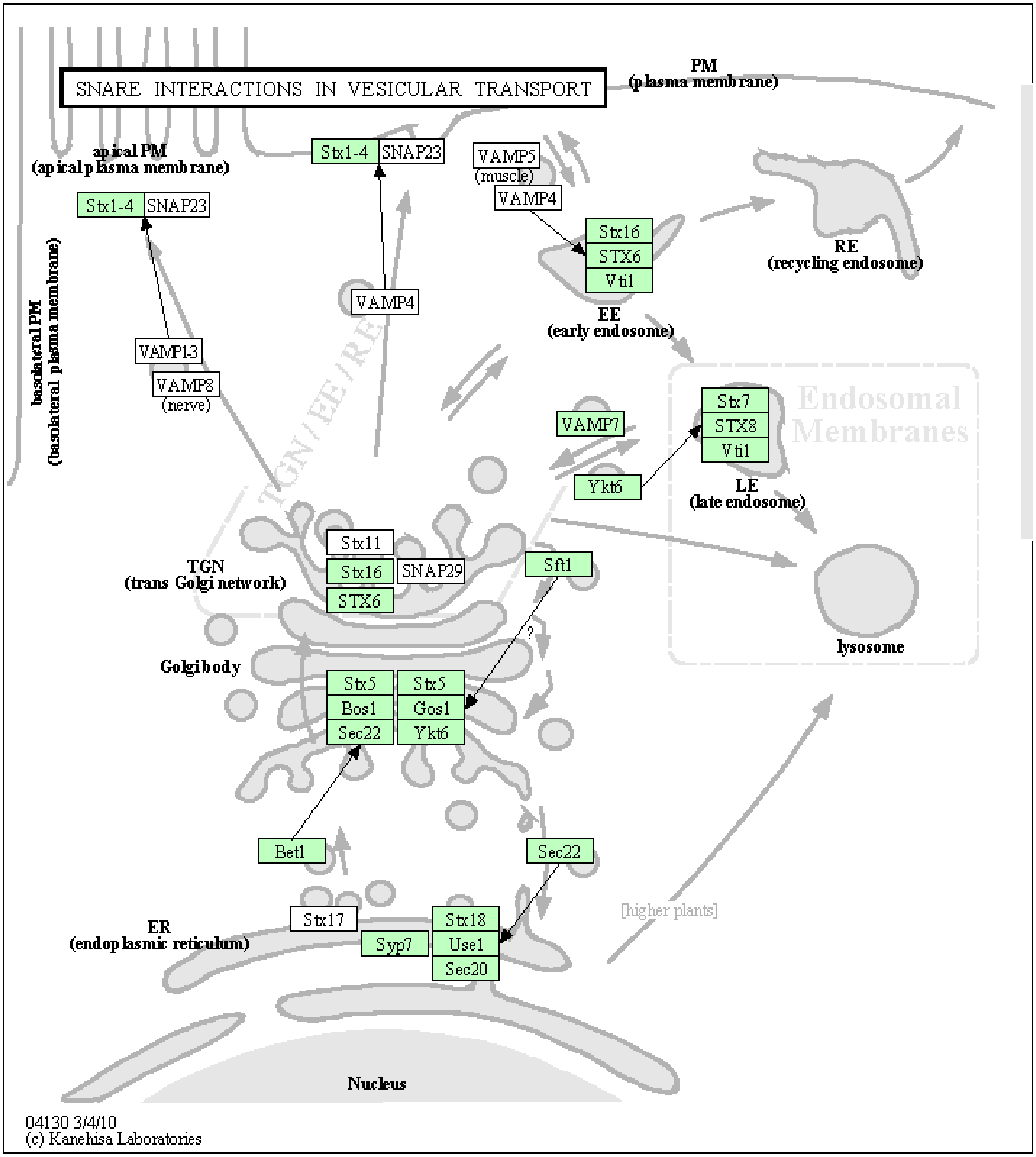

SNARE INTERACTIONS IN VESICULAR TRANSPORT
PM
(plasma membrane)
apical PM
(apical plasma membrane)
basolateral PM
(basolateral plasma membrane)
Stx1-4
SNAP23
Stx1-4
SNAP23
VAMP5
(muscle)
VAMP4
Stx16
STX6
Vti1
RE
(recycling endosome)
EE
(early endosome)
VAMP4
VAMP13
VAMP8
(nerve)
TGN/EE/RE
Endosomal Membranes
VAMP7
Ykt6
Stx7
STX8
Vti1
LE
(late endosome)
Stx11
Stx16
SNAP29
STX6
TGN
(trans Golgi network)
Sft1
lysosome
Golgi body
Stx5
Bos1
Sec22
Stx5
Gos1
Ykt6
?
Bet1
Sec22
[higher plants]
Stx17
Stx18
Use1
Sec20
Syp7
ER
(endoplasmic reticulum)
Nucleus
04130 3/4/10
(c) Kanehisa Laboratories

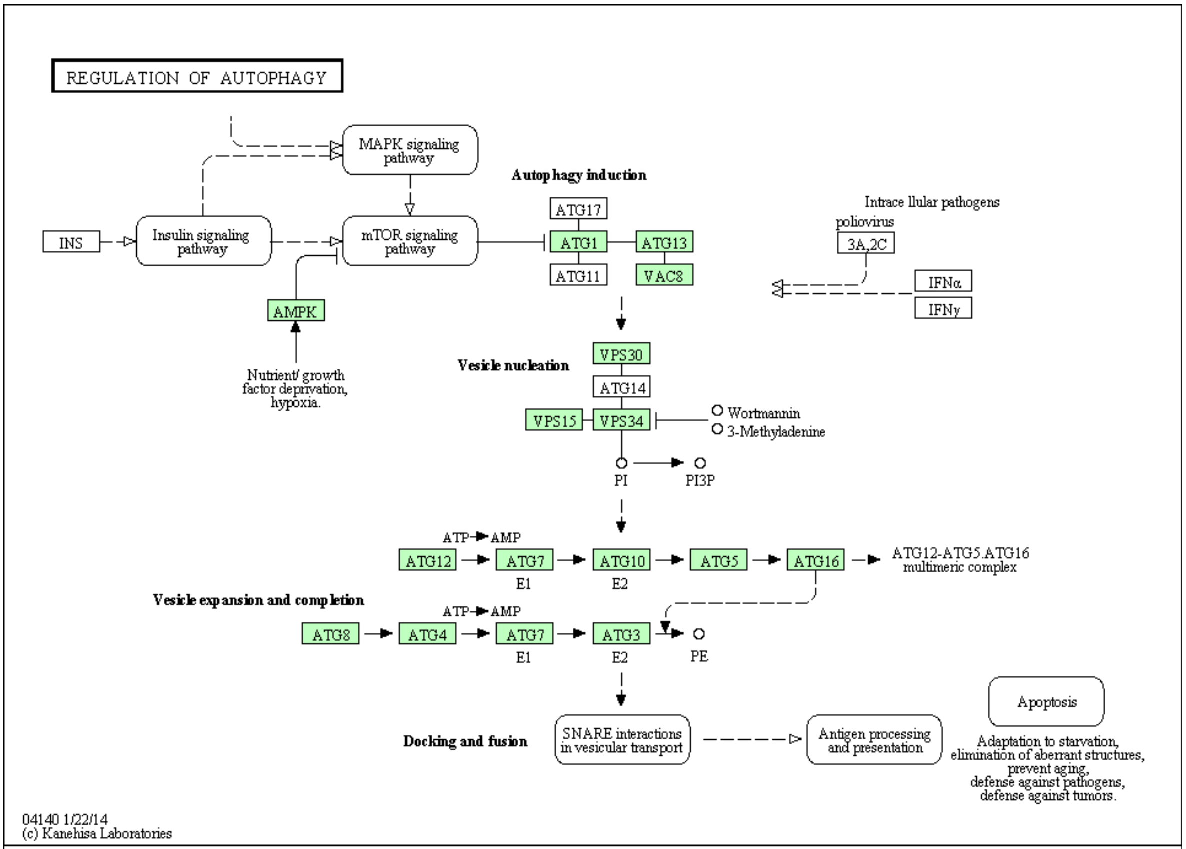
REGULATION OF AUTOPHAGY
MAPK signaling pathway
INS
Insulin signaling pathway
mTOR signaling pathway
AMPK
Nutrient/ growth factor deprivation, hypoxia.
Autophagy induction
ATG17
ATG1
ATG13
ATG11
VAC8
Intracellular pathogens
poliovirus
3A,2C
IFNα
IFNγ
Vesicle nucleation
VPS30
ATG14
VPS15
VPS34
Wortmannin
3-Methyladenine
PI
PI3P
ATP
AMP
ATG12
ATG7
ATG10
ATG5
ATG16
ATG12-ATG5.ATG16 multimeric complex
E1
E2
Vesicle expansion and completion
ATG8
ATG4
ATG7
ATG3
PE
Docking and fusion
SNARE interactions in vesicular transport
Antigen processing and presentation
Apoptosis
Adaptation to starvation, elimination of aberrant structures, prevent aging, defense against pathogens, defense against tumors.
04140 1/22/14
(c) Kanehisa Laboratories

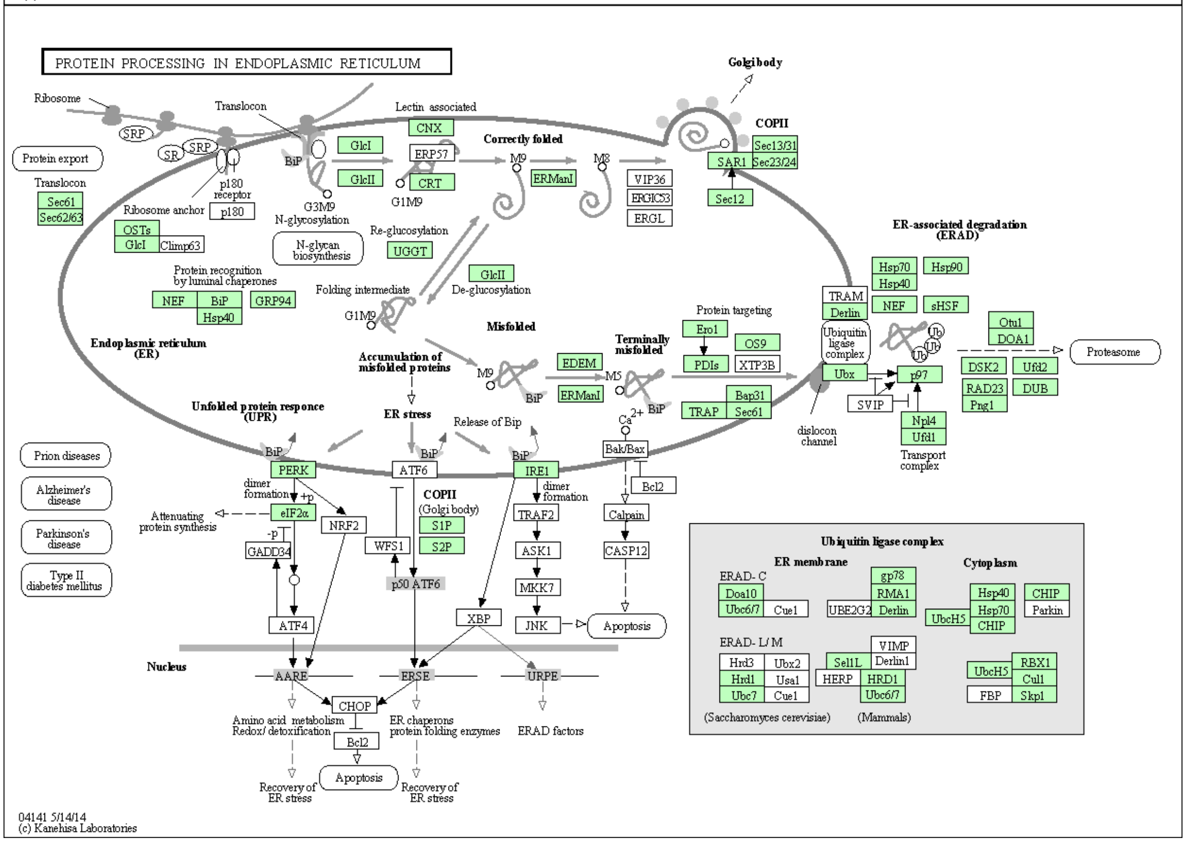
PROTEIN PROCESSING IN ENDOPLASMIC RETICULUM
Ribosome
Translocon
SRP
SR
Protein export
Translocon
Sec61
Sec62/63
p180 receptor
Ribosome anchor
p180
OSTs
GlcI
Climp63
BiP
G3M9
N-glycosylation
N-glycan biosynthesis
Lectin associated
GlcI
GlcII
CNX
ERP57
CRT
G1M9
Correctly folded
M9
M8
ERManI
VIP36
ERGIC53
ERGL
Golgi body
COPII
Sec13/31
SAR1
Sec23/24
Sec12
Re-glucosylation
UGGT
GlcII
De-glucosylation
Protein recognition by luminal chaperones
NEF
BiP
GRP94
Hsp40
Folding intermediate
G1M9
Endoplasmic reticulum (ER)
Misfolded
Accumulation of misfolded proteins
EDEM
ERManI
M5
Terminally misfolded
Protein targeting
Ero1
OS9
PDIs
XTP3B
TRAP
Bap31
Sec61
ER-associated degradation (ERAD)
Hsp70
Hsp90
Hsp40
TRAM
Derlin
NEF
sHSF
Ubiquitin ligase complex
Ub
Otu1
DOA1
Proteasome
Ubx
p97
SVIP
DSK2
Ufd2
RAD23
DUB
Png1
Npl4
Ufd1
Transport complex
dislocon channel
Unfolded protein responce (UPR)
ER stress
Release of Bip
Ca2+
Bak/Bax
Bcl2
PERK
ATF6
IRE1
dimer formation
eIF2α
+p
-p
Attenuating protein synthesis
GADD34
NRF2
COPII (Golgi body)
S1P
S2P
WFS1
p50 ATF6
TRAF2
ASK1
MKK7
JNK
Calpain
CASP12
Apoptosis
ATF4
XBP
Nucleus
AARE
ERSE
UPRE
CHOP
Bcl2
Amino acid metabolism Redox/ detoxification
ER chaperons protein folding enzymes
ERAD factors
Apoptosis
Recovery of ER stress
Recovery of ER stress
Prion diseases
Alzheimer's disease
Parkinson's disease
Type II diabetes mellitus
Ubiquitin ligase complex
ER membrane
Cytoplasm
ERAD- C
Doa10
Ubc6/7
Cue1
gp78
RMA1
UBE2G2
Derlin
Hsp40
CHIP
UbcH5
Hsp70
Parkin
CHIP
ERAD- L/ M
VIMP
Hrd3
Ubx2
Sel1L
Derlin1
Hrd1
Usa1
HERP
HRD1
UbcH5
RBX1
Cul1
Ubc7
Cue1
Ubc6/7
FBP
Skp1
(Saccharomyces cerevisiae)
(Mammals)
04141 5/14/14
(c) Kanehisa Laboratories

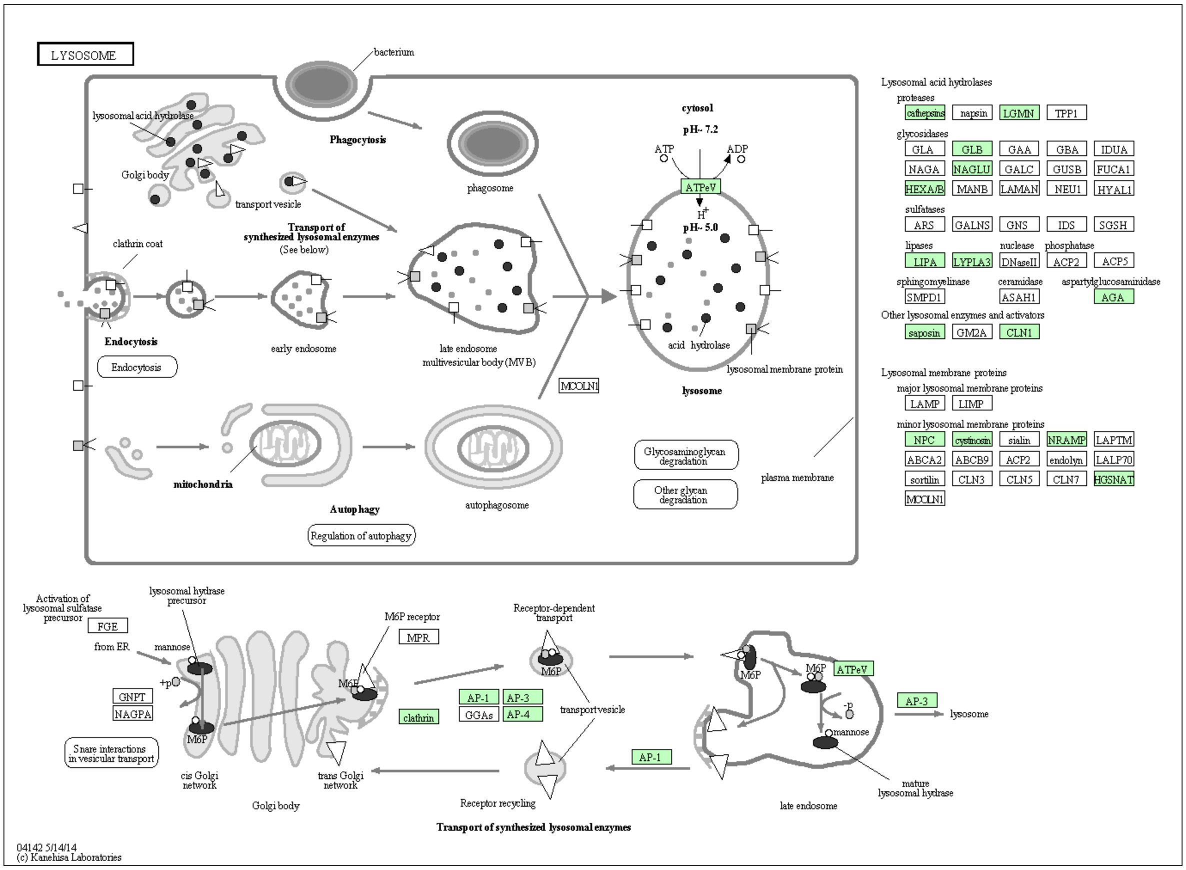
LYSOSOME
bacterium
lysosomal acid hydrolase
Golgi body
transport vesicle
Phagocytosis
phagosome
cytosol
pH~ 7.2
ATP
ADP
ATPeV
H+
pH~ 5.0
clathrin coat
Transport of
synthesized lysosomal enzymes
(See below)
Endocytosis
Endocytosis
early endosome
late endosome
multivesicular body (MVB)
MCOLN1
acid hydrolase
lysosomal membrane protein
lysosome
mitochondria
Autophagy
Regulation of autophagy
autophagosome
Glycosaminoglycan degradation
Other glycan degradation
plasma membrane
Lysosomal acid hydrolases
proteases
cathepsins
napsin
LGMN
TPP1
glycosidases
GLA
GLB
GAA
GBA
IDUA
NAGA
NAGLU
GALC
GUSB
FUCA1
HEXA/B
MANB
LAMAN
NEU1
HYAL1
sulfatases
ARS
GALNS
GNS
IDS
SGSH
lipases
LIPA
LYPLA3
nuclease
DNaseII
phosphatase
ACP2
ACP5
sphingomyelinase
SMPD1
ceramidase
ASAH1
aspartylglucosaminidase
AGA
Other lysosomal enzymes and activators
saposin
GM2A
CLN1
Lysosomal membrane proteins
major lysosomal membrane proteins
LAMP
LIMP
minor lysosomal membrane proteins
NPC
cystinosin
sialin
NRAMP
LAPTM
ABCA2
ABCB9
ACP2
endolyn
LALP70
sortilin
CLN3
CLN5
CLN7
HGSNAT
MCOLN1
Activation of lysosomal sulfatase precursor
FGE
lysosomal hydrase precursor
from ER
mannose
+p
GNPT
NAGPA
M6P
Snare interactions in vesicular transport
cis Golgi network
Golgi body
M6P receptor
MPR
M6P
clathrin
trans Golgi network
Receptor-dependent transport
M6P
AP-1
AP-3
GGAs
AP-4
transport vesicle
Receptor recycling
AP-1
M6P
M6P
ATPeV
-p
mannose
AP-3
lysosome
late endosome
mature lysosomal hydrase
Transport of synthesized lysosomal enzymes
04142 5/14/14
(c) Kanehisa Laboratories

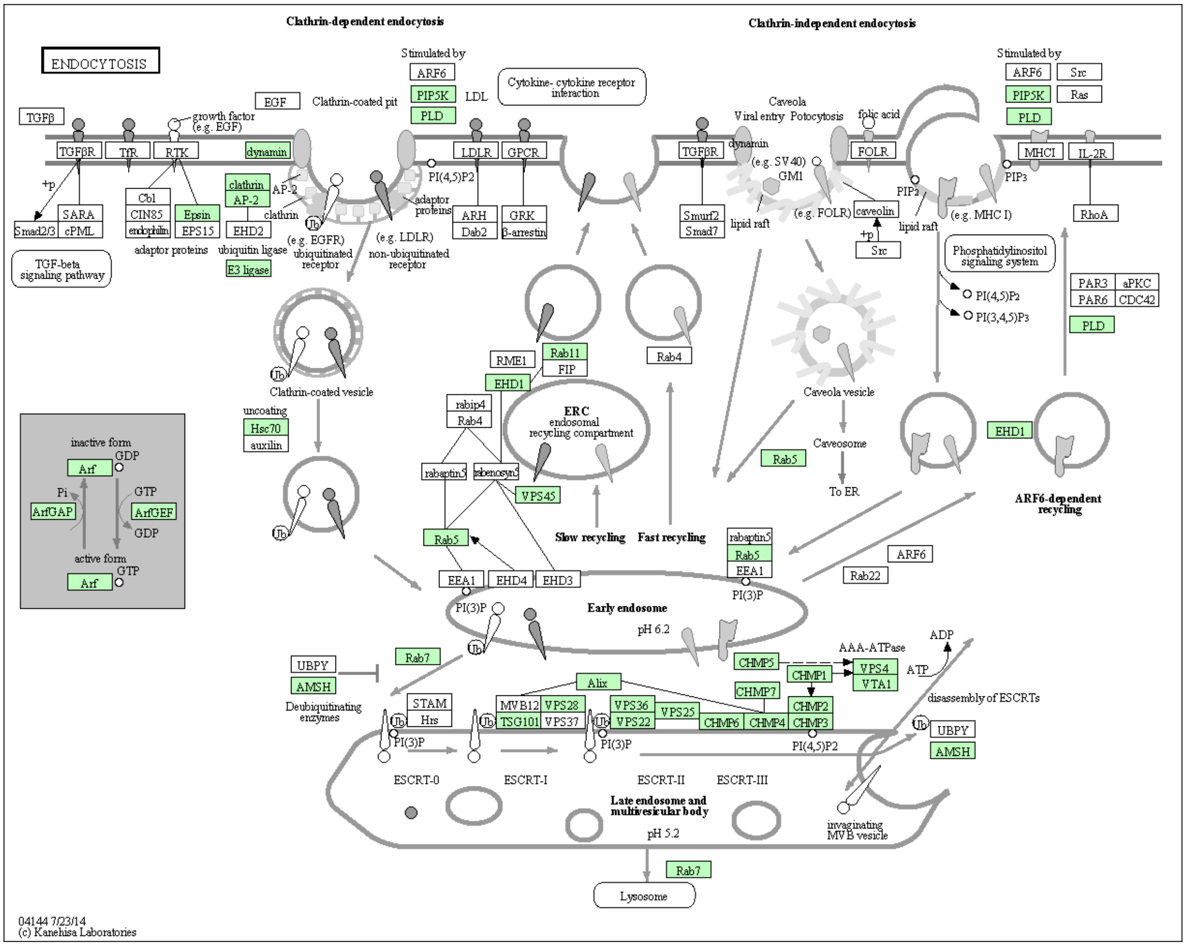
Clathrin-dependent endocytosis
Clathrin-independent endocytosis
ENDOCYTOSIS
Stimulated by
ARF6
PIP5K
PLD
Cytokine- cytokine receptor interaction
Stimulated by
ARF6
Src
PIP5K
Ras
PLD
TGFβ
EGF
Clathrin-coated pit
LDL
growth factor (e.g. EGF)
Caveola
Viral entry
Potocytosis
folic acid
TGFβR
TfR
RTK
dynamin
LDLR
GPCR
TGFβR
dynamin
FOLR
MHCI
IL-2R
(e.g. SV40)
GM1
PI(4,5)P2
PIP2
PIP3
+p
clathrin
AP-2
AP-2
Cbl
CIN85
endophilin
Epsin
EPS15
EHD2
clathrin
adaptor proteins
ARH
Dab2
GRK
β-arrestin
SARA
Smad2/3
cPML
Smurf2
Smad7
lipid raft
(e.g. FOLR)
caveolin
lipid raft
(e.g. MHC I)
RhoA
adaptor proteins
ubiquitin ligase
E3 ligase
(e.g. EGFR) ubiquitinated receptor
(e.g. LDLR) non-ubiquitinated receptor
+p
Src
Phosphatidylinositol signaling system
PI(4,5)P2
PI(3,4,5)P3
PAR3
aPKC
PAR6
CDC42
PLD
TGF-beta signaling pathway
Clathrin-coated vesicle
Rab4
RME1
Rab11
FIP
EHD1
rabip4
Rab4
ERC
endosomal recycling compartment
Caveola vesicle
Caveosome
Rab5
To ER
EHD1
ARF6-dependent recycling
uncoating
Hsc70
auxilin
inactive form
GDP
Arf
Pi
GTP
ArfGAP
ArfGEF
GDP
active form
GTP
Arf
rabaptin5
rabenosyn5
VPS45
Rab5
Slow recycling
Fast recycling
rabaptin5
Rab5
EEA1
ARF6
Rab22
EEA1
EHD4
EHD3
PI(3)P
PI(3)P
Early endosome
pH 6.2
UBPY
AMSH
Deubiquitinating enzymes
Rab7
Alix
CHMP5
CHMP1
VPS4
VTA1
AAA-ATPase
ADP
ATP
CHMP7
disassembly of ESCRTs
STAM
Hrs
MVB12
VPS28
TSG101
VPS37
VPS36
VPS22
VPS25
CHMP6
CHMP4
CHMP2
CHMP3
UBPY
AMSH
PI(3)P
PI(3)P
PI(4,5)P2
ESCRT-0
ESCRT-I
ESCRT-II
ESCRT-III
Late endosome and multivesicular body
pH 5.2
invaginating MVB vesicle
Rab7
Lysosome
04144 7/23/14
(c) Kanehisa Laboratories

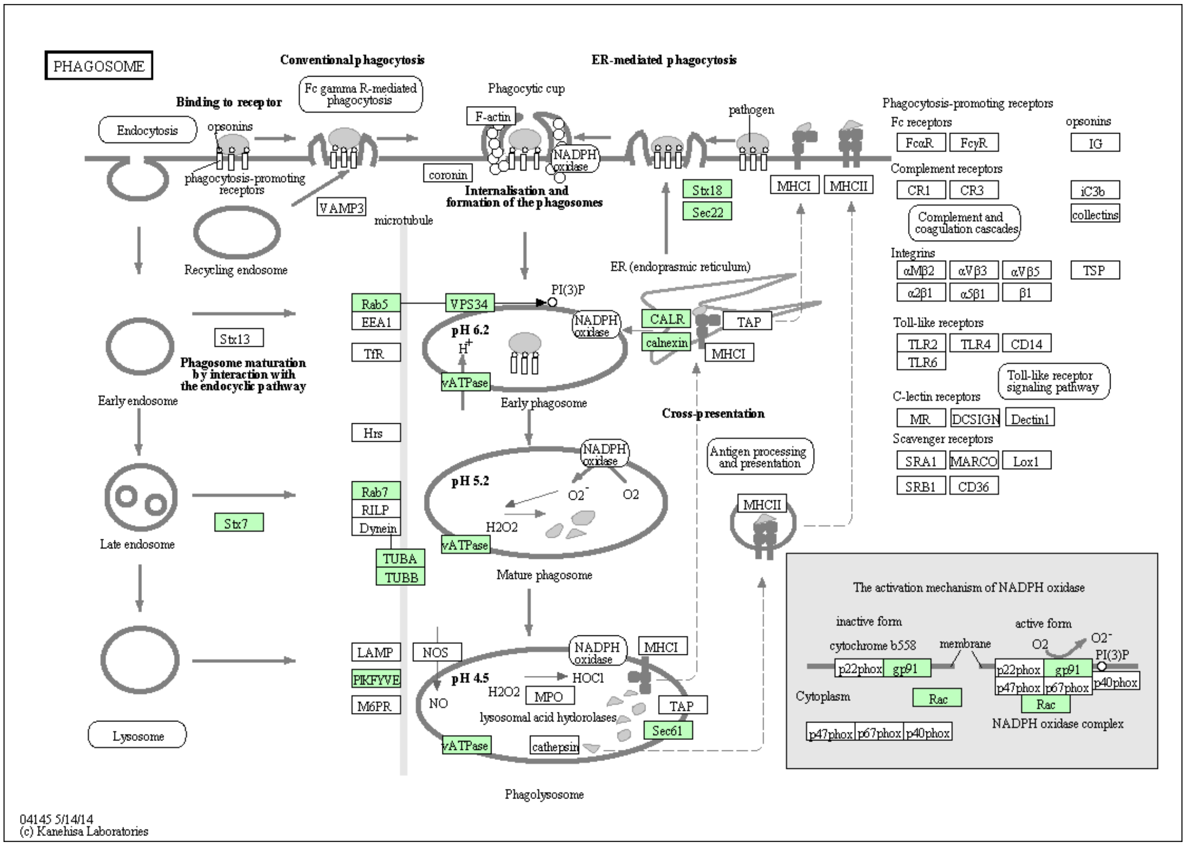
PHAGOSOME
Conventional phagocytosis
ER-mediated phagocytosis
Binding to receptor
Fc gamma R-mediated phagocytosis
Phagocytic cup
pathogen
Phagocytosis-promoting receptors
Fc receptors
opsonins
FcαR
FcγR
IG
Endocytosis
opsonins
F-actin
NADPH oxidase
phagocytosis-promoting receptors
coronin
Internalisation and formation of the phagosomes
Stx18
Sec22
MHCI
MHCII
Complement receptors
CR1
CR3
iC3b
collectins
Complement and coagulation cascades
VAMP3
microtubule
Recycling endosome
ER (endoplasmic reticulum)
Integrins
αMβ2
αVβ3
αVβ5
TSP
α2β1
α5β1
β1
PI(3)P
Rab5
VPS34
EEA1
Stx13
pH 6.2
H+
NADPH oxidase
CALR
calnexin
TAP
MHCI
Toll-like receptors
TLR2
TLR4
CD14
TLR6
Toll-like receptor signaling pathway
TfR
vATPase
Phagosome maturation by interaction with the endocytic pathway
Early endosome
Early phagosome
C-lectin receptors
MR
DCSIGN
Dectin1
Cross-presentation
Scavenger receptors
SRA1
MARCO
Lox1
SRB1
CD36
Hrs
Antigen processing and presentation
pH 5.2
NADPH oxidase
O2-
O2
Rab7
RILP
Dynein
Stx7
H2O2
MHCII
Late endosome
vATPase
TUBA
TUBB
Mature phagosome
The activation mechanism of NADPH oxidase
inactive form
active form
cytochrome b558
membrane
O2
O2-
PI(3)P
p22phox
gp91
p47phox
p67phox
p40phox
Cytoplasm
Rac
NADPH oxidase complex
LAMP
NOS
NADPH oxidase
MHCI
PIKFYVE
pH 4.5
H2O2
HOCl
MPO
NO
M6PR
TAP
lysosomal acid hydrolases
Sec61
Lysosome
vATPase
cathepsin
Phagolysosome
04145 5/14/14
(c) Kanehisa Laboratories

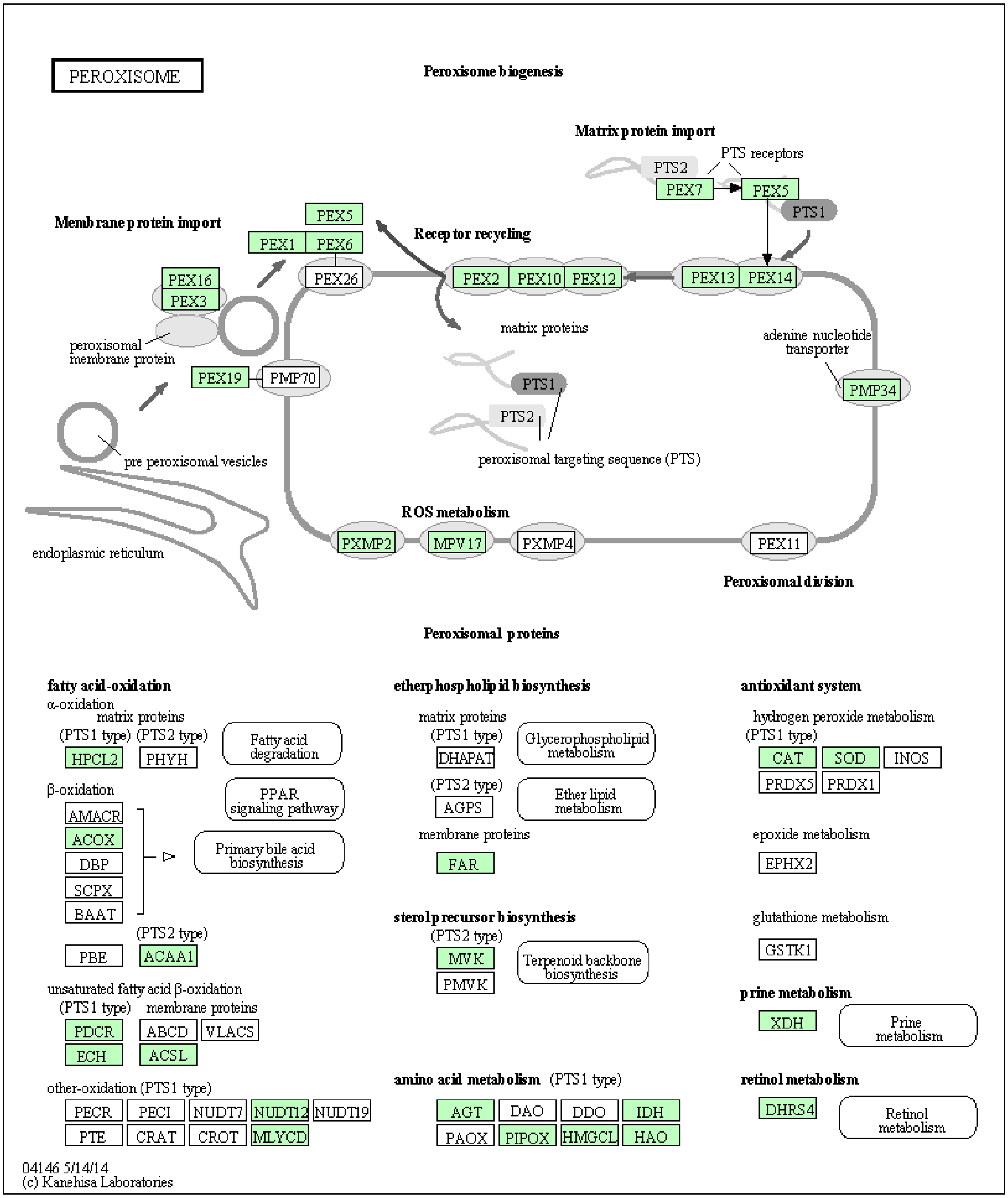
PEROXISOME
Peroxisome biogenesis
Matrix protein import
PTS2
PTS receptors
PEX7
PEX5
PTS1
Membrane protein import
PEX5
PEX1
PEX6
Receptor recycling
PEX16
PEX3
PEX26
PEX2
PEX10
PEX12
PEX13
PEX14
matrix proteins
adenine nucleotide transporter
peroxisomal membrane protein
PEX19
PMP70
PTS1
PMP34
PTS2
peroxisomal targeting sequence (PTS)
pre peroxisomal vesicles
ROS metabolism
endoplasmic reticulum
PXMP2
MPV17
PXMP4
PEX11
Peroxisomal division
Peroxisomal proteins
fatty acid-oxidation
α-oxidation
matrix proteins
(PTS1 type) (PTS2 type)
HPCL2
PHYH
Fatty acid degradation
β-oxidation
PPAR signaling pathway
AMACR
ACOX
DBP
SCPX
BAAT
Primary bile acid biosynthesis
(PTS2 type)
PBE
ACAA1
unsaturated fatty acid β-oxidation
(PTS1 type) membrane proteins
PDCR
ABCD
VLACS
ECH
ACSL
other-oxidation (PTS1 type)
PECR
PECI
NUDT7
NUDT12
NUDT19
PTE
CRAT
CROT
MLYCD
ether phospholipid biosynthesis
matrix proteins
(PTS1 type)
DHAPAT
Glycerophospholipid metabolism
(PTS2 type)
AGPS
Ether lipid metabolism
membrane proteins
FAR
sterol precursor biosynthesis
(PTS2 type)
MVK
PMVK
Terpenoid backbone biosynthesis
amino acid metabolism (PTS1 type)
AGT
DAO
DDO
IDH
PAOX
PIPOX
HMGCL
HAO
antioxidant system
hydrogen peroxide metabolism
(PTS1 type)
CAT
SOD
INOS
PRDX5
PRDX1
epoxide metabolism
EPHX2
glutathione metabolism
GSTK1
prine metabolism
XDH
Prine metabolism
retinol metabolism
DHRS4
Retinol metabolism
04146 5/14/14
(c) Kanehisa Laboratories

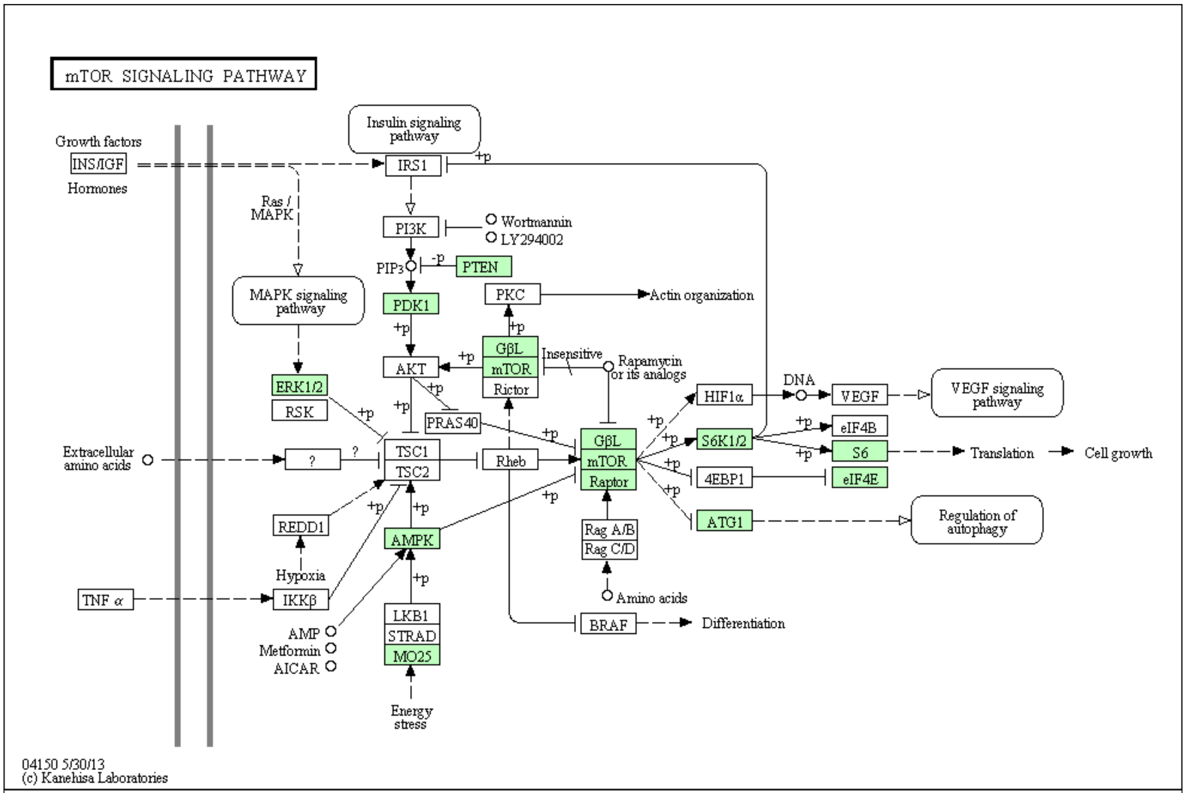
mTOR SIGNALING PATHWAY
Growth factors
INS/IGF
Hormones
Insulin signaling pathway
IRS1
+p
Ras / MAPK
PI3K
Wortmannin
LY294002
PIP3
-p
PTEN
MAPK signaling pathway
PDK1
PKC
Actin organization
ERK1/2
RSK
AKT
GβL
mTOR
Rictor
Insensitive
Rapamycin or its analogs
PRAS40
HIF1α
DNA
VEGF
VEGF signaling pathway
Extracellular amino acids
?
TSC1
TSC2
Rheb
GβL
mTOR
Raptor
S6K1/2
eIF4B
S6
Translation
Cell growth
4EBP1
eIF4E
REDD1
AMPK
ATG1
Regulation of autophagy
Hypoxia
Rag A/B
Rag C/D
TNF α
IKKβ
LKB1
STRAD
MO25
Amino acids
AMP
Metformin
AICAR
BRAF
Differentiation
Energy stress
04150 5/30/13
(c) Kanehisa Laboratories

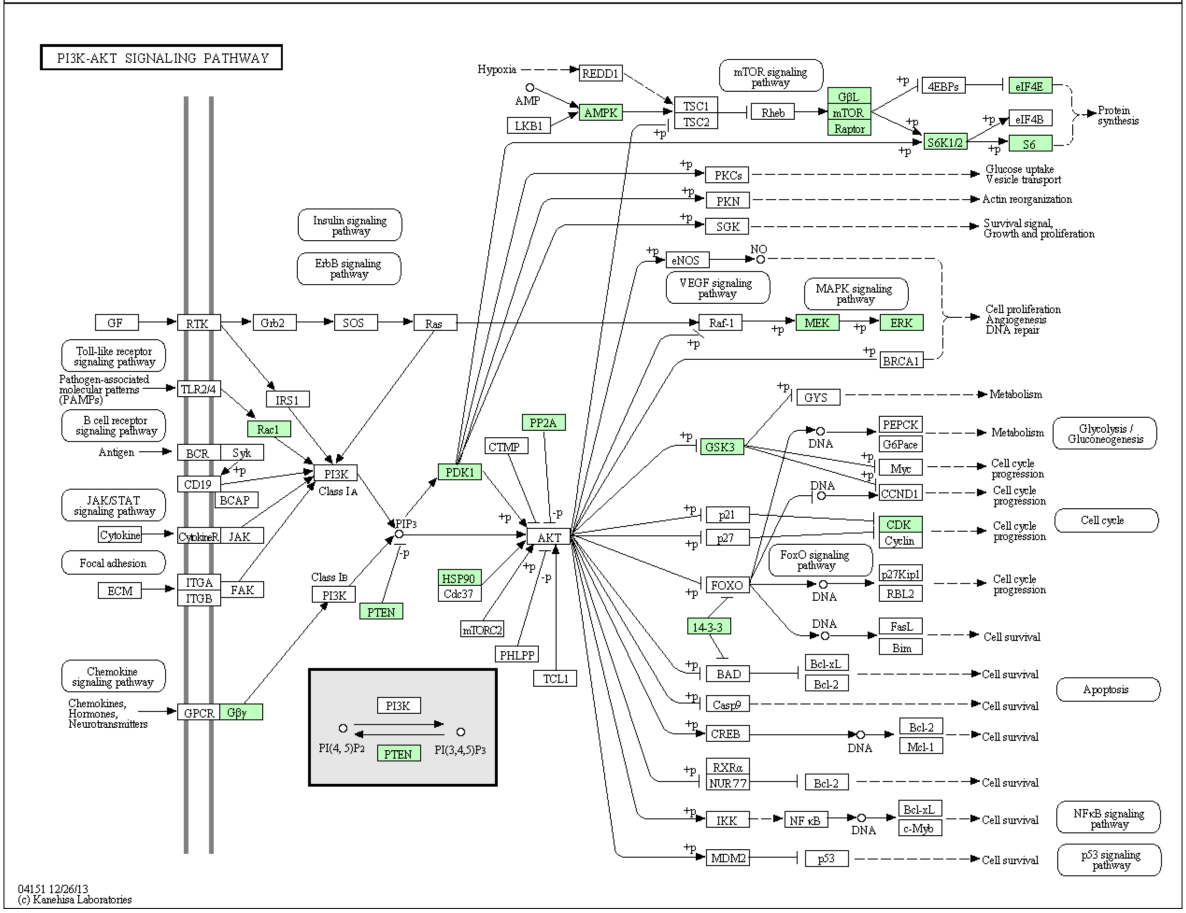
PI3K-AKT SIGNALING PATHWAY
Hypoxia
REDD1
AMP
AMPK
LKB1
TSC1
TSC2
Rheb
mTOR signaling pathway
GβL
mTOR
Raptor
4EBPs
eIF4E
eIF4B
S6K1/2
S6
Protein synthesis
PKCs
Glucose uptake
Vesicle transport
PKN
Actin reorganization
SGK
Survival signal, Growth and proliferation
Insulin signaling pathway
ErbB signaling pathway
eNOS
NO
VEGF signaling pathway
MAPK signaling pathway
GF
RTK
Grb2
SOS
Ras
Raf-1
MEK
ERK
Cell proliferation
Angiogenesis
DNA repair
Toll-like receptor signaling pathway
Pathogen-associated molecular patterns (PAMPs)
TLR2/4
IRS1
BRCA1
GYS
Metabolism
B cell receptor signaling pathway
Rac1
PP2A
Antigen
BCR
Syk
PI3K
Class IA
CTMP
GSK3
PEPCK
G6Pace
Metabolism
Glycolysis / Gluconeogenesis
CD19
BCAP
PDK1
Myc
Cell cycle progression
JAK/STAT signaling pathway
CCND1
Cell cycle progression
Cytokine
CytokineR
JAK
PIP3
AKT
p21
p27
CDK
Cyclin
Cell cycle progression
Cell cycle
Focal adhesion
HSP90
Cdc37
FoxO signaling pathway
ECM
ITGA
ITGB
FAK
Class IB
PI3K
PTEN
FOXO
DNA
p27Kip1
RBL2
Cell cycle progression
mTORC2
14-3-3
FasL
Bim
Cell survival
PHLPP
Chemokine signaling pathway
TCL1
BAD
Bcl-xL
Bcl-2
Cell survival
Apoptosis
Chemokines, Hormones, Neurotransmitters
GPCR
Gβγ
PI3K
PI(4, 5)P2
PTEN
PI(3,4,5)P3
Casp9
Cell survival
CREB
Bcl-2
Mcl-1
Cell survival
RXRα
NUR77
Bcl-2
Cell survival
IKK
NF-κB
Bcl-xL
c-Myb
Cell survival
NFκB signaling pathway
MDM2
p53
Cell survival
p53 signaling pathway
04151 12/26/13
(c) Kanehisa Laboratories

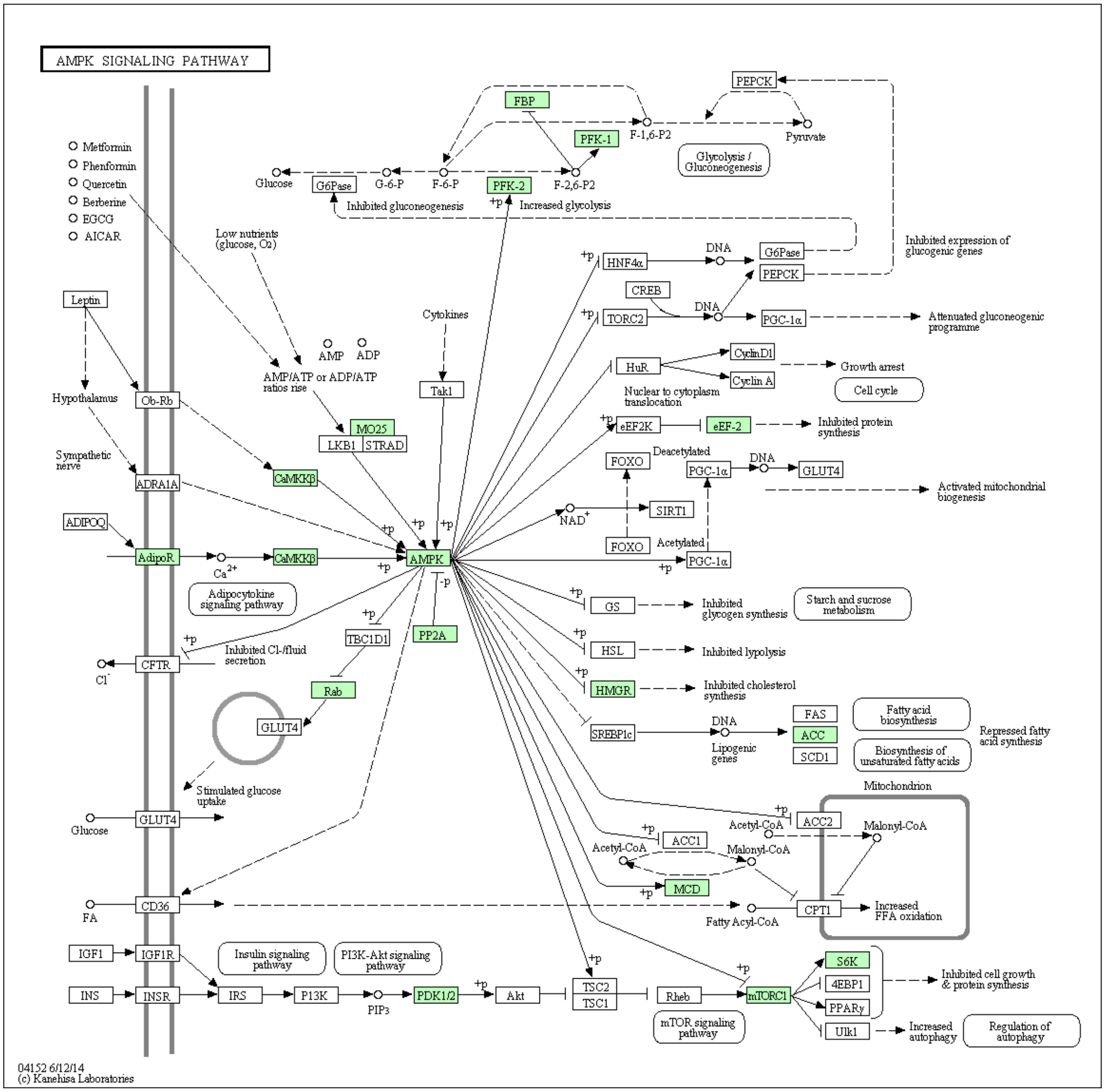
AMPK SIGNALING PATHWAY
Metformin
Phenformin
Quercetin
Berberine
EGCG
AICAR
Low nutrients (glucose, O2)
Leptin
Hypothalamus
Ob-Rb
Sympathetic nerve
ADRA1A
ADIPOQ
AdipoR
Ca2+
CaMKKβ
AMP
ADP
AMP/ATP or ADP/ATP ratios rise
MO25
LKB1
STRAD
Cytokines
Tak1
AMPK
PP2A
TBC1D1
Rab
GLUT4
Adipocytokine signaling pathway
CFTR
Cl-
Inhibited Cl-/fluid secretion
Stimulated glucose uptake
Glucose
FA
CD36
IGF1
IGF1R
INS
INSR
IRS
PI3K
PIP3
PDK1/2
Akt
Insulin signaling pathway
PI3K-Akt signaling pathway
TSC2
TSC1
Rheb
mTORC1
mTOR signaling pathway
S6K
4EBP1
PPARγ
Ulk1
Inhibited cell growth & protein synthesis
Increased autophagy
Regulation of autophagy
FBP
PFK-1
PFK-2
F-1,6-P2
F-2,6-P2
F-6-P
G-6-P
G6Pase
Pyruvate
PEPCK
Glycolysis / Gluconeogenesis
Inhibited gluconeogenesis
Increased glycolysis
HNF4α
DNA
CREB
TORC2
PGC-1α
Attenuated gluconeogenic programme
Inhibited expression of glucogenic genes
HuR
CyclinD1
Cyclin A
Growth arrest
Cell cycle
Nuclear to cytoplasm translocation
eEF2K
eEF-2
Inhibited protein synthesis
FOXO
Deacetylated
Acetylated
GLUT4
Activated mitochondrial biogenesis
NAD+
SIRT1
GS
Inhibited glycogen synthesis
Starch and sucrose metabolism
HSL
Inhibited lypolysis
HMGR
Inhibited cholesterol synthesis
SREBP1c
Lipogenic genes
FAS
ACC
SCD1
Fatty acid biosynthesis
Biosynthesis of unsaturated fatty acids
Repressed fatty acid synthesis
Mitochondrion
ACC2
Acetyl-CoA
Malonyl-CoA
ACC1
MCD
Fatty Acyl-CoA
CPT1
Increased FFA oxidation
+p
-p
04152 6/12/14
(c) Kanehisa Laboratories

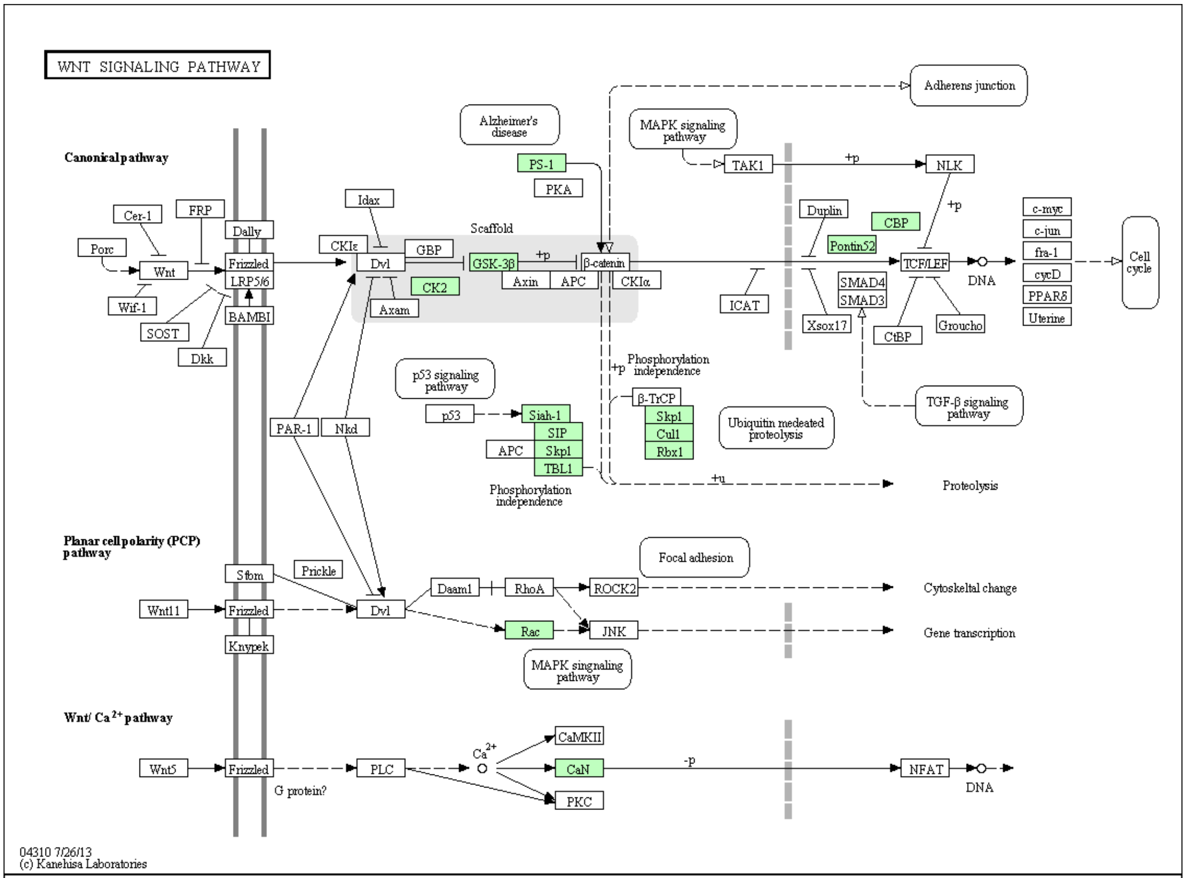
WNT SIGNALING PATHWAY
Canonical pathway
Cer-1
FRP
Dally
Porc
Wnt
Frizzled
LRP5/6
Wif-1
SOST
Dkk
BAMBI
Idax
CKIε
Dvl
GBP
GSK-3β
CK2
Axam
Scaffold
+p
Axin
APC
β-catenin
CKIα
Alzheimer's disease
PS-1
PKA
Adherens junction
MAPK signaling pathway
TAK1
+p
NLK
Duplin
CBP
Pontin52
+p
TCF/LEF
DNA
c-myc
c-jun
fra-1
cycD
PPARδ
Uterine
Cell cycle
ICAT
SMAD4
SMAD3
Xsox17
CtBP
Groucho
TGF-β signaling pathway
p53 signaling pathway
p53
Siah-1
SIP
Skp1
TBL1
APC
Phosphorylation independence
+p
Phosphorylation independence
β-TrCP
Skp1
Cul1
Rbx1
Ubiquitin medeated proteolysis
+u
Proteolysis
PAR-1
Nkd
Planar cell polarity (PCP) pathway
Stbm
Prickle
Wnt11
Frizzled
Knypek
Dvl
Daam1
RhoA
ROCK2
Focal adhesion
Cytoskeltal change
Rac
JNK
Gene transcription
MAPK singnaling pathway
Wnt/ Ca2+ pathway
Wnt5
Frizzled
G protein?
PLC
Ca2+
CaMKII
CaN
-p
NFAT
DNA
PKC
04310 7/26/13
(c) Kanehisa Laboratories

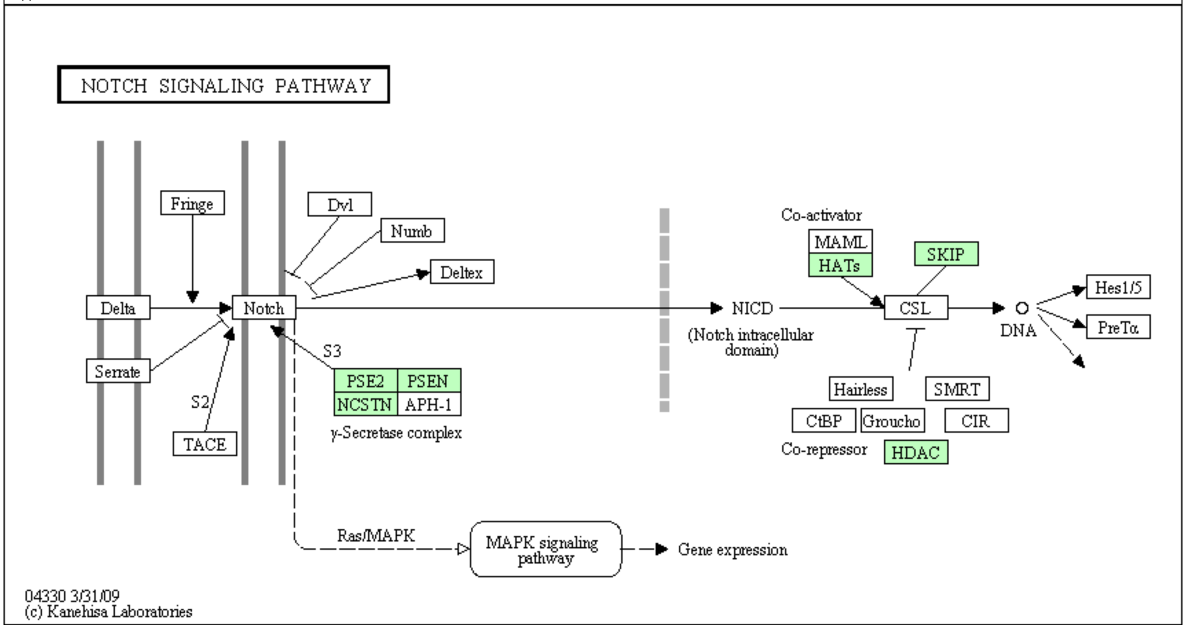
NOTCH SIGNALING PATHWAY
Fringe
Dvl
Numb
Deltex
Delta
Notch
Serrate
S2
TACE
S3
PSE2
PSEN
NCSTN
APH-1
γ-Secretase complex
NICD
(Notch intracellular domain)
Co-activator
MAML
HATs
SKIP
CSL
DNA
Hes1/5
PreTα
Hairless
SMRT
CtBP
Groucho
CIR
Co-repressor
HDAC
Ras/MAPK
MAPK signaling pathway
Gene expression
04330 3/31/09
(c) Kanehisa Laboratories

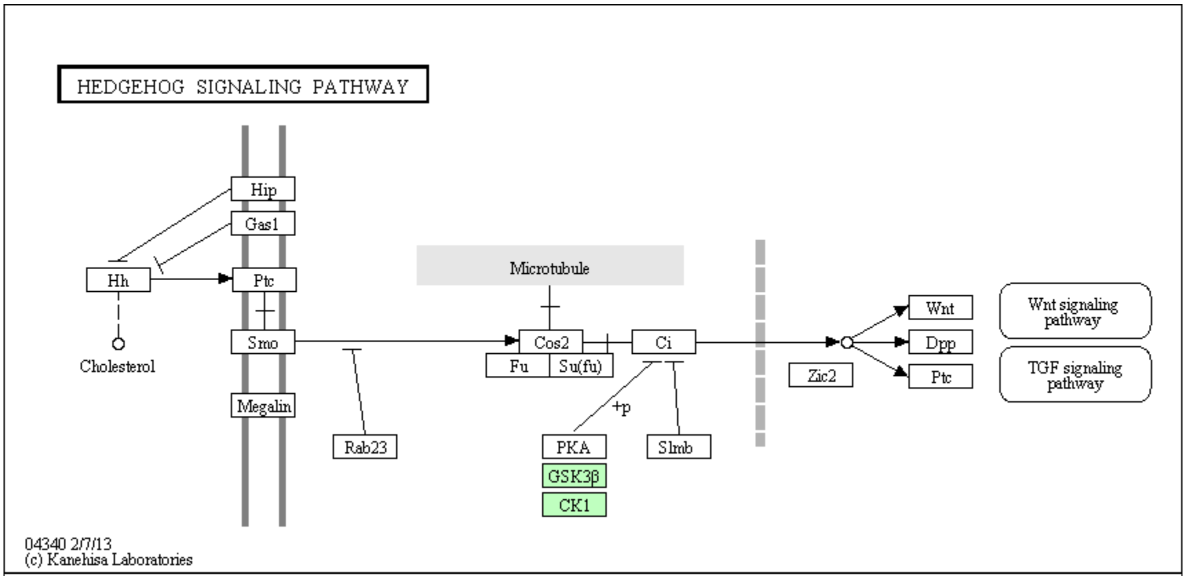
HEDGEHOG SIGNALING PATHWAY
Hip
Gas1
Hh
Ptc
Smo
Megalin
Cholesterol
Microtubule
Cos2
Fu
Su(fu)
Ci
Rab23
+p
PKA
GSK3β
CK1
Slmb
Zic2
Wnt
Dpp
Ptc
Wnt signaling pathway
TGF signaling pathway
04340 2/7/13
(c) Kanehisa Laboratories

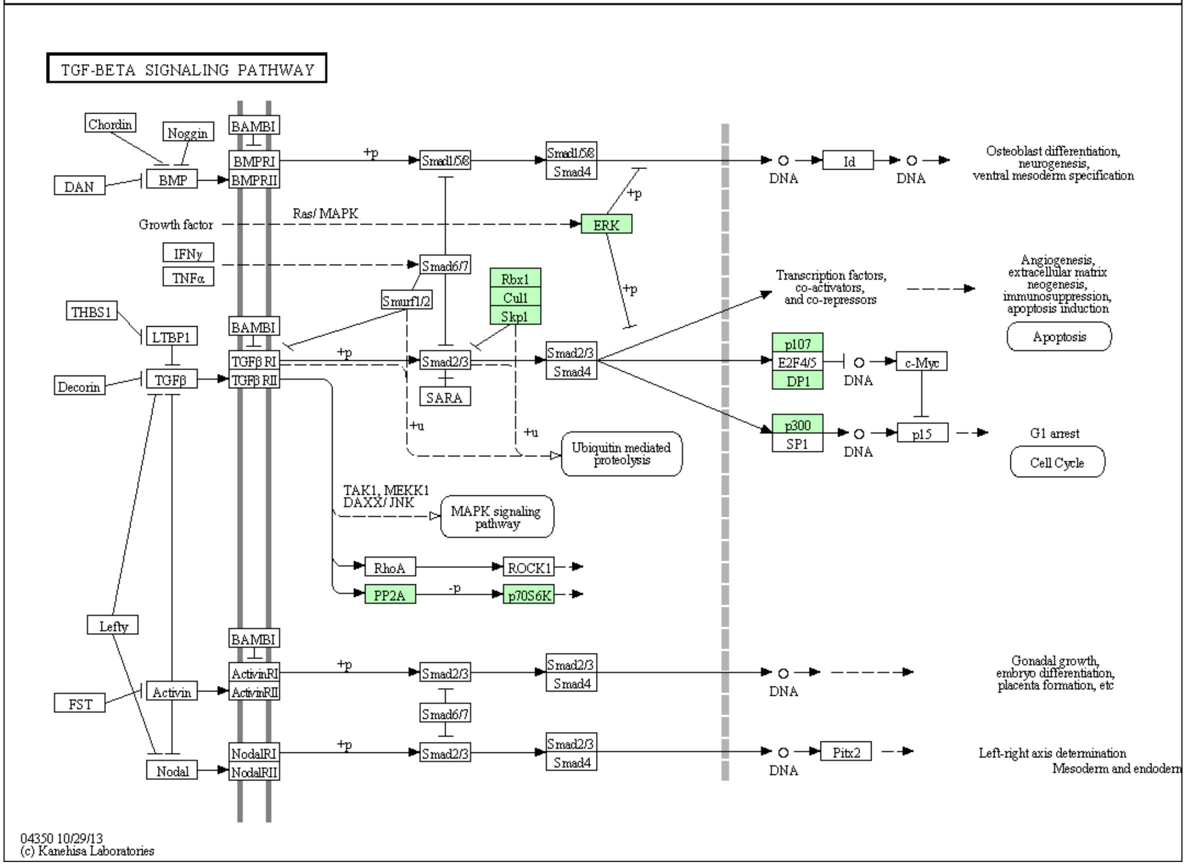
TGF-BETA SIGNALING PATHWAY
Chordin
Noggin
DAN
BMP
BAMBI
BMPRI
BMPRII
+p
Smad1/5/8
Smad1/5/8
Smad4
DNA
Id
DNA
Osteoblast differentiation, neurogenesis, ventral mesoderm specification
+p
Growth factor
Ras/ MAPK
ERK
IFNγ
TNFα
Smad6/7
Rbx1
Cul1
Skp1
Smurf1/2
+p
Transcription factors, co-activators, and co-repressors
Angiogenesis, extracellular matrix neogenesis, immunosuppression, apoptosis induction
Apoptosis
THBS1
LTBP1
BAMBI
Decorin
TGFβ
TGFβ RI
TGFβ RII
+p
Smad2/3
SARA
Smad2/3
Smad4
p107
E2F4/5
DP1
DNA
c-Myc
p300
SP1
DNA
p15
G1 arrest
Cell Cycle
+u
+u
Ubiquitin mediated proteolysis
TAK1, MEKK1
DAXX/ JNK
MAPK signaling pathway
RhoA
ROCK1
PP2A
-p
p70S6K
Lefty
BAMBI
ActivinRI
ActivinRII
+p
Smad2/3
Smad2/3
Smad4
DNA
Gonadal growth, embryo differentiation, placenta formation, etc
FST
Activin
Smad6/7
Nodal
NodalRI
NodalRII
+p
Smad2/3
Smad2/3
Smad4
DNA
Pitx2
Left-right axis determination
Mesoderm and endoderm
04350 10/29/13
(c) Kanehisa Laboratories

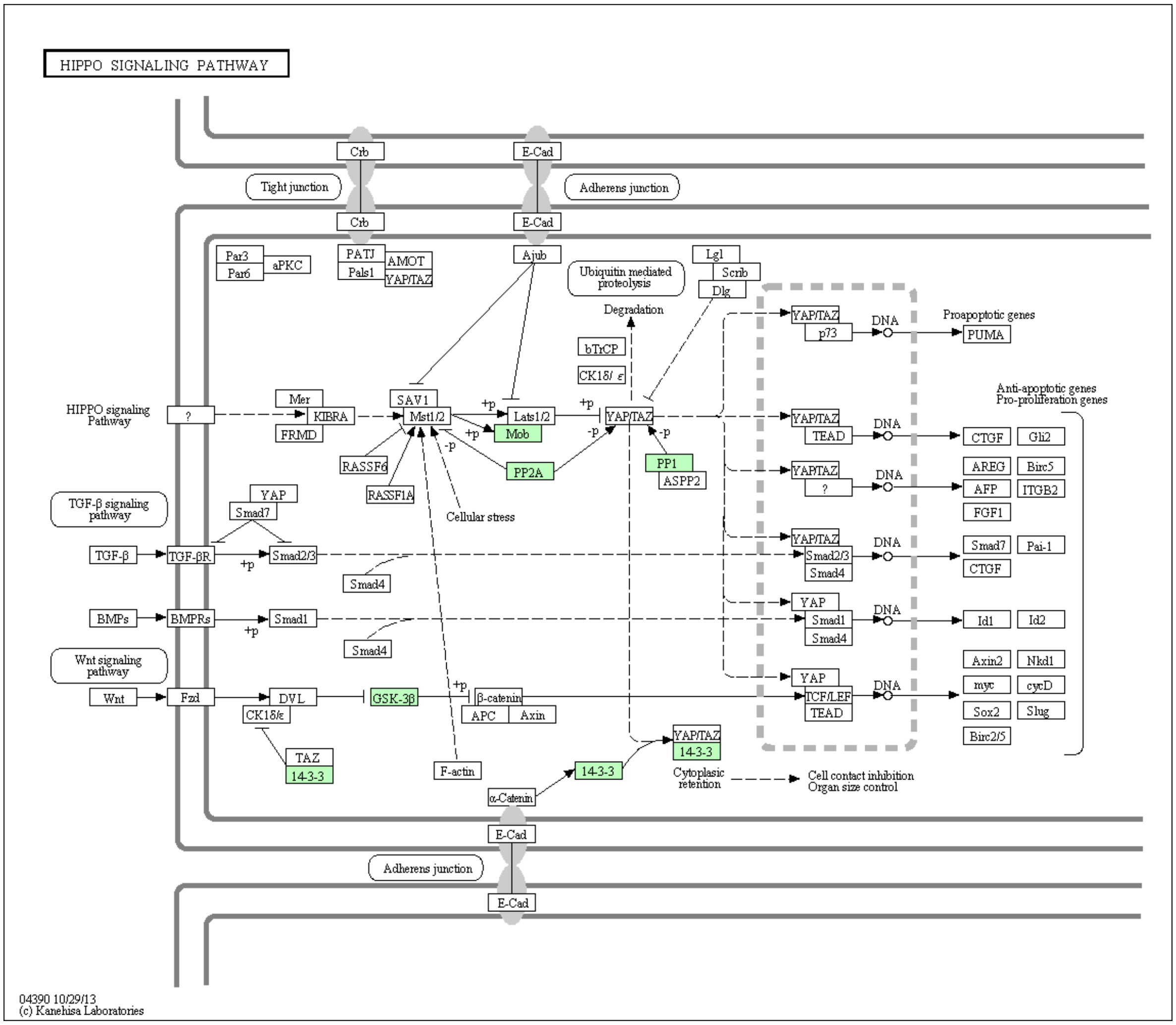
HIPPO SIGNALING PATHWAY
Crb
E-Cad
Tight junction
Adherens junction
Par3
Par6
aPKC
PATJ
Pals1
AMOT
YAP/TAZ
Ajub
Ubiquitin mediated proteolysis
Lgl
Scrib
Dlg
Degradation
bTrCP
CK1δ/ε
YAP/TAZ
p73
DNA
Proapoptotic genes
PUMA
HIPPO signaling Pathway
?
Mer
KIBRA
FRMD
SAV1
Mst1/2
+p
Lats1/2
Mob
PP2A
-p
YAP/TAZ
PP1
ASPP2
RASSF6
RASSF1A
Cellular stress
TEAD
CTGF
Gli2
Anti-apoptotic genes
Pro-proliferation genes
AREG
Birc5
AFP
ITGB2
FGF1
TGF-β signaling pathway
YAP
Smad7
TGF-β
TGF-βR
Smad2/3
Smad4
Pai-1
BMPs
BMPRs
Smad1
Id1
Id2
Wnt signaling pathway
Wnt
Fzd
DVL
CK1δ/ε
GSK-3β
β-catenin
APC
Axin
TCF/LEF
Axin2
Nkd1
myc
cycD
Sox2
Slug
Birc2/5
TAZ
14-3-3
F-actin
α-Catenin
Cytoplasic retention
Cell contact inhibition
Organ size control
04390 10/29/13
(c) Kanehisa Laboratories

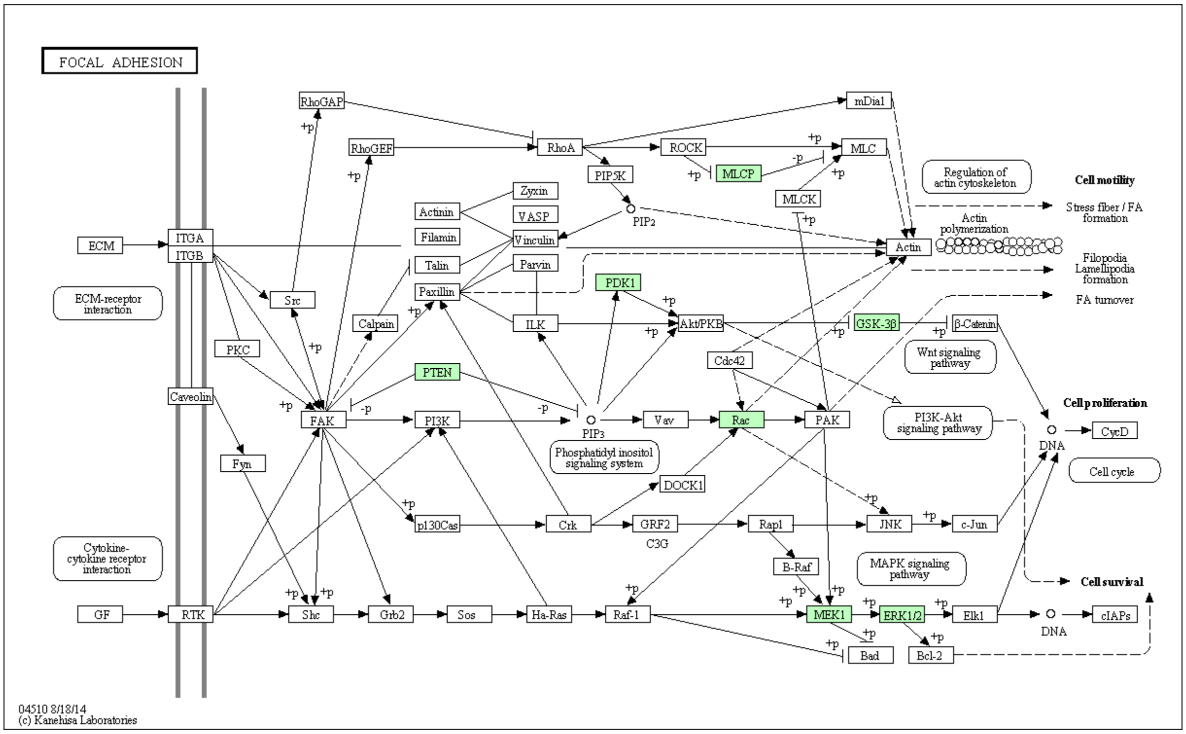
FOCAL ADHESION
ECM
ITGA
ITGB
ECM-receptor interaction
PKC
Caveolin
Fyn
Src
RhoGAP
RhoGEF
Calpain
FAK
PTEN
PI3K
Actinin
Filamin
Talin
Paxillin
Zyxin
VASP
Vinculin
Parvin
ILK
PDK1
Akt/PKB
GSK-3β
β-Catenin
Wnt signaling pathway
RhoA
ROCK
MLCP
PIP5K
PIP2
mDia1
MLC
MLCK
Actin
Actin polymerization
Regulation of actin cytoskeleton
Cell motility
Stress fiber / FA formation
Filopodia Lamellipodia formation
FA turnover
PIP3
Phosphatidyl inositol signaling system
Vav
Rac
Cdc42
PAK
PI3K-Akt signaling pathway
DOCK1
p130Cas
Crk
GRF2
C3G
Rap1
B-Raf
JNK
c-Jun
MAPK signaling pathway
DNA
CycD
Cell proliferation
Cell cycle
Cytokine-cytokine receptor interaction
GF
RTK
Shc
Grb2
Sos
Ha-Ras
Raf-1
MEK1
ERK1/2
Elk1
cIAPs
Bad
Bcl-2
Cell survival
+p
-p
04510 8/18/14
(c) Kanehisa Laboratories

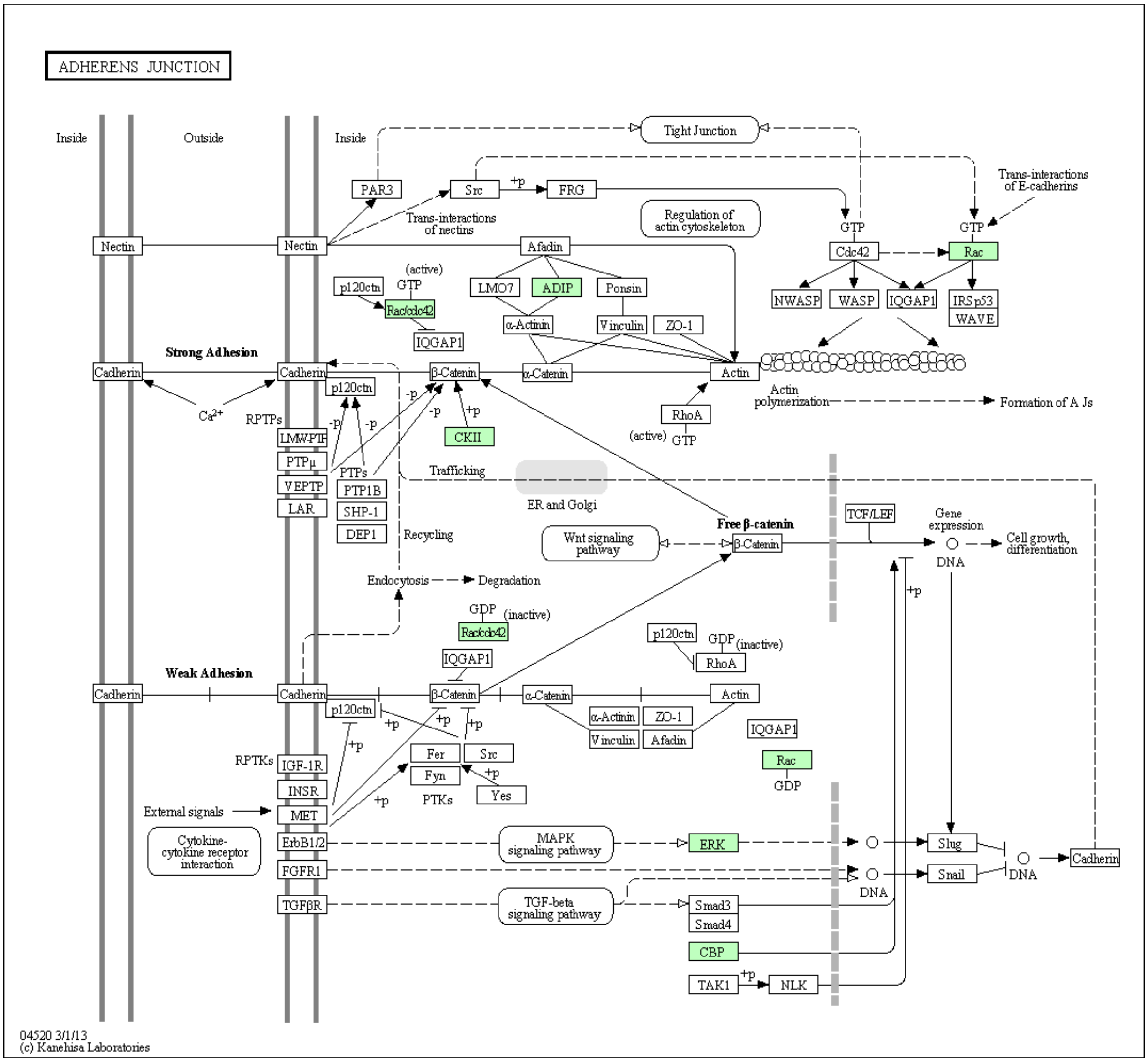

ADHERENS JUNCTION
Inside
Outside
Inside
Tight Junction
PAR3
Src
+p
FRG
Trans-interactions of E-cadherins
Trans-interactions of nectins
Regulation of actin cytoskeleton
GTP
GTP
Nectin
Nectin
Afadin
Cdc42
Rac
(active)
GTP
p120ctn
Rac/cdc42
LMO7
ADIP
Ponsin
NWASP
WASP
IQGAP1
IRSp53
WAVE
α-Actinin
Vinculin
ZO-1
IQGAP1
Strong Adhesion
Cadherin
Cadherin
β-Catenin
α-Catenin
Actin
p120ctn
-p
-p
-p
+p
Ca2+
RPTPs
LMW-PTP
PTPμ
VEPTP
LAR
PTPs
PTP1B
SHP-1
DEP1
CKII
RhoA
(active)
GTP
Actin polymerization
Formation of A Js
Trafficking
ER and Golgi
Recycling
Free β-catenin
TCF/LEF
Gene expression
Wnt signaling pathway
β-Catenin
Cell growth, differentiation
DNA
Endocytosis
Degradation
+p
GDP
(inactive)
Rac/cdc42
p120ctn
GDP
(inactive)
RhoA
IQGAP1
Weak Adhesion
Cadherin
Cadherin
β-Catenin
α-Catenin
Actin
p120ctn
+p
+p
+p
α-Actinin
ZO-1
Vinculin
Afadin
IQGAP1
Rac
GDP
RPTKs
IGF-1R
INSR
MET
+p
Fer
Src
Fyn
+p
Yes
+p
PTKs
External signals
Cytokine-cytokine receptor interaction
ErbB1/2
FGFR1
TGFβR
MAPK signaling pathway
ERK
Slug
Snail
DNA
DNA
Cadherin
TGF-beta signaling pathway
Smad3
Smad4
CBP
TAK1
+p
NLK
04520 3/1/13
(c) Kanehisa Laboratories

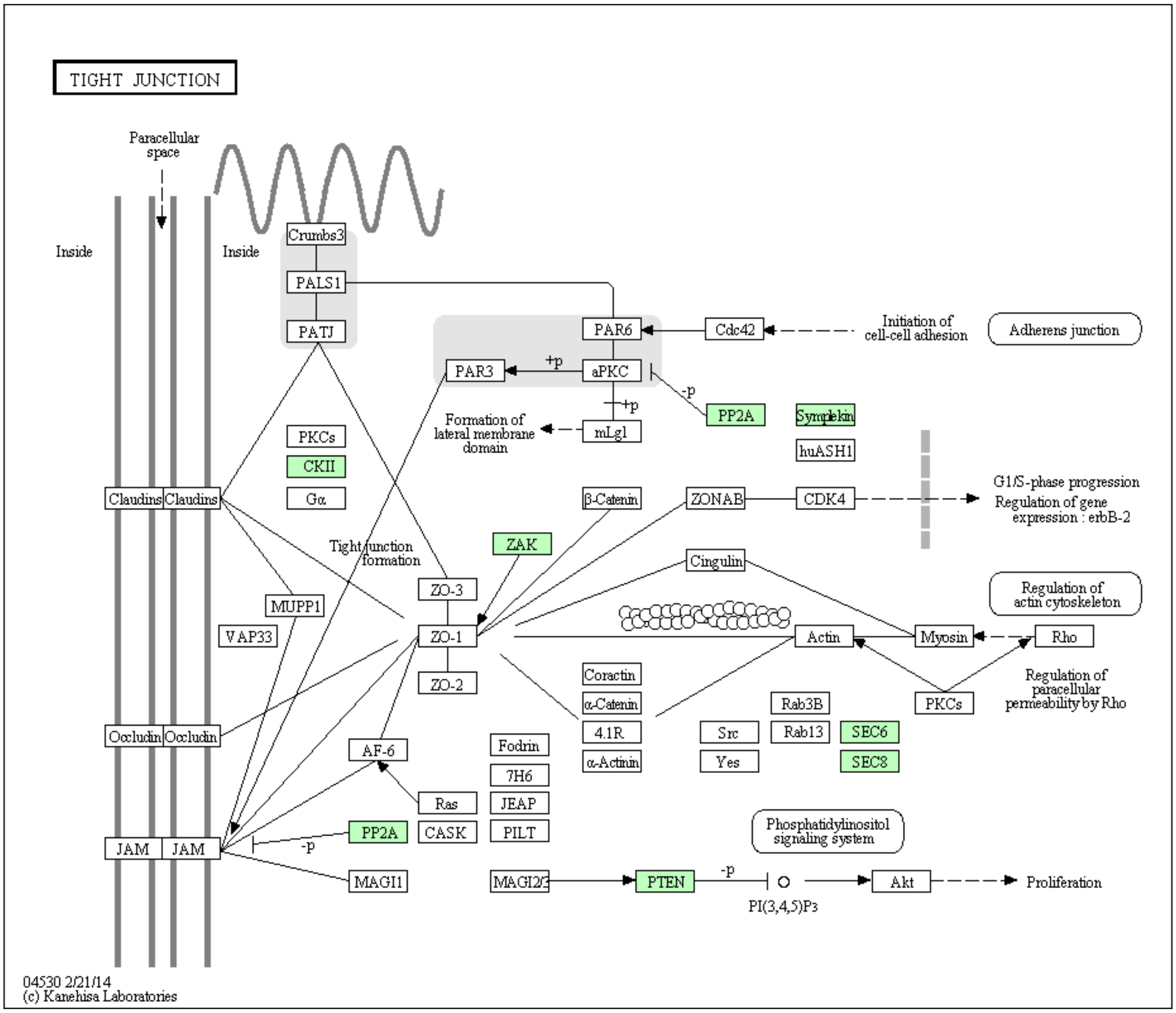
TIGHT JUNCTION
Paracellular space
Inside
Inside
Crumbs3
PALS1
PATJ
PAR6
Cdc42
Initiation of cell-cell adhesion
Adherens junction
PAR3
+p
aPKC
-p
+p
PP2A
Symplekin
huASH1
Formation of lateral membrane domain
mLgl
PKCs
CKII
Gα
Claudins
Claudins
β-Catenin
ZONAB
CDK4
G1/S-phase progression
Regulation of gene expression : erbB-2
ZAK
Tight junction formation
Cingulin
Regulation of actin cytoskeleton
ZO-3
MUPP1
VAP33
ZO-1
Actin
Myosin
Rho
ZO-2
Coractin
α-Catenin
4.1R
α-Actinin
Rab3B
Rab13
PKCs
Regulation of paracellular permeability by Rho
Src
Yes
SEC6
SEC8
Occludin
Occludin
AF-6
Fodrin
7H6
Ras
JEAP
CASK
PILT
Phosphatidylinositol signaling system
JAM
JAM
-p
PP2A
MAGI1
MAGI2/3
PTEN
-p
Akt
Proliferation
PI(3,4,5)P3
04530 2/21/14
(c) Kanehisa Laboratories

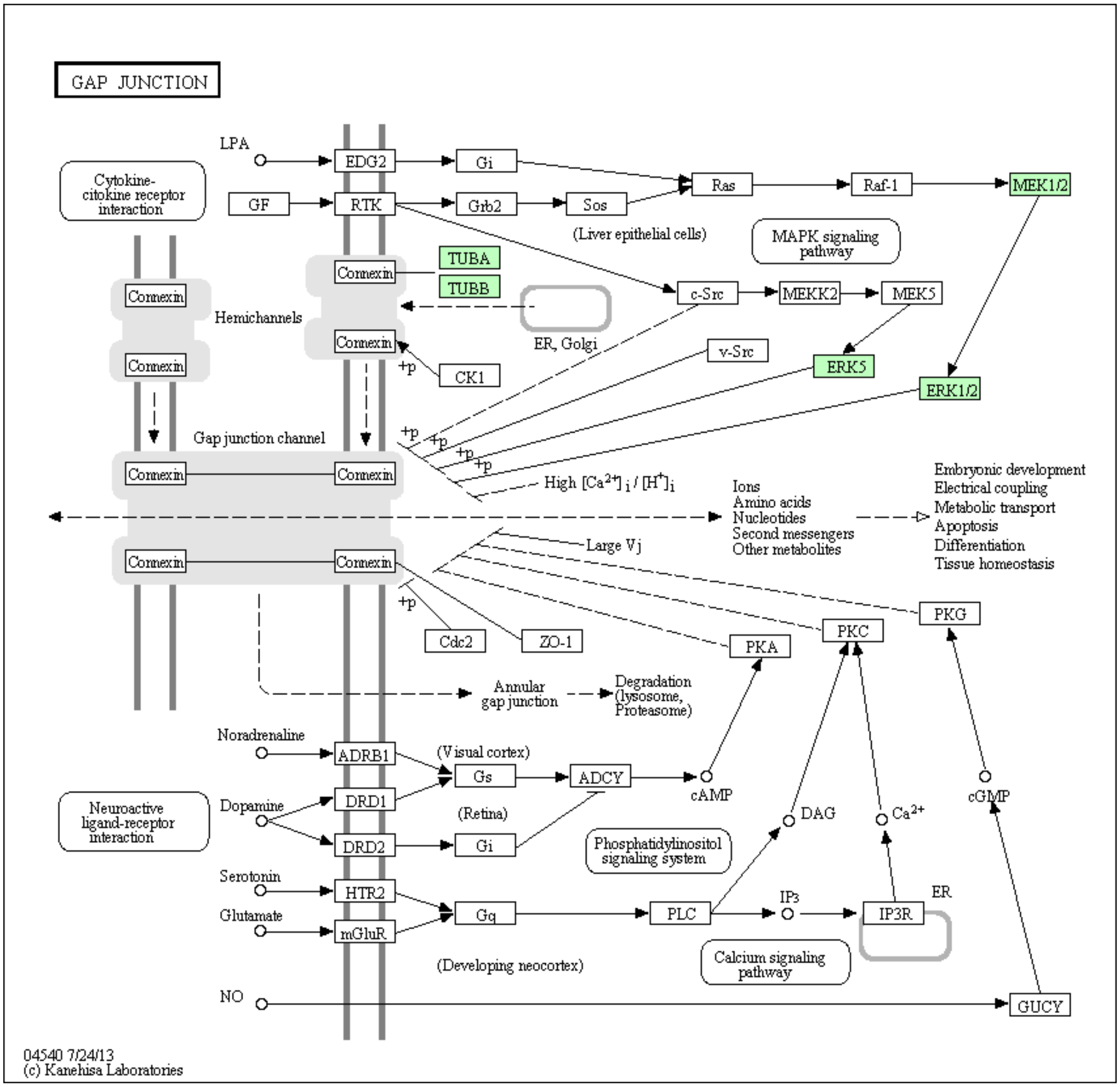
GAP JUNCTION
LPA
EDG2
Gi
Ras
Raf-1
MEK1/2
Cytokine-cytokine receptor interaction
GF
RTK
Grb2
Sos
(Liver epithelial cells)
MAPK signaling pathway
TUBA
TUBB
Connexin
Hemichannels
c-Src
MEKK2
MEK5
ER, Golgi
v-Src
ERK5
CK1
+p
ERK1/2
Gap junction channel
High [Ca2+]i / [H+]i
Ions
Amino acids
Nucleotides
Second messengers
Other metabolites
Embryonic development
Electrical coupling
Metabolic transport
Apoptosis
Differentiation
Tissue homeostasis
Large Vj
PKG
PKC
PKA
Cdc2
ZO-1
Annular gap junction
Degradation (lysosome, Proteasome)
Noradrenaline
ADRB1
(Visual cortex)
Gs
ADCY
cAMP
Dopamine
DRD1
DRD2
(Retina)
Neuroactive ligand-receptor interaction
Phosphatidylinositol signaling system
DAG
Ca2+
cGMP
Serotonin
HTR2
Glutamate
mGluR
Gq
PLC
IP3
IP3R
ER
Calcium signaling pathway
(Developing neocortex)
NO
GUCY
04540 7/24/13
(c) Kanehisa Laboratories

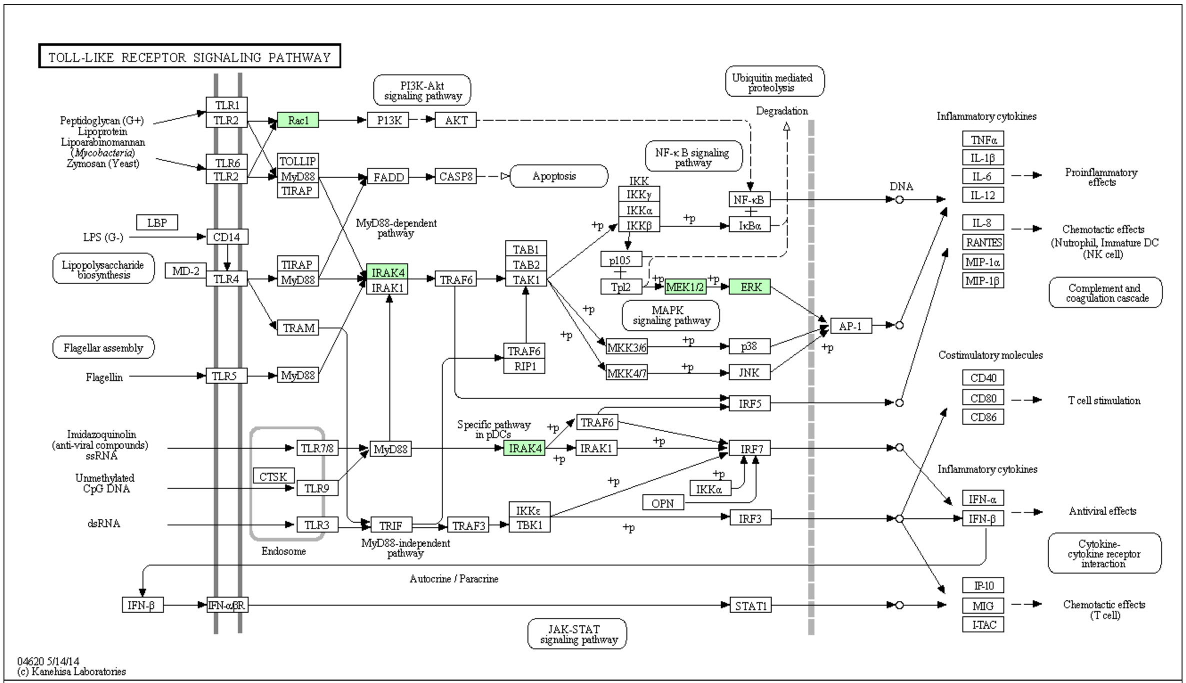
TOLL-LIKE RECEPTOR SIGNALING PATHWAY
Peptidoglycan (G+)
Lipoprotein
Lipoarabinomannan
(Mycobacteria)
Zymosan (Yeast)
LBP
LPS (G-)
Lipopolysaccharide biosynthesis
MD-2
Flagellar assembly
Flagellin
Imidazoquinolin (anti-viral compounds) ssRNA
Unmethylated CpG DNA
dsRNA
TLR1
TLR2
TLR6
CD14
TLR4
TLR5
TLR7/8
TLR9
TLR3
CTSK
Endosome
Rac1
TOLLIP
MyD88
TIRAP
TRAM
PI3K-Akt signaling pathway
PI3K
AKT
FADD
CASP8
Apoptosis
MyD88-dependent pathway
IRAK4
IRAK1
TRAF6
TAB1
TAB2
TAK1
RIP1
TRIF
TRAF3
IKKε
TBK1
MyD88-independent pathway
Specific pathway in pDCs
OPN
IKKα
IRF7
IRF3
IRF5
IKK
IKKγ
IKKβ
NF-κB signaling pathway
NF-κB
IκBα
p105
Tpl2
MEK1/2
ERK
MAPK signaling pathway
MKK3/6
MKK4/7
p38
JNK
AP-1
Ubiquitin mediated proteolysis
Degradation
DNA
+p
Inflammatory cytokines
TNFα
IL-1β
IL-6
IL-12
Proinflammatory effects
IL-8
RANTES
MIP-1α
MIP-1β
Chemotactic effects (Nutrophil, Immature DC (NK cell)
Complement and coagulation cascade
Costimulatory molecules
CD40
CD80
CD86
T cell stimulation
IFN-α
IFN-β
Antiviral effects
Cytokine-cytokine receptor interaction
IP-10
MIG
I-TAC
Chemotactic effects (T cell)
Autocrine / Paracrine
IFN-αβR
STAT1
JAK-STAT signaling pathway
04620 5/14/14
(c) Kanehisa Laboratories

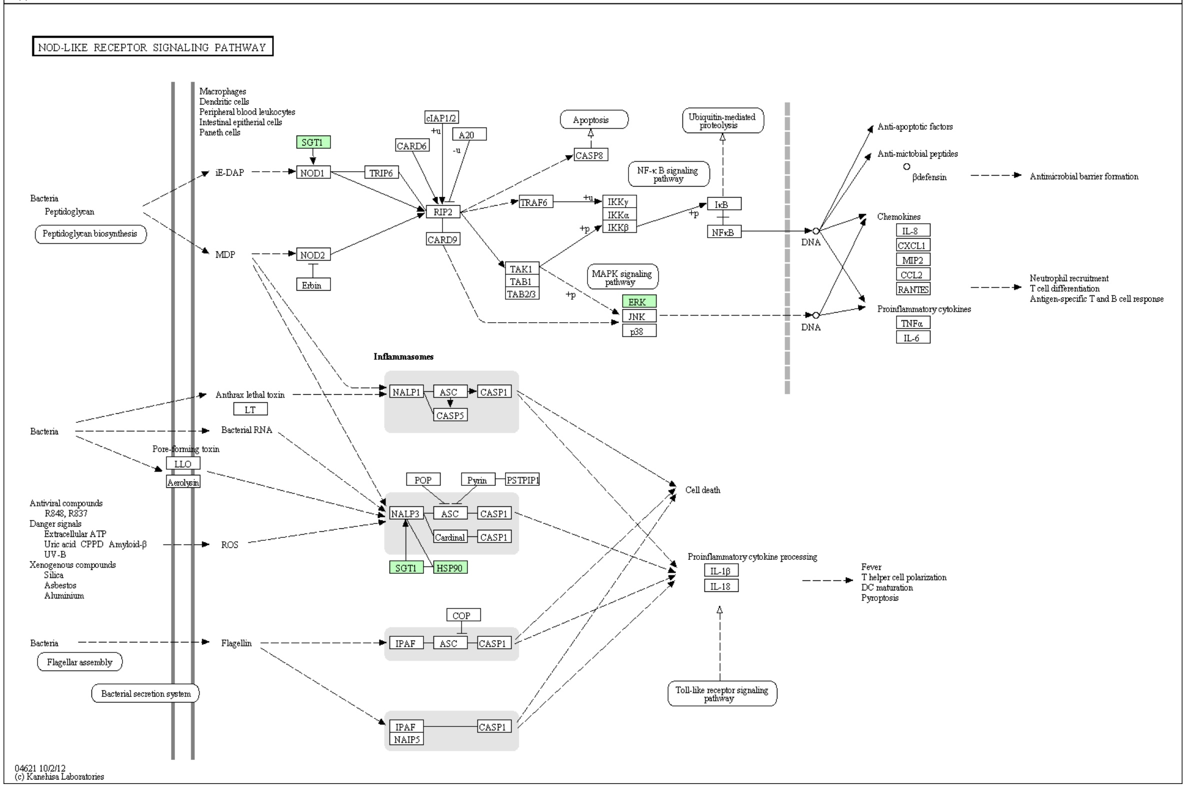
NOD-LIKE RECEPTOR SIGNALING PATHWAY
Macrophages
Dendritic cells
Peripheral blood leukocytes
Intestinal epithelial cells
Paneth cells
Bacteria
Peptidoglycan
Peptidoglycan biosynthesis
iE-DAP
MDP
SGT1
NOD1
NOD2
Erbin
TRIP6
CARD6
cIAP1/2
A20
+u
-u
RIP2
CARD9
CASP8
Apoptosis
TRAF6
IKKγ
IKKα
IKKβ
NF-κB signaling pathway
IκB
NFκB
Ubiquitin-mediated proteolysis
TAK1
TAB1
TAB2/3
MAPK signaling pathway
ERK
JNK
p38
DNA
Anti-apoptotic factors
Anti-microbial peptides
βdefensin
Antimicrobial barrier formation
Chemokines
IL-8
CXCL1
MIP2
CCL2
RANTES
Proinflammatory cytokines
TNFα
IL-6
Neutrophil recruitment
T cell differentiation
Antigen-specific T and B cell response
Inflammasomes
Anthrax lethal toxin
LT
Bacterial RNA
Pore-forming toxin
LLO
Aerolysin
NALP1
ASC
CASP1
CASP5
POP
Pyrin
PSTPIP1
NALP3
Cardinal
HSP90
Antiviral compounds
R848, R837
Danger signals
Extracellular ATP
Uric acid CPPD Amyloid-β
UV-B
Xenogenous compounds
Silica
Asbestos
Aluminium
ROS
Flagellin
Flagellar assembly
Bacterial secretion system
COP
IPAF
NAIP5
Cell death
Proinflammatory cytokine processing
IL-1β
IL-18
Fever
T helper cell polarization
DC maturation
Pyroptosis
Toll-like receptor signaling pathway
04621 10/2/12
(c) Kanehisa Laboratories

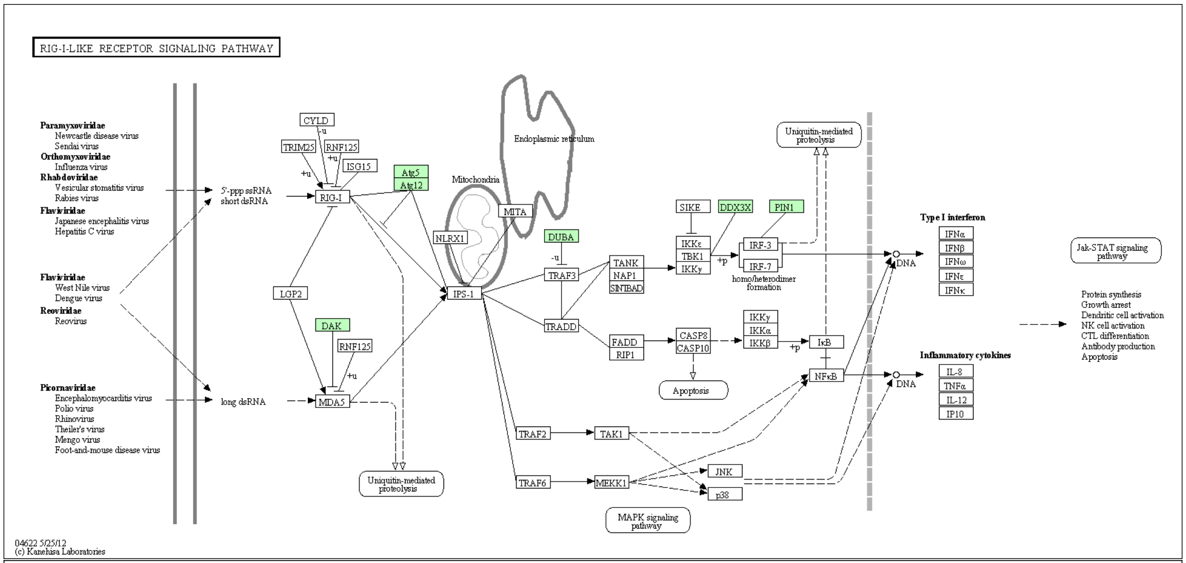
RIG-I-LIKE RECEPTOR SIGNALING PATHWAY
Paramyxoviridae
Newcastle disease virus
Sendai virus
Orthomyxoviridae
Influenza virus
Rhabdoviridae
Vesicular stomatitis virus
Rabies virus
Flaviviridae
Japanese encephalitis virus
Hepatitis C virus
Flaviviridae
West Nile virus
Dengue virus
Reoviridae
Reovirus
Picornaviridae
Encephalomyocarditis virus
Polio virus
Rhinovirus
Theiler's virus
Mengo virus
Foot-and-mouth disease virus
5'-ppp ssRNA
short dsRNA
long dsRNA
CYLD
TRIM25
RNF125
ISG15
Atg5
Atg12
RIG-I
LGP2
DAK
RNF125
MDA5
Mitochondria
Endoplasmic reticulum
NLRX1
MITA
IPS-1
DUBA
TRAF3
TRADD
TANK
NAP1
SINTBAD
FADD
RIP1
CASP8
CASP10
Apoptosis
SIKE
DDX3X
PIN1
IKKε
TBK1
IKKγ
IRF-3
IRF-7
homo/heterodimer formation
IKKγ
IKKα
IKKβ
IκB
NFκB
Uniquitin-mediated proteolysis
TRAF2
TAK1
TRAF6
MEKK1
JNK
p38
MAPK signaling pathway
Uniquitin-mediated proteolysis
DNA
DNA
Type I interferon
IFNα
IFNβ
IFNω
IFNε
IFNκ
Inflammatory cytokines
IL-8
TNFα
IL-12
IP10
Jak-STAT signaling pathway
Protein synthesis
Growth arrest
Dendritic cell activation
NK cell activation
CTL differentiation
Antibody production
Apoptosis
04622 5/25/12
(c) Kanehisa Laboratories

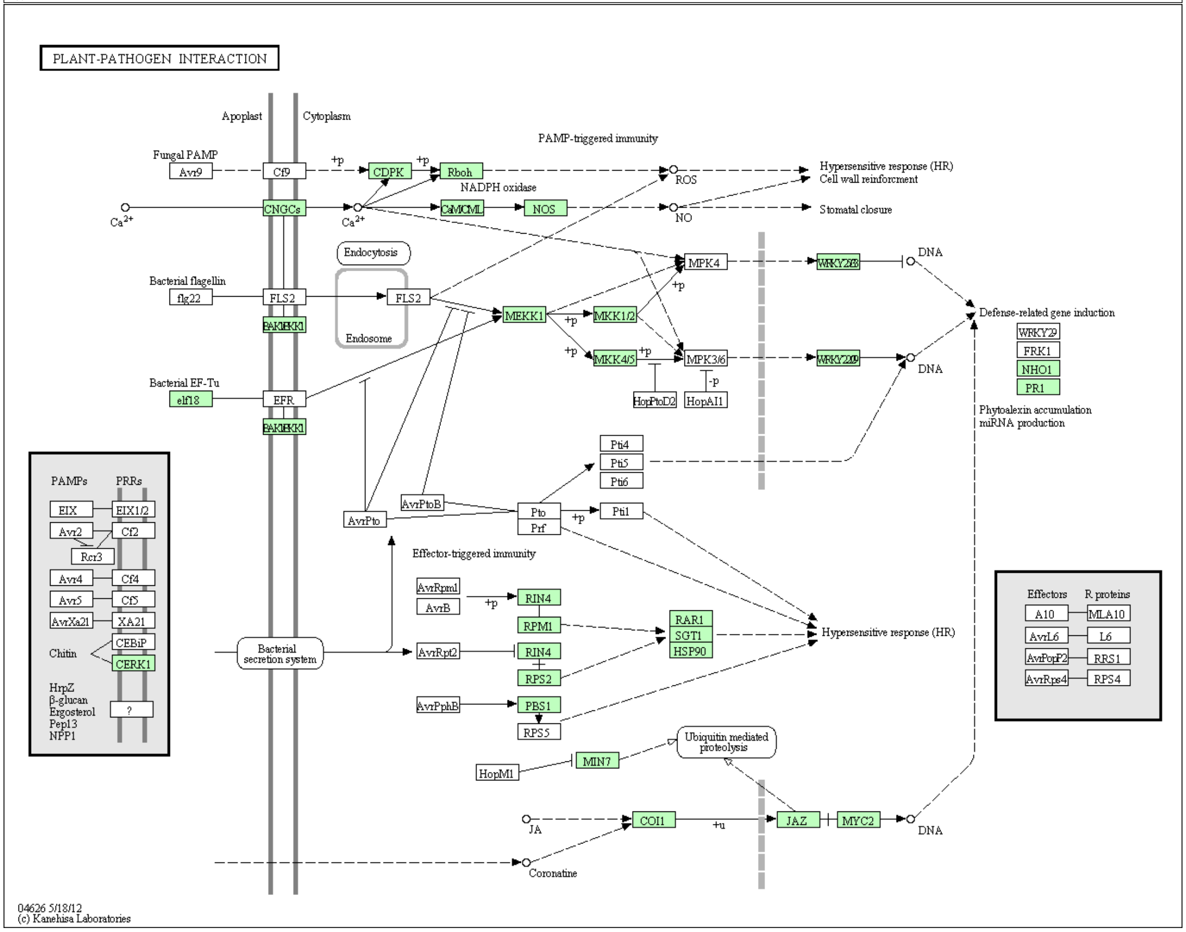
PLANT-PATHOGEN INTERACTION
Apoplast
Cytoplasm
PAMP-triggered immunity
Fungal PAMP
Avr9
Cf9
CDPK
Rboh
NADPH oxidase
ROS
Hypersensitive response (HR)
Cell wall reinforcement
Ca2+
CNGCs
Ca2+
CaM/CML
NOS
NO
Stomatal closure
Endocytosis
Endosome
Bacterial flagellin
flg22
FLS2
FLS2
BAK1/BKK1
MEKK1
MKK1/2
MKK4/5
MPK4
MPK3/6
WRKY25/33
WRKY22/29
DNA
DNA
Defense-related gene induction
WRKY29
FRK1
NHO1
PR1
Phytoalexin accumulation
miRNA production
HopPtoD2
HopAI1
Bacterial EF-Tu
elf18
EFR
BAK1/BKK1
Pti4
Pti5
Pti6
AvrPtoB
AvrPto
Pto
Prf
Pti1
Effector-triggered immunity
AvrRpm1
AvrB
RIN4
RPM1
RAR1
SGT1
HSP90
Hypersensitive response (HR)
Bacterial secretion system
AvrRpt2
RIN4
RPS2
AvrPphB
PBS1
RPS5
Ubiquitin mediated proteolysis
HopM1
MIN7
JA
COI1
JAZ
MYC2
DNA
Coronatine
PAMPs
PRRs
EIX
EIX1/2
Avr2
Cf2
Rcr3
Avr4
Cf4
Avr5
Cf5
AvrXa21
XA21
Chitin
CEBiP
CERK1
HrpZ
β-glucan
Ergosterol
Pep13
NPP1
?
Effectors
R proteins
A10
MLA10
AvrL6
L6
AvrPphF2
RRS1
AvrRps4
RPS4
04626 5/18/12
(c) Kanehisa Laboratories

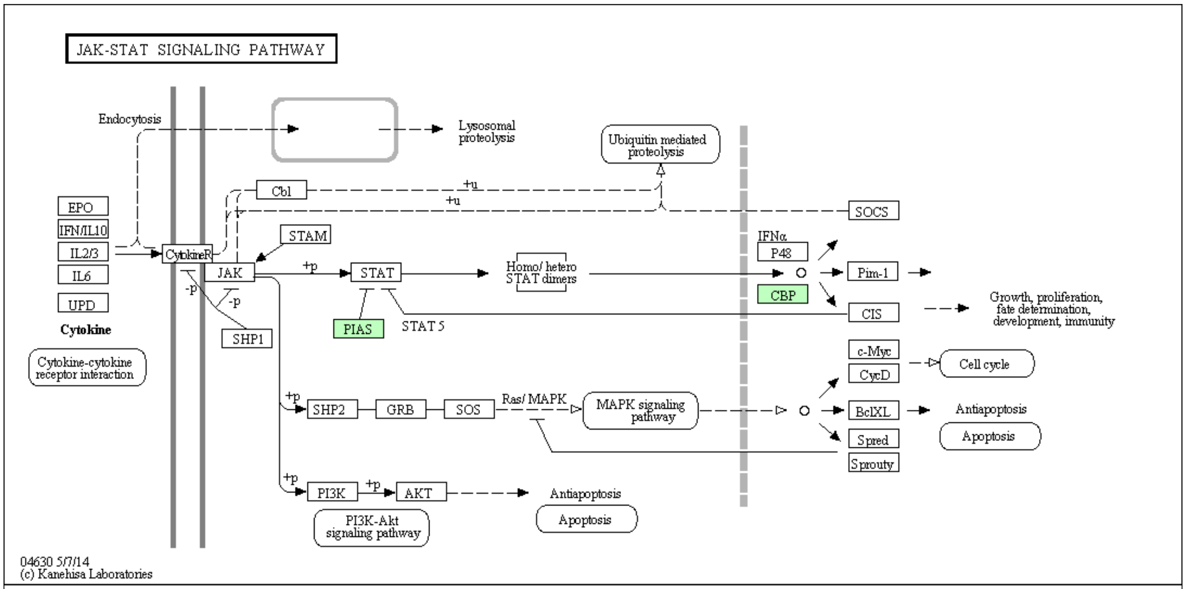
JAK-STAT SIGNALING PATHWAY
Endocytosis
Lysosomal proteolysis
Ubiquitin mediated proteolysis
Cbl
+u
+u
SOCS
EPO
IFN/IL10
IL2/3
IL6
UPD
Cytokine
Cytokine-cytokine receptor interaction
CytokineR
STAM
JAK
+p
STAT
Homo/ hetero STAT dimers
IFNα
P48
CBP
Pim-1
CIS
Growth, proliferation, fate determination, development, immunity
-p
-p
SHP1
PIAS
STAT 5
c-Myc
CycD
Cell cycle
+p
SHP2
GRB
SOS
Ras/ MAPK
MAPK signaling pathway
BclXL
Spred
Sprouty
Antiapoptosis
Apoptosis
+p
PI3K
+p
AKT
Antiapoptosis
Apoptosis
PI3K-Akt signaling pathway
04630 5/7/14
(c) Kanehisa Laboratories

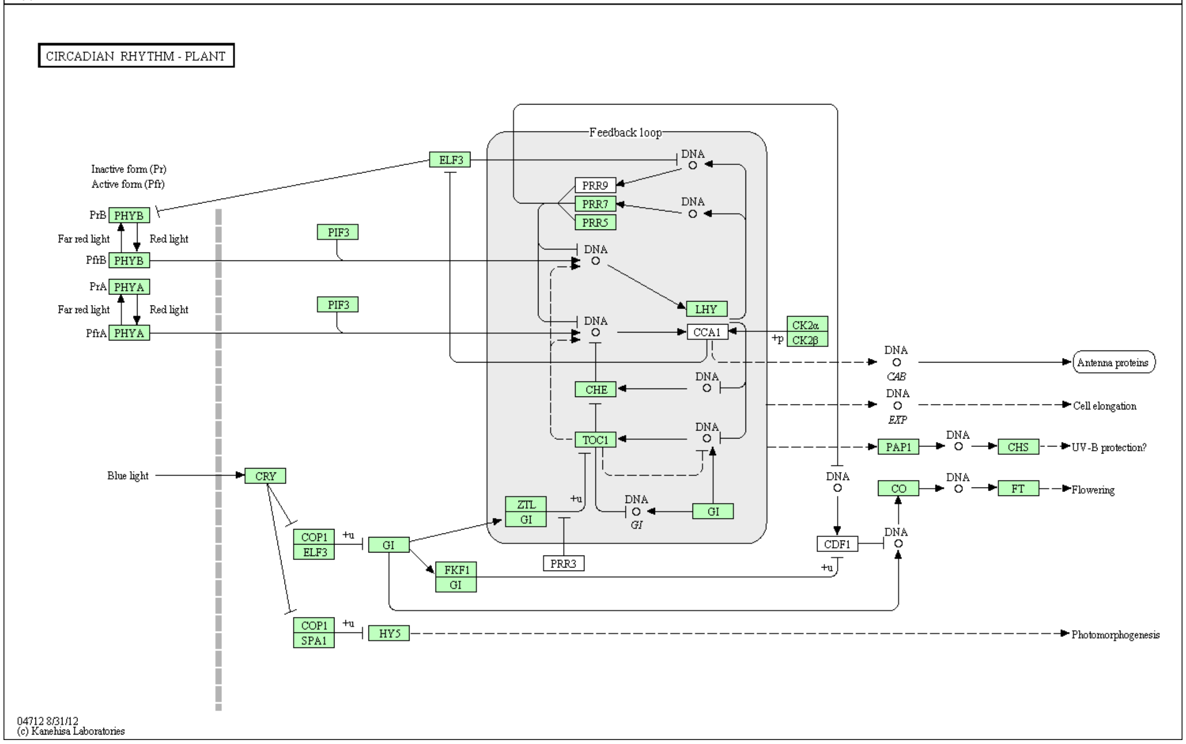
CIRCADIAN RHYTHM - PLANT
Feedback loop
Inactive form (Pr)
Active form (Pfr)
ELF3
DNA
PRR9
PRR7
PRR5
DNA
PrB
PHYB
Far red light
Red light
PfrB
PHYB
PIF3
DNA
PrA
PHYA
Far red light
Red light
PfrA
PHYA
PIF3
LHY
DNA
CCA1
CK2α
CK2β
+p
DNA
CAB
Antenna proteins
CHE
DNA
DNA
EXP
Cell elongation
TOC1
DNA
DNA
PAP1
DNA
CHS
UV-B protection?
Blue light
CRY
DNA
CO
DNA
FT
Flowering
ZTL
GI
+u
DNA
GI
GI
COP1
ELF3
+u
GI
CDF1
DNA
FKF1
GI
PRR3
+u
COP1
SPA1
+u
HY5
Photomorphogenesis
04712 8/31/12
(c) Kanehisa Laboratories

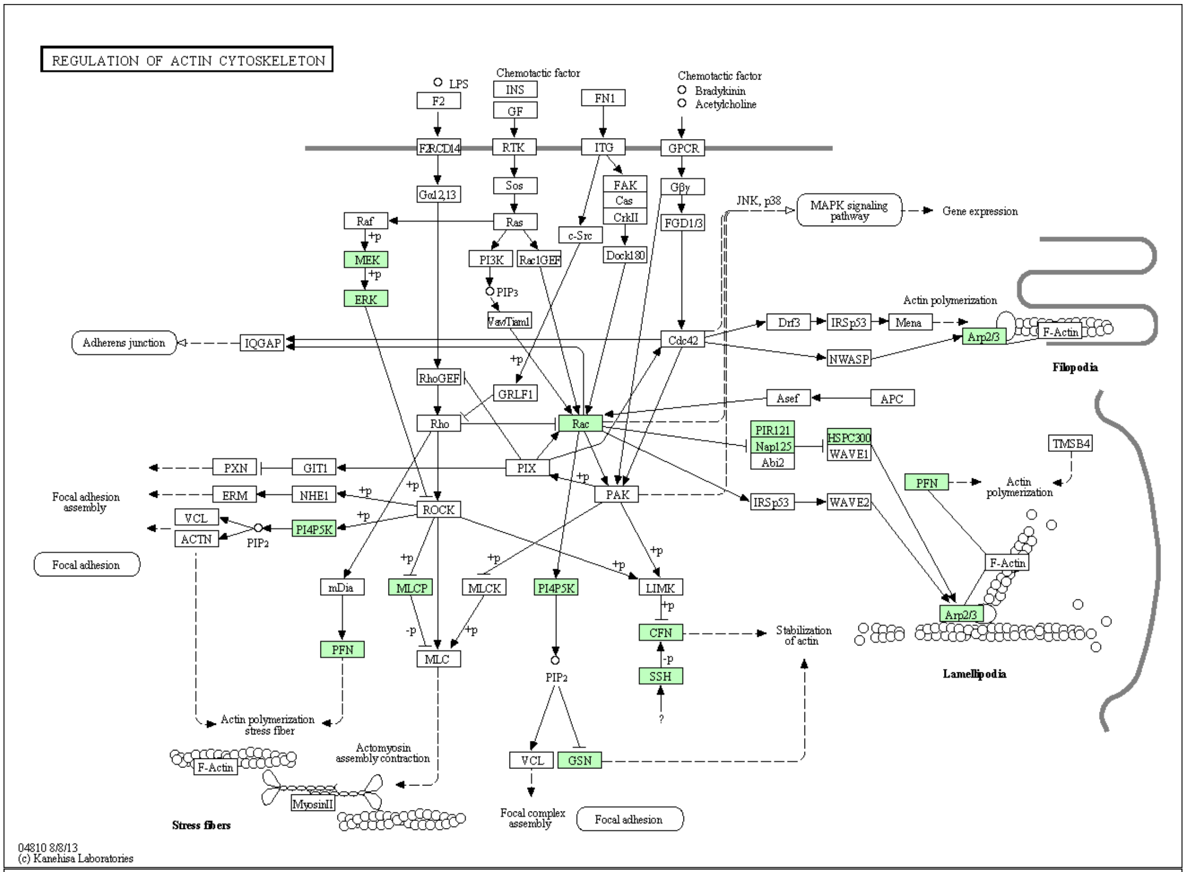
REGULATION OF ACTIN CYTOSKELETON
LPS
F2
Chemotactic factor
INS
GF
FN1
Chemotactic factor
Bradykinin
Acetylcholine
F2RCD14
RTK
ITG
GPCR
Sos
FAK
Cas
CrkII
Gβγ
Gα12,13
Raf
Ras
c-Src
FGD1/3
Dock180
MEK
ERK
PI3K
Rac1GEF
PIP3
Vav/Tiam1
JNK, p38
MAPK signaling pathway
Gene expression
Actin polymerization
Drf3
IRSp53
Mena
Arp2/3
F-Actin
Filopodia
Adherens junction
IQGAP
Cdc42
NWASP
RhoGEF
GRLF1
Asef
APC
Rho
Rac
PIR121
Nap125
Abi2
HSPC300
WAVE1
TMSB4
PXN
GIT1
PIX
PAK
PFN
Actin polymerization
Focal adhesion assembly
ERM
NHE1
IRSp53
WAVE2
VCL
ROCK
ACTN
PIP2
PI4P5K
F-Actin
Focal adhesion
mDia
MLCP
MLCK
PI4P5K
LIMK
Arp2/3
PFN
MLC
CFN
Stabilization of actin
SSH
Lamellipodia
PIP2
Actin polymerization stress fiber
Actomyosin assembly contraction
VCL
GSN
F-Actin
MyosinII
Focal complex assembly
Focal adhesion
Stress fibers
04810 8/8/13
(c) Kanehisa Laboratories

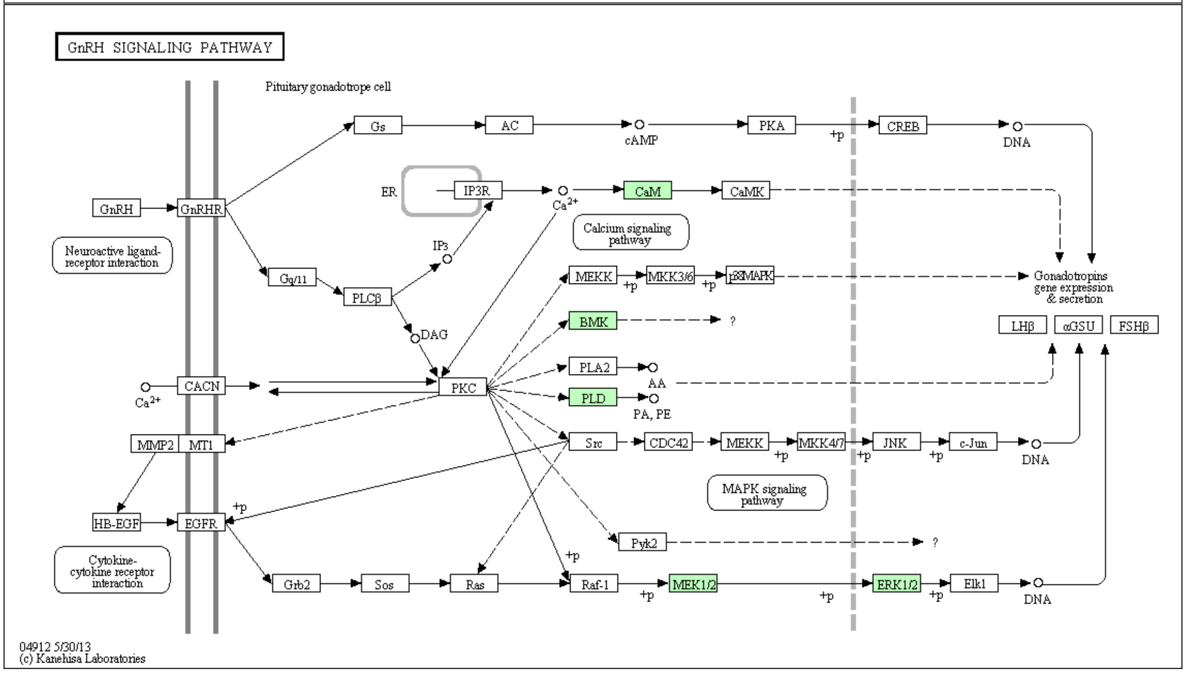
GnRH SIGNALING PATHWAY
Pituitary gonadotrope cell
Gs
AC
cAMP
PKA
CREB
DNA
ER
IP3R
Ca2+
CaM
CaMK
GnRH
GnRHR
Calcium signaling pathway
Neuroactive ligand-receptor interaction
IP3
Gq/11
PLCβ
MEKK
MKK3/6
p38MAPK
Gonadotropins gene expression & secretion
DAG
BMK
LHβ
αGSU
FSHβ
PLA2
AA
CACN
PKC
Ca2+
PLD
PA, PE
MMP2
MT1
Src
CDC42
MEKK
MKK4/7
JNK
c-Jun
DNA
MAPK signaling pathway
HB-EGF
EGFR
Pyk2
Cytokine-cytokine receptor interaction
Grb2
Sos
Ras
Raf-1
MEK1/2
ERK1/2
Elk1
DNA
04912 5/30/13
(c) Kanehisa Laboratories

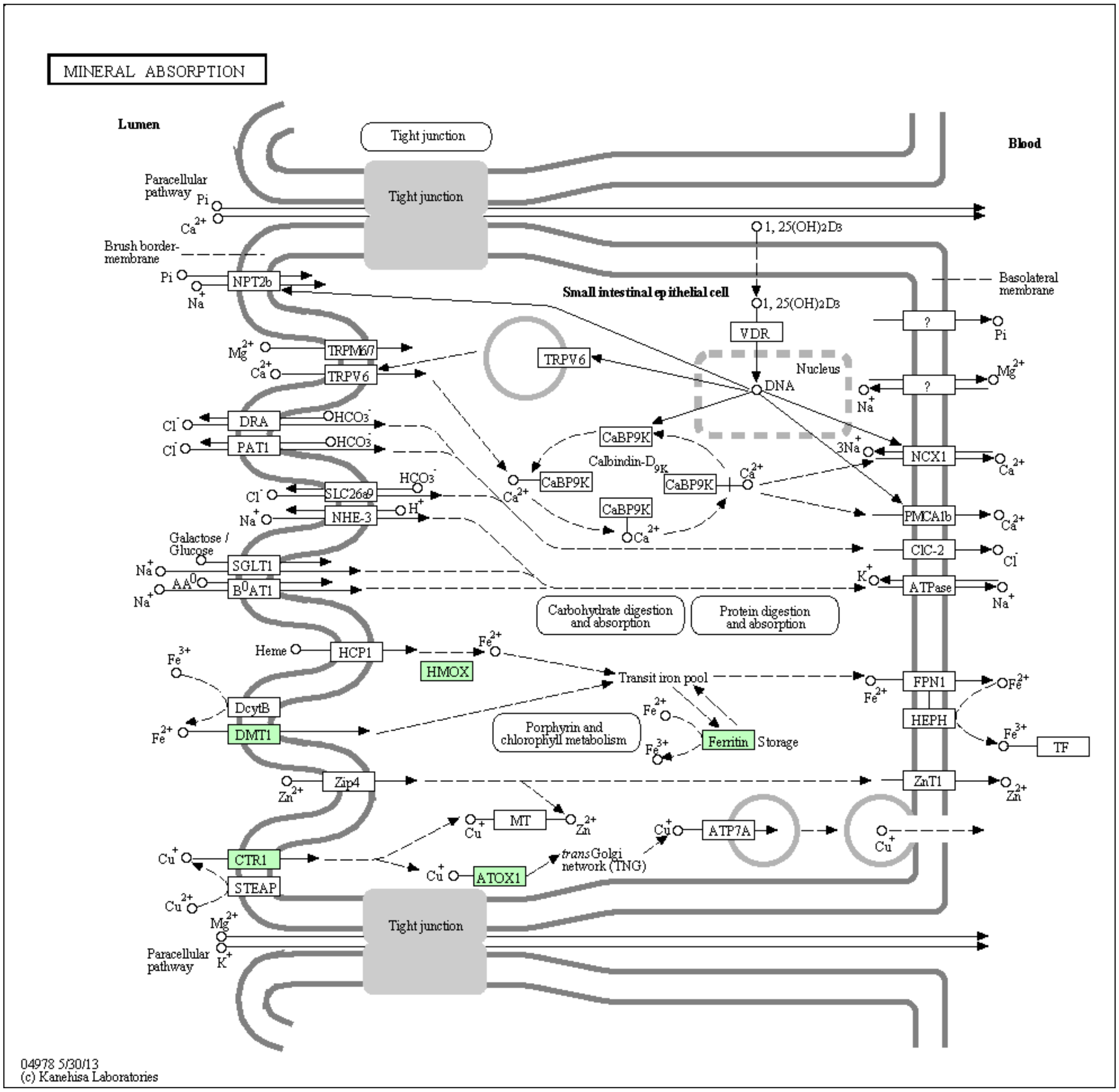
MINERAL ABSORPTION
Lumen
Blood
Tight junction
Paracellular pathway
Brush border-membrane
Basolateral membrane
Small intestinal epithelial cell
Nucleus
DNA
VDR
1, 25(OH)2D3
NPT2b
TRPM6/7
TRPV6
DRA
PAT1
SLC26a9
NHE-3
SGLT1
B0AT1
Galactose / Glucose
HCP1
Heme
HMOX
DcytB
DMT1
Zip4
CTR1
STEAP
MT
ATOX1
ATP7A
trans Golgi network (TNG)
CaBP9K
Calbindin-D9K
Carbohydrate digestion and absorption
Protein digestion and absorption
Porphyrin and chlorophyll metabolism
Transit iron pool
Ferritin
Storage
NCX1
PMCA1b
ClC-2
ATPase
FPN1
HEPH
TF
ZnT1
?
04978 5/30/13
(c) Kanehisa Laboratories
