# Supplementary figures and images for: De novo assembly and functional annotation of Myrciaria dubia fruit transcriptome reveals multiple metabolic pathways for L-ascorbic acid biosynthesis
Source: BMC Genomics. 2015 Nov 24;16:997. doi: 10.1186/s12864-015-2225-6 (PMC4658800; doi:10.1186/s12864-015-2225-6)

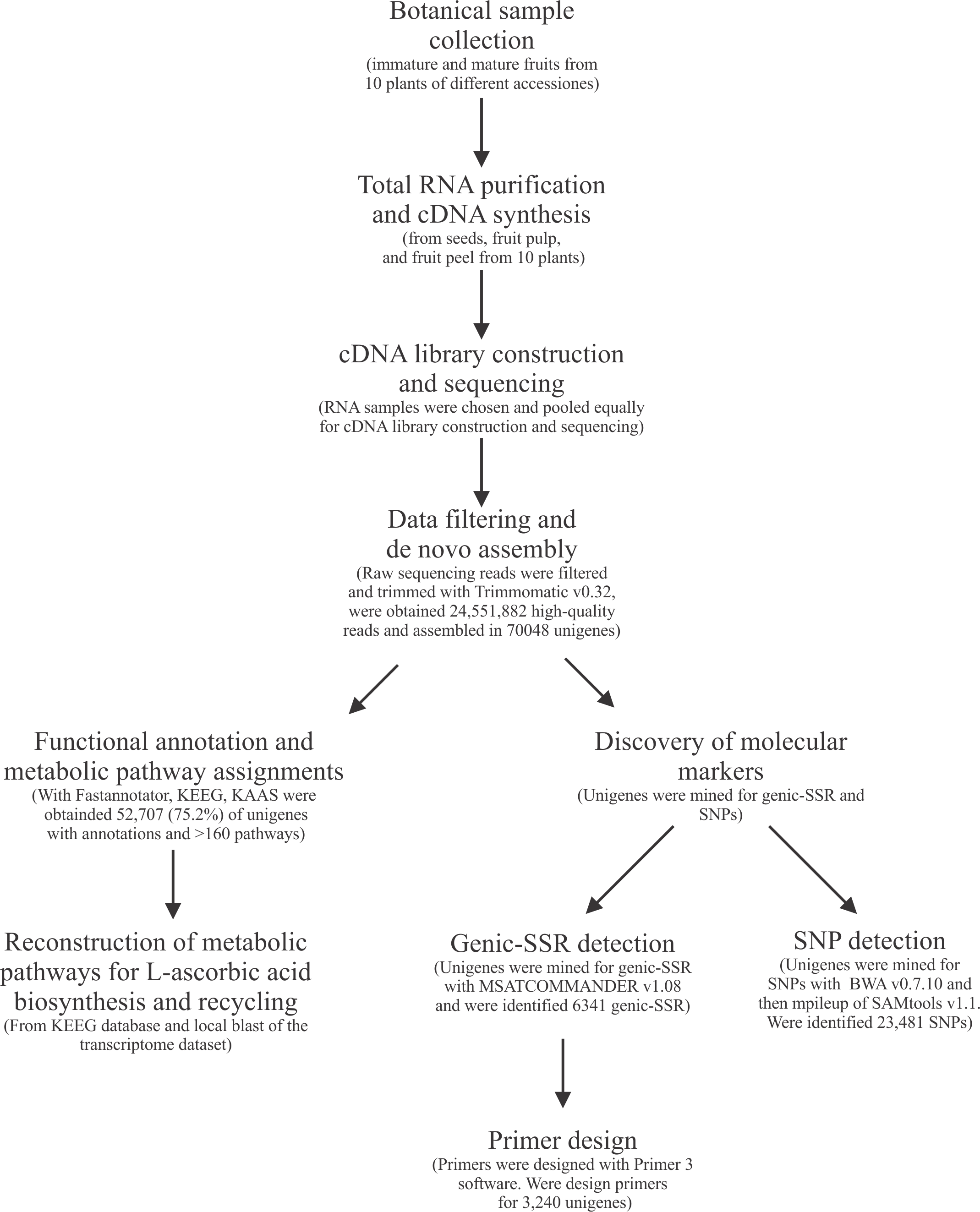

Supplement: Additional file 7: Figure S2. — Flow chart of methods used. (PNG 495 kb) [file 12864_2015_2225_MOESM7_ESM.png]
